# Supplementary material for: Shade Effect on Phenology, Fruit Yield, and Phenolic Content of Two Wild Blueberry Species in Northwestern Ontario, Canada
Source: Plants (Basel). 2023 Dec 7;12(24):4099. doi: 10.3390/plants12244099 (PMC10747682; doi:10.3390/plants12244099)
Supplement: Supplementary file 1 [file plants-12-04099-s001.zip › plants-2729099-supplementary.pdf]

Supplementary Materials

Figure S1: Vegetative phenological stages of *Vaccinium* sp. (Fournier *et al*., 2020).

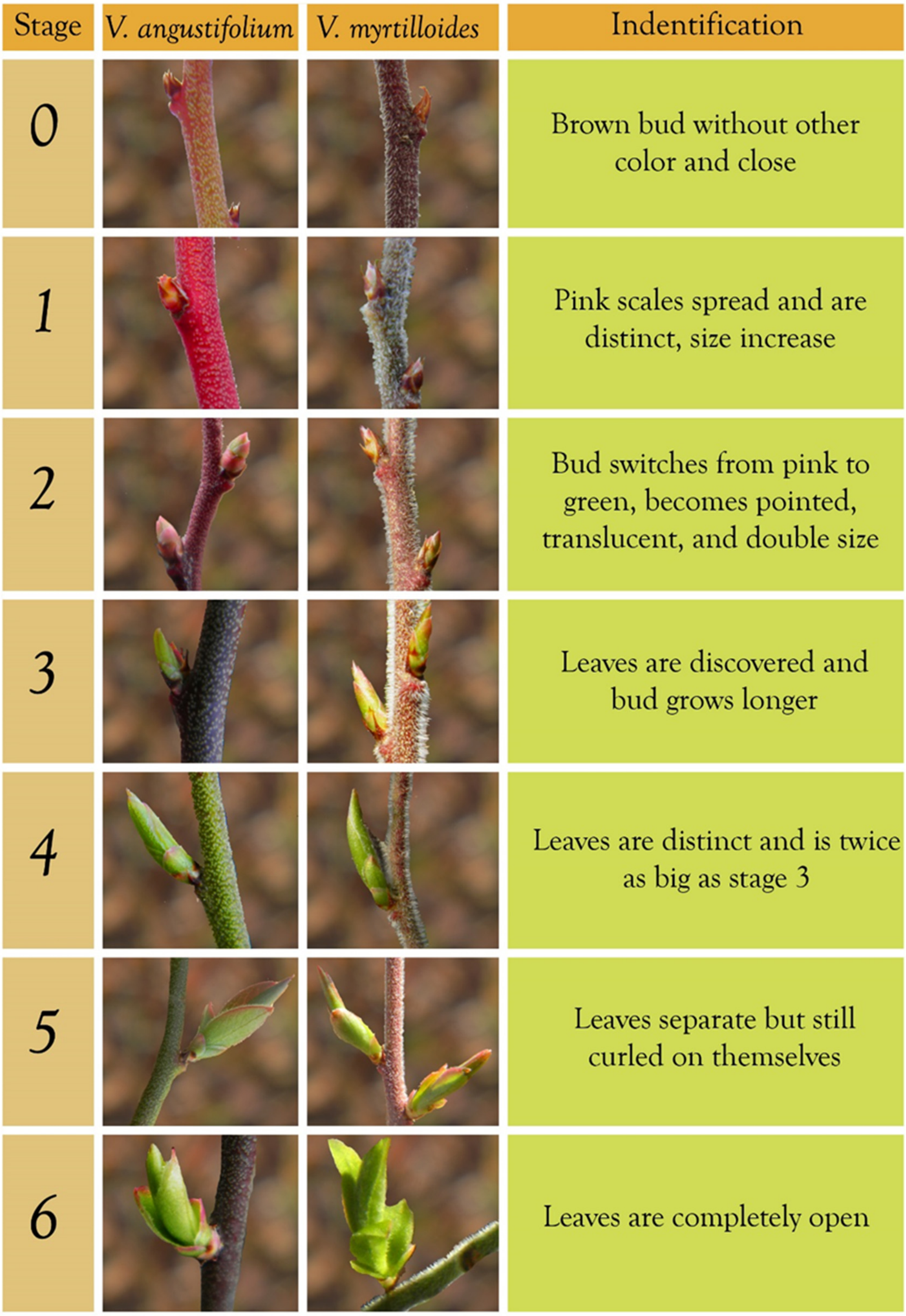

Figure S2: Floral phenological stages of *Vaccinium* sp. (Fournier *et al*., 2020).

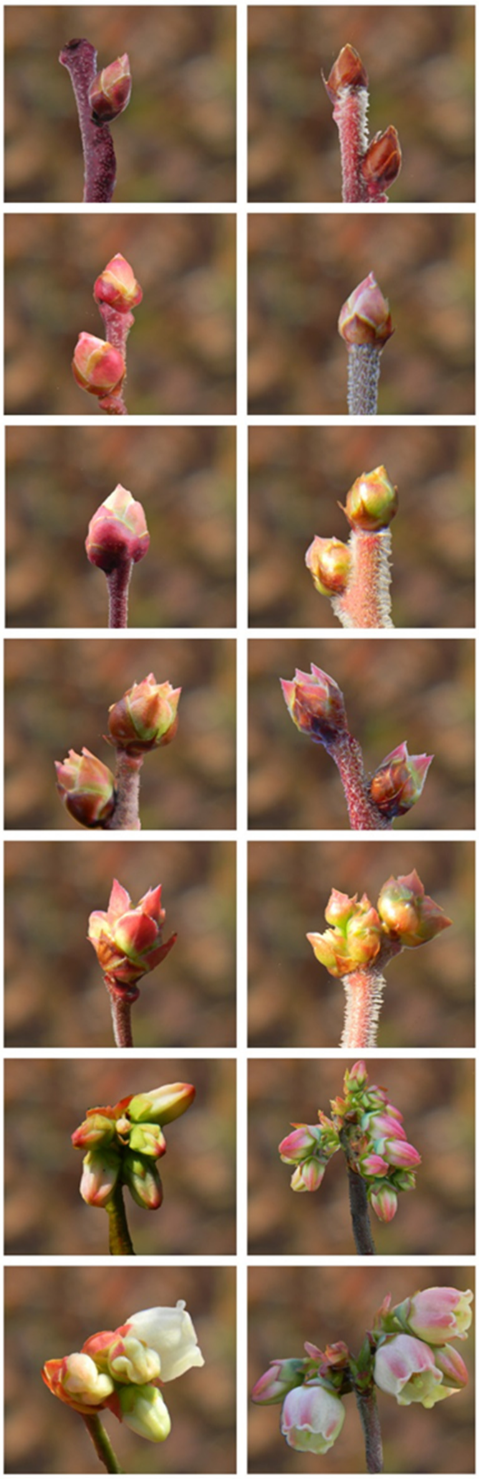

Stage	*V. angustifolium*	*V. myrtilloides*	Indentification
0			Brown bud, small, without other colors and close
1			Pink coloration appers with distinction between scales and start to swell
2			Colored scales, size a third larger that stage 1
3			Bud starts to open, increase in size
4			Bud completely open
5			Distinction between sepals and petals of flowers
6			First flower open

Figure S3: Fruit phenological stages of *Vaccinium* sp. (Fournier *et al*., 2020).

Stage	*Vaccinium sp.*	Indentification
7*	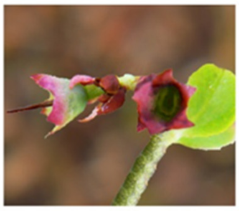	Petals of flowers have fallen, the underside of the sepals stays round and not swollen
8	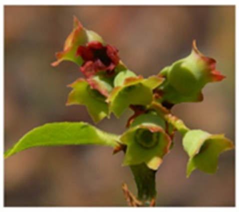	The underside of the sepals swells but does not exceed the width of the calix
9	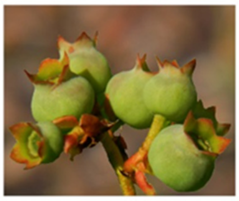	The underside of the sepals exceed the width of the calix but the fruit stays green
10	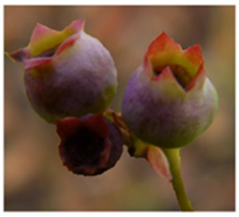	Fruit is coloured from pink to purple or not completely blue
11	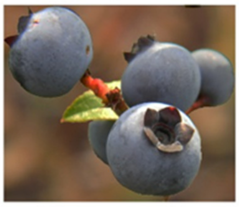	Fruit is blue and ripe

**Development stages of fruit follow development stages of flower.*

Table S1: Phenological data from tagged vegetative and reproductive shoots of *V. myrtilloides*.

	V. myrtilloides (0% Shade)							*V. myrtilloides (30% Shade)*							*V. myrtilloides (50% Shade)*							*V. myrtilloides (80% Shade)*						
Date	Pot #	A(V)	B(V)	C(V)	D(R)	E(R)	F(R)	Pot #	A(V)	B(V)	C(V)	D(R)	E(R)	F(R)	Pot #	A(V)	B(V)	C(V)	D(R)	E(R)	F(R)	Pot #	A(V)	B(V)	C(V)	D(R)	E(R)	F(R)
05/10/2022	51	0	0	1	0	0	0	63	0	0	0	0	0	0	75	1	1	0	1	1	1	85	0	0	0	0	1	0
	52	1	0	1	1	2	1	64	0	0	0	0	0	0	76	1	2	2	1	1	1	87	0	0	0	0	0	0
	53	0	0	0	1	1	1	65	0	0	0	0	0	0	77	0	0	0	0	0	0	88	0	0	0	1	0	1
	54	0	2	0	1	1	1	67	0	1	1	1	1	1	78	0	1	0	0	1	1	89	0	1	0	1	0	0
	55	0	1	0	0	2	0	68	2	1	2	2	1	1	79	0	2	0	1	1	0	91	0	0	0	0	0	0
	57	0	0	0	1	1	0	69	1	0	0	1	1	0	80	1	1	0	1	0	0	92	2	1	0	1	1	1
	58	0	0	0	0	0	1	70	1	0	1	1	0	0	82	0	0	0	1	0	1	93	2	2	2	1	0	0
	60	0	1	0	2	2	3	71	0	0	1	1	0	1	83	0	0	0	0	0	0	94	0	0	0	0	0	1
	61	1	0	0	1	2	0	72	0	1	0	2	2	1	84	0	0	0	1	0	1	95	0	0	0	0	0	0
	62	0	0	0	0	1	2	73	0	1	1	1	2	2	86	1	1	0	2	2	2	96	0	0	0	1	1	1
05/13/2022	51	1	1	1	0	0	0	63	0	1	0	1	0	0	75	1	2	0	2	2	2	85	2	1	1	1	1	1
	52	1	1	2	1	2	2	64	0	0	0	0	1	1	76	1	2	2	2	1	1	87	1	0	1	1	1	0
	53	1	1	0	2	2	2	65	1	1	0	0	1	1	77	1	1	1	1	0	1	88	1	2	1	1	1	1
	54	1	2	0	2	1	2	67	0	2	1	1	1	1	78	1	1	1	1	1	1	89	0	1	0	1	0	1
	55	1	2	1	0	4	2	68	3	2	3	3	2	2	79	0	4	1	1	1	0	91	0	0	0	0	0	0
	57	1	2	1	1	1	1	69	1	0	1	1	1	1	80	1	1	0	2	1	0	92	2	2	1	1	1	1
	58	1	0	1	1	1	1	70	1	2	1	2	1	1	82	1	1	1	1	0	1	93	2	2	2	1	1	1
	60	1	1	2	3	3	4	71	0	0	1	2	1	1	83	1	1	1	1	1	1	94	1	0	1	1	0	1
	61	1	0	1	2	3	0	72	0	2	1	4	3	2	84	1	1	1	2	1	3	95	1	0	0	0	0	0
	62	0	0	0	1	2	3	73	1	2	2	2	3	3	86	2	1	1	1	2	3	96	0	1	0	1	1	1
05/16/2022	51	1	1	4	0	0	0	63	0	1	0	1	0	0	75	4	4	1	3	3	3	85	4	4	4	2	2	2
	52	1	4	4	1	2	2	64	0	0	2	0	2	2	76	4	5	4	3	3	2	87	3	0	4	2	2	1
	53	3	1	0	2	2	2	65	3	3	0	2	2	1	77	3	4	2	2	0	4	88	4	5	4	3	2	3
	54	3	4	0	3	2	2	67	0	2	3	2	2	2	78	3	4	4	2	3	3	89	0	3	2	3	3	2
	55	4	2	4	0	4	4	68	4	4	5	4	4	3	79	0	6	1	1	1	0	91	0	0	0	0	0	0
	57	3	3	2	3	3	2	69	3	0	4	2	1	1	80	3	4	0	4	1	0	92	4	4	3	3	2	2
	58	2	0	2	2	2	3	70	3	3	2	2	2	5	82	4	4	4	2	2	2	93	4	4	4	3	3	2
	60	3	1	5	4	4	4	71	0	0	1	2	4	1	83	4	3	4	2	2	2	94	3	0	3	3	0	2
	61	1	0	1	3	3	1	72	0	4	3	4	4	2	84	3	4	4	3	3	3	95	3	3	4	0	3	0
	62	0	0	0	2	2	4	73	3	4	4	4	4	4	86	4	3	3	2	2	6	96	0	4	0	2	2	1
05/19/22	97	5	4	4	3	3	3	74	5	5	5	5	5	5	75	4	4	1	3	3	3	85	4	4	4	3	3	3
	52	1	5	5	2	3	3	64	0	0	3	0	3	3	76	5	6	5	3	3	2	87	5	0	5	3	3	3
	53	4	3	0	3	3	3	65	4	4	0	3	3	3	77	5	4	3	2	0	4	88	5	5	5	3	2	3
	54	4	5	0	3	3	3	67	1	2	4	2	3	3	78	4	5	5	2	3	3	89	1	3	3	3	3	3
	55	5	2	5	0	4	5	68	5	5	6	4	4	4	79	0	6	1	1	1	0	98	5	4	5	3	3	1
	57	4	4	4	3	3	3	69	3	0	5	3	3	3	80	4	4	0	4	1	0	92	5	5	4	3	2	2
	58	3	1	3	3	3	3	70	3	3	3	3	3	6	82	4	4	4	3	3	3	93	5	5	5	3	3	3
	60	4	1	5	4	4	4	71	0	0	1	3	4	2	83	5	4	5	2	3	2	94	4	0	4	3	0	3
	61	1	0	1	4	4	1	72	0	5	4	4	4	2	84	4	5	5	4	3	4	95	5	4	5	0	4	0
	62	0	0	0	3	2	5	73	4	5	4	4	4	4	86	5	4	5	3	3	6	96	0	5	0	3	2	2
05/19/22	NOTES: Changed 63 for 74(all veg); Changed 51 for 97; changed 91 for 98.																											
05/22/22	97	6	6	6	4	4	4	74	6	5	6	6	6	6	75	5	5	1	4	4	4	85	5	5	5	3	3	3
	52	1	6	6	3	4	3	64	0	0	5	0	3	3	76	6	6	6	3	3	3	87	5	0	5	3	3	3
	53	4	4	0	3	4	3	65	4	4	0	3	3	3	77	5	5	3	3	0	4	88	5	5	5	4	2	4
	54	6	6	0	4	4	4	67	3	2	5	3	3	3	78	5	5	5	3	3	3	89	2	4	4	3	3	3
	55	5	2	5	1	5	5	68	6	6	6	4	4	4	79	0	6	1	1	1	0	98	5	4	5	3	3	5
	57	5	5	5	3	3	3	69	4	0	5	3	3	3	80	4	5	0	4	1	0	92	5	5	5	4	3	3
	58	4	1	5	3	3	4	70	4	4	4	5	4	6	82	5	5	6	3	3	3	93	5	5	5	4	4	3
	60	6	0	6	5	5	5	71	0	0	1	4	3	3	83	5	5	5	3	3	3	94	4	0	4	4	0	3
	61	1	0	1	4	4	1	72	0	5	4	5	4	3	84	5	5	5	4	4	4	95	5	5	5	0	4	0
	62	0	0	0	4	2	6	73	5	6	5	4	4	4	86	6	6	6	3	3	6	96	0	5	0	3	2	2
05/25/2022	97	6	6	6	4	4	4	74	6	5	6	6	6	6	75	6	6	1	4	4	4	85	5	5	5	3	3	3
	52	0	6	6	4	4	4	64	0	0	5	0	4	4	76	6	6	6	4	4	4	87	5	0	5	4	3	3
	53	6	6	0	3	4	3	65	5	6	0	4	4	4	77	5	6	3	4	0	5	88	6	6	6	3	3	4
	54	6	6	0	4	4	4	67	5	2	5	4	4	4	78	5	6	6	4	4	4	89	3	5	5	4	3	3
	55	6	6	6	2	5	6	68	6	6	6	5	5	5	79	0	6	1	1	1	0	98	5	5	5	3	3	5
	57	6	5	5	4	4	4	69	5	0	6	4	3	4	80	6	6	0	4	1	0	92	5	6	5	4	3	3
	58	4	1	6	4	4	5	70	6	5	5	5	4	6	82	6	6	6	4	4	4	93	6	6	6	4	4	3
	60	6	0	6	5	5	5	71	0	0	1	4	3	3	83	6	6	6	4	4	4	94	4	0	6	5	0	3
	61	1	0	1	4	4	1	72	0	6	5	5	5	4	84	5	5	6	5	4	4	95	6	6	6	0	6	0
	62	0	0	0	5	3	6	73	6	6	6	5	5	5	86	6	6	6	3	4	6	96	0	6	0	4	3	3

05/28/2022	97	6	6	6	4	4	4	74	6	6	6	6	6	6	75	6	6	1	4	4	4	85	6	6	6	3	4	3
	52	0	6	6	4	4	4	64	0	0	6	0	4	4	76	6	6	6	4	4	4	87	6	0	6	4	3	3
	53	6	6	0	4	4	4	65	6	6	0	4	4	4	77	6	6	4	4	0	5	88	6	6	6	4	3	4
	54	6	6	0	4	4	4	67	6	2	6	4	4	4	78	5	6	6	4	4	4	89	4	5	5	4	3	3
	55	6	6	6	2	5	6	68	6	6	6	5	5	5	79	0	6	1	1	1	0	98	6	6	6	4	4	5
	57	6	5	5	4	4	4	69	6	0	6	4	3	4	80	6	6	0	5	1	0	92	6	6	6	4	3	3
	58	4	1	6	4	4	5	70	6	5	5	5	4	6	82	6	6	6	4	4	4	93	6	6	6	5	5	5
	60	6	0	6	5	5	5	71	0	0	1	5	3	3	83	6	6	6	4	4	4	94	5	0	6	5	0	3
	61	1	0	1	5	5	1	72	0	6	6	5	5	4	84	6	6	6	5	5	5	95	6	6	6	0	6	0
	62	0	0	0	5	3	6	73	6	6	6	5	5	5	86	6	6	6	3	4	6	96	0	6	0	4	3	3
05/31/2022	97	6	6	6	5	5	5	74	6	6	6	6	6	6	75	6	6	1	4	4	5	85	6	6	6	4	5	4
	52	0	6	6	5	5	4	64	0	0	6	0	5	5	76	6	6	6	4	4	4	87	6	0	6	4	3	3
	53	6	6	0	5	5	5	65	6	6	0	4	4	4	77	6	6	4	4	0	5	88	6	6	6	4	3	4
	54	6	6	0	4	4	4	67	6	2	6	4	4	4	78	5	6	6	4	5	5	89	4	5	5	5	4	4
	55	6	6	6	3	5	6	68	6	6	6	5	5	5	79	0	6	1	1	1	0	98	6	6	6	4	4	5
	57	6	6	6	5	5	5	69	6	0	6	5	4	4	80	6	6	0	5	1	0	92	6	6	6	5	3	3
	58	6	1	6	5	5	5	70	6	5	5	5	4	6	82	6	6	6	5	5	5	93	6	6	6	5	5	5
	60	6	0	6	6	5	5	71	0	0	1	5	3	5	83	6	6	6	4	4	4	94	6	0	6	6	0	5
	61	1	0	1	5	5	1	72	0	6	6	5	5	4	84	6	6	6	5	5	5	95	6	6	6	0	6	0
	62	0	0	0	5	3	6	73	6	6	6	5	5	5	86	6	6	6	3	4	6	96	0	6	0	5	5	4
06/03/2022	97	6	6	6	5	5	5	74	6	6	6	6	6	6	75	6	6	1	5	5	5	85	6	6	6	5	5	5
	52	0	6	6	5	5	4	64	0	0	6	0	5	5	76	6	6	6	5	5	5	87	6	0	6	5	3	4
	53	6	6	0	5	5	5	65	6	6	0	5	5	5	77	6	6	5	5	0	5	88	6	6	6	5	4	5
	54	6	6	0	4	6	4	67	6	2	6	5	5	5	78	6	6	6	5	5	5	89	5	5	5	5	5	5
	55	6	6	6	5	5	6	68	6	6	6	5	5	5	79	0	6	1	1	1	0	98	6	6	6	5	5	5
	57	6	6	6	5	5	5	69	6	0	6	5	5	5	80	6	6	0	5	1	0	92	6	6	6	5	4	4
	58	6	1	6	5	6	6	70	6	6	6	5	4	6	82	6	6	6	5	5	5	93	6	6	6	5	5	5
	60	6	0	6	6	6	5	71	0	0	1	5	5	5	83	6	6	6	5	5	5	94	6	0	6	6	0	5
	61	1	0	1	5	5	1	72	0	6	6	6	5	5	84	6	6	6	5	5	5	95	6	6	6	0	6	0
	62	0	0	0	5	4	6	73	6	6	6	5	6	6	86	6	6	6	4	5	6	96	0	6	0	5	5	4
06/06/2022	97	6	6	6	5	6	5	74	6	6	6	6	6	6	75	6	6	1	5	5	5	85	6	6	6	5	5	5
	52	0	6	6	5	5	5	64	0	0	6	0	5	5	76	6	6	6	5	5	5	87	6	0	6	5	3	4
	53	6	6	0	5	6	5	65	6	6	0	5	5	5	77	6	6	6	5	0	5	88	6	6	6	5	4	5
	54	6	6	0	6	6	5	67	6	2	6	5	5	5	78	6	6	6	5	5	5	89	6	6	6	5	5	5
	55	6	6	6	5	6	6	68	6	6	6	6	6	5	79	0	6	1	1	1	0	98	6	6	6	5	5	5
	57	6	6	6	5	6	5	69	6	0	6	5	5	5	80	6	6	0	6	1	0	92	6	6	6	5	4	4
	58	6	1	6	5	6	6	70	6	6	6	5	5	6	82	6	6	6	5	5	5	93	6	6	6	6	5	5
	60	6	0	6	6	6	6	71	0	0	1	6	5	5	83	6	6	6	5	5	5	94	6	0	6	6	0	5
	61	1	0	1	6	6	1	72	0	6	6	6	6	5	84	6	6	6	6	6	6	95	6	6	6	0	6	0
	62	0	0	0	6	5	6	73	6	6	6	6	6	6	86	6	6	6	5	5	6	96	0	6	0	5	5	4
06/09/2022	97	6	6	6	6	6	6	74	6	6	6	6	6	6	75	6	6	1	6	6	6	85	6	6	6	5	5	5
	52	0	6	6	5	6	5	64	0	0	6	0	6	5	76	6	6	6	6	5	5	87	6	0	6	5	4	4
	53	6	6	0	5	6	5	65	6	6	0	6	5	5	77	6	6	6	6	0	5	88	6	6	6	5	5	5
	54	6	6	0	6	6	5	67	6	2	6	5	5	5	78	6	6	6	5	5	6	89	6	6	6	5	5	5
	55	6	6	6	5	7	6	68	6	6	6	6	6	6	79	0	6	1	1	1	0	98	6	6	6	5	5	5
	57	6	6	6	6	6	6	69	6	0	6	6	6	6	80	6	6	0	6	1	0	92	6	6	6	5	4	5
	58	6	1	6	6	6	6	70	6	6	6	5	5	6	82	6	6	6	6	5	5	93	6	6	6	6	5	5
	60	6	0	6	6	6	6	71	0	0	1	6	6	5	83	6	6	6	5	6	5	94	6	0	6	6	0	5
	61	1	0	1	6	6	1	72	0	6	6	6	6	5	84	6	6	6	6	6	6	95	6	6	6	0	6	0
	62	0	0	0	6	5	6	73	6	6	6	6	6	6	86	6	6	6	6	6	6	96	0	6	0	5	5	5
06/12/2022	97	6	6	6	6	6	6	74	6	6	6	6	6	6	75	6	6	1	6	6	6	85	6	6	6	6	6	6
	52	0	6	6	6	6	5	64	0	0	6	0	6	6	76	6	6	6	6	6	6	87	6	0	6	6	6	5
	53	6	6	0	6	7	5	65	6	6	0	6	6	6	77	6	6	6	6	0	6	88	6	6	6	6	6	6
	54	6	6	0	6	6	6	67	6	2	6	6	6	5	78	6	6	6	6	6	6	89	6	6	6	6	6	6
	55	6	6	6	5	7	6	68	6	6	6	6	6	6	79	0	6	1	1	1	0	98	6	6	6	6	6	6
	57	6	6	6	6	6	6	69	6	0	6	6	6	6	80	6	6	0	6	1	0	92	6	6	6	6	5	6
	58	6	1	6	6	6	6	70	6	6	6	5	6	6	82	6	6	6	6	6	6	93	6	6	6	6	6	6
	60	6	0	6	6	6	6	71	0	0	1	6	6	5	83	6	6	6	6	6	5	94	6	0	6	6	0	6
	61	1	0	1	6	6	1	72	0	6	6	7	6	6	84	6	6	6	6	6	6	95	6	6	6	0	6	0
	62	0	0	0	7	6	6	73	6	6	6	7	7	7	86	6	6	6	6	6	6	96	0	6	0	6	6	6
06/15/2022	97	6	6	6	6	6	6	74	6	6	6	6	6	6	75	6	6	1	6	6	6	85	6	6	6	6	6	6
	52	0	6	6	6	6	5	64	0	0	6	0	6	6	76	6	6	6	6	6	6	87	6	0	6	6	6	6
	53	6	6	0	6	7	6	65	6	6	0	6	6	6	77	6	6	6	6	0	6	88	6	6	6	6	6	6
	54	6	6	0	7	7	7	67	6	2	6	6	6	6	78	6	6	6	6	6	6	89	6	6	6	6	6	6
	55	6	6	6	6	7	6	68	6	6	6	6	7	6	79	0	6	1	1	1	0	98	6	6	6	6	6	6
	57	6	6	6	6	6	6	69	6	0	6	6	6	6	80	6	6	0	6	1	0	92	6	6	6	6	6	6
	58	6	1	6	6	6	6	70	6	6	6	5	6	6	82	6	6	6	6	6	6	93	6	6	6	6	6	6
	60	6	0	6	6	7	7	71	0	0	1	6	6	5	83	6	6	6	6	6	6	94	6	0	6	6	0	6
	61	1	0	1	6	6	1	72	0	6	6	7	6	6	84	6	6	6	6	6	6	95	6	6	6	0	6	0
	62	0	0	0	7	7	6	73	6	6	6	7	7	7	86	6	6	6	6	6	6	96	0	6	0	6	6	6

06/18/2022	97	6	6	6	6	6	6	74	6	6	6	6	6	6	75	6	6	1	6	6	6	85	6	6	6	6	6	6
	52	0	6	6	6	6	5	64	0	0	6	0	6	6	76	6	6	6	6	6	6	87	6	0	6	6	6	6
	53	6	6	0	7	7	7	65	6	6	0	6	6	6	77	6	6	6	6	0	6	88	6	6	6	6	6	6
	54	6	6	0	7	7	7	67	6	2	6	6	6	6	78	6	6	6	6	6	6	89	6	6	6	6	6	6
	55	6	6	6	6	7	6	68	6	6	6	6	7	6	79	0	6	1	1	1	0	98	6	6	6	6	6	6
	57	6	6	6	6	6	6	69	6	0	6	6	6	6	80	6	6	0	7	1	0	92	6	6	6	6	7	6
	58	6	1	6	6	7	7	70	6	6	6	6	6	6	82	6	6	6	6	6	6	93	6	6	6	6	6	7
	60	6	0	6	7	7	8	71	0	0	1	6	6	7	83	6	6	6	6	6	6	94	6	0	6	6	0	6
	61	1	0	1	7	6	1	72	0	6	6	7	6	6	84	6	6	6	6	6	6	95	6	6	6	0	6	0
	62	0	0	0	8	7	6	73	6	6	6	7	7	7	86	6	6	6	6	6	6	96	0	6	0	6	6	6
06/21/2022	97	6	6	6	7	7	7	74	6	6	6	6	6	6	75	6	6	1	7	7	7	85	6	6	6	6	6	6
	52	0	6	6	7	7	6	64	0	0	6	0	7	7	76	6	6	6	7	7	7	87	6	0	6	6	6	6
	53	6	6	0	7	7	7	65	6	6	0	7	7	7	77	6	6	6	7	0	7	88	6	6	6	6	6	6
	54	6	6	0	7	7	7	67	6	2	6	6	6	7	78	6	6	6	7	7	7	89	6	6	6	6	6	6
	55	6	6	6	6	8	7	68	6	6	6	6	7	6	79	0	6	1	1	1	0	98	6	6	6	6	7	6
	57	6	6	6	8	8	7	69	6	0	6	7	7	7	80	6	6	0	8	1	0	92	6	6	6	7	7	7
	58	6	1	6	7	8	8	70	6	6	6	6	6	6	82	6	6	6	7	6	7	93	6	6	6	7	7	8
	60	6	0	6	8	8	8	71	0	0	1	8	7	8	83	6	6	6	7	7	6	94	6	0	6	6	0	6
	61	1	0	1	7	6	1	72	0	6	6	8	7	7	84	6	6	6	7	7	7	95	6	6	6	0	6	0
	62	0	0	0	8	7	6	73	6	6	6	8	8	8	86	6	6	6	6	6	6	96	0	6	0	7	6	6
06/24/2022	97	6	6	6	8	7	8	74	6	6	6	6	6	6	75	6	6	1	7	7	7	85	6	6	6	6	6	6
	52	0	6	6	8	7	8	64	0	0	6	0	8	8	76	6	6	6	7	7	7	87	6	0	6	7	7	7
	53	6	6	0	8	8	8	65	6	6	0	8	8	8	77	6	6	6	7	0	8	88	6	6	6	6	6	6
	54	6	6	0	7	7	7	67	6	2	6	7	7	7	78	6	6	6	7	7	7	89	6	6	6	6	6	6
	55	6	6	6	7	8	7	68	6	6	6	8	7	7	79	0	6	1	1	1	0	98	6	6	6	7	7	7
	57	6	6	6	8	8	7	69	6	0	6	8	8	8	80	6	6	0	8	1	0	92	6	6	6	8	8	7
	58	6	1	6	7	8	8	70	6	6	6	7	7	7	82	6	6	6	8	8	8	93	6	6	6	8	8	8
	60	6	0	6	8	8	8	71	0	0	1	7	7	8	83	6	6	6	7	7	7	94	6	0	6	6	0	6
	61	1	0	1	7	6	1	72	0	6	6	8	8	8	84	6	6	6	8	8	8	95	6	6	6	0	6	0
	62	0	0	0	8	7	6	73	6	6	6	8	8	8	86	6	6	6	6	6	6	96	0	6	0	7	7	6
06/27/2022	97	6	6	6	9	9	9	74	6	6	6	6	6	6	75	6	6	1	8	8	8	85	6	6	6	8	8	8
	52	0	6	6	9	9	9	64	0	0	6	0	9	9	76	6	6	6	9	9	8	87	6	0	6	8	8	8
	53	6	6	0	9	9	9	65	6	6	0	9	9	9	77	6	6	6	8	0	9	88	6	6	6	8	7	8
	54	6	6	0	9	8	9	67	6	2	6	7	7	7	78	6	6	6	8	9	8	89	6	6	6	6	6	6
	55	6	6	6	9	9	8	68	6	6	6	9	9	9	79	0	6	1	1	1	0	98	6	6	6	8	8	8
	57	6	6	6	9	9	9	69	6	0	6	9	9	9	80	6	6	0	9	1	0	92	6	6	6	9	9	9
	58	6	1	6	9	9	9	70	6	6	6	8	8	8	82	6	6	6	9	9	9	93	6	6	6	9	9	9
	60	6	0	6	9	9	9	71	0	0	1	9	8	9	83	6	6	6	9	9	9	94	6	0	6	6	0	6
	61	1	0	1	8	6	1	72	0	6	6	9	9	9	84	6	6	6	9	9	9	95	6	6	6	0	6	0
	62	0	0	0	9	8	6	73	6	6	6	9	9	9	86	6	6	6	6	6	6	96	0	6	0	8	8	6
06/30/2022	97	6	6	6	9	9	9	74	6	6	6	6	6	6	75	6	6	1	9	9	9	85	6	6	6	9	9	9
	52	0	6	6	9	9	9	64	0	0	6	0	9	9	76	6	6	6	9	9	9	87	6	0	6	9	9	9
	53	6	6	0	9	9	9	65	6	6	0	9	9	9	77	6	6	6	9	0	9	88	6	6	6	9	8	9
	54	6	6	0	9	9	9	67	6	2	6	8	8	8	78	6	6	6	9	9	9	89	6	6	6	6	6	6
	55	6	6	6	9	9	9	68	6	6	6	9	9	9	79	0	6	1	1	1	0	98	6	6	6	8	8	8
	57	6	6	6	9	9	9	69	6	0	6	9	9	9	80	6	6	0	9	1	0	92	6	6	6	9	9	9
	58	6	1	6	9	9	9	70	6	6	6	9	9	9	82	6	6	6	9	9	9	93	6	6	6	9	9	9
	60	6	0	6	9	9	9	71	0	0	1	9	9	9	83	6	6	6	9	9	9	94	6	0	6	6	0	6
	61	1	0	1	8	6	1	72	0	6	6	9	9	9	84	6	6	6	9	9	9	95	6	6	6	0	6	0
	62	0	0	0	9	9	6	73	6	6	6	9	9	9	86	6	6	6	6	6	6	96	0	6	0	8	8	6
07/03/2022	97	6	6	6	9	9	9	74	6	6	6	6	6	6	75	6	6	1	9	9	9	85	6	6	6	9	9	9
	52	0	6	6	9	9	9	64	0	0	6	0	9	9	76	6	6	6	9	9	9	87	6	0	6	9	9	9
	53	6	6	0	9	9	9	65	6	6	0	9	9	9	77	6	6	6	9	0	9	88	6	6	6	9	8	9
	54	6	6	0	9	9	9	67	6	2	6	8	8	8	78	6	6	6	9	9	9	89	6	6	6	6	6	6
	55	6	6	6	9	9	9	68	6	6	6	9	9	9	79	0	6	1	1	1	0	98	6	6	6	8	8	8
	57	6	6	6	9	9	9	69	6	0	6	9	9	9	80	6	6	0	9	1	0	92	6	6	6	9	9	9
	58	6	1	6	9	9	9	70	6	6	6	9	9	9	82	6	6	6	9	9	9	93	6	6	6	9	9	9
	60	6	0	6	9	9	9	71	0	0	1	9	9	9	83	6	6	6	9	9	9	94	6	0	6	6	0	6
	61	1	0	1	9	6	1	72	0	6	6	9	9	9	84	6	6	6	9	9	9	95	6	6	6	0	6	0
	62	0	0	0	9	9	6	73	6	6	6	9	9	9	86	6	6	6	6	6	6	96	0	6	0	8	8	6
07/06/2022	97	6	6	6	9	9	9	74	6	6	6	6	6	6	75	6	6	1	9	9	9	85	6	6	6	9	9	9
	52	0	6	6	9	9	9	64	0	0	6	0	9	9	76	6	6	6	9	9	9	87	6	0	6	9	9	9
	53	6	6	0	9	9	9	65	6	6	0	9	9	9	77	6	6	6	9	0	9	88	6	6	6	9	8	9
	54	6	6	0	9	9	9	67	6	2	6	9	8	8	78	6	6	6	9	9	9	89	6	6	6	6	6	6
	55	6	6	6	9	9	9	68	6	6	6	9	9	9	79	0	6	1	1	1	0	98	6	6	6	8	8	8
	57	6	6	6	9	9	9	69	6	0	6	9	9	9	80	6	6	0	9	1	0	92	6	6	6	9	9	9
	58	6	1	6	9	9	9	70	6	6	6	9	9	9	82	6	6	6	9	9	9	93	6	6	6	9	9	9
	60	6	0	6	9	9	9	71	0	0	1	9	9	9	83	6	6	6	9	9	9	94	6	0	6	6	0	6
	61	1	0	1	9	6	1	72	0	6	6	9	9	9	84	6	6	6	9	9	9	95	6	6	6	0	6	0
	62	0	0	0	9	9	6	73	6	6	6	9	9	9	86	6	6	6	6	6	6	96	0	6	0	8	8	6

07/09/2022	97	6	6	6	9	9	9	74	6	6	6	6	6	6	75	6	6	1	9	9	9	85	6	6	6	9	9	9
	52	0	6	6	9	9	9	64	0	0	6	0	9	9	76	6	6	6	9	9	9	87	6	0	6	9	9	9
	53	6	6	0	9	9	9	65	6	6	0	9	9	9	77	6	6	6	9	0	9	88	6	6	6	9	9	9
	54	6	6	0	9	9	9	67	6	2	6	9	9	9	78	6	6	6	9	9	9	89	6	6	6	6	6	6
	55	6	6	6	9	9	9	68	6	6	6	9	9	9	79	0	6	1	1	1	0	98	6	6	6	8	8	8
	57	6	6	6	9	9	9	69	6	0	6	9	9	9	80	6	6	0	9	1	0	92	6	6	6	9	9	9
	58	6	1	6	9	9	9	70	6	6	6	9	9	9	82	6	6	6	9	9	9	93	6	6	6	9	9	9
	60	6	0	6	9	9	9	71	0	0	1	9	9	9	83	6	6	6	9	9	9	94	6	0	6	6	0	6
	61	1	0	1	9	6	1	72	0	6	6	9	9	9	84	6	6	6	9	9	9	95	6	6	6	0	6	0
	62	0	0	0	9	9	6	73	6	6	6	9	9	9	86	6	6	6	6	6	6	96	0	6	0	8	8	6
07/12/2022	97	6	6	6	9	9	9	74	6	6	6	6	6	6	75	6	6	1	9	9	9	85	6	6	6	9	9	9
	52	0	6	6	9	9	9	64	0	0	6	0	9	9	76	6	6	6	9	9	9	87	6	0	6	9	9	9
	53	6	6	0	9	9	9	65	6	6	0	9	9	9	77	6	6	6	9	0	9	88	6	6	6	9	9	9
	54	6	6	0	9	9	9	67	6	2	6	9	9	9	78	6	6	6	9	9	9	89	6	6	6	6	6	6
	55	6	6	6	9	9	9	68	6	6	6	9	9	9	79	0	6	1	1	1	0	98	6	6	6	8	8	8
	57	6	6	6	9	9	9	69	6	0	6	9	9	9	80	6	6	0	9	1	0	92	6	6	6	9	9	9
	58	6	1	6	9	9	9	70	6	6	6	9	9	9	82	6	6	6	9	9	9	93	6	6	6	9	9	9
	60	6	0	6	9	9	9	71	0	0	1	9	9	9	83	6	6	6	9	9	9	94	6	0	6	6	0	6
	61	1	0	1	9	6	1	72	0	6	6	9	9	9	84	6	6	6	9	9	9	95	6	6	6	0	6	0
	62	0	0	0	9	9	6	73	6	6	6	9	9	9	86	6	6	6	6	6	6	96	0	6	0	8	8	6
07/15/2022	97	6	6	6	10	10	9	74	6	6	6	6	6	6	75	6	6	1	9	9	9	85	6	6	6	9	9	9
	52	0	6	6	10	10	10	64	0	0	6	0	9	9	76	6	6	6	9	9	9	87	6	0	6	9	9	9
	53	6	6	0	10	10	10	65	6	6	0	9	9	9	77	6	6	6	9	0	9	88	6	6	6	9	9	9
	54	6	6	0	10	10	10	67	6	2	6	9	9	9	78	6	6	6	9	9	9	89	6	6	6	6	6	6
	55	6	6	6	9	9	9	68	6	6	6	9	9	9	79	0	6	1	1	1	0	98	6	6	6	9	8	8
	57	6	6	6	9	9	9	69	6	0	6	9	9	9	80	6	6	0	9	1	0	92	6	6	6	9	9	9
	58	6	1	6	10	9	9	70	6	6	6	9	9	9	82	6	6	6	9	9	9	93	6	6	6	9	9	9
	60	6	0	6	9	9	9	71	0	0	1	9	9	9	83	6	6	6	9	9	9	94	6	0	6	6	0	6
	61	1	0	1	9	6	1	72	0	6	6	9	9	9	84	6	6	6	9	9	9	95	6	6	6	0	6	0
	62	0	0	0	9	9	6	73	6	6	6	9	9	9	86	6	6	6	6	6	6	96	0	6	0	8	9	6
07/18/2022	97	6	6	6	10	10	9	74	6	6	6	6	6	6	75	6	6	1	9	9	9	85	6	6	6	9	9	9
	52	0	6	6	10	10	10	64	0	0	6	0	9	9	76	6	6	6	9	9	9	87	6	0	6	9	9	9
	53	6	6	0	10	10	10	65	6	6	0	9	9	9	77	6	6	6	9	0	9	88	6	6	6	9	9	9
	54	6	6	0	10	10	10	67	6	2	6	9	9	9	78	6	6	6	9	9	9	89	6	6	6	6	6	6
	55	6	6	6	9	9	9	68	6	6	6	9	9	9	79	0	6	1	1	1	0	98	6	6	6	9	9	9
	57	6	6	6	10	10	10	69	6	0	6	9	9	9	80	6	6	0	9	1	0	92	6	6	6	9	9	9
	58	6	1	6	10	9	9	70	6	6	6	9	9	9	82	6	6	6	9	9	9	93	6	6	6	9	9	9
	60	6	0	6	10	9	9	71	0	0	1	9	9	9	83	6	6	6	9	9	9	94	6	0	6	6	0	6
	61	1	0	1	9	6	1	72	0	6	6	9	10	10	84	6	6	6	9	9	9	95	6	6	6	0	6	0
	62	0	0	0	10	9	6	73	6	6	6	9	9	9	86	6	6	6	6	6	6	96	0	6	0	9	9	6
07/21/2022	97	6	6	6	10	10	10	74	6	6	6	6	6	6	75	6	6	1	9	9	9	85	6	6	6	9	9	9
	52	0	6	6	10	10	10	64	0	0	6	0	9	9	76	6	6	6	9	9	9	87	6	0	6	9	9	9
	53	6	6	0	10	10	10	65	6	6	0	9	9	9	77	6	6	6	9	0	9	88	6	6	6	9	9	9
	54	6	6	0	10	10	10	67	6	2	6	9	9	9	78	6	6	6	9	9	9	89	6	6	6	6	6	6
	55	6	6	6	9	10	9	68	6	6	6	9	9	9	79	0	6	1	1	1	0	98	6	6	6	9	9	9
	57	6	6	6	10	10	10	69	6	0	6	9	9	9	80	6	6	0	9	1	0	92	6	6	6	9	9	9
	58	6	1	6	10	9	9	70	6	6	6	9	9	9	82	6	6	6	9	9	9	93	6	6	6	9	9	9
	60	6	0	6	10	9	9	71	0	0	1	10	9	9	83	6	6	6	9	9	9	94	6	0	6	6	0	6
	61	1	0	1	9	6	1	72	0	6	6	9	10	10	84	6	6	6	9	9	9	95	6	6	6	0	6	0
	62	0	0	0	10	9	6	73	6	6	6	9	9	9	86	6	6	6	6	6	6	96	0	6	0	9	9	6
07/24/2022	97	6	6	6	10	10	10	74	6	6	6	6	6	6	75	6	6	1	9	9	9	85	6	6	6	9	9	9
	52	0	6	6	10	10	10	64	0	0	6	0	9	9	76	6	6	6	9	9	9	87	6	0	6	9	9	9
	53	6	6	0	10	10	10	65	6	6	0	9	9	9	77	6	6	6	9	0	9	88	6	6	6	9	9	9
	54	6	6	0	10	10	10	67	6	2	6	9	9	9	78	6	6	6	9	9	9	89	6	6	6	6	6	6
	55	6	6	6	10	10	9	68	6	6	6	9	9	9	79	0	6	1	1	1	0	98	6	6	6	9	9	9
	57	6	6	6	11	10	10	69	6	0	6	9	9	9	80	6	6	0	10	1	0	92	6	6	6	9	9	9
	58	6	1	6	10	10	10	70	6	6	6	9	9	9	82	6	6	6	9	9	9	93	6	6	6	9	9	9
	60	6	0	6	10	9	9	71	0	0	1	10	9	9	83	6	6	6	9	9	9	94	6	0	6	6	0	6
	61	1	0	1	9	6	1	72	0	6	6	9	10	10	84	6	6	6	9	10	10	95	6	6	6	0	6	0
	62	0	0	0	10	9	6	73	6	6	6	10	10	10	86	6	6	6	6	6	6	96	0	6	0	9	9	6
07/27/2022	97	6	6	6	10	11	10	74	6	6	6	6	6	6	75	6	6	1	9	9	9	85	6	6	6	9	9	9
	52	0	6	6	10	10	10	64	0	0	6	0	9	9	76	6	6	6	9	9	9	87	6	0	6	9	9	9
	53	6	6	0	10	10	10	65	6	6	0	9	9	10	77	6	6	6	9	0	9	88	6	6	6	9	9	9
	54	6	6	0	10	10	10	67	6	2	6	9	9	9	78	6	6	6	9	9	9	89	6	6	6	6	6	6
	55	6	6	6	10	10	9	68	6	6	6	9	9	9	79	0	6	1	1	1	0	98	6	6	6	9	9	9
	57	6	6	6	11	10	10	69	6	0	6	9	9	9	80	6	6	0	11	1	0	92	6	6	6	9	9	9
	58	6	1	6	10	11	11	70	6	6	6	9	9	9	82	6	6	6	9	10	9	93	6	6	6	9	9	9
	60	6	0	6	11	9	9	71	0	0	1	10	9	9	83	6	6	6	9	9	9	94	6	0	6	6	0	6
	61	1	0	1	9	6	1	72	0	6	6	9	10	10	84	6	6	6	9	10	10	95	6	6	6	0	6	0
	62	0	0	0	10	9	6	73	6	6	6	10	10	10	86	6	6	6	6	6	6	96	0	6	0	9	9	6

07/29/2022	97	6	6	6	10	11	11	74	6	6	6	6	6	6	75	6	6	1	9	9	9	85	6	6	6	9	9	9
	52	0	6	6	10	10	10	64	0	0	6	0	9	9	76	6	6	6	9	9	9	87	6	0	6	9	9	9
	53	6	6	0	10	10	10	65	6	6	0	10	10	10	77	6	6	6	9	0	9	88	6	6	6	9	9	9
	54	6	6	0	10	10	10	67	6	2	6	9	9	9	78	6	6	6	9	9	9	89	6	6	6	6	6	6
	55	6	6	6	10	10	9	68	6	6	6	10	9	9	79	0	6	1	1	1	0	98	6	6	6	9	9	9
	57	6	6	6	11	11	11	69	6	0	6	9	9	9	80	6	6	0	11	1	0	92	6	6	6	9	9	9
	58	6	1	6	10	11	11	70	6	6	6	9	9	9	82	6	6	6	9	10	9	93	6	6	6	9	9	9
	60	6	0	6	11	10	10	71	0	0	1	10	9	9	83	6	6	6	9	9	9	94	6	0	6	6	0	6
	61	1	0	1	9	6	1	72	0	6	6	11	10	10	84	6	6	6	10	10	10	95	6	6	6	0	6	0
	62	0	0	0	10	9	6	73	6	6	6	10	10	10	86	6	6	6	6	6	6	96	0	6	0	9	9	6
08/01/2022	97	6	6	6	11	11	11	74	6	6	6	6	6	6	75	6	6	1	11	11	10	85	6	6	6	9	9	9
	52	0	6	6	11	10	11	64	0	0	6	0	9	9	76	6	6	6	9	10	9	87	6	0	6	9	9	9
	53	6	6	0	11	11	11	65	6	6	0	10	10	10	77	6	6	6	9	0	9	88	6	6	6	9	9	9
	54	6	6	0	10	10	10	67	6	2	6	9	9	9	78	6	6	6	9	9	10	89	6	6	6	6	6	6
	55	6	6	6	11	11	9	68	6	6	6	11	11	11	79	0	6	1	1	1	0	98	6	6	6	9	9	9
	57	6	6	6	11	11	11	69	6	0	6	9	10	11	80	6	6	0	11	1	0	92	6	6	6	10	9	9
	58	6	1	6	11	11	11	70	6	6	6	9	9	9	82	6	6	6	11	11	10	93	6	6	6	9	10	10
	60	6	0	6	11	11	10	71	0	0	1	11	10	10	83	6	6	6	11	10	9	94	6	0	6	6	0	6
	61	1	0	1	9	6	1	72	0	6	6	11	10	11	84	6	6	6	10	10	10	95	6	6	6	0	6	0
	62	0	0	0	11	11	6	73	6	6	6	11	10	11	86	6	6	6	6	6	6	96	0	6	0	9	9	6
08/04/2022	97	6	6	6	11	11	11	74	6	6	6	6	6	6	75	6	6	1	11	11	10	85	6	6	6	9	9	9
	52	0	6	6	11	11	11	64	0	0	6	0	10	9	76	6	6	6	10	11	9	87	6	0	6	9	9	9
	53	6	6	0	11	11	11	65	6	6	0	10	10	10	77	6	6	6	9	0	9	88	6	6	6	9	9	9
	54	6	6	0	10	10	10	67	6	2	6	9	9	9	78	6	6	6	9	10	11	89	6	6	6	6	6	6
	55	6	6	6	11	11	9	68	6	6	6	11	11	11	79	0	6	1	1	1	0	98	6	6	6	9	9	9
	57	6	6	6	11	11	11	69	6	0	6	10	10	11	80	6	6	0	11	1	0	92	6	6	6	10	9	9
	58	6	1	6	11	11	11	70	6	6	6	9	9	9	82	6	6	6	11	11	10	93	6	6	6	9	10	10
	60	6	0	6	11	11	11	71	0	0	1	11	11	10	83	6	6	6	11	10	10	94	6	0	6	6	0	6
	61	1	0	1	9	6	1	72	0	6	6	11	11	11	84	6	6	6	10	11	10	95	6	6	6	0	6	0
	62	0	0	0	11	11	6	73	6	6	6	11	10	11	86	6	6	6	6	6	6	96	0	6	0	9	9	6
08/07/2022	97	6	6	6	11	11	11	74	6	6	6	6	6	6	75	6	6	1	11	11	10	85	6	6	6	9	9	9
	52	0	6	6	11	11	11	64	0	0	6	0	10	9	76	6	6	6	10	11	9	87	6	0	6	9	9	9
	53	6	6	0	11	11	11	65	6	6	0	10	10	10	77	6	6	6	9	0	9	88	6	6	6	9	11	9
	54	6	6	0	11	10	10	67	6	2	6	9	9	9	78	6	6	6	10	10	11	89	6	6	6	6	6	6
	55	6	6	6	11	11	9	68	6	6	6	11	11	11	79	0	6	1	1	1	0	98	6	6	6	9	9	9
	57	6	6	6	11	11	11	69	6	0	6	11	10	11	80	6	6	0	11	1	0	92	6	6	6	10	9	9
	58	6	1	6	11	11	11	70	6	6	6	9	9	9	82	6	6	6	11	11	10	93	6	6	6	9	10	10
	60	6	0	6	11	11	11	71	0	0	1	11	11	10	83	6	6	6	11	10	10	94	6	0	6	6	0	6
	61	1	0	1	9	6	1	72	0	6	6	11	11	11	84	6	6	6	10	11	10	95	6	6	6	0	6	0
	62	0	0	0	11	11	6	73	6	6	6	11	11	11	86	6	6	6	6	6	6	96	0	6	0	9	9	6
08/11/2022	97	6	6	6	11	11	11	74	6	6	6	6	6	6	75	6	6	1	11	11	10	85	6	6	6	9	9	9
	52	0	6	6	11	11	11	64	0	0	6	0	10	9	76	6	6	6	10	11	9	87	6	0	6	9	9	9
	53	6	6	0	11	11	11	65	6	6	0	10	10	10	77	6	6	6	9	0	10	88	6	6	6	9	11	9
	54	6	6	0	11	10	11	67	6	2	6	9	9	9	78	6	6	6	10	10	11	89	6	6	6	6	6	6
	55	6	6	6	11	11	9	68	6	6	6	11	11	11	79	0	6	1	1	1	0	98	6	6	6	9	9	9
	57	6	6	6	11	11	11	69	6	0	6	11	11	11	80	6	6	0	11	1	0	92	6	6	6	10	9	9
	58	6	1	6	11	11	11	70	6	6	6	9	9	9	82	6	6	6	11	11	10	93	6	6	6	9	10	10
	60	6	0	6	11	11	11	71	0	0	1	11	11	10	83	6	6	6	11	10	10	94	6	0	6	6	0	6
	61	1	0	1	9	6	1	72	0	6	6	11	11	11	84	6	6	6	11	11	11	95	6	6	6	0	6	0
	62	0	0	0	11	11	6	73	6	6	6	11	11	11	86	6	6	6	6	6	6	96	0	6	0	9	9	6
08/14/2022	97	6	6	6	11	11	11	74	6	6	6	6	6	6	75	6	6	1	11	11	10	85	6	6	6	10	9	9
	52	0	6	6	11	11	11	64	0	0	6	0	11	11	76	6	6	6	10	11	10	87	6	0	6	9	9	9
	53	6	6	0	11	11	11	65	6	6	0	11	11	10	77	6	6	6	9	0	10	88	6	6	6	9	11	9
	54	6	6	0	11	10	11	67	6	2	6	9	9	9	78	6	6	6	10	11	11	89	6	6	6	6	6	6
	55	6	6	6	11	11	9	68	6	6	6	11	11	11	79	0	6	1	1	1	0	98	6	6	6	9	10	11
	57	6	6	6	11	11	11	69	6	0	6	11	11	11	80	6	6	0	11	1	0	92	6	6	6	10	10	10
	58	6	1	6	11	11	11	70	6	6	6	9	9	9	82	6	6	6	11	11	10	93	6	6	6	10	10	10
	60	6	0	6	11	11	11	71	0	0	1	11	11	11	83	6	6	6	11	10	11	94	6	0	6	6	0	6
	61	1	0	1	9	6	1	72	0	6	6	11	11	11	84	6	6	6	11	11	11	95	6	6	6	0	6	0
	62	0	0	0	11	11	6	73	6	6	6	11	11	11	86	6	6	6	6	6	6	96	0	6	0	9	9	6
08/17/2022	97	6	6	6	11	11	11	74	6	6	6	6	6	6	75	6	6	1	11	11	10	85	6	6	6	10	10	9
	52	0	6	6	11	11	11	64	0	0	6	0	11	11	76	6	6	6	11	11	10	87	6	0	6	9	10	9
	53	6	6	0	11	11	11	65	6	6	0	11	11	10	77	6	6	6	9	0	10	88	6	6	6	9	11	10
	54	6	6	0	11	10	11	67	6	2	6	9	9	9	78	6	6	6	10	11	11	89	6	6	6	6	6	6
	55	6	6	6	11	11	9	68	6	6	6	11	11	11	79	0	6	1	1	1	0	98	6	6	6	9	10	11
	57	6	6	6	11	11	11	69	6	0	6	11	11	11	80	6	6	0	11	1	0	92	6	6	6	10	10	10
	58	6	1	6	11	11	11	70	6	6	6	9	9	9	82	6	6	6	11	11	11	93	6	6	6	10	10	10
	60	6	0	6	11	11	11	71	0	0	1	11	11	11	83	6	6	6	11	11	11	94	6	0	6	6	0	6
	61	1	0	1	9	6	1	72	0	6	6	11	11	11	84	6	6	6	11	11	11	95	6	6	6	0	6	0
	62	0	0	0	11	11	6	73	6	6	6	11	11	11	86	6	6	6	6	6	6	96	0	6	0	9	9	6

08/20/2022	97	6	6	6	11	11	11	74	6	6	6	6	6	6	75	6	6	1	11	11	10	85	6	6	6	10	10	9
	52	0	6	6	11	11	11	64	0	0	6	0	11	11	76	6	6	6	11	11	10	87	6	0	6	9	10	9
	53	6	6	0	11	11	11	65	6	6	0	11	11	11	77	6	6	6	9	0	10	88	6	6	6	9	11	10
	54	6	6	0	11	11	11	67	6	2	6	9	9	9	78	6	6	6	11	11	11	89	6	6	6	6	6	6
	55	6	6	6	11	11	9	68	6	6	6	11	11	11	79	0	6	1	1	1	0	98	6	6	6	9	10	11
	57	6	6	6	11	11	11	69	6	0	6	11	11	11	80	6	6	0	11	1	0	92	6	6	6	10	10	10
	58	6	1	6	11	11	11	70	6	6	6	9	9	9	82	6	6	6	11	11	11	93	6	6	6	10	10	10
	60	6	0	6	11	11	11	71	0	0	1	11	11	11	83	6	6	6	11	11	11	94	6	0	6	6	0	6
	61	1	0	1	9	6	1	72	0	6	6	11	11	11	84	6	6	6	11	11	11	95	6	6	6	0	6	0
	62	0	0	0	11	11	6	73	6	6	6	11	11	11	86	6	6	6	6	6	6	96	0	6	0	9	9	6
08/23/2022	97	6	6	6	11	11	11	74	6	6	6	6	6	6	75	6	6	1	11	11	11	85	6	6	6	10	10	10
	52	0	6	6	11	11	11	64	0	0	6	0	11	11	76	6	6	6	11	11	10	87	6	0	6	9	10	9
	53	6	6	0	11	11	11	65	6	6	0	11	11	11	77	6	6	6	9	0	10	88	6	6	6	9	11	10
	54	6	6	0	11	11	11	67	6	2	6	9	9	9	78	6	6	6	11	11	11	89	6	6	6	6	6	6
	55	6	6	6	11	11	9	68	6	6	6	11	11	11	79	0	6	1	1	1	0	98	6	6	6	9	10	11
	57	6	6	6	11	11	11	69	6	0	6	11	11	11	80	6	6	0	11	1	0	92	6	6	6	11	10	10
	58	6	1	6	11	11	11	70	6	6	6	9	9	9	82	6	6	6	11	11	11	93	6	6	6	10	11	10
	60	6	0	6	11	11	11	71	0	0	1	11	11	11	83	6	6	6	11	11	11	94	6	0	6	6	0	6
	61	1	0	1	9	6	1	72	0	6	6	11	11	11	84	6	6	6	11	11	11	95	6	6	6	0	6	0
	62	0	0	0	11	11	6	73	6	6	6	11	11	11	86	6	6	6	6	6	6	96	0	6	0	9	9	6
08/26/2022	97	6	6	6	11	11	11	74	6	6	6	6	6	6	75	6	6	1	11	11	11	85	6	6	6	10	10	10
	52	0	6	6	11	11	11	64	0	0	6	0	11	11	76	6	6	6	11	11	10	87	6	0	6	9	10	9
	53	6	6	0	11	11	11	65	6	6	0	11	11	11	77	6	6	6	9	0	10	88	6	6	6	9	11	10
	54	6	6	0	11	11	11	67	6	2	6	9	9	9	78	6	6	6	11	11	11	89	6	6	6	6	6	6
	55	6	6	6	11	11	9	68	6	6	6	11	11	11	79	0	6	1	1	1	0	98	6	6	6	9	10	11
	57	6	6	6	11	11	11	69	6	0	6	11	11	11	80	6	6	0	11	1	0	92	6	6	6	11	10	10
	58	6	1	6	11	11	11	70	6	6	6	9	9	9	82	6	6	6	11	11	11	93	6	6	6	10	11	10
	60	6	0	6	11	11	11	71	0	0	1	11	11	11	83	6	6	6	11	11	11	94	6	0	6	6	0	6
	61	1	0	1	9	6	1	72	0	6	6	11	11	11	84	6	6	6	11	11	11	95	6	6	6	0	6	0
	62	0	0	0	11	11	6	73	6	6	6	11	11	11	86	6	6	6	6	6	6	96	0	6	0	9	9	6
08/29/2022	97	6	6	6	11	11	11	74	6	6	6	6	6	6	75	6	6	1	11	11	11	85	6	6	6	10	10	10
	52	0	6	6	11	11	11	64	0	0	6	0	11	11	76	6	6	6	11	11	10	87	6	0	6	9	10	9
	53	6	6	0	11	11	11	65	6	6	0	11	11	11	77	6	6	6	9	0	10	88	6	6	6	9	11	10
	54	6	6	0	11	11	11	67	6	2	6	9	9	9	78	6	6	6	11	11	11	89	6	6	6	6	6	6
	55	6	6	6	11	11	9	68	6	6	6	11	11	11	79	0	6	1	1	1	0	98	6	6	6	9	10	11
	57	6	6	6	11	11	11	69	6	0	6	11	11	11	80	6	6	0	11	1	0	92	6	6	6	11	10	10
	58	6	1	6	11	11	11	70	6	6	6	9	9	9	82	6	6	6	11	11	11	93	6	6	6	10	11	10
	60	6	0	6	11	11	11	71	0	0	1	11	11	11	83	6	6	6	11	11	11	94	6	0	6	6	0	6
	61	1	0	1	9	6	1	72	0	6	6	11	11	11	84	6	6	6	11	11	11	95	6	6	6	0	6	0
	62	0	0	0	11	11	6	73	6	6	6	11	11	11	86	6	6	6	6	6	6	96	0	6	0	9	9	6
09/01/2022	97	6	6	6	11	11	11	74	6	6	6	6	6	6	75	6	6	1	11	11	11	85	6	6	6	10	10	10
	52	0	6	6	11	11	11	64	0	0	6	0	11	11	76	6	6	6	11	11	10	87	6	0	6	9	10	9
	53	6	6	0	11	11	11	65	6	6	0	11	11	11	77	6	6	6	9	0	10	88	6	6	6	9	11	10
	54	6	6	0	11	11	11	67	6	2	6	9	9	9	78	6	6	6	11	11	11	89	6	6	6	6	6	6
	55	6	6	6	11	11	9	68	6	6	6	11	11	11	79	0	6	1	1	1	0	98	6	6	6	9	10	11
	57	6	6	6	11	11	11	69	6	0	6	11	11	11	80	6	6	0	11	1	0	92	6	6	6	11	10	10
	58	6	1	6	11	11	11	70	6	6	6	9	9	9	82	6	6	6	11	11	11	93	6	6	6	10	11	10
	60	6	0	6	11	11	11	71	0	0	1	11	11	11	83	6	6	6	11	11	11	94	6	0	6	6	0	6
	61	1	0	1	9	6	1	72	0	6	6	11	11	11	84	6	6	6	11	11	11	95	6	6	6	0	6	0
	62	0	0	0	11	11	6	73	6	6	6	11	11	11	86	6	6	6	6	6	6	96	0	6	0	9	9	6
09/03/2022	97	6	6	6	11	11	11	74	6	6	6	6	6	6	75	6	6	1	11	11	11	85	6	6	6	10	10	10
	52	0	6	6	11	11	11	64	0	0	6	0	11	11	76	6	6	6	11	11	10	87	6	0	6	9	10	9
	53	6	6	0	11	11	11	65	6	6	0	11	11	11	77	6	6	6	9	0	10	88	6	6	6	9	11	10
	54	6	6	0	11	11	11	67	6	2	6	9	9	9	78	6	6	6	11	11	11	89	6	6	6	6	6	6
	55	6	6	6	11	11	9	68	6	6	6	11	11	11	79	0	6	1	1	1	0	98	6	6	6	9	10	11
	57	6	6	6	11	11	11	69	6	0	6	11	11	11	80	6	6	0	11	1	0	92	6	6	6	11	10	10
	58	6	1	6	11	11	11	70	6	6	6	9	9	9	82	6	6	6	11	11	11	93	6	6	6	10	11	10
	60	6	0	6	11	11	11	71	0	0	1	11	11	11	83	6	6	6	11	11	11	94	6	0	6	6	0	6
	61	1	0	1	9	6	1	72	0	6	6	11	11	11	84	6	6	6	11	11	11	95	6	6	6	0	6	0
	62	0	0	0	11	11	6	73	6	6	6	11	11	11	86	6	6	6	6	6	6	96	0	6	0	9	9	6
09/07/2022	97	6	6	6	11	11	11	74	6	6	6	6	6	6	75	6	6	1	11	11	11	85	6	6	6	11	10	10
	52	0	6	6	11	11	11	64	0	0	6	0	11	11	76	6	6	6	11	11	10	87	6	0	6	9	10	9
	53	6	6	0	11	11	11	65	6	6	0	11	11	11	77	6	6	6	9	0	10	88	6	6	6	9	11	10
	54	6	6	0	11	11	11	67	6	2	6	9	9	9	78	6	6	6	11	11	11	89	6	6	6	6	6	6
	55	6	6	6	11	11	9	68	6	6	6	11	11	11	79	0	6	1	1	1	0	98	6	6	6	9	10	11
	57	6	6	6	11	11	11	69	6	0	6	11	11	11	80	6	6	0	11	1	0	92	6	6	6	11	10	11
	58	6	1	6	11	11	11	70	6	6	6	9	9	9	82	6	6	6	11	11	11	93	6	6	6	10	11	10
	60	6	0	6	11	11	11	71	0	0	1	11	11	11	83	6	6	6	11	11	11	94	6	0	6	6	0	6
	61	1	0	1	9	6	1	72	0	6	6	11	11	11	84	6	6	6	11	11	11	95	6	6	6	0	6	0
	62	0	0	0	11	11	6	73	6	6	6	11	11	11	86	6	6	6	6	6	6	96	0	6	0	9	9	6

09/10/2022	97	6	6	6	11	11	11	74	6	6	6	6	6	6	75	6	6	1	11	11	11	85	6	6	6	11	10	10
	52	0	6	6	11	11	11	64	0	0	6	0	11	11	76	6	6	6	11	11	10	87	6	0	6	9	10	9
	53	6	6	0	11	11	11	65	6	6	0	11	11	11	77	6	6	6	9	0	10	88	6	6	6	9	11	10
	54	6	6	0	11	11	11	67	6	2	6	9	9	9	78	6	6	6	11	11	11	89	6	6	6	6	6	6
	55	6	6	6	11	11	9	68	6	6	6	11	11	11	79	0	6	1	1	1	0	98	6	6	6	9	10	11
	57	6	6	6	11	11	11	69	6	0	6	11	11	11	80	6	6	0	11	1	0	92	6	6	6	11	10	11
	58	6	1	6	11	11	11	70	6	6	6	9	9	9	82	6	6	6	11	11	11	93	6	6	6	10	11	10
	60	6	0	6	11	11	11	71	0	0	1	11	11	11	83	6	6	6	11	11	11	94	6	0	6	6	0	6
	61	1	0	1	9	6	1	72	0	6	6	11	11	11	84	6	6	6	11	11	11	95	6	6	6	0	6	0
	62	0	0	0	11	11	6	73	6	6	6	11	11	11	86	6	6	6	6	6	6	96	0	6	0	9	9	6
09/13/2022	97	6	6	6	11	11	11	74	6	6	6	6	6	6	75	6	6	1	11	11	11	85	6	6	6	11	10	10
	52	0	6	6	11	11	11	64	0	0	6	0	11	11	76	6	6	6	11	11	10	87	6	0	6	9	10	9
	53	6	6	0	11	11	11	65	6	6	0	11	11	11	77	6	6	6	9	0	10	88	6	6	6	9	11	10
	54	6	6	0	11	11	11	67	6	2	6	9	9	9	78	6	6	6	11	11	11	89	6	6	6	6	6	6
	55	6	6	6	11	11	9	68	6	6	6	11	11	11	79	0	6	1	1	1	0	98	6	6	6	9	10	11
	57	6	6	6	11	11	11	69	6	0	6	11	11	11	80	6	6	0	11	1	0	92	6	6	6	11	10	11
	58	6	1	6	11	11	11	70	6	6	6	9	9	9	82	6	6	6	11	11	11	93	6	6	6	10	11	10
	60	6	0	6	11	11	11	71	0	0	1	11	11	11	83	6	6	6	11	11	11	94	6	0	6	6	0	6
	61	1	0	1	9	6	1	72	0	6	6	11	11	11	84	6	6	6	11	11	11	95	6	6	6	0	6	0
	62	0	0	0	11	11	6	73	6	6	6	11	11	11	86	6	6	6	6	6	6	96	0	6	0	9	9	6

Table S2: Phenological data from tagged vegetative and reproductive shoots of *V. angustifolium*.

	V. angustifolium (0% Shade)							*V. angustifolium (30% Shade)*							*V. angustifolium (50% Shade)*							*V. angustifolium (80% Shade)*						
Date	Pot #	A(V)	B(V)	C(V)	D(R)	E(R)	F(R)	Pot #	A(V)	B(V)	C(V)	D(R)	E(R)	F(R)	Pot #	A(V)	B(V)	C(V)	D(R)	E(R)	F(R)	Pot #	A(V)	B(V)	C(V)	D(R)	E(R)	F(R)
05/10/2022	1	2	2	1	4	3	3	13	1	1	1	1	1	1	22	2	2	2	3	3	3	10	0	1	1	3	2	3
	2	1	1	1	1	1	0	14	0	1	2	2	1	2	23	1	1	1	0	0	0	16	1	1	1	1	1	1
	4	2	1	0	3	4	4	15	1	1	1	1	1	2	27	2	1	1	4	3	3	36	0	0	0	0	0	0
	5	0	2	1	2	3	2	17	1	2	2	2	0	0	28	1	1	2	3	3	1	38	1	1	0	0	0	0
	7	1	2	3	4	4	4	18	1	1	0	0	0	0	29	1	2	1	1	2	0	40	2	2	2	2	2	3
	8	1	1	2	3	2	3	3	1	1	1	1	1	1	30	2	1	1	1	0	0	41	1	1	1	0	0	0
	6	1	2	2	2	2	2	19	0	2	1	1	2	2	31	1	2	1	3	1	0	46	1	1	1	0	0	0
	12	2	1	2	2	3	2	20	0	0	1	1	0	0	34	0	1	1	2	2	2	47	0	0	0	0	0	0
	26	1	0	0	0	0	0	25	0	1	2	2	0	0	37	2	1	1	1	1	1	48	1	0	1	1	1	1
	33	1	1	1	1	1	1	35	2	2	2	2	1	1	45	2	2	2	3	2	3	49	1	2	2	3	1	2
05/13/2022	1	2	3	2	4	4	3	13	1	2	1	2	2	3	22	3	3	4	4	4	4	10	1	1	2	3	3	3
	2	1	1	1	1	3	1	14	1	1	2	3	2	3	23	1	1	1	0	0	0	16	1	2	2	1	1	1
	4	3	3	1	4	4	4	15	1	2	2	3	2	3	27	2	1	1	4	4	4	36	0	0	0	0	0	0
	5	1	3	1	3	3	2	17	2	3	3	3	1	2	28	1	1	3	3	3	1	38	1	1	1	1	1	2
	7	2	3	3	4	4	4	18	2	2	0	0	0	0	29	2	3	3	3	3	0	40	2	2	2	3	2	4
	8	2	2	3	3	2	3	3	1	1	1	3	1	1	30	2	1	1	1	1	1	41	1	1	1	0	0	1
	6	2	2	2	2	2	2	19	1	2	2	2	1	4	31	2	3	1	3	1	0	46	1	1	1	2	2	1
	12	3	2	3	3	3	3	20	1	1	2	0	1	1	34	0	2	1	2	3	3	47	0	0	0	0	0	0
	26	2	2	2	0	0	0	25	0	3	2	3	2	1	37	2	1	1	2	2	2	48	1	1	2	2	1	1
	33	2	2	2	2	2	2	35	2	3	2	3	3	3	45	2	3	3	3	3	4	49	1	2	2	4	2	2
05/16/2022	1	3	3	2	5	4	4	13	1	4	3	3	3	3	22	4	4	5	4	4	4	10	4	3	3	4	4	4
	2	1	2	1	2	4	1	14	1	1	2	4	3	3	23	1	1	1	0	0	0	16	3	3	3	1	1	2
	4	3	3	1	5	5	5	15	2	3	4	3	3	3	27	4	4	3	4	4	4	36	0	0	0	0	0	0
	5	1	4	1	3	3	2	17	3	4	4	4	2	3	28	3	3	5	4	4	3	38	3	4	3	2	2	3
	7	3	4	4	4	4	4	18	4	4	0	0	0	0	29	5	4	4	4	4	0	40	5	4	4	3	4	4
	8	2	2	3	3	2	4	3	1	1	1	3	1	2	30	4	3	3	3	4	2	41	3	3	3	1	0	3
	6	2	2	2	3	3	3	19	1	4	3	4	1	4	31	4	6	3	4	3	0	46	4	4	4	4	4	4
	12	4	5	4	4	4	4	20	2	1	3	1	1	2	34	0	4	1	3	4	5	47	0	0	0	0	0	0
	26	2	2	2	0	0	0	25	1	5	3	4	4	3	37	4	3	3	3	3	3	48	3	3	4	4	3	3
	33	5	4	4	5	4	5	35	4	4	3	4	4	4	45	4	4	5	3	5	5	49	4	4	5	4	3	3
05/19/22	1	5	4	3	5	4	4	13	1	4	4	3	3	3	22	5	5	6	5	5	4	10	4	4	4	4	4	4
	2	1	3	1	2	6	1	14	1	1	3	4	3	3	23	1	1	1	1	1	1	16	4	4	4	1	3	3
	4	5	4	1	5	5	5	15	4	4	4	3	3	4	27	6	4	4	5	4	4	21	5	5	5	3	5	5
	5	3	5	1	4	3	3	17	4	5	5	3	3	3	28	4	4	5	4	4	3	38	3	4	3	3	4	4
	7	4	6	6	4	5	4	18	5	5	0	0	0	0	29	5	5	5	5	4	0	40	5	6	6	3	3	4
	8	2	2	5	3	2	5	3	3	1	1	3	1	3	30	5	4	4	3	5	2	41	4	3	4	1	0	3
	6	3	3	3	3	3	3	19	1	4	4	4	3	5	31	5	6	3	4	3	0	46	5	5	4	5	5	4
	12	4	5	5	4	4	4	20	3	1	3	2	1	2	34	0	5	1	3	5	5	44	5	4	4	4	5	3
	26	2	2	2	0	0	0	25	3	6	3	4	4	4	37	4	3	4	4	4	4	48	3	4	5	4	3	3
	33	6	5	6	6	5	6	35	4	5	5	4	4	4	45	5	5	5	3	5	5	49	5	6	6	4	3	3
05/19/22	NOTES: 36 was switched to 21(all veg) due to plant death; 47 was switched to 44 due to plant death.																											
05/22/22	1	5	4	4	5	4	4	13	1	4	4	4	4	4	22	6	6	6	5	5	4	10	5	4	5	5	5	5
	2	1	3	1	3	6	1	14	1	1	3	4	4	4	23	1	1	1	1	1	1	16	4	4	4	1	3	3
	4	5	5	1	5	5	5	15	5	5	4	4	4	4	27	6	5	5	5	4	5	21	6	6	5	6	6	6
	5	5	5	1	5	4	4	17	4	6	5	4	4	4	28	4	4	6	5	5	4	38	4	5	3	3	3	5
	7	5	4	4	4	5	4	18	5	5	0	0	0	0	29	6	6	6	6	4	0	40	6	6	6	4	4	4
	8	2	2	5	4	3	5	3	3	1	1	4	1	4	30	6	4	4	4	3	6	41	5	4	5	3	0	3
	6	3	4	3	4	4	4	19	3	5	5	4	3	5	31	6	6	3	5	4	0	46	5	5	5	6	5	4
	12	6	6	6	4	5	5	20	3	1	4	3	1	3	34	0	6	1	4	6	6	44	5	5	5	5	5	3
	26	2	2	2	0	0	0	25	3	6	4	5	5	4	37	6	4	5	5	5	5	48	5	4	6	4	4	3
	33	6	6	6	6	6	6	35	5	6	5	5	5	4	45	6	6	6	4	5	6	49	5	6	6	5	4	3
05/25/2022	1	6	6	6	5	5	5	13	1	6	6	4	4	4	22	6	6	6	5	5	5	10	5	5	5	5	5	5
	2	1	4	1	3	6	1	14	1	1	3	5	4	4	23	1	1	1	1	1	1	16	5	5	5	1	3	4
	4	5	6	1	6	6	6	15	5	5	4	4	4	4	27	6	5	5	5	5	5	21	6	6	6	5	6	6
	5	5	6	1	5	5	4	17	5	6	6	5	4	4	28	6	5	6	5	5	6	38	5	5	5	4	4	4
	7	6	5	6	5	5	5	18	6	6	0	0	0	0	29	6	6	6	6	5	0	40	6	6	6	5	5	5
	8	2	2	6	4	3	5	3	3	1	1	4	1	4	30	6	6	5	5	6	3	41	5	5	5	5	0	5
	6	4	5	5	4	4	5	19	3	6	6	5	4	5	31	6	6	3	5	4	0	46	6	6	6	6	6	6
	12	6	6	6	5	5	5	20	3	1	5	4	1	4	34	0	6	1	4	6	6	44	5	6	5	5	5	4
	26	2	2	2	0	0	0	25	3	6	5	5	5	5	37	6	5	6	5	5	5	48	5	4	6	4	4	4
	33	6	6	6	6	6	6	35	6	6	6	5	5	5	45	6	6	6	4	6	5	49	6	6	6	5	5	4

05/28/2022	1	6	6	6	5	5	5	13	1	6	6	5	4	4	22	6	6	6	5	5	5	10	5	5	5	5	5	5
	2	1	5	1	4	6	1	14	1	1	3	5	5	5	23	1	1	1	1	1	1	16	5	5	5	1	3	4
	4	6	6	1	6	6	6	15	6	6	5	5	5	5	27	6	6	6	5	5	5	21	6	6	6	6	6	6
	5	5	6	1	5	5	4	17	6	6	6	5	5	4	28	6	6	6	5	5	6	38	6	6	5	4	4	4
	7	6	6	6	5	5	5	18	6	6	0	0	0	0	29	6	6	6	6	5	0	40	6	6	6	5	5	5
	8	2	2	6	5	3	5	3	3	1	1	5	1	4	30	6	6	6	5	6	3	41	6	6	6	6	0	6
	6	4	5	5	5	5	5	19	3	6	6	5	4	5	31	6	6	3	5	5	0	46	6	6	6	6	6	6
	12	6	6	6	5	5	5	20	3	1	6	4	1	4	34	0	6	1	4	6	6	44	6	6	6	5	5	4
	26	2	2	2	0	0	0	25	3	6	6	5	5	5	37	6	5	6	5	5	5	48	6	6	6	4	4	4
	33	6	6	6	6	6	6	35	6	6	6	5	5	5	45	6	6	6	4	6	5	49	6	6	6	5	5	5
05/31/2022	1	6	6	6	6	5	5	13	1	6	6	5	5	5	22	6	6	6	5	5	5	10	6	6	6	6	5	5
	2	1	6	3	6	6	1	14	1	1	3	5	5	5	23	1	1	1	1	1	1	16	6	6	6	1	6	6
	4	6	6	1	6	6	6	15	6	6	6	5	5	5	27	6	6	6	5	5	5	21	6	6	6	6	6	6
	5	6	6	1	5	6	5	17	6	6	6	5	5	5	28	6	6	6	5	5	6	38	6	6	5	4	4	4
	7	6	6	6	5	6	5	18	6	6	0	0	0	0	29	6	6	6	6	5	0	40	6	6	6	5	5	5
	8	2	2	6	5	3	5	3	3	1	1	5	1	4	30	6	6	6	5	6	5	41	6	6	6	6	0	6
	6	6	6	6	5	5	5	19	3	6	6	5	5	6	31	6	6	3	6	5	0	46	6	6	6	6	6	6
	12	6	6	6	5	5	5	20	5	1	6	5	1	4	34	0	6	1	4	6	6	44	6	6	6	5	5	5
	26	2	2	2	0	0	0	25	3	6	6	5	5	5	37	6	5	6	5	5	5	48	6	6	6	5	5	5
	33	6	6	6	6	6	6	35	6	6	6	5	5	5	45	6	6	6	4	6	6	49	6	6	6	5	5	5
06/03/2022	1	6	6	6	6	6	6	13	1	6	6	5	5	5	22	6	6	6	6	6	5	10	6	6	6	6	6	6
	2	3	6	3	6	6	1	14	1	1	4	6	5	5	23	1	1	1	1	1	1	16	6	6	6	1	6	6
	4	6	6	1	6	6	6	15	6	6	6	6	5	6	27	6	6	6	6	6	6	21	6	6	6	6	6	6
	5	6	6	1	6	6	5	17	6	6	6	6	6	6	28	6	6	6	5	6	6	38	6	6	6	4	4	4
	7	6	6	6	6	6	6	18	6	6	0	0	0	0	29	6	6	6	6	5	0	40	6	6	6	6	6	6
	8	2	2	6	5	3	6	3	4	1	1	5	1	5	30	6	6	6	5	6	5	41	6	6	6	6	0	6
	6	6	6	6	5	6	6	19	4	6	6	5	5	6	31	6	6	3	6	6	0	46	6	6	6	6	6	6
	12	6	6	6	6	6	6	20	6	1	6	5	1	4	34	0	6	1	4	6	6	44	6	6	6	5	5	5
	26	2	2	2	0	0	0	25	3	6	6	6	6	5	37	6	6	6	6	6	6	48	6	6	6	5	5	5
	33	6	6	6	6	6	6	35	6	6	6	6	6	6	45	6	6	6	4	6	6	49	6	6	6	6	5	5
06/06/2022	1	6	6	6	6	6	6	13	1	6	6	5	6	5	22	6	6	6	6	6	6	10	6	6	6	6	6	6
	2	5	6	5	6	6	1	14	1	1	4	6	6	6	23	1	1	1	1	1	1	16	6	6	6	1	6	6
	4	6	6	1	6	6	6	15	6	6	6	6	5	6	27	6	6	6	6	6	6	21	6	6	6	6	6	6
	5	6	6	1	6	6	6	17	6	6	6	6	6	6	28	6	6	6	5	6	6	38	6	6	6	5	5	5
	7	6	6	6	6	6	6	18	6	6	0	0	0	0	29	6	6	6	6	6	0	40	6	6	6	6	6	6
	8	2	2	6	6	4	6	3	4	1	1	5	1	5	30	6	6	6	6	6	5	41	6	6	6	6	0	6
	6	6	6	6	6	6	6	19	4	6	6	6	6	6	31	6	6	3	6	6	0	46	6	6	6	6	6	6
	12	6	6	6	6	6	6	20	6	1	6	6	1	4	34	0	6	1	5	6	6	44	6	6	6	6	5	5
	26	2	2	2	0	0	0	25	3	6	6	6	6	6	37	6	6	6	6	6	6	48	6	6	6	6	6	6
	33	6	6	6	6	6	6	35	6	6	6	6	6	6	45	6	6	6	4	6	6	49	6	6	6	6	5	5
06/09/2022	1	6	6	6	7	7	7	13	1	6	6	6	6	6	22	6	6	6	6	6	6	10	6	6	6	6	6	6
	2	6	6	6	6	6	1	14	1	1	5	6	6	6	23	1	1	1	1	1	1	16	6	6	6	1	6	6
	4	6	6	1	6	6	6	15	6	6	6	6	5	6	27	6	6	6	6	6	6	21	6	6	6	6	6	6
	5	6	6	1	6	6	6	17	6	6	6	6	6	6	28	6	6	6	6	6	6	38	6	6	6	6	5	5
	7	6	6	6	6	6	6	18	6	6	0	0	0	0	29	6	6	6	6	6	0	40	6	6	6	6	6	6
	8	2	3	6	6	5	6	3	4	1	1	6	1	5	30	6	6	6	6	6	6	41	6	6	6	6	0	6
	6	6	6	6	6	6	6	19	4	6	6	6	6	6	31	6	6	4	6	6	0	46	6	6	6	6	6	6
	12	6	6	6	7	6	6	20	6	1	6	6	1	4	34	0	6	1	6	6	6	44	6	6	6	6	6	5
	26	2	2	2	0	0	0	25	3	6	6	6	6	6	37	6	6	6	6	6	6	48	6	6	6	6	6	6
	33	6	6	6	6	6	6	35	6	6	6	6	6	6	45	6	6	6	6	6	6	49	6	6	6	6	5	5
06/12/2022	1	6	6	6	7	7	7	13	1	6	6	6	6	6	22	6	6	6	7	6	6	10	6	6	6	6	6	6
	2	6	6	6	6	6	1	14	1	1	6	7	7	6	23	1	1	1	1	1	1	16	6	6	6	1	6	6
	4	6	6	1	7	7	7	15	6	6	6	7	6	6	27	6	6	6	7	7	6	21	6	6	6	6	6	6
	5	6	6	1	7	7	6	17	6	6	6	6	6	6	28	6	6	6	6	6	6	38	6	6	6	6	5	5
	7	6	6	6	7	7	7	18	6	6	0	0	0	0	29	6	6	6	7	6	0	40	6	6	6	6	6	6
	8	2	3	6	6	6	7	3	5	1	1	6	1	6	30	6	6	6	7	7	7	41	6	6	6	6	0	6
	6	6	6	6	6	6	7	19	6	6	6	6	6	6	31	6	6	5	6	6	0	46	6	6	6	6	6	6
	12	6	6	6	7	7	7	20	6	1	6	6	1	5	34	0	6	1	6	6	6	44	6	6	6	6	6	5
	26	2	2	2	0	0	0	25	3	6	6	7	7	6	37	6	6	6	6	6	6	48	6	6	6	6	6	6
	33	6	6	6	6	6	6	35	6	6	6	7	7	7	45	6	6	6	6	6	7	49	6	6	6	6	6	6
06/15/2022	1	6	6	6	7	8	7	13	1	6	6	6	6	6	22	6	6	6	7	7	7	10	6	6	6	6	6	6
	2	6	6	6	6	6	1	14	1	1	6	7	7	6	23	1	1	1	1	1	1	16	6	6	6	1	6	6
	4	6	6	1	7	7	7	15	6	6	6	7	7	7	27	6	6	6	7	7	6	21	6	6	6	6	6	6
	5	6	6	1	8	8	7	17	6	6	6	7	7	7	28	6	6	6	7	7	6	38	6	6	6	6	6	6
	7	6	6	6	7	7	7	18	6	6	0	0	0	0	29	6	6	6	7	6	0	40	6	6	6	7	7	6
	8	3	4	6	7	6	7	3	5	1	1	6	1	6	30	6	6	6	7	7	7	41	6	6	6	6	0	6
	6	6	6	6	7	7	7	19	6	6	6	7	6	6	31	6	6	5	7	7	0	46	6	6	6	6	6	6
	12	6	6	6	8	8	7	20	6	1	6	6	1	6	34	0	6	1	7	6	6	44	6	6	6	6	6	6
	26	2	2	2	0	0	0	25	3	6	6	7	7	7	37	6	6	6	6	6	6	48	6	6	6	6	6	6
	33	6	6	6	6	6	6	35	6	6	6	7	7	7	45	6	6	6	6	6	7	49	6	6	6	7	7	7

06/18/2022	1	6	6	6	8	8	7	13	1	6	6	6	7	6	22	6	6	6	8	8	7	10	6	6	6	6	6	6
	2	6	6	6	6	6	1	14	1	1	6	7	7	6	23	1	1	1	1	1	1	16	6	6	6	1	6	6
	4	6	6	1	7	7	8	15	6	6	6	7	7	7	27	6	6	6	7	7	7	21	6	6	6	6	6	6
	5	6	6	1	8	8	7	17	6	6	6	7	7	7	28	6	6	6	7	7	7	38	6	6	6	6	6	6
	7	6	6	6	7	8	8	18	6	6	0	0	0	0	29	6	6	6	8	7	0	40	6	6	6	7	7	7
	8	6	6	6	8	6	7	3	6	1	1	7	1	6	30	6	6	6	7	7	7	41	6	6	6	7	0	6
	6	6	6	6	7	7	8	19	6	6	6	8	7	8	31	6	6	5	7	7	0	46	6	6	6	6	6	6
	12	6	6	6	8	8	7	20	6	1	6	6	1	6	34	0	6	1	6	6	6	44	6	6	6	6	6	6
	26	2	2	2	0	0	0	25	3	6	6	7	7	7	37	6	6	6	7	7	7	48	6	6	6	6	6	6
	33	6	6	6	6	6	6	35	6	6	6	7	7	7	45	6	6	6	7	7	8	49	6	6	6	7	7	7
06/21/2022	1	6	6	6	8	8	8	13	1	6	6	6	7	6	22	6	6	6	8	8	8	10	6	6	6	8	8	7
	2	6	6	6	6	6	1	14	1	1	6	8	8	7	23	1	1	1	1	1	1	16	6	6	6	1	6	6
	4	6	6	1	8	8	8	15	6	6	6	8	8	8	27	6	6	6	7	8	8	21	6	6	6	6	6	6
	5	6	6	1	8	8	7	17	6	6	6	7	7	7	28	6	6	6	8	7	7	38	6	6	6	6	7	6
	7	6	6	6	7	8	8	18	6	6	0	0	0	0	29	6	6	6	8	7	0	40	6	6	6	7	7	7
	8	6	6	6	8	6	7	3	5	1	1	7	1	7	30	6	6	6	7	7	7	41	6	6	6	7	0	6
	6	6	6	6	8	7	8	19	6	6	6	8	7	8	31	6	6	6	7	7	0	46	6	6	6	6	6	6
	12	6	6	6	8	8	8	20	6	1	6	8	1	7	34	0	6	1	6	6	6	44	6	6	6	7	7	6
	26	2	2	2	0	0	0	25	3	6	6	8	8	8	37	6	6	6	7	7	7	48	6	6	6	6	6	6
	33	6	6	6	6	6	6	35	6	6	6	7	8	7	45	6	6	6	7	7	8	49	6	6	6	7	7	7
06/24/2022	1	6	6	6	9	8	8	13	1	6	6	8	7	7	22	6	6	6	9	9	9	10	6	6	6	8	8	8
	2	6	6	6	6	6	1	14	1	1	6	8	8	8	23	1	1	1	1	1	1	16	6	6	6	1	6	6
	4	6	6	1	8	8	8	15	6	6	6	9	9	8	27	6	6	6	8	9	8	21	6	6	6	6	6	6
	5	6	6	1	9	9	7	17	6	6	6	8	8	8	28	6	6	6	8	9	8	38	6	6	6	6	8	6
	7	6	6	6	9	8	8	18	6	6	0	0	0	0	29	6	6	6	9	8	0	40	6	6	6	7	8	8
	8	6	6	6	9	7	7	3	6	1	1	8	1	8	30	6	6	6	8	7	8	41	6	6	6	7	0	6
	6	6	6	6	8	8	8	19	6	6	6	9	9	8	31	6	6	6	7	8	0	46	6	6	6	6	6	6
	12	6	6	6	9	9	9	20	6	1	6	8	1	8	34	0	6	1	6	6	6	44	6	6	6	8	8	7
	26	2	2	2	0	0	0	25	3	6	6	9	9	8	37	6	6	6	8	8	8	48	6	6	6	7	7	7
	33	6	6	6	6	6	6	35	6	6	6	8	8	8	45	6	6	6	7	7	8	49	6	6	6	7	7	7
06/27/2022	1	6	6	6	9	9	9	13	1	6	6	9	9	9	22	6	6	6	9	9	9	10	6	6	6	9	8	8
	2	6	6	6	6	6	1	14	1	1	6	9	9	9	23	1	1	1	1	1	1	16	6	6	6	1	6	6
	4	6	6	1	9	9	9	15	6	6	6	9	9	9	27	6	6	6	9	9	9	21	6	6	6	6	6	6
	5	6	6	1	9	9	9	17	6	6	6	9	8	9	28	6	6	6	9	9	8	38	6	6	6	8	9	6
	7	6	6	6	9	9	9	18	6	6	0	0	0	0	29	6	6	6	9	9	0	40	6	6	6	8	9	8
	8	6	6	6	9	7	9	3	6	1	1	9	1	9	30	6	6	6	9	9	9	41	6	6	6	8	0	6
	6	6	6	6	9	9	9	19	6	6	6	9	9	8	31	6	6	6	9	9	0	46	6	6	6	6	6	6
	12	6	6	6	10	10	10	20	6	1	6	9	1	9	34	0	6	1	6	6	6	44	6	6	6	8	8	7
	26	2	2	2	0	0	0	25	3	6	6	9	9	9	37	6	6	6	9	9	9	48	6	6	6	8	8	8
	33	6	6	6	6	6	6	35	6	6	6	9	9	9	45	6	6	6	9	8	9	49	6	6	6	8	8	8
06/30/2022	1	6	6	6	9	9	9	13	1	6	6	9	9	9	22	6	6	6	9	9	9	10	6	6	6	9	9	9
	2	6	6	6	6	6	1	14	1	1	6	9	9	9	23	1	1	1	1	1	1	16	6	6	6	1	6	6
	4	6	6	1	9	9	9	15	6	6	6	9	9	9	27	6	6	6	9	9	9	21	6	6	6	6	6	6
	5	6	6	1	9	9	9	17	6	6	6	9	9	9	28	6	6	6	9	9	9	38	6	6	6	9	9	6
	7	6	6	6	9	9	9	18	6	6	0	0	0	0	29	6	6	6	9	9	0	40	6	6	6	9	9	9
	8	6	6	6	9	8	9	3	6	1	1	9	1	9	30	6	6	6	9	9	9	41	6	6	6	9	0	6
	6	6	6	6	9	9	9	19	6	6	6	9	9	8	31	6	6	6	9	9	0	46	6	6	6	6	6	6
	12	6	6	6	10	10	10	20	6	1	6	9	1	9	34	0	6	1	6	6	6	44	6	6	6	9	9	8
	26	2	2	2	0	0	0	25	3	6	6	9	9	9	37	6	6	6	9	9	9	48	6	6	6	8	8	8
	33	6	6	6	6	6	6	35	6	6	6	9	9	9	45	6	6	6	9	9	9	49	6	6	6	9	9	8
07/03/2022	1	6	6	6	9	9	9	13	1	6	6	9	9	9	22	6	6	6	9	9	9	10	6	6	6	9	9	9
	2	6	6	6	6	6	1	14	1	1	6	9	9	9	23	1	1	1	1	1	1	16	6	6	6	1	6	6
	4	6	6	1	9	9	9	15	6	6	6	9	9	9	27	6	6	6	9	9	9	21	6	6	6	6	6	6
	5	6	6	1	9	9	9	17	6	6	6	9	9	9	28	6	6	6	9	9	9	38	6	6	6	9	9	6
	7	6	6	6	9	9	9	18	6	6	0	0	0	0	29	6	6	6	9	9	0	40	6	6	6	9	9	9
	8	6	6	6	9	8	9	3	6	1	1	9	1	9	30	6	6	6	9	9	9	41	6	6	6	9	0	6
	6	6	6	6	9	9	9	19	6	6	6	9	9	8	31	6	6	6	9	9	0	46	6	6	6	6	6	6
	12	6	6	6	10	10	10	20	6	1	6	9	1	9	34	0	6	1	6	6	6	44	6	6	6	9	9	9
	26	2	2	2	0	0	0	25	3	6	6	9	9	9	37	6	6	6	9	9	9	48	6	6	6	8	8	8
	33	6	6	6	6	6	6	35	6	6	6	9	9	9	45	6	6	6	9	9	9	49	6	6	6	9	9	8
07/06/2022	1	6	6	6	9	9	9	13	1	6	6	9	9	9	22	6	6	6	9	9	9	10	6	6	6	9	9	9
	2	6	6	6	6	6	1	14	1	1	6	9	9	9	23	1	1	1	1	1	1	16	6	6	6	1	6	6
	4	6	6	1	9	9	9	15	6	6	6	9	9	9	27	6	6	6	9	9	9	21	6	6	6	6	6	6
	5	6	6	1	9	9	9	17	6	6	6	9	9	9	28	6	6	6	9	9	9	38	6	6	6	9	9	6
	7	6	6	6	9	9	9	18	6	6	0	0	0	0	29	6	6	6	9	9	0	40	6	6	6	9	9	9
	8	6	6	6	9	8	9	3	6	1	1	9	1	9	30	6	6	6	9	9	9	41	6	6	6	9	0	6
	6	6	6	6	9	9	9	19	6	6	6	9	9	8	31	6	6	6	9	9	0	46	6	6	6	6	6	6
	12	6	6	6	10	10	10	20	6	1	6	9	1	9	34	0	6	1	6	6	6	44	6	6	6	9	9	9
	26	2	2	2	0	0	0	25	3	6	6	9	9	9	37	6	6	6	9	9	9	48	6	6	6	8	8	8
	33	6	6	6	6	6	6	35	6	6	6	9	9	9	45	6	6	6	9	9	9	49	6	6	6	9	9	8

07/09/2022	1	6	6	6	9	9	9	13	1	6	6	9	9	9	22	6	6	6	9	9	9	10	6	6	6	9	9	9
	2	6	6	6	6	6	1	14	1	1	6	9	9	9	23	1	1	1	1	1	1	16	6	6	6	1	6	6
	4	6	6	1	9	9	9	15	6	6	6	9	9	9	27	6	6	6	9	9	9	21	6	6	6	6	6	6
	5	6	6	1	9	9	9	17	6	6	6	9	9	9	28	6	6	6	9	9	9	38	6	6	6	9	9	6
	7	6	6	6	9	9	9	18	6	6	0	0	0	0	29	6	6	6	9	9	0	40	6	6	6	9	9	9
	8	6	6	6	9	8	9	3	6	1	1	9	1	9	30	6	6	6	9	9	9	41	6	6	6	9	0	6
	6	6	6	6	9	9	9	19	6	6	6	9	9	8	31	6	6	6	9	9	0	46	6	6	6	6	6	6
	12	6	6	6	10	10	10	20	6	1	6	9	1	9	34	0	6	1	6	6	6	44	6	6	6	9	9	9
	26	2	2	2	0	0	0	25	3	6	6	9	9	9	37	6	6	6	9	9	9	48	6	6	6	8	8	8
	33	6	6	6	6	6	6	35	6	6	6	9	9	9	45	6	6	6	9	9	9	49	6	6	6	9	9	8
07/12/2022	1	6	6	6	9	9	9	13	1	6	6	9	9	9	22	6	6	6	9	9	9	10	6	6	6	9	9	9
	2	6	6	6	6	6	1	14	1	1	6	9	9	9	23	1	1	1	1	1	1	16	6	6	6	1	6	6
		6	6	1	9	9	9	15	6	6	6	9	9	9	27	6	6	6	9	9	9	21	6	6	6	6	6	6
	5	6	6	1	9	9	9	17	6	6	6	9	9	9	28	6	6	6	9	9	9	38	6	6	6	9	9	6
	7	6	6	6	9	9	9	18	6	6	0	0	0	0	29	6	6	6	9	9	0	40	6	6	6	9	9	9
	8	6	6	6	9	8	9	3	6	1	1	9	1	9	30	6	6	6	9	9	9	41	6	6	6	9	0	6
	6	6	6	6	9	9	9	19	6	6	6	9	9	8	31	6	6	6	9	9	0	46	6	6	6	6	6	6
	12	6	6	6	10	10	10	20	6	1	6	9	1	9	34	0	6	1	6	6	6	44	6	6	6	9	9	9
	26	2	2	2	0	0	0	25	3	6	6	9	9	9	37	6	6	6	9	9	9	48	6	6	6	9	8	8
	33	6	6	6	6	6	6	35	6	6	6	9	9	9	45	6	6	6	9	9	9	49	6	6	6	9	9	8
07/15/2022	1	6	6	6	9	9	9	13	1	6	6	9	9	9	22	6	6	6	9	9	9	10	6	6	6	9	9	9
	2	6	6	6	6	6	1	14	1	1	6	9	9	9	23	1	1	1	1	1	1	16	6	6	6	1	6	6
	4	6	6	1	9	9	9	15	6	6	6	9	9	9	27	6	6	6	9	9	9	21	6	6	6	6	6	6
	5	6	6	1	9	9	9	17	6	6	6	9	9	9	28	6	6	6	9	9	9	38	6	6	6	9	9	6
	7	6	6	6	10	9	10	18	6	6	0	0	0	0	29	6	6	6	9	9	0	40	6	6	6	9	9	9
	8	6	6	6	9	9	9	3	6	1	1	9	1	9	30	6	6	6	9	9	9	41	6	6	6	9	0	6
	6	6	6	6	9	9	9	19	6	6	6	9	9	8	31	6	6	6	9	9	0	46	6	6	6	6	6	6
	12	6	6	6	10	10	10	20	6	1	6	9	1	9	34	0	6	1	6	6	6	44	6	6	6	9	9	9
	26	2	2	2	0	0	0	25	3	6	6	9	9	9	37	6	6	6	9	9	9	48	6	6	6	8	8	8
	33	6	6	6	6	6	6	35	6	6	6	9	9	9	45	6	6	6	9	9	9	49	6	6	6	9	9	8
07/18/2022	1	6	6	6	9	9	9	13	1	6	6	9	9	9	22	6	6	6	9	9	9	10	6	6	6	9	9	9
	2	6	6	6	6	6	1	14	1	1	6	9	9	9	23	1	1	1	1	1	1	16	6	6	6	1	6	6
	4	6	6	1	10	10	9	15	6	6	6	9	9	9	27	6	6	6	9	9	9	21	6	6	6	6	6	6
	5	6	6	1	9	9	9	17	6	6	6	9	9	9	28	6	6	6	9	9	9	38	6	6	6	9	9	6
	7	6	6	6	10	9	10	18	6	6	0	0	0	0	29	6	6	6	9	9	0	40	6	6	6	9	9	9
	8	6	6	6	10	10	10	3	6	1	1	9	1	9	30	6	6	6	9	9	9	41	6	6	6	9	0	6
	6	6	6	6	10	10	10	19	6	6	6	9	9	9	31	6	6	6	9	9	0	46	6	6	6	6	6	6
	12	6	6	6	10	10	10	20	6	1	6	9	1	9	34	0	6	1	6	6	6	44	6	6	6	9	9	9
	26	2	2	2	0	0	0	25	3	6	6	9	9	9	37	6	6	6	9	9	9	48	6	6	6	9	9	8
	33	6	6	6	6	6	6	35	6	6	6	9	9	9	45	6	6	6	9	9	9	49	6	6	6	9	9	8
07/21/2022	1	6	6	6	9	9	9	13	1	6	6	9	9	9	22	6	6	6	9	9	9	10	6	6	6	9	9	9
	2	6	6	6	6	6	1	14	1	1	6	9	9	9	23	1	1	1	1	1	1	16	6	6	6	1	6	6
	4	6	6	1	10	10	10	15	6	6	6	9	9	9	27	6	6	6	9	9	9	21	6	6	6	6	6	6
	5	6	6	1	10	10	10	17	6	6	6	9	9	9	28	6	6	6	9	9	9	38	6	6	6	9	9	6
	7	6	6	6	10	11	11	18	6	6	0	0	0	0	29	6	6	6	9	9	0	40	6	6	6	9	9	9
	8	6	6	6	11	10	10	3	6	1	1	9	1	9	30	6	6	6	9	9	9	41	6	6	6	9	0	6
	6	6	6	6	10	10	10	19	6	6	6	9	9	9	31	6	6	6	9	9	0	46	6	6	6	6	6	6
	12	6	6	6	10	11	10	20	6	1	6	9	1	9	34	0	6	1	6	6	6	44	6	6	6	9	9	9
	26	2	2	2	0	0	0	25	3	6	6	9	9	9	37	6	6	6	9	9	9	48	6	6	6	9	9	8*
	33	6	6	6	6	6	6	35	6	6	6	9	9	9	45	6	6	6	9	9	9	49	6	6	6	9	9	8*
07/24/2022	1	6	6	6	10	11	10	13	1	6	6	9	9	9	22	6	6	6	10	9	9	10	6	6	6	9	9	9
	2	6	6	6	6	6	1	14	1	1	6	9	9	9	23	1	1	1	1	1	1	16	6	6	6	1	6	6
	4	6	6	1	11	10	10	15	6	6	6	10	10	10	27	6	6	6	9	9	9	21	6	6	6	6	6	6
	5	6	6	1	11	11	11	17	6	6	6	10	10	10	28	6	6	6	9	9	9	38	6	6	6	9	9	6
	7	6	6	6	10	11	11	18	6	6	0	0	0	0	29	6	6	6	9	9	0	40	6	6	6	9	9	9
	8	6	6	6	11	11	11	3	6	1	1	9	1	9	30	6	6	6	9	9	9	41	6	6	6	9	0	6
	6	6	6	6	10	10	11	19	6	6	6	9	9	9	31	6	6	6	9	9	0	46	6	6	6	6	6	6
	12	6	6	6	10	11	11	20	6	1	6	9	1	9	34	0	6	1	6	6	6	44	6	6	6	9	9	9
	26	2	2	2	0	0	0	25	3	6	6	11	10	10	37	6	6	6	10	11	11	48	6	6	6	9	9	8*
	33	6	6	6	6	6	6	35	6	6	6	9	9	9	45	6	6	6	9	11	9	49	6	6	6	9	9	8*
07/27/2022	1	6	6	6	11	11	10	13	1	6	6	9	9	9	22	6	6	6	10	9	9	10	6	6	6	9	9	9
	2	6	6	6	6	6	1	14	1	1	6	9	9	9	23	1	1	1	1	1	1	16	6	6	6	1	6	6
	4	6	6	1	11	11	11	15	6	6	6	11	11	10	27	6	6	6	9	11	9	21	6	6	6	6	6	6
	5	6	6	1	11	11	11	17	6	6	6	10	10	11	28	6	6	6	9	10	9	38	6	6	6	9	9	6
	7	6	6	6	11	11	11	18	6	6	0	0	0	0	29	6	6	6	9	9	0	40	6	6	6	9	9	9
	8	6	6	6	11	11	11	3	6	1	1	9	1	9	30	6	6	6	9	9	9	41	6	6	6	9	0	6
	6	6	6	6	10	10	11	19	6	6	6	11	9	9	31	6	6	6	9	9	0	46	6	6	6	6	6	6
	12	6	6	6	10	11	11	20	6	1	6	9	1	9	34	0	6	1	6	6	6	44	6	6	6	9	9	9
	26	2	2	2	0	0	0	25	3	6	6	11	10	10	37	6	6	6	10	11	11	48	6	6	6	9	9	8*
	33	6	6	6	6	6	6	35	6	6	6	9	9	9	45	6	6	6	9	11	9	49	6	6	6	9	9	8*

07/30/2022	1	6	6	6	11	11	11	13	1	6	6	9	9	9	22	6	6	6	10	10	9	10	6	6	6	9	9	9
	2	6	6	6	6	6	1	14	1	1	6	9	9	9	23	1	1	1	1	1	1	16	6	6	6	1	6	6
	4	6	6	1	11	11	11	15	6	6	6	11	11	10	27	6	6	6	10	11	10	21	6	6	6	6	6	6
	5	6	6	1	11	11	11	17	6	6	6	11	11	11	28	6	6	6	10	10	10	38	6	6	6	9	9	6
	7	6	6	6	11	11	11	18	6	6	0	0	0	0	29	6	6	6	9	10	0	40	6	6	6	9	9	9
	8	6	6	6	11	11	11	3	6	1	1	9	1	9	30	6	6	6	9	9	9	41	6	6	6	9	0	6
	6	6	6	6	10	10	11	19	6	6	6	11	10	9	31	6	6	6	9	9	0	46	6	6	6	6	6	6
	12	6	6	6	10	11	11	20	6	1	6	10	1	9	34	0	6	1	6	6	6	44	6	6	6	9	9	9
	26	2	2	2	0	0	0	25	3	6	6	11	10	10	37	6	6	6	10	11	11	48	6	6	6	9	9	8*
	33	6	6	6	6	6	6	35	6	6	6	10	9	9	45	6	6	6	9	11	9	49	6	6	6	9	9	8*
08/01/2022	1	6	6	6	11	11	11	13	1	6	6	10	10	10	22	6	6	6	10	10	9	10	6	6	6	11	11	10
	2	6	6	6	6	6	1	14	1	1	6	9	11	9	23	1	1	1	1	1	1	16	6	6	6	1	6	6
	4	6	6	1	11	11	11	15	6	6	6	11	11	11	27	6	6	6	10	11	10	21	6	6	6	6	6	6
	5	6	6	1	11	11	11	17	6	6	6	11	11	11	28	6	6	6	11	11	11	38	6	6	6	9	11	6
	7	6	6	6	11	11	11	18	6	6	0	0	0	0	29	6	6	6	9	11	0	40	6	6	6	9	9	9
	8	6	6	6	11	11	11	3	6	1	1	9	1	9	30	6	6	6	11	9	9	41	6	6	6	9	0	6
	6	6	6	6	10	10	11	19	6	6	6	11	10	11	31	6	6	6	9	9	0	46	6	6	6	6	6	6
	12	6	6	6	10	11	11	20	6	1	6	11	1	10	34	0	6	1	6	6	6	44	6	6	6	9	9	9
	26	2	2	2	0	0	0	25	3	6	6	11	11	11	37	6	6	6	10	11	11	48	6	6	6	9	9	8*
	33	6	6	6	6	6	6	35	6	6	6	11	10	10	45	6	6	6	9	11	9	49	6	6	6	9	9	8*
08/04/2022	1	6	6	6	11	11	11	13	1	6	6	11	11	11	22	6	6	6	11	10	9	10	6	6	6	11	11	10
	2	6	6	6	6	6	1	14	1	1	6	11	11	10	23	1	1	1	1	1	1	16	6	6	6	1	6	6
	4	6	6	1	11	11	11	15	6	6	6	11	11	11	27	6	6	6	10	11	11	21	6	6	6	6	6	6
	5	6	6	1	11	11	11	17	6	6	6	11	11	11	28	6	6	6	11	11	11	38	6	6	6	9	11	6
	7	6	6	6	11	11	11	18	6	6	0	0	0	0	29	6	6	6	9	11	0	40	6	6	6	9	9	9
	8	6	6	6	11	11	11	3	6	1	1	10	1	9	30	6	6	6	11	10	9	41	6	6	6	9	0	6
	6	6	6	6	11	11	11	19	6	6	6	11	10	11	31	6	6	6	9	9	0	46	6	6	6	6	6	6
	12	6	6	6	10	11	11	20	6	1	6	11	1	11	34	0	6	1	6	6	6	44	6	6	6	9	10	9
	26	2	2	2	0	0	0	25	3	6	6	11	11	11	37	6	6	6	10	11	11	48	6	6	6	9	9	8*
	33	6	6	6	6	6	6	35	6	6	6	11	11	10	45	6	6	6	10	11	10	49	6	6	6	9	9	8*
08/07/2022	1	6	6	6	11	11	11	13	1	6	6	11	11	11	22	6	6	6	11	10	10	10	6	6	6	11	11	10
	2	6	6	6	6	6	1	14	1	1	6	11	11	10	23	1	1	1	1	1	1	16	6	6	6	1	6	6
	4	6	6	1	11	11	11	15	6	6	6	11	11	11	27	6	6	6	10	11	11	21	6	6	6	6	6	6
	5	6	6	1	11	11	11	17	6	6	6	11	11	11	28	6	6	6	11	11	11	38	6	6	6	9	11	6
	7	6	6	6	11	11	11	18	6	6	0	0	0	0	29	6	6	6	9	11	0	40	6	6	6	9	9	9
	8	6	6	6	11	11	11	3	6	1	1	10	1	9	30	6	6	6	11	10	9	41	6	6	6	9	0	6
	6	6	6	6	11	11	11	19	6	6	6	11	11	11	31	6	6	6	9	9	0	46	6	6	6	6	6	6
	12	6	6	6	11	11	11	20	6	1	6	11	1	11	34	0	6	1	6	6	6	44	6	6	6	9	10	9
	26	2	2	2	0	0	0	25	3	6	6	11	11	11	37	6	6	6	10	11	11	48	6	6	6	9	9	8*
	33	6	6	6	6	6	6	35	6	6	6	11	11	10	45	6	6	6	10	11	10	49	6	6	6	9	9	8*
08/11/2022	1	6	6	6	11	11	11	13	1	6	6	11	11	11	22	6	6	6	11	10	11	10	6	6	6	11	11	10
	2	6	6	6	6	6	1	14	1	1	6	11	11	11	23	1	1	1	1	1	1	16	6	6	6	1	6	6
	4	6	6	1	11	11	11	15	6	6	6	11	11	11	27	6	6	6	10	11	11	21	6	6	6	6	6	6
	5	6	6	1	11	11	11	17	6	6	6	11	11	11	28	6	6	6	11	11	11	38	6	6	6	9	11	6
	7	6	6	6	11	11	11	18	6	6	0	0	0	0	29	6	6	6	9	11	0	40	6	6	6	9	9	9
	8	6	6	6	11	11	11	3	6	1	1	10	1	9	30	6	6	6	11	10	10	41	6	6	6	9	0	6
	6	6	6	6	11	11	11	19	6	6	6	11	11	11	31	6	6	6	9	9	0	46	6	6	6	6	6	6
	12	6	6	6	11	11	11	20	6	1	6	11	1	11	34	0	6	1	6	6	6	44	6	6	6	9	10	9
	26	2	2	2	0	0	0	25	3	6	6	11	11	11	37	6	6	6	10	11	11	48	6	6	6	9	9	8*
	33	6	6	6	6	6	6	35	6	6	6	11	11	10	45	6	6	6	10	11	10	49	6	6	6	9	9	8*
08/14/2022	1	6	6	6	11	11	11	13	1	6	6	11	11	11	22	6	6	6	11	10	11	10	6	6	6	11	11	11
	2	6	6	6	6	6	1	14	1	1	6	11	11	11	23	1	1	1	1	1	1	16	6	6	6	1	6	6
	4	6	6	1	11	11	11	15	6	6	6	11	11	11	27	6	6	6	11	11	11	21	6	6	6	6	6	6
	5	6	6	1	11	11	11	17	6	6	6	11	11	11	28	6	6	6	11	11	11	38	6	6	6	9	11	6
	7	6	6	6	11	11	11	18	6	6	0	0	0	0	29	6	6	6	10	11	0	40	6	6	6	9	9	9
	8	6	6	6	11	11	11	3	6	1	1	11	1	10	30	6	6	6	11	10	10	41	6	6	6	9	0	6
	6	6	6	6	11	11	11	19	6	6	6	11	11	11	31	6	6	6	9	9	0	46	6	6	6	6	6	6
	12	6	6	6	11	11	11	20	6	1	6	11	1	11	34	0	6	1	6	6	6	44	6	6	6	10	11	10
	26	2	2	2	0	0	0	25	3	6	6	11	11	11	37	6	6	6	11	11	11	48	6	6	6	9	9	8*
	33	6	6	6	6	6	6	35	6	6	6	11	11	11	45	6	6	6	11	11	11	49	6	6	6	9	9	8*
08/17/2022	1	6	6	6	11	11	11	13	1	6	6	11	11	11	22	6	6	6	11	10	11	10	6	6	6	11	11	11
	2	6	6	6	6	6	1	14	1	1	6	11	11	11	23	1	1	1	1	1	1	16	6	6	6	1	6	6
	4	6	6	1	11	11	11	15	6	6	6	11	11	11	27	6	6	6	11	11	11	21	6	6	6	6	6	6
	5	6	6	1	11	11	11	17	6	6	6	11	11	11	28	6	6	6	11	11	11	38	6	6	6	9	11	6
	7	6	6	6	11	11	11	18	6	6	0	0	0	0	29	6	6	6	10	11	0	40	6	6	6	9	9	9
	8	6	6	6	11	11	11	3	6	1	1	11	1	10	30	6	6	6	11	10	10	41	6	6	6	9	0	6
	6	6	6	6	11	11	11	19	6	6	6	11	11	11	31	6	6	6	9	9	0	46	6	6	6	6	6	6
	12	6	6	6	11	11	11	20	6	1	6	11	1	11	34	0	6	1	6	6	6	44	6	6	6	10	11	10
	26	2	2	2	0	0	0	25	3	6	6	11	11	11	37	6	6	6	11	11	11	48	6	6	6	9	9	8*
	33	6	6	6	6	6	6	35	6	6	6	11	11	11	45	6	6	6	11	11	11	49	6	6	6	9	9	8*

08/20/2022	1	6	6	6	11	11	11	13	1	6	6	11	11	11	22	6	6	6	11	10	11	10	6	6	6	11	11	11
	2	6	6	6	6	6	1	14	1	1	6	11	11	11	23	1	1	1	1	1	1	16	6	6	6	1	6	6
	4	6	6	1	11	11	11	15	6	6	6	11	11	11	27	6	6	6	11	11	11	21	6	6	6	6	6	6
	5	6	6	1	11	11	11	17	6	6	6	11	11	11	28	6	6	6	11	11	11	38	6	6	6	9	11	6
	7	6	6	6	11	11	11	18	6	6	0	0	0	0	29	6	6	6	10	11	0	40	6	6	6	9	9	9
	8	6	6	6	11	11	11	3	6	1	1	11	1	10	30	6	6	6	11	10	10	41	6	6	6	9	0	6
	6	6	6	6	11	11	11	19	6	6	6	11	11	11	31	6	6	6	9	9	0	46	6	6	6	6	6	6
	12	6	6	6	11	11	11	20	6	1	6	11	1	11	34	0	6	1	6	6	6	44	6	6	6	10	11	10
	26	2	2	2	0	0	0	25	3	6	6	11	11	11	37	6	6	6	11	11	11	48	6	6	6	9	9	8*
	33	6	6	6	6	6	6	35	6	6	6	11	11	11	45	6	6	6	11	11	11	49	6	6	6	9	9	8*
08/23/2022	1	6	6	6	11	11	11	13	1	6	6	11	11	11	22	6	6	6	11	10	11	10	6	6	6	11	11	11
	2	6	6	6	6	6	1	14	1	1	6	11	11	11	23	1	1	1	1	1	1	16	6	6	6	1	6	6
	4	6	6	1	11	11	11	15	6	6	6	11	11	11	27	6	6	6	11	11	11	21	6	6	6	6	6	6
	5	6	6	1	11	11	11	17	6	6	6	11	11	11	28	6	6	6	11	11	11	38	6	6	6	9	11	6
	7	6	6	6	11	11	11	18	6	6	0	0	0	0	29	6	6	6	10	11	0	40	6	6	6	9	9	9
	8	6	6	6	11	11	11	3	6	1	1	11	1	10	30	6	6	6	11	10	10	41	6	6	6	9	0	6
	6	6	6	6	11	11	11	19	6	6	6	11	11	11	31	6	6	6	9	9	0	46	6	6	6	6	6	6
	12	6	6	6	11	11	11	20	6	1	6	11	1	11	34	0	6	1	6	6	6	44	6	6	6	11	11	11
	26	2	2	2	0	0	0	25	3	6	6	11	11	11	37	6	6	6	11	11	11	48	6	6	6	9	9	8*
	33	6	6	6	6	6	6	35	6	6	6	11	11	11	45	6	6	6	11	11	11	49	6	6	6	9	9	8*
08/26/2022	1	6	6	6	11	11	11	13	1	6	6	11	11	11	22	6	6	6	11	10	11	10	6	6	6	11	11	11
	2	6	6	6	6	6	1	14	1	1	6	11	11	11	23	1	1	1	1	1	1	16	6	6	6	1	6	6
	4	6	6	1	11	11	11	15	6	6	6	11	11	11	27	6	6	6	11	11	11	21	6	6	6	6	6	6
	5	6	6	1	11	11	11	17	6	6	6	11	11	11	28	6	6	6	11	11	11	38	6	6	6	9	11	6
	7	6	6	6	11	11	11	18	6	6	0	0	0	0	29	6	6	6	10	11	0	40	6	6	6	9	9	9
	8	6	6	6	11	11	11	3	6	1	1	11	1	10	30	6	6	6	11	10	10	41	6	6	6	9	0	6
	6	6	6	6	11	11	11	19	6	6	6	11	11	11	31	6	6	6	9	9	0	46	6	6	6	6	6	6
	12	6	6	6	11	11	11	20	6	1	6	11	1	11	34	0	6	1	6	6	6	44	6	6	6	11	11	11
	26	2	2	2	0	0	0	25	3	6	6	11	11	11	37	6	6	6	11	11	11	48	6	6	6	9	9	8*
	33	6	6	6	6	6	6	35	6	6	6	11	11	11	45	6	6	6	11	11	11	49	6	6	6	9	9	8*
08/29/2022	1	6	6	6	11	11	11	13	1	6	6	11	11	11	22	6	6	6	11	10	11	10	6	6	6	11	11	11
	2	6	6	6	6	6	1	14	1	1	6	11	11	11	23	1	1	1	1	1	1	16	6	6	6	1	6	6
	4	6	6	1	11	11	11	15	6	6	6	11	11	11	27	6	6	6	11	11	11	21	6	6	6	6	6	6
	5	6	6	1	11	11	11	17	6	6	6	11	11	11	28	6	6	6	11	11	11	38	6	6	6	9	11	6
	7	6	6	6	11	11	11	18	6	6	0	0	0	0	29	6	6	6	10	11	0	40	6	6	6	9	9	9
	8	6	6	6	11	11	11	3	6	1	1	11	1	10	30	6	6	6	11	10	10	41	6	6	6	9	0	6
	6	6	6	6	11	11	11	19	6	6	6	11	11	11	31	6	6	6	9	9	0	46	6	6	6	6	6	6
	12	6	6	6	11	11	11	20	6	1	6	11	1	11	34	0	6	1	6	6	6	44	6	6	6	11	11	11
	26	2	2	2	0	0	0	25	3	6	6	11	11	11	37	6	6	6	11	11	11	48	6	6	6	9	9	8*
	33	6	6	6	6	6	6	35	6	6	6	11	11	11	45	6	6	6	11	11	11	49	6	6	6	9	9	8*
09/01/2022	1	6	6	6	11	11	11	13	1	6	6	11	11	11	22	6	6	6	11	11	11	10	6	6	6	11	11	11
	2	6	6	6	6	6	1	14	1	1	6	11	11	11	23	1	1	1	1	1	1	16	6	6	6	1	6	6
	4	6	6	1	11	11	11	15	6	6	6	11	11	11	27	6	6	6	11	11	11	21	6	6	6	6	6	6
	5	6	6	1	11	11	11	17	6	6	6	11	11	11	28	6	6	6	11	11	11	38	6	6	6	9	11	6
	7	6	6	6	11	11	11	18	6	6	0	0	0	0	29	6	6	6	10	11	0	40	6	6	6	9	9	9
	8	6	6	6	11	11	11	3	6	1	1	11	1	10	30	6	6	6	11	10	10	41	6	6	6	9	0	6
	6	6	6	6	11	11	11	19	6	6	6	11	11	11	31	6	6	6	9	9	0	46	6	6	6	6	6	6
	12	6	6	6	11	11	11	20	6	1	6	11	1	11	34	0	6	1	6	6	6	44	6	6	6	11	11	11
	26	2	2	2	0	0	0	25	3	6	6	11	11	11	37	6	6	6	11	11	11	48	6	6	6	9	9	8*
	33	6	6	6	6	6	6	35	6	6	6	11	11	11	45	6	6	6	11	11	11	49	6	6	6	9	9	8*
09/03/2022	1	6	6	6	11	11	11	13	1	6	6	11	11	11	22	6	6	6	11	11	11	10	6	6	6	11	11	11
	2	6	6	6	6	6	1	14	1	1	6	11	11	11	23	1	1	1	1	1	1	16	6	6	6	1	6	6
	4	6	6	1	11	11	11	15	6	6	6	11	11	11	27	6	6	6	11	11	11	21	6	6	6	6	6	6
	5	6	6	1	11	11	11	17	6	6	6	11	11	11	28	6	6	6	11	11	11	38	6	6	6	9	11	6
	7	6	6	6	11	11	11	18	6	6	0	0	0	0	29	6	6	6	10	11	0	40	6	6	6	9	9	9
	8	6	6	6	11	11	11	3	6	1	1	11	1	10	30	6	6	6	11	10	10	41	6	6	6	9	0	6
	6	6	6	6	11	11	11	19	6	6	6	11	11	11	31	6	6	6	9	9	0	46	6	6	6	6	6	6
	12	6	6	6	11	11	11	20	6	1	6	11	1	11	34	0	6	1	6	6	6	44	6	6	6	11	11	11
	26	2	2	2	0	0	0	25	3	6	6	11	11	11	37	6	6	6	11	11	11	48	6	6	6	9	9	8*
	33	6	6	6	6	6	6	35	6	6	6	11	11	11	45	6	6	6	11	11	11	49	6	6	6	9	9	8*
09/07/2022	1	6	6	6	11	11	11	13	1	6	6	11	11	11	22	6	6	6	11	11	11	10	6	6	6	11	11	11
	2	6	6	6	6	6	1	14	1	1	6	11	11	11	23	1	1	1	1	1	1	16	6	6	6	1	6	6
	4	6	6	1	11	11	11	15	6	6	6	11	11	11	27	6	6	6	11	11	11	21	6	6	6	6	6	6
	5	6	6	1	11	11	11	17	6	6	6	11	11	11	28	6	6	6	11	11	11	38	6	6	6	9	11	6
	7	6	6	6	11	11	11	18	6	6	0	0	0	0	29	6	6	6	10	11	0	40	6	6	6	9	9	9
	8	6	6	6	11	11	11	3	6	1	1	11	1	10	30	6	6	6	11	10	10	41	6	6	6	9	0	6
	6	6	6	6	11	11	11	19	6	6	6	11	11	11	31	6	6	6	9	9	0	46	6	6	6	6	6	6
	12	6	6	6	11	11	11	20	6	1	6	11	1	11	34	0	6	1	6	6	6	44	6	6	6	11	11	11
	26	2	2	2	0	0	0	25	3	6	6	11	11	11	37	6	6	6	11	11	11	48	6	6	6	9	9	8*
	33	6	6	6	6	6	6	35	6	6	6	11	11	11	45	6	6	6	11	11	11	49	6	6	6	9	9	8*

09/10/2022	1	6	6	6	11	11	11	13	1	6	6	11	11	11	22	6	6	6	11	11	11	10	6	6	6	11	11	11
	2	6	6	6	6	6	1	14	1	1	6	11	11	11	23	1	1	1	1	1	1	16	6	6	6	1	6	6
	4	6	6	1	11	11	11	15	6	6	6	11	11	11	27	6	6	6	11	11	11	21	6	6	6	6	6	6
	5	6	6	1	11	11	11	17	6	6	6	11	11	11	28	6	6	6	11	11	11	38	6	6	6	9	11	6
	7	6	6	6	11	11	11	18	6	6	0	0	0	0	29	6	6	6	10	11	0	40	6	6	6	9	9	9
	8	6	6	6	11	11	11	3	6	1	1	11	1	10	30	6	6	6	11	10	10	41	6	6	6	9	0	6
	6	6	6	6	11	11	11	19	6	6	6	11	11	11	31	6	6	6	9	9	0	46	6	6	6	6	6	6
	12	6	6	6	11	11	11	20	6	1	6	11	1	11	34	0	6	1	6	6	6	44	6	6	6	11	11	11
	26	2	2	2	0	0	0	25	3	6	6	11	11	11	37	6	6	6	11	11	11	48	6	6	6	9	9	8*
	33	6	6	6	6	6	6	35	6	6	6	11	11	11	45	6	6	6	11	11	11	49	6	6	6	9	9	8*
09/13/2022	1	6	6	6	11	11	11	13	1	6	6	11	11	11	22	6	6	6	11	11	11	10	6	6	6	11	11	11
	2	6	6	6	6	6	1	14	1	1	6	11	11	11	23	1	1	1	1	1	1	16	6	6	6	1	6	6
	4	6	6	1	11	11	11	15	6	6	6	11	11	11	27	6	6	6	11	11	11	21	6	6	6	6	6	6
	5	6	6	1	11	11	11	17	6	6	6	11	11	11	28	6	6	6	11	11	11	38	6	6	6	9	11	6
	7	6	6	6	11	11	11	18	6	6	0	0	0	0	29	6	6	6	10	11	0	40	6	6	6	9	9	9
	8	6	6	6	11	11	11	3	6	1	1	11	1	10	30	6	6	6	11	10	10	41	6	6	6	9	0	6
	6	6	6	6	11	11	11	19	6	6	6	11	11	11	31	6	6	6	9	9	0	46	6	6	6	6	6	6
	12	6	6	6	11	11	11	20	6	1	6	11	1	11	34	0	6	1	6	6	6	44	6	6	6	11	11	11
	26	2	2	2	0	0	0	25	3	6	6	11	11	11	37	6	6	6	11	11	11	48	6	6	6	9	9	8*
	33	6	6	6	6	6	6	35	6	6	6	11	11	11	45	6	6	6	11	11	11	49	6	6	6	9	9	8*

Table S3: Hourly air temperature under 0% shade from May 10 to June 22, 2023.

	Port 1	Port 2	Port 3	Average
	5TE Moisture/ Temp/EC	5TE Moisture/ Temp/EC	5TE Moisture/ Temp/EC	5TE Moisture/ Temp/EC
Measurement Time	°C Temp	°C Temp	°C Temp	°C Temp
2022-05-11 1:32 PM	27.2	19.0	#N/A	#N/A
2022-05-11 1:33 PM	26.9	19.0	22.6	22.8
2022-05-11 2:00 PM	26.4	19.2	23.4	23.0
2022-05-11 3:00 PM	25.8	20.1	25.6	23.8
2022-05-11 4:00 PM	24.4	22.3	29.0	25.2
2022-05-11 5:00 PM	22.0	21.0	25.6	22.9
2022-05-11 6:00 PM	18.5	17.6	19.2	18.4
2022-05-11 7:00 PM	17.2	16.6	17.6	17.1
2022-05-11 8:00 PM	16.5	16.0	17.0	16.5
2022-05-11 9:00 PM	15.4	15.2	16.1	15.6
2022-05-11 10:00 PM	13.6	14.1	14.0	13.9
2022-05-11 11:00 PM	12.5	13.3	13.1	13.0
2022-05-12 12:00 AM	12.0	12.8	12.6	12.5
2022-05-12 1:00 AM	11.4	12.3	12.0	11.9
2022-05-12 2:00 AM	12.3	12.6	12.9	12.6
2022-05-12 3:00 AM	13.1	12.9	13.5	13.2
2022-05-12 4:00 AM	9.8	11.0	10.2	10.3
2022-05-12 5:00 AM	10.2	11.1	10.5	10.6
2022-05-12 6:00 AM	10.8	11.4	11.1	11.1
2022-05-12 7:00 AM	10.6	11.3	10.9	10.9
2022-05-12 8:00 AM	10.0	10.9	10.6	10.5
2022-05-12 9:00 AM	10.7	11.3	11.0	11.0
2022-05-12 10:00 AM	11.0	11.4	11.1	11.2
2022-05-12 11:00 AM	11.6	11.8	11.7	11.7
2022-05-12 12:00 PM	14.1	13.4	14.0	13.8
2022-05-12 1:00 PM	17.9	15.1	16.4	16.5
2022-05-12 2:00 PM	21.1	17.2	19.4	19.2
2022-05-12 3:00 PM	22.0	17.6	20.2	19.9
2022-05-12 4:00 PM	17.9	16.2	17.7	17.3
2022-05-12 5:00 PM	16.4	15.4	16.4	16.1
2022-05-12 6:00 PM	16.5	15.6	17.1	16.4
2022-05-12 7:00 PM	15.6	15.3	16.5	15.8
2022-05-12 8:00 PM	14.7	14.6	15.1	14.8
2022-05-12 9:00 PM	12.3	13.1	12.6	12.7
2022-05-12 10:00 PM	11.6	12.5	11.8	12.0
2022-05-12 11:00 PM	11.7	12.4	12.0	12.0
2022-05-13 12:00 AM	11.5	12.3	11.9	11.9

2022-05-13 1:00 AM	11.0	11.9	11.3	11.4
2022-05-13 2:00 AM	10.5	11.4	10.8	10.9
2022-05-13 3:00 AM	11.3	11.9	11.6	11.6
2022-05-13 4:00 AM	10.4	11.3	10.6	10.8
2022-05-13 5:00 AM	10.3	11.2	10.7	10.7
2022-05-13 6:00 AM	10.3	11.2	10.7	10.7
2022-05-13 7:00 AM	9.6	10.5	9.9	10.0
2022-05-13 8:00 AM	9.8	10.8	10.3	10.3
2022-05-13 9:00 AM	10.3	11.0	10.7	10.7
2022-05-13 10:00 AM	11.2	11.8	11.8	11.6
2022-05-13 11:00 AM	13.8	13.4	13.4	13.5
2022-05-13 12:00 PM	20.9	16.2	14.5	17.2
2022-05-13 1:00 PM	22.4	16.2	16.3	18.3
2022-05-13 2:00 PM	23.0	16.8	18.5	19.4
2022-05-13 3:00 PM	24.2	18.0	22.7	21.6
2022-05-13 4:00 PM	23.8	22.1	30.3	25.4
2022-05-13 5:00 PM	23.3	25.2	36.8	28.4
2022-05-13 6:00 PM	20.2	22.8	38.1	27.0
2022-05-13 7:00 PM	16.4	18.2	25.1	19.9
2022-05-13 8:00 PM	14.6	15.8	16.7	15.7
2022-05-13 9:00 PM	13.8	15.0	15.1	14.6
2022-05-13 10:00 PM	11.7	13.4	12.4	12.5
2022-05-13 11:00 PM	10.2	12.2	10.8	11.1
2022-05-14 12:00 AM	9.7	11.7	9.9	10.4
2022-05-14 1:00 AM	10.2	11.8	10.2	10.7
2022-05-14 2:00 AM	9.9	11.5	10.4	10.6
2022-05-14 3:00 AM	9.8	11.3	10.0	10.4
2022-05-14 4:00 AM	9.6	11.2	10.0	10.3
2022-05-14 5:00 AM	9.2	10.8	9.2	9.7
2022-05-14 6:00 AM	8.5	10.3	8.9	9.2
2022-05-14 7:00 AM	7.8	9.6	7.9	8.4
2022-05-14 8:00 AM	8.8	10.4	9.0	9.4
2022-05-14 9:00 AM	9.8	11.1	10.9	10.6
2022-05-14 10:00 AM	11.7	12.6	12.3	12.2
2022-05-14 11:00 AM	16.2	14.8	14.1	15.0
2022-05-14 12:00 PM	24.0	17.7	14.5	18.7
2022-05-14 1:00 PM	25.6	16.8	16.2	19.5
2022-05-14 2:00 PM	26.4	17.6	19.0	21.0
2022-05-14 3:00 PM	26.3	18.6	23.0	22.6
2022-05-14 4:00 PM	26.6	24.5	35.0	28.7
2022-05-14 5:00 PM	24.8	26.7	37.5	29.7
2022-05-14 6:00 PM	21.4	21.7	31.2	24.8
2022-05-14 7:00 PM	21.3	20.9	31.1	24.4

2022-05-14 8:00 PM	19.7	19.1	20.1	19.6
2022-05-14 9:00 PM	17.9	17.8	18.1	17.9
2022-05-14 10:00 PM	15.4	16.3	16.3	16.0
2022-05-14 11:00 PM	14.6	15.4	15.7	15.2
2022-05-15 12:00 AM	13.3	14.3	14.4	14.0
2022-05-15 1:00 AM	11.9	13.5	13.1	12.8
2022-05-15 2:00 AM	10.9	12.8	12.2	12.0
2022-05-15 3:00 AM	10.2	12.2	11.4	11.3
2022-05-15 4:00 AM	9.2	11.4	10.1	10.2
2022-05-15 5:00 AM	9.1	11.1	9.4	9.9
2022-05-15 6:00 AM	9.0	10.8	8.6	9.5
2022-05-15 7:00 AM	8.1	9.9	7.9	8.6
2022-05-15 8:00 AM	8.3	10.0	8.5	8.9
2022-05-15 9:00 AM	10.3	11.2	11.0	10.8
2022-05-15 10:00 AM	12.7	12.8	12.8	12.8
2022-05-15 11:00 AM	14.6	14.0	13.9	14.2
2022-05-15 12:00 PM	17.1	15.0	15.2	15.8
2022-05-15 1:00 PM	19.8	16.0	16.7	17.5
2022-05-15 2:00 PM	22.2	17.2	19.0	19.5
2022-05-15 3:00 PM	20.1	16.9	19.1	18.7
2022-05-15 4:00 PM	19.2	18.0	23.3	20.2
2022-05-15 5:00 PM	19.4	18.9	24.5	20.9
2022-05-15 6:00 PM	18.1	17.2	19.5	18.3
2022-05-15 7:00 PM	17.0	16.4	17.2	16.9
2022-05-15 8:00 PM	15.9	15.6	16.5	16.0
2022-05-15 9:00 PM	14.7	14.8	15.7	15.1
2022-05-15 10:00 PM	14.1	14.3	14.6	14.3
2022-05-15 11:00 PM	12.7	13.5	13.3	13.2
2022-05-16 12:00 AM	11.5	12.8	12.5	12.3
2022-05-16 1:00 AM	11.4	12.2	12.3	12.0
2022-05-16 2:00 AM	10.7	11.4	10.9	11.0
2022-05-16 3:00 AM	9.6	10.4	9.5	9.8
2022-05-16 4:00 AM	8.9	9.9	8.8	9.2
2022-05-16 5:00 AM	8.6	9.7	8.7	9.0
2022-05-16 6:00 AM	8.2	9.5	8.8	8.8
2022-05-16 7:00 AM	8.5	9.6	8.6	8.9
2022-05-16 8:00 AM	8.9	9.8	8.6	9.1
2022-05-16 9:00 AM	9.7	10.1	9.2	9.7
2022-05-16 10:00 AM	10.5	10.5	9.7	10.2
2022-05-16 11:00 AM	11.1	9.9	8.9	10.0
2022-05-16 12:00 PM	15.4	11.6	11.3	12.8
2022-05-16 1:00 PM	16.7	12.3	13.4	14.1
2022-05-16 2:00 PM	20.5	13.1	15.4	16.3

2022-05-16 3:00 PM	18.4	13.8	17.1	16.4
2022-05-16 4:00 PM	20.3	16.8	24.4	20.5
2022-05-16 5:00 PM	17.6	15.7	20.4	17.9
2022-05-16 6:00 PM	15.5	13.9	16.3	15.2
2022-05-16 7:00 PM	13.7	12.7	14.7	13.7
2022-05-16 8:00 PM	12.4	11.8	12.8	12.3
2022-05-16 9:00 PM	11.3	11.1	11.4	11.3
2022-05-16 10:00 PM	9.6	10.1	9.7	9.8
2022-05-16 11:00 PM	9.6	10.1	9.5	9.7
2022-05-17 12:00 AM	8.4	9.4	8.5	8.8
2022-05-17 1:00 AM	7.4	9.0	7.4	7.9
2022-05-17 2:00 AM	7.3	8.7	7.0	7.7
2022-05-17 3:00 AM	6.1	8.0	5.6	6.6
2022-05-17 4:00 AM	5.8	7.8	5.7	6.4
2022-05-17 5:00 AM	5.3	7.3	4.8	5.8
2022-05-17 6:00 AM	4.5	6.6	3.2	4.8
2022-05-17 7:00 AM	3.8	6.0	2.3	4.0
2022-05-17 8:00 AM	4.3	6.0	3.4	4.6
2022-05-17 9:00 AM	5.8	7.1	5.8	6.2
2022-05-17 10:00 AM	7.1	7.9	6.9	7.3
2022-05-17 11:00 AM	10.4	9.6	8.3	9.4
2022-05-17 12:00 PM	17.1	12.8	10.5	13.5
2022-05-17 1:00 PM	17.5	12.3	12.9	14.2
2022-05-17 2:00 PM	18.7	12.8	15.4	15.6
2022-05-17 3:00 PM	20.3	14.4	20.4	18.4
2022-05-17 4:00 PM	20.6	19.6	30.7	23.6
2022-05-17 5:00 PM	18.9	21.4	34.1	24.8
2022-05-17 6:00 PM	15.3	17.5	30.2	21.0
2022-05-17 7:00 PM	12.8	14.0	19.1	15.3
2022-05-17 8:00 PM	11.9	12.7	13.9	12.8
2022-05-17 9:00 PM	10.7	11.8	11.6	11.4
2022-05-17 10:00 PM	8.7	10.6	8.9	9.4
2022-05-17 11:00 PM	7.6	9.6	7.3	8.2
2022-05-18 12:00 AM	6.8	8.9	6.2	7.3
2022-05-18 1:00 AM	6.3	8.4	5.3	6.7
2022-05-18 2:00 AM	6.4	8.4	5.1	6.6
2022-05-18 3:00 AM	7.0	8.6	5.7	7.1
2022-05-18 4:00 AM	7.3	8.9	7.0	7.7
2022-05-18 5:00 AM	7.3	8.8	6.5	7.5
2022-05-18 6:00 AM	7.3	8.6	6.3	7.4
2022-05-18 7:00 AM	7.5	8.7	6.8	7.7
2022-05-18 8:00 AM	7.9	8.9	7.8	8.2
2022-05-18 9:00 AM	7.9	9.2	8.1	8.4

2022-05-18 10:00 AM	9.3	10.3	9.4	9.7
2022-05-18 11:00 AM	9.8	10.8	10.2	10.3
2022-05-18 12:00 PM	10.2	10.9	10.4	10.5
2022-05-18 1:00 PM	9.6	10.2	9.8	9.9
2022-05-18 2:00 PM	11.4	11.4	11.8	11.5
2022-05-18 3:00 PM	10.2	11.0	10.9	10.7
2022-05-18 4:00 PM	9.0	10.2	9.0	9.4
2022-05-18 5:00 PM	8.7	9.9	8.7	9.1
2022-05-18 6:00 PM	7.5	9.1	7.3	8.0
2022-05-18 7:00 PM	7.8	9.4	9.0	8.7
2022-05-18 8:00 PM	7.0	8.9	7.1	7.7
2022-05-18 9:00 PM	6.8	8.4	6.6	7.3
2022-05-18 10:00 PM	6.8	8.3	6.6	7.2
2022-05-18 11:00 PM	6.6	8.2	6.4	7.1
2022-05-19 12:00 AM	6.5	8.0	6.2	6.9
2022-05-19 1:00 AM	6.6	8.0	6.3	7.0
2022-05-19 2:00 AM	6.4	8.1	6.5	7.0
2022-05-19 3:00 AM	6.4	8.1	6.6	7.0
2022-05-19 4:00 AM	6.5	7.9	6.8	7.1
2022-05-19 5:00 AM	6.4	7.5	7.1	7.0
2022-05-19 6:00 AM	6.5	7.6	7.1	7.1
2022-05-19 7:00 AM	6.9	8.0	7.0	7.3
2022-05-19 8:00 AM	7.3	8.1	7.8	7.7
2022-05-19 9:00 AM	7.7	8.4	8.4	8.2
2022-05-19 10:00 AM	8.8	9.2	9.5	9.2
2022-05-19 11:00 AM	11.4	10.8	11.5	11.2
2022-05-19 12:00 PM	13.8	12.3	13.5	13.2
2022-05-19 1:00 PM	16.1	13.5	14.7	14.8
2022-05-19 2:00 PM	14.5	13.3	14.5	14.1
2022-05-19 3:00 PM	16.2	14.3	16.4	15.6
2022-05-19 4:00 PM	15.1	13.9	15.8	14.9
2022-05-19 5:00 PM	15.2	14.5	17.7	15.8
2022-05-19 6:00 PM	13.8	13.7	15.7	14.4
2022-05-19 7:00 PM	13.3	13.2	13.9	13.5
2022-05-19 8:00 PM	11.3	11.8	12.4	11.8
2022-05-19 9:00 PM	9.0	10.6	9.9	9.8
2022-05-19 10:00 PM	7.6	9.7	8.4	8.6
2022-05-19 11:00 PM	7.7	9.6	8.3	8.5
2022-05-20 12:00 AM	7.4	9.4	8.0	8.3
2022-05-20 1:00 AM	6.8	8.8	7.2	7.6
2022-05-20 2:00 AM	6.4	8.3	6.7	7.1
2022-05-20 3:00 AM	6.4	8.3	6.8	7.2
2022-05-20 4:00 AM	6.9	8.5	6.8	7.4

2022-05-20 5:00 AM	7.2	8.7	7.1	7.7
2022-05-20 6:00 AM	7.4	8.8	7.4	7.9
2022-05-20 7:00 AM	6.6	8.3	7.0	7.3
2022-05-20 8:00 AM	6.2	7.9	6.8	7.0
2022-05-20 9:00 AM	7.1	8.5	7.5	7.7
2022-05-20 10:00 AM	7.9	8.7	8.3	8.3
2022-05-20 11:00 AM	8.1	8.6	8.7	8.5
2022-05-20 12:00 PM	10.2	9.9	10.0	10.0
2022-05-20 1:00 PM	18.8	13.6	13.7	15.4
2022-05-20 2:00 PM	19.4	14.4	15.3	16.4
2022-05-20 3:00 PM	20.4	14.9	18.7	18.0
2022-05-20 4:00 PM	21.8	17.3	24.8	21.3
2022-05-20 5:00 PM	19.1	16.7	22.0	19.3
2022-05-20 6:00 PM	18.2	16.2	23.0	19.1
2022-05-20 7:00 PM	16.7	15.4	20.0	17.4
2022-05-20 8:00 PM	15.7	14.8	16.4	15.6
2022-05-20 9:00 PM	13.8	13.1	14.6	13.8
2022-05-20 10:00 PM	11.6	11.8	11.7	11.7
2022-05-20 11:00 PM	9.1	10.4	9.1	9.5
2022-05-21 12:00 AM	7.6	9.0	7.2	7.9
2022-05-21 1:00 AM	6.8	7.9	6.0	6.9
2022-05-21 2:00 AM	6.2	7.1	5.5	6.3
2022-05-21 3:00 AM	5.6	6.5	5.0	5.7
2022-05-21 4:00 AM	5.0	5.9	4.4	5.1
2022-05-21 5:00 AM	4.2	5.7	3.9	4.6
2022-05-21 6:00 AM	3.6	5.7	3.7	4.3
2022-05-21 7:00 AM	3.1	5.3	3.9	4.1
2022-05-21 8:00 AM	3.7	5.9	4.9	4.8
2022-05-21 9:00 AM	6.1	6.9	6.0	6.3
2022-05-21 10:00 AM	8.0	7.7	6.9	7.5
2022-05-21 11:00 AM	9.3	8.3	8.2	8.6
2022-05-21 12:00 PM	11.3	9.0	9.0	9.8
2022-05-21 1:00 PM	15.8	10.6	11.2	12.5
2022-05-21 2:00 PM	12.3	10.2	10.9	11.1
2022-05-21 3:00 PM	11.4	10.3	10.9	10.9
2022-05-21 4:00 PM	12.3	10.6	11.8	11.6
2022-05-21 5:00 PM	13.9	11.6	15.0	13.5
2022-05-21 6:00 PM	13.1	10.9	13.2	12.4
2022-05-21 7:00 PM	10.7	10.5	10.5	10.6
2022-05-21 8:00 PM	9.1	9.9	9.4	9.5
2022-05-21 9:00 PM	8.7	9.6	8.9	9.1
2022-05-21 10:00 PM	8.2	9.0	8.0	8.4
2022-05-21 11:00 PM	7.5	8.8	7.6	8.0

2022-05-22 12:00 AM	7.4	8.6	7.1	7.7
2022-05-22 1:00 AM	7.0	8.4	7.1	7.5
2022-05-22 2:00 AM	6.4	8.1	6.3	6.9
2022-05-22 3:00 AM	5.8	7.4	5.7	6.3
2022-05-22 4:00 AM	5.4	6.9	4.7	5.7
2022-05-22 5:00 AM	5.2	6.9	5.1	5.7
2022-05-22 6:00 AM	5.5	6.8	4.7	5.7
2022-05-22 7:00 AM	5.5	6.8	5.6	6.0
2022-05-22 8:00 AM	5.5	7.1	6.8	6.5
2022-05-22 9:00 AM	6.9	7.6	7.6	7.4
2022-05-22 10:00 AM	9.4	8.6	8.0	8.7
2022-05-22 11:00 AM	9.9	8.8	8.4	9.0
2022-05-22 12:00 PM	15.4	10.6	9.1	11.7
2022-05-22 1:00 PM	20.1	10.3	11.0	13.8
2022-05-22 2:00 PM	23.1	12.0	12.8	16.0
2022-05-22 3:00 PM	26.7	13.0	15.6	18.4
2022-05-22 4:00 PM	15.3	11.7	12.5	13.2
2022-05-22 5:00 PM	17.5	12.1	13.8	14.5
2022-05-22 6:00 PM	19.2	12.8	18.6	16.9
2022-05-22 7:00 PM	13.2	11.5	13.1	12.6
2022-05-22 8:00 PM	9.7	10.7	11.4	10.6
2022-05-22 9:00 PM	8.4	10.3	10.3	9.7
2022-05-22 10:00 PM	4.3	9.0	8.2	7.2
2022-05-22 11:00 PM	3.3	8.2	6.4	6.0
2022-05-23 12:00 AM	2.4	7.5	5.4	5.1
2022-05-23 1:00 AM	1.7	6.8	4.4	4.3
2022-05-23 2:00 AM	0.9	5.9	3.2	3.3
2022-05-23 3:00 AM	0.0	5.5	2.8	2.8
2022-05-23 4:00 AM	-0.6	5.3	2.0	2.2
2022-05-23 5:00 AM	-0.9	4.5	1.2	1.6
2022-05-23 6:00 AM	-0.8	4.5	1.5	1.7
2022-05-23 7:00 AM	-0.4	4.1	1.5	1.7
2022-05-23 8:00 AM	1.3	4.9	3.0	3.1
2022-05-23 9:00 AM	4.1	6.4	5.4	5.3
2022-05-23 10:00 AM	7.4	7.8	7.1	7.4
2022-05-23 11:00 AM	11.3	9.9	8.6	9.9
2022-05-23 12:00 PM	32.4	13.6	11.4	19.1
2022-05-23 1:00 PM	27.6	13.5	14.6	18.6
2022-05-23 2:00 PM	30.0	13.4	16.0	19.8
2022-05-23 3:00 PM	38.3	15.1	22.0	25.1
2022-05-23 4:00 PM	31.3	17.0	27.1	25.1
2022-05-23 5:00 PM	30.7	18.9	34.2	27.9
2022-05-23 6:00 PM	21.0	15.0	24.2	20.1

2022-05-23 7:00 PM	16.7	13.8	18.0	16.2
2022-05-23 8:00 PM	12.7	12.6	13.8	13.0
2022-05-23 9:00 PM	11.1	12.0	12.1	11.7
2022-05-23 10:00 PM	8.6	11.2	10.9	10.2
2022-05-23 11:00 PM	5.9	10.2	9.6	8.6
2022-05-24 12:00 AM	4.4	9.2	8.2	7.3
2022-05-24 1:00 AM	3.5	8.5	6.9	6.3
2022-05-24 2:00 AM	3.1	7.8	6.0	5.6
2022-05-24 3:00 AM	2.5	7.1	5.0	4.9
2022-05-24 4:00 AM	1.7	6.5	3.9	4.0
2022-05-24 5:00 AM	1.3	6.0	3.3	3.5
2022-05-24 6:00 AM	1.1	5.7	3.0	3.3
2022-05-24 7:00 AM	2.5	6.2	3.8	4.2
2022-05-24 8:00 AM	4.8	6.9	5.5	5.7
2022-05-24 9:00 AM	9.9	8.9	8.3	9.0
2022-05-24 10:00 AM	11.9	9.8	9.4	10.4
2022-05-24 11:00 AM	12.8	11.0	9.7	11.2
2022-05-24 12:00 PM	34.9	15.0	11.6	20.5
2022-05-24 1:00 PM	40.9	14.8	14.9	23.5
2022-05-24 2:00 PM	25.2	15.5	17.5	19.4
2022-05-24 3:00 PM	23.0	17.0	19.9	20.0
2022-05-24 4:00 PM	21.5	18.4	23.2	21.0
2022-05-24 5:00 PM	19.9	17.7	23.7	20.4
2022-05-24 6:00 PM	19.2	17.0	23.4	19.9
2022-05-24 7:00 PM	18.9	16.8	21.3	19.0
2022-05-24 8:00 PM	17.6	15.9	17.0	16.8
2022-05-24 9:00 PM	16.0	15.0	15.2	15.4
2022-05-24 10:00 PM	13.4	13.8	13.6	13.6
2022-05-24 11:00 PM	10.6	12.1	11.8	11.5
2022-05-25 12:00 AM	9.6	11.3	10.8	10.6
2022-05-25 1:00 AM	9.0	10.7	10.1	9.9
2022-05-25 2:00 AM	8.7	10.4	9.5	9.5
2022-05-25 3:00 AM	8.4	10.0	9.0	9.1
2022-05-25 4:00 AM	8.0	9.7	8.4	8.7
2022-05-25 5:00 AM	7.8	9.4	8.0	8.4
2022-05-25 6:00 AM	7.7	9.3	7.7	8.2
2022-05-25 7:00 AM	7.9	9.3	8.3	8.5
2022-05-25 8:00 AM	8.7	9.9	8.8	9.1
2022-05-25 9:00 AM	9.2	10.4	9.7	9.8
2022-05-25 10:00 AM	9.8	10.9	10.5	10.4
2022-05-25 11:00 AM	10.1	11.1	10.8	10.7
2022-05-25 12:00 PM	10.5	11.2	10.9	10.9
2022-05-25 1:00 PM	11.2	11.7	11.9	11.6

2022-05-25 2:00 PM	12.8	12.6	13.3	12.9
2022-05-25 3:00 PM	12.9	13.0	13.4	13.1
2022-05-25 4:00 PM	11.8	12.4	12.2	12.1
2022-05-25 5:00 PM	10.9	11.6	10.9	11.1
2022-05-25 6:00 PM	10.3	11.1	10.1	10.5
2022-05-25 7:00 PM	10.4	10.9	9.9	10.4
2022-05-25 8:00 PM	10.0	10.7	9.7	10.1
2022-05-25 9:00 PM	9.6	10.4	9.4	9.8
2022-05-25 10:00 PM	9.4	10.2	9.2	9.6
2022-05-25 11:00 PM	9.2	10.0	9.0	9.4
2022-05-26 12:00 AM	9.3	9.9	9.0	9.4
2022-05-26 1:00 AM	9.4	10.0	9.3	9.6
2022-05-26 2:00 AM	9.5	10.0	9.3	9.6
2022-05-26 3:00 AM	9.5	10.0	9.3	9.6
2022-05-26 4:00 AM	9.5	10.1	9.4	9.7
2022-05-26 5:00 AM	9.5	10.1	9.3	9.6
2022-05-26 6:00 AM	9.5	9.9	9.2	9.5
2022-05-26 7:00 AM	9.4	9.8	9.1	9.4
2022-05-26 8:00 AM	9.7	9.9	9.4	9.7
2022-05-26 9:00 AM	10.3	10.3	10.0	10.2
2022-05-26 10:00 AM	11.7	11.1	11.2	11.3
2022-05-26 11:00 AM	13.7	12.2	12.6	12.8
2022-05-26 12:00 PM	14.6	12.6	13.1	13.4
2022-05-26 1:00 PM	14.0	12.5	13.4	13.3
2022-05-26 2:00 PM	14.5	12.9	14.2	13.9
2022-05-26 3:00 PM	15.5	13.6	14.8	14.6
2022-05-26 4:00 PM	17.0	14.7	17.2	16.3
2022-05-26 5:00 PM	15.7	14.1	15.5	15.1
2022-05-26 6:00 PM	14.5	13.3	15.0	14.3
2022-05-26 7:00 PM	14.4	13.3	14.7	14.1
2022-05-26 8:00 PM	14.0	13.2	14.4	13.9
2022-05-26 9:00 PM	13.1	12.8	13.9	13.3
2022-05-26 10:00 PM	12.3	12.5	12.9	12.6
2022-05-26 11:00 PM	11.9	12.4	12.4	12.2
2022-05-27 12:00 AM	11.7	12.2	12.2	12.0
2022-05-27 1:00 AM	11.4	12.1	11.9	11.8
2022-05-27 2:00 AM	11.1	11.9	11.6	11.5
2022-05-27 3:00 AM	10.9	11.7	11.6	11.4
2022-05-27 4:00 AM	10.6	11.5	11.5	11.2
2022-05-27 5:00 AM	10.3	11.3	11.3	11.0
2022-05-27 6:00 AM	10.3	11.3	11.2	10.9
2022-05-27 7:00 AM	10.6	11.3	10.9	10.9
2022-05-27 8:00 AM	10.6	11.3	10.8	10.9

2022-05-27 9:00 AM	10.4	11.3	11.0	10.9
2022-05-27 10:00 AM	12.0	12.1	12.0	12.0
2022-05-27 11:00 AM	16.8	14.3	14.1	15.1
2022-05-27 12:00 PM	21.7	16.1	15.2	17.7
2022-05-27 1:00 PM	20.7	16.5	16.9	18.0
2022-05-27 2:00 PM	24.1	17.1	19.7	20.3
2022-05-27 3:00 PM	25.4	18.6	25.4	23.1
2022-05-27 4:00 PM	25.3	21.7	34.0	27.0
2022-05-27 5:00 PM	22.7	21.5	32.5	25.6
2022-05-27 6:00 PM	18.9	18.2	25.8	21.0
2022-05-27 7:00 PM	17.9	17.3	22.4	19.2
2022-05-27 8:00 PM	16.0	15.8	17.2	16.3
2022-05-27 9:00 PM	14.8	15.3	15.9	15.3
2022-05-27 10:00 PM	12.7	14.0	13.9	13.5
2022-05-27 11:00 PM	11.9	13.4	12.9	12.7
2022-05-28 12:00 AM	12.0	13.1	12.4	12.5
2022-05-28 1:00 AM	10.9	12.4	11.4	11.6
2022-05-28 2:00 AM	10.1	11.9	10.9	11.0
2022-05-28 3:00 AM	9.5	11.4	10.3	10.4
2022-05-28 4:00 AM	9.5	11.3	9.6	10.1
2022-05-28 5:00 AM	9.5	11.3	9.2	10.0
2022-05-28 6:00 AM	9.7	11.2	9.2	10.0
2022-05-28 7:00 AM	10.1	11.4	9.8	10.4
2022-05-28 8:00 AM	10.6	11.7	11.0	11.1
2022-05-28 9:00 AM	10.4	11.6	11.0	11.0
2022-05-28 10:00 AM	10.7	11.7	10.7	11.0
2022-05-28 11:00 AM	10.7	11.9	11.6	11.4
2022-05-28 12:00 PM	11.5	12.4	12.3	12.1
2022-05-28 1:00 PM	11.5	12.6	12.3	12.1
2022-05-28 2:00 PM	12.3	13.2	13.3	12.9
2022-05-28 3:00 PM	12.1	13.1	12.9	12.7
2022-05-28 4:00 PM	11.6	12.7	12.1	12.1
2022-05-28 5:00 PM	11.1	12.4	11.4	11.6
2022-05-28 6:00 PM	11.5	12.3	11.6	11.8
2022-05-28 7:00 PM	11.7	12.4	11.9	12.0
2022-05-28 8:00 PM	11.3	12.2	11.5	11.7
2022-05-28 9:00 PM	11.0	11.9	11.1	11.3
2022-05-28 10:00 PM	10.7	11.7	11.0	11.1
2022-05-28 11:00 PM	10.6	11.5	10.7	10.9
2022-05-29 12:00 AM	10.5	11.5	10.9	11.0
2022-05-29 1:00 AM	10.7	11.5	10.8	11.0
2022-05-29 2:00 AM	10.5	11.4	10.6	10.8
2022-05-29 3:00 AM	10.1	11.1	10.1	10.4

2022-05-29 4:00 AM	9.4	10.7	9.6	9.9
2022-05-29 5:00 AM	9.1	10.5	9.2	9.6
2022-05-29 6:00 AM	9.0	10.4	9.2	9.5
2022-05-29 7:00 AM	9.1	10.5	9.4	9.7
2022-05-29 8:00 AM	9.3	10.6	9.6	9.8
2022-05-29 9:00 AM	10.1	11.1	10.2	10.5
2022-05-29 10:00 AM	11.4	11.9	11.1	11.5
2022-05-29 11:00 AM	13.0	12.5	11.1	12.2
2022-05-29 12:00 PM	18.7	14.7	12.4	15.3
2022-05-29 1:00 PM	17.7	15.0	14.2	15.6
2022-05-29 2:00 PM	16.5	14.9	14.8	15.4
2022-05-29 3:00 PM	16.2	15.1	15.5	15.6
2022-05-29 4:00 PM	14.4	14.5	14.2	14.4
2022-05-29 5:00 PM	13.0	13.5	12.5	13.0
2022-05-29 6:00 PM	14.0	14.1	15.9	14.7
2022-05-29 7:00 PM	14.4	14.4	16.2	15.0
2022-05-29 8:00 PM	13.5	13.7	14.1	13.8
2022-05-29 9:00 PM	12.6	13.2	12.9	12.9
2022-05-29 10:00 PM	11.9	12.7	12.1	12.2
2022-05-29 11:00 PM	12.0	12.7	12.2	12.3
2022-05-30 12:00 AM	11.8	12.7	12.2	12.2
2022-05-30 1:00 AM	11.6	12.5	11.8	12.0
2022-05-30 2:00 AM	11.5	12.4	11.7	11.9
2022-05-30 3:00 AM	11.4	12.3	11.6	11.8
2022-05-30 4:00 AM	10.9	11.9	11.0	11.3
2022-05-30 5:00 AM	10.7	11.8	11.1	11.2
2022-05-30 6:00 AM	10.6	11.8	11.0	11.1
2022-05-30 7:00 AM	10.6	11.8	11.2	11.2
2022-05-30 8:00 AM	11.2	12.2	11.6	11.7
2022-05-30 9:00 AM	11.0	12.1	11.4	11.5
2022-05-30 10:00 AM	10.6	11.6	11.0	11.1
2022-05-30 11:00 AM	11.8	12.4	12.7	12.3
2022-05-30 12:00 PM	12.3	12.8	12.7	12.6
2022-05-30 1:00 PM	14.5	13.7	14.1	14.1
2022-05-30 2:00 PM	12.2	12.6	12.2	12.3
2022-05-30 3:00 PM	13.1	13.4	13.7	13.4
2022-05-30 4:00 PM	15.4	14.8	17.1	15.8
2022-05-30 5:00 PM	17.4	16.2	23.5	19.0
2022-05-30 6:00 PM	17.1	15.9	22.3	18.4
2022-05-30 7:00 PM	16.0	15.3	18.0	16.4
2022-05-30 8:00 PM	15.3	14.9	16.5	15.6
2022-05-30 9:00 PM	14.6	14.5	15.6	14.9
2022-05-30 10:00 PM	13.6	13.8	14.1	13.8

2022-05-30 11:00 PM	12.6	13.3	13.1	13.0
2022-05-31 12:00 AM	12.4	13.1	12.9	12.8
2022-05-31 1:00 AM	12.3	13.0	12.8	12.7
2022-05-31 2:00 AM	11.8	12.8	12.3	12.3
2022-05-31 3:00 AM	11.2	12.4	11.9	11.8
2022-05-31 4:00 AM	10.9	12.2	11.4	11.5
2022-05-31 5:00 AM	10.6	11.9	11.2	11.2
2022-05-31 6:00 AM	10.6	11.9	11.0	11.2
2022-05-31 7:00 AM	10.6	11.8	10.8	11.1
2022-05-31 8:00 AM	11.0	12.1	11.4	11.5
2022-05-31 9:00 AM	11.8	12.6	12.2	12.2
2022-05-31 10:00 AM	13.3	13.3	13.7	13.4
2022-05-31 11:00 AM	16.0	14.3	14.2	14.8
2022-05-31 12:00 PM	22.3	17.2	17.4	19.0
2022-05-31 1:00 PM	21.6	17.7	19.4	19.6
2022-05-31 2:00 PM	20.6	17.9	20.0	19.5
2022-05-31 3:00 PM	20.7	18.4	20.7	19.9
2022-05-31 4:00 PM	21.2	18.9	22.3	20.8
2022-05-31 5:00 PM	19.4	18.0	21.0	19.5
2022-05-31 6:00 PM	19.6	18.1	23.2	20.3
2022-05-31 7:00 PM	17.6	16.7	18.1	17.5
2022-05-31 8:00 PM	16.7	16.0	17.0	16.6
2022-05-31 9:00 PM	15.8	15.3	16.0	15.7
2022-05-31 10:00 PM	14.5	14.4	14.7	14.5
2022-05-31 11:00 PM	13.2	13.6	13.5	13.4
2022-06-01 12:00 AM	12.7	13.3	13.0	13.0
2022-06-01 1:00 AM	11.7	12.6	11.9	12.1
2022-06-01 2:00 AM	11.0	12.1	11.3	11.5
2022-06-01 3:00 AM	10.8	11.9	11.1	11.3
2022-06-01 4:00 AM	10.8	11.8	11.0	11.2
2022-06-01 5:00 AM	10.7	11.5	10.2	10.8
2022-06-01 6:00 AM	10.2	11.1	9.7	10.3
2022-06-01 7:00 AM	9.6	10.8	9.1	9.8
2022-06-01 8:00 AM	10.4	11.1	9.7	10.4
2022-06-01 9:00 AM	10.7	11.0	9.6	10.4
2022-06-01 10:00 AM	11.4	11.5	10.6	11.2
2022-06-01 11:00 AM	13.6	12.1	11.6	12.4
2022-06-01 12:00 PM	20.5	13.9	11.7	15.4
2022-06-01 1:00 PM	22.4	15.0	14.2	17.2
2022-06-01 2:00 PM	22.9	16.2	16.8	18.6
2022-06-01 3:00 PM	24.3	17.4	21.3	21.0
2022-06-01 4:00 PM	24.3	19.8	28.5	24.2
2022-06-01 5:00 PM	23.7	21.4	32.6	25.9

2022-06-01 6:00 PM	21.1	20.0	32.9	24.7
2022-06-01 7:00 PM	18.2	17.8	22.2	19.4
2022-06-01 8:00 PM	16.7	16.8	17.7	17.1
2022-06-01 9:00 PM	14.9	15.4	15.9	15.4
2022-06-01 10:00 PM	13.3	13.9	14.0	13.7
2022-06-01 11:00 PM	12.1	13.0	12.3	12.5
2022-06-02 12:00 AM	11.2	12.2	11.1	11.5
2022-06-02 1:00 AM	10.6	11.5	10.5	10.9
2022-06-02 2:00 AM	9.9	11.1	9.6	10.2
2022-06-02 3:00 AM	9.7	11.1	9.0	9.9
2022-06-02 4:00 AM	9.5	10.8	8.5	9.6
2022-06-02 5:00 AM	9.0	10.1	8.2	9.1
2022-06-02 6:00 AM	8.6	9.8	7.2	8.5
2022-06-02 7:00 AM	8.6	9.6	6.9	8.4
2022-06-02 8:00 AM	9.3	10.2	9.1	9.5
2022-06-02 9:00 AM	10.1	11.3	10.6	10.7
2022-06-02 10:00 AM	11.3	12.1	11.4	11.6
2022-06-02 11:00 AM	13.7	13.5	11.9	13.0
2022-06-02 12:00 PM	18.7	16.1	12.8	15.9
2022-06-02 1:00 PM	22.0	16.1	15.3	17.8
2022-06-02 2:00 PM	24.0	17.3	18.8	20.0
2022-06-02 3:00 PM	20.1	17.1	19.1	18.8
2022-06-02 4:00 PM	17.1	16.2	16.6	16.6
2022-06-02 5:00 PM	19.4	18.5	23.9	20.6
2022-06-02 6:00 PM	18.9	18.3	23.9	20.4
2022-06-02 7:00 PM	17.1	16.8	19.1	17.7
2022-06-02 8:00 PM	15.7	15.7	16.5	16.0
2022-06-02 9:00 PM	14.3	14.5	15.0	14.6
2022-06-02 10:00 PM	13.2	13.8	13.8	13.6
2022-06-02 11:00 PM	12.7	13.4	13.4	13.2
2022-06-03 12:00 AM	12.2	12.9	12.5	12.5
2022-06-03 1:00 AM	12.2	12.9	12.6	12.6
2022-06-03 2:00 AM	12.1	12.7	12.2	12.3
2022-06-03 3:00 AM	11.7	12.1	11.3	11.7
2022-06-03 4:00 AM	11.5	12.0	11.1	11.5
2022-06-03 5:00 AM	11.2	11.8	10.7	11.2
2022-06-03 6:00 AM	10.9	11.4	10.2	10.8
2022-06-03 7:00 AM	10.6	11.1	9.4	10.4
2022-06-03 8:00 AM	10.6	11.0	9.2	10.3
2022-06-03 9:00 AM	11.0	11.3	9.5	10.6
2022-06-03 10:00 AM	11.9	11.6	10.3	11.3
2022-06-03 11:00 AM	13.5	12.7	11.3	12.5
2022-06-03 12:00 PM	18.4	14.8	12.2	15.1

2022-06-03 1:00 PM	18.9	14.9	13.6	15.8
2022-06-03 2:00 PM	21.2	15.3	15.3	17.3
2022-06-03 3:00 PM	20.7	15.3	16.1	17.4
2022-06-03 4:00 PM	19.9	17.1	21.8	19.6
2022-06-03 5:00 PM	17.4	15.8	17.6	16.9
2022-06-03 6:00 PM	15.2	13.8	13.4	14.1
2022-06-03 7:00 PM	14.4	13.5	12.4	13.4
2022-06-03 8:00 PM	13.5	12.7	12.2	12.8
2022-06-03 9:00 PM	12.7	12.6	12.2	12.5
2022-06-03 10:00 PM	12.4	12.5	12.1	12.3
2022-06-03 11:00 PM	11.6	11.9	11.4	11.6
2022-06-04 12:00 AM	11.1	11.7	11.3	11.4
2022-06-04 1:00 AM	10.8	11.2	10.6	10.9
2022-06-04 2:00 AM	9.3	9.9	8.6	9.3
2022-06-04 3:00 AM	8.6	10.0	7.5	8.7
2022-06-04 4:00 AM	8.2	9.5	6.5	8.1
2022-06-04 5:00 AM	8.0	8.8	6.6	7.8
2022-06-04 6:00 AM	7.3	8.1	6.3	7.2
2022-06-04 7:00 AM	7.4	8.2	5.9	7.2
2022-06-04 8:00 AM	8.7	9.2	7.5	8.5
2022-06-04 9:00 AM	9.2	10.1	9.1	9.5
2022-06-04 10:00 AM	9.7	10.8	9.7	10.1
2022-06-04 11:00 AM	11.7	12.1	10.4	11.4
2022-06-04 12:00 PM	18.1	15.3	11.5	15.0
2022-06-04 1:00 PM	20.4	15.5	14.1	16.7
2022-06-04 2:00 PM	20.3	15.9	16.5	17.6
2022-06-04 3:00 PM	22.1	17.1	21.3	20.2
2022-06-04 4:00 PM	22.3	18.8	26.2	22.4
2022-06-04 5:00 PM	21.1	20.6	32.0	24.6
2022-06-04 6:00 PM	17.2	17.5	26.7	20.5
2022-06-04 7:00 PM	15.1	15.8	19.4	16.8
2022-06-04 8:00 PM	13.2	14.2	14.6	14.0
2022-06-04 9:00 PM	12.3	13.2	12.9	12.8
2022-06-04 10:00 PM	11.3	12.3	11.6	11.7
2022-06-04 11:00 PM	10.7	11.6	10.8	11.0
2022-06-05 12:00 AM	10.2	11.0	10.2	10.5
2022-06-05 1:00 AM	9.7	10.7	8.9	9.8
2022-06-05 2:00 AM	9.1	10.4	7.9	9.1
2022-06-05 3:00 AM	8.7	10.0	6.7	8.5
2022-06-05 4:00 AM	8.3	9.6	6.0	8.0
2022-06-05 5:00 AM	8.0	9.3	5.4	7.6
2022-06-05 6:00 AM	7.7	8.7	5.3	7.2
2022-06-05 7:00 AM	7.3	8.7	4.8	6.9

2022-06-05 8:00 AM	7.9	8.7	6.2	7.6
2022-06-05 9:00 AM	8.9	9.6	8.6	9.0
2022-06-05 10:00 AM	9.8	10.8	9.7	10.1
2022-06-05 11:00 AM	11.9	12.1	10.7	11.6
2022-06-05 12:00 PM	17.0	14.9	12.9	14.9
2022-06-05 1:00 PM	19.7	15.8	15.4	17.0
2022-06-05 2:00 PM	22.2	16.2	17.9	18.8
2022-06-05 3:00 PM	23.7	17.0	22.1	20.9
2022-06-05 4:00 PM	21.6	19.3	25.9	22.3
2022-06-05 5:00 PM	18.7	18.7	23.9	20.4
2022-06-05 6:00 PM	19.1	18.6	28.5	22.1
2022-06-05 7:00 PM	16.1	16.6	20.1	17.6
2022-06-05 8:00 PM	13.9	15.0	15.4	14.8
2022-06-05 9:00 PM	12.3	14.0	13.7	13.3
2022-06-05 10:00 PM	11.6	12.9	12.6	12.4
2022-06-05 11:00 PM	11.2	12.7	11.9	11.9
2022-06-06 12:00 AM	10.8	12.2	11.8	11.6
2022-06-06 1:00 AM	10.7	12.1	11.1	11.3
2022-06-06 2:00 AM	10.9	12.3	11.1	11.4
2022-06-06 3:00 AM	11.1	12.3	11.4	11.6
2022-06-06 4:00 AM	11.1	12.2	11.6	11.6
2022-06-06 5:00 AM	11.5	12.6	11.7	11.9
2022-06-06 6:00 AM	11.3	12.3	11.9	11.8
2022-06-06 7:00 AM	11.5	12.5	11.5	11.8
2022-06-06 8:00 AM	11.9	12.7	12.2	12.3
2022-06-06 9:00 AM	12.4	12.9	11.9	12.4
2022-06-06 10:00 AM	12.4	12.8	11.6	12.3
2022-06-06 11:00 AM	12.7	13.1	12.8	12.9
2022-06-06 12:00 PM	13.3	13.2	12.2	12.9
2022-06-06 1:00 PM	14.9	13.9	13.4	14.1
2022-06-06 2:00 PM	21.6	16.1	17.8	18.5
2022-06-06 3:00 PM	26.1	17.8	23.8	22.6
2022-06-06 4:00 PM	25.4	20.4	31.0	25.6
2022-06-06 5:00 PM	24.6	21.4	31.9	26.0
2022-06-06 6:00 PM	21.3	19.4	29.7	23.5
2022-06-06 7:00 PM	18.6	17.7	21.9	19.4
2022-06-06 8:00 PM	16.1	16.5	17.9	16.8
2022-06-06 9:00 PM	15.4	16.1	16.9	16.1
2022-06-06 10:00 PM	14.4	15.3	15.6	15.1
2022-06-06 11:00 PM	12.6	13.8	13.5	13.3
2022-06-07 12:00 AM	12.0	13.2	12.7	12.6
2022-06-07 1:00 AM	11.2	13.0	12.0	12.1
2022-06-07 2:00 AM	10.9	12.9	11.3	11.7

2022-06-07 3:00 AM	10.5	12.3	10.8	11.2
2022-06-07 4:00 AM	9.7	11.3	10.6	10.5
2022-06-07 5:00 AM	9.2	10.6	9.6	9.8
2022-06-07 6:00 AM	8.8	10.3	9.4	9.5
2022-06-07 7:00 AM	8.7	10.4	9.7	9.6
2022-06-07 8:00 AM	9.1	10.9	10.7	10.2
2022-06-07 9:00 AM	10.7	12.0	11.9	11.5
2022-06-07 10:00 AM	13.0	13.9	13.6	13.5
2022-06-07 11:00 AM	15.2	14.9	13.8	14.6
2022-06-07 12:00 PM	16.7	15.3	15.0	15.7
2022-06-07 1:00 PM	19.8	16.3	17.1	17.7
2022-06-07 2:00 PM	19.4	16.8	17.0	17.7
2022-06-07 3:00 PM	19.6	16.9	20.1	18.9
2022-06-07 4:00 PM	22.8	19.3	25.3	22.5
2022-06-07 5:00 PM	20.3	18.8	23.7	20.9
2022-06-07 6:00 PM	19.1	18.1	24.2	20.5
2022-06-07 7:00 PM	18.0	17.3	20.0	18.4
2022-06-07 8:00 PM	16.4	16.2	17.1	16.6
2022-06-07 9:00 PM	15.5	15.9	16.3	15.9
2022-06-07 10:00 PM	13.2	14.5	14.3	14.0
2022-06-07 11:00 PM	12.7	14.1	13.8	13.5
2022-06-08 12:00 AM	11.8	13.3	12.6	12.6
2022-06-08 1:00 AM	11.5	13.6	12.2	12.4
2022-06-08 2:00 AM	11.5	13.4	12.0	12.3
2022-06-08 3:00 AM	11.0	13.1	11.6	11.9
2022-06-08 4:00 AM	10.6	12.2	11.1	11.3
2022-06-08 5:00 AM	10.2	12.4	10.6	11.1
2022-06-08 6:00 AM	11.0	12.8	10.8	11.5
2022-06-08 7:00 AM	11.4	12.7	11.3	11.8
2022-06-08 8:00 AM	11.9	13.0	12.6	12.5
2022-06-08 9:00 AM	12.8	13.9	13.5	13.4
2022-06-08 10:00 AM	13.7	14.1	14.1	14.0
2022-06-08 11:00 AM	16.1	15.1	14.7	15.3
2022-06-08 12:00 PM	23.8	18.1	16.1	19.3
2022-06-08 1:00 PM	22.9	17.6	18.3	19.6
2022-06-08 2:00 PM	21.1	17.1	17.8	18.7
2022-06-08 3:00 PM	15.1	15.7	15.2	15.3
2022-06-08 4:00 PM	15.4	15.7	15.4	15.5
2022-06-08 5:00 PM	16.8	16.4	17.1	16.8
2022-06-08 6:00 PM	18.3	17.6	21.0	19.0
2022-06-08 7:00 PM	17.4	16.9	19.5	17.9
2022-06-08 8:00 PM	16.8	16.6	17.4	16.9
2022-06-08 9:00 PM	15.6	16.0	16.3	16.0

2022-06-08 10:00 PM	14.4	15.3	14.8	14.8
2022-06-08 11:00 PM	13.3	14.7	14.1	14.0
2022-06-09 12:00 AM	13.3	14.4	13.3	13.7
2022-06-09 1:00 AM	13.0	14.1	13.4	13.5
2022-06-09 2:00 AM	12.6	13.8	12.8	13.1
2022-06-09 3:00 AM	11.6	13.2	12.2	12.3
2022-06-09 4:00 AM	11.2	12.7	11.9	11.9
2022-06-09 5:00 AM	11.1	12.5	12.1	11.9
2022-06-09 6:00 AM	11.2	12.5	12.1	11.9
2022-06-09 7:00 AM	11.5	12.8	12.4	12.2
2022-06-09 8:00 AM	12.5	13.5	13.1	13.0
2022-06-09 9:00 AM	13.7	14.1	13.8	13.9
2022-06-09 10:00 AM	13.3	13.7	13.8	13.6
2022-06-09 11:00 AM	15.2	14.1	15.1	14.8
2022-06-09 12:00 PM	20.9	15.3	17.3	17.8
2022-06-09 1:00 PM	25.0	16.8	19.9	20.6
2022-06-09 2:00 PM	21.5	16.3	17.3	18.4
2022-06-09 3:00 PM	22.5	17.4	19.8	19.9
2022-06-09 4:00 PM	25.0	19.0	25.6	23.2
2022-06-09 5:00 PM	21.3	17.8	23.0	20.7
2022-06-09 6:00 PM	19.8	17.8	26.2	21.3
2022-06-09 7:00 PM	18.4	17.1	22.1	19.2
2022-06-09 8:00 PM	16.8	16.4	18.5	17.2
2022-06-09 9:00 PM	16.3	16.4	17.9	16.9
2022-06-09 10:00 PM	15.4	16.0	16.2	15.9
2022-06-09 11:00 PM	13.6	14.9	14.6	14.4
2022-06-10 12:00 AM	13.0	14.5	13.9	13.8
2022-06-10 1:00 AM	13.0	14.2	13.7	13.6
2022-06-10 2:00 AM	12.1	13.4	12.7	12.7
2022-06-10 3:00 AM	12.2	13.4	12.8	12.8
2022-06-10 4:00 AM	12.0	13.2	12.6	12.6
2022-06-10 5:00 AM	11.0	12.4	11.4	11.6
2022-06-10 6:00 AM	10.4	12.0	10.9	11.1
2022-06-10 7:00 AM	10.4	11.9	11.1	11.1
2022-06-10 8:00 AM	10.4	12.0	11.4	11.3
2022-06-10 9:00 AM	11.6	12.8	12.5	12.3
2022-06-10 10:00 AM	13.1	13.7	13.3	13.4
2022-06-10 11:00 AM	16.3	15.4	15.4	15.7
2022-06-10 12:00 PM	22.1	17.0	18.3	19.1
2022-06-10 1:00 PM	25.7	17.9	20.3	21.3
2022-06-10 2:00 PM	27.5	18.7	21.7	22.6
2022-06-10 3:00 PM	29.5	19.7	25.3	24.8
2022-06-10 4:00 PM	30.0	22.1	33.7	28.6

2022-06-10 5:00 PM	27.7	23.4	34.9	28.7
2022-06-10 6:00 PM	23.7	21.2	34.2	26.4
2022-06-10 7:00 PM	20.1	19.0	24.4	21.2
2022-06-10 8:00 PM	17.8	17.8	21.4	19.0
2022-06-10 9:00 PM	17.0	17.4	20.2	18.2
2022-06-10 10:00 PM	15.9	16.8	17.7	16.8
2022-06-10 11:00 PM	13.8	15.0	14.5	14.4
2022-06-11 12:00 AM	12.8	14.2	13.4	13.5
2022-06-11 1:00 AM	12.1	13.6	12.7	12.8
2022-06-11 2:00 AM	11.9	13.4	12.5	12.6
2022-06-11 3:00 AM	11.6	13.4	12.4	12.5
2022-06-11 4:00 AM	11.2	12.7	11.7	11.9
2022-06-11 5:00 AM	10.7	12.5	10.7	11.3
2022-06-11 6:00 AM	10.4	12.2	10.7	11.1
2022-06-11 7:00 AM	10.7	12.7	10.7	11.4
2022-06-11 8:00 AM	11.4	12.7	11.2	11.8
2022-06-11 9:00 AM	12.2	13.1	12.0	12.4
2022-06-11 10:00 AM	14.1	14.2	13.7	14.0
2022-06-11 11:00 AM	15.5	15.4	15.2	15.4
2022-06-11 12:00 PM	16.7	16.1	15.8	16.2
2022-06-11 1:00 PM	24.1	17.6	17.9	19.9
2022-06-11 2:00 PM	25.9	18.4	20.2	21.5
2022-06-11 3:00 PM	26.4	19.5	25.1	23.7
2022-06-11 4:00 PM	26.7	22.0	34.1	27.6
2022-06-11 5:00 PM	23.2	23.0	34.6	26.9
2022-06-11 6:00 PM	19.7	20.5	33.1	24.4
2022-06-11 7:00 PM	17.2	18.3	22.6	19.4
2022-06-11 8:00 PM	15.0	16.6	17.5	16.4
2022-06-11 9:00 PM	14.3	15.8	16.3	15.5
2022-06-11 10:00 PM	13.5	15.1	15.1	14.6
2022-06-11 11:00 PM	12.5	14.2	13.6	13.4
2022-06-12 12:00 AM	11.8	13.9	12.6	12.8
2022-06-12 1:00 AM	11.2	13.0	12.0	12.1
2022-06-12 2:00 AM	10.6	12.6	11.0	11.4
2022-06-12 3:00 AM	10.2	12.5	10.2	11.0
2022-06-12 4:00 AM	9.7	11.9	9.6	10.4
2022-06-12 5:00 AM	9.4	11.7	9.0	10.0
2022-06-12 6:00 AM	8.9	11.3	8.5	9.6
2022-06-12 7:00 AM	8.9	11.0	8.3	9.4
2022-06-12 8:00 AM	9.3	11.1	9.5	10.0
2022-06-12 9:00 AM	10.5	11.9	11.4	11.3
2022-06-12 10:00 AM	11.9	13.0	12.6	12.5
2022-06-12 11:00 AM	14.9	14.3	13.4	14.2

2022-06-12 12:00 PM	21.5	16.5	14.9	17.6
2022-06-12 1:00 PM	23.1	16.8	16.7	18.9
2022-06-12 2:00 PM	22.1	17.3	18.1	19.2
2022-06-12 3:00 PM	21.1	17.9	21.0	20.0
2022-06-12 4:00 PM	21.7	19.2	26.5	22.5
2022-06-12 5:00 PM	17.9	17.7	20.8	18.8
2022-06-12 6:00 PM	17.4	17.8	21.5	18.9
2022-06-12 7:00 PM	16.6	17.2	18.4	17.4
2022-06-12 8:00 PM	15.6	16.4	17.1	16.4
2022-06-12 9:00 PM	15.0	16.0	16.3	15.8
2022-06-12 10:00 PM	13.7	15.0	14.7	14.5
2022-06-12 11:00 PM	13.1	14.4	14.0	13.8
2022-06-13 12:00 AM	12.9	14.2	13.3	13.5
2022-06-13 1:00 AM	13.0	14.3	13.8	13.7
2022-06-13 2:00 AM	12.8	14.4	13.7	13.6
2022-06-13 3:00 AM	12.7	14.2	13.5	13.5
2022-06-13 4:00 AM	12.9	14.2	12.8	13.3
2022-06-13 5:00 AM	12.8	14.0	13.1	13.3
2022-06-13 6:00 AM	12.3	13.6	12.6	12.8
2022-06-13 7:00 AM	11.8	13.3	12.0	12.4
2022-06-13 8:00 AM	11.6	13.4	11.7	12.2
2022-06-13 9:00 AM	11.9	13.6	11.8	12.4
2022-06-13 10:00 AM	12.5	14.1	12.3	13.0
2022-06-13 11:00 AM	12.6	14.0	12.3	13.0
2022-06-13 12:00 PM	12.8	14.2	12.7	13.2
2022-06-13 1:00 PM	13.3	14.6	13.6	13.8
2022-06-13 2:00 PM	13.5	14.7	13.6	13.9
2022-06-13 3:00 PM	12.9	14.3	13.0	13.4
2022-06-13 4:00 PM	12.6	14.2	12.7	13.2
2022-06-13 5:00 PM	12.4	13.9	12.3	12.9
2022-06-13 6:00 PM	12.4	13.7	11.8	12.6
2022-06-13 7:00 PM	12.2	13.6	12.0	12.6
2022-06-13 8:00 PM	12.3	13.5	11.7	12.5
2022-06-13 9:00 PM	12.7	13.5	11.9	12.7
2022-06-13 10:00 PM	12.6	13.5	11.9	12.7
2022-06-13 11:00 PM	12.2	13.2	11.7	12.4
2022-06-14 12:00 AM	12.2	13.1	11.9	12.4
2022-06-14 1:00 AM	12.1	13.1	11.9	12.4
2022-06-14 2:00 AM	12.1	13.1	12.0	12.4
2022-06-14 3:00 AM	12.0	13.0	11.9	12.3
2022-06-14 4:00 AM	12.0	12.9	11.7	12.2
2022-06-14 5:00 AM	12.0	12.8	11.8	12.2
2022-06-14 6:00 AM	12.0	12.8	11.8	12.2

2022-06-14 7:00 AM	12.1	12.9	11.9	12.3
2022-06-14 8:00 AM	12.3	13.0	12.0	12.4
2022-06-14 9:00 AM	12.3	12.9	12.0	12.4
2022-06-14 10:00 AM	12.4	13.0	12.3	12.6
2022-06-14 11:00 AM	12.6	13.0	12.5	12.7
2022-06-14 12:00 PM	12.4	12.9	12.2	12.5
2022-06-14 1:00 PM	12.6	13.1	12.4	12.7
2022-06-14 2:00 PM	12.8	13.4	12.6	12.9
2022-06-14 3:00 PM	13.4	13.7	13.2	13.4
2022-06-14 4:00 PM	14.2	14.1	14.1	14.1
2022-06-14 5:00 PM	14.5	14.4	14.5	14.5
2022-06-14 6:00 PM	14.6	14.4	14.7	14.6
2022-06-14 7:00 PM	14.8	14.6	14.9	14.8
2022-06-14 8:00 PM	14.5	14.4	14.5	14.5
2022-06-14 9:00 PM	13.7	14.0	13.7	13.8
2022-06-14 10:00 PM	13.3	13.7	13.3	13.4
2022-06-14 11:00 PM	13.2	13.7	13.4	13.4
2022-06-15 12:00 AM	13.3	13.8	13.6	13.6
2022-06-15 1:00 AM	13.4	13.9	13.8	13.7
2022-06-15 2:00 AM	13.3	13.9	13.6	13.6
2022-06-15 3:00 AM	13.0	13.7	13.3	13.3
2022-06-15 4:00 AM	12.9	13.6	13.3	13.3
2022-06-15 5:00 AM	12.9	13.5	13.3	13.2
2022-06-15 6:00 AM	12.8	13.5	13.1	13.1
2022-06-15 7:00 AM	12.7	13.5	13.3	13.2
2022-06-15 8:00 AM	13.0	13.7	13.3	13.3
2022-06-15 9:00 AM	13.5	14.1	14.1	13.9
2022-06-15 10:00 AM	15.0	15.0	15.1	15.0
2022-06-15 11:00 AM	16.9	16.3	16.5	16.6
2022-06-15 12:00 PM	18.2	17.0	17.7	17.6
2022-06-15 1:00 PM	20.4	17.9	19.2	19.2
2022-06-15 2:00 PM	18.0	17.1	17.6	17.6
2022-06-15 3:00 PM	20.0	18.0	20.4	19.5
2022-06-15 4:00 PM	22.1	19.1	23.7	21.6
2022-06-15 5:00 PM	19.8	18.9	22.1	20.3
2022-06-15 6:00 PM	18.4	18.1	19.1	18.5
2022-06-15 7:00 PM	17.8	17.6	18.4	17.9
2022-06-15 8:00 PM	16.5	17.0	17.2	16.9
2022-06-15 9:00 PM	16.0	16.8	16.6	16.5
2022-06-15 10:00 PM	14.5	15.8	14.5	14.9
2022-06-15 11:00 PM	13.8	15.1	13.7	14.2
2022-06-16 12:00 AM	14.0	15.1	12.7	13.9
2022-06-16 1:00 AM	13.7	14.7	13.9	14.1

2022-06-16 2:00 AM	13.4	14.7	14.3	14.1
2022-06-16 3:00 AM	13.3	14.6	13.1	13.7
2022-06-16 4:00 AM	12.9	14.1	12.4	13.1
2022-06-16 5:00 AM	12.5	13.6	12.1	12.7
2022-06-16 6:00 AM	12.2	13.4	11.4	12.3
2022-06-16 7:00 AM	12.3	13.4	11.1	12.3
2022-06-16 8:00 AM	12.5	13.7	11.6	12.6
2022-06-16 9:00 AM	13.1	14.1	12.2	13.1
2022-06-16 10:00 AM	14.1	14.7	13.4	14.1
2022-06-16 11:00 AM	14.8	15.4	15.1	15.1
2022-06-16 12:00 PM	19.6	17.6	16.1	17.8
2022-06-16 1:00 PM	20.4	17.9	17.6	18.6
2022-06-16 2:00 PM	20.8	17.9	18.4	19.0
2022-06-16 3:00 PM	20.6	17.8	18.7	19.0
2022-06-16 4:00 PM	20.0	17.9	20.7	19.5
2022-06-16 5:00 PM	19.8	18.1	20.9	19.6
2022-06-16 6:00 PM	19.3	18.1	24.1	20.5
2022-06-16 7:00 PM	18.4	17.5	20.0	18.6
2022-06-16 8:00 PM	17.2	17.0	18.5	17.6
2022-06-16 9:00 PM	16.6	16.7	17.8	17.0
2022-06-16 10:00 PM	16.0	16.4	17.1	16.5
2022-06-16 11:00 PM	15.5	16.1	16.7	16.1
2022-06-17 12:00 AM	15.1	15.9	16.3	15.8
2022-06-17 1:00 AM	14.9	15.8	16.1	15.6
2022-06-17 2:00 AM	14.6	15.6	15.7	15.3
2022-06-17 3:00 AM	14.2	15.4	15.4	15.0
2022-06-17 4:00 AM	14.0	15.2	15.1	14.8
2022-06-17 5:00 AM	14.0	15.1	15.1	14.7
2022-06-17 6:00 AM	13.9	15.0	15.0	14.6
2022-06-17 7:00 AM	14.1	15.0	15.1	14.7
2022-06-17 8:00 AM	14.4	14.9	15.4	14.9
2022-06-17 9:00 AM	14.2	14.7	15.5	14.8
2022-06-17 10:00 AM	14.2	14.5	15.6	14.8
2022-06-17 11:00 AM	15.3	14.5	15.9	15.2
2022-06-17 12:00 PM	21.2	16.4	17.4	18.3
2022-06-17 1:00 PM	24.8	17.3	19.6	20.6
2022-06-17 2:00 PM	26.8	17.9	21.3	22.0
2022-06-17 3:00 PM	27.4	18.8	23.8	23.3
2022-06-17 4:00 PM	25.9	20.3	28.6	24.9
2022-06-17 5:00 PM	23.7	20.6	30.3	24.9
2022-06-17 6:00 PM	21.6	19.1	30.7	23.8
2022-06-17 7:00 PM	18.9	17.7	22.9	19.8
2022-06-17 8:00 PM	17.1	16.9	19.8	17.9

2022-06-17 9:00 PM	16.1	16.8	18.6	17.2
2022-06-17 10:00 PM	15.0	16.0	16.4	15.8
2022-06-17 11:00 PM	13.6	15.2	14.8	14.5
2022-06-18 12:00 AM	12.9	14.8	14.1	13.9
2022-06-18 1:00 AM	12.2	14.2	13.2	13.2
2022-06-18 2:00 AM	11.8	14.0	12.2	12.7
2022-06-18 3:00 AM	11.4	13.4	11.7	12.2
2022-06-18 4:00 AM	10.6	12.7	10.9	11.4
2022-06-18 5:00 AM	10.1	12.4	10.0	10.8
2022-06-18 6:00 AM	9.3	11.5	9.4	10.1
2022-06-18 7:00 AM	9.3	11.6	9.0	10.0
2022-06-18 8:00 AM	10.0	11.7	10.4	10.7
2022-06-18 9:00 AM	11.4	12.8	12.5	12.2
2022-06-18 10:00 AM	12.7	13.9	13.4	13.3
2022-06-18 11:00 AM	14.3	14.6	14.0	14.3
2022-06-18 12:00 PM	18.1	16.5	14.8	16.5
2022-06-18 1:00 PM	20.1	17.1	16.7	18.0
2022-06-18 2:00 PM	23.7	17.8	19.2	20.2
2022-06-18 3:00 PM	22.7	18.4	21.3	20.8
2022-06-18 4:00 PM	21.5	19.1	25.2	21.9
2022-06-18 5:00 PM	20.2	19.8	27.4	22.5
2022-06-18 6:00 PM	18.0	18.5	26.6	21.0
2022-06-18 7:00 PM	16.3	17.3	20.1	17.9
2022-06-18 8:00 PM	15.3	16.5	17.6	16.5
2022-06-18 9:00 PM	14.5	15.6	16.1	15.4
2022-06-18 10:00 PM	13.4	14.7	14.7	14.3
2022-06-18 11:00 PM	12.5	13.9	13.5	13.3
2022-06-19 12:00 AM	11.8	13.5	12.4	12.6
2022-06-19 1:00 AM	11.9	13.7	12.2	12.6
2022-06-19 2:00 AM	12.0	13.9	11.8	12.6
2022-06-19 3:00 AM	12.4	13.9	13.1	13.1
2022-06-19 4:00 AM	11.9	13.4	12.3	12.5
2022-06-19 5:00 AM	11.6	13.0	12.3	12.3
2022-06-19 6:00 AM	11.7	13.1	12.7	12.5
2022-06-19 7:00 AM	11.3	12.8	12.4	12.2
2022-06-19 8:00 AM	11.6	13.1	12.3	12.3
2022-06-19 9:00 AM	11.9	13.2	12.4	12.5
2022-06-19 10:00 AM	10.4	11.4	10.4	10.7
2022-06-19 11:00 AM	9.5	10.9	10.0	10.1
2022-06-19 12:00 PM	10.2	11.6	10.7	10.8
2022-06-19 1:00 PM	11.8	12.6	12.6	12.3
2022-06-19 2:00 PM	14.2	13.9	14.4	14.2
2022-06-19 3:00 PM	18.4	15.5	17.6	17.2

2022-06-19 4:00 PM	15.9	15.4	16.9	16.1
2022-06-19 5:00 PM	15.5	15.9	18.3	16.6
2022-06-19 6:00 PM	15.1	15.2	15.9	15.4
2022-06-19 7:00 PM	14.6	15.0	15.5	15.0
2022-06-19 8:00 PM	14.2	14.8	15.1	14.7
2022-06-19 9:00 PM	13.3	14.3	14.1	13.9
2022-06-19 10:00 PM	12.4	13.6	12.9	13.0
2022-06-19 11:00 PM	12.2	13.4	12.5	12.7
2022-06-20 12:00 AM	12.0	13.2	12.5	12.6
2022-06-20 1:00 AM	12.1	13.2	12.7	12.7
2022-06-20 2:00 AM	12.0	13.2	12.6	12.6
2022-06-20 3:00 AM	12.0	13.2	12.5	12.6
2022-06-20 4:00 AM	11.9	13.0	12.6	12.5
2022-06-20 5:00 AM	11.7	12.7	12.6	12.3
2022-06-20 6:00 AM	11.5	12.4	12.2	12.0
2022-06-20 7:00 AM	11.8	12.6	12.5	12.3
2022-06-20 8:00 AM	12.2	13.3	13.2	12.9
2022-06-20 9:00 AM	12.3	13.3	12.9	12.8
2022-06-20 10:00 AM	12.8	13.6	13.2	13.2
2022-06-20 11:00 AM	14.2	14.2	13.5	14.0
2022-06-20 12:00 PM	20.0	16.8	14.6	17.1
2022-06-20 1:00 PM	23.4	17.6	17.4	19.5
2022-06-20 2:00 PM	24.9	18.3	19.8	21.0
2022-06-20 3:00 PM	25.5	18.9	22.7	22.4
2022-06-20 4:00 PM	24.9	20.3	26.8	24.0
2022-06-20 5:00 PM	22.5	21.1	28.9	24.2
2022-06-20 6:00 PM	21.0	20.0	29.8	23.6
2022-06-20 7:00 PM	19.7	19.1	23.9	20.9
2022-06-20 8:00 PM	18.6	18.5	20.3	19.1
2022-06-20 9:00 PM	18.3	18.4	20.0	18.9
2022-06-20 10:00 PM	17.9	18.1	19.4	18.5
2022-06-20 11:00 PM	17.0	17.6	18.3	17.6
2022-06-21 12:00 AM	16.5	17.3	17.7	17.2
2022-06-21 1:00 AM	16.3	17.1	17.5	17.0
2022-06-21 2:00 AM	16.3	17.0	17.5	16.9
2022-06-21 3:00 AM	17.4	17.8	19.3	18.2
2022-06-21 4:00 AM	17.7	17.8	18.8	18.1
2022-06-21 5:00 AM	17.5	17.7	18.6	17.9
2022-06-21 6:00 AM	17.5	17.7	18.4	17.9
2022-06-21 7:00 AM	17.4	17.5	18.8	17.9
2022-06-21 8:00 AM	17.4	17.6	18.4	17.8
2022-06-21 9:00 AM	17.3	17.4	16.9	17.2
2022-06-21 10:00 AM	17.5	17.8	18.0	17.8

2022-06-21 11:00 AM	18.2	18.3	18.8	18.4
2022-06-21 12:00 PM	21.6	20.3	20.2	20.7
2022-06-21 1:00 PM	25.2	21.9	22.5	23.2
2022-06-21 2:00 PM	26.9	22.6	24.0	24.5
2022-06-21 3:00 PM	28.6	22.9	26.2	25.9
2022-06-21 4:00 PM	28.0	24.0	28.2	26.7
2022-06-21 5:00 PM	27.8	24.2	27.9	26.6
2022-06-21 6:00 PM	26.1	23.0	29.7	26.3
2022-06-21 7:00 PM	23.8	21.7	24.7	23.4
2022-06-21 8:00 PM	21.7	20.9	22.1	21.6
2022-06-21 9:00 PM	20.9	20.6	21.4	21.0
2022-06-21 10:00 PM	20.0	20.3	21.0	20.4
2022-06-21 11:00 PM	19.0	19.9	20.4	19.8
2022-06-22 12:00 AM	18.3	19.5	19.9	19.2
2022-06-22 1:00 AM	18.0	19.1	19.5	18.9
2022-06-22 2:00 AM	17.6	18.8	19.1	18.5
2022-06-22 3:00 AM	17.4	18.6	18.8	18.3
2022-06-22 4:00 AM	17.1	18.4	18.1	17.9
2022-06-22 5:00 AM	16.2	17.8	17.0	17.0
2022-06-22 6:00 AM	15.7	17.4	16.6	16.6
2022-06-22 7:00 AM	15.5	17.2	16.5	16.4
2022-06-22 8:00 AM	16.1	17.4	16.4	16.6
2022-06-22 9:00 AM	16.2	17.0	15.6	16.3
2022-06-22 10:00 AM	15.9	16.7	15.8	16.1
2022-06-22 11:00 AM	18.0	17.3	16.6	17.3
2022-06-22 12:00 PM	24.4	18.8	18.3	20.5
2022-06-22 1:00 PM	27.0	19.4	20.7	22.4
2022-06-22 2:00 PM	28.8	20.5	22.3	23.9
2022-06-22 3:00 PM	28.1	20.9	23.6	24.2
2022-06-22 4:00 PM	26.9	21.5	25.0	24.5

Table S4: Hourly air temperature under 30% shade from May 10 to September 23, 2023.

	Port 1	Port 2	Port 3	Average
	5TE Moisture/ Temp/EC	5TE Moisture/ Temp/EC	5TE Moisture/ Temp/EC	5TE Moisture/ Temp/EC
**Measurement Time**	**°C Temp**	**°C Temp**	**°C Temp**	**°C Temp**
2022-05-11 2:00 PM	18.4	20.1	21.2	19.9
2022-05-11 3:00 PM	18.4	21.1	21.0	20.2
2022-05-11 4:00 PM	18.6	24.9	21.0	21.5

2022-05-11 5:00 PM	18.5	21.5	20.2	20.1
2022-05-11 6:00 PM	17.1	17.8	18.2	17.7
2022-05-11 7:00 PM	15.7	16.2	16.6	16.2
2022-05-11 8:00 PM	15.0	15.8	15.6	15.5
2022-05-11 9:00 PM	14.0	14.7	14.8	14.5
2022-05-11 10:00 PM	12.5	12.7	13.1	12.8
2022-05-11 11:00 PM	11.8	12.0	12.0	11.9
2022-05-12 12:00 AM	11.4	11.6	11.6	11.5
2022-05-12 1:00 AM	10.8	10.9	10.6	10.8
2022-05-12 2:00 AM	11.6	11.9	11.7	11.7
2022-05-12 3:00 AM	12.4	12.7	13.2	12.8
2022-05-12 4:00 AM	9.8	9.8	9.6	9.7
2022-05-12 5:00 AM	10.0	10.2	9.7	10.0
2022-05-12 6:00 AM	10.5	10.7	10.3	10.5
2022-05-12 7:00 AM	10.4	10.5	10.2	10.4
2022-05-12 8:00 AM	9.9	10.0	9.7	9.9
2022-05-12 9:00 AM	10.4	10.6	10.2	10.4
2022-05-12 10:00 AM	10.8	10.9	10.5	10.7
2022-05-12 11:00 AM	11.4	11.5	11.0	11.3
2022-05-12 12:00 PM	13.6	13.4	12.5	13.2
2022-05-12 1:00 PM	15.1	15.2	14.8	15.0
2022-05-12 2:00 PM	17.0	17.3	17.4	17.2
2022-05-12 3:00 PM	17.3	18.5	18.7	18.2
2022-05-12 4:00 PM	16.1	16.7	16.3	16.4
2022-05-12 5:00 PM	14.9	15.6	15.3	15.3
2022-05-12 6:00 PM	15.0	15.9	15.2	15.4
2022-05-12 7:00 PM	14.4	15.2	14.6	14.7
2022-05-12 8:00 PM	13.4	14.1	13.8	13.8
2022-05-12 9:00 PM	11.7	11.7	11.5	11.6
2022-05-12 10:00 PM	10.9	11.0	10.7	10.9
2022-05-12 11:00 PM	11.0	11.3	11.0	11.1
2022-05-13 12:00 AM	11.1	11.3	11.1	11.2
2022-05-13 1:00 AM	10.7	10.8	10.5	10.7
2022-05-13 2:00 AM	10.2	10.4	9.9	10.2
2022-05-13 3:00 AM	11.1	11.3	11.0	11.1
2022-05-13 4:00 AM	10.3	10.4	10.0	10.2
2022-05-13 5:00 AM	10.1	10.2	9.9	10.1
2022-05-13 6:00 AM	10.1	10.3	10.0	10.1
2022-05-13 7:00 AM	9.6	9.7	9.3	9.5
2022-05-13 8:00 AM	9.7	9.7	9.4	9.6
2022-05-13 9:00 AM	10.2	10.2	9.8	10.1
2022-05-13 10:00 AM	11.0	10.8	10.3	10.7
2022-05-13 11:00 AM	13.2	12.1	11.6	12.3

2022-05-13 12:00 PM	16.0	13.5	14.9	14.8
2022-05-13 1:00 PM	14.8	14.5	16.7	15.3
2022-05-13 2:00 PM	14.9	15.6	16.9	15.8
2022-05-13 3:00 PM	15.2	17.2	17.6	16.7
2022-05-13 4:00 PM	15.9	25.0	18.5	19.8
2022-05-13 5:00 PM	15.7	24.6	18.0	19.4
2022-05-13 6:00 PM	15.2	26.5	18.0	19.9
2022-05-13 7:00 PM	14.1	19.8	15.8	16.6
2022-05-13 8:00 PM	13.1	14.6	13.9	13.9
2022-05-13 9:00 PM	12.5	13.4	13.3	13.1
2022-05-13 10:00 PM	10.8	11.3	11.1	11.1
2022-05-13 11:00 PM	9.4	9.8	9.2	9.5
2022-05-14 12:00 AM	8.9	9.2	8.8	9.0
2022-05-14 1:00 AM	9.3	9.5	9.1	9.3
2022-05-14 2:00 AM	8.9	9.3	8.7	9.0
2022-05-14 3:00 AM	8.9	9.2	8.6	8.9
2022-05-14 4:00 AM	8.8	9.2	8.5	8.8
2022-05-14 5:00 AM	8.5	8.8	8.2	8.5
2022-05-14 6:00 AM	7.9	8.2	7.5	7.9
2022-05-14 7:00 AM	7.2	7.4	6.7	7.1
2022-05-14 8:00 AM	8.5	8.6	7.6	8.2
2022-05-14 9:00 AM	9.6	9.5	8.8	9.3
2022-05-14 10:00 AM	11.7	11.7	11.7	11.7
2022-05-14 11:00 AM	15.9	14.7	16.2	15.6
2022-05-14 12:00 PM	18.2	14.8	17.5	16.8
2022-05-14 1:00 PM	15.5	15.5	18.8	16.6
2022-05-14 2:00 PM	15.7	17.0	19.3	17.3
2022-05-14 3:00 PM	15.4	17.9	18.8	17.4
2022-05-14 4:00 PM	16.6	30.8	20.8	22.7
2022-05-14 5:00 PM	16.6	26.8	19.8	21.1
2022-05-14 6:00 PM	17.8	25.3	20.8	21.3
2022-05-14 7:00 PM	18.1	26.8	22.5	22.5
2022-05-14 8:00 PM	16.9	19.8	20.0	18.9
2022-05-14 9:00 PM	15.4	17.2	17.5	16.7
2022-05-14 10:00 PM	13.7	14.9	14.9	14.5
2022-05-14 11:00 PM	13.3	15.0	14.6	14.3
2022-05-15 12:00 AM	12.3	13.9	13.3	13.2
2022-05-15 1:00 AM	11.3	12.3	11.7	11.8
2022-05-15 2:00 AM	10.4	11.2	10.5	10.7
2022-05-15 3:00 AM	9.6	10.4	9.5	9.8
2022-05-15 4:00 AM	8.7	9.3	8.4	8.8
2022-05-15 5:00 AM	8.6	9.0	8.2	8.6
2022-05-15 6:00 AM	8.5	8.5	8.1	8.4

2022-05-15 7:00 AM	7.6	7.8	7.1	7.5
2022-05-15 8:00 AM	7.8	8.2	7.2	7.7
2022-05-15 9:00 AM	9.8	10.3	9.5	9.9
2022-05-15 10:00 AM	12.8	12.7	12.5	12.7
2022-05-15 11:00 AM	14.6	14.3	14.3	14.4
2022-05-15 12:00 PM	15.7	15.3	15.7	15.6
2022-05-15 1:00 PM	16.5	16.7	17.7	17.0
2022-05-15 2:00 PM	17.3	18.6	19.6	18.5
2022-05-15 3:00 PM	17.2	18.5	18.5	18.1
2022-05-15 4:00 PM	17.0	22.1	18.6	19.2
2022-05-15 5:00 PM	17.3	22.0	19.1	19.5
2022-05-15 6:00 PM	17.1	18.7	18.0	17.9
2022-05-15 7:00 PM	15.8	16.8	16.8	16.5
2022-05-15 8:00 PM	14.9	15.6	15.5	15.3
2022-05-15 9:00 PM	13.7	15.0	14.4	14.4
2022-05-15 10:00 PM	13.1	14.1	13.9	13.7
2022-05-15 11:00 PM	12.0	12.6	12.3	12.3
2022-05-16 12:00 AM	11.2	12.0	11.4	11.5
2022-05-16 1:00 AM	11.0	11.9	11.4	11.4
2022-05-16 2:00 AM	10.4	10.9	10.5	10.6
2022-05-16 3:00 AM	9.4	9.6	9.3	9.4
2022-05-16 4:00 AM	8.7	8.8	8.5	8.7
2022-05-16 5:00 AM	8.3	8.5	8.0	8.3
2022-05-16 6:00 AM	8.0	8.3	7.4	7.9
2022-05-16 7:00 AM	8.1	8.2	7.5	7.9
2022-05-16 8:00 AM	8.8	8.6	8.1	8.5
2022-05-16 9:00 AM	10.0	9.2	8.8	9.3
2022-05-16 10:00 AM	11.0	9.8	9.5	10.1
2022-05-16 11:00 AM	11.4	9.7	8.8	10.0
2022-05-16 12:00 PM	13.1	11.5	11.0	11.9
2022-05-16 1:00 PM	13.3	13.1	13.2	13.2
2022-05-16 2:00 PM	13.6	14.7	15.4	14.6
2022-05-16 3:00 PM	14.8	15.9	15.5	15.4
2022-05-16 4:00 PM	15.3	23.2	17.5	18.7
2022-05-16 5:00 PM	14.8	18.0	16.0	16.3
2022-05-16 6:00 PM	14.5	15.5	14.7	14.9
2022-05-16 7:00 PM	12.9	14.1	13.3	13.4
2022-05-16 8:00 PM	11.9	12.7	12.3	12.3
2022-05-16 9:00 PM	11.0	11.4	11.2	11.2
2022-05-16 10:00 PM	9.2	9.7	9.4	9.4
2022-05-16 11:00 PM	9.4	9.6	9.3	9.4
2022-05-17 12:00 AM	8.2	8.5	8.1	8.3
2022-05-17 1:00 AM	7.3	7.6	6.9	7.3

2022-05-17 2:00 AM	7.0	7.2	6.6	6.9
2022-05-17 3:00 AM	5.9	5.9	5.2	5.7
2022-05-17 4:00 AM	5.7	5.9	5.0	5.5
2022-05-17 5:00 AM	5.1	5.3	4.4	4.9
2022-05-17 6:00 AM	4.2	4.1	3.6	4.0
2022-05-17 7:00 AM	3.7	3.2	2.8	3.2
2022-05-17 8:00 AM	4.2	4.0	3.3	3.8
2022-05-17 9:00 AM	5.8	6.0	5.1	5.6
2022-05-17 10:00 AM	7.7	7.4	6.9	7.3
2022-05-17 11:00 AM	11.0	9.0	9.5	9.8
2022-05-17 12:00 PM	14.0	10.3	12.4	12.2
2022-05-17 1:00 PM	11.6	11.2	13.3	12.0
2022-05-17 2:00 PM	11.8	12.4	13.7	12.6
2022-05-17 3:00 PM	12.4	14.5	14.7	13.9
2022-05-17 4:00 PM	13.5	26.6	15.8	18.6
2022-05-17 5:00 PM	13.0	23.2	14.8	17.0
2022-05-17 6:00 PM	12.5	22.0	14.1	16.2
2022-05-17 7:00 PM	11.9	15.5	12.7	13.4
2022-05-17 8:00 PM	11.2	12.3	11.8	11.8
2022-05-17 9:00 PM	9.9	10.5	10.3	10.2
2022-05-17 10:00 PM	7.9	8.3	7.7	8.0
2022-05-17 11:00 PM	6.8	7.1	6.5	6.8
2022-05-18 12:00 AM	5.9	6.1	5.5	5.8
2022-05-18 1:00 AM	5.4	5.3	5.0	5.2
2022-05-18 2:00 AM	5.5	5.3	5.1	5.3
2022-05-18 3:00 AM	6.2	5.8	5.6	5.9
2022-05-18 4:00 AM	6.7	6.7	5.9	6.4
2022-05-18 5:00 AM	6.5	6.4	5.9	6.3
2022-05-18 6:00 AM	6.5	6.4	6.0	6.3
2022-05-18 7:00 AM	6.7	6.7	6.3	6.6
2022-05-18 8:00 AM	7.1	7.1	6.6	6.9
2022-05-18 9:00 AM	7.4	7.3	6.5	7.1
2022-05-18 10:00 AM	9.2	8.2	7.5	8.3
2022-05-18 11:00 AM	9.4	8.7	7.9	8.7
2022-05-18 12:00 PM	9.6	9.1	8.5	9.1
2022-05-18 1:00 PM	8.7	8.3	8.2	8.4
2022-05-18 2:00 PM	10.1	9.5	10.0	9.9
2022-05-18 3:00 PM	9.3	9.0	9.0	9.1
2022-05-18 4:00 PM	8.3	7.7	7.4	7.8
2022-05-18 5:00 PM	8.0	7.6	7.0	7.5
2022-05-18 6:00 PM	7.0	6.6	5.9	6.5
2022-05-18 7:00 PM	7.5	7.7	6.4	7.2
2022-05-18 8:00 PM	6.7	6.3	5.3	6.1

2022-05-18 9:00 PM	6.3	5.9	5.1	5.8
2022-05-18 10:00 PM	6.3	6.1	5.2	5.9
2022-05-18 11:00 PM	6.0	5.8	4.9	5.6
2022-05-19 12:00 AM	5.9	5.7	4.9	5.5
2022-05-19 1:00 AM	6.0	5.8	5.0	5.6
2022-05-19 2:00 AM	6.1	5.9	4.9	5.6
2022-05-19 3:00 AM	6.1	5.9	4.9	5.6
2022-05-19 4:00 AM	6.0	5.9	5.0	5.6
2022-05-19 5:00 AM	6.2	6.2	5.3	5.9
2022-05-19 6:00 AM	6.2	6.2	5.3	5.9
2022-05-19 7:00 AM	6.5	6.4	5.7	6.2
2022-05-19 8:00 AM	7.0	6.9	6.3	6.7
2022-05-19 9:00 AM	7.6	7.6	7.0	7.4
2022-05-19 10:00 AM	8.7	8.5	8.1	8.4
2022-05-19 11:00 AM	11.2	10.4	10.7	10.8
2022-05-19 12:00 PM	12.7	11.7	12.5	12.3
2022-05-19 1:00 PM	13.1	12.7	14.1	13.3
2022-05-19 2:00 PM	12.8	12.4	13.1	12.8
2022-05-19 3:00 PM	13.5	13.7	14.4	13.9
2022-05-19 4:00 PM	13.0	13.4	13.6	13.3
2022-05-19 5:00 PM	12.9	14.4	13.7	13.7
2022-05-19 6:00 PM	12.2	12.9	12.4	12.5
2022-05-19 7:00 PM	11.9	12.1	12.1	12.0
2022-05-19 8:00 PM	10.4	11.2	10.7	10.8
2022-05-19 9:00 PM	8.9	9.1	8.6	8.9
2022-05-19 10:00 PM	7.8	7.9	7.0	7.6
2022-05-19 11:00 PM	7.9	7.8	7.0	7.6
2022-05-20 12:00 AM	7.5	7.4	6.7	7.2
2022-05-20 1:00 AM	6.9	6.8	6.1	6.6
2022-05-20 2:00 AM	6.4	6.4	5.6	6.1
2022-05-20 3:00 AM	6.4	6.3	5.6	6.1
2022-05-20 4:00 AM	6.9	6.6	6.1	6.5
2022-05-20 5:00 AM	7.1	7.0	6.5	6.9
2022-05-20 6:00 AM	7.3	7.3	6.7	7.1
2022-05-20 7:00 AM	6.8	6.9	6.2	6.6
2022-05-20 8:00 AM	6.5	6.5	5.7	6.2
2022-05-20 9:00 AM	7.1	7.1	6.2	6.8
2022-05-20 10:00 AM	7.8	7.8	7.1	7.6
2022-05-20 11:00 AM	8.2	8.2	7.6	8.0
2022-05-20 12:00 PM	9.4	9.3	9.5	9.4
2022-05-20 1:00 PM	12.8	12.8	17.7	14.4
2022-05-20 2:00 PM	13.3	14.3	18.3	15.3
2022-05-20 3:00 PM	14.2	15.9	21.9	17.3

2022-05-20 4:00 PM	15.1	22.7	25.1	21.0
2022-05-20 5:00 PM	15.0	19.1	19.4	17.8
2022-05-20 6:00 PM	14.8	20.0	18.9	17.9
2022-05-20 7:00 PM	14.2	18.3	17.1	16.5
2022-05-20 8:00 PM	13.5	15.4	15.5	14.8
2022-05-20 9:00 PM	12.7	13.9	13.5	13.4
2022-05-20 10:00 PM	11.1	11.3	11.1	11.2
2022-05-20 11:00 PM	9.0	8.5	8.3	8.6
2022-05-21 12:00 AM	7.7	7.0	6.8	7.2
2022-05-21 1:00 AM	7.0	6.2	6.1	6.4
2022-05-21 2:00 AM	6.6	5.6	5.5	5.9
2022-05-21 3:00 AM	6.1	5.2	5.1	5.5
2022-05-21 4:00 AM	5.6	4.8	4.5	5.0
2022-05-21 5:00 AM	5.0	4.6	3.8	4.5
2022-05-21 6:00 AM	4.0	4.3	2.8	3.7
2022-05-21 7:00 AM	3.4	3.9	2.1	3.1
2022-05-21 8:00 AM	4.3	4.6	3.1	4.0
2022-05-21 9:00 AM	6.6	5.9	5.6	6.0
2022-05-21 10:00 AM	8.0	7.2	7.5	7.6
2022-05-21 11:00 AM	9.0	8.1	8.8	8.6
2022-05-21 12:00 PM	9.6	8.8	11.0	9.8
2022-05-21 1:00 PM	11.3	10.7	14.9	12.3
2022-05-21 2:00 PM	10.8	10.6	11.7	11.0
2022-05-21 3:00 PM	10.3	10.5	11.0	10.6
2022-05-21 4:00 PM	11.1	11.4	12.3	11.6
2022-05-21 5:00 PM	11.9	13.6	14.3	13.3
2022-05-21 6:00 PM	11.4	12.6	13.3	12.4
2022-05-21 7:00 PM	9.9	10.0	10.1	10.0
2022-05-21 8:00 PM	8.8	8.9	8.5	8.7
2022-05-21 9:00 PM	8.3	8.5	8.1	8.3
2022-05-21 10:00 PM	7.9	7.9	7.4	7.7
2022-05-21 11:00 PM	7.4	7.5	6.8	7.2
2022-05-22 12:00 AM	7.1	7.2	6.5	6.9
2022-05-22 1:00 AM	6.9	7.0	6.1	6.7
2022-05-22 2:00 AM	6.5	6.4	5.5	6.1
2022-05-22 3:00 AM	5.7	5.8	4.9	5.5
2022-05-22 4:00 AM	5.4	5.4	4.5	5.1
2022-05-22 5:00 AM	5.2	5.3	4.3	4.9
2022-05-22 6:00 AM	5.4	5.2	4.6	5.1
2022-05-22 7:00 AM	5.5	5.5	4.7	5.2
2022-05-22 8:00 AM	5.9	5.8	5.0	5.6
2022-05-22 9:00 AM	6.9	6.8	6.4	6.7
2022-05-22 10:00 AM	8.9	8.0	8.6	8.5

2022-05-22 11:00 AM	9.6	8.8	9.6	9.3
2022-05-22 12:00 PM	10.7	9.1	14.1	11.3
2022-05-22 1:00 PM	10.2	10.3	14.9	11.8
2022-05-22 2:00 PM	11.1	11.6	15.4	12.7
2022-05-22 3:00 PM	11.8	13.2	18.8	14.6
2022-05-22 4:00 PM	10.8	11.5	13.2	11.8
2022-05-22 5:00 PM	11.4	12.9	14.1	12.8
2022-05-22 6:00 PM	11.1	16.1	15.1	14.1
2022-05-22 7:00 PM	10.3	12.5	12.3	11.7
2022-05-22 8:00 PM	9.2	10.6	10.0	9.9
2022-05-22 9:00 PM	8.8	9.5	9.2	9.2
2022-05-22 10:00 PM	7.4	8.1	6.9	7.5
2022-05-22 11:00 PM	6.4	6.6	5.4	6.1
2022-05-23 12:00 AM	5.7	5.6	4.6	5.3
2022-05-23 1:00 AM	5.1	4.9	3.9	4.6
2022-05-23 2:00 AM	4.3	4.3	3.0	3.9
2022-05-23 3:00 AM	3.7	4.0	2.4	3.4
2022-05-23 4:00 AM	3.3	3.4	2.0	2.9
2022-05-23 5:00 AM	2.8	2.8	1.7	2.4
2022-05-23 6:00 AM	2.8	3.0	1.5	2.4
2022-05-23 7:00 AM	2.8	2.7	1.5	2.3
2022-05-23 8:00 AM	3.7	3.5	2.7	3.3
2022-05-23 9:00 AM	5.5	5.7	4.8	5.3
2022-05-23 10:00 AM	8.0	7.7	8.1	7.9
2022-05-23 11:00 AM	11.1	9.7	11.3	10.7
2022-05-23 12:00 PM	13.3	11.0	18.2	14.2
2022-05-23 1:00 PM	13.1	13.0	17.5	14.5
2022-05-23 2:00 PM	12.5	13.7	18.1	14.8
2022-05-23 3:00 PM	13.4	16.1	22.4	17.3
2022-05-23 4:00 PM	14.0	24.1	22.3	20.1
2022-05-23 5:00 PM	14.1	24.1	19.6	19.3
2022-05-23 6:00 PM	13.2	19.5	16.6	16.4
2022-05-23 7:00 PM	12.8	16.0	14.9	14.6
2022-05-23 8:00 PM	12.0	13.4	13.2	12.9
2022-05-23 9:00 PM	11.0	12.0	11.9	11.6
2022-05-23 10:00 PM	9.9	10.6	10.2	10.2
2022-05-23 11:00 PM	8.7	9.2	8.2	8.7
2022-05-24 12:00 AM	7.7	8.0	6.7	7.5
2022-05-24 1:00 AM	6.8	6.9	5.6	6.4
2022-05-24 2:00 AM	6.2	6.4	5.0	5.9
2022-05-24 3:00 AM	5.6	5.8	4.4	5.3
2022-05-24 4:00 AM	5.0	5.1	3.7	4.6
2022-05-24 5:00 AM	4.6	4.2	3.4	4.1

2022-05-24 6:00 AM	4.2	3.9	3.1	3.7
2022-05-24 7:00 AM	4.9	4.6	3.8	4.4
2022-05-24 8:00 AM	5.8	5.9	4.8	5.5
2022-05-24 9:00 AM	8.3	8.0	7.7	8.0
2022-05-24 10:00 AM	9.9	9.4	9.7	9.7
2022-05-24 11:00 AM	12.0	10.8	12.2	11.7
2022-05-24 12:00 PM	14.6	12.1	20.6	15.8
2022-05-24 1:00 PM	14.1	14.1	27.4	18.5
2022-05-24 2:00 PM	14.4	15.4	21.5	17.1
2022-05-24 3:00 PM	15.6	17.5	21.0	18.0
2022-05-24 4:00 PM	16.4	22.5	21.3	20.1
2022-05-24 5:00 PM	16.4	20.4	19.4	18.7
2022-05-24 6:00 PM	16.0	20.9	19.4	18.8
2022-05-24 7:00 PM	16.2	20.2	19.6	18.7
2022-05-24 8:00 PM	15.1	16.7	17.2	16.3
2022-05-24 9:00 PM	13.9	14.9	15.3	14.7
2022-05-24 10:00 PM	12.6	13.0	12.9	12.8
2022-05-24 11:00 PM	10.9	11.3	10.3	10.8
2022-05-25 12:00 AM	10.0	10.4	9.1	9.8
2022-05-25 1:00 AM	9.4	9.7	8.4	9.2
2022-05-25 2:00 AM	9.0	9.3	7.9	8.7
2022-05-25 3:00 AM	8.6	8.9	7.4	8.3
2022-05-25 4:00 AM	8.3	8.4	7.0	7.9
2022-05-25 5:00 AM	8.0	8.2	6.6	7.6
2022-05-25 6:00 AM	7.8	8.0	6.6	7.5
2022-05-25 7:00 AM	8.0	8.3	6.7	7.7
2022-05-25 8:00 AM	8.7	8.8	7.7	8.4
2022-05-25 9:00 AM	9.3	9.2	8.4	9.0
2022-05-25 10:00 AM	9.7	9.7	9.2	9.5
2022-05-25 11:00 AM	9.9	9.8	9.4	9.7
2022-05-25 12:00 PM	10.0	10.1	9.8	10.0
2022-05-25 1:00 PM	10.7	10.8	10.7	10.7
2022-05-25 2:00 PM	11.7	11.8	12.1	11.9
2022-05-25 3:00 PM	11.9	12.1	12.3	12.1
2022-05-25 4:00 PM	11.2	11.1	11.1	11.1
2022-05-25 5:00 PM	10.4	10.2	9.9	10.2
2022-05-25 6:00 PM	9.9	9.7	9.3	9.6
2022-05-25 7:00 PM	9.9	9.7	9.4	9.7
2022-05-25 8:00 PM	9.6	9.5	9.2	9.4
2022-05-25 9:00 PM	9.4	9.3	8.9	9.2
2022-05-25 10:00 PM	9.2	9.1	8.7	9.0
2022-05-25 11:00 PM	9.1	9.0	8.6	8.9
2022-05-26 12:00 AM	9.1	9.0	8.7	8.9

2022-05-26 1:00 AM	9.3	9.3	8.9	9.2
2022-05-26 2:00 AM	9.3	9.3	9.0	9.2
2022-05-26 3:00 AM	9.3	9.3	9.0	9.2
2022-05-26 4:00 AM	9.3	9.3	8.9	9.2
2022-05-26 5:00 AM	9.3	9.3	8.9	9.2
2022-05-26 6:00 AM	9.2	9.2	8.9	9.1
2022-05-26 7:00 AM	9.2	9.2	8.9	9.1
2022-05-26 8:00 AM	9.4	9.4	9.1	9.3
2022-05-26 9:00 AM	9.9	9.9	9.7	9.8
2022-05-26 10:00 AM	11.0	10.8	11.0	10.9
2022-05-26 11:00 AM	12.3	12.0	12.7	12.3
2022-05-26 12:00 PM	12.8	12.3	13.1	12.7
2022-05-26 1:00 PM	12.5	12.4	13.0	12.6
2022-05-26 2:00 PM	12.8	13.0	13.7	13.2
2022-05-26 3:00 PM	13.5	13.6	14.6	13.9
2022-05-26 4:00 PM	14.2	15.5	16.3	15.3
2022-05-26 5:00 PM	13.7	14.3	15.3	14.4
2022-05-26 6:00 PM	13.1	14.1	14.5	13.9
2022-05-26 7:00 PM	13.1	14.0	14.3	13.8
2022-05-26 8:00 PM	12.8	13.4	13.9	13.4
2022-05-26 9:00 PM	12.3	13.0	13.1	12.8
2022-05-26 10:00 PM	11.8	12.2	12.2	12.1
2022-05-26 11:00 PM	11.6	11.7	11.5	11.6
2022-05-27 12:00 AM	11.4	11.5	11.3	11.4
2022-05-27 1:00 AM	11.2	11.3	10.9	11.1
2022-05-27 2:00 AM	11.0	11.1	10.6	10.9
2022-05-27 3:00 AM	10.9	11.0	10.5	10.8
2022-05-27 4:00 AM	10.6	10.7	10.0	10.4
2022-05-27 5:00 AM	10.4	10.5	9.7	10.2
2022-05-27 6:00 AM	10.4	10.5	9.7	10.2
2022-05-27 7:00 AM	10.5	10.5	9.9	10.3
2022-05-27 8:00 AM	10.8	10.6	10.3	10.6
2022-05-27 9:00 AM	10.6	10.6	9.9	10.4
2022-05-27 10:00 AM	12.6	11.8	12.3	12.2
2022-05-27 11:00 AM	15.5	14.1	16.6	15.4
2022-05-27 12:00 PM	16.2	14.8	19.0	16.7
2022-05-27 1:00 PM	16.2	15.7	19.7	17.2
2022-05-27 2:00 PM	16.1	16.7	22.1	18.3
2022-05-27 3:00 PM	16.7	18.5	24.6	19.9
2022-05-27 4:00 PM	17.4	30.2	27.9	25.2
2022-05-27 5:00 PM	17.1	26.3	23.5	22.3
2022-05-27 6:00 PM	16.1	21.9	21.1	19.7
2022-05-27 7:00 PM	15.7	20.7	20.8	19.1

2022-05-27 8:00 PM	14.6	15.8	16.2	15.5
2022-05-27 9:00 PM	13.9	14.6	14.6	14.4
2022-05-27 10:00 PM	12.4	13.0	12.2	12.5
2022-05-27 11:00 PM	11.7	12.1	11.2	11.7
2022-05-28 12:00 AM	11.5	11.7	10.9	11.4
2022-05-28 1:00 AM	10.8	11.0	9.9	10.6
2022-05-28 2:00 AM	10.2	10.6	9.0	9.9
2022-05-28 3:00 AM	9.6	10.0	8.3	9.3
2022-05-28 4:00 AM	9.4	9.8	8.1	9.1
2022-05-28 5:00 AM	9.4	9.6	8.2	9.1
2022-05-28 6:00 AM	9.5	9.7	8.4	9.2
2022-05-28 7:00 AM	9.8	10.0	8.8	9.5
2022-05-28 8:00 AM	10.6	10.6	9.6	10.3
2022-05-28 9:00 AM	10.5	10.5	9.7	10.2
2022-05-28 10:00 AM	10.6	10.6	9.8	10.3
2022-05-28 11:00 AM	10.8	10.8	10.2	10.6
2022-05-28 12:00 PM	11.3	11.4	11.2	11.3
2022-05-28 1:00 PM	11.5	11.6	11.5	11.5
2022-05-28 2:00 PM	12.0	12.1	12.3	12.1
2022-05-28 3:00 PM	11.9	11.8	12.0	11.9
2022-05-28 4:00 PM	11.4	11.4	11.2	11.3
2022-05-28 5:00 PM	11.0	11.0	10.6	10.9
2022-05-28 6:00 PM	11.1	11.0	10.8	11.0
2022-05-28 7:00 PM	11.3	11.3	11.1	11.2
2022-05-28 8:00 PM	11.1	11.0	10.7	10.9
2022-05-28 9:00 PM	10.8	10.7	10.3	10.6
2022-05-28 10:00 PM	10.6	10.6	10.0	10.4
2022-05-28 11:00 PM	10.5	10.4	9.9	10.3
2022-05-29 12:00 AM	10.5	10.5	10.0	10.3
2022-05-29 1:00 AM	10.5	10.6	10.1	10.4
2022-05-29 2:00 AM	10.4	10.4	9.9	10.2
2022-05-29 3:00 AM	10.0	10.0	9.4	9.8
2022-05-29 4:00 AM	9.5	9.5	8.8	9.3
2022-05-29 5:00 AM	9.2	9.2	8.4	8.9
2022-05-29 6:00 AM	9.1	9.1	8.3	8.8
2022-05-29 7:00 AM	9.2	9.2	8.5	9.0
2022-05-29 8:00 AM	9.4	9.4	8.7	9.2
2022-05-29 9:00 AM	10.0	9.8	9.4	9.7
2022-05-29 10:00 AM	11.5	10.8	11.0	11.1
2022-05-29 11:00 AM	12.6	11.2	12.4	12.1
2022-05-29 12:00 PM	14.1	12.0	15.7	13.9
2022-05-29 1:00 PM	13.8	12.9	16.1	14.3
2022-05-29 2:00 PM	13.7	13.3	15.4	14.1

2022-05-29 3:00 PM	13.5	13.5	15.5	14.2
2022-05-29 4:00 PM	13.1	12.9	14.0	13.3
2022-05-29 5:00 PM	12.1	11.7	12.1	12.0
2022-05-29 6:00 PM	12.5	13.6	14.0	13.4
2022-05-29 7:00 PM	13.0	14.4	15.1	14.2
2022-05-29 8:00 PM	12.5	12.7	13.2	12.8
2022-05-29 9:00 PM	12.0	12.0	12.2	12.1
2022-05-29 10:00 PM	11.5	11.6	11.4	11.5
2022-05-29 11:00 PM	11.6	11.6	11.5	11.6
2022-05-30 12:00 AM	11.5	11.5	11.4	11.5
2022-05-30 1:00 AM	11.3	11.3	11.0	11.2
2022-05-30 2:00 AM	11.2	11.1	10.9	11.1
2022-05-30 3:00 AM	11.1	11.1	10.8	11.0
2022-05-30 4:00 AM	10.8	10.8	10.1	10.6
2022-05-30 5:00 AM	10.7	10.7	9.9	10.4
2022-05-30 6:00 AM	10.6	10.7	9.8	10.4
2022-05-30 7:00 AM	10.7	10.7	9.9	10.4
2022-05-30 8:00 AM	11.2	11.1	10.7	11.0
2022-05-30 9:00 AM	11.0	10.9	10.6	10.8
2022-05-30 10:00 AM	10.7	10.7	10.2	10.5
2022-05-30 11:00 AM	11.8	12.1	12.1	12.0
2022-05-30 12:00 PM	12.0	12.0	12.3	12.1
2022-05-30 1:00 PM	13.0	12.9	14.3	13.4
2022-05-30 2:00 PM	11.9	11.9	11.8	11.9
2022-05-30 3:00 PM	12.3	12.3	12.9	12.5
2022-05-30 4:00 PM	13.9	14.3	16.2	14.8
2022-05-30 5:00 PM	14.3	16.9	18.7	16.6
2022-05-30 6:00 PM	14.6	17.7	19.1	17.1
2022-05-30 7:00 PM	14.2	15.5	16.9	15.5
2022-05-30 8:00 PM	14.0	14.6	15.7	14.8
2022-05-30 9:00 PM	13.8	14.1	14.8	14.2
2022-05-30 10:00 PM	13.0	13.2	13.4	13.2
2022-05-30 11:00 PM	12.3	12.4	12.2	12.3
2022-05-31 12:00 AM	12.2	12.3	12.0	12.2
2022-05-31 1:00 AM	12.1	12.2	11.8	12.0
2022-05-31 2:00 AM	11.7	11.8	11.2	11.6
2022-05-31 3:00 AM	11.3	11.5	10.5	11.1
2022-05-31 4:00 AM	11.1	11.2	10.2	10.8
2022-05-31 5:00 AM	10.8	11.0	9.7	10.5
2022-05-31 6:00 AM	10.8	10.9	9.8	10.5
2022-05-31 7:00 AM	10.7	10.7	9.9	10.4
2022-05-31 8:00 AM	11.1	11.0	10.4	10.8
2022-05-31 9:00 AM	11.8	11.6	11.4	11.6

2022-05-31 10:00 AM	13.3	12.7	13.4	13.1
2022-05-31 11:00 AM	14.5	13.4	15.3	14.4
2022-05-31 12:00 PM	16.8	15.6	19.9	17.4
2022-05-31 1:00 PM	16.9	16.6	20.8	18.1
2022-05-31 2:00 PM	16.9	17.2	19.8	18.0
2022-05-31 3:00 PM	17.4	17.8	20.1	18.4
2022-05-31 4:00 PM	17.8	19.4	21.1	19.4
2022-05-31 5:00 PM	17.0	18.1	19.3	18.1
2022-05-31 6:00 PM	17.0	19.9	20.5	19.1
2022-05-31 7:00 PM	16.0	16.8	17.7	16.8
2022-05-31 8:00 PM	15.3	15.9	16.5	15.9
2022-05-31 9:00 PM	14.6	15.2	15.4	15.1
2022-05-31 10:00 PM	13.6	14.1	13.9	13.9
2022-05-31 11:00 PM	12.7	13.1	12.6	12.8
2022-06-01 12:00 AM	12.6	12.8	12.2	12.5
2022-06-01 1:00 AM	11.9	12.0	11.2	11.7
2022-06-01 2:00 AM	11.1	11.3	10.3	10.9
2022-06-01 3:00 AM	11.1	11.1	10.1	10.8
2022-06-01 4:00 AM	11.0	11.0	10.1	10.7
2022-06-01 5:00 AM	10.5	10.5	9.7	10.2
2022-06-01 6:00 AM	10.0	10.1	9.2	9.8
2022-06-01 7:00 AM	9.5	9.6	8.6	9.2
2022-06-01 8:00 AM	10.1	10.1	9.2	9.8
2022-06-01 9:00 AM	10.3	10.0	9.4	9.9
2022-06-01 10:00 AM	11.3	10.8	10.5	10.9
2022-06-01 11:00 AM	13.0	12.0	12.7	12.6
2022-06-01 12:00 PM	14.5	12.7	15.5	14.2
2022-06-01 1:00 PM	13.3	13.1	17.9	14.8
2022-06-01 2:00 PM	13.7	14.4	18.8	15.6
2022-06-01 3:00 PM	14.5	16.6	21.4	17.5
2022-06-01 4:00 PM	15.4	24.1	24.3	21.3
2022-06-01 5:00 PM	15.3	24.1	23.2	20.9
2022-06-01 6:00 PM	15.3	26.7	24.8	22.3
2022-06-01 7:00 PM	15.1	20.2	20.6	18.6
2022-06-01 8:00 PM	14.7	16.4	16.9	16.0
2022-06-01 9:00 PM	13.7	14.8	14.6	14.4
2022-06-01 10:00 PM	12.6	13.3	12.4	12.8
2022-06-01 11:00 PM	11.7	12.1	10.8	11.5
2022-06-02 12:00 AM	10.9	11.0	9.6	10.5
2022-06-02 1:00 AM	10.4	10.6	8.8	9.9
2022-06-02 2:00 AM	9.8	10.0	8.2	9.3
2022-06-02 3:00 AM	9.6	9.6	7.9	9.0
2022-06-02 4:00 AM	9.4	9.4	7.6	8.8

2022-06-02 5:00 AM	8.9	9.0	6.9	8.3
2022-06-02 6:00 AM	8.6	8.6	6.6	7.9
2022-06-02 7:00 AM	8.4	8.4	6.3	7.7
2022-06-02 8:00 AM	9.4	9.4	7.5	8.8
2022-06-02 9:00 AM	10.6	10.5	9.1	10.1
2022-06-02 10:00 AM	12.2	11.5	11.0	11.6
2022-06-02 11:00 AM	13.9	12.5	13.7	13.4
2022-06-02 12:00 PM	14.8	12.7	15.7	14.4
2022-06-02 1:00 PM	14.4	13.9	19.3	15.9
2022-06-02 2:00 PM	15.5	16.1	20.5	17.4
2022-06-02 3:00 PM	15.4	16.0	18.6	16.7
2022-06-02 4:00 PM	14.7	15.0	16.3	15.3
2022-06-02 5:00 PM	15.4	18.9	19.0	17.8
2022-06-02 6:00 PM	15.6	20.6	20.7	19.0
2022-06-02 7:00 PM	15.1	17.7	18.3	17.0
2022-06-02 8:00 PM	14.3	15.4	15.7	15.1
2022-06-02 9:00 PM	13.4	14.2	14.1	13.9
2022-06-02 10:00 PM	12.6	13.1	12.5	12.7
2022-06-02 11:00 PM	12.3	12.7	11.9	12.3
2022-06-03 12:00 AM	11.8	12.1	11.0	11.6
2022-06-03 1:00 AM	11.9	12.1	11.1	11.7
2022-06-03 2:00 AM	11.7	11.9	11.1	11.6
2022-06-03 3:00 AM	11.3	11.4	10.7	11.1
2022-06-03 4:00 AM	11.1	11.2	10.3	10.9
2022-06-03 5:00 AM	10.8	11.0	10.0	10.6
2022-06-03 6:00 AM	10.5	10.6	9.5	10.2
2022-06-03 7:00 AM	10.3	10.1	9.2	9.9
2022-06-03 8:00 AM	10.2	10.0	9.0	9.7
2022-06-03 9:00 AM	10.6	10.2	9.3	10.0
2022-06-03 10:00 AM	11.4	10.7	10.2	10.8
2022-06-03 11:00 AM	12.8	11.6	11.9	12.1
2022-06-03 12:00 PM	14.5	12.5	15.0	14.0
2022-06-03 1:00 PM	14.1	13.3	16.3	14.6
2022-06-03 2:00 PM	14.1	14.3	17.5	15.3
2022-06-03 3:00 PM	13.9	14.6	17.2	15.2
2022-06-03 4:00 PM	14.3	19.6	19.2	17.7
2022-06-03 5:00 PM	14.3	17.3	17.2	16.3
2022-06-03 6:00 PM	13.0	13.7	14.2	13.6
2022-06-03 7:00 PM	12.6	12.4	12.9	12.6
2022-06-03 8:00 PM	11.8	11.7	12.1	11.9
2022-06-03 9:00 PM	11.6	11.7	11.6	11.6
2022-06-03 10:00 PM	11.5	11.5	11.3	11.4
2022-06-03 11:00 PM	10.9	11.1	10.4	10.8

2022-06-04 12:00 AM	10.7	10.9	9.8	10.5
2022-06-04 1:00 AM	10.2	10.4	9.4	10.0
2022-06-04 2:00 AM	8.9	9.2	7.6	8.6
2022-06-04 3:00 AM	8.4	8.5	6.8	7.9
2022-06-04 4:00 AM	8.0	8.0	6.4	7.5
2022-06-04 5:00 AM	7.6	7.6	6.0	7.1
2022-06-04 6:00 AM	6.9	7.1	5.2	6.4
2022-06-04 7:00 AM	7.0	7.2	5.3	6.5
2022-06-04 8:00 AM	8.3	8.3	7.0	7.9
2022-06-04 9:00 AM	9.4	9.0	8.1	8.8
2022-06-04 10:00 AM	11.0	10.1	9.4	10.2
2022-06-04 11:00 AM	12.5	11.0	11.5	11.7
2022-06-04 12:00 PM	13.8	11.7	14.9	13.5
2022-06-04 1:00 PM	13.5	12.7	17.6	14.6
2022-06-04 2:00 PM	13.6	13.7	17.7	15.0
2022-06-04 3:00 PM	14.2	15.6	18.9	16.2
2022-06-04 4:00 PM	14.1	21.2	21.3	18.9
2022-06-04 5:00 PM	13.8	21.6	20.1	18.5
2022-06-04 6:00 PM	13.0	20.3	19.2	17.5
2022-06-04 7:00 PM	12.7	16.6	16.9	15.4
2022-06-04 8:00 PM	11.8	13.0	12.8	12.5
2022-06-04 9:00 PM	11.3	12.0	11.5	11.6
2022-06-04 10:00 PM	10.5	11.1	10.2	10.6
2022-06-04 11:00 PM	10.0	10.6	9.4	10.0
2022-06-05 12:00 AM	9.6	10.1	8.9	9.5
2022-06-05 1:00 AM	9.1	9.4	8.2	8.9
2022-06-05 2:00 AM	8.5	8.8	7.2	8.2
2022-06-05 3:00 AM	8.0	8.2	6.6	7.6
2022-06-05 4:00 AM	7.5	7.7	6.2	7.1
2022-06-05 5:00 AM	7.1	7.3	5.7	6.7
2022-06-05 6:00 AM	6.8	7.0	5.4	6.4
2022-06-05 7:00 AM	6.5	6.9	5.1	6.2
2022-06-05 8:00 AM	7.2	7.6	5.8	6.9
2022-06-05 9:00 AM	8.8	9.0	7.5	8.4
2022-06-05 10:00 AM	10.9	10.1	9.4	10.1
2022-06-05 11:00 AM	12.4	11.2	11.8	11.8
2022-06-05 12:00 PM	13.6	12.3	14.9	13.6
2022-06-05 1:00 PM	14.1	13.5	17.4	15.0
2022-06-05 2:00 PM	13.7	14.7	18.6	15.7
2022-06-05 3:00 PM	13.9	16.0	20.0	16.6
2022-06-05 4:00 PM	14.6	22.8	22.3	19.9
2022-06-05 5:00 PM	14.4	18.4	18.7	17.2
2022-06-05 6:00 PM	14.3	21.2	21.1	18.9

2022-06-05 7:00 PM	13.4	16.7	17.9	16.0
2022-06-05 8:00 PM	12.6	13.6	13.8	13.3
2022-06-05 9:00 PM	11.9	12.6	12.2	12.2
2022-06-05 10:00 PM	11.2	11.8	11.1	11.4
2022-06-05 11:00 PM	10.9	11.4	10.5	10.9
2022-06-06 12:00 AM	10.7	11.2	10.1	10.7
2022-06-06 1:00 AM	10.4	10.9	9.7	10.3
2022-06-06 2:00 AM	10.6	11.0	10.0	10.5
2022-06-06 3:00 AM	10.7	11.2	10.2	10.7
2022-06-06 4:00 AM	10.8	11.2	10.3	10.8
2022-06-06 5:00 AM	11.2	11.3	10.6	11.0
2022-06-06 6:00 AM	11.0	11.4	10.5	11.0
2022-06-06 7:00 AM	11.2	11.4	10.6	11.1
2022-06-06 8:00 AM	11.7	11.6	11.2	11.5
2022-06-06 9:00 AM	11.7	11.9	11.4	11.7
2022-06-06 10:00 AM	11.8	11.7	11.5	11.7
2022-06-06 11:00 AM	12.2	12.2	12.1	12.2
2022-06-06 12:00 PM	12.3	12.3	12.2	12.3
2022-06-06 1:00 PM	13.2	13.0	13.2	13.1
2022-06-06 2:00 PM	14.6	14.9	16.8	15.4
2022-06-06 3:00 PM	15.1	16.5	20.5	17.4
2022-06-06 4:00 PM	16.0	22.1	24.3	20.8
2022-06-06 5:00 PM	15.8	21.9	22.7	20.1
2022-06-06 6:00 PM	15.2	22.1	23.0	20.1
2022-06-06 7:00 PM	14.8	18.6	20.2	17.9
2022-06-06 8:00 PM	14.2	15.5	16.0	15.2
2022-06-06 9:00 PM	14.1	14.9	15.1	14.7
2022-06-06 10:00 PM	13.4	14.1	14.0	13.8
2022-06-06 11:00 PM	12.2	12.8	12.0	12.3
2022-06-07 12:00 AM	11.7	12.3	11.2	11.7
2022-06-07 1:00 AM	11.2	11.8	10.5	11.2
2022-06-07 2:00 AM	11.1	11.5	10.2	10.9
2022-06-07 3:00 AM	10.6	11.2	9.6	10.5
2022-06-07 4:00 AM	9.8	10.5	8.9	9.7
2022-06-07 5:00 AM	9.3	9.9	8.3	9.2
2022-06-07 6:00 AM	9.0	9.6	7.9	8.8
2022-06-07 7:00 AM	9.1	9.6	7.9	8.9
2022-06-07 8:00 AM	9.8	10.3	8.6	9.6
2022-06-07 9:00 AM	11.1	11.3	10.2	10.9
2022-06-07 10:00 AM	12.9	13.0	12.4	12.8
2022-06-07 11:00 AM	14.0	14.5	15.0	14.5
2022-06-07 12:00 PM	14.0	14.4	15.4	14.6
2022-06-07 1:00 PM	14.5	14.9	17.6	15.7

2022-06-07 2:00 PM	14.8	15.1	17.5	15.8
2022-06-07 3:00 PM	14.6	15.7	16.9	15.7
2022-06-07 4:00 PM	15.8	19.5	20.9	18.7
2022-06-07 5:00 PM	15.0	18.3	19.1	17.5
2022-06-07 6:00 PM	15.1	18.7	19.4	17.7
2022-06-07 7:00 PM	15.2	17.1	18.6	17.0
2022-06-07 8:00 PM	14.5	15.3	15.8	15.2
2022-06-07 9:00 PM	14.4	14.9	15.1	14.8
2022-06-07 10:00 PM	13.2	13.6	13.0	13.3
2022-06-07 11:00 PM	12.8	13.1	12.3	12.7
2022-06-08 12:00 AM	12.0	12.4	11.4	11.9
2022-06-08 1:00 AM	11.9	12.2	11.1	11.7
2022-06-08 2:00 AM	11.8	12.1	11.1	11.7
2022-06-08 3:00 AM	11.5	11.9	10.6	11.3
2022-06-08 4:00 AM	11.0	11.3	10.0	10.8
2022-06-08 5:00 AM	10.7	11.1	9.6	10.5
2022-06-08 6:00 AM	11.2	11.5	10.3	11.0
2022-06-08 7:00 AM	11.5	11.7	10.6	11.3
2022-06-08 8:00 AM	12.1	12.1	11.2	11.8
2022-06-08 9:00 AM	13.0	12.8	12.4	12.7
2022-06-08 10:00 AM	14.2	13.4	13.4	13.7
2022-06-08 11:00 AM	15.3	14.8	15.7	15.3
2022-06-08 12:00 PM	16.6	15.5	18.7	16.9
2022-06-08 1:00 PM	16.1	15.8	19.2	17.0
2022-06-08 2:00 PM	15.6	15.6	17.9	16.4
2022-06-08 3:00 PM	14.6	14.3	14.7	14.5
2022-06-08 4:00 PM	14.7	14.5	14.9	14.7
2022-06-08 5:00 PM	15.1	15.2	15.8	15.4
2022-06-08 6:00 PM	15.7	16.7	17.7	16.7
2022-06-08 7:00 PM	15.3	16.2	17.5	16.3
2022-06-08 8:00 PM	15.2	15.4	16.2	15.6
2022-06-08 9:00 PM	14.7	14.8	15.1	14.9
2022-06-08 10:00 PM	14.0	14.0	13.9	14.0
2022-06-08 11:00 PM	13.4	13.4	13.0	13.3
2022-06-09 12:00 AM	13.2	13.1	12.6	13.0
2022-06-09 1:00 AM	13.1	13.1	12.5	12.9
2022-06-09 2:00 AM	12.8	12.8	12.1	12.6
2022-06-09 3:00 AM	12.2	12.3	11.3	11.9
2022-06-09 4:00 AM	11.8	11.9	10.7	11.5
2022-06-09 5:00 AM	11.9	12.1	10.9	11.6
2022-06-09 6:00 AM	11.9	12.1	11.0	11.7
2022-06-09 7:00 AM	12.0	12.0	11.1	11.7
2022-06-09 8:00 AM	12.7	12.6	11.9	12.4

2022-06-09 9:00 AM	13.4	13.2	13.0	13.2
2022-06-09 10:00 AM	14.0	13.2	12.9	13.4
2022-06-09 11:00 AM	14.6	14.0	14.4	14.3
2022-06-09 12:00 PM	15.2	14.5	16.1	15.3
2022-06-09 1:00 PM	15.9	15.6	18.7	16.7
2022-06-09 2:00 PM	15.3	15.3	16.6	15.7
2022-06-09 3:00 PM	15.5	16.1	17.7	16.4
2022-06-09 4:00 PM	16.2	19.2	20.3	18.6
2022-06-09 5:00 PM	15.6	17.5	18.2	17.1
2022-06-09 6:00 PM	15.5	18.7	19.1	17.8
2022-06-09 7:00 PM	15.4	17.5	18.7	17.2
2022-06-09 8:00 PM	15.0	15.7	16.3	15.7
2022-06-09 9:00 PM	15.0	15.4	15.6	15.3
2022-06-09 10:00 PM	14.8	15.0	15.0	14.9
2022-06-09 11:00 PM	13.6	14.0	13.3	13.6
2022-06-10 12:00 AM	13.2	13.5	12.6	13.1
2022-06-10 1:00 AM	13.0	13.3	12.4	12.9
2022-06-10 2:00 AM	12.5	12.6	11.6	12.2
2022-06-10 3:00 AM	12.4	12.6	11.6	12.2
2022-06-10 4:00 AM	12.2	12.4	11.4	12.0
2022-06-10 5:00 AM	11.6	11.8	10.5	11.3
2022-06-10 6:00 AM	11.2	11.4	9.9	10.8
2022-06-10 7:00 AM	11.2	11.3	9.9	10.8
2022-06-10 8:00 AM	11.2	11.4	10.0	10.9
2022-06-10 9:00 AM	12.3	12.4	11.2	12.0
2022-06-10 10:00 AM	14.0	13.2	12.9	13.4
2022-06-10 11:00 AM	14.8	14.3	14.8	14.6
2022-06-10 12:00 PM	15.9	15.2	16.8	16.0
2022-06-10 1:00 PM	16.4	16.5	19.3	17.4
2022-06-10 2:00 PM	16.1	17.3	19.6	17.7
2022-06-10 3:00 PM	16.1	17.8	20.4	18.1
2022-06-10 4:00 PM	16.8	24.2	24.8	21.9
2022-06-10 5:00 PM	16.3	21.3	21.8	19.8
2022-06-10 6:00 PM	16.5	22.7	23.6	20.9
2022-06-10 7:00 PM	16.2	19.6	21.4	19.1
2022-06-10 8:00 PM	15.7	17.2	18.0	17.0
2022-06-10 9:00 PM	15.6	16.6	17.0	16.4
2022-06-10 10:00 PM	15.1	15.9	15.8	15.6
2022-06-10 11:00 PM	13.6	14.3	13.4	13.8
2022-06-11 12:00 AM	12.9	13.4	12.3	12.9
2022-06-11 1:00 AM	12.4	12.8	11.6	12.3
2022-06-11 2:00 AM	12.3	12.6	11.4	12.1
2022-06-11 3:00 AM	12.2	12.4	11.2	11.9

2022-06-11 4:00 AM	11.9	12.1	10.8	11.6
2022-06-11 5:00 AM	11.4	11.5	10.2	11.0
2022-06-11 6:00 AM	11.3	11.4	9.9	10.9
2022-06-11 7:00 AM	11.6	11.5	10.3	11.1
2022-06-11 8:00 AM	12.1	11.6	10.8	11.5
2022-06-11 9:00 AM	12.5	12.1	11.3	12.0
2022-06-11 10:00 AM	13.7	13.3	12.8	13.3
2022-06-11 11:00 AM	14.4	14.3	14.5	14.4
2022-06-11 12:00 PM	14.4	14.4	14.8	14.5
2022-06-11 1:00 PM	15.4	15.7	18.6	16.6
2022-06-11 2:00 PM	15.1	16.6	19.2	17.0
2022-06-11 3:00 PM	14.9	16.3	17.4	16.2
2022-06-11 4:00 PM	15.5	23.6	21.4	20.2
2022-06-11 5:00 PM	14.8	19.4	17.9	17.4
2022-06-11 6:00 PM	14.7	20.3	18.3	17.8
2022-06-11 7:00 PM	14.5	17.6	17.9	16.7
2022-06-11 8:00 PM	13.8	14.8	14.4	14.3
2022-06-11 9:00 PM	13.5	14.2	13.6	13.8
2022-06-11 10:00 PM	13.2	13.7	12.9	13.3
2022-06-11 11:00 PM	12.5	12.9	12.0	12.5
2022-06-12 12:00 AM	12.1	12.3	11.4	11.9
2022-06-12 1:00 AM	11.6	11.8	10.7	11.4
2022-06-12 2:00 AM	11.2	11.3	10.0	10.8
2022-06-12 3:00 AM	10.8	10.8	9.6	10.4
2022-06-12 4:00 AM	10.4	10.4	9.1	10.0
2022-06-12 5:00 AM	10.0	10.0	8.8	9.6
2022-06-12 6:00 AM	9.5	9.6	8.3	9.1
2022-06-12 7:00 AM	9.4	9.5	8.2	9.0
2022-06-12 8:00 AM	9.9	10.0	8.7	9.5
2022-06-12 9:00 AM	11.1	11.2	10.0	10.8
2022-06-12 10:00 AM	12.9	12.4	11.9	12.4
2022-06-12 11:00 AM	14.1	13.8	14.3	14.1
2022-06-12 12:00 PM	15.2	14.8	16.4	15.5
2022-06-12 1:00 PM	15.3	15.5	18.7	16.5
2022-06-12 2:00 PM	14.8	15.5	17.8	16.0
2022-06-12 3:00 PM	14.7	15.3	17.3	15.8
2022-06-12 4:00 PM	14.8	18.3	20.9	18.0
2022-06-12 5:00 PM	14.4	15.6	16.7	15.6
2022-06-12 6:00 PM	14.5	15.8	16.8	15.7
2022-06-12 7:00 PM	14.4	15.1	16.0	15.2
2022-06-12 8:00 PM	14.1	14.4	14.9	14.5
2022-06-12 9:00 PM	13.9	14.1	14.2	14.1
2022-06-12 10:00 PM	13.3	13.4	12.8	13.2

2022-06-12 11:00 PM	12.9	13.0	12.1	12.7
2022-06-13 12:00 AM	12.7	12.8	11.7	12.4
2022-06-13 1:00 AM	12.9	12.9	11.9	12.6
2022-06-13 2:00 AM	12.9	12.7	11.9	12.5
2022-06-13 3:00 AM	12.7	12.6	11.8	12.4
2022-06-13 4:00 AM	12.7	12.5	11.7	12.3
2022-06-13 5:00 AM	12.6	12.5	11.7	12.3
2022-06-13 6:00 AM	12.3	12.2	11.2	11.9
2022-06-13 7:00 AM	12.1	11.9	10.7	11.6
2022-06-13 8:00 AM	12.0	11.7	10.6	11.4
2022-06-13 9:00 AM	12.1	11.8	10.8	11.6
2022-06-13 10:00 AM	12.5	12.1	11.4	12.0
2022-06-13 11:00 AM	12.4	12.1	11.4	12.0
2022-06-13 12:00 PM	12.5	12.3	11.7	12.2
2022-06-13 1:00 PM	12.9	12.6	12.4	12.6
2022-06-13 2:00 PM	12.9	12.7	12.5	12.7
2022-06-13 3:00 PM	12.6	12.4	11.9	12.3
2022-06-13 4:00 PM	12.6	12.3	11.7	12.2
2022-06-13 5:00 PM	12.3	12.1	11.4	11.9
2022-06-13 6:00 PM	12.2	12.0	11.3	11.8
2022-06-13 7:00 PM	12.2	12.0	11.2	11.8
2022-06-13 8:00 PM	12.1	11.9	11.1	11.7
2022-06-13 9:00 PM	12.2	12.1	11.5	11.9
2022-06-13 10:00 PM	12.3	12.1	11.4	11.9
2022-06-13 11:00 PM	12.0	12.0	11.2	11.7
2022-06-14 12:00 AM	12.0	12.0	11.2	11.7
2022-06-14 1:00 AM	12.0	11.9	11.2	11.7
2022-06-14 2:00 AM	12.0	11.9	11.2	11.7
2022-06-14 3:00 AM	12.0	11.8	11.1	11.6
2022-06-14 4:00 AM	11.9	11.8	11.1	11.6
2022-06-14 5:00 AM	11.9	11.8	11.1	11.6
2022-06-14 6:00 AM	11.9	11.8	11.1	11.6
2022-06-14 7:00 AM	11.9	11.8	11.2	11.6
2022-06-14 8:00 AM	12.0	11.9	11.4	11.8
2022-06-14 9:00 AM	11.9	11.8	11.3	11.7
2022-06-14 10:00 AM	12.0	11.9	11.5	11.8
2022-06-14 11:00 AM	12.1	12.0	11.8	12.0
2022-06-14 12:00 PM	11.9	11.8	11.4	11.7
2022-06-14 1:00 PM	12.0	11.9	11.6	11.8
2022-06-14 2:00 PM	12.2	12.1	11.9	12.1
2022-06-14 3:00 PM	12.4	12.4	12.4	12.4
2022-06-14 4:00 PM	12.7	12.8	13.1	12.9
2022-06-14 5:00 PM	12.9	13.0	13.6	13.2

2022-06-14 6:00 PM	13.0	13.2	13.8	13.3
2022-06-14 7:00 PM	13.1	13.3	14.0	13.5
2022-06-14 8:00 PM	13.0	13.1	13.6	13.2
2022-06-14 9:00 PM	12.7	12.7	12.8	12.7
2022-06-14 10:00 PM	12.6	12.5	12.4	12.5
2022-06-14 11:00 PM	12.6	12.6	12.4	12.5
2022-06-15 12:00 AM	12.8	12.8	12.8	12.8
2022-06-15 1:00 AM	12.9	12.9	13.0	12.9
2022-06-15 2:00 AM	12.8	12.8	12.8	12.8
2022-06-15 3:00 AM	12.7	12.6	12.4	12.6
2022-06-15 4:00 AM	12.6	12.6	12.3	12.5
2022-06-15 5:00 AM	12.6	12.6	12.3	12.5
2022-06-15 6:00 AM	12.6	12.6	12.1	12.4
2022-06-15 7:00 AM	12.6	12.6	12.1	12.4
2022-06-15 8:00 AM	12.7	12.7	12.4	12.6
2022-06-15 9:00 AM	13.0	13.0	13.1	13.0
2022-06-15 10:00 AM	14.2	13.9	14.2	14.1
2022-06-15 11:00 AM	15.8	15.5	15.9	15.7
2022-06-15 12:00 PM	17.0	16.2	17.2	16.8
2022-06-15 1:00 PM	18.1	17.4	19.0	18.2
2022-06-15 2:00 PM	16.5	16.3	17.2	16.7
2022-06-15 3:00 PM	16.8	17.2	17.6	17.2
2022-06-15 4:00 PM	16.6	21.2	20.2	19.3
2022-06-15 5:00 PM	16.9	17.9	18.0	17.6
2022-06-15 6:00 PM	16.6	16.9	17.3	16.9
2022-06-15 7:00 PM	16.2	16.6	17.0	16.6
2022-06-15 8:00 PM	15.5	15.6	15.8	15.6
2022-06-15 9:00 PM	15.2	15.2	15.3	15.2
2022-06-15 10:00 PM	13.8	13.7	13.7	13.7
2022-06-15 11:00 PM	13.2	13.1	12.9	13.1
2022-06-16 12:00 AM	13.0	12.8	12.6	12.8
2022-06-16 1:00 AM	13.1	13.0	12.7	12.9
2022-06-16 2:00 AM	13.1	13.2	12.7	13.0
2022-06-16 3:00 AM	12.8	12.7	12.3	12.6
2022-06-16 4:00 AM	12.3	12.2	11.7	12.1
2022-06-16 5:00 AM	11.9	12.0	11.4	11.8
2022-06-16 6:00 AM	11.5	11.5	11.0	11.3
2022-06-16 7:00 AM	11.6	11.4	10.9	11.3
2022-06-16 8:00 AM	12.0	11.8	11.2	11.7
2022-06-16 9:00 AM	12.6	12.1	11.8	12.2
2022-06-16 10:00 AM	13.5	13.0	12.7	13.1
2022-06-16 11:00 AM	14.9	14.1	13.9	14.3
2022-06-16 12:00 PM	18.6	15.5	17.1	17.1

2022-06-16 1:00 PM	17.6	16.6	18.2	17.5
2022-06-16 2:00 PM	16.9	16.8	18.1	17.3
2022-06-16 3:00 PM	17.1	17.1	17.8	17.3
2022-06-16 4:00 PM	17.1	19.8	18.7	18.5
2022-06-16 5:00 PM	17.3	18.3	18.2	17.9
2022-06-16 6:00 PM	17.3	21.3	19.8	19.5
2022-06-16 7:00 PM	17.1	19.0	18.7	18.3
2022-06-16 8:00 PM	16.2	17.3	17.1	16.9
2022-06-16 9:00 PM	15.9	16.6	16.4	16.3
2022-06-16 10:00 PM	15.4	16.0	15.8	15.7
2022-06-16 11:00 PM	15.0	15.6	15.2	15.3
2022-06-17 12:00 AM	14.8	15.3	14.8	15.0
2022-06-17 1:00 AM	14.6	15.1	14.6	14.8
2022-06-17 2:00 AM	14.4	14.9	14.3	14.5
2022-06-17 3:00 AM	14.1	14.6	13.8	14.2
2022-06-17 4:00 AM	13.8	14.3	13.5	13.9
2022-06-17 5:00 AM	13.9	14.3	13.5	13.9
2022-06-17 6:00 AM	13.8	14.2	13.5	13.8
2022-06-17 7:00 AM	14.0	14.4	13.7	14.0
2022-06-17 8:00 AM	14.1	14.7	14.1	14.3
2022-06-17 9:00 AM	14.1	14.9	14.1	14.4
2022-06-17 10:00 AM	16.1	15.5	14.6	15.4
2022-06-17 11:00 AM	17.3	16.3	15.9	16.5
2022-06-17 12:00 PM	21.2	17.3	18.5	19.0
2022-06-17 1:00 PM	19.5	18.5	21.2	19.7
2022-06-17 2:00 PM	17.3	19.5	21.4	19.4
2022-06-17 3:00 PM	17.3	20.8	21.4	19.8
2022-06-17 4:00 PM	17.5	29.6	25.8	24.3
2022-06-17 5:00 PM	16.6	22.2	20.3	19.7
2022-06-17 6:00 PM	17.0	28.1	22.8	22.6
2022-06-17 7:00 PM	16.5	23.0	21.3	20.3
2022-06-17 8:00 PM	15.6	18.2	17.3	17.0
2022-06-17 9:00 PM	15.2	17.3	16.4	16.3
2022-06-17 10:00 PM	14.4	15.9	15.0	15.1
2022-06-17 11:00 PM	13.3	14.5	13.4	13.7
2022-06-18 12:00 AM	12.8	13.8	12.6	13.1
2022-06-18 1:00 AM	12.0	13.0	11.6	12.2
2022-06-18 2:00 AM	11.7	12.2	11.1	11.7
2022-06-18 3:00 AM	11.3	12.0	10.7	11.3
2022-06-18 4:00 AM	10.7	11.4	10.0	10.7
2022-06-18 5:00 AM	9.9	10.9	9.1	10.0
2022-06-18 6:00 AM	9.3	10.2	8.5	9.3
2022-06-18 7:00 AM	9.1	10.1	8.3	9.2

2022-06-18 8:00 AM	10.1	10.8	9.2	10.0
2022-06-18 9:00 AM	12.0	12.4	11.0	11.8
2022-06-18 10:00 AM	14.7	13.4	12.6	13.6
2022-06-18 11:00 AM	15.9	14.5	14.5	15.0
2022-06-18 12:00 PM	17.9	14.9	16.3	16.4
2022-06-18 1:00 PM	17.1	15.6	17.9	16.9
2022-06-18 2:00 PM	15.4	16.2	18.3	16.6
2022-06-18 3:00 PM	14.7	15.5	15.4	15.2
2022-06-18 4:00 PM	14.6	25.2	19.5	19.8
2022-06-18 5:00 PM	14.3	18.3	16.3	16.3
2022-06-18 6:00 PM	14.3	20.9	17.1	17.4
2022-06-18 7:00 PM	14.0	17.8	16.6	16.1
2022-06-18 8:00 PM	13.9	15.2	14.6	14.6
2022-06-18 9:00 PM	13.2	14.3	13.7	13.7
2022-06-18 10:00 PM	12.4	13.4	12.6	12.8
2022-06-18 11:00 PM	11.7	12.6	11.7	12.0
2022-06-19 12:00 AM	11.2	12.0	10.9	11.4
2022-06-19 1:00 AM	11.2	11.8	10.8	11.3
2022-06-19 2:00 AM	11.2	11.7	10.8	11.2
2022-06-19 3:00 AM	11.8	12.2	11.3	11.8
2022-06-19 4:00 AM	11.3	11.9	11.0	11.4
2022-06-19 5:00 AM	11.1	11.7	10.8	11.2
2022-06-19 6:00 AM	11.3	11.8	11.0	11.4
2022-06-19 7:00 AM	11.1	11.5	10.6	11.1
2022-06-19 8:00 AM	11.4	11.6	10.8	11.3
2022-06-19 9:00 AM	11.5	11.7	11.0	11.4
2022-06-19 10:00 AM	9.9	10.4	9.4	9.9
2022-06-19 11:00 AM	9.9	10.1	9.2	9.7
2022-06-19 12:00 PM	10.7	10.7	9.8	10.4
2022-06-19 1:00 PM	12.1	12.2	11.6	12.0
2022-06-19 2:00 PM	13.4	13.3	13.2	13.3
2022-06-19 3:00 PM	13.0	13.3	13.5	13.3
2022-06-19 4:00 PM	13.3	14.6	13.9	13.9
2022-06-19 5:00 PM	13.2	14.2	13.4	13.6
2022-06-19 6:00 PM	13.6	13.8	13.7	13.7
2022-06-19 7:00 PM	13.4	13.6	13.6	13.5
2022-06-19 8:00 PM	13.2	13.2	13.2	13.2
2022-06-19 9:00 PM	12.6	12.6	12.5	12.6
2022-06-19 10:00 PM	11.7	11.7	11.4	11.6
2022-06-19 11:00 PM	11.6	11.4	11.1	11.4
2022-06-20 12:00 AM	11.5	11.3	11.0	11.3
2022-06-20 1:00 AM	11.6	11.6	11.2	11.5
2022-06-20 2:00 AM	11.6	11.5	11.1	11.4

2022-06-20 3:00 AM	11.5	11.5	11.1	11.4
2022-06-20 4:00 AM	11.6	11.7	11.1	11.5
2022-06-20 5:00 AM	11.3	11.5	10.9	11.2
2022-06-20 6:00 AM	11.1	11.4	10.7	11.1
2022-06-20 7:00 AM	11.4	11.6	11.0	11.3
2022-06-20 8:00 AM	12.2	12.1	11.5	11.9
2022-06-20 9:00 AM	12.3	12.1	11.6	12.0
2022-06-20 10:00 AM	14.1	12.7	12.4	13.1
2022-06-20 11:00 AM	15.5	13.5	14.1	14.4
2022-06-20 12:00 PM	18.7	14.6	16.9	16.7
2022-06-20 1:00 PM	18.1	15.7	19.7	17.8
2022-06-20 2:00 PM	16.0	16.4	19.2	17.2
2022-06-20 3:00 PM	15.6	16.2	17.1	16.3
2022-06-20 4:00 PM	16.2	26.0	22.4	21.5
2022-06-20 5:00 PM	15.3	18.6	17.6	17.2
2022-06-20 6:00 PM	15.8	22.7	19.4	19.3
2022-06-20 7:00 PM	16.5	20.8	20.6	19.3
2022-06-20 8:00 PM	16.0	17.2	17.7	17.0
2022-06-20 9:00 PM	16.1	16.8	17.3	16.7
2022-06-20 10:00 PM	15.9	16.4	16.9	16.4
2022-06-20 11:00 PM	15.4	15.9	16.0	15.8
2022-06-21 12:00 AM	15.1	15.6	15.5	15.4
2022-06-21 1:00 AM	15.0	15.4	15.3	15.2
2022-06-21 2:00 AM	15.1	15.5	15.2	15.3
2022-06-21 3:00 AM	16.4	17.2	17.3	17.0
2022-06-21 4:00 AM	16.3	16.8	17.1	16.7
2022-06-21 5:00 AM	16.2	16.6	16.9	16.6
2022-06-21 6:00 AM	16.1	16.4	16.6	16.4
2022-06-21 7:00 AM	16.4	16.7	16.8	16.6
2022-06-21 8:00 AM	16.3	16.5	16.7	16.5
2022-06-21 9:00 AM	16.3	16.1	16.0	16.1
2022-06-21 10:00 AM	17.4	16.6	16.7	16.9
2022-06-21 11:00 AM	17.9	17.1	17.5	17.5
2022-06-21 12:00 PM	20.2	18.7	20.0	19.6
2022-06-21 1:00 PM	21.2	20.8	23.1	21.7
2022-06-21 2:00 PM	20.5	21.4	23.5	21.8
2022-06-21 3:00 PM	21.0	22.3	23.6	22.3
2022-06-21 4:00 PM	21.0	30.5	28.0	26.5
2022-06-21 5:00 PM	20.0	23.1	23.2	22.1
2022-06-21 6:00 PM	19.7	25.7	25.0	23.5
2022-06-21 7:00 PM	19.1	23.9	24.7	22.6
2022-06-21 8:00 PM	18.4	20.2	20.9	19.8
2022-06-21 9:00 PM	18.1	19.5	20.0	19.2

2022-06-21 10:00 PM	17.9	19.1	19.3	18.8
2022-06-21 11:00 PM	17.6	18.6	18.5	18.2
2022-06-22 12:00 AM	17.2	18.1	17.7	17.7
2022-06-22 1:00 AM	17.0	17.7	17.3	17.3
2022-06-22 2:00 AM	16.7	17.5	16.9	17.0
2022-06-22 3:00 AM	16.6	17.3	16.8	16.9
2022-06-22 4:00 AM	16.3	16.9	16.4	16.5
2022-06-22 5:00 AM	15.5	16.1	15.2	15.6
2022-06-22 6:00 AM	15.2	15.7	14.7	15.2
2022-06-22 7:00 AM	15.1	15.6	14.5	15.1
2022-06-22 8:00 AM	15.5	15.7	14.9	15.4
2022-06-22 9:00 AM	15.5	15.4	14.7	15.2
2022-06-22 10:00 AM	16.7	15.7	15.0	15.8
2022-06-22 11:00 AM	18.1	17.0	16.9	17.3
2022-06-22 12:00 PM	21.4	18.2	19.9	19.8
2022-06-22 1:00 PM	20.3	19.5	22.5	20.8
2022-06-22 2:00 PM	18.6	20.3	22.2	20.4
2022-06-22 3:00 PM	19.2	20.7	21.8	20.6
2022-06-22 4:00 PM	19.7	24.6	24.7	23.0
2022-06-22 5:00 PM	18.9	20.8	20.9	20.2
2022-06-22 6:00 PM	18.7	24.1	21.9	21.6
2022-06-22 7:00 PM	18.6	22.6	22.8	21.3
2022-06-22 8:00 PM	17.8	18.9	18.9	18.5
2022-06-22 9:00 PM	17.3	18.1	17.9	17.8
2022-06-22 10:00 PM	16.7	17.6	17.0	17.1
2022-06-22 11:00 PM	15.9	16.7	15.8	16.1
2022-06-23 12:00 AM	15.3	16.1	14.9	15.4
2022-06-23 1:00 AM	14.9	15.5	14.3	14.9
2022-06-23 2:00 AM	14.4	14.9	13.5	14.3
2022-06-23 3:00 AM	14.1	14.5	13.0	13.9
2022-06-23 4:00 AM	13.5	14.0	12.4	13.3
2022-06-23 5:00 AM	13.2	13.6	12.1	13.0
2022-06-23 6:00 AM	13.1	13.4	12.0	12.8
2022-06-23 7:00 AM	13.0	13.3	11.8	12.7
2022-06-23 8:00 AM	13.8	14.0	12.6	13.5
2022-06-23 9:00 AM	14.3	14.5	13.4	14.1
2022-06-23 10:00 AM	16.3	15.3	14.6	15.4
2022-06-23 11:00 AM	17.6	16.3	16.7	16.9
2022-06-23 12:00 PM	20.3	17.2	19.6	19.0
2022-06-23 1:00 PM	19.7	18.1	22.2	20.0
2022-06-23 2:00 PM	17.6	18.5	20.6	18.9
2022-06-23 3:00 PM	18.3	19.0	20.1	19.1
2022-06-23 4:00 PM	19.0	25.5	24.8	23.1

2022-06-23 5:00 PM	19.0	21.3	21.9	20.7
2022-06-23 6:00 PM	18.6	21.6	21.4	20.5
2022-06-23 7:00 PM	18.5	19.8	20.8	19.7
2022-06-23 8:00 PM	18.8	19.3	20.0	19.4
2022-06-23 9:00 PM	18.2	18.6	19.0	18.6
2022-06-23 10:00 PM	17.9	18.2	18.3	18.1
2022-06-23 11:00 PM	17.5	17.8	17.7	17.7
2022-06-24 12:00 AM	17.0	17.3	16.9	17.1
2022-06-24 1:00 AM	16.1	16.3	15.7	16.0
2022-06-24 2:00 AM	15.9	16.2	15.3	15.8
2022-06-24 3:00 AM	15.6	15.9	15.0	15.5
2022-06-24 4:00 AM	14.5	14.7	13.5	14.2
2022-06-24 5:00 AM	14.2	14.4	13.2	13.9
2022-06-24 6:00 AM	14.1	14.2	12.9	13.7
2022-06-24 7:00 AM	14.2	14.4	13.0	13.9
2022-06-24 8:00 AM	14.9	15.0	13.9	14.6
2022-06-24 9:00 AM	16.1	16.1	15.5	15.9
2022-06-24 10:00 AM	17.3	16.4	16.2	16.6
2022-06-24 11:00 AM	18.2	16.9	17.3	17.5
2022-06-24 12:00 PM	20.3	17.6	19.5	19.1
2022-06-24 1:00 PM	20.1	18.5	22.0	20.2
2022-06-24 2:00 PM	18.4	18.8	21.1	19.4
2022-06-24 3:00 PM	18.8	19.2	20.1	19.4
2022-06-24 4:00 PM	18.6	22.6	22.6	21.3
2022-06-24 5:00 PM	17.8	19.4	19.0	18.7
2022-06-24 6:00 PM	17.7	20.2	19.0	19.0
2022-06-24 7:00 PM	17.6	19.5	19.4	18.8
2022-06-24 8:00 PM	17.4	18.0	17.9	17.8
2022-06-24 9:00 PM	17.2	17.7	17.5	17.5
2022-06-24 10:00 PM	16.8	17.2	16.8	16.9
2022-06-24 11:00 PM	16.4	16.8	16.1	16.4
2022-06-25 12:00 AM	16.1	16.4	15.6	16.0
2022-06-25 1:00 AM	15.7	16.1	15.2	15.7
2022-06-25 2:00 AM	15.6	16.0	15.0	15.5
2022-06-25 3:00 AM	15.8	16.1	15.2	15.7
2022-06-25 4:00 AM	15.8	16.0	15.3	15.7
2022-06-25 5:00 AM	15.5	15.6	14.9	15.3
2022-06-25 6:00 AM	15.3	15.4	14.5	15.1
2022-06-25 7:00 AM	15.1	15.2	14.3	14.9
2022-06-25 8:00 AM	15.2	15.1	14.3	14.9
2022-06-25 9:00 AM	15.6	15.3	14.6	15.2
2022-06-25 10:00 AM	15.9	15.6	15.2	15.6
2022-06-25 11:00 AM	16.1	15.9	15.6	15.9

2022-06-25 12:00 PM	15.9	15.8	15.5	15.7
2022-06-25 1:00 PM	16.0	15.8	15.5	15.8
2022-06-25 2:00 PM	16.6	16.3	16.2	16.4
2022-06-25 3:00 PM	17.1	17.1	17.3	17.2
2022-06-25 4:00 PM	17.4	21.0	20.2	19.5
2022-06-25 5:00 PM	17.2	17.8	18.0	17.7
2022-06-25 6:00 PM	17.2	18.5	18.0	17.9
2022-06-25 7:00 PM	16.8	17.3	17.4	17.2
2022-06-25 8:00 PM	16.6	16.6	16.6	16.6
2022-06-25 9:00 PM	16.4	16.5	16.3	16.4
2022-06-25 10:00 PM	15.8	16.0	15.5	15.8
2022-06-25 11:00 PM	15.2	15.5	14.7	15.1
2022-06-26 12:00 AM	14.8	15.1	14.1	14.7
2022-06-26 1:00 AM	14.6	14.9	13.9	14.5
2022-06-26 2:00 AM	14.6	14.9	13.9	14.5
2022-06-26 3:00 AM	14.7	15.1	14.1	14.6
2022-06-26 4:00 AM	14.3	14.8	13.6	14.2
2022-06-26 5:00 AM	13.9	14.5	13.1	13.8
2022-06-26 6:00 AM	13.3	13.8	12.5	13.2
2022-06-26 7:00 AM	13.1	13.4	12.1	12.9
2022-06-26 8:00 AM	13.6	14.0	12.7	13.4
2022-06-26 9:00 AM	14.5	14.6	13.9	14.3
2022-06-26 10:00 AM	15.7	14.9	14.4	15.0
2022-06-26 11:00 AM	16.6	15.6	15.7	16.0
2022-06-26 12:00 PM	17.3	16.0	16.5	16.6
2022-06-26 1:00 PM	17.2	16.4	17.0	16.9
2022-06-26 2:00 PM	17.1	16.5	17.0	16.9
2022-06-26 3:00 PM	17.2	16.8	17.3	17.1
2022-06-26 4:00 PM	16.6	19.4	18.5	18.2
2022-06-26 5:00 PM	16.3	17.3	16.9	16.8
2022-06-26 6:00 PM	16.1	17.3	17.0	16.8
2022-06-26 7:00 PM	15.5	15.7	15.5	15.6
2022-06-26 8:00 PM	15.3	15.0	14.8	15.0
2022-06-26 9:00 PM	14.7	14.4	14.0	14.4
2022-06-26 10:00 PM	14.1	13.9	13.2	13.7
2022-06-26 11:00 PM	13.5	13.5	12.5	13.2
2022-06-27 12:00 AM	13.3	13.3	12.3	13.0
2022-06-27 1:00 AM	13.2	13.0	12.1	12.8
2022-06-27 2:00 AM	13.2	13.1	12.2	12.8
2022-06-27 3:00 AM	13.0	12.8	11.9	12.6
2022-06-27 4:00 AM	12.8	12.6	11.6	12.3
2022-06-27 5:00 AM	12.5	12.6	11.4	12.2
2022-06-27 6:00 AM	12.0	12.2	10.8	11.7

2022-06-27 7:00 AM	11.3	11.6	10.0	11.0
2022-06-27 8:00 AM	11.6	12.0	10.5	11.4
2022-06-27 9:00 AM	12.6	12.8	11.6	12.3
2022-06-27 10:00 AM	14.7	13.6	12.7	13.7
2022-06-27 11:00 AM	16.0	14.8	14.9	15.2
2022-06-27 12:00 PM	18.2	15.8	17.7	17.2
2022-06-27 1:00 PM	18.0	16.7	20.2	18.3
2022-06-27 2:00 PM	16.1	16.5	17.7	16.8
2022-06-27 3:00 PM	16.8	17.3	17.8	17.3
2022-06-27 4:00 PM	16.8	19.3	19.4	18.5
2022-06-27 5:00 PM	16.3	17.7	17.7	17.2
2022-06-27 6:00 PM	16.4	18.8	18.2	17.8
2022-06-27 7:00 PM	16.5	17.8	17.8	17.4
2022-06-27 8:00 PM	16.3	16.8	16.9	16.7
2022-06-27 9:00 PM	16.0	16.4	16.3	16.2
2022-06-27 10:00 PM	15.3	15.7	15.4	15.5
2022-06-27 11:00 PM	14.3	14.6	13.8	14.2
2022-06-28 12:00 AM	13.8	14.1	12.9	13.6
2022-06-28 1:00 AM	13.8	14.0	12.9	13.6
2022-06-28 2:00 AM	14.7	15.0	14.2	14.6
2022-06-28 3:00 AM	14.9	15.2	14.6	14.9
2022-06-28 4:00 AM	15.1	15.3	14.9	15.1
2022-06-28 5:00 AM	15.0	15.3	14.7	15.0
2022-06-28 6:00 AM	14.8	15.1	14.4	14.8
2022-06-28 7:00 AM	14.8	15.1	14.3	14.7
2022-06-28 8:00 AM	15.0	15.2	14.6	14.9
2022-06-28 9:00 AM	15.1	15.2	14.7	15.0
2022-06-28 10:00 AM	16.5	15.9	15.7	16.0
2022-06-28 11:00 AM	17.4	16.6	16.9	17.0
2022-06-28 12:00 PM	17.2	16.3	16.7	16.7
2022-06-28 1:00 PM	17.0	16.1	16.4	16.5
2022-06-28 2:00 PM	16.4	15.8	15.9	16.0
2022-06-28 3:00 PM	17.3	17.0	17.6	17.3
2022-06-28 4:00 PM	17.3	21.3	21.6	20.1
2022-06-28 5:00 PM	16.8	18.0	18.1	17.6
2022-06-28 6:00 PM	16.8	20.9	19.9	19.2
2022-06-28 7:00 PM	16.4	19.3	19.4	18.4
2022-06-28 8:00 PM	15.8	16.5	16.3	16.2
2022-06-28 9:00 PM	15.3	15.8	15.4	15.5
2022-06-28 10:00 PM	14.8	15.3	14.6	14.9
2022-06-28 11:00 PM	13.9	14.5	13.1	13.8
2022-06-29 12:00 AM	13.2	13.6	12.2	13.0
2022-06-29 1:00 AM	12.7	12.8	11.6	12.4

2022-06-29 2:00 AM	12.0	12.3	10.8	11.7
2022-06-29 3:00 AM	11.5	11.9	10.1	11.2
2022-06-29 4:00 AM	11.3	11.6	9.8	10.9
2022-06-29 5:00 AM	10.8	11.3	9.3	10.5
2022-06-29 6:00 AM	10.5	10.8	8.9	10.1
2022-06-29 7:00 AM	10.0	10.4	8.3	9.6
2022-06-29 8:00 AM	10.6	10.9	9.0	10.2
2022-06-29 9:00 AM	12.4	12.6	11.2	12.1
2022-06-29 10:00 AM	14.8	13.7	13.0	13.8
2022-06-29 11:00 AM	16.2	15.1	15.7	15.7
2022-06-29 12:00 PM	18.5	16.2	18.6	17.8
2022-06-29 1:00 PM	17.8	16.6	19.1	17.8
2022-06-29 2:00 PM	16.2	16.2	17.6	16.7
2022-06-29 3:00 PM	15.8	15.9	15.8	15.8
2022-06-29 4:00 PM	16.3	16.7	16.9	16.6
2022-06-29 5:00 PM	16.1	16.0	16.5	16.2
2022-06-29 6:00 PM	15.8	15.6	15.6	15.7
2022-06-29 7:00 PM	15.5	15.2	15.0	15.2
2022-06-29 8:00 PM	14.8	14.6	14.2	14.5
2022-06-29 9:00 PM	14.2	14.1	13.2	13.8
2022-06-29 10:00 PM	13.6	13.6	12.8	13.3
2022-06-29 11:00 PM	13.4	13.5	12.6	13.2
2022-06-30 12:00 AM	13.3	13.3	12.5	13.0
2022-06-30 1:00 AM	13.0	13.0	12.2	12.7
2022-06-30 2:00 AM	12.8	12.4	11.7	12.3
2022-06-30 3:00 AM	12.9	12.7	11.9	12.5
2022-06-30 4:00 AM	12.9	12.9	12.1	12.6
2022-06-30 5:00 AM	13.1	13.1	12.4	12.9
2022-06-30 6:00 AM	13.1	13.1	12.4	12.9
2022-06-30 7:00 AM	13.2	13.3	12.6	13.0
2022-06-30 8:00 AM	13.6	13.5	12.9	13.3
2022-06-30 9:00 AM	13.7	13.7	13.2	13.5
2022-06-30 10:00 AM	14.0	14.0	13.4	13.8
2022-06-30 11:00 AM	16.2	15.1	14.2	15.2
2022-06-30 12:00 PM	17.8	16.9	17.9	17.5
2022-06-30 1:00 PM	17.4	16.8	19.3	17.8
2022-06-30 2:00 PM	16.5	17.0	18.7	17.4
2022-06-30 3:00 PM	16.5	16.8	17.7	17.0
2022-06-30 4:00 PM	17.2	20.9	21.6	19.9
2022-06-30 5:00 PM	17.5	19.2	20.4	19.0
2022-06-30 6:00 PM	18.0	21.4	22.2	20.5
2022-06-30 7:00 PM	17.8	20.8	22.1	20.2
2022-06-30 8:00 PM	17.4	18.8	19.4	18.5

2022-06-30 9:00 PM	17.0	18.1	18.4	17.8
2022-06-30 10:00 PM	16.6	17.4	17.4	17.1
2022-06-30 11:00 PM	16.1	16.7	16.3	16.4
2022-07-01 12:00 AM	15.6	16.2	15.5	15.8
2022-07-01 1:00 AM	15.5	15.8	15.2	15.5
2022-07-01 2:00 AM	15.0	15.5	14.6	15.0
2022-07-01 3:00 AM	14.4	14.7	13.6	14.2
2022-07-01 4:00 AM	13.8	14.1	12.8	13.6
2022-07-01 5:00 AM	13.2	13.2	12.0	12.8
2022-07-01 6:00 AM	12.8	13.0	11.6	12.5
2022-07-01 7:00 AM	12.6	12.8	11.5	12.3
2022-07-01 8:00 AM	12.7	12.9	11.6	12.4
2022-07-01 9:00 AM	13.8	13.9	12.7	13.5
2022-07-01 10:00 AM	15.3	14.8	14.0	14.7
2022-07-01 11:00 AM	16.0	15.8	15.7	15.8
2022-07-01 12:00 PM	16.8	16.6	17.8	17.1
2022-07-01 1:00 PM	16.4	16.7	17.7	16.9
2022-07-01 2:00 PM	16.3	16.7	17.3	16.8
2022-07-01 3:00 PM	16.4	17.1	17.4	17.0
2022-07-01 4:00 PM	17.0	21.9	23.1	20.7
2022-07-01 5:00 PM	16.5	18.5	19.0	18.0
2022-07-01 6:00 PM	16.7	20.0	19.6	18.8
2022-07-01 7:00 PM	16.5	19.0	19.5	18.3
2022-07-01 8:00 PM	16.1	17.1	17.3	16.8
2022-07-01 9:00 PM	15.8	16.7	16.6	16.4
2022-07-01 10:00 PM	15.5	16.2	15.9	15.9
2022-07-01 11:00 PM	15.0	15.7	15.1	15.3
2022-07-02 12:00 AM	14.5	15.1	14.2	14.6
2022-07-02 1:00 AM	14.1	14.7	13.7	14.2
2022-07-02 2:00 AM	13.5	14.0	13.0	13.5
2022-07-02 3:00 AM	12.9	13.3	12.2	12.8
2022-07-02 4:00 AM	12.5	12.9	11.7	12.4
2022-07-02 5:00 AM	12.1	12.5	11.2	11.9
2022-07-02 6:00 AM	11.9	12.4	10.9	11.7
2022-07-02 7:00 AM	11.6	12.0	10.6	11.4
2022-07-02 8:00 AM	11.9	12.3	10.9	11.7
2022-07-02 9:00 AM	12.9	13.2	12.0	12.7
2022-07-02 10:00 AM	14.7	14.1	13.3	14.0
2022-07-02 11:00 AM	15.7	15.5	15.6	15.6
2022-07-02 12:00 PM	17.5	16.7	18.2	17.5
2022-07-02 1:00 PM	17.3	18.0	20.8	18.7
2022-07-02 2:00 PM	16.7	18.1	19.7	18.2
2022-07-02 3:00 PM	17.0	17.9	18.5	17.8

2022-07-02 4:00 PM	16.9	17.5	17.9	17.4
2022-07-02 5:00 PM	16.6	17.3	17.5	17.1
2022-07-02 6:00 PM	16.6	19.7	18.9	18.4
2022-07-02 7:00 PM	16.5	19.3	19.6	18.5
2022-07-02 8:00 PM	16.0	16.8	16.7	16.5
2022-07-02 9:00 PM	15.7	16.3	15.9	16.0
2022-07-02 10:00 PM	15.3	15.9	15.3	15.5
2022-07-02 11:00 PM	14.8	15.3	14.4	14.8
2022-07-03 12:00 AM	14.3	14.8	13.7	14.3
2022-07-03 1:00 AM	14.0	14.4	13.3	13.9
2022-07-03 2:00 AM	13.3	13.7	12.5	13.2
2022-07-03 3:00 AM	12.9	13.3	12.1	12.8
2022-07-03 4:00 AM	12.5	12.8	11.7	12.3
2022-07-03 5:00 AM	12.3	12.5	11.3	12.0
2022-07-03 6:00 AM	12.1	12.3	11.1	11.8
2022-07-03 7:00 AM	12.4	12.6	11.6	12.2
2022-07-03 8:00 AM	13.1	13.3	12.3	12.9
2022-07-03 9:00 AM	14.1	14.1	13.3	13.8
2022-07-03 10:00 AM	15.2	14.9	14.6	14.9
2022-07-03 11:00 AM	16.1	16.1	16.4	16.2
2022-07-03 12:00 PM	17.4	17.0	18.6	17.7
2022-07-03 1:00 PM	17.5	18.1	21.6	19.1
2022-07-03 2:00 PM	16.4	17.8	19.6	17.9
2022-07-03 3:00 PM	16.3	17.1	17.6	17.0
2022-07-03 4:00 PM	16.7	21.7	21.5	20.0
2022-07-03 5:00 PM	16.5	18.4	18.2	17.7
2022-07-03 6:00 PM	16.3	17.2	17.1	16.9
2022-07-03 7:00 PM	16.2	16.8	16.8	16.6
2022-07-03 8:00 PM	16.1	16.6	16.4	16.4
2022-07-03 9:00 PM	15.9	16.3	16.1	16.1
2022-07-03 10:00 PM	15.2	15.4	15.1	15.2
2022-07-03 11:00 PM	15.0	15.1	14.6	14.9
2022-07-04 12:00 AM	14.3	14.5	13.9	14.2
2022-07-04 1:00 AM	13.8	14.0	13.2	13.7
2022-07-04 2:00 AM	13.5	13.6	12.8	13.3
2022-07-04 3:00 AM	13.2	13.3	12.4	13.0
2022-07-04 4:00 AM	12.9	13.0	12.1	12.7
2022-07-04 5:00 AM	12.8	12.8	11.9	12.5
2022-07-04 6:00 AM	12.6	12.7	11.8	12.4
2022-07-04 7:00 AM	12.8	12.7	11.9	12.5
2022-07-04 8:00 AM	13.5	13.6	12.7	13.3
2022-07-04 9:00 AM	14.1	14.0	13.3	13.8
2022-07-04 10:00 AM	14.6	14.1	13.8	14.2

2022-07-04 11:00 AM	14.8	14.4	14.3	14.5
2022-07-04 12:00 PM	14.7	14.6	14.5	14.6
2022-07-04 1:00 PM	15.4	15.6	16.0	15.7
2022-07-04 2:00 PM	15.4	15.7	16.0	15.7
2022-07-04 3:00 PM	14.9	15.1	15.1	15.0
2022-07-04 4:00 PM	14.6	14.6	14.4	14.5
2022-07-04 5:00 PM	14.9	14.9	14.7	14.8
2022-07-04 6:00 PM	15.1	15.1	15.1	15.1
2022-07-04 7:00 PM	14.9	14.9	14.8	14.9
2022-07-04 8:00 PM	14.8	14.7	14.5	14.7
2022-07-04 9:00 PM	14.4	14.4	14.0	14.3
2022-07-04 10:00 PM	14.4	14.4	13.9	14.2
2022-07-04 11:00 PM	14.5	14.4	14.0	14.3
2022-07-05 12:00 AM	14.3	14.3	13.8	14.1
2022-07-05 1:00 AM	14.4	14.4	13.9	14.2
2022-07-05 2:00 AM	14.6	14.6	14.2	14.5
2022-07-05 3:00 AM	14.6	14.7	14.3	14.5
2022-07-05 4:00 AM	14.6	14.7	14.3	14.5
2022-07-05 5:00 AM	14.6	14.7	14.3	14.5
2022-07-05 6:00 AM	14.6	14.7	14.3	14.5
2022-07-05 7:00 AM	14.6	14.7	14.3	14.5
2022-07-05 8:00 AM	14.6	14.7	14.3	14.5
2022-07-05 9:00 AM	14.5	14.6	14.2	14.4
2022-07-05 10:00 AM	14.7	14.7	14.3	14.6
2022-07-05 11:00 AM	14.8	14.9	14.6	14.8
2022-07-05 12:00 PM	14.9	15.0	14.8	14.9
2022-07-05 1:00 PM	15.2	15.5	15.5	15.4
2022-07-05 2:00 PM	15.7	16.2	16.6	16.2
2022-07-05 3:00 PM	15.9	16.6	17.0	16.5
2022-07-05 4:00 PM	16.3	17.4	17.7	17.1
2022-07-05 5:00 PM	16.6	18.1	18.4	17.7
2022-07-05 6:00 PM	16.7	18.0	18.4	17.7
2022-07-05 7:00 PM	16.5	18.1	18.6	17.7
2022-07-05 8:00 PM	16.1	16.9	17.0	16.7
2022-07-05 9:00 PM	16.0	16.6	16.6	16.4
2022-07-05 10:00 PM	15.8	16.3	16.1	16.1
2022-07-05 11:00 PM	15.4	15.8	15.3	15.5
2022-07-06 12:00 AM	14.9	15.3	14.5	14.9
2022-07-06 1:00 AM	14.4	14.7	13.8	14.3
2022-07-06 2:00 AM	14.1	14.4	13.4	14.0
2022-07-06 3:00 AM	13.8	14.1	13.1	13.7
2022-07-06 4:00 AM	13.4	13.7	12.7	13.3
2022-07-06 5:00 AM	13.1	13.3	12.4	12.9

2022-07-06 6:00 AM	12.8	13.1	12.1	12.7
2022-07-06 7:00 AM	12.6	12.8	11.7	12.4
2022-07-06 8:00 AM	12.9	13.1	12.0	12.7
2022-07-06 9:00 AM	13.8	14.1	13.1	13.7
2022-07-06 10:00 AM	15.1	14.9	14.6	14.9
2022-07-06 11:00 AM	16.2	16.1	16.5	16.3
2022-07-06 12:00 PM	17.8	16.9	18.6	17.8
2022-07-06 1:00 PM	17.6	17.3	20.3	18.4
2022-07-06 2:00 PM	16.3	17.2	18.7	17.4
2022-07-06 3:00 PM	16.2	16.6	17.0	16.6
2022-07-06 4:00 PM	16.5	21.2	21.3	19.7
2022-07-06 5:00 PM	15.9	17.5	17.4	16.9
2022-07-06 6:00 PM	15.8	17.5	16.8	16.7
2022-07-06 7:00 PM	15.9	18.0	17.8	17.2
2022-07-06 8:00 PM	15.6	16.3	16.0	16.0
2022-07-06 9:00 PM	15.5	16.0	15.6	15.7
2022-07-06 10:00 PM	15.1	15.6	15.0	15.2
2022-07-06 11:00 PM	14.4	14.8	13.9	14.4
2022-07-07 12:00 AM	13.9	14.1	13.3	13.8
2022-07-07 1:00 AM	13.6	13.8	12.8	13.4
2022-07-07 2:00 AM	13.3	13.4	12.4	13.0
2022-07-07 3:00 AM	13.0	13.1	12.1	12.7
2022-07-07 4:00 AM	12.6	12.7	11.7	12.3
2022-07-07 5:00 AM	12.2	12.5	11.3	12.0
2022-07-07 6:00 AM	12.2	12.3	11.2	11.9
2022-07-07 7:00 AM	12.5	12.6	11.5	12.2
2022-07-07 8:00 AM	13.0	13.1	12.1	12.7
2022-07-07 9:00 AM	13.7	13.6	12.9	13.4
2022-07-07 10:00 AM	14.7	14.5	14.1	14.4
2022-07-07 11:00 AM	15.8	15.7	16.0	15.8
2022-07-07 12:00 PM	17.6	17.1	18.7	17.8
2022-07-07 1:00 PM	18.0	18.4	21.2	19.2
2022-07-07 2:00 PM	17.3	18.9	20.2	18.8
2022-07-07 3:00 PM	17.5	18.6	19.9	18.7
2022-07-07 4:00 PM	17.5	21.4	22.4	20.4
2022-07-07 5:00 PM	17.0	18.8	19.3	18.4
2022-07-07 6:00 PM	17.1	18.3	18.4	17.9
2022-07-07 7:00 PM	17.2	18.4	18.8	18.1
2022-07-07 8:00 PM	16.9	17.6	17.8	17.4
2022-07-07 9:00 PM	16.7	17.3	17.5	17.2
2022-07-07 10:00 PM	16.5	17.0	16.9	16.8
2022-07-07 11:00 PM	16.2	16.6	16.3	16.4
2022-07-08 12:00 AM	16.0	16.3	15.8	16.0

2022-07-08 1:00 AM	15.7	16.0	15.4	15.7
2022-07-08 2:00 AM	15.2	15.7	14.8	15.2
2022-07-08 3:00 AM	14.8	15.2	14.2	14.7
2022-07-08 4:00 AM	14.3	14.5	13.5	14.1
2022-07-08 5:00 AM	13.9	14.1	13.0	13.7
2022-07-08 6:00 AM	13.3	13.5	12.3	13.0
2022-07-08 7:00 AM	12.9	13.1	12.0	12.7
2022-07-08 8:00 AM	13.3	13.6	12.4	13.1
2022-07-08 9:00 AM	14.4	14.6	13.6	14.2
2022-07-08 10:00 AM	15.7	15.6	15.2	15.5
2022-07-08 11:00 AM	16.9	16.8	17.2	17.0
2022-07-08 12:00 PM	18.3	17.8	19.3	18.5
2022-07-08 1:00 PM	18.9	18.7	21.5	19.7
2022-07-08 2:00 PM	17.5	18.4	19.6	18.5
2022-07-08 3:00 PM	17.3	18.0	18.7	18.0
2022-07-08 4:00 PM	17.6	22.5	22.7	20.9
2022-07-08 5:00 PM	17.9	19.3	19.0	18.7
2022-07-08 6:00 PM	19.0	18.7	17.6	18.4
2022-07-08 7:00 PM	17.8	18.7	19.1	18.5
2022-07-08 8:00 PM	17.3	17.6	17.5	17.5
2022-07-08 9:00 PM	17.0	17.3	17.0	17.1
2022-07-08 10:00 PM	16.4	16.9	16.3	16.5
2022-07-08 11:00 PM	15.8	16.4	15.5	15.9
2022-07-09 12:00 AM	15.4	15.8	15.0	15.4
2022-07-09 1:00 AM	15.0	15.4	14.3	14.9
2022-07-09 2:00 AM	14.7	15.1	13.9	14.6
2022-07-09 3:00 AM	14.4	14.7	13.4	14.2
2022-07-09 4:00 AM	14.0	14.2	13.0	13.7
2022-07-09 5:00 AM	13.6	13.8	12.5	13.3
2022-07-09 6:00 AM	13.4	13.6	12.3	13.1
2022-07-09 7:00 AM	13.1	13.2	12.0	12.8
2022-07-09 8:00 AM	13.5	13.6	12.4	13.2
2022-07-09 9:00 AM	14.7	14.9	13.8	14.5
2022-07-09 10:00 AM	15.8	15.8	15.4	15.7
2022-07-09 11:00 AM	17.0	17.1	17.5	17.2
2022-07-09 12:00 PM	19.1	18.2	19.8	19.0
2022-07-09 1:00 PM	19.1	18.9	22.1	20.0
2022-07-09 2:00 PM	17.8	18.8	21.2	19.3
2022-07-09 3:00 PM	17.6	18.3	19.7	18.5
2022-07-09 4:00 PM	17.8	21.3	22.2	20.4
2022-07-09 5:00 PM	17.6	19.2	19.9	18.9
2022-07-09 6:00 PM	17.7	19.4	19.7	18.9
2022-07-09 7:00 PM	17.6	19.6	20.3	19.2

2022-07-09 8:00 PM	17.1	18.0	18.0	17.7
2022-07-09 9:00 PM	16.9	17.6	17.4	17.3
2022-07-09 10:00 PM	16.5	17.1	16.7	16.8
2022-07-09 11:00 PM	15.7	16.2	15.4	15.8
2022-07-10 12:00 AM	15.3	15.6	14.8	15.2
2022-07-10 1:00 AM	14.8	15.0	14.1	14.6
2022-07-10 2:00 AM	14.5	14.8	13.6	14.3
2022-07-10 3:00 AM	14.7	14.6	13.6	14.3
2022-07-10 4:00 AM	14.7	14.8	13.9	14.5
2022-07-10 5:00 AM	14.8	14.4	13.6	14.3
2022-07-10 6:00 AM	14.5	14.5	13.4	14.1
2022-07-10 7:00 AM	14.7	14.5	13.4	14.2
2022-07-10 8:00 AM	15.2	15.2	14.4	14.9
2022-07-10 9:00 AM	15.5	15.6	15.1	15.4
2022-07-10 10:00 AM	16.3	16.1	15.9	16.1
2022-07-10 11:00 AM	17.0	16.9	17.1	17.0
2022-07-10 12:00 PM	17.6	17.5	18.0	17.7
2022-07-10 1:00 PM	18.0	18.1	18.9	18.3
2022-07-10 2:00 PM	17.9	18.7	19.7	18.8
2022-07-10 3:00 PM	18.1	18.7	19.8	18.9
2022-07-10 4:00 PM	18.0	18.2	18.7	18.3
2022-07-10 5:00 PM	17.3	17.4	17.5	17.4
2022-07-10 6:00 PM	17.5	17.8	18.0	17.8
2022-07-10 7:00 PM	17.5	17.8	18.0	17.8
2022-07-10 8:00 PM	17.6	18.0	18.2	17.9
2022-07-10 9:00 PM	17.5	17.8	17.9	17.7
2022-07-10 10:00 PM	17.2	17.5	17.4	17.4
2022-07-10 11:00 PM	17.0	17.4	17.1	17.2
2022-07-11 12:00 AM	16.8	17.1	16.6	16.8
2022-07-11 1:00 AM	16.7	17.0	16.4	16.7
2022-07-11 2:00 AM	16.5	16.8	16.2	16.5
2022-07-11 3:00 AM	16.5	16.8	16.2	16.5
2022-07-11 4:00 AM	16.8	17.1	16.6	16.8
2022-07-11 5:00 AM	16.6	16.9	16.4	16.6
2022-07-11 6:00 AM	16.5	16.9	16.3	16.6
2022-07-11 7:00 AM	16.3	16.7	16.0	16.3
2022-07-11 8:00 AM	16.4	16.8	16.1	16.4
2022-07-11 9:00 AM	16.8	17.3	16.9	17.0
2022-07-11 10:00 AM	17.7	17.9	17.8	17.8
2022-07-11 11:00 AM	18.7	18.8	19.1	18.9
2022-07-11 12:00 PM	20.3	19.7	20.8	20.3
2022-07-11 1:00 PM	19.8	20.0	21.2	20.3
2022-07-11 2:00 PM	19.7	20.3	21.2	20.4

2022-07-11 3:00 PM	19.8	20.5	21.4	20.6
2022-07-11 4:00 PM	19.8	20.4	21.2	20.5
2022-07-11 5:00 PM	19.6	20.1	20.6	20.1
2022-07-11 6:00 PM	19.6	20.1	20.6	20.1
2022-07-11 7:00 PM	19.2	19.6	19.9	19.6
2022-07-11 8:00 PM	19.2	19.7	20.0	19.6
2022-07-11 9:00 PM	18.6	18.5	19.0	18.7
2022-07-11 10:00 PM	18.1	17.9	17.7	17.9
2022-07-11 11:00 PM	17.8	17.7	17.3	17.6
2022-07-12 12:00 AM	17.5	17.5	16.9	17.3
2022-07-12 1:00 AM	17.4	17.4	16.7	17.2
2022-07-12 2:00 AM	17.5	17.6	16.9	17.3
2022-07-12 3:00 AM	17.5	17.6	17.0	17.4
2022-07-12 4:00 AM	17.3	17.5	16.8	17.2
2022-07-12 5:00 AM	17.3	17.4	16.7	17.1
2022-07-12 6:00 AM	17.2	17.2	16.6	17.0
2022-07-12 7:00 AM	17.1	17.2	16.6	17.0
2022-07-12 8:00 AM	16.9	17.0	16.4	16.8
2022-07-12 9:00 AM	16.9	16.8	16.2	16.6
2022-07-12 10:00 AM	16.8	16.6	16.1	16.5
2022-07-12 11:00 AM	16.5	16.2	15.6	16.1
2022-07-12 12:00 PM	16.5	16.2	15.6	16.1
2022-07-12 1:00 PM	16.2	15.9	16.0	16.0
2022-07-12 2:00 PM	17.2	16.8	16.6	16.9
2022-07-12 3:00 PM	17.9	17.6	17.9	17.8
2022-07-12 4:00 PM	17.8	17.4	17.7	17.6
2022-07-12 5:00 PM	17.6	17.1	17.3	17.3
2022-07-12 6:00 PM	17.6	17.4	17.6	17.5
2022-07-12 7:00 PM	17.3	17.0	17.1	17.1
2022-07-12 8:00 PM	17.1	16.8	16.8	16.9
2022-07-12 9:00 PM	16.8	16.6	16.4	16.6
2022-07-12 10:00 PM	16.2	16.2	15.8	16.1
2022-07-12 11:00 PM	15.5	15.6	14.8	15.3
2022-07-13 12:00 AM	15.2	15.4	14.3	15.0
2022-07-13 1:00 AM	14.6	14.9	13.7	14.4
2022-07-13 2:00 AM	14.1	14.3	13.0	13.8
2022-07-13 3:00 AM	13.7	13.9	12.5	13.4
2022-07-13 4:00 AM	13.2	13.4	12.0	12.9
2022-07-13 5:00 AM	12.8	13.0	11.5	12.4
2022-07-13 6:00 AM	12.6	12.8	11.3	12.2
2022-07-13 7:00 AM	12.5	12.6	11.2	12.1
2022-07-13 8:00 AM	12.9	13.1	11.8	12.6
2022-07-13 9:00 AM	14.0	14.3	13.1	13.8

2022-07-13 10:00 AM	15.3	15.1	14.6	15.0
2022-07-13 11:00 AM	16.5	16.5	16.8	16.6
2022-07-13 12:00 PM	18.6	17.4	19.0	18.3
2022-07-13 1:00 PM	18.3	17.9	20.4	18.9
2022-07-13 2:00 PM	17.5	18.4	20.4	18.8
2022-07-13 3:00 PM	17.6	18.1	19.0	18.2
2022-07-13 4:00 PM	17.6	20.9	21.2	19.9
2022-07-13 5:00 PM	17.1	18.3	18.7	18.0
2022-07-13 6:00 PM	16.9	18.2	18.1	17.7
2022-07-13 7:00 PM	16.9	18.7	19.2	18.3
2022-07-13 8:00 PM	16.6	17.2	17.1	17.0
2022-07-13 9:00 PM	16.5	16.9	16.6	16.7
2022-07-13 10:00 PM	16.1	16.5	16.0	16.2
2022-07-13 11:00 PM	15.4	15.8	15.0	15.4
2022-07-14 12:00 AM	15.0	15.3	14.5	14.9
2022-07-14 1:00 AM	14.7	14.9	13.9	14.5
2022-07-14 2:00 AM	14.4	14.6	13.4	14.1
2022-07-14 3:00 AM	14.1	14.3	13.0	13.8
2022-07-14 4:00 AM	13.6	13.8	12.4	13.3
2022-07-14 5:00 AM	13.0	13.3	11.7	12.7
2022-07-14 6:00 AM	12.6	12.8	11.0	12.1
2022-07-14 7:00 AM	12.3	12.5	10.7	11.8
2022-07-14 8:00 AM	12.8	13.1	11.3	12.4
2022-07-14 9:00 AM	14.2	14.3	13.0	13.8
2022-07-14 10:00 AM	15.9	15.5	15.0	15.5
2022-07-14 11:00 AM	17.2	16.0	16.2	16.5
2022-07-14 12:00 PM	20.8	17.3	19.3	19.1
2022-07-14 1:00 PM	19.6	18.6	22.7	20.3
2022-07-14 2:00 PM	17.9	19.3	21.3	19.5
2022-07-14 3:00 PM	17.6	19.4	19.3	18.8
2022-07-14 4:00 PM	17.8	27.0	23.3	22.7
2022-07-14 5:00 PM	17.4	20.6	19.5	19.2
2022-07-14 6:00 PM	17.4	21.1	19.5	19.3
2022-07-14 7:00 PM	17.4	22.0	21.0	20.1
2022-07-14 8:00 PM	16.9	19.2	17.9	18.0
2022-07-14 9:00 PM	16.5	17.9	17.0	17.1
2022-07-14 10:00 PM	16.1	16.7	16.0	16.3
2022-07-14 11:00 PM	15.8	16.2	15.3	15.8
2022-07-15 12:00 AM	15.3	15.7	14.7	15.2
2022-07-15 1:00 AM	15.1	15.4	14.4	15.0
2022-07-15 2:00 AM	14.9	15.1	14.1	14.7
2022-07-15 3:00 AM	14.8	15.1	14.0	14.6
2022-07-15 4:00 AM	14.7	15.0	13.9	14.5

2022-07-15 5:00 AM	14.9	15.1	14.1	14.7
2022-07-15 6:00 AM	14.8	14.9	14.1	14.6
2022-07-15 7:00 AM	14.6	14.7	13.8	14.4
2022-07-15 8:00 AM	15.1	15.1	14.1	14.8
2022-07-15 9:00 AM	15.9	15.7	15.2	15.6
2022-07-15 10:00 AM	16.8	16.2	15.7	16.2
2022-07-15 11:00 AM	18.2	17.2	17.3	17.6
2022-07-15 12:00 PM	22.1	18.1	19.8	20.0
2022-07-15 1:00 PM	20.3	19.0	22.6	20.6
2022-07-15 2:00 PM	18.1	18.7	20.5	19.1
2022-07-15 3:00 PM	17.7	18.7	18.8	18.4
2022-07-15 4:00 PM	17.9	26.4	23.1	22.5
2022-07-15 5:00 PM	17.7	19.9	19.4	19.0
2022-07-15 6:00 PM	17.6	20.4	19.3	19.1
2022-07-15 7:00 PM	18.1	22.7	22.2	21.0
2022-07-15 8:00 PM	17.8	19.3	19.2	18.8
2022-07-15 9:00 PM	17.5	18.7	18.3	18.2
2022-07-15 10:00 PM	17.3	18.1	17.6	17.7
2022-07-15 11:00 PM	16.8	17.2	16.7	16.9
2022-07-16 12:00 AM	16.4	16.5	15.9	16.3
2022-07-16 1:00 AM	16.0	16.1	15.3	15.8
2022-07-16 2:00 AM	15.9	16.1	15.2	15.7
2022-07-16 3:00 AM	15.6	16.0	14.8	15.5
2022-07-16 4:00 AM	15.3	15.7	14.5	15.2
2022-07-16 5:00 AM	15.1	15.4	14.2	14.9
2022-07-16 6:00 AM	15.2	15.4	14.2	14.9
2022-07-16 7:00 AM	15.0	15.3	14.1	14.8
2022-07-16 8:00 AM	15.3	15.6	14.4	15.1
2022-07-16 9:00 AM	16.2	16.6	15.8	16.2
2022-07-16 10:00 AM	17.7	17.6	17.4	17.6
2022-07-16 11:00 AM	19.6	19.0	19.4	19.3
2022-07-16 12:00 PM	23.1	19.6	21.4	21.4
2022-07-16 1:00 PM	21.1	19.7	22.9	21.2
2022-07-16 2:00 PM	19.0	19.7	21.2	20.0
2022-07-16 3:00 PM	19.2	19.7	20.0	19.6
2022-07-16 4:00 PM	19.2	27.3	23.9	23.5
2022-07-16 5:00 PM	19.1	21.7	21.2	20.7
2022-07-16 6:00 PM	19.2	22.0	21.4	20.9
2022-07-16 7:00 PM	19.1	23.1	22.9	21.7
2022-07-16 8:00 PM	18.8	20.0	19.9	19.6
2022-07-16 9:00 PM	18.4	19.2	18.8	18.8
2022-07-16 10:00 PM	17.8	18.5	18.0	18.1
2022-07-16 11:00 PM	17.3	17.9	17.0	17.4

2022-07-17 12:00 AM	17.0	17.5	16.6	17.0
2022-07-17 1:00 AM	16.7	17.0	16.1	16.6
2022-07-17 2:00 AM	16.4	16.7	15.6	16.2
2022-07-17 3:00 AM	16.1	16.3	15.3	15.9
2022-07-17 4:00 AM	15.8	16.1	14.9	15.6
2022-07-17 5:00 AM	15.4	15.6	14.4	15.1
2022-07-17 6:00 AM	15.3	15.5	14.4	15.1
2022-07-17 7:00 AM	15.2	15.3	14.3	14.9
2022-07-17 8:00 AM	15.5	15.7	14.5	15.2
2022-07-17 9:00 AM	16.3	16.6	15.6	16.2
2022-07-17 10:00 AM	17.4	17.1	16.5	17.0
2022-07-17 11:00 AM	18.8	17.2	17.4	17.8
2022-07-17 12:00 PM	21.4	17.6	19.3	19.4
2022-07-17 1:00 PM	20.5	18.8	22.4	20.6
2022-07-17 2:00 PM	18.9	19.4	21.6	20.0
2022-07-17 3:00 PM	18.6	19.2	19.7	19.2
2022-07-17 4:00 PM	18.7	25.6	23.1	22.5
2022-07-17 5:00 PM	18.0	19.5	19.2	18.9
2022-07-17 6:00 PM	18.0	19.8	19.1	19.0
2022-07-17 7:00 PM	17.9	21.0	20.8	19.9
2022-07-17 8:00 PM	17.4	17.9	17.6	17.6
2022-07-17 9:00 PM	17.1	17.3	16.8	17.1
2022-07-17 10:00 PM	16.5	16.6	16.0	16.4
2022-07-17 11:00 PM	16.2	16.3	15.5	16.0
2022-07-18 12:00 AM	16.2	16.2	15.5	16.0
2022-07-18 1:00 AM	16.2	16.1	15.4	15.9
2022-07-18 2:00 AM	16.0	16.0	15.2	15.7
2022-07-18 3:00 AM	15.9	16.0	15.2	15.7
2022-07-18 4:00 AM	15.8	16.1	15.1	15.7
2022-07-18 5:00 AM	16.0	16.2	15.3	15.8
2022-07-18 6:00 AM	16.1	16.3	15.5	16.0
2022-07-18 7:00 AM	16.3	16.5	15.8	16.2
2022-07-18 8:00 AM	16.2	16.6	15.7	16.2
2022-07-18 9:00 AM	16.9	17.3	16.9	17.0
2022-07-18 10:00 AM	18.1	18.3	18.2	18.2
2022-07-18 11:00 AM	20.0	20.0	20.6	20.2
2022-07-18 12:00 PM	24.4	21.6	23.9	23.3
2022-07-18 1:00 PM	22.8	22.9	27.1	24.3
2022-07-18 2:00 PM	21.0	23.3	25.5	23.3
2022-07-18 3:00 PM	21.6	23.6	24.6	23.3
2022-07-18 4:00 PM	21.9	28.7	27.7	26.1
2022-07-18 5:00 PM	21.3	24.6	24.7	23.5
2022-07-18 6:00 PM	21.3	25.0	25.0	23.8

2022-07-18 7:00 PM	21.3	26.7	26.8	24.9
2022-07-18 8:00 PM	21.0	23.0	23.4	22.5
2022-07-18 9:00 PM	20.9	22.3	22.7	22.0
2022-07-18 10:00 PM	20.5	21.6	21.6	21.2
2022-07-18 11:00 PM	20.1	21.1	20.8	20.7
2022-07-19 12:00 AM	19.8	20.6	20.1	20.2
2022-07-19 1:00 AM	19.8	20.6	20.0	20.1
2022-07-19 2:00 AM	19.8	20.5	20.0	20.1
2022-07-19 3:00 AM	19.6	20.3	19.8	19.9
2022-07-19 4:00 AM	19.6	20.3	19.7	19.9
2022-07-19 5:00 AM	19.4	20.1	19.5	19.7
2022-07-19 6:00 AM	19.2	19.9	19.2	19.4
2022-07-19 7:00 AM	19.2	19.8	19.0	19.3
2022-07-19 8:00 AM	19.1	19.7	19.0	19.3
2022-07-19 9:00 AM	19.1	19.6	19.0	19.2
2022-07-19 10:00 AM	19.1	19.5	19.0	19.2
2022-07-19 11:00 AM	19.2	19.4	18.9	19.2
2022-07-19 12:00 PM	18.8	18.6	18.2	18.5
2022-07-19 1:00 PM	18.2	17.7	17.3	17.7
2022-07-19 2:00 PM	18.6	18.4	17.9	18.3
2022-07-19 3:00 PM	18.7	18.5	18.1	18.4
2022-07-19 4:00 PM	18.3	17.7	17.4	17.8
2022-07-19 5:00 PM	18.1	17.5	17.2	17.6
2022-07-19 6:00 PM	18.8	18.4	18.1	18.4
2022-07-19 7:00 PM	18.8	18.4	18.3	18.5
2022-07-19 8:00 PM	18.5	18.3	18.1	18.3
2022-07-19 9:00 PM	18.1	17.8	17.5	17.8
2022-07-19 10:00 PM	17.7	17.5	17.1	17.4
2022-07-19 11:00 PM	17.5	17.1	16.6	17.1
2022-07-20 12:00 AM	17.4	17.0	16.5	17.0
2022-07-20 1:00 AM	17.4	17.4	16.7	17.2
2022-07-20 2:00 AM	17.3	17.3	16.7	17.1
2022-07-20 3:00 AM	17.1	17.2	16.4	16.9
2022-07-20 4:00 AM	16.9	17.0	16.2	16.7
2022-07-20 5:00 AM	16.5	16.6	15.7	16.3
2022-07-20 6:00 AM	16.2	16.3	15.3	15.9
2022-07-20 7:00 AM	15.8	15.9	15.0	15.6
2022-07-20 8:00 AM	16.1	16.1	15.2	15.8
2022-07-20 9:00 AM	17.0	16.9	16.2	16.7
2022-07-20 10:00 AM	17.9	17.6	17.3	17.6
2022-07-20 11:00 AM	19.5	19.1	19.3	19.3
2022-07-20 12:00 PM	21.0	19.9	20.8	20.6
2022-07-20 1:00 PM	20.6	20.4	21.4	20.8

2022-07-20 2:00 PM	20.4	20.5	21.3	20.7
2022-07-20 3:00 PM	19.9	20.2	20.7	20.3
2022-07-20 4:00 PM	20.1	20.6	20.8	20.5
2022-07-20 5:00 PM	19.9	20.3	20.4	20.2
2022-07-20 6:00 PM	20.2	20.5	20.7	20.5
2022-07-20 7:00 PM	20.2	20.8	20.9	20.6
2022-07-20 8:00 PM	20.1	20.5	20.8	20.5
2022-07-20 9:00 PM	19.9	20.3	20.5	20.2
2022-07-20 10:00 PM	19.8	20.1	20.2	20.0
2022-07-20 11:00 PM	19.1	19.5	19.1	19.2
2022-07-21 12:00 AM	18.6	19.0	18.3	18.6
2022-07-21 1:00 AM	18.3	18.7	17.9	18.3
2022-07-21 2:00 AM	18.0	18.3	17.4	17.9
2022-07-21 3:00 AM	17.8	18.1	17.1	17.7
2022-07-21 4:00 AM	17.9	18.1	17.3	17.8
2022-07-21 5:00 AM	17.9	18.2	17.3	17.8
2022-07-21 6:00 AM	17.8	18.1	17.3	17.7
2022-07-21 7:00 AM	17.6	17.9	17.0	17.5
2022-07-21 8:00 AM	17.7	17.9	16.9	17.5
2022-07-21 9:00 AM	18.1	18.2	17.5	17.9
2022-07-21 10:00 AM	18.9	18.8	18.3	18.7
2022-07-21 11:00 AM	20.4	19.8	20.1	20.1
2022-07-21 12:00 PM	24.2	20.8	23.0	22.7
2022-07-21 1:00 PM	22.2	21.5	24.1	22.6
2022-07-21 2:00 PM	20.9	21.4	22.6	21.6
2022-07-21 3:00 PM	20.7	22.0	22.7	21.8
2022-07-21 4:00 PM	20.8	29.5	25.8	25.4
2022-07-21 5:00 PM	20.4	22.7	22.3	21.8
2022-07-21 6:00 PM	20.3	21.9	21.6	21.3
2022-07-21 7:00 PM	19.9	22.7	21.6	21.4
2022-07-21 8:00 PM	19.5	20.5	20.0	20.0
2022-07-21 9:00 PM	19.4	20.1	19.5	19.7
2022-07-21 10:00 PM	19.0	19.7	18.9	19.2
2022-07-21 11:00 PM	18.3	18.9	17.9	18.4
2022-07-22 12:00 AM	17.8	18.1	17.1	17.7
2022-07-22 1:00 AM	17.4	17.7	16.6	17.2
2022-07-22 2:00 AM	17.1	17.4	16.2	16.9
2022-07-22 3:00 AM	16.9	17.1	15.9	16.6
2022-07-22 4:00 AM	16.6	16.8	15.6	16.3
2022-07-22 5:00 AM	16.3	16.5	15.2	16.0
2022-07-22 6:00 AM	16.0	16.2	14.9	15.7
2022-07-22 7:00 AM	15.9	16.1	14.8	15.6
2022-07-22 8:00 AM	16.1	16.4	15.0	15.8

2022-07-22 9:00 AM	17.1	17.4	16.3	16.9
2022-07-22 10:00 AM	18.2	18.3	17.8	18.1
2022-07-22 11:00 AM	19.8	19.7	19.9	19.8
2022-07-22 12:00 PM	23.5	20.8	22.6	22.3
2022-07-22 1:00 PM	22.3	21.8	25.2	23.1
2022-07-22 2:00 PM	20.7	21.7	23.1	21.8
2022-07-22 3:00 PM	20.7	21.9	22.1	21.6
2022-07-22 4:00 PM	21.0	25.3	23.9	23.4
2022-07-22 5:00 PM	20.6	21.9	22.2	21.6
2022-07-22 6:00 PM	20.7	22.1	22.6	21.8
2022-07-22 7:00 PM	20.6	21.8	22.2	21.5
2022-07-22 8:00 PM	20.2	21.2	21.2	20.9
2022-07-22 9:00 PM	20.1	20.9	20.8	20.6
2022-07-22 10:00 PM	19.6	20.4	19.8	19.9
2022-07-22 11:00 PM	19.1	19.8	18.7	19.2
2022-07-23 12:00 AM	18.7	19.3	18.2	18.7
2022-07-23 1:00 AM	18.3	18.8	17.5	18.2
2022-07-23 2:00 AM	18.1	18.5	17.3	18.0
2022-07-23 3:00 AM	17.6	18.0	16.7	17.4
2022-07-23 4:00 AM	17.2	17.6	16.1	17.0
2022-07-23 5:00 AM	17.0	17.3	15.9	16.7
2022-07-23 6:00 AM	16.8	17.2	15.8	16.6
2022-07-23 7:00 AM	16.7	17.1	15.7	16.5
2022-07-23 8:00 AM	17.1	17.3	16.1	16.8
2022-07-23 9:00 AM	17.9	17.9	17.0	17.6
2022-07-23 10:00 AM	18.6	18.6	18.1	18.4
2022-07-23 11:00 AM	19.8	19.2	19.1	19.4
2022-07-23 12:00 PM	23.3	20.1	21.9	21.8
2022-07-23 1:00 PM	21.7	20.9	23.4	22.0
2022-07-23 2:00 PM	21.0	20.9	21.9	21.3
2022-07-23 3:00 PM	20.8	20.8	21.4	21.0
2022-07-23 4:00 PM	19.8	19.7	19.8	19.8
2022-07-23 5:00 PM	20.2	20.1	20.3	20.2
2022-07-23 6:00 PM	19.7	19.7	19.8	19.7
2022-07-23 7:00 PM	19.1	19.1	18.7	19.0
2022-07-23 8:00 PM	18.8	18.8	18.3	18.6
2022-07-23 9:00 PM	18.7	18.8	18.1	18.5
2022-07-23 10:00 PM	18.6	18.7	18.1	18.5
2022-07-23 11:00 PM	18.5	18.6	17.9	18.3
2022-07-24 12:00 AM	18.1	18.3	17.5	18.0
2022-07-24 1:00 AM	17.7	17.9	16.9	17.5
2022-07-24 2:00 AM	18.0	18.2	17.3	17.8
2022-07-24 3:00 AM	17.9	18.0	17.3	17.7

2022-07-24 4:00 AM	17.8	17.9	17.1	17.6
2022-07-24 5:00 AM	17.6	17.7	16.9	17.4
2022-07-24 6:00 AM	17.5	17.7	16.8	17.3
2022-07-24 7:00 AM	17.4	17.5	16.6	17.2
2022-07-24 8:00 AM	17.3	17.3	16.4	17.0
2022-07-24 9:00 AM	17.6	17.8	16.9	17.4
2022-07-24 10:00 AM	18.3	18.1	17.5	18.0
2022-07-24 11:00 AM	19.4	18.8	18.7	19.0
2022-07-24 12:00 PM	19.3	18.3	18.4	18.7
2022-07-24 1:00 PM	19.2	18.3	18.3	18.6
2022-07-24 2:00 PM	19.3	18.1	18.3	18.6
2022-07-24 3:00 PM	18.7	17.3	17.5	17.8
2022-07-24 4:00 PM	18.4	16.9	17.0	17.4
2022-07-24 5:00 PM	18.3	17.4	17.3	17.7
2022-07-24 6:00 PM	18.1	17.6	17.4	17.7
2022-07-24 7:00 PM	17.7	18.3	17.9	18.0
2022-07-24 8:00 PM	17.1	16.9	16.2	16.7
2022-07-24 9:00 PM	16.7	16.5	15.8	16.3
2022-07-24 10:00 PM	16.3	16.0	15.2	15.8
2022-07-24 11:00 PM	16.0	16.0	14.8	15.6
2022-07-25 12:00 AM	16.0	16.0	15.0	15.7
2022-07-25 1:00 AM	16.0	15.9	15.0	15.6
2022-07-25 2:00 AM	16.0	15.6	14.9	15.5
2022-07-25 3:00 AM	15.8	15.6	14.8	15.4
2022-07-25 4:00 AM	15.4	15.4	14.2	15.0
2022-07-25 5:00 AM	15.1	15.1	13.8	14.7
2022-07-25 6:00 AM	14.8	14.9	13.5	14.4
2022-07-25 7:00 AM	14.6	14.8	13.3	14.2
2022-07-25 8:00 AM	14.8	15.0	13.5	14.4
2022-07-25 9:00 AM	15.4	15.6	14.3	15.1
2022-07-25 10:00 AM	16.4	16.3	15.5	16.1
2022-07-25 11:00 AM	17.9	17.4	17.4	17.6
2022-07-25 12:00 PM	21.4	18.4	20.1	20.0
2022-07-25 1:00 PM	20.3	19.6	22.5	20.8
2022-07-25 2:00 PM	18.9	19.7	20.9	19.8
2022-07-25 3:00 PM	18.8	19.8	20.1	19.6
2022-07-25 4:00 PM	19.0	24.8	22.3	22.0
2022-07-25 5:00 PM	18.7	20.3	20.2	19.7
2022-07-25 6:00 PM	18.9	21.1	20.5	20.2
2022-07-25 7:00 PM	18.9	22.5	22.3	21.2
2022-07-25 8:00 PM	18.2	19.3	19.1	18.9
2022-07-25 9:00 PM	18.0	18.8	18.4	18.4
2022-07-25 10:00 PM	17.5	18.2	17.5	17.7

2022-07-25 11:00 PM	17.0	17.6	16.6	17.1
2022-07-26 12:00 AM	16.6	16.9	15.8	16.4
2022-07-26 1:00 AM	16.1	16.3	15.1	15.8
2022-07-26 2:00 AM	15.8	16.0	14.8	15.5
2022-07-26 3:00 AM	15.6	15.7	14.5	15.3
2022-07-26 4:00 AM	15.4	15.5	14.3	15.1
2022-07-26 5:00 AM	15.1	15.2	14.0	14.8
2022-07-26 6:00 AM	15.0	15.2	13.7	14.6
2022-07-26 7:00 AM	14.9	14.9	13.6	14.5
2022-07-26 8:00 AM	15.2	15.4	14.2	14.9
2022-07-26 9:00 AM	16.2	16.2	15.2	15.9
2022-07-26 10:00 AM	17.1	17.0	16.5	16.9
2022-07-26 11:00 AM	18.1	17.8	17.8	17.9
2022-07-26 12:00 PM	19.0	18.1	18.5	18.5
2022-07-26 1:00 PM	18.7	18.6	19.5	18.9
2022-07-26 2:00 PM	18.9	19.8	21.0	19.9
2022-07-26 3:00 PM	19.1	19.8	20.5	19.8
2022-07-26 4:00 PM	19.0	19.5	20.0	19.5
2022-07-26 5:00 PM	19.2	19.8	20.2	19.7
2022-07-26 6:00 PM	19.3	19.8	20.3	19.8
2022-07-26 7:00 PM	19.0	19.5	19.8	19.4
2022-07-26 8:00 PM	18.7	19.1	19.2	19.0
2022-07-26 9:00 PM	18.5	18.8	18.7	18.7
2022-07-26 10:00 PM	18.5	18.8	18.6	18.6
2022-07-26 11:00 PM	18.1	18.4	17.9	18.1
2022-07-27 12:00 AM	17.8	18.1	17.4	17.8
2022-07-27 1:00 AM	18.0	18.2	17.6	17.9
2022-07-27 2:00 AM	17.7	18.0	17.2	17.6
2022-07-27 3:00 AM	17.6	17.9	17.1	17.5
2022-07-27 4:00 AM	17.1	17.5	16.4	17.0
2022-07-27 5:00 AM	16.6	16.9	15.7	16.4
2022-07-27 6:00 AM	16.4	16.7	15.5	16.2
2022-07-27 7:00 AM	16.3	16.5	15.4	16.1
2022-07-27 8:00 AM	16.4	16.6	15.5	16.2
2022-07-27 9:00 AM	17.0	17.3	16.2	16.8
2022-07-27 10:00 AM	17.7	17.8	17.2	17.6
2022-07-27 11:00 AM	19.0	18.5	18.5	18.7
2022-07-27 12:00 PM	22.2	19.3	20.8	20.8
2022-07-27 1:00 PM	21.1	20.2	23.6	21.6
2022-07-27 2:00 PM	20.1	20.3	22.0	20.8
2022-07-27 3:00 PM	19.9	20.1	20.9	20.3
2022-07-27 4:00 PM	19.9	25.0	23.0	22.6
2022-07-27 5:00 PM	19.7	21.1	21.1	20.6

2022-07-27 6:00 PM	19.6	20.4	20.6	20.2
2022-07-27 7:00 PM	18.9	18.8	18.8	18.8
2022-07-27 8:00 PM	18.3	18.3	17.9	18.2
2022-07-27 9:00 PM	17.7	16.7	16.6	17.0
2022-07-27 10:00 PM	17.3	17.0	16.3	16.9
2022-07-27 11:00 PM	17.0	17.1	16.3	16.8
2022-07-28 12:00 AM	16.5	16.6	15.8	16.3
2022-07-28 1:00 AM	16.2	16.3	15.3	15.9
2022-07-28 2:00 AM	16.3	16.4	15.5	16.1
2022-07-28 3:00 AM	16.3	16.3	15.5	16.0
2022-07-28 4:00 AM	16.2	16.4	15.5	16.0
2022-07-28 5:00 AM	16.2	16.4	15.5	16.0
2022-07-28 6:00 AM	16.1	16.2	15.2	15.8
2022-07-28 7:00 AM	15.8	16.0	14.9	15.6
2022-07-28 8:00 AM	15.8	15.9	14.8	15.5
2022-07-28 9:00 AM	16.2	16.3	15.4	16.0
2022-07-28 10:00 AM	16.7	16.2	15.7	16.2
2022-07-28 11:00 AM	17.7	16.9	16.8	17.1
2022-07-28 12:00 PM	18.6	17.3	17.6	17.8
2022-07-28 1:00 PM	18.8	18.0	19.3	18.7
2022-07-28 2:00 PM	18.6	18.4	19.3	18.8
2022-07-28 3:00 PM	18.5	18.3	18.5	18.4
2022-07-28 4:00 PM	18.5	18.3	18.3	18.4
2022-07-28 5:00 PM	18.7	18.9	18.9	18.8
2022-07-28 6:00 PM	18.6	19.1	19.0	18.9
2022-07-28 7:00 PM	18.4	18.3	18.3	18.3
2022-07-28 8:00 PM	18.0	18.0	17.8	17.9
2022-07-28 9:00 PM	17.7	17.9	17.4	17.7
2022-07-28 10:00 PM	17.1	17.4	16.6	17.0
2022-07-28 11:00 PM	16.9	17.2	16.3	16.8
2022-07-29 12:00 AM	16.6	16.8	15.8	16.4
2022-07-29 1:00 AM	16.2	16.5	15.3	16.0
2022-07-29 2:00 AM	16.0	16.1	15.1	15.7
2022-07-29 3:00 AM	16.1	16.3	15.3	15.9
2022-07-29 4:00 AM	16.2	16.4	15.5	16.0
2022-07-29 5:00 AM	16.2	16.4	15.5	16.0
2022-07-29 6:00 AM	16.2	16.3	15.4	16.0
2022-07-29 7:00 AM	16.2	16.4	15.5	16.0
2022-07-29 8:00 AM	16.4	16.3	15.6	16.1
2022-07-29 9:00 AM	16.7	16.2	15.6	16.2
2022-07-29 10:00 AM	17.3	16.9	16.5	16.9
2022-07-29 11:00 AM	18.3	18.1	18.1	18.2
2022-07-29 12:00 PM	19.7	18.7	19.2	19.2

2022-07-29 1:00 PM	19.7	19.2	20.0	19.6
2022-07-29 2:00 PM	19.4	19.2	19.8	19.5
2022-07-29 3:00 PM	19.2	19.0	19.4	19.2
2022-07-29 4:00 PM	19.3	19.1	19.4	19.3
2022-07-29 5:00 PM	19.0	18.8	19.1	19.0
2022-07-29 6:00 PM	18.9	18.6	19.0	18.8
2022-07-29 7:00 PM	18.8	18.6	19.0	18.8
2022-07-29 8:00 PM	18.1	17.8	17.9	17.9
2022-07-29 9:00 PM	17.8	17.9	17.4	17.7
2022-07-29 10:00 PM	17.3	17.5	16.7	17.2
2022-07-29 11:00 PM	17.2	17.2	16.4	16.9
2022-07-30 12:00 AM	16.9	17.0	16.1	16.7
2022-07-30 1:00 AM	16.5	16.5	15.5	16.2
2022-07-30 2:00 AM	16.1	16.1	15.0	15.7
2022-07-30 3:00 AM	15.7	15.8	14.6	15.4
2022-07-30 4:00 AM	15.4	15.4	14.3	15.0
2022-07-30 5:00 AM	15.1	15.2	14.0	14.8
2022-07-30 6:00 AM	15.0	15.0	13.9	14.6
2022-07-30 7:00 AM	14.9	14.9	13.8	14.5
2022-07-30 8:00 AM	15.1	15.3	14.1	14.8
2022-07-30 9:00 AM	16.0	16.3	15.2	15.8
2022-07-30 10:00 AM	16.9	17.0	16.5	16.8
2022-07-30 11:00 AM	18.2	18.2	18.5	18.3
2022-07-30 12:00 PM	21.7	19.4	21.4	20.8
2022-07-30 1:00 PM	20.9	20.3	23.5	21.6
2022-07-30 2:00 PM	19.7	20.1	21.8	20.5
2022-07-30 3:00 PM	19.8	20.2	21.2	20.4
2022-07-30 4:00 PM	19.8	22.3	21.8	21.3
2022-07-30 5:00 PM	19.6	20.7	21.1	20.5
2022-07-30 6:00 PM	19.6	21.4	21.2	20.7
2022-07-30 7:00 PM	19.6	22.8	22.5	21.6
2022-07-30 8:00 PM	19.2	20.4	20.5	20.0
2022-07-30 9:00 PM	19.0	19.8	19.7	19.5
2022-07-30 10:00 PM	18.6	19.3	18.8	18.9
2022-07-30 11:00 PM	18.2	18.7	18.0	18.3
2022-07-31 12:00 AM	17.8	18.2	17.3	17.8
2022-07-31 1:00 AM	17.4	17.8	16.8	17.3
2022-07-31 2:00 AM	17.3	17.7	16.7	17.2
2022-07-31 3:00 AM	17.1	17.4	16.4	17.0
2022-07-31 4:00 AM	16.9	17.1	16.0	16.7
2022-07-31 5:00 AM	16.8	16.9	16.0	16.6
2022-07-31 6:00 AM	16.9	16.8	16.1	16.6
2022-07-31 7:00 AM	16.6	16.6	15.8	16.3

2022-07-31 8:00 AM	16.8	16.8	15.9	16.5
2022-07-31 9:00 AM	17.6	17.8	17.0	17.5
2022-07-31 10:00 AM	18.1	18.1	17.6	17.9
2022-07-31 11:00 AM	18.9	18.5	18.4	18.6
2022-07-31 12:00 PM	20.2	19.2	19.8	19.7
2022-07-31 1:00 PM	20.4	19.8	22.0	20.7
2022-07-31 2:00 PM	20.0	20.5	22.2	20.9
2022-07-31 3:00 PM	19.8	19.6	20.4	19.9
2022-07-31 4:00 PM	19.6	19.6	19.9	19.7
2022-07-31 5:00 PM	20.1	20.7	21.2	20.7
2022-07-31 6:00 PM	19.7	20.1	20.4	20.1
2022-07-31 7:00 PM	19.3	19.6	19.6	19.5
2022-07-31 8:00 PM	19.1	19.4	19.2	19.2
2022-07-31 9:00 PM	18.9	18.9	18.6	18.8
2022-07-31 10:00 PM	18.3	18.2	17.8	18.1
2022-07-31 11:00 PM	18.1	18.1	17.6	17.9
2022-08-01 12:00 AM	17.9	17.7	17.1	17.6
2022-08-01 1:00 AM	17.6	17.6	16.9	17.4
2022-08-01 2:00 AM	17.6	17.5	16.9	17.3
2022-08-01 3:00 AM	17.6	17.7	17.0	17.4
2022-08-01 4:00 AM	17.6	17.7	16.9	17.4
2022-08-01 5:00 AM	17.6	17.8	17.0	17.5
2022-08-01 6:00 AM	17.6	17.8	16.9	17.4
2022-08-01 7:00 AM	17.7	17.9	17.2	17.6
2022-08-01 8:00 AM	17.9	17.9	17.3	17.7
2022-08-01 9:00 AM	17.9	17.7	17.1	17.6
2022-08-01 10:00 AM	18.3	17.5	17.2	17.7
2022-08-01 11:00 AM	18.7	17.7	17.8	18.1
2022-08-01 12:00 PM	19.1	17.9	18.2	18.4
2022-08-01 1:00 PM	19.5	18.3	19.2	19.0
2022-08-01 2:00 PM	19.4	18.4	19.1	19.0
2022-08-01 3:00 PM	19.4	18.7	19.3	19.1
2022-08-01 4:00 PM	19.4	19.0	19.5	19.3
2022-08-01 5:00 PM	19.4	19.4	19.8	19.5
2022-08-01 6:00 PM	19.1	19.6	19.8	19.5
2022-08-01 7:00 PM	19.0	20.5	20.4	20.0
2022-08-01 8:00 PM	18.4	18.7	18.7	18.6
2022-08-01 9:00 PM	18.0	18.3	17.8	18.0
2022-08-01 10:00 PM	17.4	17.8	16.9	17.4
2022-08-01 11:00 PM	17.1	17.3	16.1	16.8
2022-08-02 12:00 AM	16.7	16.9	15.6	16.4
2022-08-02 1:00 AM	16.3	16.4	15.1	15.9
2022-08-02 2:00 AM	15.9	16.1	14.7	15.6

2022-08-02 3:00 AM	15.9	16.1	14.9	15.6
2022-08-02 4:00 AM	15.6	15.7	14.4	15.2
2022-08-02 5:00 AM	15.4	15.4	14.1	15.0
2022-08-02 6:00 AM	15.2	15.3	14.0	14.8
2022-08-02 7:00 AM	15.2	15.5	14.2	15.0
2022-08-02 8:00 AM	15.6	15.7	14.6	15.3
2022-08-02 9:00 AM	16.4	16.4	15.4	16.1
2022-08-02 10:00 AM	17.4	17.3	16.9	17.2
2022-08-02 11:00 AM	18.2	18.0	18.3	18.2
2022-08-02 12:00 PM	20.9	18.7	20.6	20.1
2022-08-02 1:00 PM	20.3	19.4	22.9	20.9
2022-08-02 2:00 PM	19.1	20.0	22.2	20.4
2022-08-02 3:00 PM	19.1	19.8	20.9	19.9
2022-08-02 4:00 PM	19.3	21.6	21.4	20.8
2022-08-02 5:00 PM	18.6	19.2	18.7	18.8
2022-08-02 6:00 PM	18.5	19.2	18.7	18.8
2022-08-02 7:00 PM	18.2	18.4	17.8	18.1
2022-08-02 8:00 PM	17.9	17.9	17.4	17.7
2022-08-02 9:00 PM	17.7	17.6	17.2	17.5
2022-08-02 10:00 PM	17.6	17.6	17.0	17.4
2022-08-02 11:00 PM	17.4	17.3	16.6	17.1
2022-08-03 12:00 AM	17.2	17.2	16.3	16.9
2022-08-03 1:00 AM	17.2	17.3	16.4	17.0
2022-08-03 2:00 AM	17.2	17.2	16.5	17.0
2022-08-03 3:00 AM	17.3	17.3	16.5	17.0
2022-08-03 4:00 AM	17.3	17.4	16.6	17.1
2022-08-03 5:00 AM	17.2	17.3	16.6	17.0
2022-08-03 6:00 AM	16.9	17.1	16.1	16.7
2022-08-03 7:00 AM	16.8	17.0	15.9	16.6
2022-08-03 8:00 AM	16.9	17.1	16.0	16.7
2022-08-03 9:00 AM	17.4	17.6	17.0	17.3
2022-08-03 10:00 AM	18.2	18.5	18.5	18.4
2022-08-03 11:00 AM	19.4	19.7	20.3	19.8
2022-08-03 12:00 PM	20.6	20.2	21.2	20.7
2022-08-03 1:00 PM	19.8	20.0	20.7	20.2
2022-08-03 2:00 PM	20.0	20.6	21.4	20.7
2022-08-03 3:00 PM	20.2	20.8	21.3	20.8
2022-08-03 4:00 PM	20.1	23.0	22.5	21.9
2022-08-03 5:00 PM	19.8	21.1	21.1	20.7
2022-08-03 6:00 PM	20.0	20.9	20.9	20.6
2022-08-03 7:00 PM	19.5	20.1	19.8	19.8
2022-08-03 8:00 PM	19.3	19.7	19.3	19.4
2022-08-03 9:00 PM	18.9	19.3	18.8	19.0

2022-08-03 10:00 PM	18.5	18.7	18.1	18.4
2022-08-03 11:00 PM	18.1	18.2	17.6	18.0
2022-08-04 12:00 AM	18.0	18.3	17.4	17.9
2022-08-04 1:00 AM	17.9	18.1	17.3	17.8
2022-08-04 2:00 AM	17.3	17.5	16.5	17.1
2022-08-04 3:00 AM	17.1	17.2	16.1	16.8
2022-08-04 4:00 AM	16.4	16.5	15.4	16.1
2022-08-04 5:00 AM	16.0	16.2	14.9	15.7
2022-08-04 6:00 AM	15.7	15.7	14.3	15.2
2022-08-04 7:00 AM	15.3	15.4	14.0	14.9
2022-08-04 8:00 AM	15.4	15.5	14.1	15.0
2022-08-04 9:00 AM	16.1	16.3	15.0	15.8
2022-08-04 10:00 AM	17.2	17.4	16.6	17.1
2022-08-04 11:00 AM	18.2	18.1	17.9	18.1
2022-08-04 12:00 PM	19.8	18.5	19.4	19.2
2022-08-04 1:00 PM	19.6	19.1	21.4	20.0
2022-08-04 2:00 PM	18.9	19.6	21.0	19.8
2022-08-04 3:00 PM	18.9	19.4	20.0	19.4
2022-08-04 4:00 PM	19.1	21.0	21.0	20.4
2022-08-04 5:00 PM	18.8	19.5	19.8	19.4
2022-08-04 6:00 PM	18.6	19.5	19.8	19.3
2022-08-04 7:00 PM	18.7	20.9	20.5	20.0
2022-08-04 8:00 PM	18.3	19.0	18.6	18.6
2022-08-04 9:00 PM	18.1	18.6	18.0	18.2
2022-08-04 10:00 PM	17.7	18.1	17.3	17.7
2022-08-04 11:00 PM	17.2	17.5	16.6	17.1
2022-08-05 12:00 AM	16.9	17.0	16.1	16.7
2022-08-05 1:00 AM	16.6	16.7	15.5	16.3
2022-08-05 2:00 AM	16.2	16.4	15.1	15.9
2022-08-05 3:00 AM	16.1	16.1	14.7	15.6
2022-08-05 4:00 AM	15.8	15.9	14.6	15.4
2022-08-05 5:00 AM	15.8	16.0	14.6	15.5
2022-08-05 6:00 AM	15.6	15.8	14.5	15.3
2022-08-05 7:00 AM	15.4	15.6	14.3	15.1
2022-08-05 8:00 AM	16.0	16.1	15.0	15.7
2022-08-05 9:00 AM	16.6	16.7	15.6	16.3
2022-08-05 10:00 AM	17.1	17.2	16.6	17.0
2022-08-05 11:00 AM	18.1	17.9	18.2	18.1
2022-08-05 12:00 PM	20.4	18.7	21.0	20.0
2022-08-05 1:00 PM	20.2	19.6	24.1	21.3
2022-08-05 2:00 PM	19.4	20.2	23.1	20.9
2022-08-05 3:00 PM	19.7	20.4	22.0	20.7
2022-08-05 4:00 PM	19.8	21.7	22.8	21.4

2022-08-05 5:00 PM	19.6	20.8	21.8	20.7
2022-08-05 6:00 PM	19.4	19.9	20.4	19.9
2022-08-05 7:00 PM	18.9	19.2	19.0	19.0
2022-08-05 8:00 PM	18.8	19.2	18.9	19.0
2022-08-05 9:00 PM	18.7	18.9	18.5	18.7
2022-08-05 10:00 PM	18.4	18.5	18.0	18.3
2022-08-05 11:00 PM	18.2	18.3	17.6	18.0
2022-08-06 12:00 AM	17.8	17.7	16.9	17.5
2022-08-06 1:00 AM	17.8	17.9	17.1	17.6
2022-08-06 2:00 AM	18.0	18.1	17.5	17.9
2022-08-06 3:00 AM	18.1	18.2	17.6	18.0
2022-08-06 4:00 AM	18.1	18.3	17.6	18.0
2022-08-06 5:00 AM	18.2	18.4	17.8	18.1
2022-08-06 6:00 AM	18.3	18.6	18.3	18.4
2022-08-06 7:00 AM	18.3	18.5	18.1	18.3
2022-08-06 8:00 AM	18.3	18.6	18.2	18.4
2022-08-06 9:00 AM	18.7	19.0	18.8	18.8
2022-08-06 10:00 AM	19.4	19.7	20.0	19.7
2022-08-06 11:00 AM	19.9	20.3	20.8	20.3
2022-08-06 12:00 PM	20.4	20.6	21.3	20.8
2022-08-06 1:00 PM	20.6	20.7	21.6	21.0
2022-08-06 2:00 PM	20.5	20.8	21.7	21.0
2022-08-06 3:00 PM	20.5	21.0	21.8	21.1
2022-08-06 4:00 PM	20.5	22.2	22.6	21.8
2022-08-06 5:00 PM	20.2	21.1	21.5	20.9
2022-08-06 6:00 PM	20.5	21.5	22.1	21.4
2022-08-06 7:00 PM	20.4	21.3	21.8	21.2
2022-08-06 8:00 PM	20.0	20.2	20.6	20.3
2022-08-06 9:00 PM	19.6	19.8	19.8	19.7
2022-08-06 10:00 PM	19.1	19.4	18.8	19.1
2022-08-06 11:00 PM	18.8	18.7	18.2	18.6
2022-08-07 12:00 AM	18.2	17.7	17.3	17.7
2022-08-07 1:00 AM	17.7	17.3	16.6	17.2
2022-08-07 2:00 AM	17.5	17.0	16.3	16.9
2022-08-07 3:00 AM	17.3	16.9	16.1	16.8
2022-08-07 4:00 AM	17.1	16.6	15.9	16.5
2022-08-07 5:00 AM	16.8	16.6	15.7	16.4
2022-08-07 6:00 AM	16.6	16.2	15.3	16.0
2022-08-07 7:00 AM	16.4	16.1	15.1	15.9
2022-08-07 8:00 AM	16.4	16.0	15.1	15.8
2022-08-07 9:00 AM	16.7	16.1	15.5	16.1
2022-08-07 10:00 AM	17.3	16.5	16.1	16.6
2022-08-07 11:00 AM	18.4	17.5	17.4	17.8

2022-08-07 12:00 PM	19.6	17.9	18.9	18.8
2022-08-07 1:00 PM	19.6	18.7	21.4	19.9
2022-08-07 2:00 PM	18.9	19.0	20.2	19.4
2022-08-07 3:00 PM	18.1	18.3	17.7	18.0
2022-08-07 4:00 PM	17.6	18.8	17.7	18.0
2022-08-07 5:00 PM	17.3	17.9	16.8	17.3
2022-08-07 6:00 PM	17.2	17.6	16.6	17.1
2022-08-07 7:00 PM	17.2	17.4	16.5	17.0
2022-08-07 8:00 PM	17.0	17.0	16.2	16.7
2022-08-07 9:00 PM	16.8	16.7	16.0	16.5
2022-08-07 10:00 PM	16.5	16.4	15.5	16.1
2022-08-07 11:00 PM	16.3	16.2	15.4	16.0
2022-08-08 12:00 AM	16.2	16.2	15.2	15.9
2022-08-08 1:00 AM	16.0	16.2	15.1	15.8
2022-08-08 2:00 AM	16.0	16.0	15.0	15.7
2022-08-08 3:00 AM	15.7	15.7	14.8	15.4
2022-08-08 4:00 AM	15.6	15.5	14.7	15.3
2022-08-08 5:00 AM	15.6	15.6	14.7	15.3
2022-08-08 6:00 AM	15.5	15.7	14.8	15.3
2022-08-08 7:00 AM	15.5	15.6	14.6	15.2
2022-08-08 8:00 AM	15.6	15.6	14.8	15.3
2022-08-08 9:00 AM	15.9	15.9	15.2	15.7
2022-08-08 10:00 AM	16.5	16.4	15.8	16.2
2022-08-08 11:00 AM	17.4	17.2	17.2	17.3
2022-08-08 12:00 PM	19.2	17.8	19.1	18.7
2022-08-08 1:00 PM	18.7	18.4	21.3	19.5
2022-08-08 2:00 PM	17.9	18.5	20.0	18.8
2022-08-08 3:00 PM	17.7	18.2	18.6	18.2
2022-08-08 4:00 PM	18.1	19.1	19.6	18.9
2022-08-08 5:00 PM	18.2	18.8	19.5	18.8
2022-08-08 6:00 PM	18.2	19.0	19.4	18.9
2022-08-08 7:00 PM	18.4	20.5	20.9	19.9
2022-08-08 8:00 PM	17.9	18.6	18.5	18.3
2022-08-08 9:00 PM	17.7	18.2	17.8	17.9
2022-08-08 10:00 PM	17.3	17.6	16.9	17.3
2022-08-08 11:00 PM	16.8	17.0	16.2	16.7
2022-08-09 12:00 AM	16.4	16.6	15.7	16.2
2022-08-09 1:00 AM	16.1	16.3	15.3	15.9
2022-08-09 2:00 AM	15.8	16.0	14.9	15.6
2022-08-09 3:00 AM	15.6	15.8	14.6	15.3
2022-08-09 4:00 AM	15.4	15.6	14.2	15.1
2022-08-09 5:00 AM	15.2	15.4	14.1	14.9
2022-08-09 6:00 AM	15.0	15.2	13.7	14.6

2022-08-09 7:00 AM	14.7	14.9	13.7	14.4
2022-08-09 8:00 AM	14.8	14.9	13.7	14.5
2022-08-09 9:00 AM	15.6	15.8	14.6	15.3
2022-08-09 10:00 AM	16.7	16.9	16.4	16.7
2022-08-09 11:00 AM	17.7	17.8	18.1	17.9
2022-08-09 12:00 PM	19.3	18.6	20.1	19.3
2022-08-09 1:00 PM	19.3	19.6	22.3	20.4
2022-08-09 2:00 PM	18.6	19.8	21.4	19.9
2022-08-09 3:00 PM	18.6	19.3	20.2	19.4
2022-08-09 4:00 PM	18.9	20.8	21.6	20.4
2022-08-09 5:00 PM	19.1	19.9	20.8	19.9
2022-08-09 6:00 PM	19.4	20.7	21.4	20.5
2022-08-09 7:00 PM	19.7	22.4	23.0	21.7
2022-08-09 8:00 PM	19.5	20.5	21.0	20.3
2022-08-09 9:00 PM	19.4	20.2	20.4	20.0
2022-08-09 10:00 PM	19.2	19.8	19.8	19.6
2022-08-09 11:00 PM	19.0	19.6	19.4	19.3
2022-08-10 12:00 AM	18.8	19.4	18.9	19.0
2022-08-10 1:00 AM	18.5	19.0	18.3	18.6
2022-08-10 2:00 AM	18.2	18.7	17.8	18.2
2022-08-10 3:00 AM	17.7	18.1	17.1	17.6
2022-08-10 4:00 AM	17.1	17.6	16.4	17.0
2022-08-10 5:00 AM	16.7	17.1	15.8	16.5
2022-08-10 6:00 AM	16.7	17.0	15.7	16.5
2022-08-10 7:00 AM	16.4	16.8	15.4	16.2
2022-08-10 8:00 AM	16.4	16.8	15.3	16.2
2022-08-10 9:00 AM	16.8	17.3	16.0	16.7
2022-08-10 10:00 AM	17.5	17.8	17.0	17.4
2022-08-10 11:00 AM	18.6	18.6	18.7	18.6
2022-08-10 12:00 PM	20.6	19.3	20.7	20.2
2022-08-10 1:00 PM	20.0	19.8	21.4	20.4
2022-08-10 2:00 PM	18.4	19.1	19.6	19.0
2022-08-10 3:00 PM	18.3	19.3	19.7	19.1
2022-08-10 4:00 PM	18.3	20.4	20.1	19.6
2022-08-10 5:00 PM	18.6	19.8	20.0	19.5
2022-08-10 6:00 PM	18.7	20.3	20.4	19.8
2022-08-10 7:00 PM	18.7	20.4	20.2	19.8
2022-08-10 8:00 PM	18.4	19.1	18.9	18.8
2022-08-10 9:00 PM	18.2	18.7	18.2	18.4
2022-08-10 10:00 PM	17.6	18.3	17.2	17.7
2022-08-10 11:00 PM	17.2	17.7	16.5	17.1
2022-08-11 12:00 AM	16.8	17.3	15.9	16.7
2022-08-11 1:00 AM	16.4	16.8	15.4	16.2

2022-08-11 2:00 AM	16.0	16.5	14.8	15.8
2022-08-11 3:00 AM	15.6	16.2	14.5	15.4
2022-08-11 4:00 AM	15.4	15.7	14.1	15.1
2022-08-11 5:00 AM	15.0	15.3	13.6	14.6
2022-08-11 6:00 AM	14.8	14.9	13.2	14.3
2022-08-11 7:00 AM	14.3	14.5	12.8	13.9
2022-08-11 8:00 AM	14.1	14.4	12.8	13.8
2022-08-11 9:00 AM	14.7	15.2	13.6	14.5
2022-08-11 10:00 AM	15.9	16.3	15.1	15.8
2022-08-11 11:00 AM	17.1	17.2	17.0	17.1
2022-08-11 12:00 PM	19.3	18.0	19.5	18.9
2022-08-11 1:00 PM	18.8	18.9	21.6	19.8
2022-08-11 2:00 PM	17.9	18.9	20.0	18.9
2022-08-11 3:00 PM	17.6	18.4	18.5	18.2
2022-08-11 4:00 PM	17.8	20.2	20.1	19.4
2022-08-11 5:00 PM	17.8	18.8	18.8	18.5
2022-08-11 6:00 PM	18.0	19.1	19.4	18.8
2022-08-11 7:00 PM	18.2	20.9	20.4	19.8
2022-08-11 8:00 PM	17.7	18.6	18.3	18.2
2022-08-11 9:00 PM	17.4	18.0	17.5	17.6
2022-08-11 10:00 PM	16.7	17.3	16.4	16.8
2022-08-11 11:00 PM	16.3	16.7	15.6	16.2
2022-08-12 12:00 AM	15.8	16.1	14.9	15.6
2022-08-12 1:00 AM	15.3	15.6	14.2	15.0
2022-08-12 2:00 AM	14.9	15.2	13.8	14.6
2022-08-12 3:00 AM	14.5	14.8	13.3	14.2
2022-08-12 4:00 AM	14.1	14.5	12.8	13.8
2022-08-12 5:00 AM	13.8	14.2	12.4	13.5
2022-08-12 6:00 AM	13.4	13.9	12.0	13.1
2022-08-12 7:00 AM	13.2	13.5	11.9	12.9
2022-08-12 8:00 AM	13.2	13.5	12.0	12.9
2022-08-12 9:00 AM	13.6	13.9	12.4	13.3
2022-08-12 10:00 AM	15.2	15.6	14.5	15.1
2022-08-12 11:00 AM	16.5	16.6	16.2	16.4
2022-08-12 12:00 PM	17.4	17.0	17.5	17.3
2022-08-12 1:00 PM	17.5	17.7	19.4	18.2
2022-08-12 2:00 PM	16.9	18.0	19.0	18.0
2022-08-12 3:00 PM	16.8	17.6	17.8	17.4
2022-08-12 4:00 PM	16.8	18.8	18.4	18.0
2022-08-12 5:00 PM	16.8	17.7	17.6	17.4
2022-08-12 6:00 PM	17.2	18.1	18.3	17.9
2022-08-12 7:00 PM	17.2	18.2	18.0	17.8
2022-08-12 8:00 PM	16.8	17.1	16.7	16.9

2022-08-12 9:00 PM	16.5	16.7	16.1	16.4
2022-08-12 10:00 PM	16.2	16.4	15.6	16.1
2022-08-12 11:00 PM	15.8	16.0	15.1	15.6
2022-08-13 12:00 AM	15.5	15.7	14.7	15.3
2022-08-13 1:00 AM	15.2	15.6	14.3	15.0
2022-08-13 2:00 AM	15.1	15.3	14.0	14.8
2022-08-13 3:00 AM	15.0	15.2	14.1	14.8
2022-08-13 4:00 AM	15.4	15.6	14.6	15.2
2022-08-13 5:00 AM	15.6	15.8	14.9	15.4
2022-08-13 6:00 AM	15.7	15.9	15.0	15.5
2022-08-13 7:00 AM	15.8	16.0	15.1	15.6
2022-08-13 8:00 AM	15.9	16.1	15.2	15.7
2022-08-13 9:00 AM	16.1	16.3	15.6	16.0
2022-08-13 10:00 AM	16.4	16.7	16.2	16.4
2022-08-13 11:00 AM	16.7	16.9	16.5	16.7
2022-08-13 12:00 PM	17.8	17.8	17.9	17.8
2022-08-13 1:00 PM	18.6	18.9	20.9	19.5
2022-08-13 2:00 PM	17.9	19.2	20.5	19.2
2022-08-13 3:00 PM	17.6	18.4	18.8	18.3
2022-08-13 4:00 PM	17.6	19.8	19.3	18.9
2022-08-13 5:00 PM	17.5	18.5	18.4	18.1
2022-08-13 6:00 PM	17.6	18.5	18.3	18.1
2022-08-13 7:00 PM	17.5	19.7	18.6	18.6
2022-08-13 8:00 PM	17.0	17.6	16.9	17.2
2022-08-13 9:00 PM	16.6	17.1	16.2	16.6
2022-08-13 10:00 PM	16.6	16.9	16.0	16.5
2022-08-13 11:00 PM	16.6	16.8	16.1	16.5
2022-08-14 12:00 AM	16.4	16.6	15.8	16.3
2022-08-14 1:00 AM	16.4	16.6	15.7	16.2
2022-08-14 2:00 AM	16.5	16.7	15.8	16.3
2022-08-14 3:00 AM	16.5	16.7	15.8	16.3
2022-08-14 4:00 AM	16.4	16.7	15.8	16.3
2022-08-14 5:00 AM	16.4	16.6	15.8	16.3
2022-08-14 6:00 AM	16.4	16.6	15.8	16.3
2022-08-14 7:00 AM	16.4	16.6	15.8	16.3
2022-08-14 8:00 AM	16.5	16.7	15.9	16.4
2022-08-14 9:00 AM	16.6	16.7	16.0	16.4
2022-08-14 10:00 AM	16.7	16.8	16.0	16.5
2022-08-14 11:00 AM	16.9	17.0	16.4	16.8
2022-08-14 12:00 PM	17.0	17.1	16.6	16.9
2022-08-14 1:00 PM	17.5	17.7	17.4	17.5
2022-08-14 2:00 PM	17.6	18.2	18.0	17.9
2022-08-14 3:00 PM	17.9	18.5	18.3	18.2

2022-08-14 4:00 PM	18.1	18.6	18.5	18.4
2022-08-14 5:00 PM	18.0	18.7	18.6	18.4
2022-08-14 6:00 PM	17.8	18.5	18.2	18.2
2022-08-14 7:00 PM	17.7	18.9	18.1	18.2
2022-08-14 8:00 PM	17.4	17.8	17.2	17.5
2022-08-14 9:00 PM	17.0	17.2	16.5	16.9
2022-08-14 10:00 PM	16.5	16.7	15.9	16.4
2022-08-14 11:00 PM	16.1	16.3	15.5	16.0
2022-08-15 12:00 AM	16.1	16.3	15.4	15.9
2022-08-15 1:00 AM	16.4	16.6	15.7	16.2
2022-08-15 2:00 AM	16.5	16.7	15.9	16.4
2022-08-15 3:00 AM	16.4	16.6	15.7	16.2
2022-08-15 4:00 AM	16.5	16.7	15.9	16.4
2022-08-15 5:00 AM	16.5	16.7	15.8	16.3
2022-08-15 6:00 AM	16.5	16.6	15.7	16.3
2022-08-15 7:00 AM	16.5	16.7	15.8	16.3
2022-08-15 8:00 AM	16.5	16.7	15.9	16.4
2022-08-15 9:00 AM	16.7	16.8	16.2	16.6
2022-08-15 10:00 AM	17.1	17.2	16.8	17.0
2022-08-15 11:00 AM	17.7	17.8	17.7	17.7
2022-08-15 12:00 PM	19.2	18.6	19.5	19.1
2022-08-15 1:00 PM	19.3	19.3	21.3	20.0
2022-08-15 2:00 PM	18.4	19.6	20.6	19.5
2022-08-15 3:00 PM	19.2	18.9	18.8	19.0
2022-08-15 4:00 PM	18.4	19.2	18.6	18.7
2022-08-15 5:00 PM	18.0	18.4	18.1	18.2
2022-08-15 6:00 PM	18.2	18.7	18.5	18.5
2022-08-15 7:00 PM	18.1	19.6	19.1	18.9
2022-08-15 8:00 PM	17.7	18.1	17.8	17.9
2022-08-15 9:00 PM	17.4	17.8	17.2	17.5
2022-08-15 10:00 PM	17.0	17.5	16.6	17.0
2022-08-15 11:00 PM	16.5	17.0	16.0	16.5
2022-08-16 12:00 AM	16.2	16.6	15.7	16.2
2022-08-16 1:00 AM	15.9	16.1	15.2	15.7
2022-08-16 2:00 AM	15.5	15.7	14.7	15.3
2022-08-16 3:00 AM	15.2	15.4	14.3	15.0
2022-08-16 4:00 AM	14.9	15.1	13.9	14.6
2022-08-16 5:00 AM	14.8	14.9	13.7	14.5
2022-08-16 6:00 AM	14.6	14.7	13.5	14.3
2022-08-16 7:00 AM	14.4	14.5	13.3	14.1
2022-08-16 8:00 AM	14.4	14.6	13.3	14.1
2022-08-16 9:00 AM	15.2	15.6	14.3	15.0
2022-08-16 10:00 AM	16.1	16.5	16.0	16.2

2022-08-16 11:00 AM	17.5	17.6	17.9	17.7
2022-08-16 12:00 PM	19.1	18.4	19.9	19.1
2022-08-16 1:00 PM	19.0	19.2	21.7	20.0
2022-08-16 2:00 PM	18.2	19.4	20.8	19.5
2022-08-16 3:00 PM	17.9	18.8	19.5	18.7
2022-08-16 4:00 PM	18.0	19.5	19.8	19.1
2022-08-16 5:00 PM	18.1	19.1	19.4	18.9
2022-08-16 6:00 PM	18.4	19.4	19.6	19.1
2022-08-16 7:00 PM	18.2	19.8	19.5	19.2
2022-08-16 8:00 PM	17.7	18.3	18.0	18.0
2022-08-16 9:00 PM	17.7	18.0	17.5	17.7
2022-08-16 10:00 PM	17.4	17.7	17.1	17.4
2022-08-16 11:00 PM	17.1	17.5	16.6	17.1
2022-08-17 12:00 AM	16.9	17.3	16.3	16.8
2022-08-17 1:00 AM	17.0	17.3	16.5	16.9
2022-08-17 2:00 AM	17.2	17.5	16.7	17.1
2022-08-17 3:00 AM	17.2	17.5	16.8	17.2
2022-08-17 4:00 AM	17.2	17.5	16.7	17.1
2022-08-17 5:00 AM	16.9	17.3	16.3	16.8
2022-08-17 6:00 AM	16.7	16.9	16.1	16.6
2022-08-17 7:00 AM	16.7	16.9	15.9	16.5
2022-08-17 8:00 AM	16.8	17.0	16.2	16.7
2022-08-17 9:00 AM	17.2	17.4	16.8	17.1
2022-08-17 10:00 AM	17.6	17.8	17.4	17.6
2022-08-17 11:00 AM	18.2	18.2	18.1	18.2
2022-08-17 12:00 PM	19.0	18.7	19.0	18.9
2022-08-17 1:00 PM	19.6	19.6	20.2	19.8
2022-08-17 2:00 PM	19.8	20.1	20.9	20.3
2022-08-17 3:00 PM	19.9	20.2	20.9	20.3
2022-08-17 4:00 PM	19.7	20.6	21.0	20.4
2022-08-17 5:00 PM	19.4	19.7	20.2	19.8
2022-08-17 6:00 PM	19.1	19.3	19.3	19.2
2022-08-17 7:00 PM	19.1	19.5	19.4	19.3
2022-08-17 8:00 PM	18.7	18.8	18.6	18.7
2022-08-17 9:00 PM	18.5	18.5	18.1	18.4
2022-08-17 10:00 PM	18.1	18.0	17.5	17.9
2022-08-17 11:00 PM	17.7	17.8	17.0	17.5
2022-08-18 12:00 AM	17.5	17.6	16.7	17.3
2022-08-18 1:00 AM	17.5	17.5	16.7	17.2
2022-08-18 2:00 AM	17.7	17.8	17.0	17.5
2022-08-18 3:00 AM	17.5	17.7	16.8	17.3
2022-08-18 4:00 AM	17.3	17.5	16.6	17.1
2022-08-18 5:00 AM	17.1	17.3	16.2	16.9

2022-08-18 6:00 AM	16.8	16.8	15.8	16.5
2022-08-18 7:00 AM	16.7	16.7	15.8	16.4
2022-08-18 8:00 AM	16.8	16.9	16.0	16.6
2022-08-18 9:00 AM	17.1	17.3	16.5	17.0
2022-08-18 10:00 AM	17.8	18.1	17.8	17.9
2022-08-18 11:00 AM	18.7	19.0	19.2	19.0
2022-08-18 12:00 PM	19.8	20.1	20.9	20.3
2022-08-18 1:00 PM	20.8	21.6	23.6	22.0
2022-08-18 2:00 PM	20.5	21.5	22.5	21.5
2022-08-18 3:00 PM	20.0	20.4	20.9	20.4
2022-08-18 4:00 PM	19.8	20.0	19.7	19.8
2022-08-18 5:00 PM	20.0	20.0	19.9	20.0
2022-08-18 6:00 PM	19.6	19.6	19.4	19.5
2022-08-18 7:00 PM	19.6	19.6	19.3	19.5
2022-08-18 8:00 PM	19.4	19.7	19.3	19.5
2022-08-18 9:00 PM	19.0	19.1	18.6	18.9
2022-08-18 10:00 PM	18.5	18.7	17.8	18.3
2022-08-18 11:00 PM	18.2	18.4	17.5	18.0
2022-08-19 12:00 AM	18.0	18.1	17.2	17.8
2022-08-19 1:00 AM	18.1	18.2	17.3	17.9
2022-08-19 2:00 AM	18.3	18.4	17.6	18.1
2022-08-19 3:00 AM	18.2	18.4	17.6	18.1
2022-08-19 4:00 AM	18.2	18.4	17.6	18.1
2022-08-19 5:00 AM	18.2	18.4	17.6	18.1
2022-08-19 6:00 AM	18.2	18.3	17.6	18.0
2022-08-19 7:00 AM	18.1	18.2	17.5	17.9
2022-08-19 8:00 AM	18.0	18.1	17.4	17.8
2022-08-19 9:00 AM	18.2	18.3	17.6	18.0
2022-08-19 10:00 AM	18.4	18.5	18.0	18.3
2022-08-19 11:00 AM	18.7	18.9	18.4	18.7
2022-08-19 12:00 PM	19.2	19.3	19.0	19.2
2022-08-19 1:00 PM	19.7	19.9	20.1	19.9
2022-08-19 2:00 PM	19.4	20.3	20.7	20.1
2022-08-19 3:00 PM	19.3	19.9	20.0	19.7
2022-08-19 4:00 PM	19.6	20.6	20.3	20.2
2022-08-19 5:00 PM	20.0	20.3	20.3	20.2
2022-08-19 6:00 PM	19.8	20.0	20.1	20.0
2022-08-19 7:00 PM	19.6	19.8	19.6	19.7
2022-08-19 8:00 PM	19.6	19.8	19.7	19.7
2022-08-19 9:00 PM	19.2	19.5	19.1	19.3
2022-08-19 10:00 PM	19.0	19.1	18.7	18.9
2022-08-19 11:00 PM	18.7	18.6	18.1	18.5
2022-08-20 12:00 AM	18.5	18.5	17.8	18.3

2022-08-20 1:00 AM	18.4	18.2	17.6	18.1
2022-08-20 2:00 AM	18.4	18.2	17.6	18.1
2022-08-20 3:00 AM	18.3	18.3	17.6	18.1
2022-08-20 4:00 AM	18.3	18.1	17.4	17.9
2022-08-20 5:00 AM	18.2	18.0	17.4	17.9
2022-08-20 6:00 AM	18.1	17.8	17.1	17.7
2022-08-20 7:00 AM	17.7	17.8	16.7	17.4
2022-08-20 8:00 AM	17.5	17.4	16.4	17.1
2022-08-20 9:00 AM	17.9	17.5	16.7	17.4
2022-08-20 10:00 AM	18.3	17.8	17.1	17.7
2022-08-20 11:00 AM	18.9	18.7	18.2	18.6
2022-08-20 12:00 PM	19.8	19.8	19.9	19.8
2022-08-20 1:00 PM	19.6	19.7	19.7	19.7
2022-08-20 2:00 PM	19.6	20.0	20.0	19.9
2022-08-20 3:00 PM	19.0	19.5	19.2	19.2
2022-08-20 4:00 PM	18.8	20.2	19.1	19.4
2022-08-20 5:00 PM	18.9	19.4	18.8	19.0
2022-08-20 6:00 PM	19.1	20.2	19.3	19.5
2022-08-20 7:00 PM	19.0	20.7	19.8	19.8
2022-08-20 8:00 PM	18.5	19.0	18.2	18.6
2022-08-20 9:00 PM	18.2	18.5	17.6	18.1
2022-08-20 10:00 PM	17.6	17.9	16.8	17.4
2022-08-20 11:00 PM	17.2	17.4	16.1	16.9
2022-08-21 12:00 AM	16.9	16.7	15.6	16.4
2022-08-21 1:00 AM	16.5	16.4	15.2	16.0
2022-08-21 2:00 AM	16.1	16.0	14.8	15.6
2022-08-21 3:00 AM	15.8	15.6	14.4	15.3
2022-08-21 4:00 AM	15.4	15.1	13.9	14.8
2022-08-21 5:00 AM	15.1	14.8	13.5	14.5
2022-08-21 6:00 AM	14.8	14.5	13.3	14.2
2022-08-21 7:00 AM	14.6	14.2	13.0	13.9
2022-08-21 8:00 AM	14.5	14.1	13.0	13.9
2022-08-21 9:00 AM	15.1	15.1	13.9	14.7
2022-08-21 10:00 AM	16.2	16.5	15.5	16.1
2022-08-21 11:00 AM	17.5	17.7	17.6	17.6
2022-08-21 12:00 PM	18.8	18.8	19.6	19.1
2022-08-21 1:00 PM	18.9	19.7	21.3	20.0
2022-08-21 2:00 PM	18.4	19.9	20.9	19.7
2022-08-21 3:00 PM	18.1	19.1	19.4	18.9
2022-08-21 4:00 PM	18.1	20.0	19.6	19.2
2022-08-21 5:00 PM	18.3	19.3	19.5	19.0
2022-08-21 6:00 PM	18.5	20.1	19.7	19.4
2022-08-21 7:00 PM	18.4	20.8	20.2	19.8

2022-08-21 8:00 PM	18.2	19.1	18.9	18.7
2022-08-21 9:00 PM	17.9	18.6	18.0	18.2
2022-08-21 10:00 PM	17.5	18.0	17.0	17.5
2022-08-21 11:00 PM	17.3	17.7	16.6	17.2
2022-08-22 12:00 AM	17.1	17.3	16.2	16.9
2022-08-22 1:00 AM	16.8	16.9	15.8	16.5
2022-08-22 2:00 AM	16.5	16.6	15.4	16.2
2022-08-22 3:00 AM	16.3	16.3	15.2	15.9
2022-08-22 4:00 AM	16.1	16.2	14.9	15.7
2022-08-22 5:00 AM	15.8	15.8	14.7	15.4
2022-08-22 6:00 AM	15.8	15.8	14.8	15.5
2022-08-22 7:00 AM	15.7	15.7	14.6	15.3
2022-08-22 8:00 AM	15.6	15.6	14.6	15.3
2022-08-22 9:00 AM	15.9	16.0	14.9	15.6
2022-08-22 10:00 AM	17.0	17.3	16.6	17.0
2022-08-22 11:00 AM	18.0	18.4	18.4	18.3
2022-08-22 12:00 PM	18.9	19.4	19.8	19.4
2022-08-22 1:00 PM	19.0	20.1	21.1	20.1
2022-08-22 2:00 PM	19.1	20.2	21.0	20.1
2022-08-22 3:00 PM	19.0	19.9	20.3	19.7
2022-08-22 4:00 PM	18.9	19.6	19.8	19.4
2022-08-22 5:00 PM	18.5	18.8	18.6	18.6
2022-08-22 6:00 PM	18.8	19.2	19.0	19.0
2022-08-22 7:00 PM	19.0	19.7	19.7	19.5
2022-08-22 8:00 PM	18.9	19.4	19.4	19.2
2022-08-22 9:00 PM	18.7	19.0	18.7	18.8
2022-08-22 10:00 PM	18.4	18.6	18.1	18.4
2022-08-22 11:00 PM	18.5	18.7	18.2	18.5
2022-08-23 12:00 AM	18.4	18.7	18.1	18.4
2022-08-23 1:00 AM	18.4	18.4	17.9	18.2
2022-08-23 2:00 AM	18.3	18.4	17.8	18.2
2022-08-23 3:00 AM	18.3	18.5	17.9	18.2
2022-08-23 4:00 AM	18.3	18.5	17.9	18.2
2022-08-23 5:00 AM	18.2	18.4	17.8	18.1
2022-08-23 6:00 AM	18.1	18.2	17.5	17.9
2022-08-23 7:00 AM	18.0	18.0	17.3	17.8
2022-08-23 8:00 AM	18.0	18.0	17.2	17.7
2022-08-23 9:00 AM	18.1	18.2	17.5	17.9
2022-08-23 10:00 AM	18.4	18.5	17.9	18.3
2022-08-23 11:00 AM	18.9	19.1	18.8	18.9
2022-08-23 12:00 PM	19.1	19.4	19.2	19.2
2022-08-23 1:00 PM	19.4	19.9	20.1	19.8
2022-08-23 2:00 PM	19.8	20.7	21.1	20.5

2022-08-23 3:00 PM	19.9	20.6	20.9	20.5
2022-08-23 4:00 PM	19.8	20.6	20.6	20.3
2022-08-23 5:00 PM	19.8	20.4	20.5	20.2
2022-08-23 6:00 PM	20.0	20.7	20.7	20.5
2022-08-23 7:00 PM	19.8	21.0	20.7	20.5
2022-08-23 8:00 PM	19.3	19.6	19.3	19.4
2022-08-23 9:00 PM	18.8	19.0	18.4	18.7
2022-08-23 10:00 PM	18.6	18.8	18.0	18.5
2022-08-23 11:00 PM	18.2	18.3	17.4	18.0
2022-08-24 12:00 AM	17.8	17.9	16.7	17.5
2022-08-24 1:00 AM	17.5	17.6	16.5	17.2
2022-08-24 2:00 AM	17.2	17.3	16.0	16.8
2022-08-24 3:00 AM	16.8	16.7	15.5	16.3
2022-08-24 4:00 AM	16.5	16.4	15.2	16.0
2022-08-24 5:00 AM	16.5	16.5	15.3	16.1
2022-08-24 6:00 AM	16.7	16.7	15.6	16.3
2022-08-24 7:00 AM	16.8	16.9	15.7	16.5
2022-08-24 8:00 AM	16.8	16.9	15.9	16.5
2022-08-24 9:00 AM	17.0	17.0	16.1	16.7
2022-08-24 10:00 AM	17.3	17.1	16.4	16.9
2022-08-24 11:00 AM	17.5	17.6	16.9	17.3
2022-08-24 12:00 PM	17.7	17.7	17.2	17.5
2022-08-24 1:00 PM	17.7	17.9	17.3	17.6
2022-08-24 2:00 PM	17.8	17.9	17.4	17.7
2022-08-24 3:00 PM	17.9	17.8	17.4	17.7
2022-08-24 4:00 PM	18.0	17.8	17.4	17.7
2022-08-24 5:00 PM	18.1	18.0	17.6	17.9
2022-08-24 6:00 PM	18.1	18.0	17.5	17.9
2022-08-24 7:00 PM	18.0	18.2	17.6	17.9
2022-08-24 8:00 PM	18.0	18.1	17.6	17.9
2022-08-24 9:00 PM	17.8	17.8	17.3	17.6
2022-08-24 10:00 PM	17.6	17.7	17.1	17.5
2022-08-24 11:00 PM	17.5	17.5	16.8	17.3
2022-08-25 12:00 AM	17.3	17.1	16.3	16.9
2022-08-25 1:00 AM	16.9	16.7	15.8	16.5
2022-08-25 2:00 AM	16.9	16.1	15.4	16.1
2022-08-25 3:00 AM	16.8	15.9	15.3	16.0
2022-08-25 4:00 AM	16.7	16.4	15.5	16.2
2022-08-25 5:00 AM	16.6	16.0	15.3	16.0
2022-08-25 6:00 AM	16.3	15.6	14.8	15.6
2022-08-25 7:00 AM	16.3	15.9	15.1	15.8
2022-08-25 8:00 AM	16.3	15.7	14.9	15.6
2022-08-25 9:00 AM	16.3	15.7	14.9	15.6

2022-08-25 10:00 AM	16.8	16.5	15.8	16.4
2022-08-25 11:00 AM	17.2	17.0	16.6	16.9
2022-08-25 12:00 PM	17.7	17.7	17.5	17.6
2022-08-25 1:00 PM	18.2	18.8	19.3	18.8
2022-08-25 2:00 PM	18.2	19.1	19.4	18.9
2022-08-25 3:00 PM	18.2	18.8	18.9	18.6
2022-08-25 4:00 PM	18.3	19.2	19.1	18.9
2022-08-25 5:00 PM	18.4	18.9	18.9	18.7
2022-08-25 6:00 PM	18.4	18.8	18.6	18.6
2022-08-25 7:00 PM	18.4	18.7	18.5	18.5
2022-08-25 8:00 PM	18.1	18.3	17.9	18.1
2022-08-25 9:00 PM	17.9	18.0	17.4	17.8
2022-08-25 10:00 PM	17.7	17.8	17.2	17.6
2022-08-25 11:00 PM	17.6	17.7	17.0	17.4
2022-08-26 12:00 AM	17.5	17.5	16.8	17.3
2022-08-26 1:00 AM	17.4	17.4	16.6	17.1
2022-08-26 2:00 AM	17.4	17.4	16.6	17.1
2022-08-26 3:00 AM	17.1	17.1	16.2	16.8
2022-08-26 4:00 AM	16.6	16.6	15.5	16.2
2022-08-26 5:00 AM	16.6	16.7	15.7	16.3
2022-08-26 6:00 AM	16.6	16.6	15.6	16.3
2022-08-26 7:00 AM	16.4	16.4	15.3	16.0
2022-08-26 8:00 AM	16.5	16.5	15.6	16.2
2022-08-26 9:00 AM	16.8	16.8	16.0	16.5
2022-08-26 10:00 AM	17.2	17.3	16.6	17.0
2022-08-26 11:00 AM	17.8	18.0	17.7	17.8
2022-08-26 12:00 PM	18.4	18.7	18.9	18.7
2022-08-26 1:00 PM	18.5	19.4	19.9	19.3
2022-08-26 2:00 PM	18.5	19.8	20.3	19.5
2022-08-26 3:00 PM	18.8	19.8	20.3	19.6
2022-08-26 4:00 PM	18.9	20.1	20.5	19.8
2022-08-26 5:00 PM	19.1	20.1	20.5	19.9
2022-08-26 6:00 PM	19.4	20.5	21.0	20.3
2022-08-26 7:00 PM	19.4	21.3	21.6	20.8
2022-08-26 8:00 PM	19.3	20.1	20.4	19.9
2022-08-26 9:00 PM	19.1	19.6	19.6	19.4
2022-08-26 10:00 PM	18.7	19.2	18.7	18.9
2022-08-26 11:00 PM	18.5	18.9	18.2	18.5
2022-08-27 12:00 AM	18.2	18.7	17.8	18.2
2022-08-27 1:00 AM	18.0	18.3	17.3	17.9
2022-08-27 2:00 AM	17.8	18.1	17.1	17.7
2022-08-27 3:00 AM	17.7	18.0	16.9	17.5
2022-08-27 4:00 AM	17.5	17.7	16.5	17.2

2022-08-27 5:00 AM	17.3	17.4	16.3	17.0
2022-08-27 6:00 AM	17.0	17.1	16.0	16.7
2022-08-27 7:00 AM	16.8	16.9	15.7	16.5
2022-08-27 8:00 AM	16.7	16.6	15.5	16.3
2022-08-27 9:00 AM	17.0	17.2	16.1	16.8
2022-08-27 10:00 AM	17.9	18.2	17.6	17.9
2022-08-27 11:00 AM	18.8	19.1	19.0	19.0
2022-08-27 12:00 PM	19.1	19.6	19.9	19.5
2022-08-27 1:00 PM	19.3	20.0	20.7	20.0
2022-08-27 2:00 PM	19.1	19.7	20.0	19.6
2022-08-27 3:00 PM	19.1	19.8	19.9	19.6
2022-08-27 4:00 PM	19.0	19.3	19.2	19.2
2022-08-27 5:00 PM	18.7	18.9	18.5	18.7
2022-08-27 6:00 PM	18.8	19.0	18.5	18.8
2022-08-27 7:00 PM	18.7	18.9	18.4	18.7
2022-08-27 8:00 PM	18.6	18.8	18.3	18.6
2022-08-27 9:00 PM	18.6	18.8	18.3	18.6
2022-08-27 10:00 PM	18.6	18.8	18.2	18.5
2022-08-27 11:00 PM	18.6	18.8	18.3	18.6
2022-08-28 12:00 AM	18.5	18.7	18.1	18.4
2022-08-28 1:00 AM	18.4	18.6	17.9	18.3
2022-08-28 2:00 AM	18.5	18.7	18.2	18.5
2022-08-28 3:00 AM	18.5	18.7	18.3	18.5
2022-08-28 4:00 AM	18.5	18.7	18.3	18.5
2022-08-28 5:00 AM	18.5	18.7	18.3	18.5
2022-08-28 6:00 AM	18.5	18.8	18.4	18.6
2022-08-28 7:00 AM	18.3	18.6	18.0	18.3
2022-08-28 8:00 AM	18.3	18.5	17.9	18.2
2022-08-28 9:00 AM	18.5	18.7	18.2	18.5
2022-08-28 10:00 AM	18.6	18.9	18.5	18.7
2022-08-28 11:00 AM	19.0	19.3	19.1	19.1
2022-08-28 12:00 PM	19.4	19.8	19.9	19.7
2022-08-28 1:00 PM	19.7	20.3	20.5	20.2
2022-08-28 2:00 PM	19.6	20.1	20.2	20.0
2022-08-28 3:00 PM	19.7	20.2	20.4	20.1
2022-08-28 4:00 PM	19.9	20.4	20.6	20.3
2022-08-28 5:00 PM	19.7	20.0	20.0	19.9
2022-08-28 6:00 PM	19.6	19.9	19.8	19.8
2022-08-28 7:00 PM	19.7	20.0	19.8	19.8
2022-08-28 8:00 PM	19.6	19.9	19.7	19.7
2022-08-28 9:00 PM	19.4	19.5	19.1	19.3
2022-08-28 10:00 PM	19.2	19.4	18.9	19.2
2022-08-28 11:00 PM	19.1	19.2	18.6	19.0

2022-08-29 12:00 AM	18.8	19.0	18.2	18.7
2022-08-29 1:00 AM	18.5	18.6	17.7	18.3
2022-08-29 2:00 AM	18.2	18.4	17.4	18.0
2022-08-29 3:00 AM	18.5	18.6	17.8	18.3
2022-08-29 4:00 AM	18.5	18.7	18.0	18.4
2022-08-29 5:00 AM	18.5	18.7	17.9	18.4
2022-08-29 6:00 AM	18.6	18.7	18.0	18.4
2022-08-29 7:00 AM	18.6	18.7	18.0	18.4
2022-08-29 8:00 AM	18.4	18.6	17.8	18.3
2022-08-29 9:00 AM	18.6	18.8	18.2	18.5
2022-08-29 10:00 AM	18.9	19.1	18.6	18.9
2022-08-29 11:00 AM	19.6	19.8	19.7	19.7
2022-08-29 12:00 PM	20.1	20.6	20.9	20.5
2022-08-29 1:00 PM	19.9	20.8	21.1	20.6
2022-08-29 2:00 PM	19.7	20.7	20.6	20.3
2022-08-29 3:00 PM	19.6	20.2	20.0	19.9
2022-08-29 4:00 PM	19.4	19.9	19.7	19.7
2022-08-29 5:00 PM	19.6	20.0	19.9	19.8
2022-08-29 6:00 PM	19.7	20.0	20.0	19.9
2022-08-29 7:00 PM	19.5	19.8	19.6	19.6
2022-08-29 8:00 PM	19.6	19.9	19.6	19.7
2022-08-29 9:00 PM	19.2	19.4	18.7	19.1
2022-08-29 10:00 PM	18.9	19.1	18.2	18.7
2022-08-29 11:00 PM	18.8	18.9	17.9	18.5
2022-08-30 12:00 AM	18.8	18.9	18.0	18.6
2022-08-30 1:00 AM	18.4	18.4	17.4	18.1
2022-08-30 2:00 AM	18.0	18.1	16.8	17.6
2022-08-30 3:00 AM	17.9	17.9	16.7	17.5
2022-08-30 4:00 AM	17.9	17.9	16.7	17.5
2022-08-30 5:00 AM	17.9	17.9	16.8	17.5
2022-08-30 6:00 AM	17.7	17.6	16.7	17.3
2022-08-30 7:00 AM	17.5	17.1	16.3	17.0
2022-08-30 8:00 AM	17.0	16.4	15.4	16.3
2022-08-30 9:00 AM	16.5	15.9	14.8	15.7
2022-08-30 10:00 AM	16.7	16.3	15.3	16.1
2022-08-30 11:00 AM	17.7	17.2	16.6	17.2
2022-08-30 12:00 PM	18.4	18.2	18.1	18.2
2022-08-30 1:00 PM	18.5	19.2	19.3	19.0
2022-08-30 2:00 PM	18.2	19.5	19.2	19.0
2022-08-30 3:00 PM	18.2	19.5	19.3	19.0
2022-08-30 4:00 PM	18.2	19.9	19.6	19.2
2022-08-30 5:00 PM	18.7	20.1	20.0	19.6
2022-08-30 6:00 PM	18.6	19.9	19.7	19.4

2022-08-30 7:00 PM	18.6	20.4	20.0	19.7
2022-08-30 8:00 PM	18.4	19.3	18.9	18.9
2022-08-30 9:00 PM	18.0	18.6	17.8	18.1
2022-08-30 10:00 PM	17.6	18.1	16.9	17.5
2022-08-30 11:00 PM	17.3	17.8	16.4	17.2
2022-08-31 12:00 AM	17.2	17.5	16.1	16.9
2022-08-31 1:00 AM	17.1	17.3	15.9	16.8
2022-08-31 2:00 AM	16.8	17.1	15.7	16.5
2022-08-31 3:00 AM	16.7	16.9	15.5	16.4
2022-08-31 4:00 AM	16.5	16.7	15.2	16.1
2022-08-31 5:00 AM	16.2	16.4	14.9	15.8
2022-08-31 6:00 AM	15.9	16.1	14.5	15.5
2022-08-31 7:00 AM	15.7	15.8	14.2	15.2
2022-08-31 8:00 AM	15.4	15.3	13.7	14.8
2022-08-31 9:00 AM	15.3	15.3	13.6	14.7
2022-08-31 10:00 AM	15.6	15.4	14.1	15.0
2022-08-31 11:00 AM	16.7	16.2	15.3	16.1
2022-08-31 12:00 PM	17.3	17.1	16.6	17.0
2022-08-31 1:00 PM	17.2	17.8	17.5	17.5
2022-08-31 2:00 PM	16.7	17.7	16.9	17.1
2022-08-31 3:00 PM	16.6	17.1	16.6	16.8
2022-08-31 4:00 PM	16.5	16.1	17.1	16.6
2022-08-31 5:00 PM	16.9	16.7	18.0	17.2
2022-08-31 6:00 PM	17.2	17.9	19.8	18.3
2022-08-31 7:00 PM	17.1	18.7	19.8	18.5
2022-08-31 8:00 PM	16.6	17.2	17.1	17.0
2022-08-31 9:00 PM	16.3	16.8	15.6	16.2
2022-08-31 10:00 PM	16.0	16.3	14.5	15.6
2022-08-31 11:00 PM	15.8	16.1	14.2	15.4
2022-09-01 12:00 AM	15.7	16.0	14.3	15.3
2022-09-01 1:00 AM	16.0	16.2	14.6	15.6
2022-09-01 2:00 AM	16.0	16.1	14.6	15.6
2022-09-01 3:00 AM	15.4	15.4	13.3	14.7
2022-09-01 4:00 AM	15.2	15.2	13.0	14.5
2022-09-01 5:00 AM	15.3	15.1	13.1	14.5
2022-09-01 6:00 AM	15.0	14.9	12.7	14.2
2022-09-01 7:00 AM	14.8	14.6	12.4	13.9
2022-09-01 8:00 AM	14.7	14.6	12.5	13.9
2022-09-01 9:00 AM	15.0	15.0	13.1	14.4
2022-09-01 10:00 AM	15.9	16.1	15.1	15.7
2022-09-01 11:00 AM	16.7	16.7	16.6	16.7
2022-09-01 12:00 PM	17.3	17.4	17.9	17.5
2022-09-01 1:00 PM	17.6	18.4	19.9	18.6

2022-09-01 2:00 PM	17.3	18.5	19.4	18.4
2022-09-01 3:00 PM	17.2	18.0	18.3	17.8
2022-09-01 4:00 PM	17.3	18.0	18.2	17.8
2022-09-01 5:00 PM	17.5	18.1	18.2	17.9
2022-09-01 6:00 PM	17.8	18.7	19.4	18.6
2022-09-01 7:00 PM	17.7	19.3	19.9	19.0
2022-09-01 8:00 PM	17.5	18.2	18.2	18.0
2022-09-01 9:00 PM	17.3	17.7	17.2	17.4
2022-09-01 10:00 PM	17.0	17.4	16.5	17.0
2022-09-01 11:00 PM	16.6	17.0	15.6	16.4
2022-09-02 12:00 AM	16.3	16.5	14.8	15.9
2022-09-02 1:00 AM	16.0	16.2	14.3	15.5
2022-09-02 2:00 AM	15.8	15.9	14.0	15.2
2022-09-02 3:00 AM	15.7	15.8	13.8	15.1
2022-09-02 4:00 AM	15.6	15.7	13.7	15.0
2022-09-02 5:00 AM	15.5	15.5	13.5	14.8
2022-09-02 6:00 AM	15.4	15.5	13.5	14.8
2022-09-02 7:00 AM	15.3	15.4	13.4	14.7
2022-09-02 8:00 AM	15.6	15.8	14.1	15.2
2022-09-02 9:00 AM	16.1	16.4	15.5	16.0
2022-09-02 10:00 AM	16.6	17.1	17.0	16.9
2022-09-02 11:00 AM	17.9	18.6	19.8	18.8
2022-09-02 12:00 PM	18.2	19.1	20.5	19.3
2022-09-02 1:00 PM	18.3	19.3	20.5	19.4
2022-09-02 2:00 PM	18.8	19.9	21.4	20.0
2022-09-02 3:00 PM	19.2	20.3	21.8	20.4
2022-09-02 4:00 PM	19.4	20.4	21.9	20.6
2022-09-02 5:00 PM	19.3	19.8	21.5	20.2
2022-09-02 6:00 PM	19.0	20.1	22.2	20.4
2022-09-02 7:00 PM	18.8	20.3	22.0	20.4
2022-09-02 8:00 PM	18.2	18.9	19.9	19.0
2022-09-02 9:00 PM	17.9	18.5	18.6	18.3
2022-09-02 10:00 PM	17.6	18.1	17.5	17.7
2022-09-02 11:00 PM	17.0	17.6	16.3	17.0
2022-09-03 12:00 AM	16.6	17.1	15.5	16.4
2022-09-03 1:00 AM	16.2	16.6	14.8	15.9
2022-09-03 2:00 AM	16.1	15.8	14.4	15.4
2022-09-03 3:00 AM	15.6	15.0	13.4	14.7
2022-09-03 4:00 AM	15.0	14.4	12.4	13.9
2022-09-03 5:00 AM	14.4	13.7	11.4	13.2
2022-09-03 6:00 AM	13.6	13.4	10.6	12.5
2022-09-03 7:00 AM	12.9	12.7	9.4	11.7
2022-09-03 8:00 AM	12.6	12.4	9.2	11.4

2022-09-03 9:00 AM	13.0	12.4	9.9	11.8
2022-09-03 10:00 AM	13.8	12.8	11.1	12.6
2022-09-03 11:00 AM	14.8	13.4	12.5	13.6
2022-09-03 12:00 PM	15.4	14.5	14.2	14.7
2022-09-03 1:00 PM	15.5	15.6	15.6	15.6
2022-09-03 2:00 PM	15.2	15.5	15.1	15.3
2022-09-03 3:00 PM	15.2	15.6	15.0	15.3
2022-09-03 4:00 PM	15.5	16.0	15.6	15.7
2022-09-03 5:00 PM	15.8	16.0	15.7	15.8
2022-09-03 6:00 PM	15.9	16.6	16.2	16.2
2022-09-03 7:00 PM	15.6	16.6	15.7	16.0
2022-09-03 8:00 PM	15.3	15.7	14.7	15.2
2022-09-03 9:00 PM	14.7	15.0	13.3	14.3
2022-09-03 10:00 PM	14.1	14.0	12.1	13.4
2022-09-03 11:00 PM	13.7	13.6	11.3	12.9
2022-09-04 12:00 AM	13.0	13.2	10.7	12.3
2022-09-04 1:00 AM	12.5	12.7	10.1	11.8
2022-09-04 2:00 AM	12.0	11.9	8.9	10.9
2022-09-04 3:00 AM	11.6	11.3	8.4	10.4
2022-09-04 4:00 AM	11.1	10.9	7.6	9.9
2022-09-04 5:00 AM	10.8	10.4	7.2	9.5
2022-09-04 6:00 AM	10.4	10.3	6.8	9.2
2022-09-04 7:00 AM	9.8	9.6	6.1	8.5
2022-09-04 8:00 AM	9.8	9.6	6.1	8.5
2022-09-04 9:00 AM	10.4	10.2	7.4	9.3
2022-09-04 10:00 AM	11.8	12.1	10.1	11.3
2022-09-04 11:00 AM	13.8	13.9	13.9	13.9
2022-09-04 12:00 PM	14.9	15.4	16.9	15.7
2022-09-04 1:00 PM	15.2	16.3	18.2	16.6
2022-09-04 2:00 PM	14.8	16.1	16.8	15.9
2022-09-04 3:00 PM	14.7	15.6	15.9	15.4
2022-09-04 4:00 PM	14.8	15.6	16.0	15.5
2022-09-04 5:00 PM	14.9	15.5	15.5	15.3
2022-09-04 6:00 PM	15.1	16.1	15.7	15.6
2022-09-04 7:00 PM	14.8	16.0	15.2	15.3
2022-09-04 8:00 PM	14.5	14.9	14.1	14.5
2022-09-04 9:00 PM	13.8	14.2	12.6	13.5
2022-09-04 10:00 PM	13.1	13.4	11.4	12.6
2022-09-04 11:00 PM	12.7	12.7	10.6	12.0
2022-09-05 12:00 AM	12.1	12.2	9.8	11.4
2022-09-05 1:00 AM	11.6	11.7	9.1	10.8
2022-09-05 2:00 AM	11.2	11.4	8.6	10.4
2022-09-05 3:00 AM	11.0	10.9	8.1	10.0

2022-09-05 4:00 AM	10.7	10.4	7.7	9.6
2022-09-05 5:00 AM	10.2	10.2	7.0	9.1
2022-09-05 6:00 AM	9.7	9.5	6.2	8.5
2022-09-05 7:00 AM	9.6	9.3	6.1	8.3
2022-09-05 8:00 AM	9.2	9.0	5.6	7.9
2022-09-05 9:00 AM	9.6	9.8	7.0	8.8
2022-09-05 10:00 AM	11.1	11.4	9.6	10.7
2022-09-05 11:00 AM	13.2	13.3	13.5	13.3
2022-09-05 12:00 PM	14.3	14.7	16.2	15.1
2022-09-05 1:00 PM	14.7	15.8	17.9	16.1
2022-09-05 2:00 PM	14.3	15.8	16.6	15.6
2022-09-05 3:00 PM	14.2	15.2	15.8	15.1
2022-09-05 4:00 PM	14.4	15.4	16.4	15.4
2022-09-05 5:00 PM	14.8	15.6	16.4	15.6
2022-09-05 6:00 PM	15.0	16.2	16.5	15.9
2022-09-05 7:00 PM	14.8	16.3	16.2	15.8
2022-09-05 8:00 PM	14.6	15.4	15.1	15.0
2022-09-05 9:00 PM	14.1	14.8	13.7	14.2
2022-09-05 10:00 PM	13.7	14.2	12.6	13.5
2022-09-05 11:00 PM	13.3	13.7	12.0	13.0
2022-09-06 12:00 AM	12.9	13.3	11.5	12.6
2022-09-06 1:00 AM	12.7	13.0	11.0	12.2
2022-09-06 2:00 AM	12.3	12.5	10.4	11.7
2022-09-06 3:00 AM	12.1	12.2	10.1	11.5
2022-09-06 4:00 AM	11.8	11.9	9.6	11.1
2022-09-06 5:00 AM	11.4	11.5	8.9	10.6
2022-09-06 6:00 AM	10.9	11.0	8.1	10.0
2022-09-06 7:00 AM	10.6	10.6	7.7	9.6
2022-09-06 8:00 AM	10.5	10.5	7.6	9.5
2022-09-06 9:00 AM	10.8	11.0	8.4	10.1
2022-09-06 10:00 AM	12.1	12.5	11.1	11.9
2022-09-06 11:00 AM	14.0	14.2	14.6	14.3
2022-09-06 12:00 PM	15.1	15.7	17.4	16.1
2022-09-06 1:00 PM	15.5	16.9	19.0	17.1
2022-09-06 2:00 PM	15.4	16.7	17.6	16.6
2022-09-06 3:00 PM	15.4	16.4	16.8	16.2
2022-09-06 4:00 PM	15.7	16.7	18.3	16.9
2022-09-06 5:00 PM	16.2	17.2	19.1	17.5
2022-09-06 6:00 PM	16.4	17.6	18.9	17.6
2022-09-06 7:00 PM	16.3	17.5	18.5	17.4
2022-09-06 8:00 PM	16.1	16.9	17.5	16.8
2022-09-06 9:00 PM	15.8	16.5	16.3	16.2
2022-09-06 10:00 PM	15.6	16.1	15.5	15.7

2022-09-06 11:00 PM	15.6	15.9	15.3	15.6
2022-09-07 12:00 AM	15.5	15.8	15.0	15.4
2022-09-07 1:00 AM	15.1	15.5	14.5	15.0
2022-09-07 2:00 AM	14.9	15.2	14.0	14.7
2022-09-07 3:00 AM	14.5	14.9	13.3	14.2
2022-09-07 4:00 AM	14.3	14.7	13.0	14.0
2022-09-07 5:00 AM	14.2	14.5	12.8	13.8
2022-09-07 6:00 AM	14.0	14.4	12.6	13.7
2022-09-07 7:00 AM	13.7	14.1	12.3	13.4
2022-09-07 8:00 AM	13.4	13.7	11.8	13.0
2022-09-07 9:00 AM	13.7	14.1	12.4	13.4
2022-09-07 10:00 AM	14.7	15.1	14.5	14.8
2022-09-07 11:00 AM	16.1	16.4	16.8	16.4
2022-09-07 12:00 PM	16.8	17.7	19.4	18.0
2022-09-07 1:00 PM	17.2	18.7	21.5	19.1
2022-09-07 2:00 PM	16.7	18.5	20.0	18.4
2022-09-07 3:00 PM	16.5	17.9	19.0	17.8
2022-09-07 4:00 PM	16.7	18.1	20.2	18.3
2022-09-07 5:00 PM	17.0	18.2	20.0	18.4
2022-09-07 6:00 PM	17.1	18.7	20.4	18.7
2022-09-07 7:00 PM	16.9	18.5	19.6	18.3
2022-09-07 8:00 PM	16.5	17.5	17.7	17.2
2022-09-07 9:00 PM	15.8	16.6	15.7	16.0
2022-09-07 10:00 PM	15.3	15.8	14.5	15.2
2022-09-07 11:00 PM	14.8	15.2	13.6	14.5
2022-09-08 12:00 AM	14.3	14.7	12.8	13.9
2022-09-08 1:00 AM	13.9	14.1	12.2	13.4
2022-09-08 2:00 AM	13.6	13.5	11.5	12.9
2022-09-08 3:00 AM	13.2	13.0	11.0	12.4
2022-09-08 4:00 AM	12.7	12.9	10.5	12.0
2022-09-08 5:00 AM	12.4	12.7	10.2	11.8
2022-09-08 6:00 AM	12.2	12.4	9.9	11.5
2022-09-08 7:00 AM	12.2	12.6	10.2	11.7
2022-09-08 8:00 AM	12.4	12.6	10.5	11.8
2022-09-08 9:00 AM	13.0	13.2	11.5	12.6
2022-09-08 10:00 AM	14.4	14.7	14.4	14.5
2022-09-08 11:00 AM	15.5	16.0	16.7	16.1
2022-09-08 12:00 PM	16.6	17.3	19.4	17.8
2022-09-08 1:00 PM	17.3	18.4	21.0	18.9
2022-09-08 2:00 PM	17.6	18.6	20.6	18.9
2022-09-08 3:00 PM	17.6	18.3	19.8	18.6
2022-09-08 4:00 PM	17.9	18.7	20.6	19.1
2022-09-08 5:00 PM	18.0	18.6	20.2	18.9

2022-09-08 6:00 PM	18.0	18.4	19.7	18.7
2022-09-08 7:00 PM	17.8	18.3	19.1	18.4
2022-09-08 8:00 PM	17.7	18.1	18.7	18.2
2022-09-08 9:00 PM	17.5	17.9	18.0	17.8
2022-09-08 10:00 PM	17.5	17.9	18.0	17.8
2022-09-08 11:00 PM	17.5	17.8	17.8	17.7
2022-09-09 12:00 AM	17.5	17.8	17.8	17.7
2022-09-09 1:00 AM	17.4	17.7	17.6	17.6
2022-09-09 2:00 AM	17.4	17.7	17.6	17.6
2022-09-09 3:00 AM	17.4	17.8	17.6	17.6
2022-09-09 4:00 AM	17.5	17.9	17.9	17.8
2022-09-09 5:00 AM	17.4	17.7	17.6	17.6
2022-09-09 6:00 AM	17.3	17.6	17.3	17.4
2022-09-09 7:00 AM	17.2	17.4	17.0	17.2
2022-09-09 8:00 AM	17.0	17.2	16.5	16.9
2022-09-09 9:00 AM	16.8	16.9	16.2	16.6
2022-09-09 10:00 AM	16.8	16.8	16.1	16.6
2022-09-09 11:00 AM	16.8	16.7	16.0	16.5
2022-09-09 12:00 PM	16.5	16.5	15.6	16.2
2022-09-09 1:00 PM	16.3	16.0	15.2	15.8
2022-09-09 2:00 PM	16.4	15.8	15.2	15.8
2022-09-09 3:00 PM	16.4	16.1	15.3	15.9
2022-09-09 4:00 PM	16.5	16.1	15.1	15.9
2022-09-09 5:00 PM	16.4	16.1	15.3	15.9
2022-09-09 6:00 PM	16.3	16.0	15.3	15.9
2022-09-09 7:00 PM	16.3	16.2	15.3	15.9
2022-09-09 8:00 PM	16.0	16.0	15.0	15.7
2022-09-09 9:00 PM	15.7	15.7	14.6	15.3
2022-09-09 10:00 PM	15.7	15.7	14.6	15.3
2022-09-09 11:00 PM	15.5	15.5	14.4	15.1
2022-09-10 12:00 AM	15.5	15.5	14.4	15.1
2022-09-10 1:00 AM	15.3	15.3	14.2	14.9
2022-09-10 2:00 AM	15.3	15.2	14.1	14.9
2022-09-10 3:00 AM	15.3	15.2	14.1	14.9
2022-09-10 4:00 AM	15.1	15.1	13.8	14.7
2022-09-10 5:00 AM	15.0	15.1	13.8	14.6
2022-09-10 6:00 AM	14.5	14.6	13.0	14.0
2022-09-10 7:00 AM	14.5	14.5	13.0	14.0
2022-09-10 8:00 AM	14.2	14.1	12.5	13.6
2022-09-10 9:00 AM	14.4	14.4	12.9	13.9
2022-09-10 10:00 AM	15.1	15.2	14.2	14.8
2022-09-10 11:00 AM	15.5	15.5	15.0	15.3
2022-09-10 12:00 PM	15.9	16.1	15.8	15.9

2022-09-10 1:00 PM	16.2	16.5	16.6	16.4
2022-09-10 2:00 PM	16.3	16.5	16.7	16.5
2022-09-10 3:00 PM	16.4	16.7	16.8	16.6
2022-09-10 4:00 PM	16.4	16.8	16.9	16.7
2022-09-10 5:00 PM	16.4	16.6	16.5	16.5
2022-09-10 6:00 PM	16.1	16.3	16.0	16.1
2022-09-10 7:00 PM	16.0	16.3	16.0	16.1
2022-09-10 8:00 PM	15.7	16.0	15.4	15.7
2022-09-10 9:00 PM	15.1	15.2	14.0	14.8
2022-09-10 10:00 PM	14.7	14.6	13.2	14.2
2022-09-10 11:00 PM	14.3	14.3	12.6	13.7
2022-09-11 12:00 AM	14.1	14.2	12.5	13.6
2022-09-11 1:00 AM	13.8	13.8	12.0	13.2
2022-09-11 2:00 AM	13.3	13.2	11.3	12.6
2022-09-11 3:00 AM	12.9	12.7	10.6	12.1
2022-09-11 4:00 AM	12.5	12.2	10.1	11.6
2022-09-11 5:00 AM	12.1	11.8	9.5	11.1
2022-09-11 6:00 AM	11.9	11.6	9.2	10.9
2022-09-11 7:00 AM	11.6	11.2	9.0	10.6
2022-09-11 8:00 AM	11.4	11.0	8.8	10.4
2022-09-11 9:00 AM	11.5	11.3	9.0	10.6
2022-09-11 10:00 AM	12.7	12.8	11.1	12.2
2022-09-11 11:00 AM	14.1	14.2	13.6	14.0
2022-09-11 12:00 PM	15.0	15.5	16.3	15.6
2022-09-11 1:00 PM	15.2	16.2	17.7	16.4
2022-09-11 2:00 PM	15.0	16.1	16.7	15.9
2022-09-11 3:00 PM	15.2	16.2	16.9	16.1
2022-09-11 4:00 PM	15.4	16.6	17.4	16.5
2022-09-11 5:00 PM	15.6	16.5	17.1	16.4
2022-09-11 6:00 PM	15.7	16.4	16.8	16.3
2022-09-11 7:00 PM	15.5	16.3	16.5	16.1
2022-09-11 8:00 PM	15.3	15.9	15.7	15.6
2022-09-11 9:00 PM	14.8	15.3	14.3	14.8
2022-09-11 10:00 PM	14.4	14.7	13.4	14.2
2022-09-11 11:00 PM	14.0	14.3	12.7	13.7
2022-09-12 12:00 AM	13.6	13.8	12.0	13.1
2022-09-12 1:00 AM	13.1	13.2	11.2	12.5
2022-09-12 2:00 AM	12.8	12.6	10.7	12.0
2022-09-12 3:00 AM	12.2	12.1	9.9	11.4
2022-09-12 4:00 AM	11.9	11.7	9.5	11.0
2022-09-12 5:00 AM	11.7	11.4	9.2	10.8
2022-09-12 6:00 AM	11.4	11.2	9.0	10.5
2022-09-12 7:00 AM	11.1	10.9	8.6	10.2

2022-09-12 8:00 AM	10.9	10.6	8.3	9.9
2022-09-12 9:00 AM	11.2	11.0	8.8	10.3
2022-09-12 10:00 AM	12.6	12.7	11.2	12.2
2022-09-12 11:00 AM	13.9	14.0	13.6	13.8
2022-09-12 12:00 PM	14.6	15.1	15.9	15.2
2022-09-12 1:00 PM	15.0	16.3	17.6	16.3
2022-09-12 2:00 PM	15.0	16.4	17.6	16.3
2022-09-12 3:00 PM	15.0	16.2	17.5	16.2
2022-09-12 4:00 PM	15.2	16.4	17.2	16.3
2022-09-12 5:00 PM	15.4	16.2	16.4	16.0
2022-09-12 6:00 PM	15.5	16.3	16.8	16.2
2022-09-12 7:00 PM	15.5	16.2	16.6	16.1
2022-09-12 8:00 PM	15.4	15.9	16.0	15.8
2022-09-12 9:00 PM	15.0	15.5	14.9	15.1
2022-09-12 10:00 PM	14.7	15.1	14.0	14.6
2022-09-12 11:00 PM	14.3	14.7	13.3	14.1
2022-09-13 12:00 AM	13.9	14.2	12.7	13.6
2022-09-13 1:00 AM	13.7	13.7	12.2	13.2
2022-09-13 2:00 AM	13.3	13.4	11.7	12.8
2022-09-13 3:00 AM	13.0	13.0	11.3	12.4
2022-09-13 4:00 AM	12.5	12.7	10.6	11.9
2022-09-13 5:00 AM	12.3	12.4	10.4	11.7
2022-09-13 6:00 AM	12.0	12.0	10.0	11.3
2022-09-13 7:00 AM	12.1	12.2	10.2	11.5
2022-09-13 8:00 AM	12.1	12.3	10.3	11.6
2022-09-13 9:00 AM	12.8	13.1	11.5	12.5
2022-09-13 10:00 AM	13.8	14.0	13.3	13.7
2022-09-13 11:00 AM	14.8	15.1	14.9	14.9
2022-09-13 12:00 PM	15.1	16.2	16.5	15.9
2022-09-13 1:00 PM	15.3	16.9	17.1	16.4
2022-09-13 2:00 PM	15.3	16.7	16.5	16.2
2022-09-13 3:00 PM	15.3	16.8	16.7	16.3
2022-09-13 4:00 PM	15.5	16.8	16.6	16.3
2022-09-13 5:00 PM	15.5	16.3	16.1	16.0
2022-09-13 6:00 PM	15.2	15.7	15.3	15.4
2022-09-13 7:00 PM	15.1	15.4	15.0	15.2
2022-09-13 8:00 PM	14.6	15.0	14.1	14.6
2022-09-13 9:00 PM	13.9	14.1	12.4	13.5
2022-09-13 10:00 PM	13.6	13.3	11.8	12.9
2022-09-13 11:00 PM	13.4	12.4	11.4	12.4
2022-09-14 12:00 AM	12.7	12.2	10.6	11.8
2022-09-14 1:00 AM	11.9	11.7	9.5	11.0
2022-09-14 2:00 AM	11.5	11.3	8.9	10.6

2022-09-14 3:00 AM	11.2	11.0	8.6	10.3
2022-09-14 4:00 AM	11.0	11.0	8.5	10.2
2022-09-14 5:00 AM	10.5	10.2	7.7	9.5
2022-09-14 6:00 AM	10.1	9.6	7.0	8.9
2022-09-14 7:00 AM	9.6	9.3	6.5	8.5
2022-09-14 8:00 AM	9.3	8.9	6.2	8.1
2022-09-14 9:00 AM	9.6	9.3	6.8	8.6
2022-09-14 10:00 AM	10.9	10.9	8.9	10.2
2022-09-14 11:00 AM	12.8	12.7	12.2	12.6
2022-09-14 12:00 PM	13.4	13.8	14.6	13.9
2022-09-14 1:00 PM	13.7	14.8	15.9	14.8
2022-09-14 2:00 PM	13.7	14.7	14.9	14.4
2022-09-14 3:00 PM	13.4	14.3	14.2	14.0
2022-09-14 4:00 PM	13.4	14.3	14.3	14.0
2022-09-14 5:00 PM	13.6	14.3	14.2	14.0
2022-09-14 6:00 PM	13.4	14.0	13.5	13.6
2022-09-14 7:00 PM	13.1	13.6	12.7	13.1
2022-09-14 8:00 PM	13.1	13.4	12.4	13.0
2022-09-14 9:00 PM	12.8	13.0	11.7	12.5
2022-09-14 10:00 PM	12.1	12.3	10.5	11.6
2022-09-14 11:00 PM	11.7	11.6	9.8	11.0
2022-09-15 12:00 AM	11.4	11.0	9.3	10.6
2022-09-15 1:00 AM	11.2	11.2	9.2	10.5
2022-09-15 2:00 AM	11.7	11.9	10.4	11.3
2022-09-15 3:00 AM	12.0	12.2	11.1	11.8
2022-09-15 4:00 AM	12.2	12.4	11.3	12.0
2022-09-15 5:00 AM	12.4	12.6	11.6	12.2
2022-09-15 6:00 AM	12.5	12.7	11.8	12.3
2022-09-15 7:00 AM	12.5	12.8	11.7	12.3
2022-09-15 8:00 AM	12.6	12.8	11.9	12.4
2022-09-15 9:00 AM	12.8	13.1	12.5	12.8
2022-09-15 10:00 AM	12.9	13.1	12.4	12.8
2022-09-15 11:00 AM	13.0	13.2	12.5	12.9
2022-09-15 12:00 PM	13.2	13.4	12.9	13.2
2022-09-15 1:00 PM	13.4	13.7	13.3	13.5
2022-09-15 2:00 PM	13.5	13.8	13.4	13.6
2022-09-15 3:00 PM	13.6	14.0	13.7	13.8
2022-09-15 4:00 PM	13.8	14.1	14.0	14.0
2022-09-15 5:00 PM	13.8	14.1	14.0	14.0
2022-09-15 6:00 PM	14.0	14.3	14.2	14.2
2022-09-15 7:00 PM	14.0	14.3	14.1	14.1
2022-09-15 8:00 PM	14.0	14.3	14.0	14.1
2022-09-15 9:00 PM	14.0	14.2	13.9	14.0

2022-09-15 10:00 PM	14.0	14.2	13.9	14.0
2022-09-15 11:00 PM	14.0	14.3	13.9	14.1
2022-09-16 12:00 AM	14.0	14.1	13.6	13.9
2022-09-16 1:00 AM	14.0	14.1	13.6	13.9
2022-09-16 2:00 AM	14.0	14.1	13.6	13.9
2022-09-16 3:00 AM	13.9	13.9	13.2	13.7
2022-09-16 4:00 AM	13.8	13.6	12.8	13.4
2022-09-16 5:00 AM	13.6	13.2	12.5	13.1
2022-09-16 6:00 AM	13.6	13.2	12.4	13.1
2022-09-16 7:00 AM	13.5	13.1	12.2	12.9
2022-09-16 8:00 AM	13.3	12.7	11.8	12.6
2022-09-16 9:00 AM	13.2	12.7	11.8	12.6
2022-09-16 10:00 AM	13.3	13.0	12.1	12.8
2022-09-16 11:00 AM	13.4	13.1	12.3	12.9
2022-09-16 12:00 PM	13.4	13.3	12.5	13.1
2022-09-16 1:00 PM	13.5	13.5	12.7	13.2
2022-09-16 2:00 PM	13.6	13.5	12.9	13.3
2022-09-16 3:00 PM	13.6	13.5	12.8	13.3
2022-09-16 4:00 PM	13.6	13.5	12.8	13.3
2022-09-16 5:00 PM	13.6	13.5	12.8	13.3
2022-09-16 6:00 PM	13.6	13.5	12.9	13.3
2022-09-16 7:00 PM	13.5	13.5	12.9	13.3
2022-09-16 8:00 PM	13.5	13.5	12.9	13.3
2022-09-16 9:00 PM	13.5	13.6	13.0	13.4
2022-09-16 10:00 PM	13.6	13.8	13.3	13.6
2022-09-16 11:00 PM	13.7	13.9	13.5	13.7
2022-09-17 12:00 AM	13.8	14.0	13.6	13.8
2022-09-17 1:00 AM	13.9	14.2	13.7	13.9
2022-09-17 2:00 AM	14.0	14.2	13.8	14.0
2022-09-17 3:00 AM	14.0	14.2	13.8	14.0
2022-09-17 4:00 AM	14.0	14.2	13.9	14.0
2022-09-17 5:00 AM	14.0	14.3	13.9	14.1
2022-09-17 6:00 AM	14.1	14.3	13.9	14.1
2022-09-17 7:00 AM	14.1	14.3	13.9	14.1
2022-09-17 8:00 AM	14.1	14.3	13.9	14.1
2022-09-17 9:00 AM	14.3	14.4	14.2	14.3
2022-09-17 10:00 AM	14.5	14.7	14.6	14.6
2022-09-17 11:00 AM	14.7	14.9	14.9	14.8
2022-09-17 12:00 PM	14.9	15.2	15.3	15.1
2022-09-17 1:00 PM	15.2	15.5	15.8	15.5
2022-09-17 2:00 PM	15.5	15.8	16.2	15.8
2022-09-17 3:00 PM	15.6	15.9	16.2	15.9
2022-09-17 4:00 PM	15.6	15.8	16.0	15.8

2022-09-17 5:00 PM	15.5	15.7	15.8	15.7
2022-09-17 6:00 PM	15.5	15.7	15.8	15.7
2022-09-17 7:00 PM	15.4	15.6	15.7	15.6
2022-09-17 8:00 PM	15.4	15.6	15.5	15.5
2022-09-17 9:00 PM	15.3	15.5	15.4	15.4
2022-09-17 10:00 PM	15.3	15.5	15.3	15.4
2022-09-17 11:00 PM	15.3	15.5	15.3	15.4
2022-09-18 12:00 AM	15.3	15.5	15.3	15.4
2022-09-18 1:00 AM	15.3	15.5	15.4	15.4
2022-09-18 2:00 AM	15.3	15.5	15.4	15.4
2022-09-18 3:00 AM	15.3	15.5	15.3	15.4
2022-09-18 4:00 AM	15.3	15.5	15.3	15.4
2022-09-18 5:00 AM	15.3	15.5	15.2	15.3
2022-09-18 6:00 AM	15.3	15.5	15.2	15.3
2022-09-18 7:00 AM	15.3	15.6	15.2	15.4
2022-09-18 8:00 AM	15.3	15.5	15.1	15.3
2022-09-18 9:00 AM	15.3	15.4	15.0	15.2
2022-09-18 10:00 AM	15.4	15.5	15.2	15.4
2022-09-18 11:00 AM	15.6	15.8	15.6	15.7
2022-09-18 12:00 PM	15.8	16.0	15.8	15.9
2022-09-18 1:00 PM	16.2	16.5	16.5	16.4
2022-09-18 2:00 PM	16.7	17.0	17.5	17.1
2022-09-18 3:00 PM	16.7	17.1	17.6	17.1
2022-09-18 4:00 PM	16.8	17.2	18.0	17.3
2022-09-18 5:00 PM	16.8	17.2	17.6	17.2
2022-09-18 6:00 PM	16.9	17.4	17.9	17.4
2022-09-18 7:00 PM	16.9	17.2	17.7	17.3
2022-09-18 8:00 PM	16.6	16.8	16.9	16.8
2022-09-18 9:00 PM	16.3	16.4	16.2	16.3
2022-09-18 10:00 PM	16.0	16.1	15.6	15.9
2022-09-18 11:00 PM	15.5	15.6	14.6	15.2
2022-09-19 12:00 AM	15.1	15.0	13.8	14.6
2022-09-19 1:00 AM	14.8	14.5	13.2	14.2
2022-09-19 2:00 AM	14.6	14.3	12.9	13.9
2022-09-19 3:00 AM	14.3	13.9	12.5	13.6
2022-09-19 4:00 AM	14.1	13.6	12.1	13.3
2022-09-19 5:00 AM	14.2	14.0	12.5	13.6
2022-09-19 6:00 AM	14.0	13.6	12.1	13.2
2022-09-19 7:00 AM	13.8	13.5	12.0	13.1
2022-09-19 8:00 AM	13.7	13.5	11.8	13.0
2022-09-19 9:00 AM	13.8	13.6	12.1	13.2
2022-09-19 10:00 AM	14.6	14.7	13.9	14.4
2022-09-19 11:00 AM	15.7	15.7	15.7	15.7

2022-09-19 12:00 PM	16.0	16.4	17.1	16.5
2022-09-19 1:00 PM	16.1	17.2	18.3	17.2
2022-09-19 2:00 PM	16.2	17.2	18.1	17.2
2022-09-19 3:00 PM	16.3	17.3	18.6	17.4
2022-09-19 4:00 PM	16.6	18.0	18.7	17.8
2022-09-19 5:00 PM	16.8	18.1	18.7	17.9
2022-09-19 6:00 PM	16.7	17.9	18.1	17.6
2022-09-19 7:00 PM	16.4	17.3	17.1	16.9
2022-09-19 8:00 PM	16.2	16.6	16.2	16.3
2022-09-19 9:00 PM	15.7	15.9	15.2	15.6
2022-09-19 10:00 PM	15.4	15.6	14.6	15.2
2022-09-19 11:00 PM	15.3	15.4	14.4	15.0
2022-09-20 12:00 AM	15.4	15.5	14.7	15.2
2022-09-20 1:00 AM	15.5	15.5	14.8	15.3
2022-09-20 2:00 AM	15.5	15.5	14.7	15.2
2022-09-20 3:00 AM	15.3	15.4	14.5	15.1
2022-09-20 4:00 AM	15.4	15.6	14.7	15.2
2022-09-20 5:00 AM	15.4	15.5	14.7	15.2
2022-09-20 6:00 AM	15.2	15.4	14.5	15.0
2022-09-20 7:00 AM	15.3	15.6	14.8	15.2
2022-09-20 8:00 AM	15.4	15.6	14.9	15.3
2022-09-20 9:00 AM	15.5	15.7	15.1	15.4
2022-09-20 10:00 AM	15.8	16.0	15.5	15.8
2022-09-20 11:00 AM	16.2	16.4	16.3	16.3
2022-09-20 12:00 PM	16.6	17.0	17.8	17.1
2022-09-20 1:00 PM	16.8	18.0	19.8	18.2
2022-09-20 2:00 PM	16.8	18.0	18.7	17.8
2022-09-20 3:00 PM	16.9	18.0	18.6	17.8
2022-09-20 4:00 PM	17.4	18.6	19.4	18.5
2022-09-20 5:00 PM	17.4	18.3	18.7	18.1
2022-09-20 6:00 PM	17.2	18.0	17.9	17.7
2022-09-20 7:00 PM	17.1	17.8	17.8	17.6
2022-09-20 8:00 PM	17.0	17.6	17.5	17.4
2022-09-20 9:00 PM	16.8	17.3	17.0	17.0
2022-09-20 10:00 PM	16.4	16.8	16.2	16.5
2022-09-20 11:00 PM	16.4	16.8	16.2	16.5
2022-09-21 12:00 AM	16.4	16.8	16.3	16.5
2022-09-21 1:00 AM	16.4	16.7	16.3	16.5
2022-09-21 2:00 AM	16.2	16.5	16.0	16.2
2022-09-21 3:00 AM	16.0	16.2	15.5	15.9
2022-09-21 4:00 AM	15.9	16.0	15.3	15.7
2022-09-21 5:00 AM	15.7	15.8	15.0	15.5
2022-09-21 6:00 AM	15.4	15.5	14.5	15.1

2022-09-21 7:00 AM	14.9	14.6	13.5	14.3
2022-09-21 8:00 AM	14.5	14.2	12.8	13.8
2022-09-21 9:00 AM	14.5	14.3	12.9	13.9
2022-09-21 10:00 AM	15.0	15.0	14.1	14.7
2022-09-21 11:00 AM	15.5	15.2	14.8	15.2
2022-09-21 12:00 PM	15.5	15.1	15.2	15.3
2022-09-21 1:00 PM	15.3	15.0	15.0	15.1
2022-09-21 2:00 PM	15.4	15.3	15.1	15.3
2022-09-21 3:00 PM	15.3	14.3	13.9	14.5
2022-09-21 4:00 PM	15.0	13.9	13.4	14.1
2022-09-21 5:00 PM	14.9	13.9	13.5	14.1
2022-09-21 6:00 PM	14.5	13.2	12.7	13.5
2022-09-21 7:00 PM	13.9	12.4	11.8	12.7
2022-09-21 8:00 PM	13.6	12.0	11.3	12.3
2022-09-21 9:00 PM	13.2	11.6	10.9	11.9
2022-09-21 10:00 PM	12.8	11.9	10.8	11.8
2022-09-21 11:00 PM	12.6	11.9	10.6	11.7
2022-09-22 12:00 AM	12.4	11.7	10.4	11.5
2022-09-22 1:00 AM	12.1	11.0	9.7	10.9
2022-09-22 2:00 AM	12.0	10.5	9.4	10.6
2022-09-22 3:00 AM	11.7	10.5	9.2	10.5
2022-09-22 4:00 AM	11.1	10.2	8.5	9.9
2022-09-22 5:00 AM	11.2	9.8	8.6	9.9
2022-09-22 6:00 AM	11.2	10.2	8.8	10.1
2022-09-22 7:00 AM	11.3	10.1	8.9	10.1
2022-09-22 8:00 AM	11.0	8.9	8.1	9.3
2022-09-22 9:00 AM	10.6	9.2	7.9	9.2
2022-09-22 10:00 AM	10.8	8.4	7.9	9.0
2022-09-22 11:00 AM	11.8	9.3	9.1	10.1
2022-09-22 12:00 PM	12.1	10.6	10.9	11.2
2022-09-22 1:00 PM	12.2	11.6	12.1	12.0
2022-09-22 2:00 PM	12.1	11.6	11.1	11.6
2022-09-22 3:00 PM	11.9	11.7	11.0	11.5
2022-09-22 4:00 PM	12.0	12.6	12.1	12.2
2022-09-22 5:00 PM	12.4	13.0	12.6	12.7
2022-09-22 6:00 PM	12.3	12.7	11.7	12.2
2022-09-22 7:00 PM	12.1	12.6	11.4	12.0
2022-09-22 8:00 PM	11.8	12.0	10.5	11.4
2022-09-22 9:00 PM	11.3	11.1	9.5	10.6
2022-09-22 10:00 PM	10.7	10.3	8.6	9.9
2022-09-22 11:00 PM	10.3	9.7	8.0	9.3
2022-09-23 12:00 AM	9.8	9.1	7.3	8.7
2022-09-23 1:00 AM	9.4	8.4	6.7	8.2

2022-09-23 2:00 AM	9.1	8.4	6.3	7.9
2022-09-23 3:00 AM	8.9	8.0	6.1	7.7
2022-09-23 4:00 AM	8.7	7.7	5.8	7.4
2022-09-23 5:00 AM	8.6	7.5	5.7	7.3
2022-09-23 6:00 AM	8.5	7.1	5.5	7.0
2022-09-23 7:00 AM	8.1	6.9	5.1	6.7
2022-09-23 8:00 AM	7.9	6.0	4.5	6.1
2022-09-23 9:00 AM	7.9	7.0	4.9	6.6
2022-09-23 10:00 AM	9.4	8.9	7.4	8.6
2022-09-23 11:00 AM	11.3	11.1	10.7	11.0
2022-09-23 12:00 PM	11.8	12.2	13.3	12.4
2022-09-23 1:00 PM	12.3	13.1	14.2	13.2
2022-09-23 2:00 PM	12.7	13.5	14.1	13.4

Table S5: Hourly air temperature under 50% shade from May 10 to September 23, 2023.

	Port 1	Port 2	Port 3	Average
	5TE Moisture/ Temp/EC	5TE Moisture/ Temp/EC	5TE Moisture/ Temp/EC	5TE Moisture/ Temp/EC
Measurement Time	°C Temp	°C Temp	°C Temp	°C Temp
2022-05-11 1:53 PM	16.6	26.1	21.3	21.3
2022-05-11 2:00 PM	19.4	26.0	21.7	22.4
2022-05-11 3:00 PM	21.2	24.7	25.9	23.9
2022-05-11 4:00 PM	20.6	22.1	26.0	22.9
2022-05-11 5:00 PM	19.8	20.9	22.4	21.0
2022-05-11 6:00 PM	17.7	18.5	17.9	18.0
2022-05-11 7:00 PM	16.5	17.0	16.3	16.6
2022-05-11 8:00 PM	16.4	16.7	15.6	16.2
2022-05-11 9:00 PM	15.7	15.9	14.9	15.5
2022-05-11 10:00 PM	13.3	13.4	13.1	13.3
2022-05-11 11:00 PM	12.5	12.4	12.3	12.4
2022-05-12 12:00 AM	11.9	11.8	11.8	11.8
2022-05-12 1:00 AM	11.4	11.2	11.3	11.3
2022-05-12 2:00 AM	12.2	12.2	11.9	12.1
2022-05-12 3:00 AM	12.9	13.1	12.5	12.8
2022-05-12 4:00 AM	9.9	9.8	10.0	9.9
2022-05-12 5:00 AM	10.2	10.1	10.2	10.2
2022-05-12 6:00 AM	10.8	10.7	10.7	10.7
2022-05-12 7:00 AM	10.6	10.6	10.6	10.6
2022-05-12 8:00 AM	10.3	10.1	10.2	10.2
2022-05-12 9:00 AM	10.6	10.6	10.6	10.6

2022-05-12 10:00 AM	10.8	10.8	10.8	10.8
2022-05-12 11:00 AM	11.3	11.3	11.2	11.3
2022-05-12 12:00 PM	13.1	13.1	12.5	12.9
2022-05-12 1:00 PM	15.4	16.2	14.4	15.3
2022-05-12 2:00 PM	17.5	19.1	17.1	17.9
2022-05-12 3:00 PM	17.6	18.7	18.3	18.2
2022-05-12 4:00 PM	16.0	16.2	15.8	16.0
2022-05-12 5:00 PM	15.4	15.5	14.9	15.3
2022-05-12 6:00 PM	15.2	15.2	14.8	15.1
2022-05-12 7:00 PM	14.6	14.4	14.2	14.4
2022-05-12 8:00 PM	13.9	13.8	13.6	13.8
2022-05-12 9:00 PM	12.3	12.1	12.1	12.2
2022-05-12 10:00 PM	11.4	11.0	11.3	11.2
2022-05-12 11:00 PM	11.5	11.2	11.4	11.4
2022-05-13 12:00 AM	11.4	11.2	11.3	11.3
2022-05-13 1:00 AM	10.9	10.8	10.9	10.9
2022-05-13 2:00 AM	10.5	10.3	10.5	10.4
2022-05-13 3:00 AM	11.3	11.2	11.3	11.3
2022-05-13 4:00 AM	10.4	10.3	10.5	10.4
2022-05-13 5:00 AM	10.3	10.2	10.3	10.3
2022-05-13 6:00 AM	10.3	10.2	10.4	10.3
2022-05-13 7:00 AM	9.6	9.6	9.8	9.7
2022-05-13 8:00 AM	9.8	9.7	9.9	9.8
2022-05-13 9:00 AM	10.2	10.1	10.2	10.2
2022-05-13 10:00 AM	10.8	10.9	10.8	10.8
2022-05-13 11:00 AM	13.2	13.9	12.0	13.0
2022-05-13 12:00 PM	18.5	22.7	13.3	18.2
2022-05-13 1:00 PM	19.2	23.9	15.0	19.4
2022-05-13 2:00 PM	18.8	22.9	17.9	19.9
2022-05-13 3:00 PM	17.4	20.6	23.0	20.3
2022-05-13 4:00 PM	17.3	18.2	24.2	19.9
2022-05-13 5:00 PM	17.6	17.8	26.8	20.7
2022-05-13 6:00 PM	16.6	16.6	23.3	18.8
2022-05-13 7:00 PM	15.0	14.9	16.4	15.4
2022-05-13 8:00 PM	14.1	13.8	14.7	14.2
2022-05-13 9:00 PM	13.3	12.9	13.7	13.3
2022-05-13 10:00 PM	11.5	11.0	11.9	11.5
2022-05-13 11:00 PM	10.0	9.4	10.4	9.9
2022-05-14 12:00 AM	9.3	8.7	9.8	9.3
2022-05-14 1:00 AM	9.6	9.0	10.0	9.5
2022-05-14 2:00 AM	9.4	8.8	9.7	9.3
2022-05-14 3:00 AM	9.3	8.8	9.6	9.2
2022-05-14 4:00 AM	9.2	8.7	9.4	9.1
2022-05-14 5:00 AM	8.8	8.3	9.1	8.7
2022-05-14 6:00 AM	8.3	7.8	8.6	8.2

2022-05-14 7:00 AM	7.5	6.9	7.9	7.4
2022-05-14 8:00 AM	8.5	8.0	8.6	8.4
2022-05-14 9:00 AM	9.8	9.5	9.2	9.5
2022-05-14 10:00 AM	12.3	12.6	10.6	11.8
2022-05-14 11:00 AM	20.2	26.3	13.0	19.8
2022-05-14 12:00 PM	20.8	26.8	13.2	20.3
2022-05-14 1:00 PM	20.0	26.0	14.4	20.1
2022-05-14 2:00 PM	19.4	24.1	17.9	20.5
2022-05-14 3:00 PM	17.1	20.5	23.2	20.3
2022-05-14 4:00 PM	18.0	18.9	27.2	21.4
2022-05-14 5:00 PM	18.4	18.9	28.0	21.8
2022-05-14 6:00 PM	18.7	19.4	22.3	20.1
2022-05-14 7:00 PM	18.2	18.6	20.0	18.9
2022-05-14 8:00 PM	17.3	17.7	18.8	17.9
2022-05-14 9:00 PM	16.0	16.3	16.9	16.4
2022-05-14 10:00 PM	14.4	14.3	14.8	14.5
2022-05-14 11:00 PM	14.9	14.8	14.8	14.8
2022-05-15 12:00 AM	13.7	13.6	13.7	13.7
2022-05-15 1:00 AM	12.3	12.0	12.5	12.3
2022-05-15 2:00 AM	11.3	10.8	11.5	11.2
2022-05-15 3:00 AM	10.3	9.5	10.5	10.1
2022-05-15 4:00 AM	9.2	8.4	9.4	9.0
2022-05-15 5:00 AM	8.8	8.2	9.2	8.7
2022-05-15 6:00 AM	8.5	7.8	8.7	8.3
2022-05-15 7:00 AM	7.8	7.0	8.2	7.7
2022-05-15 8:00 AM	7.8	7.2	8.3	7.8
2022-05-15 9:00 AM	9.8	9.4	9.7	9.6
2022-05-15 10:00 AM	13.2	13.3	12.1	12.9
2022-05-15 11:00 AM	16.0	17.7	13.6	15.8
2022-05-15 12:00 PM	16.6	18.2	14.5	16.4
2022-05-15 1:00 PM	18.0	20.2	16.1	18.1
2022-05-15 2:00 PM	19.1	21.8	18.7	19.9
2022-05-15 3:00 PM	17.5	18.7	18.4	18.2
2022-05-15 4:00 PM	17.0	17.6	19.2	17.9
2022-05-15 5:00 PM	17.4	18.1	19.9	18.5
2022-05-15 6:00 PM	17.0	17.7	17.4	17.4
2022-05-15 7:00 PM	16.0	16.6	16.2	16.3
2022-05-15 8:00 PM	15.2	15.6	15.2	15.3
2022-05-15 9:00 PM	14.6	14.7	14.4	14.6
2022-05-15 10:00 PM	13.9	14.0	13.9	13.9
2022-05-15 11:00 PM	12.5	12.3	12.5	12.4
2022-05-16 12:00 AM	11.7	11.5	11.9	11.7
2022-05-16 1:00 AM	11.8	11.7	11.5	11.7
2022-05-16 2:00 AM	10.8	10.6	10.6	10.7
2022-05-16 3:00 AM	9.6	9.3	9.5	9.5

2022-05-16 4:00 AM	8.8	8.4	8.9	8.7
2022-05-16 5:00 AM	8.5	7.8	8.6	8.3
2022-05-16 6:00 AM	8.2	7.3	8.2	7.9
2022-05-16 7:00 AM	8.2	7.4	8.2	7.9
2022-05-16 8:00 AM	8.5	8.0	8.5	8.3
2022-05-16 9:00 AM	9.3	9.2	8.8	9.1
2022-05-16 10:00 AM	10.1	10.3	9.2	9.9
2022-05-16 11:00 AM	10.9	11.8	9.1	10.6
2022-05-16 12:00 PM	12.5	13.8	9.7	12.0
2022-05-16 1:00 PM	13.2	14.8	11.4	13.1
2022-05-16 2:00 PM	14.5	16.6	13.8	15.0
2022-05-16 3:00 PM	14.3	15.2	14.8	14.8
2022-05-16 4:00 PM	14.6	15.0	18.5	16.0
2022-05-16 5:00 PM	14.1	14.4	16.6	15.0
2022-05-16 6:00 PM	13.7	13.9	14.2	13.9
2022-05-16 7:00 PM	12.6	12.6	12.7	12.6
2022-05-16 8:00 PM	11.8	11.7	11.7	11.7
2022-05-16 9:00 PM	11.0	10.8	10.8	10.9
2022-05-16 10:00 PM	9.5	9.2	9.4	9.4
2022-05-16 11:00 PM	9.4	9.1	9.3	9.3
2022-05-17 12:00 AM	8.5	8.0	8.4	8.3
2022-05-17 1:00 AM	7.4	6.7	7.6	7.2
2022-05-17 2:00 AM	7.0	6.4	7.3	6.9
2022-05-17 3:00 AM	5.9	5.2	6.4	5.8
2022-05-17 4:00 AM	5.9	5.2	6.2	5.8
2022-05-17 5:00 AM	5.2	4.3	5.6	5.0
2022-05-17 6:00 AM	4.1	3.2	4.8	4.0
2022-05-17 7:00 AM	3.4	2.4	4.1	3.3
2022-05-17 8:00 AM	3.8	3.1	4.4	3.8
2022-05-17 9:00 AM	5.7	5.2	5.6	5.5
2022-05-17 10:00 AM	8.2	7.8	6.7	7.6
2022-05-17 11:00 AM	14.8	19.7	8.5	14.3
2022-05-17 12:00 PM	19.2	25.0	10.7	18.3
2022-05-17 1:00 PM	18.0	23.8	12.8	18.2
2022-05-17 2:00 PM	17.3	22.0	17.4	18.9
2022-05-17 3:00 PM	15.2	19.4	23.4	19.3
2022-05-17 4:00 PM	15.2	16.5	25.9	19.2
2022-05-17 5:00 PM	14.8	15.2	26.9	19.0
2022-05-17 6:00 PM	13.8	13.9	20.3	16.0
2022-05-17 7:00 PM	12.2	12.1	13.4	12.6
2022-05-17 8:00 PM	11.6	11.4	12.1	11.7
2022-05-17 9:00 PM	10.4	10.0	10.8	10.4
2022-05-17 10:00 PM	8.5	7.8	8.9	8.4
2022-05-17 11:00 PM	7.4	6.6	7.9	7.3
2022-05-18 12:00 AM	6.4	5.6	7.0	6.3

2022-05-18 1:00 AM	5.7	4.9	6.4	5.7
2022-05-18 2:00 AM	5.6	4.8	6.4	5.6
2022-05-18 3:00 AM	6.0	5.3	6.7	6.0
2022-05-18 4:00 AM	6.6	6.1	7.0	6.6
2022-05-18 5:00 AM	6.4	5.8	6.9	6.4
2022-05-18 6:00 AM	6.3	5.6	6.7	6.2
2022-05-18 7:00 AM	6.6	6.1	6.9	6.5
2022-05-18 8:00 AM	7.0	6.6	7.1	6.9
2022-05-18 9:00 AM	7.1	6.9	7.4	7.1
2022-05-18 10:00 AM	8.2	8.6	8.2	8.3
2022-05-18 11:00 AM	9.4	9.7	8.8	9.3
2022-05-18 12:00 PM	9.5	9.9	9.3	9.6
2022-05-18 1:00 PM	8.4	8.4	8.3	8.4
2022-05-18 2:00 PM	10.3	10.7	9.4	10.1
2022-05-18 3:00 PM	9.4	9.5	9.0	9.3
2022-05-18 4:00 PM	7.7	7.7	8.0	7.8
2022-05-18 5:00 PM	7.5	7.4	7.8	7.6
2022-05-18 6:00 PM	6.6	6.4	6.9	6.6
2022-05-18 7:00 PM	6.7	6.7	7.4	6.9
2022-05-18 8:00 PM	6.5	6.0	6.7	6.4
2022-05-18 9:00 PM	5.9	5.5	6.4	5.9
2022-05-18 10:00 PM	5.9	5.5	6.3	5.9
2022-05-18 11:00 PM	5.7	5.4	6.1	5.7
2022-05-19 12:00 AM	5.5	5.2	5.9	5.5
2022-05-19 1:00 AM	5.8	5.3	5.9	5.7
2022-05-19 2:00 AM	6.0	5.5	5.9	5.8
2022-05-19 3:00 AM	6.0	5.5	5.8	5.8
2022-05-19 4:00 AM	5.9	5.5	5.9	5.8
2022-05-19 5:00 AM	6.1	5.6	5.8	5.8
2022-05-19 6:00 AM	6.1	5.7	5.8	5.9
2022-05-19 7:00 AM	6.3	5.9	6.2	6.1
2022-05-19 8:00 AM	6.7	6.5	6.6	6.6
2022-05-19 9:00 AM	7.3	7.3	7.1	7.2
2022-05-19 10:00 AM	8.3	8.6	8.1	8.3
2022-05-19 11:00 AM	10.6	11.5	9.9	10.7
2022-05-19 12:00 PM	12.1	13.1	11.5	12.2
2022-05-19 1:00 PM	13.6	15.0	13.0	13.9
2022-05-19 2:00 PM	13.1	13.7	15.1	14.0
2022-05-19 3:00 PM	14.0	14.9	17.7	15.5
2022-05-19 4:00 PM	13.1	13.5	16.3	14.3
2022-05-19 5:00 PM	12.8	13.1	16.4	14.1
2022-05-19 6:00 PM	11.9	12.1	14.5	12.8
2022-05-19 7:00 PM	11.9	11.9	13.6	12.5
2022-05-19 8:00 PM	10.3	10.0	10.5	10.3
2022-05-19 9:00 PM	8.9	8.5	8.8	8.7

2022-05-19 10:00 PM	7.9	7.4	7.5	7.6
2022-05-19 11:00 PM	7.8	7.3	7.7	7.6
2022-05-20 12:00 AM	7.4	7.0	7.3	7.2
2022-05-20 1:00 AM	6.5	6.4	6.7	6.5
2022-05-20 2:00 AM	6.2	5.9	6.4	6.2
2022-05-20 3:00 AM	6.3	5.9	6.3	6.2
2022-05-20 4:00 AM	6.5	6.1	6.5	6.4
2022-05-20 5:00 AM	6.8	6.6	6.8	6.7
2022-05-20 6:00 AM	7.1	6.9	7.0	7.0
2022-05-20 7:00 AM	6.8	6.6	6.8	6.7
2022-05-20 8:00 AM	6.4	6.1	6.4	6.3
2022-05-20 9:00 AM	6.9	6.5	6.9	6.8
2022-05-20 10:00 AM	7.6	7.4	7.7	7.6
2022-05-20 11:00 AM	8.1	8.0	8.3	8.1
2022-05-20 12:00 PM	9.6	10.2	11.1	10.3
2022-05-20 1:00 PM	15.3	19.7	22.8	19.3
2022-05-20 2:00 PM	15.2	18.6	21.8	18.5
2022-05-20 3:00 PM	14.9	17.7	24.5	19.0
2022-05-20 4:00 PM	15.5	17.1	26.2	19.6
2022-05-20 5:00 PM	15.5	16.5	21.4	17.8
2022-05-20 6:00 PM	15.4	15.6	19.8	16.9
2022-05-20 7:00 PM	14.6	14.7	16.1	15.1
2022-05-20 8:00 PM	13.8	14.1	15.0	14.3
2022-05-20 9:00 PM	13.5	13.3	13.6	13.5
2022-05-20 10:00 PM	10.9	10.9	10.9	10.9
2022-05-20 11:00 PM	8.2	8.1	8.1	8.1
2022-05-21 12:00 AM	7.1	6.7	6.5	6.8
2022-05-21 1:00 AM	6.3	5.7	5.8	5.9
2022-05-21 2:00 AM	5.5	5.2	5.2	5.3
2022-05-21 3:00 AM	5.2	4.7	4.8	4.9
2022-05-21 4:00 AM	4.8	4.2	4.4	4.5
2022-05-21 5:00 AM	4.6	3.7	3.7	4.0
2022-05-21 6:00 AM	4.1	3.1	3.0	3.4
2022-05-21 7:00 AM	3.5	2.6	2.4	2.8
2022-05-21 8:00 AM	4.3	3.3	3.4	3.7
2022-05-21 9:00 AM	6.0	5.6	5.7	5.8
2022-05-21 10:00 AM	7.3	7.4	7.5	7.4
2022-05-21 11:00 AM	7.6	7.9	8.8	8.1
2022-05-21 12:00 PM	9.0	9.5	11.6	10.0
2022-05-21 1:00 PM	11.1	12.7	16.0	13.3
2022-05-21 2:00 PM	10.1	10.5	12.5	11.0
2022-05-21 3:00 PM	10.1	10.5	11.9	10.8
2022-05-21 4:00 PM	10.6	10.9	13.2	11.6
2022-05-21 5:00 PM	11.4	11.9	15.6	13.0
2022-05-21 6:00 PM	10.9	11.0	13.5	11.8

2022-05-21 7:00 PM	9.5	9.6	10.7	9.9
2022-05-21 8:00 PM	8.8	8.7	9.2	8.9
2022-05-21 9:00 PM	8.3	8.0	8.4	8.2
2022-05-21 10:00 PM	7.6	7.2	7.4	7.4
2022-05-21 11:00 PM	7.2	6.9	7.0	7.0
2022-05-22 12:00 AM	6.9	6.5	6.7	6.7
2022-05-22 1:00 AM	6.7	6.3	6.3	6.4
2022-05-22 2:00 AM	6.2	5.7	5.8	5.9
2022-05-22 3:00 AM	5.5	5.1	5.1	5.2
2022-05-22 4:00 AM	5.2	4.5	4.7	4.8
2022-05-22 5:00 AM	5.0	4.4	4.6	4.7
2022-05-22 6:00 AM	5.1	4.5	4.8	4.8
2022-05-22 7:00 AM	5.3	4.8	4.9	5.0
2022-05-22 8:00 AM	5.6	5.2	5.3	5.4
2022-05-22 9:00 AM	6.5	6.3	6.6	6.5
2022-05-22 10:00 AM	8.3	8.7	8.9	8.6
2022-05-22 11:00 AM	11.5	14.2	11.2	12.3
2022-05-22 12:00 PM	12.3	14.8	15.4	14.2
2022-05-22 1:00 PM	10.5	11.4	9.2	10.4
2022-05-22 2:00 PM	11.8	12.7	10.8	11.8
2022-05-22 3:00 PM	12.3	13.6	14.6	13.5
2022-05-22 4:00 PM	11.2	11.5	11.2	11.3
2022-05-22 5:00 PM	11.5	11.8	12.1	11.8
2022-05-22 6:00 PM	11.4	11.5	14.4	12.4
2022-05-22 7:00 PM	10.5	10.6	10.9	10.7
2022-05-22 8:00 PM	9.3	9.1	9.7	9.4
2022-05-22 9:00 PM	8.8	8.5	9.0	8.8
2022-05-22 10:00 PM	7.4	6.9	7.6	7.3
2022-05-22 11:00 PM	6.4	5.8	6.6	6.3
2022-05-23 12:00 AM	5.6	5.0	5.8	5.5
2022-05-23 1:00 AM	4.8	4.1	5.1	4.7
2022-05-23 2:00 AM	3.9	3.0	4.3	3.7
2022-05-23 3:00 AM	3.5	2.7	4.0	3.4
2022-05-23 4:00 AM	2.8	2.2	3.6	2.9
2022-05-23 5:00 AM	2.5	1.5	2.9	2.3
2022-05-23 6:00 AM	2.6	1.5	2.9	2.3
2022-05-23 7:00 AM	2.5	1.4	2.7	2.2
2022-05-23 8:00 AM	3.2	2.7	3.5	3.1
2022-05-23 9:00 AM	4.9	4.6	4.9	4.8
2022-05-23 10:00 AM	8.0	9.1	6.6	7.9
2022-05-23 11:00 AM	16.1	23.0	9.5	16.2
2022-05-23 12:00 PM	19.0	25.8	11.6	18.8
2022-05-23 1:00 PM	16.0	18.9	13.2	16.0
2022-05-23 2:00 PM	15.6	18.4	14.6	16.2
2022-05-23 3:00 PM	15.8	20.2	22.4	19.5

2022-05-23 4:00 PM	15.3	16.7	23.7	18.6
2022-05-23 5:00 PM	15.6	16.4	26.8	19.6
2022-05-23 6:00 PM	14.1	14.6	17.0	15.2
2022-05-23 7:00 PM	13.1	13.3	14.4	13.6
2022-05-23 8:00 PM	12.1	12.1	12.9	12.4
2022-05-23 9:00 PM	11.0	10.9	11.8	11.2
2022-05-23 10:00 PM	10.0	9.6	10.5	10.0
2022-05-23 11:00 PM	8.9	8.3	9.2	8.8
2022-05-24 12:00 AM	8.0	7.5	8.3	7.9
2022-05-24 1:00 AM	7.0	6.4	7.4	6.9
2022-05-24 2:00 AM	6.4	5.6	6.8	6.3
2022-05-24 3:00 AM	5.7	4.7	6.1	5.5
2022-05-24 4:00 AM	5.1	3.9	5.5	4.8
2022-05-24 5:00 AM	4.3	3.6	5.0	4.3
2022-05-24 6:00 AM	3.8	3.1	4.4	3.8
2022-05-24 7:00 AM	4.4	3.8	4.9	4.4
2022-05-24 8:00 AM	5.6	4.9	5.8	5.4
2022-05-24 9:00 AM	8.0	8.1	7.4	7.8
2022-05-24 10:00 AM	10.7	12.1	8.9	10.6
2022-05-24 11:00 AM	16.4	22.3	10.3	16.3
2022-05-24 12:00 PM	19.0	25.8	12.1	19.0
2022-05-24 1:00 PM	19.4	25.3	14.1	19.6
2022-05-24 2:00 PM	18.5	22.6	16.3	19.1
2022-05-24 3:00 PM	17.3	20.1	20.4	19.3
2022-05-24 4:00 PM	17.5	19.0	22.2	19.6
2022-05-24 5:00 PM	16.9	17.7	21.2	18.6
2022-05-24 6:00 PM	16.4	17.2	19.2	17.6
2022-05-24 7:00 PM	15.9	16.6	17.5	16.7
2022-05-24 8:00 PM	15.0	15.4	16.2	15.5
2022-05-24 9:00 PM	13.9	14.1	14.6	14.2
2022-05-24 10:00 PM	12.7	12.5	12.8	12.7
2022-05-24 11:00 PM	11.4	10.9	11.5	11.3
2022-05-25 12:00 AM	10.5	9.8	10.7	10.3
2022-05-25 1:00 AM	9.8	9.2	10.1	9.7
2022-05-25 2:00 AM	9.4	8.7	9.6	9.2
2022-05-25 3:00 AM	8.8	8.3	9.3	8.8
2022-05-25 4:00 AM	8.4	7.7	8.8	8.3
2022-05-25 5:00 AM	8.0	7.2	8.4	7.9
2022-05-25 6:00 AM	7.8	7.1	8.2	7.7
2022-05-25 7:00 AM	7.8	7.3	8.2	7.8
2022-05-25 8:00 AM	8.4	8.0	8.7	8.4
2022-05-25 9:00 AM	9.0	8.7	9.1	8.9
2022-05-25 10:00 AM	9.7	9.6	9.6	9.6
2022-05-25 11:00 AM	9.8	9.8	9.7	9.8
2022-05-25 12:00 PM	10.0	10.0	10.0	10.0

2022-05-25 1:00 PM	10.8	10.9	10.7	10.8
2022-05-25 2:00 PM	11.6	12.1	11.7	11.8
2022-05-25 3:00 PM	11.9	12.3	12.0	12.1
2022-05-25 4:00 PM	10.9	10.9	10.9	10.9
2022-05-25 5:00 PM	10.0	9.9	10.1	10.0
2022-05-25 6:00 PM	9.6	9.3	9.6	9.5
2022-05-25 7:00 PM	9.5	9.3	9.6	9.5
2022-05-25 8:00 PM	9.4	9.2	9.4	9.3
2022-05-25 9:00 PM	9.2	8.9	9.2	9.1
2022-05-25 10:00 PM	9.0	8.8	9.0	8.9
2022-05-25 11:00 PM	8.8	8.7	8.9	8.8
2022-05-26 12:00 AM	8.9	8.7	8.9	8.8
2022-05-26 1:00 AM	9.1	9.0	9.2	9.1
2022-05-26 2:00 AM	9.1	9.0	9.2	9.1
2022-05-26 3:00 AM	9.1	9.0	9.2	9.1
2022-05-26 4:00 AM	9.1	9.0	9.2	9.1
2022-05-26 5:00 AM	9.0	9.0	9.1	9.0
2022-05-26 6:00 AM	9.0	8.9	9.1	9.0
2022-05-26 7:00 AM	9.0	8.9	9.1	9.0
2022-05-26 8:00 AM	9.3	9.1	9.2	9.2
2022-05-26 9:00 AM	9.7	9.6	9.6	9.6
2022-05-26 10:00 AM	10.7	10.6	10.5	10.6
2022-05-26 11:00 AM	12.0	12.2	11.6	11.9
2022-05-26 12:00 PM	12.1	12.7	11.8	12.2
2022-05-26 1:00 PM	12.3	12.4	11.8	12.2
2022-05-26 2:00 PM	12.8	12.9	12.6	12.8
2022-05-26 3:00 PM	13.5	13.8	13.2	13.5
2022-05-26 4:00 PM	14.3	14.6	15.0	14.6
2022-05-26 5:00 PM	14.2	14.4	13.9	14.2
2022-05-26 6:00 PM	13.9	13.9	13.5	13.8
2022-05-26 7:00 PM	13.9	14.0	13.4	13.8
2022-05-26 8:00 PM	13.3	13.6	13.0	13.3
2022-05-26 9:00 PM	13.1	13.1	12.6	12.9
2022-05-26 10:00 PM	12.1	12.2	12.0	12.1
2022-05-26 11:00 PM	11.6	11.5	11.5	11.5
2022-05-27 12:00 AM	11.4	11.2	11.3	11.3
2022-05-27 1:00 AM	11.1	10.9	11.1	11.0
2022-05-27 2:00 AM	10.9	10.7	10.9	10.8
2022-05-27 3:00 AM	10.7	10.5	10.8	10.7
2022-05-27 4:00 AM	10.5	10.1	10.5	10.4
2022-05-27 5:00 AM	10.1	9.7	10.3	10.0
2022-05-27 6:00 AM	10.1	9.7	10.3	10.0
2022-05-27 7:00 AM	10.3	10.0	10.4	10.2
2022-05-27 8:00 AM	10.6	10.5	10.3	10.5
2022-05-27 9:00 AM	10.4	10.1	10.2	10.2

2022-05-27 10:00 AM	13.5	14.4	11.4	13.1
2022-05-27 11:00 AM	19.8	25.5	14.2	19.8
2022-05-27 12:00 PM	19.4	24.7	15.6	19.9
2022-05-27 1:00 PM	18.7	21.7	16.4	18.9
2022-05-27 2:00 PM	19.7	24.0	18.2	20.6
2022-05-27 3:00 PM	18.9	24.0	26.9	23.3
2022-05-27 4:00 PM	18.3	20.6	31.3	23.4
2022-05-27 5:00 PM	18.4	19.8	28.6	22.3
2022-05-27 6:00 PM	17.3	17.9	20.4	18.5
2022-05-27 7:00 PM	16.0	16.2	17.1	16.4
2022-05-27 8:00 PM	15.1	15.1	15.5	15.2
2022-05-27 9:00 PM	14.6	14.4	14.7	14.6
2022-05-27 10:00 PM	13.1	12.5	13.3	13.0
2022-05-27 11:00 PM	12.3	11.6	12.5	12.1
2022-05-28 12:00 AM	11.9	11.1	12.2	11.7
2022-05-28 1:00 AM	11.0	10.3	11.4	10.9
2022-05-28 2:00 AM	10.6	9.6	10.9	10.4
2022-05-28 3:00 AM	9.8	9.1	10.5	9.8
2022-05-28 4:00 AM	9.6	8.8	10.3	9.6
2022-05-28 5:00 AM	9.5	8.8	10.0	9.4
2022-05-28 6:00 AM	9.5	8.9	10.1	9.5
2022-05-28 7:00 AM	9.7	9.2	10.3	9.7
2022-05-28 8:00 AM	10.4	9.9	10.6	10.3
2022-05-28 9:00 AM	10.3	9.8	10.4	10.2
2022-05-28 10:00 AM	10.3	9.9	10.5	10.2
2022-05-28 11:00 AM	10.6	10.4	10.7	10.6
2022-05-28 12:00 PM	11.2	11.2	11.3	11.2
2022-05-28 1:00 PM	11.6	11.5	11.5	11.5
2022-05-28 2:00 PM	12.0	12.0	12.0	12.0
2022-05-28 3:00 PM	11.7	11.5	11.7	11.6
2022-05-28 4:00 PM	11.1	10.7	11.1	11.0
2022-05-28 5:00 PM	10.7	10.3	10.8	10.6
2022-05-28 6:00 PM	10.9	10.5	10.9	10.8
2022-05-28 7:00 PM	11.1	10.9	11.1	11.0
2022-05-28 8:00 PM	10.8	10.5	10.8	10.7
2022-05-28 9:00 PM	10.5	10.3	10.6	10.5
2022-05-28 10:00 PM	10.4	10.0	10.4	10.3
2022-05-28 11:00 PM	10.2	9.9	10.3	10.1
2022-05-29 12:00 AM	10.3	10.0	10.3	10.2
2022-05-29 1:00 AM	10.4	10.2	10.4	10.3
2022-05-29 2:00 AM	10.1	9.9	10.2	10.1
2022-05-29 3:00 AM	9.7	9.5	9.8	9.7
2022-05-29 4:00 AM	9.2	8.9	9.3	9.1
2022-05-29 5:00 AM	8.9	8.5	9.0	8.8
2022-05-29 6:00 AM	8.8	8.4	9.0	8.7

2022-05-29 7:00 AM	8.9	8.5	9.0	8.8
2022-05-29 8:00 AM	9.0	8.6	9.2	8.9
2022-05-29 9:00 AM	9.5	9.2	9.6	9.4
2022-05-29 10:00 AM	11.8	11.9	10.5	11.4
2022-05-29 11:00 AM	15.0	17.0	10.9	14.3
2022-05-29 12:00 PM	16.0	19.3	12.0	15.8
2022-05-29 1:00 PM	15.1	16.8	13.1	15.0
2022-05-29 2:00 PM	14.2	14.9	13.6	14.2
2022-05-29 3:00 PM	14.1	14.8	14.6	14.5
2022-05-29 4:00 PM	13.6	13.7	13.3	13.5
2022-05-29 5:00 PM	12.2	11.9	11.9	12.0
2022-05-29 6:00 PM	12.8	12.6	12.9	12.8
2022-05-29 7:00 PM	13.1	13.0	13.4	13.2
2022-05-29 8:00 PM	12.8	12.7	12.6	12.7
2022-05-29 9:00 PM	12.2	11.9	12.0	12.0
2022-05-29 10:00 PM	11.7	11.4	11.5	11.5
2022-05-29 11:00 PM	11.7	11.5	11.6	11.6
2022-05-30 12:00 AM	11.6	11.3	11.4	11.4
2022-05-30 1:00 AM	11.3	11.0	11.2	11.2
2022-05-30 2:00 AM	11.2	10.8	11.1	11.0
2022-05-30 3:00 AM	11.1	10.7	11.0	10.9
2022-05-30 4:00 AM	10.6	10.2	10.6	10.5
2022-05-30 5:00 AM	10.5	10.1	10.5	10.4
2022-05-30 6:00 AM	10.5	10.0	10.4	10.3
2022-05-30 7:00 AM	10.5	10.1	10.4	10.3
2022-05-30 8:00 AM	11.0	10.8	10.8	10.9
2022-05-30 9:00 AM	10.8	10.6	10.7	10.7
2022-05-30 10:00 AM	10.5	10.2	10.4	10.4
2022-05-30 11:00 AM	12.2	12.4	12.1	12.2
2022-05-30 12:00 PM	12.1	12.2	12.0	12.1
2022-05-30 1:00 PM	13.4	14.2	13.0	13.5
2022-05-30 2:00 PM	11.9	11.9	11.8	11.9
2022-05-30 3:00 PM	12.5	12.8	12.9	12.7
2022-05-30 4:00 PM	14.4	15.1	15.1	14.9
2022-05-30 5:00 PM	15.0	15.8	19.9	16.9
2022-05-30 6:00 PM	15.0	15.6	18.0	16.2
2022-05-30 7:00 PM	14.7	15.2	15.5	15.1
2022-05-30 8:00 PM	14.4	14.7	14.7	14.6
2022-05-30 9:00 PM	14.1	14.4	14.2	14.2
2022-05-30 10:00 PM	13.2	13.3	13.3	13.3
2022-05-30 11:00 PM	12.5	12.3	12.5	12.4
2022-05-31 12:00 AM	12.3	12.1	12.3	12.2
2022-05-31 1:00 AM	12.1	11.9	12.2	12.1
2022-05-31 2:00 AM	11.7	11.4	11.8	11.6
2022-05-31 3:00 AM	11.2	10.8	11.3	11.1

2022-05-31 4:00 AM	11.0	10.5	11.1	10.9
2022-05-31 5:00 AM	10.7	10.2	10.8	10.6
2022-05-31 6:00 AM	10.6	10.1	10.7	10.5
2022-05-31 7:00 AM	10.5	10.0	10.6	10.4
2022-05-31 8:00 AM	10.9	10.6	10.9	10.8
2022-05-31 9:00 AM	11.7	11.7	11.5	11.6
2022-05-31 10:00 AM	13.7	14.4	12.7	13.6
2022-05-31 11:00 AM	16.3	18.8	13.3	16.1
2022-05-31 12:00 PM	19.0	23.8	16.7	19.8
2022-05-31 1:00 PM	19.2	23.3	18.4	20.3
2022-05-31 2:00 PM	18.3	21.2	19.2	19.6
2022-05-31 3:00 PM	18.7	21.4	20.0	20.0
2022-05-31 4:00 PM	18.8	21.1	21.4	20.4
2022-05-31 5:00 PM	17.9	19.6	19.8	19.1
2022-05-31 6:00 PM	17.8	19.3	21.1	19.4
2022-05-31 7:00 PM	16.7	17.7	17.4	17.3
2022-05-31 8:00 PM	16.0	16.6	16.5	16.4
2022-05-31 9:00 PM	15.2	15.5	15.5	15.4
2022-05-31 10:00 PM	14.2	13.9	14.2	14.1
2022-05-31 11:00 PM	13.1	12.5	13.2	12.9
2022-06-01 12:00 AM	12.9	12.2	12.9	12.7
2022-06-01 1:00 AM	12.1	11.2	12.0	11.8
2022-06-01 2:00 AM	11.4	10.4	11.4	11.1
2022-06-01 3:00 AM	11.1	10.2	11.2	10.8
2022-06-01 4:00 AM	11.0	10.2	11.1	10.8
2022-06-01 5:00 AM	10.3	9.6	10.6	10.2
2022-06-01 6:00 AM	9.9	9.1	10.1	9.7
2022-06-01 7:00 AM	9.4	8.6	9.6	9.2
2022-06-01 8:00 AM	9.9	9.3	10.1	9.8
2022-06-01 9:00 AM	10.0	9.6	9.9	9.8
2022-06-01 10:00 AM	11.6	11.6	10.7	11.3
2022-06-01 11:00 AM	15.9	18.4	11.6	15.3
2022-06-01 12:00 PM	16.5	20.7	12.7	16.6
2022-06-01 1:00 PM	15.5	18.4	12.3	15.4
2022-06-01 2:00 PM	16.1	20.8	14.5	17.1
2022-06-01 3:00 PM	15.7	21.6	22.8	20.0
2022-06-01 4:00 PM	16.0	18.7	26.0	20.2
2022-06-01 5:00 PM	16.2	18.2	28.2	20.9
2022-06-01 6:00 PM	16.1	17.2	22.8	18.7
2022-06-01 7:00 PM	15.5	16.3	17.1	16.3
2022-06-01 8:00 PM	15.3	15.8	16.1	15.7
2022-06-01 9:00 PM	14.1	13.9	14.7	14.2
2022-06-01 10:00 PM	12.8	12.1	13.4	12.8
2022-06-01 11:00 PM	11.8	10.9	12.3	11.7
2022-06-02 12:00 AM	11.1	9.9	11.6	10.9

2022-06-02 1:00 AM	10.5	9.2	11.0	10.2
2022-06-02 2:00 AM	9.9	8.7	10.5	9.7
2022-06-02 3:00 AM	9.6	8.3	10.1	9.3
2022-06-02 4:00 AM	9.3	8.1	9.8	9.1
2022-06-02 5:00 AM	8.7	7.2	9.1	8.3
2022-06-02 6:00 AM	8.3	7.0	9.0	8.1
2022-06-02 7:00 AM	8.1	6.8	8.6	7.8
2022-06-02 8:00 AM	9.0	8.1	9.2	8.8
2022-06-02 9:00 AM	10.1	9.7	10.1	10.0
2022-06-02 10:00 AM	12.6	12.8	11.1	12.2
2022-06-02 11:00 AM	17.5	20.0	12.0	16.5
2022-06-02 12:00 PM	17.4	21.1	12.5	17.0
2022-06-02 1:00 PM	18.6	23.1	13.8	18.5
2022-06-02 2:00 PM	18.2	22.6	15.9	18.9
2022-06-02 3:00 PM	16.8	19.6	18.2	18.2
2022-06-02 4:00 PM	15.8	16.9	15.5	16.1
2022-06-02 5:00 PM	16.5	18.3	20.9	18.6
2022-06-02 6:00 PM	16.5	18.0	19.0	17.8
2022-06-02 7:00 PM	15.7	16.7	16.3	16.2
2022-06-02 8:00 PM	14.9	15.4	15.1	15.1
2022-06-02 9:00 PM	13.9	13.9	13.9	13.9
2022-06-02 10:00 PM	13.1	12.7	13.0	12.9
2022-06-02 11:00 PM	12.6	11.9	12.7	12.4
2022-06-03 12:00 AM	11.9	11.0	12.2	11.7
2022-06-03 1:00 AM	12.0	11.3	12.1	11.8
2022-06-03 2:00 AM	11.8	11.2	11.9	11.6
2022-06-03 3:00 AM	11.3	10.7	11.5	11.2
2022-06-03 4:00 AM	11.0	10.4	11.2	10.9
2022-06-03 5:00 AM	10.8	10.0	11.0	10.6
2022-06-03 6:00 AM	10.3	9.6	10.6	10.2
2022-06-03 7:00 AM	9.8	9.1	10.3	9.7
2022-06-03 8:00 AM	9.8	9.0	10.1	9.6
2022-06-03 9:00 AM	10.0	9.5	10.2	9.9
2022-06-03 10:00 AM	10.7	10.6	10.7	10.7
2022-06-03 11:00 AM	12.3	13.2	11.4	12.3
2022-06-03 12:00 PM	15.2	18.6	12.1	15.3
2022-06-03 1:00 PM	15.5	19.1	13.0	15.9
2022-06-03 2:00 PM	15.6	19.3	14.2	16.4
2022-06-03 3:00 PM	13.8	16.8	16.7	15.8
2022-06-03 4:00 PM	14.0	16.0	21.0	17.0
2022-06-03 5:00 PM	14.4	15.7	17.7	15.9
2022-06-03 6:00 PM	12.8	11.9	13.5	12.7
2022-06-03 7:00 PM	12.1	11.4	12.5	12.0
2022-06-03 8:00 PM	11.4	11.1	11.9	11.5
2022-06-03 9:00 PM	11.5	11.1	11.6	11.4

2022-06-03 10:00 PM	11.3	10.9	11.5	11.2
2022-06-03 11:00 PM	10.7	10.0	11.0	10.6
2022-06-04 12:00 AM	10.4	9.6	10.7	10.2
2022-06-04 1:00 AM	9.9	9.0	10.3	9.7
2022-06-04 2:00 AM	8.6	7.3	9.4	8.4
2022-06-04 3:00 AM	8.2	6.7	8.9	7.9
2022-06-04 4:00 AM	7.7	6.2	8.4	7.4
2022-06-04 5:00 AM	7.1	5.6	8.0	6.9
2022-06-04 6:00 AM	6.5	5.0	7.4	6.3
2022-06-04 7:00 AM	6.6	5.1	7.5	6.4
2022-06-04 8:00 AM	7.6	6.7	8.3	7.5
2022-06-04 9:00 AM	8.6	8.2	8.7	8.5
2022-06-04 10:00 AM	11.0	11.5	9.4	10.6
2022-06-04 11:00 AM	16.3	19.1	10.3	15.2
2022-06-04 12:00 PM	17.7	21.0	11.0	16.6
2022-06-04 1:00 PM	17.6	17.0	12.0	15.5
2022-06-04 2:00 PM	17.0	16.6	13.4	15.7
2022-06-04 3:00 PM	15.8	16.5	19.9	17.4
2022-06-04 4:00 PM	14.8	15.1	22.0	17.3
2022-06-04 5:00 PM	14.7	15.4	25.1	18.4
2022-06-04 6:00 PM	13.8	13.7	18.5	15.3
2022-06-04 7:00 PM	12.8	12.3	13.6	12.9
2022-06-04 8:00 PM	12.2	11.6	12.6	12.1
2022-06-04 9:00 PM	11.6	10.9	12.0	11.5
2022-06-04 10:00 PM	10.8	9.9	11.3	10.7
2022-06-04 11:00 PM	10.3	9.2	10.8	10.1
2022-06-05 12:00 AM	9.7	8.7	10.4	9.6
2022-06-05 1:00 AM	9.2	8.2	10.1	9.2
2022-06-05 2:00 AM	8.7	7.3	9.4	8.5
2022-06-05 3:00 AM	8.1	6.4	9.1	7.9
2022-06-05 4:00 AM	7.5	5.7	8.6	7.3
2022-06-05 5:00 AM	7.2	5.3	8.3	6.9
2022-06-05 6:00 AM	6.8	5.1	7.8	6.6
2022-06-05 7:00 AM	6.6	4.9	7.5	6.3
2022-06-05 8:00 AM	7.1	5.9	8.1	7.0
2022-06-05 9:00 AM	8.3	7.8	8.7	8.3
2022-06-05 10:00 AM	10.9	11.5	9.5	10.6
2022-06-05 11:00 AM	15.9	18.4	10.5	14.9
2022-06-05 12:00 PM	16.6	18.8	11.8	15.7
2022-06-05 1:00 PM	17.1	17.5	12.8	15.8
2022-06-05 2:00 PM	17.0	16.6	14.0	15.9
2022-06-05 3:00 PM	15.9	16.6	21.5	18.0
2022-06-05 4:00 PM	15.1	15.7	22.7	17.8
2022-06-05 5:00 PM	14.9	15.1	19.3	16.4
2022-06-05 6:00 PM	14.7	14.9	19.3	16.3

2022-06-05 7:00 PM	13.3	12.8	14.1	13.4
2022-06-05 8:00 PM	12.7	12.2	13.2	12.7
2022-06-05 9:00 PM	12.1	11.4	12.3	11.9
2022-06-05 10:00 PM	11.4	10.6	11.9	11.3
2022-06-05 11:00 PM	11.0	10.2	11.6	10.9
2022-06-06 12:00 AM	10.8	9.9	11.2	10.6
2022-06-06 1:00 AM	10.5	9.6	11.1	10.4
2022-06-06 2:00 AM	10.6	9.9	11.1	10.5
2022-06-06 3:00 AM	10.7	10.1	11.0	10.6
2022-06-06 4:00 AM	10.7	10.1	11.0	10.6
2022-06-06 5:00 AM	10.9	10.6	11.2	10.9
2022-06-06 6:00 AM	10.8	10.4	11.1	10.8
2022-06-06 7:00 AM	10.9	10.6	11.1	10.9
2022-06-06 8:00 AM	11.2	11.1	11.3	11.2
2022-06-06 9:00 AM	11.3	11.2	11.5	11.3
2022-06-06 10:00 AM	11.3	11.2	11.5	11.3
2022-06-06 11:00 AM	11.8	12.1	11.9	11.9
2022-06-06 12:00 PM	11.8	11.9	11.9	11.9
2022-06-06 1:00 PM	12.6	13.1	12.6	12.8
2022-06-06 2:00 PM	14.4	15.0	14.2	14.5
2022-06-06 3:00 PM	15.3	16.4	21.0	17.6
2022-06-06 4:00 PM	16.5	17.3	26.5	20.1
2022-06-06 5:00 PM	16.5	17.6	26.8	20.3
2022-06-06 6:00 PM	15.8	16.5	21.0	17.8
2022-06-06 7:00 PM	14.8	15.0	16.1	15.3
2022-06-06 8:00 PM	14.3	14.3	15.2	14.6
2022-06-06 9:00 PM	14.2	14.2	14.8	14.4
2022-06-06 10:00 PM	13.6	13.4	14.0	13.7
2022-06-06 11:00 PM	12.4	11.9	12.9	12.4
2022-06-07 12:00 AM	11.8	11.2	12.5	11.8
2022-06-07 1:00 AM	11.4	10.6	12.1	11.4
2022-06-07 2:00 AM	11.1	10.4	11.8	11.1
2022-06-07 3:00 AM	10.6	9.8	11.4	10.6
2022-06-07 4:00 AM	9.8	9.0	10.6	9.8
2022-06-07 5:00 AM	9.2	8.4	10.0	9.2
2022-06-07 6:00 AM	8.9	8.0	9.6	8.8
2022-06-07 7:00 AM	8.8	8.1	9.6	8.8
2022-06-07 8:00 AM	9.3	8.8	10.0	9.4
2022-06-07 9:00 AM	10.5	10.4	10.8	10.6
2022-06-07 10:00 AM	13.0	13.4	11.7	12.7
2022-06-07 11:00 AM	17.6	18.8	12.8	16.4
2022-06-07 12:00 PM	16.2	16.5	13.5	15.4
2022-06-07 1:00 PM	17.7	16.9	14.6	16.4
2022-06-07 2:00 PM	16.7	16.5	14.6	15.9
2022-06-07 3:00 PM	16.1	16.1	19.4	17.2

2022-06-07 4:00 PM	17.1	17.4	21.2	18.6
2022-06-07 5:00 PM	16.3	16.4	19.9	17.5
2022-06-07 6:00 PM	16.3	16.5	18.8	17.2
2022-06-07 7:00 PM	15.8	15.7	15.8	15.8
2022-06-07 8:00 PM	15.1	14.9	15.2	15.1
2022-06-07 9:00 PM	14.8	14.6	14.7	14.7
2022-06-07 10:00 PM	13.5	13.0	13.6	13.4
2022-06-07 11:00 PM	13.0	12.5	13.1	12.9
2022-06-08 12:00 AM	12.2	11.5	12.6	12.1
2022-06-08 1:00 AM	12.0	11.3	12.3	11.9
2022-06-08 2:00 AM	11.9	11.4	12.2	11.8
2022-06-08 3:00 AM	11.5	10.9	11.8	11.4
2022-06-08 4:00 AM	11.0	10.1	11.3	10.8
2022-06-08 5:00 AM	10.8	9.9	11.3	10.7
2022-06-08 6:00 AM	11.0	10.5	11.5	11.0
2022-06-08 7:00 AM	11.2	10.8	11.6	11.2
2022-06-08 8:00 AM	11.7	11.6	11.8	11.7
2022-06-08 9:00 AM	12.7	13.1	12.3	12.7
2022-06-08 10:00 AM	14.6	15.1	12.8	14.2
2022-06-08 11:00 AM	19.9	21.4	13.8	18.4
2022-06-08 12:00 PM	21.1	22.4	14.8	19.4
2022-06-08 1:00 PM	19.9	19.0	15.5	18.1
2022-06-08 2:00 PM	18.2	17.3	15.5	17.0
2022-06-08 3:00 PM	15.9	15.3	14.3	15.2
2022-06-08 4:00 PM	15.7	15.4	14.5	15.2
2022-06-08 5:00 PM	16.2	16.0	15.4	15.9
2022-06-08 6:00 PM	16.9	16.9	16.1	16.6
2022-06-08 7:00 PM	15.9	15.6	15.3	15.6
2022-06-08 8:00 PM	15.9	15.7	15.1	15.6
2022-06-08 9:00 PM	15.2	14.9	14.6	14.9
2022-06-08 10:00 PM	14.4	13.9	13.9	14.1
2022-06-08 11:00 PM	13.8	13.2	13.3	13.4
2022-06-09 12:00 AM	13.4	12.9	13.2	13.2
2022-06-09 1:00 AM	13.2	12.8	13.1	13.0
2022-06-09 2:00 AM	12.9	12.3	12.8	12.7
2022-06-09 3:00 AM	12.3	11.7	12.3	12.1
2022-06-09 4:00 AM	11.8	11.0	11.9	11.6
2022-06-09 5:00 AM	11.7	11.4	11.8	11.6
2022-06-09 6:00 AM	11.6	11.4	11.8	11.6
2022-06-09 7:00 AM	11.7	11.4	11.9	11.7
2022-06-09 8:00 AM	12.4	12.4	12.3	12.4
2022-06-09 9:00 AM	13.3	13.5	12.7	13.2
2022-06-09 10:00 AM	13.8	14.1	12.8	13.6
2022-06-09 11:00 AM	16.5	17.4	13.3	15.7
2022-06-09 12:00 PM	16.8	18.1	13.8	16.2

2022-06-09 1:00 PM	18.2	18.3	14.9	17.1
2022-06-09 2:00 PM	16.5	16.2	15.1	15.9
2022-06-09 3:00 PM	17.1	17.0	17.6	17.2
2022-06-09 4:00 PM	16.9	17.1	19.7	17.9
2022-06-09 5:00 PM	15.8	16.1	17.5	16.5
2022-06-09 6:00 PM	16.0	15.9	16.5	16.1
2022-06-09 7:00 PM	16.1	16.0	15.5	15.9
2022-06-09 8:00 PM	15.7	15.5	15.2	15.5
2022-06-09 9:00 PM	15.4	15.0	15.1	15.2
2022-06-09 10:00 PM	15.0	14.7	14.7	14.8
2022-06-09 11:00 PM	13.9	13.3	14.0	13.7
2022-06-10 12:00 AM	13.3	12.8	13.6	13.2
2022-06-10 1:00 AM	13.1	12.6	13.3	13.0
2022-06-10 2:00 AM	12.4	11.8	12.7	12.3
2022-06-10 3:00 AM	12.3	11.9	12.7	12.3
2022-06-10 4:00 AM	12.1	11.6	12.4	12.0
2022-06-10 5:00 AM	11.5	10.7	12.0	11.4
2022-06-10 6:00 AM	11.0	10.2	11.5	10.9
2022-06-10 7:00 AM	10.9	10.2	11.3	10.8
2022-06-10 8:00 AM	10.8	10.2	11.3	10.8
2022-06-10 9:00 AM	11.8	11.7	11.9	11.8
2022-06-10 10:00 AM	14.0	14.7	12.6	13.8
2022-06-10 11:00 AM	15.7	16.4	13.7	15.3
2022-06-10 12:00 PM	18.1	19.8	14.6	17.5
2022-06-10 1:00 PM	19.2	19.8	15.5	18.2
2022-06-10 2:00 PM	19.2	19.3	16.7	18.4
2022-06-10 3:00 PM	18.8	19.1	22.4	20.1
2022-06-10 4:00 PM	18.7	19.5	25.3	21.2
2022-06-10 5:00 PM	18.7	19.7	24.8	21.1
2022-06-10 6:00 PM	18.4	19.0	20.2	19.2
2022-06-10 7:00 PM	17.3	17.4	17.0	17.2
2022-06-10 8:00 PM	17.2	17.4	16.5	17.0
2022-06-10 9:00 PM	16.6	16.5	16.2	16.4
2022-06-10 10:00 PM	15.7	15.4	15.8	15.6
2022-06-10 11:00 PM	14.0	13.2	14.5	13.9
2022-06-11 12:00 AM	13.0	12.3	13.7	13.0
2022-06-11 1:00 AM	12.4	11.6	13.1	12.4
2022-06-11 2:00 AM	12.3	11.6	13.1	12.3
2022-06-11 3:00 AM	12.3	11.7	12.9	12.3
2022-06-11 4:00 AM	11.7	11.0	12.4	11.7
2022-06-11 5:00 AM	11.2	10.4	12.0	11.2
2022-06-11 6:00 AM	10.9	10.1	11.6	10.9
2022-06-11 7:00 AM	11.2	10.6	11.8	11.2
2022-06-11 8:00 AM	11.5	11.1	12.1	11.6
2022-06-11 9:00 AM	12.0	11.8	12.3	12.0

2022-06-11 10:00 AM	13.7	14.1	13.0	13.6
2022-06-11 11:00 AM	16.5	17.0	13.9	15.8
2022-06-11 12:00 PM	16.3	16.5	14.2	15.7
2022-06-11 1:00 PM	19.6	18.3	15.2	17.7
2022-06-11 2:00 PM	19.5	18.4	16.5	18.1
2022-06-11 3:00 PM	18.3	18.5	21.2	19.3
2022-06-11 4:00 PM	17.8	18.1	24.7	20.2
2022-06-11 5:00 PM	17.4	18.0	24.7	20.0
2022-06-11 6:00 PM	16.9	17.1	20.4	18.1
2022-06-11 7:00 PM	15.3	14.9	16.1	15.4
2022-06-11 8:00 PM	14.6	14.1	15.2	14.6
2022-06-11 9:00 PM	14.2	13.6	14.8	14.2
2022-06-11 10:00 PM	13.6	13.0	14.3	13.6
2022-06-11 11:00 PM	12.8	12.0	13.6	12.8
2022-06-12 12:00 AM	12.4	11.6	13.2	12.4
2022-06-12 1:00 AM	11.8	10.9	12.5	11.7
2022-06-12 2:00 AM	11.1	10.1	12.0	11.1
2022-06-12 3:00 AM	10.8	9.7	11.7	10.7
2022-06-12 4:00 AM	10.2	9.1	11.1	10.1
2022-06-12 5:00 AM	9.8	8.8	10.7	9.8
2022-06-12 6:00 AM	9.4	8.3	10.3	9.3
2022-06-12 7:00 AM	9.1	8.2	9.8	9.0
2022-06-12 8:00 AM	9.4	8.7	10.2	9.4
2022-06-12 9:00 AM	10.4	10.0	11.1	10.5
2022-06-12 10:00 AM	13.0	13.2	11.9	12.7
2022-06-12 11:00 AM	18.0	18.8	13.1	16.6
2022-06-12 12:00 PM	19.0	19.6	14.2	17.6
2022-06-12 1:00 PM	19.8	17.3	14.9	17.3
2022-06-12 2:00 PM	17.9	16.9	15.5	16.8
2022-06-12 3:00 PM	17.5	17.5	18.3	17.8
2022-06-12 4:00 PM	17.2	17.7	20.8	18.6
2022-06-12 5:00 PM	16.6	16.5	17.1	16.7
2022-06-12 6:00 PM	16.4	16.4	17.2	16.7
2022-06-12 7:00 PM	16.2	15.9	15.9	16.0
2022-06-12 8:00 PM	15.3	14.9	15.0	15.1
2022-06-12 9:00 PM	14.8	14.4	14.7	14.6
2022-06-12 10:00 PM	13.9	13.4	14.0	13.8
2022-06-12 11:00 PM	13.5	12.9	13.5	13.3
2022-06-13 12:00 AM	13.2	12.6	13.3	13.0
2022-06-13 1:00 AM	13.2	12.7	13.3	13.1
2022-06-13 2:00 AM	13.1	12.8	13.1	13.0
2022-06-13 3:00 AM	13.0	12.6	13.0	12.9
2022-06-13 4:00 AM	12.9	12.5	13.0	12.8
2022-06-13 5:00 AM	12.8	12.4	12.8	12.7
2022-06-13 6:00 AM	12.5	12.0	12.4	12.3

2022-06-13 7:00 AM	12.1	11.7	12.2	12.0
2022-06-13 8:00 AM	12.0	11.6	12.0	11.9
2022-06-13 9:00 AM	12.2	11.7	12.0	12.0
2022-06-13 10:00 AM	12.6	12.1	12.3	12.3
2022-06-13 11:00 AM	12.4	12.0	12.2	12.2
2022-06-13 12:00 PM	12.7	12.4	12.4	12.5
2022-06-13 1:00 PM	13.2	12.8	12.6	12.9
2022-06-13 2:00 PM	13.2	12.7	12.6	12.8
2022-06-13 3:00 PM	12.7	12.3	12.4	12.5
2022-06-13 4:00 PM	12.7	12.3	12.3	12.4
2022-06-13 5:00 PM	12.3	12.0	12.1	12.1
2022-06-13 6:00 PM	12.2	11.9	12.0	12.0
2022-06-13 7:00 PM	12.2	11.9	12.0	12.0
2022-06-13 8:00 PM	12.0	11.8	11.9	11.9
2022-06-13 9:00 PM	12.2	12.0	12.0	12.1
2022-06-13 10:00 PM	12.1	11.9	12.0	12.0
2022-06-13 11:00 PM	11.8	11.7	11.8	11.8
2022-06-14 12:00 AM	11.8	11.7	11.8	11.8
2022-06-14 1:00 AM	11.8	11.7	11.8	11.8
2022-06-14 2:00 AM	11.8	11.7	11.8	11.8
2022-06-14 3:00 AM	11.8	11.6	11.7	11.7
2022-06-14 4:00 AM	11.7	11.6	11.7	11.7
2022-06-14 5:00 AM	11.7	11.5	11.6	11.6
2022-06-14 6:00 AM	11.7	11.5	11.6	11.6
2022-06-14 7:00 AM	11.8	11.5	11.6	11.6
2022-06-14 8:00 AM	11.9	11.6	11.7	11.7
2022-06-14 9:00 AM	11.8	11.6	11.6	11.7
2022-06-14 10:00 AM	11.9	11.7	11.7	11.8
2022-06-14 11:00 AM	12.0	11.8	11.8	11.9
2022-06-14 12:00 PM	11.9	11.6	11.7	11.7
2022-06-14 1:00 PM	12.0	11.8	11.8	11.9
2022-06-14 2:00 PM	12.2	11.9	11.9	12.0
2022-06-14 3:00 PM	12.5	12.3	12.1	12.3
2022-06-14 4:00 PM	13.0	12.8	12.5	12.8
2022-06-14 5:00 PM	13.2	13.1	12.7	13.0
2022-06-14 6:00 PM	13.2	13.1	12.8	13.0
2022-06-14 7:00 PM	13.3	13.2	12.9	13.1
2022-06-14 8:00 PM	13.2	13.0	12.8	13.0
2022-06-14 9:00 PM	12.8	12.6	12.4	12.6
2022-06-14 10:00 PM	12.6	12.4	12.3	12.4
2022-06-14 11:00 PM	12.6	12.4	12.4	12.5
2022-06-15 12:00 AM	12.7	12.5	12.5	12.6
2022-06-15 1:00 AM	12.8	12.6	12.6	12.7
2022-06-15 2:00 AM	12.7	12.6	12.5	12.6
2022-06-15 3:00 AM	12.5	12.4	12.4	12.4

2022-06-15 4:00 AM	12.5	12.4	12.4	12.4
2022-06-15 5:00 AM	12.4	12.4	12.4	12.4
2022-06-15 6:00 AM	12.4	12.3	12.3	12.3
2022-06-15 7:00 AM	12.4	12.3	12.3	12.3
2022-06-15 8:00 AM	12.5	12.5	12.4	12.5
2022-06-15 9:00 AM	13.1	13.0	12.7	12.9
2022-06-15 10:00 AM	14.1	14.3	13.4	13.9
2022-06-15 11:00 AM	16.9	17.6	14.6	16.4
2022-06-15 12:00 PM	17.9	19.0	15.9	17.6
2022-06-15 1:00 PM	18.9	20.1	16.7	18.6
2022-06-15 2:00 PM	17.1	17.5	16.1	16.9
2022-06-15 3:00 PM	17.6	18.2	18.5	18.1
2022-06-15 4:00 PM	16.9	17.5	19.4	17.9
2022-06-15 5:00 PM	17.4	18.1	18.9	18.1
2022-06-15 6:00 PM	17.2	17.5	17.1	17.3
2022-06-15 7:00 PM	16.7	16.9	16.4	16.7
2022-06-15 8:00 PM	16.1	16.0	15.8	16.0
2022-06-15 9:00 PM	15.7	15.5	15.4	15.5
2022-06-15 10:00 PM	14.4	13.9	14.1	14.1
2022-06-15 11:00 PM	13.7	13.2	13.5	13.5
2022-06-16 12:00 AM	13.4	12.8	13.3	13.2
2022-06-16 1:00 AM	13.4	13.0	13.2	13.2
2022-06-16 2:00 AM	13.4	13.1	13.2	13.2
2022-06-16 3:00 AM	13.1	12.7	13.0	12.9
2022-06-16 4:00 AM	12.6	12.2	12.5	12.4
2022-06-16 5:00 AM	12.2	11.7	12.2	12.0
2022-06-16 6:00 AM	11.9	11.4	11.9	11.7
2022-06-16 7:00 AM	11.8	11.3	11.8	11.6
2022-06-16 8:00 AM	12.0	11.5	12.0	11.8
2022-06-16 9:00 AM	12.4	12.1	12.2	12.2
2022-06-16 10:00 AM	13.2	13.2	12.9	13.1
2022-06-16 11:00 AM	14.8	15.2	13.7	14.6
2022-06-16 12:00 PM	19.9	22.3	15.2	19.1
2022-06-16 1:00 PM	19.4	19.4	16.2	18.3
2022-06-16 2:00 PM	18.5	18.3	16.5	17.8
2022-06-16 3:00 PM	17.8	18.4	16.8	17.7
2022-06-16 4:00 PM	17.6	18.2	18.2	18.0
2022-06-16 5:00 PM	17.7	18.5	18.4	18.2
2022-06-16 6:00 PM	17.5	18.4	18.5	18.1
2022-06-16 7:00 PM	17.4	18.1	17.1	17.5
2022-06-16 8:00 PM	16.8	17.2	16.5	16.8
2022-06-16 9:00 PM	16.4	16.8	16.2	16.5
2022-06-16 10:00 PM	15.9	16.1	15.8	15.9
2022-06-16 11:00 PM	15.5	15.7	15.4	15.5
2022-06-17 12:00 AM	15.2	15.3	15.2	15.2

2022-06-17 1:00 AM	15.1	15.1	15.1	15.1
2022-06-17 2:00 AM	14.7	14.7	14.8	14.7
2022-06-17 3:00 AM	14.3	14.2	14.5	14.3
2022-06-17 4:00 AM	14.1	14.0	14.3	14.1
2022-06-17 5:00 AM	14.1	14.1	14.3	14.2
2022-06-17 6:00 AM	14.0	14.0	14.2	14.1
2022-06-17 7:00 AM	14.2	14.3	14.3	14.3
2022-06-17 8:00 AM	14.4	14.8	14.4	14.5
2022-06-17 9:00 AM	14.5	14.9	14.4	14.6
2022-06-17 10:00 AM	15.7	17.9	14.6	16.1
2022-06-17 11:00 AM	20.0	24.1	14.7	19.6
2022-06-17 12:00 PM	22.0	24.8	15.4	20.7
2022-06-17 1:00 PM	23.4	21.5	16.1	20.3
2022-06-17 2:00 PM	20.9	20.2	17.3	19.5
2022-06-17 3:00 PM	18.8	20.2	21.2	20.1
2022-06-17 4:00 PM	18.5	20.1	24.3	21.0
2022-06-17 5:00 PM	18.3	20.5	26.0	21.6
2022-06-17 6:00 PM	17.8	19.3	21.2	19.4
2022-06-17 7:00 PM	16.9	17.5	17.3	17.2
2022-06-17 8:00 PM	16.5	17.1	16.6	16.7
2022-06-17 9:00 PM	16.0	16.2	16.3	16.2
2022-06-17 10:00 PM	15.0	14.8	15.4	15.1
2022-06-17 11:00 PM	14.0	13.3	14.5	13.9
2022-06-18 12:00 AM	13.4	12.7	14.0	13.4
2022-06-18 1:00 AM	12.7	11.8	13.1	12.5
2022-06-18 2:00 AM	12.2	11.4	12.7	12.1
2022-06-18 3:00 AM	11.8	10.9	12.3	11.7
2022-06-18 4:00 AM	11.2	9.9	11.9	11.0
2022-06-18 5:00 AM	10.6	9.2	11.1	10.3
2022-06-18 6:00 AM	9.9	8.4	10.3	9.5
2022-06-18 7:00 AM	9.7	8.1	10.4	9.4
2022-06-18 8:00 AM	10.3	9.2	10.8	10.1
2022-06-18 9:00 AM	11.7	11.3	11.8	11.6
2022-06-18 10:00 AM	14.3	16.0	12.7	14.3
2022-06-18 11:00 AM	19.2	22.3	13.7	18.4
2022-06-18 12:00 PM	20.6	22.1	14.4	19.0
2022-06-18 1:00 PM	21.4	19.8	15.2	18.8
2022-06-18 2:00 PM	20.2	18.9	16.8	18.6
2022-06-18 3:00 PM	18.0	18.3	19.9	18.7
2022-06-18 4:00 PM	17.2	17.4	23.1	19.2
2022-06-18 5:00 PM	16.6	17.0	23.8	19.1
2022-06-18 6:00 PM	16.1	16.2	19.7	17.3
2022-06-18 7:00 PM	15.3	14.8	16.0	15.4
2022-06-18 8:00 PM	15.2	14.6	15.4	15.1
2022-06-18 9:00 PM	14.6	13.8	14.7	14.4

2022-06-18 10:00 PM	13.7	12.6	14.0	13.4
2022-06-18 11:00 PM	13.0	11.7	13.4	12.7
2022-06-19 12:00 AM	12.4	10.9	12.8	12.0
2022-06-19 1:00 AM	12.2	10.9	12.5	11.9
2022-06-19 2:00 AM	12.1	10.8	12.4	11.8
2022-06-19 3:00 AM	12.4	11.6	12.6	12.2
2022-06-19 4:00 AM	12.1	11.1	12.3	11.8
2022-06-19 5:00 AM	11.8	10.9	12.2	11.6
2022-06-19 6:00 AM	11.8	11.1	12.0	11.6
2022-06-19 7:00 AM	11.5	10.8	11.7	11.3
2022-06-19 8:00 AM	11.7	11.1	11.6	11.5
2022-06-19 9:00 AM	11.7	11.2	11.7	11.5
2022-06-19 10:00 AM	10.3	9.6	10.6	10.2
2022-06-19 11:00 AM	9.8	9.7	10.4	10.0
2022-06-19 12:00 PM	10.4	10.3	10.8	10.5
2022-06-19 1:00 PM	11.9	12.2	11.9	12.0
2022-06-19 2:00 PM	13.5	14.1	13.1	13.6
2022-06-19 3:00 PM	14.0	14.8	15.4	14.7
2022-06-19 4:00 PM	13.9	14.4	15.1	14.5
2022-06-19 5:00 PM	14.3	15.1	17.2	15.5
2022-06-19 6:00 PM	14.3	14.7	14.6	14.5
2022-06-19 7:00 PM	14.2	14.3	14.0	14.2
2022-06-19 8:00 PM	13.8	13.7	13.5	13.7
2022-06-19 9:00 PM	13.0	12.6	12.9	12.8
2022-06-19 10:00 PM	12.1	11.5	12.1	11.9
2022-06-19 11:00 PM	11.9	11.2	11.8	11.6
2022-06-20 12:00 AM	11.8	11.2	11.8	11.6
2022-06-20 1:00 AM	11.9	11.4	11.9	11.7
2022-06-20 2:00 AM	11.8	11.3	11.8	11.6
2022-06-20 3:00 AM	11.8	11.3	11.7	11.6
2022-06-20 4:00 AM	11.8	11.4	11.8	11.7
2022-06-20 5:00 AM	11.5	11.1	11.6	11.4
2022-06-20 6:00 AM	11.3	10.8	11.5	11.2
2022-06-20 7:00 AM	11.6	11.2	11.7	11.5
2022-06-20 8:00 AM	12.2	12.1	12.0	12.1
2022-06-20 9:00 AM	12.3	12.3	11.9	12.2
2022-06-20 10:00 AM	14.2	15.5	12.3	14.0
2022-06-20 11:00 AM	19.5	22.0	12.9	18.1
2022-06-20 12:00 PM	21.9	23.9	13.9	19.9
2022-06-20 1:00 PM	23.0	21.1	15.1	19.7
2022-06-20 2:00 PM	21.2	20.2	17.0	19.5
2022-06-20 3:00 PM	19.0	19.9	20.5	19.8
2022-06-20 4:00 PM	18.4	19.3	23.9	20.5
2022-06-20 5:00 PM	18.0	19.2	25.3	20.8
2022-06-20 6:00 PM	18.1	19.1	21.5	19.6

2022-06-20 7:00 PM	17.8	18.2	18.0	18.0
2022-06-20 8:00 PM	17.5	17.8	17.4	17.6
2022-06-20 9:00 PM	17.5	17.8	17.1	17.5
2022-06-20 10:00 PM	17.2	17.4	16.8	17.1
2022-06-20 11:00 PM	16.6	16.5	16.4	16.5
2022-06-21 12:00 AM	16.2	16.1	16.0	16.1
2022-06-21 1:00 AM	16.0	15.9	15.8	15.9
2022-06-21 2:00 AM	16.0	15.9	15.8	15.9
2022-06-21 3:00 AM	17.4	18.0	16.9	17.4
2022-06-21 4:00 AM	17.2	17.7	16.8	17.2
2022-06-21 5:00 AM	17.0	17.4	16.6	17.0
2022-06-21 6:00 AM	16.9	17.3	16.5	16.9
2022-06-21 7:00 AM	17.1	17.5	16.6	17.1
2022-06-21 8:00 AM	17.1	17.3	16.6	17.0
2022-06-21 9:00 AM	16.8	16.9	16.2	16.6
2022-06-21 10:00 AM	18.3	19.0	16.6	18.0
2022-06-21 11:00 AM	19.3	20.0	17.2	18.8
2022-06-21 12:00 PM	22.9	24.4	18.4	21.9
2022-06-21 1:00 PM	25.1	25.3	20.1	23.5
2022-06-21 2:00 PM	24.5	24.8	21.3	23.5
2022-06-21 3:00 PM	23.8	25.2	23.2	24.1
2022-06-21 4:00 PM	23.5	25.3	27.5	25.4
2022-06-21 5:00 PM	22.9	24.2	28.3	25.1
2022-06-21 6:00 PM	22.0	22.9	24.8	23.2
2022-06-21 7:00 PM	20.5	20.6	21.1	20.7
2022-06-21 8:00 PM	19.9	20.1	20.3	20.1
2022-06-21 9:00 PM	19.5	19.8	19.8	19.7
2022-06-21 10:00 PM	19.2	19.3	19.3	19.3
2022-06-21 11:00 PM	18.7	18.7	18.9	18.8
2022-06-22 12:00 AM	18.3	18.2	18.5	18.3
2022-06-22 1:00 AM	17.9	17.8	18.1	17.9
2022-06-22 2:00 AM	17.6	17.4	17.7	17.6
2022-06-22 3:00 AM	17.4	17.3	17.6	17.4
2022-06-22 4:00 AM	16.9	16.9	17.0	16.9
2022-06-22 5:00 AM	16.1	15.8	16.4	16.1
2022-06-22 6:00 AM	15.7	15.3	16.1	15.7
2022-06-22 7:00 AM	15.6	15.2	15.9	15.6
2022-06-22 8:00 AM	15.9	15.7	15.9	15.8
2022-06-22 9:00 AM	15.4	15.3	15.4	15.4
2022-06-22 10:00 AM	16.4	17.5	15.3	16.4
2022-06-22 11:00 AM	21.2	23.9	15.8	20.3
2022-06-22 12:00 PM	23.6	25.6	16.6	21.9
2022-06-22 1:00 PM	24.5	23.0	17.3	21.6
2022-06-22 2:00 PM	23.3	22.2	18.8	21.4
2022-06-22 3:00 PM	21.2	21.9	20.5	21.2

2022-06-22 4:00 PM	21.2	22.0	22.0	21.7
2022-06-23 11:00 AM	22.1	24.6	15.7	20.8
2022-06-23 12:00 PM	25.1	26.4	16.6	22.7
2022-06-23 1:00 PM	25.9	23.6	17.5	22.3
2022-06-23 2:00 PM	23.8	22.4	18.9	21.7
2022-06-23 3:00 PM	22.0	22.7	21.3	22.0
2022-06-23 4:00 PM	21.9	22.9	23.7	22.8
2022-06-23 5:00 PM	22.1	23.7	25.8	23.9
2022-06-23 6:00 PM	21.6	22.4	23.2	22.4
2022-06-23 7:00 PM	20.7	20.6	20.7	20.7
2022-06-23 8:00 PM	20.8	20.9	20.3	20.7
2022-06-23 9:00 PM	20.0	19.9	19.7	19.9
2022-06-23 10:00 PM	19.4	19.2	19.2	19.3
2022-06-23 11:00 PM	18.8	18.5	18.8	18.7
2022-06-24 12:00 AM	18.2	17.7	18.2	18.0
2022-06-24 1:00 AM	17.2	16.4	17.3	17.0
2022-06-24 2:00 AM	16.9	16.1	17.0	16.7
2022-06-24 3:00 AM	16.5	15.8	16.8	16.4
2022-06-24 4:00 AM	15.4	14.3	15.9	15.2
2022-06-24 5:00 AM	14.9	13.8	15.5	14.7
2022-06-24 6:00 AM	14.7	13.5	15.3	14.5
2022-06-24 7:00 AM	14.7	13.8	15.3	14.6
2022-06-24 8:00 AM	15.3	14.8	15.6	15.2
2022-06-24 9:00 AM	16.5	16.4	16.2	16.4
2022-06-24 10:00 AM	18.1	18.6	16.4	17.7
2022-06-24 11:00 AM	22.6	23.5	16.7	20.9
2022-06-24 12:00 PM	24.6	25.1	17.5	22.4
2022-06-24 1:00 PM	26.2	24.2	18.6	23.0
2022-06-24 2:00 PM	24.7	23.3	20.1	22.7
2022-06-24 3:00 PM	22.6	22.9	21.8	22.4
2022-06-24 4:00 PM	21.8	22.2	23.7	22.6
2022-06-24 5:00 PM	21.0	21.7	25.0	22.6
2022-06-24 6:00 PM	20.5	21.0	22.4	21.3
2022-06-24 7:00 PM	19.6	19.3	19.7	19.5
2022-06-24 8:00 PM	19.2	18.8	19.1	19.0
2022-06-24 9:00 PM	18.8	18.5	18.7	18.7
2022-06-24 10:00 PM	18.2	17.7	18.2	18.0
2022-06-24 11:00 PM	17.6	17.0	17.8	17.5
2022-06-25 12:00 AM	17.1	16.5	17.3	17.0
2022-06-25 1:00 AM	16.7	16.0	17.0	16.6
2022-06-25 2:00 AM	16.5	15.9	16.8	16.4
2022-06-25 3:00 AM	16.5	16.1	16.8	16.5
2022-06-25 4:00 AM	16.5	16.2	16.7	16.5
2022-06-25 5:00 AM	16.1	15.7	16.4	16.1
2022-06-25 6:00 AM	15.8	15.4	16.1	15.8

2022-06-25 7:00 AM	15.7	15.2	15.9	15.6
2022-06-25 8:00 AM	15.7	15.3	15.8	15.6
2022-06-25 9:00 AM	16.2	15.9	15.8	16.0
2022-06-25 10:00 AM	16.4	16.2	16.1	16.2
2022-06-25 11:00 AM	16.6	16.6	16.3	16.5
2022-06-25 12:00 PM	16.5	16.3	16.1	16.3
2022-06-25 1:00 PM	16.6	16.5	16.2	16.4
2022-06-25 2:00 PM	17.5	17.4	16.6	17.2
2022-06-25 3:00 PM	18.7	19.1	18.3	18.7
2022-06-25 4:00 PM	18.9	19.4	20.1	19.5
2022-06-25 5:00 PM	18.2	18.3	18.6	18.4
2022-06-25 6:00 PM	17.9	17.9	17.8	17.9
2022-06-25 7:00 PM	17.6	17.5	17.4	17.5
2022-06-25 8:00 PM	17.5	17.2	17.1	17.3
2022-06-25 9:00 PM	17.1	16.9	16.9	17.0
2022-06-25 10:00 PM	16.4	15.9	16.5	16.3
2022-06-25 11:00 PM	15.8	15.1	16.0	15.6
2022-06-26 12:00 AM	15.3	14.5	15.7	15.2
2022-06-26 1:00 AM	15.1	14.4	15.5	15.0
2022-06-26 2:00 AM	15.0	14.3	15.3	14.9
2022-06-26 3:00 AM	15.1	14.6	15.5	15.1
2022-06-26 4:00 AM	14.6	14.1	15.2	14.6
2022-06-26 5:00 AM	14.2	13.4	14.8	14.1
2022-06-26 6:00 AM	13.6	12.6	14.3	13.5
2022-06-26 7:00 AM	13.3	12.4	14.1	13.3
2022-06-26 8:00 AM	13.6	12.8	14.2	13.5
2022-06-26 9:00 AM	14.4	14.1	14.6	14.4
2022-06-26 10:00 AM	15.5	15.8	14.7	15.3
2022-06-26 11:00 AM	18.0	18.4	15.3	17.2
2022-06-26 12:00 PM	18.8	18.6	15.6	17.7
2022-06-26 1:00 PM	18.7	18.2	16.1	17.7
2022-06-26 2:00 PM	18.1	17.7	16.2	17.3
2022-06-26 3:00 PM	17.6	17.4	16.3	17.1
2022-06-26 4:00 PM	16.7	16.6	17.2	16.8
2022-06-26 5:00 PM	16.5	17.0	17.9	17.1
2022-06-26 6:00 PM	15.9	16.1	16.3	16.1
2022-06-26 7:00 PM	15.2	15.0	15.0	15.1
2022-06-26 8:00 PM	15.0	14.7	14.7	14.8
2022-06-26 9:00 PM	14.5	14.1	14.4	14.3
2022-06-26 10:00 PM	14.0	13.5	14.1	13.9
2022-06-26 11:00 PM	13.4	12.9	13.8	13.4
2022-06-27 12:00 AM	13.2	12.6	13.6	13.1
2022-06-27 1:00 AM	13.0	12.4	13.4	12.9
2022-06-27 2:00 AM	13.0	12.4	13.4	12.9
2022-06-27 3:00 AM	12.7	12.2	13.3	12.7

2022-06-27 4:00 AM	12.4	11.9	13.1	12.5
2022-06-27 5:00 AM	12.3	11.7	13.0	12.3
2022-06-27 6:00 AM	11.9	11.0	12.5	11.8
2022-06-27 7:00 AM	11.2	9.9	12.0	11.0
2022-06-27 8:00 AM	11.4	10.4	12.1	11.3
2022-06-27 9:00 AM	12.0	11.4	12.5	12.0
2022-06-27 10:00 AM	13.9	14.2	13.0	13.7
2022-06-27 11:00 AM	19.9	20.3	13.9	18.0
2022-06-27 12:00 PM	22.7	22.5	14.8	20.0
2022-06-27 1:00 PM	23.2	21.0	16.1	20.1
2022-06-27 2:00 PM	20.9	19.3	17.1	19.1
2022-06-27 3:00 PM	19.5	19.3	17.6	18.8
2022-06-27 4:00 PM	19.0	19.0	18.8	18.9
2022-06-27 5:00 PM	18.8	19.7	20.9	19.8
2022-06-27 6:00 PM	18.7	19.6	20.4	19.6
2022-06-27 7:00 PM	18.0	17.8	17.9	17.9
2022-06-27 8:00 PM	17.6	17.3	17.4	17.4
2022-06-27 9:00 PM	17.0	16.7	17.0	16.9
2022-06-27 10:00 PM	16.3	15.7	16.4	16.1
2022-06-27 11:00 PM	15.2	14.3	15.5	15.0
2022-06-28 12:00 AM	14.6	13.5	15.0	14.4
2022-06-28 1:00 AM	14.4	13.4	14.9	14.2
2022-06-28 2:00 AM	15.2	14.8	15.4	15.1
2022-06-28 3:00 AM	15.3	15.0	15.5	15.3
2022-06-28 4:00 AM	15.5	15.3	15.6	15.5
2022-06-28 5:00 AM	15.3	15.1	15.5	15.3
2022-06-28 6:00 AM	15.1	14.8	15.3	15.1
2022-06-28 7:00 AM	15.0	14.7	15.3	15.0
2022-06-28 8:00 AM	15.2	15.1	15.4	15.2
2022-06-28 9:00 AM	15.3	15.2	15.3	15.3
2022-06-28 10:00 AM	16.5	16.8	15.6	16.3
2022-06-28 11:00 AM	18.1	18.7	16.0	17.6
2022-06-28 12:00 PM	17.2	17.3	15.9	16.8
2022-06-28 1:00 PM	16.6	16.6	15.6	16.3
2022-06-28 2:00 PM	16.3	16.1	15.4	15.9
2022-06-28 3:00 PM	17.4	17.6	16.1	17.0
2022-06-28 4:00 PM	17.8	18.4	18.1	18.1
2022-06-28 5:00 PM	17.5	18.7	19.4	18.5
2022-06-28 6:00 PM	17.1	18.1	18.2	17.8
2022-06-28 7:00 PM	16.2	16.4	16.3	16.3
2022-06-28 8:00 PM	16.0	16.0	15.9	16.0
2022-06-28 9:00 PM	15.5	15.5	15.5	15.5
2022-06-28 10:00 PM	15.0	14.7	15.3	15.0
2022-06-28 11:00 PM	14.1	13.2	14.7	14.0
2022-06-29 12:00 AM	13.4	12.4	14.0	13.3

2022-06-29 1:00 AM	12.7	11.7	13.6	12.7
2022-06-29 2:00 AM	12.0	10.8	12.9	11.9
2022-06-29 3:00 AM	11.5	10.3	12.5	11.4
2022-06-29 4:00 AM	11.3	10.0	12.3	11.2
2022-06-29 5:00 AM	10.9	9.6	11.7	10.7
2022-06-29 6:00 AM	10.5	9.1	11.3	10.3
2022-06-29 7:00 AM	9.9	8.6	11.0	9.8
2022-06-29 8:00 AM	10.2	9.3	11.3	10.3
2022-06-29 9:00 AM	11.5	11.0	12.1	11.5
2022-06-29 10:00 AM	13.7	14.2	12.9	13.6
2022-06-29 11:00 AM	19.8	20.8	13.9	18.2
2022-06-29 12:00 PM	21.9	22.1	14.9	19.6
2022-06-29 1:00 PM	20.5	19.4	15.6	18.5
2022-06-29 2:00 PM	20.1	19.1	16.6	18.6
2022-06-29 3:00 PM	18.3	18.1	16.8	17.7
2022-06-29 4:00 PM	18.1	17.8	16.6	17.5
2022-06-29 5:00 PM	17.5	16.9	16.0	16.8
2022-06-29 6:00 PM	17.0	16.5	15.6	16.4
2022-06-29 7:00 PM	16.5	15.8	15.3	15.9
2022-06-29 8:00 PM	15.8	15.0	14.9	15.2
2022-06-29 9:00 PM	15.2	14.4	14.6	14.7
2022-06-29 10:00 PM	14.6	13.6	14.2	14.1
2022-06-29 11:00 PM	14.1	13.2	14.0	13.8
2022-06-30 12:00 AM	13.8	13.1	13.9	13.6
2022-06-30 1:00 AM	13.5	12.9	13.6	13.3
2022-06-30 2:00 AM	13.2	12.5	13.2	13.0
2022-06-30 3:00 AM	13.2	12.7	13.2	13.0
2022-06-30 4:00 AM	13.2	12.7	13.2	13.0
2022-06-30 5:00 AM	13.3	12.9	13.3	13.2
2022-06-30 6:00 AM	13.3	13.0	13.3	13.2
2022-06-30 7:00 AM	13.4	13.1	13.4	13.3
2022-06-30 8:00 AM	13.7	13.4	13.5	13.5
2022-06-30 9:00 AM	13.9	13.7	13.7	13.8
2022-06-30 10:00 AM	14.1	14.0	13.9	14.0
2022-06-30 11:00 AM	15.2	15.3	14.1	14.9
2022-06-30 12:00 PM	19.2	19.5	15.7	18.1
2022-06-30 1:00 PM	20.6	20.1	16.5	19.1
2022-06-30 2:00 PM	20.8	20.1	17.1	19.3
2022-06-30 3:00 PM	19.6	20.2	18.2	19.3
2022-06-30 4:00 PM	19.9	20.9	20.0	20.3
2022-06-30 5:00 PM	20.5	22.1	21.7	21.4
2022-06-30 6:00 PM	20.1	21.3	20.3	20.6
2022-06-30 7:00 PM	19.4	20.0	18.9	19.4
2022-06-30 8:00 PM	19.1	19.6	18.5	19.1
2022-06-30 9:00 PM	18.5	18.7	18.1	18.4

2022-06-30 10:00 PM	17.8	17.8	17.6	17.7
2022-06-30 11:00 PM	16.9	16.7	17.1	16.9
2022-07-01 12:00 AM	16.3	15.8	16.6	16.2
2022-07-01 1:00 AM	16.0	15.7	16.3	16.0
2022-07-01 2:00 AM	15.4	15.0	15.9	15.4
2022-07-01 3:00 AM	14.6	14.0	15.3	14.6
2022-07-01 4:00 AM	13.9	13.0	14.7	13.9
2022-07-01 5:00 AM	12.9	12.1	14.0	13.0
2022-07-01 6:00 AM	12.7	11.8	13.8	12.8
2022-07-01 7:00 AM	12.5	11.6	13.4	12.5
2022-07-01 8:00 AM	12.4	11.7	13.2	12.4
2022-07-01 9:00 AM	13.1	12.7	13.7	13.2
2022-07-01 10:00 AM	15.1	15.1	14.4	14.9
2022-07-01 11:00 AM	19.9	19.8	15.1	18.3
2022-07-01 12:00 PM	21.3	21.0	15.9	19.4
2022-07-01 1:00 PM	18.9	18.5	16.1	17.8
2022-07-01 2:00 PM	18.3	18.0	16.3	17.5
2022-07-01 3:00 PM	17.8	17.6	16.5	17.3
2022-07-01 4:00 PM	19.1	19.9	19.4	19.5
2022-07-01 5:00 PM	18.9	20.2	20.6	19.9
2022-07-01 6:00 PM	18.2	19.3	19.0	18.8
2022-07-01 7:00 PM	17.4	17.7	17.2	17.4
2022-07-01 8:00 PM	17.0	17.3	16.8	17.0
2022-07-01 9:00 PM	16.7	16.8	16.4	16.6
2022-07-01 10:00 PM	16.2	16.0	16.2	16.1
2022-07-01 11:00 PM	15.5	15.2	15.8	15.5
2022-07-02 12:00 AM	14.9	14.3	15.4	14.9
2022-07-02 1:00 AM	14.4	13.9	15.1	14.5
2022-07-02 2:00 AM	13.7	12.9	14.5	13.7
2022-07-02 3:00 AM	12.9	12.1	13.9	13.0
2022-07-02 4:00 AM	12.4	11.5	13.4	12.4
2022-07-02 5:00 AM	11.9	11.0	13.0	12.0
2022-07-02 6:00 AM	11.7	10.9	12.7	11.8
2022-07-02 7:00 AM	11.2	10.4	12.3	11.3
2022-07-02 8:00 AM	11.5	10.8	12.4	11.6
2022-07-02 9:00 AM	12.3	11.9	12.9	12.4
2022-07-02 10:00 AM	14.1	14.2	13.4	13.9
2022-07-02 11:00 AM	19.6	20.3	14.3	18.1
2022-07-02 12:00 PM	21.4	21.7	15.3	19.5
2022-07-02 1:00 PM	22.0	20.9	16.1	19.7
2022-07-02 2:00 PM	20.7	20.0	16.6	19.1
2022-07-02 3:00 PM	18.8	18.8	16.6	18.1
2022-07-02 4:00 PM	18.1	18.0	16.3	17.5
2022-07-02 5:00 PM	17.9	17.8	16.5	17.4
2022-07-02 6:00 PM	17.7	18.3	17.6	17.9

2022-07-02 7:00 PM	16.8	16.7	16.3	16.6
2022-07-02 8:00 PM	16.5	16.3	16.0	16.3
2022-07-02 9:00 PM	16.0	15.8	15.8	15.9
2022-07-02 10:00 PM	15.6	15.1	15.6	15.4
2022-07-02 11:00 PM	15.0	14.3	15.1	14.8
2022-07-03 12:00 AM	14.5	13.8	14.7	14.3
2022-07-03 1:00 AM	14.0	13.4	14.4	13.9
2022-07-03 2:00 AM	13.2	12.5	13.8	13.2
2022-07-03 3:00 AM	12.7	11.9	13.5	12.7
2022-07-03 4:00 AM	12.1	11.3	13.1	12.2
2022-07-03 5:00 AM	11.7	11.0	12.7	11.8
2022-07-03 6:00 AM	11.6	10.7	12.4	11.6
2022-07-03 7:00 AM	12.0	11.4	12.7	12.0
2022-07-03 8:00 AM	12.8	12.5	13.1	12.8
2022-07-03 9:00 AM	13.9	13.8	13.5	13.7
2022-07-03 10:00 AM	15.4	15.5	14.1	15.0
2022-07-03 11:00 AM	18.2	18.6	15.0	17.3
2022-07-03 12:00 PM	21.1	21.3	16.0	19.5
2022-07-03 1:00 PM	22.6	21.5	17.0	20.4
2022-07-03 2:00 PM	21.7	21.1	18.0	20.3
2022-07-03 3:00 PM	20.3	21.1	20.5	20.6
2022-07-03 4:00 PM	20.0	21.1	22.2	21.1
2022-07-03 5:00 PM	19.4	20.1	20.4	20.0
2022-07-03 6:00 PM	18.5	18.4	17.9	18.3
2022-07-03 7:00 PM	18.1	17.9	17.4	17.8
2022-07-03 8:00 PM	17.8	17.6	17.2	17.5
2022-07-03 9:00 PM	17.3	17.0	16.8	17.0
2022-07-03 10:00 PM	16.3	15.7	16.1	16.0
2022-07-03 11:00 PM	15.9	15.3	15.8	15.7
2022-07-04 12:00 AM	15.2	14.3	15.3	14.9
2022-07-04 1:00 AM	14.5	13.5	14.8	14.3
2022-07-04 2:00 AM	14.1	13.0	14.5	13.9
2022-07-04 3:00 AM	13.6	12.5	14.1	13.4
2022-07-04 4:00 AM	13.2	12.1	13.9	13.1
2022-07-04 5:00 AM	13.0	12.0	13.6	12.9
2022-07-04 6:00 AM	12.7	11.8	13.5	12.7
2022-07-04 7:00 AM	12.8	12.0	13.5	12.8
2022-07-04 8:00 AM	13.5	13.1	13.8	13.5
2022-07-04 9:00 AM	14.3	14.1	14.2	14.2
2022-07-04 10:00 AM	15.1	14.7	14.4	14.7
2022-07-04 11:00 AM	15.7	15.4	14.4	15.2
2022-07-04 12:00 PM	15.6	15.2	14.6	15.1
2022-07-04 1:00 PM	16.8	16.7	15.2	16.2
2022-07-04 2:00 PM	16.4	16.2	15.2	15.9
2022-07-04 3:00 PM	15.9	15.5	15.0	15.5

2022-07-04 4:00 PM	15.3	14.8	14.7	14.9
2022-07-04 5:00 PM	15.5	15.2	14.9	15.2
2022-07-04 6:00 PM	15.8	15.5	15.0	15.4
2022-07-04 7:00 PM	15.6	15.3	14.9	15.3
2022-07-04 8:00 PM	15.3	15.0	14.8	15.0
2022-07-04 9:00 PM	14.8	14.4	14.5	14.6
2022-07-04 10:00 PM	14.7	14.4	14.5	14.5
2022-07-04 11:00 PM	14.7	14.4	14.5	14.5
2022-07-05 12:00 AM	14.5	14.2	14.4	14.4
2022-07-05 1:00 AM	14.6	14.3	14.5	14.5
2022-07-05 2:00 AM	14.7	14.5	14.6	14.6
2022-07-05 3:00 AM	14.7	14.6	14.6	14.6
2022-07-05 4:00 AM	14.7	14.6	14.6	14.6
2022-07-05 5:00 AM	14.7	14.5	14.6	14.6
2022-07-05 6:00 AM	14.6	14.5	14.6	14.6
2022-07-05 7:00 AM	14.7	14.5	14.6	14.6
2022-07-05 8:00 AM	14.7	14.6	14.6	14.6
2022-07-05 9:00 AM	14.6	14.5	14.5	14.5
2022-07-05 10:00 AM	14.7	14.6	14.6	14.6
2022-07-05 11:00 AM	14.9	14.8	14.7	14.8
2022-07-05 12:00 PM	15.1	15.1	14.8	15.0
2022-07-05 1:00 PM	15.6	15.6	15.1	15.4
2022-07-05 2:00 PM	16.4	16.7	15.7	16.3
2022-07-05 3:00 PM	16.6	16.9	15.8	16.4
2022-07-05 4:00 PM	17.2	17.5	16.1	16.9
2022-07-05 5:00 PM	17.3	17.9	16.7	17.3
2022-07-05 6:00 PM	17.1	17.6	16.6	17.1
2022-07-05 7:00 PM	16.6	16.8	16.4	16.6
2022-07-05 8:00 PM	16.3	16.4	16.2	16.3
2022-07-05 9:00 PM	16.1	16.2	16.1	16.1
2022-07-05 10:00 PM	15.9	15.9	15.9	15.9
2022-07-05 11:00 PM	15.4	15.2	15.6	15.4
2022-07-06 12:00 AM	14.9	14.4	15.1	14.8
2022-07-06 1:00 AM	14.4	13.6	14.7	14.2
2022-07-06 2:00 AM	14.0	13.3	14.4	13.9
2022-07-06 3:00 AM	13.7	12.9	14.1	13.6
2022-07-06 4:00 AM	13.3	12.4	14.0	13.2
2022-07-06 5:00 AM	13.0	12.1	13.7	12.9
2022-07-06 6:00 AM	12.8	11.7	13.3	12.6
2022-07-06 7:00 AM	12.5	11.4	13.1	12.3
2022-07-06 8:00 AM	12.6	11.7	13.0	12.4
2022-07-06 9:00 AM	13.2	12.7	13.5	13.1
2022-07-06 10:00 AM	14.8	15.1	14.2	14.7
2022-07-06 11:00 AM	19.1	21.0	15.1	18.4
2022-07-06 12:00 PM	21.1	23.1	16.1	20.1

2022-07-06 1:00 PM	22.5	22.1	17.0	20.5
2022-07-06 2:00 PM	21.8	21.3	18.0	20.4
2022-07-06 3:00 PM	20.3	21.0	20.2	20.5
2022-07-06 4:00 PM	19.9	20.8	21.8	20.8
2022-07-06 5:00 PM	19.3	20.7	21.9	20.6
2022-07-06 6:00 PM	18.6	19.8	20.4	19.6
2022-07-06 7:00 PM	17.4	17.3	17.8	17.5
2022-07-06 8:00 PM	16.8	16.5	17.0	16.8
2022-07-06 9:00 PM	16.5	16.0	16.7	16.4
2022-07-06 10:00 PM	16.1	15.4	16.3	15.9
2022-07-06 11:00 PM	15.2	14.3	15.6	15.0
2022-07-07 12:00 AM	14.8	13.6	15.2	14.5
2022-07-07 1:00 AM	14.4	13.2	14.7	14.1
2022-07-07 2:00 AM	13.9	12.7	14.3	13.6
2022-07-07 3:00 AM	13.5	12.3	14.0	13.3
2022-07-07 4:00 AM	12.9	11.8	13.6	12.8
2022-07-07 5:00 AM	12.5	11.3	13.1	12.3
2022-07-07 6:00 AM	12.5	11.3	13.0	12.3
2022-07-07 7:00 AM	12.7	11.8	13.1	12.5
2022-07-07 8:00 AM	13.1	12.5	13.5	13.0
2022-07-07 9:00 AM	13.7	13.4	14.0	13.7
2022-07-07 10:00 AM	15.2	15.4	14.5	15.0
2022-07-07 11:00 AM	19.0	20.5	15.3	18.3
2022-07-07 12:00 PM	21.1	23.5	16.5	20.4
2022-07-07 1:00 PM	22.1	22.5	17.6	20.7
2022-07-07 2:00 PM	21.4	21.7	18.3	20.5
2022-07-07 3:00 PM	21.1	22.0	20.2	21.1
2022-07-07 4:00 PM	20.7	21.7	21.7	21.4
2022-07-07 5:00 PM	20.1	21.3	21.7	21.0
2022-07-07 6:00 PM	19.8	20.6	20.4	20.3
2022-07-07 7:00 PM	19.2	19.4	18.9	19.2
2022-07-07 8:00 PM	18.7	18.6	18.4	18.6
2022-07-07 9:00 PM	18.0	18.0	17.9	18.0
2022-07-07 10:00 PM	17.6	17.4	17.5	17.5
2022-07-07 11:00 PM	17.2	17.0	17.2	17.1
2022-07-08 12:00 AM	16.8	16.5	16.9	16.7
2022-07-08 1:00 AM	16.5	16.1	16.6	16.4
2022-07-08 2:00 AM	15.9	15.3	16.2	15.8
2022-07-08 3:00 AM	15.4	14.7	15.8	15.3
2022-07-08 4:00 AM	14.8	13.9	15.3	14.7
2022-07-08 5:00 AM	14.3	13.3	14.8	14.1
2022-07-08 6:00 AM	13.6	12.5	14.2	13.4
2022-07-08 7:00 AM	13.1	12.2	13.8	13.0
2022-07-08 8:00 AM	13.4	12.7	14.0	13.4
2022-07-08 9:00 AM	14.3	13.9	14.6	14.3

2022-07-08 10:00 AM	15.9	16.1	15.3	15.8
2022-07-08 11:00 AM	20.1	21.9	16.2	19.4
2022-07-08 12:00 PM	21.5	23.3	17.2	20.7
2022-07-08 1:00 PM	23.3	23.2	18.3	21.6
2022-07-08 2:00 PM	22.4	22.1	19.2	21.2
2022-07-08 3:00 PM	21.6	22.3	21.5	21.8
2022-07-08 4:00 PM	21.2	22.1	23.6	22.3
2022-07-08 5:00 PM	20.7	22.1	23.9	22.2
2022-07-08 6:00 PM	20.3	21.3	21.7	21.1
2022-07-08 7:00 PM	18.9	18.9	19.3	19.0
2022-07-08 8:00 PM	18.4	18.2	18.6	18.4
2022-07-08 9:00 PM	18.1	17.8	18.3	18.1
2022-07-08 10:00 PM	17.5	17.0	17.9	17.5
2022-07-08 11:00 PM	16.9	16.1	17.4	16.8
2022-07-09 12:00 AM	16.4	15.6	17.1	16.4
2022-07-09 1:00 AM	15.8	15.0	16.4	15.7
2022-07-09 2:00 AM	15.5	14.7	16.0	15.4
2022-07-09 3:00 AM	15.0	14.2	15.6	14.9
2022-07-09 4:00 AM	14.5	13.4	15.2	14.4
2022-07-09 5:00 AM	13.9	12.8	14.8	13.8
2022-07-09 6:00 AM	13.6	12.6	14.4	13.5
2022-07-09 7:00 AM	13.3	12.2	14.0	13.2
2022-07-09 8:00 AM	13.5	12.8	14.1	13.5
2022-07-09 9:00 AM	14.6	14.4	15.0	14.7
2022-07-09 10:00 AM	16.1	16.2	15.7	16.0
2022-07-09 11:00 AM	20.1	20.9	16.7	19.2
2022-07-09 12:00 PM	21.6	23.3	17.8	20.9
2022-07-09 1:00 PM	24.1	23.2	18.8	22.0
2022-07-09 2:00 PM	23.7	23.0	20.0	22.2
2022-07-09 3:00 PM	22.1	22.7	22.1	22.3
2022-07-09 4:00 PM	21.6	22.5	23.6	22.6
2022-07-09 5:00 PM	21.1	22.3	23.3	22.2
2022-07-09 6:00 PM	20.9	21.9	22.1	21.6
2022-07-09 7:00 PM	19.3	19.2	19.6	19.4
2022-07-09 8:00 PM	18.6	18.4	18.9	18.6
2022-07-09 9:00 PM	18.3	17.9	18.5	18.2
2022-07-09 10:00 PM	17.8	17.3	18.1	17.7
2022-07-09 11:00 PM	16.9	16.1	17.3	16.8
2022-07-10 12:00 AM	16.3	15.5	16.8	16.2
2022-07-10 1:00 AM	15.8	14.9	16.4	15.7
2022-07-10 2:00 AM	15.3	14.3	15.9	15.2
2022-07-10 3:00 AM	15.3	14.6	15.8	15.2
2022-07-10 4:00 AM	15.3	14.7	15.7	15.2
2022-07-10 5:00 AM	15.0	14.4	15.3	14.9
2022-07-10 6:00 AM	14.9	14.1	15.2	14.7

2022-07-10 7:00 AM	14.8	14.4	15.1	14.8
2022-07-10 8:00 AM	15.5	15.2	15.6	15.4
2022-07-10 9:00 AM	15.9	15.7	15.9	15.8
2022-07-10 10:00 AM	16.9	17.0	16.3	16.7
2022-07-10 11:00 AM	18.4	18.9	16.9	18.1
2022-07-10 12:00 PM	18.8	19.2	17.4	18.5
2022-07-10 1:00 PM	19.4	20.0	17.9	19.1
2022-07-10 2:00 PM	21.1	21.5	19.0	20.5
2022-07-10 3:00 PM	21.0	21.7	19.9	20.9
2022-07-10 4:00 PM	19.8	19.8	18.8	19.5
2022-07-10 5:00 PM	18.6	18.3	18.0	18.3
2022-07-10 6:00 PM	18.8	18.6	18.2	18.5
2022-07-10 7:00 PM	18.9	18.7	18.2	18.6
2022-07-10 8:00 PM	18.9	18.9	18.4	18.7
2022-07-10 9:00 PM	18.7	18.6	18.3	18.5
2022-07-10 10:00 PM	18.2	18.0	17.9	18.0
2022-07-10 11:00 PM	17.9	17.7	17.8	17.8
2022-07-11 12:00 AM	17.5	17.2	17.5	17.4
2022-07-11 1:00 AM	17.4	17.1	17.4	17.3
2022-07-11 2:00 AM	17.2	16.9	17.2	17.1
2022-07-11 3:00 AM	17.1	16.9	17.1	17.0
2022-07-11 4:00 AM	17.4	17.3	17.3	17.3
2022-07-11 5:00 AM	17.1	16.9	17.2	17.1
2022-07-11 6:00 AM	17.0	16.8	17.1	17.0
2022-07-11 7:00 AM	16.8	16.5	16.9	16.7
2022-07-11 8:00 AM	16.8	16.6	16.9	16.8
2022-07-11 9:00 AM	17.3	17.3	17.1	17.2
2022-07-11 10:00 AM	18.2	18.5	17.5	18.1
2022-07-11 11:00 AM	21.0	22.4	18.1	20.5
2022-07-11 12:00 PM	22.6	24.6	19.0	22.1
2022-07-11 1:00 PM	22.1	22.8	19.3	21.4
2022-07-11 2:00 PM	21.9	22.4	19.6	21.3
2022-07-11 3:00 PM	21.8	22.4	20.1	21.4
2022-07-11 4:00 PM	21.5	21.9	19.9	21.1
2022-07-11 5:00 PM	21.2	21.4	19.7	20.8
2022-07-11 6:00 PM	21.0	21.2	19.7	20.6
2022-07-11 7:00 PM	20.4	20.4	19.3	20.0
2022-07-11 8:00 PM	20.3	20.4	19.3	20.0
2022-07-11 9:00 PM	19.2	19.3	18.7	19.1
2022-07-11 10:00 PM	18.6	18.3	18.3	18.4
2022-07-11 11:00 PM	18.1	17.8	18.0	18.0
2022-07-12 12:00 AM	17.9	17.5	17.9	17.8
2022-07-12 1:00 AM	17.7	17.3	17.7	17.6
2022-07-12 2:00 AM	17.9	17.6	17.8	17.8
2022-07-12 3:00 AM	17.9	17.7	17.8	17.8

2022-07-12 4:00 AM	17.7	17.5	17.7	17.6
2022-07-12 5:00 AM	17.6	17.4	17.6	17.5
2022-07-12 6:00 AM	17.6	17.3	17.5	17.5
2022-07-12 7:00 AM	17.4	17.2	17.4	17.3
2022-07-12 8:00 AM	17.2	17.1	17.3	17.2
2022-07-12 9:00 AM	17.2	17.0	17.1	17.1
2022-07-12 10:00 AM	17.3	17.1	17.1	17.2
2022-07-12 11:00 AM	17.1	16.8	16.9	16.9
2022-07-12 12:00 PM	16.8	16.4	16.8	16.7
2022-07-12 1:00 PM	16.6	16.2	16.5	16.4
2022-07-12 2:00 PM	17.7	17.6	17.0	17.4
2022-07-12 3:00 PM	19.1	19.2	18.2	18.8
2022-07-12 4:00 PM	19.1	19.0	18.3	18.8
2022-07-12 5:00 PM	18.6	18.3	17.9	18.3
2022-07-12 6:00 PM	18.8	18.6	18.1	18.5
2022-07-12 7:00 PM	18.2	17.8	17.6	17.9
2022-07-12 8:00 PM	17.9	17.5	17.4	17.6
2022-07-12 9:00 PM	17.5	17.0	17.2	17.2
2022-07-12 10:00 PM	16.8	16.3	16.9	16.7
2022-07-12 11:00 PM	15.8	15.2	16.2	15.7
2022-07-13 12:00 AM	15.6	14.8	15.7	15.4
2022-07-13 1:00 AM	14.9	14.1	15.2	14.7
2022-07-13 2:00 AM	14.3	13.2	14.8	14.1
2022-07-13 3:00 AM	14.0	12.8	14.4	13.7
2022-07-13 4:00 AM	13.2	12.5	13.9	13.2
2022-07-13 5:00 AM	12.7	12.0	13.4	12.7
2022-07-13 6:00 AM	12.4	11.9	13.1	12.5
2022-07-13 7:00 AM	12.2	11.7	13.0	12.3
2022-07-13 8:00 AM	12.6	12.0	13.2	12.6
2022-07-13 9:00 AM	13.5	13.1	14.0	13.5
2022-07-13 10:00 AM	14.8	15.1	14.7	14.9
2022-07-13 11:00 AM	18.4	20.2	15.6	18.1
2022-07-13 12:00 PM	20.4	22.3	16.8	19.8
2022-07-13 1:00 PM	21.6	21.1	17.3	20.0
2022-07-13 2:00 PM	21.8	21.0	18.4	20.4
2022-07-13 3:00 PM	20.8	19.6	19.8	20.1
2022-07-13 4:00 PM	20.5	19.5	21.8	20.6
2022-07-13 5:00 PM	20.1	19.9	23.0	21.0
2022-07-13 6:00 PM	19.6	19.9	21.6	20.4
2022-07-13 7:00 PM	18.4	18.0	18.8	18.4
2022-07-13 8:00 PM	17.7	17.4	17.9	17.7
2022-07-13 9:00 PM	17.4	17.1	17.5	17.3
2022-07-13 10:00 PM	17.0	16.7	17.2	17.0
2022-07-13 11:00 PM	16.2	16.0	16.6	16.3
2022-07-14 12:00 AM	15.8	15.7	16.3	15.9

2022-07-14 1:00 AM	15.3	15.4	15.8	15.5
2022-07-14 2:00 AM	14.6	15.2	15.6	15.1
2022-07-14 3:00 AM	14.5	14.7	15.0	14.7
2022-07-14 4:00 AM	13.8	14.1	14.6	14.2
2022-07-14 5:00 AM	13.1	13.6	14.1	13.6
2022-07-14 6:00 AM	12.6	13.0	13.6	13.1
2022-07-14 7:00 AM	12.2	12.7	13.3	12.7
2022-07-14 8:00 AM	12.8	13.1	13.5	13.1
2022-07-14 9:00 AM	14.1	14.1	14.2	14.1
2022-07-14 10:00 AM	15.7	15.5	15.1	15.4
2022-07-14 11:00 AM	20.2	18.8	15.8	18.3
2022-07-14 12:00 PM	22.4	20.1	16.6	19.7
2022-07-14 1:00 PM	24.5	19.6	17.4	20.5
2022-07-14 2:00 PM	23.9	19.5	18.9	20.8
2022-07-14 3:00 PM	21.7	19.4	21.9	21.0
2022-07-14 4:00 PM	21.3	19.6	25.2	22.0
2022-07-14 5:00 PM	20.8	19.8	26.9	22.5
2022-07-14 6:00 PM	20.2	19.6	24.7	21.5
2022-07-14 7:00 PM	19.0	18.2	19.8	19.0
2022-07-14 8:00 PM	18.3	17.7	18.7	18.2
2022-07-14 9:00 PM	17.8	17.4	18.2	17.8
2022-07-14 10:00 PM	17.3	17.2	17.7	17.4
2022-07-14 11:00 PM	16.8	16.8	17.2	16.9
2022-07-15 12:00 AM	16.2	16.4	16.7	16.4
2022-07-15 1:00 AM	15.9	16.1	16.4	16.1
2022-07-15 2:00 AM	15.6	15.9	16.1	15.9
2022-07-15 3:00 AM	15.5	15.7	15.9	15.7
2022-07-15 4:00 AM	15.3	15.5	15.8	15.5
2022-07-15 5:00 AM	15.5	15.6	15.8	15.6
2022-07-15 6:00 AM	15.3	15.4	15.8	15.5
2022-07-15 7:00 AM	15.1	15.3	15.5	15.3
2022-07-15 8:00 AM	15.4	15.5	15.6	15.5
2022-07-15 9:00 AM	16.2	16.1	16.1	16.1
2022-07-15 10:00 AM	16.8	16.6	16.3	16.6
2022-07-15 11:00 AM	21.1	19.3	16.8	19.1
2022-07-15 12:00 PM	23.6	20.8	17.5	20.6
2022-07-15 1:00 PM	25.2	20.3	18.2	21.2
2022-07-15 2:00 PM	24.0	19.8	19.4	21.1
2022-07-15 3:00 PM	21.7	19.9	22.0	21.2
2022-07-15 4:00 PM	21.1	19.9	25.2	22.1
2022-07-15 5:00 PM	20.8	20.2	27.2	22.7
2022-07-15 6:00 PM	20.2	20.0	24.8	21.7
2022-07-15 7:00 PM	19.7	18.9	20.4	19.7
2022-07-15 8:00 PM	19.6	18.9	19.8	19.4
2022-07-15 9:00 PM	19.1	18.6	19.3	19.0

2022-07-15 10:00 PM	18.7	18.3	18.9	18.6
2022-07-15 11:00 PM	17.9	17.7	18.4	18.0
2022-07-16 12:00 AM	17.3	17.2	17.9	17.5
2022-07-16 1:00 AM	16.9	16.8	17.5	17.1
2022-07-16 2:00 AM	16.7	16.7	17.2	16.9
2022-07-16 3:00 AM	16.3	16.3	16.7	16.4
2022-07-16 4:00 AM	15.9	16.0	16.4	16.1
2022-07-16 5:00 AM	15.6	15.8	16.2	15.9
2022-07-16 6:00 AM	15.6	15.8	16.2	15.9
2022-07-16 7:00 AM	15.5	15.6	15.9	15.7
2022-07-16 8:00 AM	15.6	15.7	16.0	15.8
2022-07-16 9:00 AM	16.5	16.5	16.6	16.5
2022-07-16 10:00 AM	18.1	17.5	17.2	17.6
2022-07-16 11:00 AM	22.4	20.7	18.0	20.4
2022-07-16 12:00 PM	24.7	22.0	18.9	21.9
2022-07-16 1:00 PM	24.8	21.1	19.1	21.7
2022-07-16 2:00 PM	24.4	20.8	20.3	21.8
2022-07-16 3:00 PM	22.8	20.9	21.8	21.8
2022-07-16 4:00 PM	22.2	20.9	25.5	22.9
2022-07-16 5:00 PM	22.2	21.3	27.4	23.6
2022-07-16 6:00 PM	22.0	21.6	25.8	23.1
2022-07-16 7:00 PM	21.0	20.2	21.5	20.9
2022-07-16 8:00 PM	20.5	19.8	20.7	20.3
2022-07-16 9:00 PM	19.9	19.3	20.1	19.8
2022-07-16 10:00 PM	19.2	18.8	19.4	19.1
2022-07-16 11:00 PM	18.4	18.1	18.7	18.4
2022-07-17 12:00 AM	18.0	17.8	18.4	18.1
2022-07-17 1:00 AM	17.5	17.4	17.9	17.6
2022-07-17 2:00 AM	17.1	17.1	17.6	17.3
2022-07-17 3:00 AM	16.8	16.8	17.2	16.9
2022-07-17 4:00 AM	16.3	16.4	16.8	16.5
2022-07-17 5:00 AM	15.9	16.0	16.5	16.1
2022-07-17 6:00 AM	15.9	15.9	16.4	16.1
2022-07-17 7:00 AM	15.7	15.8	16.3	15.9
2022-07-17 8:00 AM	15.9	15.9	16.3	16.0
2022-07-17 9:00 AM	16.5	16.5	16.7	16.6
2022-07-17 10:00 AM	17.6	17.3	17.1	17.3
2022-07-17 11:00 AM	20.9	19.7	17.4	19.3
2022-07-17 12:00 PM	22.8	20.5	17.9	20.4
2022-07-17 1:00 PM	24.1	20.6	18.7	21.1
2022-07-17 2:00 PM	24.0	21.0	20.4	21.8
2022-07-17 3:00 PM	22.1	20.8	22.3	21.7
2022-07-17 4:00 PM	21.4	20.4	25.0	22.3
2022-07-17 5:00 PM	20.6	20.1	26.3	22.3
2022-07-17 6:00 PM	20.4	20.3	24.8	21.8

2022-07-17 7:00 PM	19.2	18.8	20.2	19.4
2022-07-17 8:00 PM	18.6	18.3	19.1	18.7
2022-07-17 9:00 PM	18.2	17.9	18.6	18.2
2022-07-17 10:00 PM	17.6	17.4	18.0	17.7
2022-07-17 11:00 PM	17.1	17.0	17.6	17.2
2022-07-18 12:00 AM	17.0	17.0	17.6	17.2
2022-07-18 1:00 AM	16.9	16.9	17.4	17.1
2022-07-18 2:00 AM	16.6	16.6	17.2	16.8
2022-07-18 3:00 AM	16.6	16.6	17.1	16.8
2022-07-18 4:00 AM	16.4	16.4	16.8	16.5
2022-07-18 5:00 AM	16.5	16.5	16.8	16.6
2022-07-18 6:00 AM	16.6	16.6	16.9	16.7
2022-07-18 7:00 AM	16.8	16.8	17.0	16.9
2022-07-18 8:00 AM	16.6	16.6	16.9	16.7
2022-07-18 9:00 AM	17.3	17.2	17.3	17.3
2022-07-18 10:00 AM	18.6	18.0	17.8	18.1
2022-07-18 11:00 AM	23.0	21.3	18.8	21.0
2022-07-18 12:00 PM	26.6	23.0	20.1	23.2
2022-07-18 1:00 PM	27.7	23.3	21.4	24.1
2022-07-18 2:00 PM	27.6	23.5	22.9	24.7
2022-07-18 3:00 PM	25.7	23.6	23.7	24.3
2022-07-18 4:00 PM	25.6	23.9	26.4	25.3
2022-07-18 5:00 PM	25.0	23.9	28.4	25.8
2022-07-18 6:00 PM	24.6	23.7	26.8	25.0
2022-07-18 7:00 PM	23.7	22.2	23.1	23.0
2022-07-18 8:00 PM	23.3	22.2	22.7	22.7
2022-07-18 9:00 PM	23.1	22.1	22.3	22.5
2022-07-18 10:00 PM	22.4	21.7	21.9	22.0
2022-07-18 11:00 PM	21.8	21.1	21.5	21.5
2022-07-19 12:00 AM	21.3	20.8	21.2	21.1
2022-07-19 1:00 AM	21.2	20.8	21.1	21.0
2022-07-19 2:00 AM	21.1	20.7	21.0	20.9
2022-07-19 3:00 AM	20.9	20.5	20.8	20.7
2022-07-19 4:00 AM	20.8	20.4	20.7	20.6
2022-07-19 5:00 AM	20.5	20.3	20.5	20.4
2022-07-19 6:00 AM	20.3	20.1	20.3	20.2
2022-07-19 7:00 AM	20.2	20.0	20.2	20.1
2022-07-19 8:00 AM	20.1	19.9	20.1	20.0
2022-07-19 9:00 AM	20.0	19.9	20.0	20.0
2022-07-19 10:00 AM	20.0	19.8	19.9	19.9
2022-07-19 11:00 AM	20.0	19.8	19.9	19.9
2022-07-19 12:00 PM	19.4	19.2	19.4	19.3
2022-07-19 1:00 PM	18.6	18.6	18.9	18.7
2022-07-19 2:00 PM	19.1	19.0	19.0	19.0
2022-07-19 3:00 PM	19.3	19.2	19.1	19.2

2022-07-19 4:00 PM	18.8	18.7	18.7	18.7
2022-07-19 5:00 PM	18.7	18.5	18.4	18.5
2022-07-19 6:00 PM	19.4	19.1	18.9	19.1
2022-07-19 7:00 PM	19.4	19.2	19.0	19.2
2022-07-19 8:00 PM	19.1	18.9	18.8	18.9
2022-07-19 9:00 PM	18.5	18.4	18.5	18.5
2022-07-19 10:00 PM	18.1	18.0	18.1	18.1
2022-07-19 11:00 PM	17.7	17.8	17.8	17.8
2022-07-20 12:00 AM	17.7	17.7	17.7	17.7
2022-07-20 1:00 AM	17.7	17.6	17.7	17.7
2022-07-20 2:00 AM	17.6	17.5	17.6	17.6
2022-07-20 3:00 AM	17.4	17.3	17.4	17.4
2022-07-20 4:00 AM	17.2	17.1	17.3	17.2
2022-07-20 5:00 AM	16.8	16.8	17.1	16.9
2022-07-20 6:00 AM	16.4	16.4	16.7	16.5
2022-07-20 7:00 AM	16.1	16.1	16.3	16.2
2022-07-20 8:00 AM	16.2	16.2	16.3	16.2
2022-07-20 9:00 AM	17.1	17.0	16.9	17.0
2022-07-20 10:00 AM	18.0	17.7	17.3	17.7
2022-07-20 11:00 AM	20.9	20.0	18.1	19.7
2022-07-20 12:00 PM	22.1	20.8	18.9	20.6
2022-07-20 1:00 PM	21.9	20.9	19.5	20.8
2022-07-20 2:00 PM	21.7	20.8	19.7	20.7
2022-07-20 3:00 PM	21.0	20.3	19.7	20.3
2022-07-20 4:00 PM	21.0	20.5	19.8	20.4
2022-07-20 5:00 PM	20.8	20.3	19.7	20.3
2022-07-20 6:00 PM	20.9	20.4	19.8	20.4
2022-07-20 7:00 PM	20.8	20.3	19.9	20.3
2022-07-20 8:00 PM	20.7	20.2	19.9	20.3
2022-07-20 9:00 PM	20.6	20.2	19.8	20.2
2022-07-20 10:00 PM	20.4	20.0	19.7	20.0
2022-07-20 11:00 PM	19.7	19.3	19.3	19.4
2022-07-21 12:00 AM	19.0	18.7	18.9	18.9
2022-07-21 1:00 AM	18.7	18.5	18.7	18.6
2022-07-21 2:00 AM	18.3	18.1	18.4	18.3
2022-07-21 3:00 AM	18.0	17.9	18.2	18.0
2022-07-21 4:00 AM	18.1	18.0	18.2	18.1
2022-07-21 5:00 AM	18.0	18.0	18.2	18.1
2022-07-21 6:00 AM	17.9	17.9	18.1	18.0
2022-07-21 7:00 AM	17.7	17.7	18.0	17.8
2022-07-21 8:00 AM	17.7	17.6	17.9	17.7
2022-07-21 9:00 AM	18.0	17.9	18.0	18.0
2022-07-21 10:00 AM	18.7	18.3	18.2	18.4
2022-07-21 11:00 AM	22.1	20.6	18.7	20.5
2022-07-21 12:00 PM	25.5	21.9	19.5	22.3

2022-07-21 1:00 PM	24.5	21.6	20.1	22.1
2022-07-21 2:00 PM	23.7	21.3	20.5	21.8
2022-07-21 3:00 PM	23.1	21.3	22.4	22.3
2022-07-21 4:00 PM	22.9	21.3	25.0	23.1
2022-07-21 5:00 PM	22.8	21.7	27.5	24.0
2022-07-21 6:00 PM	21.9	21.0	23.1	22.0
2022-07-21 7:00 PM	20.7	19.9	20.7	20.4
2022-07-21 8:00 PM	20.5	19.9	20.6	20.3
2022-07-21 9:00 PM	20.2	19.8	20.4	20.1
2022-07-21 10:00 PM	19.9	19.5	20.0	19.8
2022-07-21 11:00 PM	19.0	18.7	19.2	19.0
2022-07-22 12:00 AM	18.2	18.0	18.6	18.3
2022-07-22 1:00 AM	17.8	17.7	18.2	17.9
2022-07-22 2:00 AM	17.4	17.4	17.8	17.5
2022-07-22 3:00 AM	17.0	17.1	17.5	17.2
2022-07-22 4:00 AM	16.7	16.7	17.1	16.8
2022-07-22 5:00 AM	16.3	16.5	16.8	16.5
2022-07-22 6:00 AM	16.0	16.1	16.4	16.2
2022-07-22 7:00 AM	15.8	16.0	16.2	16.0
2022-07-22 8:00 AM	16.0	16.1	16.4	16.2
2022-07-22 9:00 AM	16.9	16.9	17.0	16.9
2022-07-22 10:00 AM	18.2	17.9	17.7	17.9
2022-07-22 11:00 AM	21.9	20.6	18.6	20.4
2022-07-22 12:00 PM	25.2	21.8	19.6	22.2
2022-07-22 1:00 PM	25.5	22.2	20.5	22.7
2022-07-22 2:00 PM	24.9	21.9	21.3	22.7
2022-07-22 3:00 PM	23.5	21.7	23.4	22.9
2022-07-22 4:00 PM	21.6	21.1	23.5	22.1
2022-07-22 5:00 PM	21.6	21.1	23.3	22.0
2022-07-22 6:00 PM	21.2	20.5	21.5	21.1
2022-07-22 7:00 PM	21.1	20.4	21.0	20.8
2022-07-22 8:00 PM	20.8	20.1	20.7	20.5
2022-07-22 9:00 PM	20.6	20.0	20.5	20.4
2022-07-22 10:00 PM	20.2	19.8	20.2	20.1
2022-07-22 11:00 PM	19.6	19.2	19.8	19.5
2022-07-23 12:00 AM	19.1	18.9	19.3	19.1
2022-07-23 1:00 AM	18.6	18.4	18.8	18.6
2022-07-23 2:00 AM	18.4	18.2	18.6	18.4
2022-07-23 3:00 AM	17.8	17.7	18.1	17.9
2022-07-23 4:00 AM	17.3	17.2	17.5	17.3
2022-07-23 5:00 AM	17.0	17.0	17.3	17.1
2022-07-23 6:00 AM	16.9	16.9	17.2	17.0
2022-07-23 7:00 AM	16.8	16.8	17.0	16.9
2022-07-23 8:00 AM	17.2	17.1	17.1	17.1
2022-07-23 9:00 AM	17.9	17.7	17.6	17.7

2022-07-23 10:00 AM	18.7	18.4	18.2	18.4
2022-07-23 11:00 AM	20.4	19.4	18.6	19.5
2022-07-23 12:00 PM	24.2	20.9	19.5	21.5
2022-07-23 1:00 PM	23.7	21.4	20.6	21.9
2022-07-23 2:00 PM	22.4	21.2	20.6	21.4
2022-07-23 3:00 PM	22.2	21.1	20.7	21.3
2022-07-23 4:00 PM	20.9	20.1	19.8	20.3
2022-07-23 5:00 PM	21.2	20.3	20.0	20.5
2022-07-23 6:00 PM	20.6	19.9	19.7	20.1
2022-07-23 7:00 PM	19.9	19.3	19.2	19.5
2022-07-23 8:00 PM	19.4	19.0	19.0	19.1
2022-07-23 9:00 PM	19.2	18.8	18.8	18.9
2022-07-23 10:00 PM	19.0	18.8	18.7	18.8
2022-07-23 11:00 PM	18.8	18.6	18.6	18.7
2022-07-24 12:00 AM	18.4	18.3	18.4	18.4
2022-07-24 1:00 AM	18.0	17.9	18.0	18.0
2022-07-24 2:00 AM	18.2	18.1	18.1	18.1
2022-07-24 3:00 AM	18.1	18.0	18.0	18.0
2022-07-24 4:00 AM	18.0	17.8	17.8	17.9
2022-07-24 5:00 AM	17.7	17.6	17.7	17.7
2022-07-24 6:00 AM	17.7	17.6	17.6	17.6
2022-07-24 7:00 AM	17.6	17.4	17.5	17.5
2022-07-24 8:00 AM	17.4	17.3	17.5	17.4
2022-07-24 9:00 AM	17.5	17.5	17.6	17.5
2022-07-24 10:00 AM	18.1	17.8	17.7	17.9
2022-07-24 11:00 AM	20.2	19.6	18.1	19.3
2022-07-24 12:00 PM	19.0	18.7	18.0	18.6
2022-07-24 1:00 PM	18.9	18.5	18.1	18.5
2022-07-24 2:00 PM	18.5	18.1	17.9	18.2
2022-07-24 3:00 PM	17.8	17.5	17.4	17.6
2022-07-24 4:00 PM	17.5	17.1	17.2	17.3
2022-07-24 5:00 PM	17.5	17.1	18.3	17.6
2022-07-24 6:00 PM	17.3	16.9	18.1	17.4
2022-07-24 7:00 PM	16.7	16.4	17.1	16.7
2022-07-24 8:00 PM	16.5	16.4	16.7	16.5
2022-07-24 9:00 PM	16.3	16.2	16.5	16.3
2022-07-24 10:00 PM	16.0	15.9	16.3	16.1
2022-07-24 11:00 PM	15.8	15.6	16.0	15.8
2022-07-25 12:00 AM	15.8	15.7	16.0	15.8
2022-07-25 1:00 AM	15.7	15.7	16.0	15.8
2022-07-25 2:00 AM	15.6	15.6	15.9	15.7
2022-07-25 3:00 AM	15.5	15.4	15.7	15.5
2022-07-25 4:00 AM	15.1	15.1	15.4	15.2
2022-07-25 5:00 AM	14.7	14.6	15.0	14.8
2022-07-25 6:00 AM	14.3	14.3	14.8	14.5

2022-07-25 7:00 AM	14.2	14.1	14.5	14.3
2022-07-25 8:00 AM	14.3	14.3	14.6	14.4
2022-07-25 9:00 AM	14.8	14.8	15.0	14.9
2022-07-25 10:00 AM	16.0	15.7	15.5	15.7
2022-07-25 11:00 AM	19.4	18.1	16.2	17.9
2022-07-25 12:00 PM	21.9	19.3	16.9	19.4
2022-07-25 1:00 PM	22.1	19.4	17.7	19.7
2022-07-25 2:00 PM	22.0	19.5	18.6	20.0
2022-07-25 3:00 PM	20.6	19.3	20.1	20.0
2022-07-25 4:00 PM	20.7	19.7	23.3	21.2
2022-07-25 5:00 PM	20.4	19.6	24.3	21.4
2022-07-25 6:00 PM	20.4	19.8	22.8	21.0
2022-07-25 7:00 PM	19.5	18.8	19.7	19.3
2022-07-25 8:00 PM	18.9	18.3	18.9	18.7
2022-07-25 9:00 PM	18.6	18.1	18.7	18.5
2022-07-25 10:00 PM	18.1	17.6	18.3	18.0
2022-07-25 11:00 PM	17.5	17.1	17.7	17.4
2022-07-26 12:00 AM	16.8	16.5	17.1	16.8
2022-07-26 1:00 AM	16.2	16.0	16.5	16.2
2022-07-26 2:00 AM	15.9	15.7	16.3	16.0
2022-07-26 3:00 AM	15.6	15.5	16.0	15.7
2022-07-26 4:00 AM	15.4	15.3	15.9	15.5
2022-07-26 5:00 AM	15.0	14.9	15.4	15.1
2022-07-26 6:00 AM	15.0	14.8	15.3	15.0
2022-07-26 7:00 AM	14.9	14.7	15.3	15.0
2022-07-26 8:00 AM	15.1	15.0	15.4	15.2
2022-07-26 9:00 AM	16.0	15.9	15.9	15.9
2022-07-26 10:00 AM	17.1	16.7	16.4	16.7
2022-07-26 11:00 AM	19.1	18.3	17.0	18.1
2022-07-26 12:00 PM	19.9	18.8	17.6	18.8
2022-07-26 1:00 PM	20.3	19.0	18.1	19.1
2022-07-26 2:00 PM	21.6	20.0	19.5	20.4
2022-07-26 3:00 PM	20.7	19.8	19.4	20.0
2022-07-26 4:00 PM	20.2	19.4	19.1	19.6
2022-07-26 5:00 PM	20.4	19.7	19.6	19.9
2022-07-26 6:00 PM	20.4	19.7	19.5	19.9
2022-07-26 7:00 PM	20.0	19.3	19.2	19.5
2022-07-26 8:00 PM	19.6	19.0	18.9	19.2
2022-07-26 9:00 PM	19.1	18.7	18.7	18.8
2022-07-26 10:00 PM	19.0	18.7	18.6	18.8
2022-07-26 11:00 PM	18.5	18.2	18.3	18.3
2022-07-27 12:00 AM	18.1	17.9	18.0	18.0
2022-07-27 1:00 AM	18.3	18.0	18.1	18.1
2022-07-27 2:00 AM	17.9	17.7	17.9	17.8
2022-07-27 3:00 AM	17.8	17.6	17.8	17.7

2022-07-27 4:00 AM	17.3	17.2	17.4	17.3
2022-07-27 5:00 AM	16.8	16.6	16.9	16.8
2022-07-27 6:00 AM	16.5	16.3	16.7	16.5
2022-07-27 7:00 AM	16.3	16.1	16.5	16.3
2022-07-27 8:00 AM	16.4	16.2	16.5	16.4
2022-07-27 9:00 AM	16.9	16.8	16.9	16.9
2022-07-27 10:00 AM	17.8	17.5	17.2	17.5
2022-07-27 11:00 AM	20.3	19.5	17.7	19.2
2022-07-27 12:00 PM	23.6	20.8	18.6	21.0
2022-07-27 1:00 PM	24.1	21.1	19.5	21.6
2022-07-27 2:00 PM	23.5	21.1	20.3	21.6
2022-07-27 3:00 PM	22.0	20.7	20.6	21.1
2022-07-27 4:00 PM	22.0	20.9	24.2	22.4
2022-07-27 5:00 PM	21.9	21.1	24.2	22.4
2022-07-27 6:00 PM	21.3	20.6	21.8	21.2
2022-07-27 7:00 PM	19.8	19.1	19.7	19.5
2022-07-27 8:00 PM	19.2	18.6	19.0	18.9
2022-07-27 9:00 PM	17.7	17.4	18.0	17.7
2022-07-27 10:00 PM	17.7	17.4	17.7	17.6
2022-07-27 11:00 PM	17.6	17.2	17.5	17.4
2022-07-28 12:00 AM	17.1	16.7	17.1	17.0
2022-07-28 1:00 AM	16.6	16.2	16.7	16.5
2022-07-28 2:00 AM	16.6	16.3	16.8	16.6
2022-07-28 3:00 AM	16.6	16.3	16.6	16.5
2022-07-28 4:00 AM	16.6	16.3	16.6	16.5
2022-07-28 5:00 AM	16.5	16.3	16.5	16.4
2022-07-28 6:00 AM	16.3	16.2	16.4	16.3
2022-07-28 7:00 AM	16.0	15.8	16.1	16.0
2022-07-28 8:00 AM	15.9	15.8	16.1	15.9
2022-07-28 9:00 AM	16.1	16.0	16.3	16.1
2022-07-28 10:00 AM	16.4	16.3	16.2	16.3
2022-07-28 11:00 AM	18.3	17.8	16.5	17.5
2022-07-28 12:00 PM	18.4	17.9	16.8	17.7
2022-07-28 1:00 PM	19.3	18.3	17.4	18.3
2022-07-28 2:00 PM	19.9	18.7	18.0	18.9
2022-07-28 3:00 PM	18.9	18.4	18.3	18.5
2022-07-28 4:00 PM	18.6	18.1	18.1	18.3
2022-07-28 5:00 PM	19.3	18.9	20.3	19.5
2022-07-28 6:00 PM	18.8	18.5	19.3	18.9
2022-07-28 7:00 PM	18.4	18.1	18.3	18.3
2022-07-28 8:00 PM	18.0	17.8	17.9	17.9
2022-07-28 9:00 PM	17.9	17.6	17.8	17.8
2022-07-28 10:00 PM	17.3	17.0	17.4	17.2
2022-07-28 11:00 PM	17.2	16.9	17.2	17.1
2022-07-29 12:00 AM	16.8	16.5	16.8	16.7

2022-07-29 1:00 AM	16.4	16.1	16.6	16.4
2022-07-29 2:00 AM	16.1	15.9	16.3	16.1
2022-07-29 3:00 AM	16.2	16.0	16.4	16.2
2022-07-29 4:00 AM	16.3	16.1	16.4	16.3
2022-07-29 5:00 AM	16.2	16.1	16.3	16.2
2022-07-29 6:00 AM	16.1	15.9	16.2	16.1
2022-07-29 7:00 AM	16.2	16.0	16.2	16.1
2022-07-29 8:00 AM	16.2	16.1	16.3	16.2
2022-07-29 9:00 AM	16.2	16.1	16.2	16.2
2022-07-29 10:00 AM	16.9	16.7	16.5	16.7
2022-07-29 11:00 AM	19.4	18.2	17.0	18.2
2022-07-29 12:00 PM	19.9	18.8	17.6	18.8
2022-07-29 1:00 PM	20.1	19.0	18.1	19.1
2022-07-29 2:00 PM	19.9	19.0	18.4	19.1
2022-07-29 3:00 PM	19.8	18.9	18.4	19.0
2022-07-29 4:00 PM	19.7	19.0	18.4	19.0
2022-07-29 5:00 PM	19.4	18.9	18.3	18.9
2022-07-29 6:00 PM	19.2	18.8	18.1	18.7
2022-07-29 7:00 PM	19.1	18.7	18.2	18.7
2022-07-29 8:00 PM	18.0	17.7	17.6	17.8
2022-07-29 9:00 PM	17.8	17.5	17.6	17.6
2022-07-29 10:00 PM	17.3	17.0	17.2	17.2
2022-07-29 11:00 PM	17.2	16.9	17.1	17.1
2022-07-30 12:00 AM	17.0	16.7	16.8	16.8
2022-07-30 1:00 AM	16.5	16.1	16.4	16.3
2022-07-30 2:00 AM	16.1	15.7	16.0	15.9
2022-07-30 3:00 AM	15.7	15.4	15.6	15.6
2022-07-30 4:00 AM	15.3	15.0	15.3	15.2
2022-07-30 5:00 AM	15.1	14.7	15.0	14.9
2022-07-30 6:00 AM	14.8	14.5	15.0	14.8
2022-07-30 7:00 AM	14.8	14.5	15.0	14.8
2022-07-30 8:00 AM	14.9	14.7	15.1	14.9
2022-07-30 9:00 AM	15.5	15.4	15.6	15.5
2022-07-30 10:00 AM	16.7	16.5	16.1	16.4
2022-07-30 11:00 AM	19.8	18.4	17.1	18.4
2022-07-30 12:00 PM	23.1	20.3	18.5	20.6
2022-07-30 1:00 PM	23.7	20.7	19.6	21.3
2022-07-30 2:00 PM	22.3	20.4	19.9	20.9
2022-07-30 3:00 PM	21.9	20.5	21.0	21.1
2022-07-30 4:00 PM	21.9	20.9	23.6	22.1
2022-07-30 5:00 PM	22.1	21.5	26.2	23.3
2022-07-30 6:00 PM	21.8	21.4	24.9	22.7
2022-07-30 7:00 PM	21.0	20.4	21.7	21.0
2022-07-30 8:00 PM	20.4	19.9	20.8	20.4
2022-07-30 9:00 PM	20.0	19.5	20.2	19.9

2022-07-30 10:00 PM	19.5	19.0	19.7	19.4
2022-07-30 11:00 PM	18.8	18.4	19.2	18.8
2022-07-31 12:00 AM	18.3	17.9	18.6	18.3
2022-07-31 1:00 AM	17.9	17.6	18.2	17.9
2022-07-31 2:00 AM	17.8	17.4	18.0	17.7
2022-07-31 3:00 AM	17.5	17.2	17.7	17.5
2022-07-31 4:00 AM	17.3	17.0	17.5	17.3
2022-07-31 5:00 AM	17.1	16.8	17.2	17.0
2022-07-31 6:00 AM	17.2	17.0	17.3	17.2
2022-07-31 7:00 AM	16.9	16.6	17.0	16.8
2022-07-31 8:00 AM	17.0	16.7	17.0	16.9
2022-07-31 9:00 AM	17.8	17.5	17.5	17.6
2022-07-31 10:00 AM	18.4	18.1	17.9	18.1
2022-07-31 11:00 AM	20.4	19.4	18.3	19.4
2022-07-31 12:00 PM	21.7	20.3	19.0	20.3
2022-07-31 1:00 PM	23.1	20.8	19.7	21.2
2022-07-31 2:00 PM	24.0	21.6	20.9	22.2
2022-07-31 3:00 PM	21.6	20.4	20.0	20.7
2022-07-31 4:00 PM	20.9	20.0	19.8	20.2
2022-07-31 5:00 PM	21.7	20.9	21.2	21.3
2022-07-31 6:00 PM	21.0	20.3	20.3	20.5
2022-07-31 7:00 PM	20.5	19.9	19.8	20.1
2022-07-31 8:00 PM	20.1	19.6	19.5	19.7
2022-07-31 9:00 PM	19.7	19.1	19.2	19.3
2022-07-31 10:00 PM	19.0	18.4	18.5	18.6
2022-07-31 11:00 PM	18.8	18.3	18.3	18.5
2022-08-01 12:00 AM	18.4	18.0	18.1	18.2
2022-08-01 1:00 AM	18.0	17.7	17.9	17.9
2022-08-01 2:00 AM	17.9	17.7	17.8	17.8
2022-08-01 3:00 AM	17.9	17.7	17.8	17.8
2022-08-01 4:00 AM	17.9	17.7	17.7	17.8
2022-08-01 5:00 AM	17.9	17.7	17.8	17.8
2022-08-01 6:00 AM	17.7	17.6	17.7	17.7
2022-08-01 7:00 AM	17.8	17.8	17.8	17.8
2022-08-01 8:00 AM	17.9	17.8	17.8	17.8
2022-08-01 9:00 AM	17.7	17.7	17.6	17.7
2022-08-01 10:00 AM	17.7	17.7	17.5	17.6
2022-08-01 11:00 AM	18.7	18.3	17.5	18.2
2022-08-01 12:00 PM	18.5	18.2	17.6	18.1
2022-08-01 1:00 PM	19.1	18.5	17.9	18.5
2022-08-01 2:00 PM	18.9	18.3	18.0	18.4
2022-08-01 3:00 PM	18.9	18.4	18.3	18.5
2022-08-01 4:00 PM	18.9	18.4	18.5	18.6
2022-08-01 5:00 PM	19.4	18.9	20.7	19.7
2022-08-01 6:00 PM	19.2	18.8	20.4	19.5

2022-08-01 7:00 PM	18.7	18.2	18.8	18.6
2022-08-01 8:00 PM	18.2	17.8	18.3	18.1
2022-08-01 9:00 PM	17.9	17.5	18.0	17.8
2022-08-01 10:00 PM	17.3	17.1	17.6	17.3
2022-08-01 11:00 PM	16.9	16.8	17.2	17.0
2022-08-02 12:00 AM	16.5	16.4	16.8	16.6
2022-08-02 1:00 AM	16.1	16.0	16.5	16.2
2022-08-02 2:00 AM	15.8	15.6	16.0	15.8
2022-08-02 3:00 AM	15.8	15.7	16.0	15.8
2022-08-02 4:00 AM	15.4	15.4	15.6	15.5
2022-08-02 5:00 AM	15.2	15.1	15.4	15.2
2022-08-02 6:00 AM	15.0	15.0	15.3	15.1
2022-08-02 7:00 AM	15.2	15.1	15.3	15.2
2022-08-02 8:00 AM	15.4	15.3	15.5	15.4
2022-08-02 9:00 AM	16.1	16.0	15.9	16.0
2022-08-02 10:00 AM	17.3	17.0	16.6	17.0
2022-08-02 11:00 AM	19.9	18.5	17.1	18.5
2022-08-02 12:00 PM	22.3	19.7	17.9	20.0
2022-08-02 1:00 PM	23.2	20.2	18.9	20.8
2022-08-02 2:00 PM	23.1	20.6	20.4	21.4
2022-08-02 3:00 PM	22.1	20.5	21.8	21.5
2022-08-02 4:00 PM	21.9	20.9	23.7	22.2
2022-08-02 5:00 PM	20.9	20.6	23.5	21.7
2022-08-02 6:00 PM	20.3	20.0	21.9	20.7
2022-08-02 7:00 PM	19.7	19.3	19.6	19.5
2022-08-02 8:00 PM	19.1	18.6	18.9	18.9
2022-08-02 9:00 PM	18.7	18.4	18.5	18.5
2022-08-02 10:00 PM	18.5	18.2	18.3	18.3
2022-08-02 11:00 PM	18.1	17.9	18.0	18.0
2022-08-03 12:00 AM	17.9	17.8	17.8	17.8
2022-08-03 1:00 AM	17.9	17.6	17.7	17.7
2022-08-03 2:00 AM	17.8	17.6	17.7	17.7
2022-08-03 3:00 AM	17.8	17.6	17.7	17.7
2022-08-03 4:00 AM	17.8	17.6	17.7	17.7
2022-08-03 5:00 AM	17.7	17.5	17.6	17.6
2022-08-03 6:00 AM	17.3	17.1	17.4	17.3
2022-08-03 7:00 AM	17.0	16.9	17.1	17.0
2022-08-03 8:00 AM	17.0	16.8	17.1	17.0
2022-08-03 9:00 AM	17.4	17.3	17.5	17.4
2022-08-03 10:00 AM	18.5	18.3	18.1	18.3
2022-08-03 11:00 AM	21.0	19.9	19.1	20.0
2022-08-03 12:00 PM	21.8	20.7	19.7	20.7
2022-08-03 1:00 PM	20.9	20.2	19.6	20.2
2022-08-03 2:00 PM	21.8	20.8	20.4	21.0
2022-08-03 3:00 PM	21.4	20.7	21.5	21.2

2022-08-03 4:00 PM	21.3	20.7	23.8	21.9
2022-08-03 5:00 PM	21.1	20.5	23.5	21.7
2022-08-03 6:00 PM	20.8	20.3	21.3	20.8
2022-08-03 7:00 PM	20.0	19.6	20.3	20.0
2022-08-03 8:00 PM	19.8	19.4	20.0	19.7
2022-08-03 9:00 PM	19.4	19.1	19.6	19.4
2022-08-03 10:00 PM	18.8	18.5	19.1	18.8
2022-08-03 11:00 PM	18.4	18.2	18.7	18.4
2022-08-04 12:00 AM	18.5	18.1	18.6	18.4
2022-08-04 1:00 AM	18.4	17.9	18.4	18.2
2022-08-04 2:00 AM	17.8	17.4	17.8	17.7
2022-08-04 3:00 AM	17.4	17.0	17.4	17.3
2022-08-04 4:00 AM	16.7	16.2	16.7	16.5
2022-08-04 5:00 AM	16.3	15.8	16.2	16.1
2022-08-04 6:00 AM	16.0	15.4	15.6	15.7
2022-08-04 7:00 AM	15.4	15.1	15.4	15.3
2022-08-04 8:00 AM	15.5	15.1	15.3	15.3
2022-08-04 9:00 AM	15.9	15.7	15.9	15.8
2022-08-04 10:00 AM	17.1	17.0	16.7	16.9
2022-08-04 11:00 AM	19.4	18.4	17.5	18.4
2022-08-04 12:00 PM	21.6	19.7	18.2	19.8
2022-08-04 1:00 PM	22.2	20.2	19.1	20.5
2022-08-04 2:00 PM	22.6	20.6	20.3	21.2
2022-08-04 3:00 PM	21.5	20.4	21.6	21.2
2022-08-04 4:00 PM	21.3	20.6	24.2	22.0
2022-08-04 5:00 PM	20.9	20.6	25.2	22.2
2022-08-04 6:00 PM	20.5	20.3	24.6	21.8
2022-08-04 7:00 PM	19.8	19.4	20.7	20.0
2022-08-04 8:00 PM	19.4	19.0	19.8	19.4
2022-08-04 9:00 PM	19.1	18.6	19.3	19.0
2022-08-04 10:00 PM	18.6	18.1	18.8	18.5
2022-08-04 11:00 PM	18.2	17.7	18.3	18.1
2022-08-05 12:00 AM	17.7	17.3	17.9	17.6
2022-08-05 1:00 AM	17.3	16.9	17.4	17.2
2022-08-05 2:00 AM	16.9	16.5	17.1	16.8
2022-08-05 3:00 AM	16.6	16.2	16.8	16.5
2022-08-05 4:00 AM	16.3	16.0	16.6	16.3
2022-08-05 5:00 AM	16.3	16.0	16.4	16.2
2022-08-05 6:00 AM	16.1	15.8	16.4	16.1
2022-08-05 7:00 AM	15.9	15.6	16.1	15.9
2022-08-05 8:00 AM	16.2	16.1	16.4	16.2
2022-08-05 9:00 AM	16.6	16.6	16.7	16.6
2022-08-05 10:00 AM	17.3	17.2	17.0	17.2
2022-08-05 11:00 AM	19.3	18.3	17.4	18.3
2022-08-05 12:00 PM	21.9	19.7	18.3	20.0

2022-08-05 1:00 PM	23.3	20.7	19.9	21.3
2022-08-05 2:00 PM	23.4	21.2	21.3	22.0
2022-08-05 3:00 PM	22.1	21.0	21.7	21.6
2022-08-05 4:00 PM	22.1	21.3	25.5	23.0
2022-08-05 5:00 PM	21.9	21.3	25.0	22.7
2022-08-05 6:00 PM	21.1	20.4	21.3	20.9
2022-08-05 7:00 PM	20.3	19.8	20.2	20.1
2022-08-05 8:00 PM	20.1	19.7	20.0	19.9
2022-08-05 9:00 PM	19.9	19.4	19.7	19.7
2022-08-05 10:00 PM	19.4	19.1	19.2	19.2
2022-08-05 11:00 PM	19.1	18.9	19.0	19.0
2022-08-06 12:00 AM	18.6	18.4	18.4	18.5
2022-08-06 1:00 AM	18.5	18.4	18.4	18.4
2022-08-06 2:00 AM	18.7	18.5	18.6	18.6
2022-08-06 3:00 AM	18.7	18.5	18.6	18.6
2022-08-06 4:00 AM	18.6	18.5	18.6	18.6
2022-08-06 5:00 AM	18.7	18.6	18.6	18.6
2022-08-06 6:00 AM	18.8	18.7	18.8	18.8
2022-08-06 7:00 AM	18.7	18.6	18.7	18.7
2022-08-06 8:00 AM	18.8	18.7	18.7	18.7
2022-08-06 9:00 AM	19.2	19.0	19.0	19.1
2022-08-06 10:00 AM	20.1	19.7	19.6	19.8
2022-08-06 11:00 AM	20.9	20.3	20.0	20.4
2022-08-06 12:00 PM	21.6	20.9	20.4	21.0
2022-08-06 1:00 PM	21.8	21.2	20.7	21.2
2022-08-06 2:00 PM	21.9	21.3	20.9	21.4
2022-08-06 3:00 PM	22.0	21.5	22.3	21.9
2022-08-06 4:00 PM	22.0	21.5	23.6	22.4
2022-08-06 5:00 PM	21.3	21.0	23.9	22.1
2022-08-06 6:00 PM	21.8	21.4	22.6	21.9
2022-08-06 7:00 PM	21.4	21.0	21.6	21.3
2022-08-06 8:00 PM	20.5	20.0	20.8	20.4
2022-08-06 9:00 PM	20.1	19.8	20.3	20.1
2022-08-06 10:00 PM	19.7	19.4	19.9	19.7
2022-08-06 11:00 PM	19.0	18.8	19.3	19.0
2022-08-07 12:00 AM	18.3	18.0	18.5	18.3
2022-08-07 1:00 AM	18.0	17.7	18.2	18.0
2022-08-07 2:00 AM	17.6	17.5	17.9	17.7
2022-08-07 3:00 AM	17.5	17.3	17.7	17.5
2022-08-07 4:00 AM	17.3	17.1	17.5	17.3
2022-08-07 5:00 AM	17.1	16.9	17.2	17.1
2022-08-07 6:00 AM	16.7	16.7	17.0	16.8
2022-08-07 7:00 AM	16.5	16.5	16.8	16.6
2022-08-07 8:00 AM	16.4	16.4	16.7	16.5
2022-08-07 9:00 AM	16.6	16.6	16.7	16.6

2022-08-07 10:00 AM	17.1	17.0	16.9	17.0
2022-08-07 11:00 AM	18.7	18.0	17.3	18.0
2022-08-07 12:00 PM	20.5	18.7	17.8	19.0
2022-08-07 1:00 PM	21.8	19.2	18.6	19.9
2022-08-07 2:00 PM	21.6	19.8	19.6	20.3
2022-08-07 3:00 PM	20.7	19.8	21.7	20.7
2022-08-07 4:00 PM	19.6	19.3	24.1	21.0
2022-08-07 5:00 PM	19.0	18.8	22.1	20.0
2022-08-07 6:00 PM	18.8	18.3	19.2	18.8
2022-08-07 7:00 PM	18.5	17.9	18.2	18.2
2022-08-07 8:00 PM	18.2	17.5	17.7	17.8
2022-08-07 9:00 PM	17.7	17.1	17.4	17.4
2022-08-07 10:00 PM	17.3	16.8	17.2	17.1
2022-08-07 11:00 PM	17.1	16.7	16.9	16.9
2022-08-08 12:00 AM	17.0	16.5	16.7	16.7
2022-08-08 1:00 AM	16.7	16.4	16.6	16.6
2022-08-08 2:00 AM	16.6	16.1	16.4	16.4
2022-08-08 3:00 AM	16.3	15.8	15.9	16.0
2022-08-08 4:00 AM	16.2	15.7	15.9	15.9
2022-08-08 5:00 AM	16.1	15.7	15.8	15.9
2022-08-08 6:00 AM	16.0	15.7	15.8	15.8
2022-08-08 7:00 AM	15.9	15.5	15.7	15.7
2022-08-08 8:00 AM	15.9	15.6	15.7	15.7
2022-08-08 9:00 AM	16.1	15.9	15.9	16.0
2022-08-08 10:00 AM	16.8	16.4	16.3	16.5
2022-08-08 11:00 AM	18.9	17.7	16.8	17.8
2022-08-08 12:00 PM	21.1	18.8	17.6	19.2
2022-08-08 1:00 PM	22.0	19.2	18.6	19.9
2022-08-08 2:00 PM	22.2	19.6	19.8	20.5
2022-08-08 3:00 PM	21.2	19.6	22.0	20.9
2022-08-08 4:00 PM	20.8	19.8	22.6	21.1
2022-08-08 5:00 PM	20.8	20.1	24.0	21.6
2022-08-08 6:00 PM	20.4	19.7	21.9	20.7
2022-08-08 7:00 PM	20.0	19.4	20.3	19.9
2022-08-08 8:00 PM	19.2	18.8	19.5	19.2
2022-08-08 9:00 PM	18.8	18.5	19.1	18.8
2022-08-08 10:00 PM	18.3	18.1	18.6	18.3
2022-08-08 11:00 PM	17.7	17.6	18.1	17.8
2022-08-09 12:00 AM	17.1	17.1	17.5	17.2
2022-08-09 1:00 AM	16.7	16.8	17.2	16.9
2022-08-09 2:00 AM	16.3	16.5	16.8	16.5
2022-08-09 3:00 AM	16.0	16.2	16.5	16.2
2022-08-09 4:00 AM	15.6	15.9	16.1	15.9
2022-08-09 5:00 AM	15.4	15.6	15.9	15.6
2022-08-09 6:00 AM	15.1	15.4	15.6	15.4

2022-08-09 7:00 AM	14.9	15.2	15.4	15.2
2022-08-09 8:00 AM	14.8	15.2	15.5	15.2
2022-08-09 9:00 AM	15.5	15.7	15.9	15.7
2022-08-09 10:00 AM	17.5	17.4	16.6	17.2
2022-08-09 11:00 AM	20.3	18.8	17.3	18.8
2022-08-09 12:00 PM	22.3	19.8	18.3	20.1
2022-08-09 1:00 PM	22.5	20.0	19.5	20.7
2022-08-09 2:00 PM	22.3	20.1	20.8	21.1
2022-08-09 3:00 PM	21.5	20.0	23.4	21.6
2022-08-09 4:00 PM	21.5	20.4	25.0	22.3
2022-08-09 5:00 PM	21.7	20.9	25.2	22.6
2022-08-09 6:00 PM	21.7	20.9	24.7	22.4
2022-08-09 7:00 PM	21.2	20.4	22.1	21.2
2022-08-09 8:00 PM	21.1	20.4	21.6	21.0
2022-08-09 9:00 PM	20.9	20.3	21.3	20.8
2022-08-09 10:00 PM	20.5	20.1	20.9	20.5
2022-08-09 11:00 PM	20.3	19.9	20.7	20.3
2022-08-10 12:00 AM	19.9	19.6	20.4	20.0
2022-08-10 1:00 AM	19.3	19.2	19.8	19.4
2022-08-10 2:00 AM	18.9	18.8	19.3	19.0
2022-08-10 3:00 AM	18.3	18.3	18.8	18.5
2022-08-10 4:00 AM	17.5	17.7	18.1	17.8
2022-08-10 5:00 AM	17.1	17.2	17.7	17.3
2022-08-10 6:00 AM	17.1	17.3	17.7	17.4
2022-08-10 7:00 AM	16.8	17.1	17.5	17.1
2022-08-10 8:00 AM	16.7	16.9	17.2	16.9
2022-08-10 9:00 AM	17.0	17.1	17.4	17.2
2022-08-10 10:00 AM	17.9	17.8	17.7	17.8
2022-08-10 11:00 AM	21.0	19.4	18.2	19.5
2022-08-10 12:00 PM	22.4	20.1	18.9	20.5
2022-08-10 1:00 PM	21.9	19.6	19.0	20.2
2022-08-10 2:00 PM	21.4	19.0	19.0	19.8
2022-08-10 3:00 PM	21.0	19.1	21.8	20.6
2022-08-10 4:00 PM	20.2	18.7	21.5	20.1
2022-08-10 5:00 PM	20.5	19.3	22.5	20.8
2022-08-10 6:00 PM	20.1	18.9	21.5	20.2
2022-08-10 7:00 PM	19.3	18.4	19.5	19.1
2022-08-10 8:00 PM	19.1	18.4	19.2	18.9
2022-08-10 9:00 PM	18.8	18.3	18.9	18.7
2022-08-10 10:00 PM	18.3	18.0	18.6	18.3
2022-08-10 11:00 PM	17.8	17.6	18.1	17.8
2022-08-11 12:00 AM	17.3	17.2	17.5	17.3
2022-08-11 1:00 AM	16.7	16.9	17.0	16.9
2022-08-11 2:00 AM	16.2	16.5	16.4	16.4
2022-08-11 3:00 AM	16.1	16.2	16.0	16.1

2022-08-11 4:00 AM	15.6	15.8	15.8	15.7
2022-08-11 5:00 AM	15.3	15.5	15.5	15.4
2022-08-11 6:00 AM	14.8	15.2	15.2	15.1
2022-08-11 7:00 AM	14.2	14.7	14.6	14.5
2022-08-11 8:00 AM	13.8	14.4	14.4	14.2
2022-08-11 9:00 AM	14.4	14.8	14.9	14.7
2022-08-11 10:00 AM	15.9	16.0	15.7	15.9
2022-08-11 11:00 AM	19.2	17.7	16.5	17.8
2022-08-11 12:00 PM	21.6	19.0	17.4	19.3
2022-08-11 1:00 PM	21.6	19.1	18.5	19.7
2022-08-11 2:00 PM	21.8	19.6	20.0	20.5
2022-08-11 3:00 PM	20.7	19.3	22.8	20.9
2022-08-11 4:00 PM	20.3	19.6	24.3	21.4
2022-08-11 5:00 PM	20.2	19.8	24.8	21.6
2022-08-11 6:00 PM	20.0	19.6	24.1	21.2
2022-08-11 7:00 PM	19.3	18.9	20.4	19.5
2022-08-11 8:00 PM	18.8	18.4	19.4	18.9
2022-08-11 9:00 PM	18.3	18.0	18.8	18.4
2022-08-11 10:00 PM	17.5	17.4	18.2	17.7
2022-08-11 11:00 PM	17.0	17.0	17.6	17.2
2022-08-12 12:00 AM	16.5	16.4	17.0	16.6
2022-08-12 1:00 AM	16.0	15.9	16.5	16.1
2022-08-12 2:00 AM	15.6	15.5	16.0	15.7
2022-08-12 3:00 AM	15.2	15.0	15.6	15.3
2022-08-12 4:00 AM	14.9	14.8	15.0	14.9
2022-08-12 5:00 AM	14.4	14.5	14.5	14.5
2022-08-12 6:00 AM	13.9	14.1	14.0	14.0
2022-08-12 7:00 AM	13.5	13.8	13.8	13.7
2022-08-12 8:00 AM	13.6	13.6	13.6	13.6
2022-08-12 9:00 AM	13.5	13.8	13.9	13.7
2022-08-12 10:00 AM	15.3	15.3	15.2	15.3
2022-08-12 11:00 AM	17.5	16.9	16.1	16.8
2022-08-12 12:00 PM	18.6	17.6	16.8	17.7
2022-08-12 1:00 PM	19.3	17.8	17.4	18.2
2022-08-12 2:00 PM	19.7	18.1	18.4	18.7
2022-08-12 3:00 PM	18.9	18.1	20.5	19.2
2022-08-12 4:00 PM	18.7	18.2	22.7	19.9
2022-08-12 5:00 PM	18.7	18.5	22.5	19.9
2022-08-12 6:00 PM	18.8	18.6	21.5	19.6
2022-08-12 7:00 PM	18.2	18.0	19.1	18.4
2022-08-12 8:00 PM	17.7	17.5	18.2	17.8
2022-08-12 9:00 PM	17.3	17.1	17.8	17.4
2022-08-12 10:00 PM	16.9	16.8	17.4	17.0
2022-08-12 11:00 PM	16.5	16.4	16.9	16.6
2022-08-13 12:00 AM	16.2	16.0	16.6	16.3

2022-08-13 1:00 AM	16.0	15.7	16.2	16.0
2022-08-13 2:00 AM	15.7	15.5	15.9	15.7
2022-08-13 3:00 AM	15.6	15.5	15.9	15.7
2022-08-13 4:00 AM	15.9	15.8	16.1	15.9
2022-08-13 5:00 AM	16.0	16.0	16.2	16.1
2022-08-13 6:00 AM	16.1	16.1	16.3	16.2
2022-08-13 7:00 AM	16.1	16.2	16.3	16.2
2022-08-13 8:00 AM	16.2	16.2	16.4	16.3
2022-08-13 9:00 AM	16.4	16.5	16.5	16.5
2022-08-13 10:00 AM	16.8	16.8	16.8	16.8
2022-08-13 11:00 AM	17.2	17.1	17.0	17.1
2022-08-13 12:00 PM	18.5	18.1	17.8	18.1
2022-08-13 1:00 PM	20.5	19.0	19.0	19.5
2022-08-13 2:00 PM	21.1	19.4	20.4	20.3
2022-08-13 3:00 PM	20.6	19.5	23.9	21.3
2022-08-13 4:00 PM	20.0	19.3	24.7	21.3
2022-08-13 5:00 PM	19.8	19.5	24.2	21.2
2022-08-13 6:00 PM	19.5	19.3	23.6	20.8
2022-08-13 7:00 PM	18.6	18.4	20.0	19.0
2022-08-13 8:00 PM	18.0	17.8	18.8	18.2
2022-08-13 9:00 PM	17.4	17.4	18.2	17.7
2022-08-13 10:00 PM	17.4	17.3	18.0	17.6
2022-08-13 11:00 PM	17.5	17.4	18.0	17.6
2022-08-14 12:00 AM	17.2	17.2	17.7	17.4
2022-08-14 1:00 AM	17.0	17.0	17.5	17.2
2022-08-14 2:00 AM	17.2	17.1	17.4	17.2
2022-08-14 3:00 AM	17.1	17.0	17.3	17.1
2022-08-14 4:00 AM	17.1	16.9	17.2	17.1
2022-08-14 5:00 AM	17.0	16.9	17.1	17.0
2022-08-14 6:00 AM	17.0	16.9	17.0	17.0
2022-08-14 7:00 AM	16.9	16.8	17.0	16.9
2022-08-14 8:00 AM	17.0	16.9	17.0	17.0
2022-08-14 9:00 AM	17.1	17.0	17.1	17.1
2022-08-14 10:00 AM	17.2	17.2	17.2	17.2
2022-08-14 11:00 AM	17.5	17.4	17.3	17.4
2022-08-14 12:00 PM	17.5	17.4	17.4	17.4
2022-08-14 1:00 PM	18.3	18.0	17.9	18.1
2022-08-14 2:00 PM	19.2	18.5	18.8	18.8
2022-08-14 3:00 PM	19.9	19.1	20.4	19.8
2022-08-14 4:00 PM	19.8	19.1	19.7	19.5
2022-08-14 5:00 PM	19.6	19.1	20.5	19.7
2022-08-14 6:00 PM	19.1	18.8	21.0	19.6
2022-08-14 7:00 PM	18.6	18.3	19.1	18.7
2022-08-14 8:00 PM	18.3	18.1	18.5	18.3
2022-08-14 9:00 PM	17.8	17.7	17.9	17.8

2022-08-14 10:00 PM	17.1	17.1	17.5	17.2
2022-08-14 11:00 PM	16.8	16.8	17.2	16.9
2022-08-15 12:00 AM	16.6	16.7	17.0	16.8
2022-08-15 1:00 AM	16.9	16.8	17.1	16.9
2022-08-15 2:00 AM	17.0	16.9	17.1	17.0
2022-08-15 3:00 AM	16.8	16.8	17.0	16.9
2022-08-15 4:00 AM	17.0	16.9	17.1	17.0
2022-08-15 5:00 AM	16.9	16.9	17.0	16.9
2022-08-15 6:00 AM	16.8	16.8	16.9	16.8
2022-08-15 7:00 AM	16.8	16.8	16.9	16.8
2022-08-15 8:00 AM	16.8	16.8	16.9	16.8
2022-08-15 9:00 AM	17.1	17.0	17.0	17.0
2022-08-15 10:00 AM	17.6	17.4	17.4	17.5
2022-08-15 11:00 AM	18.7	18.1	17.9	18.2
2022-08-15 12:00 PM	20.6	19.2	18.6	19.5
2022-08-15 1:00 PM	21.7	19.7	19.7	20.4
2022-08-15 2:00 PM	21.8	20.0	20.8	20.9
2022-08-15 3:00 PM	21.1	20.1	23.2	21.5
2022-08-15 4:00 PM	20.4	20.0	23.9	21.4
2022-08-15 5:00 PM	20.2	20.0	23.6	21.3
2022-08-15 6:00 PM	20.1	19.8	23.1	21.0
2022-08-15 7:00 PM	19.3	19.0	20.3	19.5
2022-08-15 8:00 PM	18.9	18.6	19.5	19.0
2022-08-15 9:00 PM	18.4	18.2	19.0	18.5
2022-08-15 10:00 PM	18.0	17.8	18.6	18.1
2022-08-15 11:00 PM	17.5	17.2	18.1	17.6
2022-08-16 12:00 AM	17.1	16.9	17.8	17.3
2022-08-16 1:00 AM	16.8	16.5	17.4	16.9
2022-08-16 2:00 AM	16.6	16.1	16.9	16.5
2022-08-16 3:00 AM	16.1	15.6	16.5	16.1
2022-08-16 4:00 AM	15.7	15.3	16.0	15.7
2022-08-16 5:00 AM	15.4	15.1	15.7	15.4
2022-08-16 6:00 AM	15.4	15.0	15.4	15.3
2022-08-16 7:00 AM	15.1	14.8	15.1	15.0
2022-08-16 8:00 AM	15.1	14.7	15.2	15.0
2022-08-16 9:00 AM	15.5	15.3	15.8	15.5
2022-08-16 10:00 AM	16.5	16.5	16.4	16.5
2022-08-16 11:00 AM	19.0	18.5	17.1	18.2
2022-08-16 12:00 PM	20.6	19.6	18.1	19.4
2022-08-16 1:00 PM	21.3	19.9	19.2	20.1
2022-08-16 2:00 PM	21.3	19.9	20.5	20.6
2022-08-16 3:00 PM	20.9	19.9	24.0	21.6
2022-08-16 4:00 PM	20.4	19.8	24.7	21.6
2022-08-16 5:00 PM	20.3	19.9	23.3	21.2
2022-08-16 6:00 PM	20.2	19.9	22.5	20.9

2022-08-16 7:00 PM	19.5	19.2	20.3	19.7
2022-08-16 8:00 PM	18.9	18.6	19.5	19.0
2022-08-16 9:00 PM	18.8	18.5	19.1	18.8
2022-08-16 10:00 PM	18.4	18.1	18.8	18.4
2022-08-16 11:00 PM	18.0	17.8	18.4	18.1
2022-08-17 12:00 AM	17.7	17.5	18.0	17.7
2022-08-17 1:00 AM	17.8	17.7	18.1	17.9
2022-08-17 2:00 AM	17.9	17.8	18.2	18.0
2022-08-17 3:00 AM	17.9	17.9	18.2	18.0
2022-08-17 4:00 AM	17.8	17.8	18.1	17.9
2022-08-17 5:00 AM	17.6	17.5	17.8	17.6
2022-08-17 6:00 AM	17.4	17.3	17.6	17.4
2022-08-17 7:00 AM	17.2	17.1	17.4	17.2
2022-08-17 8:00 AM	17.3	17.2	17.5	17.3
2022-08-17 9:00 AM	17.7	17.7	17.8	17.7
2022-08-17 10:00 AM	18.1	18.0	18.0	18.0
2022-08-17 11:00 AM	19.1	18.9	18.4	18.8
2022-08-17 12:00 PM	20.5	19.9	18.8	19.7
2022-08-17 1:00 PM	21.1	20.3	19.7	20.4
2022-08-17 2:00 PM	21.6	20.6	20.4	20.9
2022-08-17 3:00 PM	21.7	20.8	21.2	21.2
2022-08-17 4:00 PM	21.5	20.9	23.6	22.0
2022-08-17 5:00 PM	20.9	20.4	21.3	20.9
2022-08-17 6:00 PM	20.3	19.8	20.2	20.1
2022-08-17 7:00 PM	20.2	19.8	20.1	20.0
2022-08-17 8:00 PM	19.5	19.2	19.5	19.4
2022-08-17 9:00 PM	19.1	18.9	19.1	19.0
2022-08-17 10:00 PM	18.6	18.5	18.7	18.6
2022-08-17 11:00 PM	18.2	18.1	18.3	18.2
2022-08-18 12:00 AM	17.9	17.9	18.1	18.0
2022-08-18 1:00 AM	17.9	17.9	18.1	18.0
2022-08-18 2:00 AM	18.0	18.0	18.2	18.1
2022-08-18 3:00 AM	17.8	17.8	18.0	17.9
2022-08-18 4:00 AM	17.6	17.6	17.7	17.6
2022-08-18 5:00 AM	17.3	17.4	17.5	17.4
2022-08-18 6:00 AM	16.9	17.1	17.2	17.1
2022-08-18 7:00 AM	16.8	16.9	17.0	16.9
2022-08-18 8:00 AM	17.0	17.0	17.1	17.0
2022-08-18 9:00 AM	17.3	17.3	17.4	17.3
2022-08-18 10:00 AM	18.3	18.2	17.9	18.1
2022-08-18 11:00 AM	20.1	19.8	18.6	19.5
2022-08-18 12:00 PM	21.7	20.9	19.8	20.8
2022-08-18 1:00 PM	23.6	22.0	21.6	22.4
2022-08-18 2:00 PM	22.8	21.7	21.8	22.1
2022-08-18 3:00 PM	21.2	20.6	20.8	20.9

2022-08-18 4:00 PM	20.6	20.2	20.1	20.3
2022-08-18 5:00 PM	20.9	20.5	20.3	20.6
2022-08-18 6:00 PM	20.3	20.0	19.9	20.1
2022-08-18 7:00 PM	20.3	20.0	19.9	20.1
2022-08-18 8:00 PM	20.1	19.9	20.0	20.0
2022-08-18 9:00 PM	19.4	19.3	19.4	19.4
2022-08-18 10:00 PM	18.8	18.8	18.9	18.8
2022-08-18 11:00 PM	18.5	18.5	18.6	18.5
2022-08-19 12:00 AM	18.2	18.2	18.4	18.3
2022-08-19 1:00 AM	18.4	18.4	18.5	18.4
2022-08-19 2:00 AM	18.5	18.5	18.6	18.5
2022-08-19 3:00 AM	18.5	18.5	18.5	18.5
2022-08-19 4:00 AM	18.4	18.4	18.5	18.4
2022-08-19 5:00 AM	18.4	18.4	18.5	18.4
2022-08-19 6:00 AM	18.4	18.4	18.5	18.4
2022-08-19 7:00 AM	18.2	18.3	18.4	18.3
2022-08-19 8:00 AM	18.2	18.2	18.3	18.2
2022-08-19 9:00 AM	18.4	18.3	18.4	18.4
2022-08-19 10:00 AM	18.6	18.5	18.6	18.6
2022-08-19 11:00 AM	19.2	19.0	18.9	19.0
2022-08-19 12:00 PM	20.0	19.6	19.5	19.7
2022-08-19 1:00 PM	21.2	20.5	20.3	20.7
2022-08-19 2:00 PM	21.9	20.8	21.8	21.5
2022-08-19 3:00 PM	21.6	20.7	25.1	22.5
2022-08-19 4:00 PM	21.5	20.9	26.8	23.1
2022-08-19 5:00 PM	21.8	21.4	26.4	23.2
2022-08-19 6:00 PM	21.4	21.0	22.9	21.8
2022-08-19 7:00 PM	20.9	20.5	21.6	21.0
2022-08-19 8:00 PM	20.8	20.4	21.3	20.8
2022-08-19 9:00 PM	20.2	20.0	20.8	20.3
2022-08-19 10:00 PM	19.8	19.7	20.3	19.9
2022-08-19 11:00 PM	19.3	19.3	19.9	19.5
2022-08-20 12:00 AM	19.0	19.0	19.5	19.2
2022-08-20 1:00 AM	18.8	18.8	19.2	18.9
2022-08-20 2:00 AM	18.8	18.8	19.2	18.9
2022-08-20 3:00 AM	18.7	18.7	19.1	18.8
2022-08-20 4:00 AM	18.5	18.6	18.9	18.7
2022-08-20 5:00 AM	18.4	18.5	18.7	18.5
2022-08-20 6:00 AM	18.2	18.3	18.6	18.4
2022-08-20 7:00 AM	17.8	18.0	18.3	18.0
2022-08-20 8:00 AM	17.5	17.7	18.0	17.7
2022-08-20 9:00 AM	17.9	18.0	18.1	18.0
2022-08-20 10:00 AM	18.2	18.3	18.2	18.2
2022-08-20 11:00 AM	20.3	20.5	18.5	19.8
2022-08-20 12:00 PM	21.6	21.1	19.5	20.7

2022-08-20 1:00 PM	21.1	20.5	19.9	20.5
2022-08-20 2:00 PM	21.7	20.7	21.1	21.2
2022-08-20 3:00 PM	21.3	20.4	24.8	22.2
2022-08-20 4:00 PM	20.6	20.1	26.2	22.3
2022-08-20 5:00 PM	20.5	20.2	25.9	22.2
2022-08-20 6:00 PM	20.5	20.3	23.9	21.6
2022-08-20 7:00 PM	20.1	19.8	21.5	20.5
2022-08-20 8:00 PM	19.2	19.1	20.3	19.5
2022-08-20 9:00 PM	18.8	18.7	19.7	19.1
2022-08-20 10:00 PM	18.1	18.2	19.0	18.4
2022-08-20 11:00 PM	17.7	17.6	18.4	17.9
2022-08-21 12:00 AM	17.2	17.2	17.8	17.4
2022-08-21 1:00 AM	16.7	16.8	17.2	16.9
2022-08-21 2:00 AM	16.0	16.3	16.6	16.3
2022-08-21 3:00 AM	15.7	15.9	16.1	15.9
2022-08-21 4:00 AM	14.8	15.5	15.6	15.3
2022-08-21 5:00 AM	14.4	15.2	15.3	15.0
2022-08-21 6:00 AM	14.7	14.8	14.9	14.8
2022-08-21 7:00 AM	14.1	14.7	14.7	14.5
2022-08-21 8:00 AM	13.9	14.6	14.5	14.3
2022-08-21 9:00 AM	14.6	15.0	15.2	14.9
2022-08-21 10:00 AM	16.1	16.3	16.1	16.2
2022-08-21 11:00 AM	19.0	19.1	16.9	18.3
2022-08-21 12:00 PM	20.7	20.4	18.2	19.8
2022-08-21 1:00 PM	21.6	20.1	19.4	20.4
2022-08-21 2:00 PM	21.6	20.3	21.5	21.1
2022-08-21 3:00 PM	20.8	19.9	25.4	22.0
2022-08-21 4:00 PM	20.3	19.8	26.2	22.1
2022-08-21 5:00 PM	20.3	20.0	26.7	22.3
2022-08-21 6:00 PM	20.2	20.0	24.3	21.5
2022-08-21 7:00 PM	19.6	19.3	21.1	20.0
2022-08-21 8:00 PM	19.1	18.9	20.2	19.4
2022-08-21 9:00 PM	18.7	18.5	19.6	18.9
2022-08-21 10:00 PM	18.1	18.1	19.0	18.4
2022-08-21 11:00 PM	17.8	17.8	18.5	18.0
2022-08-22 12:00 AM	17.5	17.6	18.2	17.8
2022-08-22 1:00 AM	17.2	17.2	17.8	17.4
2022-08-22 2:00 AM	16.9	16.9	17.5	17.1
2022-08-22 3:00 AM	16.6	16.7	17.2	16.8
2022-08-22 4:00 AM	16.3	16.5	16.8	16.5
2022-08-22 5:00 AM	15.8	16.2	16.4	16.1
2022-08-22 6:00 AM	15.6	16.0	16.2	15.9
2022-08-22 7:00 AM	15.4	15.9	15.9	15.7
2022-08-22 8:00 AM	15.4	15.8	15.9	15.7
2022-08-22 9:00 AM	15.8	16.0	16.2	16.0

2022-08-22 10:00 AM	17.3	17.2	17.1	17.2
2022-08-22 11:00 AM	19.5	19.0	18.1	18.9
2022-08-22 12:00 PM	20.7	20.1	19.1	20.0
2022-08-22 1:00 PM	22.1	20.5	20.0	20.9
2022-08-22 2:00 PM	21.5	20.3	20.4	20.7
2022-08-22 3:00 PM	21.3	20.2	22.5	21.3
2022-08-22 4:00 PM	20.6	19.8	20.8	20.4
2022-08-22 5:00 PM	19.7	19.2	19.5	19.5
2022-08-22 6:00 PM	20.0	19.4	19.8	19.7
2022-08-22 7:00 PM	20.3	19.7	20.0	20.0
2022-08-22 8:00 PM	19.9	19.4	19.8	19.7
2022-08-22 9:00 PM	19.4	19.1	19.5	19.3
2022-08-22 10:00 PM	19.0	18.8	19.1	19.0
2022-08-22 11:00 PM	19.0	18.8	19.1	19.0
2022-08-23 12:00 AM	19.0	18.8	19.0	18.9
2022-08-23 1:00 AM	18.8	18.7	18.8	18.8
2022-08-23 2:00 AM	18.7	18.6	18.8	18.7
2022-08-23 3:00 AM	18.7	18.7	18.8	18.7
2022-08-23 4:00 AM	18.7	18.6	18.8	18.7
2022-08-23 5:00 AM	18.6	18.6	18.6	18.6
2022-08-23 6:00 AM	18.4	18.4	18.5	18.4
2022-08-23 7:00 AM	18.2	18.3	18.3	18.3
2022-08-23 8:00 AM	18.2	18.2	18.2	18.2
2022-08-23 9:00 AM	18.5	18.4	18.4	18.4
2022-08-23 10:00 AM	18.9	18.6	18.6	18.7
2022-08-23 11:00 AM	19.8	19.2	19.2	19.4
2022-08-23 12:00 PM	20.2	19.6	19.7	19.8
2022-08-23 1:00 PM	21.1	20.0	20.3	20.5
2022-08-23 2:00 PM	22.0	20.7	21.5	21.4
2022-08-23 3:00 PM	21.8	20.7	22.2	21.6
2022-08-23 4:00 PM	21.4	20.6	23.3	21.8
2022-08-23 5:00 PM	21.4	20.8	25.9	22.7
2022-08-23 6:00 PM	21.5	20.9	24.1	22.2
2022-08-23 7:00 PM	20.8	20.3	21.6	20.9
2022-08-23 8:00 PM	20.0	19.8	20.6	20.1
2022-08-23 9:00 PM	19.3	19.2	19.9	19.5
2022-08-23 10:00 PM	18.9	19.0	19.5	19.1
2022-08-23 11:00 PM	18.5	18.5	18.9	18.6
2022-08-24 12:00 AM	18.1	18.2	18.5	18.3
2022-08-24 1:00 AM	17.8	17.8	17.9	17.8
2022-08-24 2:00 AM	17.4	17.5	17.5	17.5
2022-08-24 3:00 AM	16.9	17.1	16.9	17.0
2022-08-24 4:00 AM	16.6	16.8	16.6	16.7
2022-08-24 5:00 AM	16.7	16.7	16.7	16.7
2022-08-24 6:00 AM	16.8	16.8	16.9	16.8

2022-08-24 7:00 AM	16.9	16.9	17.0	16.9
2022-08-24 8:00 AM	16.9	16.9	17.0	16.9
2022-08-24 9:00 AM	17.1	17.1	17.2	17.1
2022-08-24 10:00 AM	17.3	17.3	17.4	17.3
2022-08-24 11:00 AM	17.7	17.6	17.7	17.7
2022-08-24 12:00 PM	17.9	17.8	17.9	17.9
2022-08-24 1:00 PM	18.0	17.9	18.0	18.0
2022-08-24 2:00 PM	18.1	18.0	18.0	18.0
2022-08-24 3:00 PM	18.2	18.0	18.0	18.1
2022-08-24 4:00 PM	18.3	18.1	18.0	18.1
2022-08-24 5:00 PM	18.5	18.2	18.3	18.3
2022-08-24 6:00 PM	18.6	18.3	18.4	18.4
2022-08-24 7:00 PM	18.5	18.2	18.4	18.4
2022-08-24 8:00 PM	18.2	18.0	18.2	18.1
2022-08-24 9:00 PM	17.9	17.8	17.9	17.9
2022-08-24 10:00 PM	17.7	17.6	17.7	17.7
2022-08-24 11:00 PM	17.6	17.5	17.6	17.6
2022-08-25 12:00 AM	17.2	17.3	17.3	17.3
2022-08-25 1:00 AM	16.8	16.8	16.9	16.8
2022-08-25 2:00 AM	16.6	16.7	16.6	16.6
2022-08-25 3:00 AM	16.5	16.6	16.3	16.5
2022-08-25 4:00 AM	16.5	16.5	16.5	16.5
2022-08-25 5:00 AM	16.3	16.5	16.2	16.3
2022-08-25 6:00 AM	16.0	16.0	15.9	16.0
2022-08-25 7:00 AM	16.1	16.1	16.1	16.1
2022-08-25 8:00 AM	15.9	15.9	15.9	15.9
2022-08-25 9:00 AM	15.9	15.9	15.9	15.9
2022-08-25 10:00 AM	16.7	16.7	16.4	16.6
2022-08-25 11:00 AM	17.3	17.1	16.9	17.1
2022-08-25 12:00 PM	18.2	17.8	17.6	17.9
2022-08-25 1:00 PM	20.1	18.9	18.9	19.3
2022-08-25 2:00 PM	20.0	19.1	19.5	19.5
2022-08-25 3:00 PM	19.7	19.0	21.4	20.0
2022-08-25 4:00 PM	19.6	19.0	20.7	19.8
2022-08-25 5:00 PM	19.4	18.9	19.4	19.2
2022-08-25 6:00 PM	19.3	18.8	19.3	19.1
2022-08-25 7:00 PM	19.1	18.6	18.9	18.9
2022-08-25 8:00 PM	18.7	18.3	18.6	18.5
2022-08-25 9:00 PM	18.2	18.0	18.2	18.1
2022-08-25 10:00 PM	18.0	17.8	18.1	18.0
2022-08-25 11:00 PM	17.9	17.7	17.9	17.8
2022-08-26 12:00 AM	17.6	17.5	17.6	17.6
2022-08-26 1:00 AM	17.4	17.4	17.5	17.4
2022-08-26 2:00 AM	17.4	17.4	17.5	17.4
2022-08-26 3:00 AM	17.0	17.0	17.2	17.1

2022-08-26 4:00 AM	16.5	16.6	16.7	16.6
2022-08-26 5:00 AM	16.4	16.5	16.6	16.5
2022-08-26 6:00 AM	16.5	16.5	16.5	16.5
2022-08-26 7:00 AM	16.3	16.3	16.3	16.3
2022-08-26 8:00 AM	16.4	16.4	16.4	16.4
2022-08-26 9:00 AM	16.8	16.7	16.7	16.7
2022-08-26 10:00 AM	17.3	17.2	17.1	17.2
2022-08-26 11:00 AM	18.7	18.6	17.5	18.3
2022-08-26 12:00 PM	19.5	19.1	18.4	19.0
2022-08-26 1:00 PM	20.4	19.4	19.3	19.7
2022-08-26 2:00 PM	20.8	19.7	20.6	20.4
2022-08-26 3:00 PM	20.8	19.8	22.5	21.0
2022-08-26 4:00 PM	20.6	19.9	22.5	21.0
2022-08-26 5:00 PM	20.6	20.1	23.6	21.4
2022-08-26 6:00 PM	20.6	20.3	22.5	21.1
2022-08-26 7:00 PM	20.3	19.9	21.0	20.4
2022-08-26 8:00 PM	20.1	19.7	20.6	20.1
2022-08-26 9:00 PM	19.7	19.3	20.2	19.7
2022-08-26 10:00 PM	19.2	19.0	19.7	19.3
2022-08-26 11:00 PM	18.9	18.7	19.3	19.0
2022-08-27 12:00 AM	18.6	18.5	19.0	18.7
2022-08-27 1:00 AM	18.2	18.2	18.6	18.3
2022-08-27 2:00 AM	18.0	18.0	18.4	18.1
2022-08-27 3:00 AM	17.8	17.8	18.2	17.9
2022-08-27 4:00 AM	17.5	17.6	17.8	17.6
2022-08-27 5:00 AM	17.3	17.4	17.6	17.4
2022-08-27 6:00 AM	17.1	17.2	17.4	17.2
2022-08-27 7:00 AM	16.9	17.0	17.1	17.0
2022-08-27 8:00 AM	16.7	16.8	17.0	16.8
2022-08-27 9:00 AM	17.1	17.1	17.2	17.1
2022-08-27 10:00 AM	18.1	18.0	17.9	18.0
2022-08-27 11:00 AM	19.5	19.2	18.7	19.1
2022-08-27 12:00 PM	20.2	19.6	19.5	19.8
2022-08-27 1:00 PM	21.0	20.1	20.1	20.4
2022-08-27 2:00 PM	20.6	19.9	20.2	20.2
2022-08-27 3:00 PM	20.5	19.8	20.1	20.1
2022-08-27 4:00 PM	20.0	19.5	19.7	19.7
2022-08-27 5:00 PM	19.5	19.1	19.2	19.3
2022-08-27 6:00 PM	19.5	19.1	19.2	19.3
2022-08-27 7:00 PM	19.3	19.0	19.1	19.1
2022-08-27 8:00 PM	19.2	18.9	19.0	19.0
2022-08-27 9:00 PM	19.1	18.9	19.0	19.0
2022-08-27 10:00 PM	19.0	18.8	18.9	18.9
2022-08-27 11:00 PM	18.9	18.8	18.9	18.9
2022-08-28 12:00 AM	18.8	18.6	18.8	18.7

2022-08-28 1:00 AM	18.6	18.5	18.7	18.6
2022-08-28 2:00 AM	18.8	18.7	18.8	18.8
2022-08-28 3:00 AM	18.8	18.7	18.8	18.8
2022-08-28 4:00 AM	18.8	18.6	18.8	18.7
2022-08-28 5:00 AM	18.8	18.6	18.8	18.7
2022-08-28 6:00 AM	18.6	18.5	18.7	18.6
2022-08-28 7:00 AM	18.2	18.2	18.4	18.3
2022-08-28 8:00 AM	18.3	18.3	18.4	18.3
2022-08-28 9:00 AM	18.6	18.5	18.6	18.6
2022-08-28 10:00 AM	18.9	18.7	18.7	18.8
2022-08-28 11:00 AM	19.6	19.2	19.1	19.3
2022-08-28 12:00 PM	20.3	19.9	20.0	20.1
2022-08-28 1:00 PM	20.9	20.3	20.5	20.6
2022-08-28 2:00 PM	20.5	20.0	20.3	20.3
2022-08-28 3:00 PM	20.6	20.1	20.4	20.4
2022-08-28 4:00 PM	20.9	20.4	20.7	20.7
2022-08-28 5:00 PM	20.5	20.0	20.2	20.2
2022-08-28 6:00 PM	20.3	19.9	20.0	20.1
2022-08-28 7:00 PM	20.4	19.9	20.1	20.1
2022-08-28 8:00 PM	20.2	19.8	20.0	20.0
2022-08-28 9:00 PM	19.8	19.5	19.7	19.7
2022-08-28 10:00 PM	19.6	19.3	19.5	19.5
2022-08-28 11:00 PM	19.3	19.1	19.3	19.2
2022-08-29 12:00 AM	18.9	18.9	19.0	18.9
2022-08-29 1:00 AM	18.5	18.5	18.6	18.5
2022-08-29 2:00 AM	18.3	18.3	18.5	18.4
2022-08-29 3:00 AM	18.5	18.5	18.6	18.5
2022-08-29 4:00 AM	18.6	18.6	18.7	18.6
2022-08-29 5:00 AM	18.5	18.5	18.7	18.6
2022-08-29 6:00 AM	18.6	18.6	18.7	18.6
2022-08-29 7:00 AM	18.6	18.6	18.7	18.6
2022-08-29 8:00 AM	18.4	18.4	18.5	18.4
2022-08-29 9:00 AM	18.8	18.7	18.7	18.7
2022-08-29 10:00 AM	19.0	18.9	18.8	18.9
2022-08-29 11:00 AM	20.3	20.0	19.3	19.9
2022-08-29 12:00 PM	21.3	20.4	20.3	20.7
2022-08-29 1:00 PM	21.5	20.5	20.7	20.9
2022-08-29 2:00 PM	21.7	20.6	22.1	21.5
2022-08-29 3:00 PM	21.0	20.4	22.8	21.4
2022-08-29 4:00 PM	20.7	20.2	23.8	21.6
2022-08-29 5:00 PM	20.8	20.4	23.9	21.7
2022-08-29 6:00 PM	20.7	20.3	22.1	21.0
2022-08-29 7:00 PM	20.4	20.0	21.1	20.5
2022-08-29 8:00 PM	20.3	20.0	20.8	20.4
2022-08-29 9:00 PM	19.6	19.5	20.1	19.7

2022-08-29 10:00 PM	19.3	19.2	19.7	19.4
2022-08-29 11:00 PM	19.1	19.1	19.5	19.2
2022-08-30 12:00 AM	19.1	19.0	19.4	19.2
2022-08-30 1:00 AM	18.6	18.7	19.0	18.8
2022-08-30 2:00 AM	18.2	18.3	18.5	18.3
2022-08-30 3:00 AM	18.1	18.1	18.3	18.2
2022-08-30 4:00 AM	18.0	18.1	18.2	18.1
2022-08-30 5:00 AM	18.0	18.1	18.3	18.1
2022-08-30 6:00 AM	17.6	17.8	17.8	17.7
2022-08-30 7:00 AM	17.0	17.4	17.2	17.2
2022-08-30 8:00 AM	16.3	16.8	16.5	16.5
2022-08-30 9:00 AM	15.7	16.3	15.9	16.0
2022-08-30 10:00 AM	15.8	16.2	15.9	16.0
2022-08-30 11:00 AM	17.2	17.5	16.4	17.0
2022-08-30 12:00 PM	18.4	18.0	17.2	17.9
2022-08-30 1:00 PM	19.1	18.3	18.0	18.5
2022-08-30 2:00 PM	19.3	18.3	19.1	18.9
2022-08-30 3:00 PM	19.1	18.0	20.3	19.1
2022-08-30 4:00 PM	19.2	18.2	21.3	19.6
2022-08-30 5:00 PM	19.4	18.5	21.6	19.8
2022-08-30 6:00 PM	19.0	18.3	19.5	18.9
2022-08-30 7:00 PM	18.8	18.2	18.9	18.6
2022-08-30 8:00 PM	18.5	18.1	18.6	18.4
2022-08-30 9:00 PM	18.1	17.9	18.3	18.1
2022-08-30 10:00 PM	17.6	17.5	17.9	17.7
2022-08-30 11:00 PM	17.2	17.2	17.6	17.3
2022-08-31 12:00 AM	17.0	17.2	17.3	17.2
2022-08-31 1:00 AM	16.8	17.0	17.2	17.0
2022-08-31 2:00 AM	16.5	16.8	16.9	16.7
2022-08-31 3:00 AM	16.3	16.6	16.7	16.5
2022-08-31 4:00 AM	16.1	16.4	16.4	16.3
2022-08-31 5:00 AM	15.9	16.2	16.3	16.1
2022-08-31 6:00 AM	15.6	15.9	16.0	15.8
2022-08-31 7:00 AM	15.4	15.8	15.8	15.7
2022-08-31 8:00 AM	15.1	15.5	15.5	15.4
2022-08-31 9:00 AM	14.9	15.3	15.3	15.2
2022-08-31 10:00 AM	14.8	15.3	15.0	15.0
2022-08-31 11:00 AM	16.2	16.5	15.3	16.0
2022-08-31 12:00 PM	17.4	16.9	16.0	16.8
2022-08-31 1:00 PM	17.7	17.3	16.5	17.2
2022-08-31 2:00 PM	17.9	17.1	17.9	17.6
2022-08-31 3:00 PM	17.2	16.8	19.7	17.9
2022-08-31 4:00 PM	17.1	16.5	20.9	18.2
2022-08-31 5:00 PM	17.3	16.6	21.2	18.4
2022-08-31 6:00 PM	17.3	16.8	19.9	18.0

2022-08-31 7:00 PM	16.8	16.4	17.7	17.0
2022-08-31 8:00 PM	16.4	16.1	17.1	16.5
2022-08-31 9:00 PM	16.3	16.1	16.8	16.4
2022-08-31 10:00 PM	15.9	15.9	16.4	16.1
2022-08-31 11:00 PM	15.7	15.7	16.3	15.9
2022-09-01 12:00 AM	15.6	15.6	16.1	15.8
2022-09-01 1:00 AM	15.9	15.9	16.2	16.0
2022-09-01 2:00 AM	15.9	15.8	16.1	15.9
2022-09-01 3:00 AM	15.2	15.3	15.6	15.4
2022-09-01 4:00 AM	15.1	15.2	15.4	15.2
2022-09-01 5:00 AM	15.1	15.2	15.5	15.3
2022-09-01 6:00 AM	14.8	15.0	15.1	15.0
2022-09-01 7:00 AM	14.6	14.8	15.0	14.8
2022-09-01 8:00 AM	14.6	14.7	14.9	14.7
2022-09-01 9:00 AM	14.9	14.9	15.1	15.0
2022-09-01 10:00 AM	15.8	15.7	15.8	15.8
2022-09-01 11:00 AM	17.1	16.4	16.2	16.6
2022-09-01 12:00 PM	17.8	16.8	16.8	17.1
2022-09-01 1:00 PM	18.8	17.4	17.6	17.9
2022-09-01 2:00 PM	19.2	17.8	19.7	18.9
2022-09-01 3:00 PM	18.5	17.5	21.0	19.0
2022-09-01 4:00 PM	18.2	17.5	21.3	19.0
2022-09-01 5:00 PM	18.4	17.8	22.8	19.7
2022-09-01 6:00 PM	18.6	18.2	21.9	19.6
2022-09-01 7:00 PM	18.1	17.7	19.5	18.4
2022-09-01 8:00 PM	17.8	17.6	18.8	18.1
2022-09-01 9:00 PM	17.5	17.4	18.3	17.7
2022-09-01 10:00 PM	17.2	17.1	17.9	17.4
2022-09-01 11:00 PM	16.8	16.8	17.5	17.0
2022-09-02 12:00 AM	16.4	16.5	17.1	16.7
2022-09-02 1:00 AM	16.1	16.2	16.7	16.3
2022-09-02 2:00 AM	15.9	16.0	16.4	16.1
2022-09-02 3:00 AM	15.8	15.9	16.2	16.0
2022-09-02 4:00 AM	15.6	15.7	16.0	15.8
2022-09-02 5:00 AM	15.5	15.6	16.0	15.7
2022-09-02 6:00 AM	15.4	15.5	15.9	15.6
2022-09-02 7:00 AM	15.3	15.4	15.8	15.5
2022-09-02 8:00 AM	15.5	15.7	16.0	15.7
2022-09-02 9:00 AM	16.1	16.2	16.4	16.2
2022-09-02 10:00 AM	16.7	16.6	16.8	16.7
2022-09-02 11:00 AM	18.5	17.7	17.9	18.0
2022-09-02 12:00 PM	18.9	18.1	18.7	18.6
2022-09-02 1:00 PM	19.2	18.3	19.1	18.9
2022-09-02 2:00 PM	20.3	19.1	21.0	20.1
2022-09-02 3:00 PM	20.6	19.5	22.4	20.8

2022-09-02 4:00 PM	20.4	19.5	21.8	20.6
2022-09-02 5:00 PM	19.8	19.2	22.2	20.4
2022-09-02 6:00 PM	19.3	18.9	21.6	19.9
2022-09-02 7:00 PM	19.0	18.4	20.0	19.1
2022-09-02 8:00 PM	18.6	17.8	19.1	18.5
2022-09-02 9:00 PM	18.2	17.8	18.6	18.2
2022-09-02 10:00 PM	17.8	17.5	18.3	17.9
2022-09-02 11:00 PM	17.3	17.1	17.8	17.4
2022-09-03 12:00 AM	16.8	16.8	17.4	17.0
2022-09-03 1:00 AM	16.3	16.4	16.9	16.5
2022-09-03 2:00 AM	15.8	16.1	16.3	16.1
2022-09-03 3:00 AM	15.2	15.6	15.6	15.5
2022-09-03 4:00 AM	14.6	15.1	15.1	14.9
2022-09-03 5:00 AM	14.0	14.5	14.4	14.3
2022-09-03 6:00 AM	13.5	13.9	13.8	13.7
2022-09-03 7:00 AM	12.6	13.3	13.0	13.0
2022-09-03 8:00 AM	12.4	13.0	12.7	12.7
2022-09-03 9:00 AM	12.7	13.4	13.0	13.0
2022-09-03 10:00 AM	13.2	13.7	13.0	13.3
2022-09-03 11:00 AM	14.3	14.4	13.2	14.0
2022-09-03 12:00 PM	15.7	14.9	14.1	14.9
2022-09-03 1:00 PM	16.1	15.3	15.1	15.5
2022-09-03 2:00 PM	16.4	15.4	16.9	16.2
2022-09-03 3:00 PM	16.2	15.5	19.2	17.0
2022-09-03 4:00 PM	16.1	15.7	19.6	17.1
2022-09-03 5:00 PM	16.2	15.9	21.6	17.9
2022-09-03 6:00 PM	16.1	16.2	20.1	17.5
2022-09-03 7:00 PM	15.6	15.6	17.4	16.2
2022-09-03 8:00 PM	15.3	15.3	16.4	15.7
2022-09-03 9:00 PM	14.8	14.8	15.7	15.1
2022-09-03 10:00 PM	14.1	14.4	15.0	14.5
2022-09-03 11:00 PM	13.6	14.0	14.3	14.0
2022-09-04 12:00 AM	13.2	13.4	13.7	13.4
2022-09-04 1:00 AM	12.7	13.0	13.1	12.9
2022-09-04 2:00 AM	11.9	12.5	12.5	12.3
2022-09-04 3:00 AM	11.6	12.0	12.1	11.9
2022-09-04 4:00 AM	11.0	11.6	11.4	11.3
2022-09-04 5:00 AM	10.7	11.2	11.0	11.0
2022-09-04 6:00 AM	10.3	10.9	10.5	10.6
2022-09-04 7:00 AM	9.5	10.5	9.8	9.9
2022-09-04 8:00 AM	9.4	10.3	9.8	9.8
2022-09-04 9:00 AM	10.2	10.7	10.6	10.5
2022-09-04 10:00 AM	11.2	11.7	11.5	11.5
2022-09-04 11:00 AM	13.7	13.3	12.8	13.3
2022-09-04 12:00 PM	15.7	14.4	14.4	14.8

2022-09-04 1:00 PM	16.2	14.9	15.5	15.5
2022-09-04 2:00 PM	16.5	15.2	17.2	16.3
2022-09-04 3:00 PM	15.8	15.0	18.7	16.5
2022-09-04 4:00 PM	15.5	15.0	18.8	16.4
2022-09-04 5:00 PM	15.4	15.0	20.4	16.9
2022-09-04 6:00 PM	15.4	15.3	19.0	16.6
2022-09-04 7:00 PM	14.9	14.8	16.5	15.4
2022-09-04 8:00 PM	14.3	14.4	15.4	14.7
2022-09-04 9:00 PM	13.7	13.8	14.6	14.0
2022-09-04 10:00 PM	13.1	13.3	14.2	13.5
2022-09-04 11:00 PM	12.8	12.9	13.6	13.1
2022-09-05 12:00 AM	12.3	12.3	12.9	12.5
2022-09-05 1:00 AM	11.8	11.8	12.3	12.0
2022-09-05 2:00 AM	11.4	11.5	11.8	11.6
2022-09-05 3:00 AM	10.9	11.2	11.4	11.2
2022-09-05 4:00 AM	10.6	10.8	11.4	10.9
2022-09-05 5:00 AM	10.1	10.6	10.6	10.4
2022-09-05 6:00 AM	9.2	10.2	9.7	9.7
2022-09-05 7:00 AM	9.3	9.9	9.5	9.6
2022-09-05 8:00 AM	9.0	9.6	9.2	9.3
2022-09-05 9:00 AM	9.5	9.9	9.6	9.7
2022-09-05 10:00 AM	10.5	11.0	10.7	10.7
2022-09-05 11:00 AM	13.3	12.6	12.2	12.7
2022-09-05 12:00 PM	15.1	13.7	13.6	14.1
2022-09-05 1:00 PM	15.6	14.3	15.0	15.0
2022-09-05 2:00 PM	16.1	14.6	16.8	15.8
2022-09-05 3:00 PM	15.4	14.5	18.0	16.0
2022-09-05 4:00 PM	15.2	14.5	18.2	16.0
2022-09-05 5:00 PM	15.5	15.0	20.6	17.0
2022-09-05 6:00 PM	15.5	15.3	19.4	16.7
2022-09-05 7:00 PM	15.1	14.8	16.8	15.6
2022-09-05 8:00 PM	14.7	14.5	15.9	15.0
2022-09-05 9:00 PM	14.2	14.1	15.1	14.5
2022-09-05 10:00 PM	13.7	13.7	14.6	14.0
2022-09-05 11:00 PM	13.4	13.5	14.3	13.7
2022-09-06 12:00 AM	13.1	13.3	13.9	13.4
2022-09-06 1:00 AM	12.8	12.9	13.3	13.0
2022-09-06 2:00 AM	12.3	12.5	13.0	12.6
2022-09-06 3:00 AM	12.1	12.3	12.7	12.4
2022-09-06 4:00 AM	11.8	11.9	12.1	11.9
2022-09-06 5:00 AM	11.3	11.6	11.4	11.4
2022-09-06 6:00 AM	10.7	11.2	10.9	10.9
2022-09-06 7:00 AM	10.5	10.9	10.5	10.6
2022-09-06 8:00 AM	10.3	10.8	10.3	10.5
2022-09-06 9:00 AM	10.5	11.0	10.7	10.7

2022-09-06 10:00 AM	11.7	11.9	11.9	11.8
2022-09-06 11:00 AM	14.3	13.6	13.2	13.7
2022-09-06 12:00 PM	16.1	14.6	14.8	15.2
2022-09-06 1:00 PM	16.5	15.0	16.0	15.8
2022-09-06 2:00 PM	17.2	15.8	17.6	16.9
2022-09-06 3:00 PM	16.5	15.5	18.0	16.7
2022-09-06 4:00 PM	16.6	15.7	18.3	16.9
2022-09-06 5:00 PM	17.0	16.3	21.0	18.1
2022-09-06 6:00 PM	17.0	16.7	20.4	18.0
2022-09-06 7:00 PM	16.7	16.3	18.3	17.1
2022-09-06 8:00 PM	16.4	16.0	17.5	16.6
2022-09-06 9:00 PM	16.0	15.8	17.0	16.3
2022-09-06 10:00 PM	15.7	15.6	16.6	16.0
2022-09-06 11:00 PM	15.7	15.5	16.4	15.9
2022-09-07 12:00 AM	15.6	15.4	16.2	15.7
2022-09-07 1:00 AM	15.2	15.2	15.8	15.4
2022-09-07 2:00 AM	14.9	14.9	15.4	15.1
2022-09-07 3:00 AM	14.6	14.6	15.0	14.7
2022-09-07 4:00 AM	14.4	14.4	14.8	14.5
2022-09-07 5:00 AM	14.2	14.3	14.7	14.4
2022-09-07 6:00 AM	13.9	14.1	14.4	14.1
2022-09-07 7:00 AM	13.7	13.8	14.0	13.8
2022-09-07 8:00 AM	13.5	13.6	13.8	13.6
2022-09-07 9:00 AM	13.7	13.8	14.1	13.9
2022-09-07 10:00 AM	14.5	14.5	14.8	14.6
2022-09-07 11:00 AM	16.7	15.8	15.7	16.1
2022-09-07 12:00 PM	18.1	16.6	17.1	17.3
2022-09-07 1:00 PM	18.3	16.9	18.3	17.8
2022-09-07 2:00 PM	18.8	17.2	20.1	18.7
2022-09-07 3:00 PM	17.9	16.8	20.8	18.5
2022-09-07 4:00 PM	17.7	16.8	19.9	18.1
2022-09-07 5:00 PM	17.9	17.2	23.1	19.4
2022-09-07 6:00 PM	17.8	17.5	21.9	19.1
2022-09-07 7:00 PM	17.4	16.9	19.2	17.8
2022-09-07 8:00 PM	16.9	16.5	18.0	17.1
2022-09-07 9:00 PM	16.2	15.9	17.2	16.4
2022-09-07 10:00 PM	15.6	15.5	16.5	15.9
2022-09-07 11:00 PM	15.1	15.1	15.8	15.3
2022-09-08 12:00 AM	14.6	14.6	15.3	14.8
2022-09-08 1:00 AM	14.1	14.2	14.8	14.4
2022-09-08 2:00 AM	13.5	13.7	14.4	13.9
2022-09-08 3:00 AM	13.2	13.4	14.0	13.5
2022-09-08 4:00 AM	12.9	13.1	13.5	13.2
2022-09-08 5:00 AM	12.6	12.8	12.9	12.8
2022-09-08 6:00 AM	12.3	12.5	12.8	12.5

2022-09-08 7:00 AM	12.4	12.6	12.8	12.6
2022-09-08 8:00 AM	12.6	12.7	12.7	12.7
2022-09-08 9:00 AM	13.1	13.2	13.3	13.2
2022-09-08 10:00 AM	14.5	14.3	14.5	14.4
2022-09-08 11:00 AM	16.1	15.4	15.4	15.6
2022-09-08 12:00 PM	17.7	16.4	16.7	16.9
2022-09-08 1:00 PM	18.1	16.9	17.9	17.6
2022-09-08 2:00 PM	18.4	17.4	18.4	18.1
2022-09-08 3:00 PM	18.2	17.4	18.2	17.9
2022-09-08 4:00 PM	18.6	17.8	18.7	18.4
2022-09-08 5:00 PM	18.5	17.7	18.5	18.2
2022-09-08 6:00 PM	18.4	17.6	18.3	18.1
2022-09-08 7:00 PM	18.2	17.5	18.0	17.9
2022-09-08 8:00 PM	18.0	17.4	17.8	17.7
2022-09-08 9:00 PM	17.7	17.2	17.6	17.5
2022-09-08 10:00 PM	17.7	17.2	17.6	17.5
2022-09-08 11:00 PM	17.7	17.2	17.6	17.5
2022-09-09 12:00 AM	17.7	17.3	17.6	17.5
2022-09-09 1:00 AM	17.6	17.2	17.6	17.5
2022-09-09 2:00 AM	17.6	17.2	17.6	17.5
2022-09-09 3:00 AM	17.6	17.3	17.6	17.5
2022-09-09 4:00 AM	17.7	17.3	17.7	17.6
2022-09-09 5:00 AM	17.5	17.2	17.5	17.4
2022-09-09 6:00 AM	17.4	17.2	17.5	17.4
2022-09-09 7:00 AM	17.3	17.1	17.4	17.3
2022-09-09 8:00 AM	17.1	16.9	17.1	17.0
2022-09-09 9:00 AM	16.9	16.8	17.0	16.9
2022-09-09 10:00 AM	16.8	16.7	16.9	16.8
2022-09-09 11:00 AM	16.7	16.7	16.8	16.7
2022-09-09 12:00 PM	16.5	16.4	16.6	16.5
2022-09-09 1:00 PM	16.2	16.2	16.3	16.2
2022-09-09 2:00 PM	16.1	16.2	16.2	16.2
2022-09-09 3:00 PM	16.3	16.3	16.2	16.3
2022-09-09 4:00 PM	16.4	16.4	16.2	16.3
2022-09-09 5:00 PM	16.4	16.3	16.2	16.3
2022-09-09 6:00 PM	16.2	16.1	16.1	16.1
2022-09-09 7:00 PM	16.2	16.1	16.1	16.1
2022-09-09 8:00 PM	16.0	15.9	15.9	15.9
2022-09-09 9:00 PM	15.7	15.7	15.7	15.7
2022-09-09 10:00 PM	15.5	15.6	15.6	15.6
2022-09-09 11:00 PM	15.5	15.5	15.5	15.5
2022-09-10 12:00 AM	15.4	15.4	15.4	15.4
2022-09-10 1:00 AM	15.3	15.3	15.3	15.3
2022-09-10 2:00 AM	15.2	15.2	15.3	15.2
2022-09-10 3:00 AM	15.2	15.2	15.2	15.2

2022-09-10 4:00 AM	14.9	15.0	15.0	15.0
2022-09-10 5:00 AM	14.8	15.0	15.0	14.9
2022-09-10 6:00 AM	14.4	14.5	14.6	14.5
2022-09-10 7:00 AM	14.3	14.5	14.5	14.4
2022-09-10 8:00 AM	14.0	14.2	14.3	14.2
2022-09-10 9:00 AM	14.3	14.4	14.3	14.3
2022-09-10 10:00 AM	14.9	14.9	14.9	14.9
2022-09-10 11:00 AM	15.4	15.3	15.3	15.3
2022-09-10 12:00 PM	15.9	15.6	15.7	15.7
2022-09-10 1:00 PM	16.5	16.0	16.3	16.3
2022-09-10 2:00 PM	16.8	16.2	16.6	16.5
2022-09-10 3:00 PM	17.0	16.4	17.2	16.9
2022-09-10 4:00 PM	16.8	16.4	17.7	17.0
2022-09-10 5:00 PM	16.8	16.5	18.3	17.2
2022-09-10 6:00 PM	16.4	16.5	18.8	17.2
2022-09-10 7:00 PM	16.2	16.1	17.1	16.5
2022-09-10 8:00 PM	15.7	15.7	16.3	15.9
2022-09-10 9:00 PM	15.1	15.2	15.7	15.3
2022-09-10 10:00 PM	14.7	14.7	15.2	14.9
2022-09-10 11:00 PM	14.3	14.3	14.7	14.4
2022-09-11 12:00 AM	13.9	14.1	14.2	14.1
2022-09-11 1:00 AM	13.5	13.8	13.9	13.7
2022-09-11 2:00 AM	13.2	13.4	13.6	13.4
2022-09-11 3:00 AM	12.7	12.9	12.9	12.8
2022-09-11 4:00 AM	12.3	12.5	12.4	12.4
2022-09-11 5:00 AM	11.9	12.1	12.1	12.0
2022-09-11 6:00 AM	11.7	11.9	11.8	11.8
2022-09-11 7:00 AM	11.3	11.6	11.4	11.4
2022-09-11 8:00 AM	11.1	11.5	11.3	11.3
2022-09-11 9:00 AM	11.2	11.5	11.4	11.4
2022-09-11 10:00 AM	12.1	12.4	12.3	12.3
2022-09-11 11:00 AM	14.2	13.8	13.4	13.8
2022-09-11 12:00 PM	15.9	14.7	14.8	15.1
2022-09-11 1:00 PM	16.2	15.0	15.9	15.7
2022-09-11 2:00 PM	16.5	15.4	18.0	16.6
2022-09-11 3:00 PM	16.4	15.4	18.6	16.8
2022-09-11 4:00 PM	16.0	15.5	17.3	16.3
2022-09-11 5:00 PM	16.1	15.7	19.5	17.1
2022-09-11 6:00 PM	16.2	16.0	19.1	17.1
2022-09-11 7:00 PM	15.9	15.6	17.1	16.2
2022-09-11 8:00 PM	15.5	15.2	16.3	15.7
2022-09-11 9:00 PM	14.9	14.7	15.6	15.1
2022-09-11 10:00 PM	14.5	14.4	15.1	14.7
2022-09-11 11:00 PM	14.0	14.0	14.5	14.2
2022-09-12 12:00 AM	13.5	13.6	13.9	13.7

2022-09-12 1:00 AM	13.0	13.1	13.4	13.2
2022-09-12 2:00 AM	12.6	12.8	12.8	12.7
2022-09-12 3:00 AM	12.1	12.3	12.2	12.2
2022-09-12 4:00 AM	11.7	11.9	11.9	11.8
2022-09-12 5:00 AM	11.5	11.7	11.7	11.6
2022-09-12 6:00 AM	11.3	11.5	11.4	11.4
2022-09-12 7:00 AM	11.0	11.2	11.2	11.1
2022-09-12 8:00 AM	10.7	11.0	11.0	10.9
2022-09-12 9:00 AM	10.9	11.2	11.1	11.1
2022-09-12 10:00 AM	12.1	12.2	12.1	12.1
2022-09-12 11:00 AM	14.0	13.5	13.1	13.5
2022-09-12 12:00 PM	15.4	14.3	14.5	14.7
2022-09-12 1:00 PM	15.9	14.6	16.1	15.5
2022-09-12 2:00 PM	16.5	15.3	18.5	16.8
2022-09-12 3:00 PM	16.1	15.2	18.7	16.7
2022-09-12 4:00 PM	16.0	15.3	17.1	16.1
2022-09-12 5:00 PM	16.3	15.7	19.2	17.1
2022-09-12 6:00 PM	16.4	16.0	19.0	17.1
2022-09-12 7:00 PM	16.0	15.7	17.3	16.3
2022-09-12 8:00 PM	15.7	15.4	16.6	15.9
2022-09-12 9:00 PM	15.3	15.1	16.0	15.5
2022-09-12 10:00 PM	14.8	14.6	15.4	14.9
2022-09-12 11:00 PM	14.4	14.3	14.9	14.5
2022-09-13 12:00 AM	14.1	14.0	14.5	14.2
2022-09-13 1:00 AM	13.7	13.7	14.2	13.9
2022-09-13 2:00 AM	13.3	13.4	13.8	13.5
2022-09-13 3:00 AM	12.9	13.1	13.3	13.1
2022-09-13 4:00 AM	12.5	12.7	12.8	12.7
2022-09-13 5:00 AM	12.4	12.5	12.6	12.5
2022-09-13 6:00 AM	12.0	12.2	12.4	12.2
2022-09-13 7:00 AM	12.1	12.2	12.3	12.2
2022-09-13 8:00 AM	11.9	12.2	12.2	12.1
2022-09-13 9:00 AM	12.6	12.7	12.8	12.7
2022-09-13 10:00 AM	13.5	13.5	13.5	13.5
2022-09-13 11:00 AM	15.4	14.8	14.2	14.8
2022-09-13 12:00 PM	16.2	15.2	14.9	15.4
2022-09-13 1:00 PM	16.1	15.1	15.6	15.6
2022-09-13 2:00 PM	16.1	15.2	16.6	16.0
2022-09-13 3:00 PM	15.8	15.0	16.6	15.8
2022-09-13 4:00 PM	15.6	15.0	15.9	15.5
2022-09-13 5:00 PM	15.4	14.9	15.4	15.2
2022-09-13 6:00 PM	15.1	14.6	14.9	14.9
2022-09-13 7:00 PM	14.9	14.5	14.7	14.7
2022-09-13 8:00 PM	14.5	14.3	14.4	14.4
2022-09-13 9:00 PM	13.7	13.6	13.8	13.7

2022-09-13 10:00 PM	13.3	13.4	13.4	13.4
2022-09-13 11:00 PM	12.9	13.3	12.7	13.0
2022-09-14 12:00 AM	12.4	12.7	12.4	12.5
2022-09-14 1:00 AM	11.7	11.9	11.8	11.8
2022-09-14 2:00 AM	11.3	11.5	11.4	11.4
2022-09-14 3:00 AM	11.1	11.3	11.0	11.1
2022-09-14 4:00 AM	10.7	11.1	11.0	10.9
2022-09-14 5:00 AM	10.2	10.6	10.3	10.4
2022-09-14 6:00 AM	9.8	10.2	9.9	10.0
2022-09-14 7:00 AM	9.2	9.8	9.5	9.5
2022-09-14 8:00 AM	8.9	9.6	9.2	9.2
2022-09-14 9:00 AM	9.2	9.7	9.2	9.4
2022-09-14 10:00 AM	10.2	10.5	10.2	10.3
2022-09-14 11:00 AM	12.9	12.3	11.4	12.2
2022-09-14 12:00 PM	14.3	13.0	12.5	13.3
2022-09-14 1:00 PM	14.7	13.2	13.6	13.8
2022-09-14 2:00 PM	14.8	13.5	14.8	14.4
2022-09-14 3:00 PM	14.1	13.3	15.2	14.2
2022-09-14 4:00 PM	13.7	13.2	14.1	13.7
2022-09-14 5:00 PM	13.7	13.5	17.1	14.8
2022-09-14 6:00 PM	13.6	13.5	16.1	14.4
2022-09-14 7:00 PM	13.1	13.1	14.0	13.4
2022-09-14 8:00 PM	13.0	13.0	13.5	13.2
2022-09-14 9:00 PM	12.6	12.6	12.9	12.7
2022-09-14 10:00 PM	12.0	12.0	12.2	12.1
2022-09-14 11:00 PM	11.5	11.7	12.0	11.7
2022-09-15 12:00 AM	11.2	11.3	11.5	11.3
2022-09-15 1:00 AM	11.1	11.4	11.2	11.2
2022-09-15 2:00 AM	11.3	11.7	11.5	11.5
2022-09-15 3:00 AM	11.6	12.0	11.8	11.8
2022-09-15 4:00 AM	11.9	12.1	12.0	12.0
2022-09-15 5:00 AM	12.1	12.2	12.2	12.2
2022-09-15 6:00 AM	12.2	12.4	12.3	12.3
2022-09-15 7:00 AM	12.3	12.4	12.4	12.4
2022-09-15 8:00 AM	12.3	12.4	12.4	12.4
2022-09-15 9:00 AM	12.6	12.6	12.7	12.6
2022-09-15 10:00 AM	12.7	12.6	12.7	12.7
2022-09-15 11:00 AM	12.8	12.8	12.9	12.8
2022-09-15 12:00 PM	13.0	12.9	13.1	13.0
2022-09-15 1:00 PM	13.3	13.1	13.3	13.2
2022-09-15 2:00 PM	13.4	13.2	13.5	13.4
2022-09-15 3:00 PM	13.6	13.4	13.6	13.5
2022-09-15 4:00 PM	13.7	13.5	13.8	13.7
2022-09-15 5:00 PM	13.7	13.5	13.8	13.7
2022-09-15 6:00 PM	13.9	13.6	14.0	13.8

2022-09-15 7:00 PM	13.9	13.6	14.0	13.8
2022-09-15 8:00 PM	13.9	13.6	14.0	13.8
2022-09-15 9:00 PM	13.9	13.6	13.9	13.8
2022-09-15 10:00 PM	13.9	13.7	13.9	13.8
2022-09-15 11:00 PM	13.9	13.7	14.0	13.9
2022-09-16 12:00 AM	13.9	13.7	13.9	13.8
2022-09-16 1:00 AM	13.8	13.7	13.9	13.8
2022-09-16 2:00 AM	13.8	13.7	13.9	13.8
2022-09-16 3:00 AM	13.7	13.6	13.7	13.7
2022-09-16 4:00 AM	13.5	13.5	13.5	13.5
2022-09-16 5:00 AM	13.3	13.4	13.3	13.3
2022-09-16 6:00 AM	13.3	13.3	13.2	13.3
2022-09-16 7:00 AM	13.1	13.3	13.1	13.2
2022-09-16 8:00 AM	12.9	13.1	12.8	12.9
2022-09-16 9:00 AM	12.9	13.1	12.7	12.9
2022-09-16 10:00 AM	13.0	13.1	12.8	13.0
2022-09-16 11:00 AM	13.1	13.2	12.9	13.1
2022-09-16 12:00 PM	13.2	13.2	13.0	13.1
2022-09-16 1:00 PM	13.3	13.3	13.1	13.2
2022-09-16 2:00 PM	13.4	13.4	13.2	13.3
2022-09-16 3:00 PM	13.4	13.3	13.2	13.3
2022-09-16 4:00 PM	13.4	13.3	13.2	13.3
2022-09-16 5:00 PM	13.4	13.3	13.2	13.3
2022-09-16 6:00 PM	13.4	13.3	13.2	13.3
2022-09-16 7:00 PM	13.4	13.3	13.3	13.3
2022-09-16 8:00 PM	13.3	13.3	13.3	13.3
2022-09-16 9:00 PM	13.4	13.3	13.4	13.4
2022-09-16 10:00 PM	13.5	13.4	13.5	13.5
2022-09-16 11:00 PM	13.6	13.5	13.6	13.6
2022-09-17 12:00 AM	13.7	13.5	13.7	13.6
2022-09-17 1:00 AM	13.7	13.6	13.9	13.7
2022-09-17 2:00 AM	13.9	13.7	13.9	13.8
2022-09-17 3:00 AM	13.9	13.7	14.0	13.9
2022-09-17 4:00 AM	13.9	13.7	14.0	13.9
2022-09-17 5:00 AM	14.0	13.8	14.0	13.9
2022-09-17 6:00 AM	14.0	13.8	14.0	13.9
2022-09-17 7:00 AM	14.0	13.9	14.0	14.0
2022-09-17 8:00 AM	14.0	13.9	14.1	14.0
2022-09-17 9:00 AM	14.2	14.0	14.2	14.1
2022-09-17 10:00 AM	14.4	14.2	14.5	14.4
2022-09-17 11:00 AM	14.7	14.4	14.7	14.6
2022-09-17 12:00 PM	15.0	14.6	15.0	14.9
2022-09-17 1:00 PM	15.4	14.9	15.4	15.2
2022-09-17 2:00 PM	15.8	15.2	15.7	15.6
2022-09-17 3:00 PM	15.9	15.3	15.8	15.7

2022-09-17 4:00 PM	15.8	15.3	15.8	15.6
2022-09-17 5:00 PM	15.8	15.2	15.7	15.6
2022-09-17 6:00 PM	15.7	15.2	15.7	15.5
2022-09-17 7:00 PM	15.6	15.2	15.6	15.5
2022-09-17 8:00 PM	15.5	15.1	15.4	15.3
2022-09-17 9:00 PM	15.4	15.1	15.4	15.3
2022-09-17 10:00 PM	15.4	15.1	15.4	15.3
2022-09-17 11:00 PM	15.4	15.1	15.4	15.3
2022-09-18 12:00 AM	15.4	15.1	15.4	15.3
2022-09-18 1:00 AM	15.4	15.1	15.4	15.3
2022-09-18 2:00 AM	15.4	15.1	15.4	15.3
2022-09-18 3:00 AM	15.4	15.1	15.4	15.3
2022-09-18 4:00 AM	15.3	15.1	15.4	15.3
2022-09-18 5:00 AM	15.3	15.1	15.4	15.3
2022-09-18 6:00 AM	15.3	15.1	15.4	15.3
2022-09-18 7:00 AM	15.3	15.1	15.4	15.3
2022-09-18 8:00 AM	15.3	15.1	15.3	15.2
2022-09-18 9:00 AM	15.3	15.1	15.3	15.2
2022-09-18 10:00 AM	15.3	15.2	15.4	15.3
2022-09-18 11:00 AM	15.7	15.3	15.6	15.5
2022-09-18 12:00 PM	15.8	15.5	15.8	15.7
2022-09-18 1:00 PM	16.5	15.9	16.5	16.3
2022-09-18 2:00 PM	17.2	16.4	17.2	16.9
2022-09-18 3:00 PM	17.3	16.5	17.4	17.1
2022-09-18 4:00 PM	17.4	16.6	17.6	17.2
2022-09-18 5:00 PM	17.2	16.6	17.2	17.0
2022-09-18 6:00 PM	17.3	16.7	17.2	17.1
2022-09-18 7:00 PM	17.2	16.7	17.0	17.0
2022-09-18 8:00 PM	16.8	16.4	16.7	16.6
2022-09-18 9:00 PM	16.5	16.1	16.4	16.3
2022-09-18 10:00 PM	16.0	15.8	16.0	15.9
2022-09-18 11:00 PM	15.4	15.4	15.4	15.4
2022-09-19 12:00 AM	15.0	15.0	15.0	15.0
2022-09-19 1:00 AM	14.7	14.7	14.7	14.7
2022-09-19 2:00 AM	14.4	14.5	14.3	14.4
2022-09-19 3:00 AM	14.1	14.3	14.1	14.2
2022-09-19 4:00 AM	13.9	14.1	13.9	14.0
2022-09-19 5:00 AM	14.0	14.1	13.8	14.0
2022-09-19 6:00 AM	13.7	13.9	13.6	13.7
2022-09-19 7:00 AM	13.5	13.7	13.5	13.6
2022-09-19 8:00 AM	13.4	13.6	13.3	13.4
2022-09-19 9:00 AM	13.5	13.7	13.4	13.5
2022-09-19 10:00 AM	14.3	14.3	14.1	14.2
2022-09-19 11:00 AM	16.2	15.4	14.9	15.5
2022-09-19 12:00 PM	17.3	16.0	15.8	16.4

2022-09-19 1:00 PM	17.2	16.1	16.8	16.7
2022-09-19 2:00 PM	17.4	16.3	20.0	17.9
2022-09-19 3:00 PM	17.2	16.4	19.5	17.7
2022-09-19 4:00 PM	17.3	16.5	18.3	17.4
2022-09-19 5:00 PM	17.6	16.8	19.1	17.8
2022-09-19 6:00 PM	17.3	16.7	18.3	17.4
2022-09-19 7:00 PM	16.7	16.3	17.3	16.8
2022-09-19 8:00 PM	16.4	16.1	16.8	16.4
2022-09-19 9:00 PM	15.9	15.8	16.2	16.0
2022-09-19 10:00 PM	15.6	15.5	15.9	15.7
2022-09-19 11:00 PM	15.4	15.4	15.7	15.5
2022-09-20 12:00 AM	15.5	15.4	15.7	15.5
2022-09-20 1:00 AM	15.5	15.5	15.7	15.6
2022-09-20 2:00 AM	15.5	15.4	15.6	15.5
2022-09-20 3:00 AM	15.3	15.3	15.5	15.4
2022-09-20 4:00 AM	15.4	15.4	15.6	15.5
2022-09-20 5:00 AM	15.3	15.4	15.5	15.4
2022-09-20 6:00 AM	15.1	15.2	15.3	15.2
2022-09-20 7:00 AM	15.3	15.3	15.5	15.4
2022-09-20 8:00 AM	15.4	15.4	15.5	15.4
2022-09-20 9:00 AM	15.5	15.4	15.6	15.5
2022-09-20 10:00 AM	15.8	15.6	15.8	15.7
2022-09-20 11:00 AM	16.3	15.9	16.3	16.2
2022-09-20 12:00 PM	17.2	16.3	16.8	16.8
2022-09-20 1:00 PM	17.5	16.4	17.7	17.2
2022-09-20 2:00 PM	17.7	16.8	20.8	18.4
2022-09-20 3:00 PM	17.6	16.8	20.2	18.2
2022-09-20 4:00 PM	17.8	17.0	18.8	17.9
2022-09-20 5:00 PM	17.8	17.1	18.3	17.7
2022-09-20 6:00 PM	17.4	16.9	17.8	17.4
2022-09-20 7:00 PM	17.5	16.9	17.8	17.4
2022-09-20 8:00 PM	17.3	16.9	17.7	17.3
2022-09-20 9:00 PM	17.0	16.7	17.3	17.0
2022-09-20 10:00 PM	16.5	16.4	16.8	16.6
2022-09-20 11:00 PM	16.6	16.4	16.8	16.6
2022-09-21 12:00 AM	16.6	16.4	16.8	16.6
2022-09-21 1:00 AM	16.4	16.3	16.7	16.5
2022-09-21 2:00 AM	16.3	16.2	16.5	16.3
2022-09-21 3:00 AM	16.0	16.0	16.3	16.1
2022-09-21 4:00 AM	15.8	15.9	16.0	15.9
2022-09-21 5:00 AM	15.6	15.7	15.8	15.7
2022-09-21 6:00 AM	15.4	15.5	15.5	15.5
2022-09-21 7:00 AM	14.6	15.0	14.9	14.8
2022-09-21 8:00 AM	14.2	14.6	14.5	14.4
2022-09-21 9:00 AM	14.2	14.6	14.4	14.4

2022-09-21 10:00 AM	15.0	14.9	14.8	14.9
2022-09-21 11:00 AM	15.2	15.2	14.9	15.1
2022-09-21 12:00 PM	15.1	15.0	14.7	14.9
2022-09-21 1:00 PM	14.4	14.3	14.2	14.3
2022-09-21 2:00 PM	14.8	14.6	15.6	15.0
2022-09-21 3:00 PM	14.1	14.1	14.1	14.1
2022-09-21 4:00 PM	13.5	13.7	13.7	13.6
2022-09-21 5:00 PM	13.5	13.5	14.6	13.9
2022-09-21 6:00 PM	12.9	13.0	13.0	13.0
2022-09-21 7:00 PM	12.6	12.8	12.3	12.6
2022-09-21 8:00 PM	12.4	12.7	12.1	12.4
2022-09-21 9:00 PM	12.1	12.5	11.7	12.1
2022-09-21 10:00 PM	12.2	12.5	11.9	12.2
2022-09-21 11:00 PM	12.1	12.4	11.9	12.1
2022-09-22 12:00 AM	12.0	12.3	11.8	12.0
2022-09-22 1:00 AM	11.5	12.0	11.4	11.6
2022-09-22 2:00 AM	11.0	11.7	11.0	11.2
2022-09-22 3:00 AM	10.9	11.5	10.9	11.1
2022-09-22 4:00 AM	10.6	11.3	10.5	10.8
2022-09-22 5:00 AM	10.5	11.2	10.5	10.7
2022-09-22 6:00 AM	10.7	11.2	10.6	10.8
2022-09-22 7:00 AM	10.5	11.1	10.6	10.7
2022-09-22 8:00 AM	9.7	10.6	9.6	10.0
2022-09-22 9:00 AM	9.7	10.5	9.6	9.9
2022-09-22 10:00 AM	9.4	10.3	8.9	9.5
2022-09-22 11:00 AM	10.6	10.7	8.9	10.1
2022-09-22 12:00 PM	11.4	11.2	9.9	10.8
2022-09-22 1:00 PM	11.5	11.3	10.9	11.2
2022-09-22 2:00 PM	11.5	11.5	14.0	12.3
2022-09-22 3:00 PM	11.3	11.4	13.2	12.0
2022-09-22 4:00 PM	11.3	11.4	11.8	11.5
2022-09-22 5:00 PM	11.5	11.6	15.2	12.8
2022-09-22 6:00 PM	11.7	11.8	14.0	12.5
2022-09-22 7:00 PM	11.5	11.6	12.5	11.9
2022-09-22 8:00 PM	11.0	11.3	11.7	11.3
2022-09-22 9:00 PM	10.5	11.0	10.9	10.8
2022-09-22 10:00 PM	10.1	10.6	10.5	10.4
2022-09-22 11:00 PM	9.8	10.3	10.1	10.1
2022-09-23 12:00 AM	9.4	9.9	9.5	9.6
2022-09-23 1:00 AM	8.9	9.6	9.1	9.2
2022-09-23 2:00 AM	8.7	9.2	8.8	8.9
2022-09-23 3:00 AM	8.6	9.0	8.6	8.7
2022-09-23 4:00 AM	8.3	8.8	8.4	8.5
2022-09-23 5:00 AM	8.1	8.7	8.2	8.3
2022-09-23 6:00 AM	7.8	8.6	7.8	8.1

2022-09-23 7:00 AM	7.4	8.3	7.5	7.7
2022-09-23 8:00 AM	6.9	7.9	7.1	7.3
2022-09-23 9:00 AM	7.1	8.0	7.0	7.4
2022-09-23 10:00 AM	8.2	8.8	8.1	8.4
2022-09-23 11:00 AM	11.0	10.4	9.6	10.3
2022-09-23 12:00 PM	11.8	11.0	11.0	11.3
2022-09-23 1:00 PM	12.2	11.4	12.0	11.9
2022-09-23 2:00 PM	12.5	11.8	12.8	12.4

Table S6: Hourly air temperature under 80% shade from May 10 to September 23, 2023.

	Port 1	Port 2	Port 3	Average
	5TE Moisture/ Temp/EC	5TE Moisture/ Temp/EC	5TE Moisture/ Temp/EC	5TE Moisture/ Temp/EC
Measurement Time	°C Temp	°C Temp	°C Temp	°C Temp
2022-06-22 5:00 PM	20.9	21.1	21.1	21.0
2022-06-22 6:00 PM	21.0	20.2	20.2	20.6
2022-06-22 7:00 PM	19.1	18.9	18.9	19.0
2022-06-22 8:00 PM	18.3	18.2	18.2	18.3
2022-06-22 9:00 PM	17.7	17.6	17.6	17.7
2022-06-22 10:00 PM	16.8	16.7	16.7	16.8
2022-06-22 11:00 PM	15.6	15.7	15.7	15.7
2022-06-23 12:00 AM	14.8	15.1	15.1	15.0
2022-06-23 1:00 AM	14.3	14.6	14.6	14.5
2022-06-23 2:00 AM	13.5	14.0	14.0	13.8
2022-06-23 3:00 AM	13.2	13.6	13.6	13.4
2022-06-23 4:00 AM	12.5	13.0	13.0	12.8
2022-06-23 5:00 AM	12.2	12.7	12.7	12.4
2022-06-23 6:00 AM	12.2	12.5	12.5	12.3
2022-06-23 7:00 AM	12.0	12.3	12.3	12.2
2022-06-23 8:00 AM	12.4	12.6	12.6	12.5
2022-06-23 9:00 AM	12.9	13.0	13.0	12.9
2022-06-23 10:00 AM	13.9	13.7	13.7	13.8
2022-06-23 11:00 AM	16.0	15.2	15.2	15.6
2022-06-23 12:00 PM	21.4	19.8	19.8	20.6
2022-06-23 1:00 PM	22.5	21.0	21.0	21.8
2022-06-23 2:00 PM	23.5	21.7	21.7	22.6
2022-06-23 3:00 PM	23.5	22.7	22.7	23.1
2022-06-23 4:00 PM	23.0	23.0	23.0	23.0
2022-06-23 5:00 PM	23.0	23.2	23.2	23.1

2022-06-23 6:00 PM	22.2	21.2	21.2	21.7
2022-06-23 7:00 PM	20.4	20.0	20.0	20.2
2022-06-23 8:00 PM	20.0	19.6	19.6	19.8
2022-06-23 9:00 PM	18.9	18.7	18.7	18.8
2022-06-23 10:00 PM	18.3	18.2	18.2	18.3
2022-06-23 11:00 PM	17.6	17.6	17.6	17.6
2022-06-24 12:00 AM	16.9	17.0	17.0	16.9
2022-06-24 1:00 AM	15.8	15.8	15.8	15.8
2022-06-24 2:00 AM	15.4	15.5	15.5	15.4
2022-06-24 3:00 AM	15.1	15.2	15.2	15.2
2022-06-24 4:00 AM	13.9	14.0	14.0	13.9
2022-06-24 5:00 AM	13.4	13.6	13.6	13.5
2022-06-24 6:00 AM	13.2	13.4	13.4	13.3
2022-06-24 7:00 AM	13.2	13.4	13.4	13.3
2022-06-24 8:00 AM	14.0	14.0	14.0	14.0
2022-06-24 9:00 AM	15.2	15.0	15.0	15.1
2022-06-24 10:00 AM	15.8	15.4	15.4	15.6
2022-06-24 11:00 AM	16.7	16.0	16.0	16.4
2022-06-24 12:00 PM	20.7	19.6	19.6	20.2
2022-06-24 1:00 PM	22.3	21.5	21.5	21.9
2022-06-24 2:00 PM	23.4	22.2	22.2	22.8
2022-06-24 3:00 PM	22.6	22.4	22.4	22.5
2022-06-24 4:00 PM	21.3	21.7	21.7	21.5
2022-06-24 5:00 PM	20.2	20.8	20.8	20.5
2022-06-24 6:00 PM	19.9	19.3	19.3	19.6
2022-06-24 7:00 PM	18.2	18.1	18.1	18.2
2022-06-24 8:00 PM	17.6	17.6	17.6	17.6
2022-06-24 9:00 PM	17.3	17.3	17.3	17.3
2022-06-24 10:00 PM	16.7	16.8	16.8	16.8
2022-06-24 11:00 PM	16.1	16.3	16.3	16.2
2022-06-25 12:00 AM	15.6	15.8	15.8	15.7
2022-06-25 1:00 AM	15.2	15.4	15.4	15.3
2022-06-25 2:00 AM	15.0	15.3	15.3	15.2
2022-06-25 3:00 AM	15.2	15.3	15.3	15.3
2022-06-25 4:00 AM	15.2	15.3	15.3	15.3
2022-06-25 5:00 AM	14.8	14.9	14.9	14.8
2022-06-25 6:00 AM	14.5	14.6	14.6	14.6
2022-06-25 7:00 AM	14.3	14.3	14.3	14.3
2022-06-25 8:00 AM	14.2	14.2	14.2	14.2
2022-06-25 9:00 AM	14.5	14.4	14.4	14.4
2022-06-25 10:00 AM	15.1	14.8	14.8	15.0
2022-06-25 11:00 AM	15.4	15.1	15.1	15.3
2022-06-25 12:00 PM	15.1	14.9	14.9	15.0

2022-06-25 1:00 PM	15.2	15.0	15.0	15.1
2022-06-25 2:00 PM	16.2	15.6	15.6	15.9
2022-06-25 3:00 PM	18.7	18.2	18.2	18.5
2022-06-25 4:00 PM	18.4	18.6	18.6	18.5
2022-06-25 5:00 PM	17.2	17.1	17.1	17.2
2022-06-25 6:00 PM	17.4	16.9	16.9	17.1
2022-06-25 7:00 PM	16.8	16.7	16.7	16.8
2022-06-25 8:00 PM	16.2	16.1	16.1	16.2
2022-06-25 9:00 PM	16.0	15.9	15.9	15.9
2022-06-25 10:00 PM	15.2	15.3	15.3	15.3
2022-06-25 11:00 PM	14.7	14.9	14.9	14.8
2022-06-26 12:00 AM	14.3	14.5	14.5	14.4
2022-06-26 1:00 AM	14.1	14.3	14.3	14.2
2022-06-26 2:00 AM	14.0	14.2	14.2	14.1
2022-06-26 3:00 AM	14.3	14.4	14.4	14.3
2022-06-26 4:00 AM	13.8	14.0	14.0	13.9
2022-06-26 5:00 AM	13.3	13.6	13.6	13.5
2022-06-26 6:00 AM	12.6	13.0	13.0	12.8
2022-06-26 7:00 AM	12.4	12.7	12.7	12.5
2022-06-26 8:00 AM	12.7	12.8	12.8	12.8
2022-06-26 9:00 AM	13.4	13.5	13.5	13.4
2022-06-26 10:00 AM	13.8	13.7	13.7	13.8
2022-06-26 11:00 AM	15.1	14.6	14.6	14.9
2022-06-26 12:00 PM	16.6	15.9	15.9	16.3
2022-06-26 1:00 PM	16.8	16.3	16.3	16.5
2022-06-26 2:00 PM	16.5	16.2	16.2	16.4
2022-06-26 3:00 PM	16.2	16.0	16.0	16.1
2022-06-26 4:00 PM	15.7	16.4	16.4	16.0
2022-06-26 5:00 PM	15.7	16.5	16.5	16.1
2022-06-26 6:00 PM	15.1	15.4	15.4	15.3
2022-06-26 7:00 PM	14.0	14.4	14.4	14.2
2022-06-26 8:00 PM	13.7	14.1	14.1	13.9
2022-06-26 9:00 PM	13.3	13.7	13.7	13.5
2022-06-26 10:00 PM	12.9	13.1	13.1	13.0
2022-06-26 11:00 PM	12.4	12.6	12.6	12.5
2022-06-27 12:00 AM	12.2	12.3	12.3	12.3
2022-06-27 1:00 AM	11.9	12.0	12.0	11.9
2022-06-27 2:00 AM	11.9	12.0	12.0	11.9
2022-06-27 3:00 AM	11.7	11.7	11.7	11.7
2022-06-27 4:00 AM	11.5	11.4	11.4	11.4
2022-06-27 5:00 AM	11.4	11.4	11.4	11.4
2022-06-27 6:00 AM	10.9	11.0	11.0	10.9
2022-06-27 7:00 AM	10.0	10.3	10.3	10.2

2022-06-27 8:00 AM	10.1	10.4	10.4	10.3
2022-06-27 9:00 AM	10.9	11.0	11.0	10.9
2022-06-27 10:00 AM	12.2	12.0	12.0	12.1
2022-06-27 11:00 AM	14.3	13.5	13.5	13.9
2022-06-27 12:00 PM	19.4	18.0	18.0	18.7
2022-06-27 1:00 PM	21.2	20.2	20.2	20.7
2022-06-27 2:00 PM	20.4	18.9	18.9	19.6
2022-06-27 3:00 PM	19.2	18.2	18.2	18.7
2022-06-27 4:00 PM	18.7	18.3	18.3	18.5
2022-06-27 5:00 PM	18.7	18.9	18.9	18.8
2022-06-27 6:00 PM	19.2	18.6	18.6	18.9
2022-06-27 7:00 PM	17.1	17.2	17.2	17.2
2022-06-27 8:00 PM	16.5	16.6	16.6	16.6
2022-06-27 9:00 PM	16.0	16.0	16.0	16.0
2022-06-27 10:00 PM	15.0	15.2	15.2	15.1
2022-06-27 11:00 PM	13.6	14.0	14.0	13.8
2022-06-28 12:00 AM	12.9	13.3	13.3	13.1
2022-06-28 1:00 AM	12.9	13.2	13.2	13.0
2022-06-28 2:00 AM	14.2	14.4	14.4	14.3
2022-06-28 3:00 AM	14.4	14.6	14.6	14.5
2022-06-28 4:00 AM	14.7	14.8	14.8	14.8
2022-06-28 5:00 AM	14.5	14.6	14.6	14.6
2022-06-28 6:00 AM	14.3	14.4	14.4	14.3
2022-06-28 7:00 AM	14.2	14.3	14.3	14.3
2022-06-28 8:00 AM	14.4	14.4	14.4	14.4
2022-06-28 9:00 AM	14.5	14.5	14.5	14.5
2022-06-28 10:00 AM	15.2	15.1	15.1	15.2
2022-06-28 11:00 AM	15.9	15.8	15.8	15.8
2022-06-28 12:00 PM	15.6	15.9	15.9	15.8
2022-06-28 1:00 PM	15.2	16.1	16.1	15.7
2022-06-28 2:00 PM	15.0	15.6	15.6	15.3
2022-06-28 3:00 PM	16.2	16.7	16.7	16.5
2022-06-28 4:00 PM	17.3	18.4	18.4	17.8
2022-06-28 5:00 PM	17.1	18.6	18.6	17.9
2022-06-28 6:00 PM	17.3	17.9	17.9	17.6
2022-06-28 7:00 PM	15.7	16.5	16.5	16.1
2022-06-28 8:00 PM	15.4	16.2	16.2	15.8
2022-06-28 9:00 PM	14.9	15.6	15.6	15.3
2022-06-28 10:00 PM	14.3	14.8	14.8	14.6
2022-06-28 11:00 PM	13.0	13.4	13.4	13.2
2022-06-29 12:00 AM	12.4	12.5	12.5	12.4
2022-06-29 1:00 AM	11.6	11.7	11.7	11.7
2022-06-29 2:00 AM	10.8	11.2	11.2	11.0

2022-06-29 3:00 AM	10.3	10.8	10.8	10.6
2022-06-29 4:00 AM	10.2	10.4	10.4	10.3
2022-06-29 5:00 AM	9.6	10.0	10.0	9.8
2022-06-29 6:00 AM	9.1	9.6	9.6	9.4
2022-06-29 7:00 AM	8.5	9.4	9.4	8.9
2022-06-29 8:00 AM	9.0	9.4	9.4	9.2
2022-06-29 9:00 AM	10.5	10.5	10.5	10.5
2022-06-29 10:00 AM	12.3	11.9	11.9	12.1
2022-06-29 11:00 AM	14.6	13.7	13.7	14.2
2022-06-29 12:00 PM	18.8	17.4	17.4	18.1
2022-06-29 1:00 PM	18.1	17.1	17.1	17.6
2022-06-29 2:00 PM	18.9	17.4	17.4	18.1
2022-06-29 3:00 PM	17.5	16.8	16.8	17.1
2022-06-29 4:00 PM	16.6	16.2	16.2	16.4
2022-06-29 5:00 PM	15.9	15.7	15.7	15.8
2022-06-29 6:00 PM	15.2	15.1	15.1	15.2
2022-06-29 7:00 PM	14.6	14.7	14.7	14.7
2022-06-29 8:00 PM	14.0	14.1	14.1	14.1
2022-06-29 9:00 PM	13.6	13.5	13.5	13.6
2022-06-29 10:00 PM	12.9	12.8	12.8	12.8
2022-06-29 11:00 PM	12.6	12.6	12.6	12.6
2022-06-30 12:00 AM	12.5	12.4	12.4	12.4
2022-06-30 1:00 AM	12.3	12.0	12.0	12.2
2022-06-30 2:00 AM	11.8	11.5	11.5	11.7
2022-06-30 3:00 AM	12.0	11.7	11.7	11.8
2022-06-30 4:00 AM	12.1	11.7	11.7	11.9
2022-06-30 5:00 AM	12.2	12.0	12.0	12.1
2022-06-30 6:00 AM	12.3	12.0	12.0	12.2
2022-06-30 7:00 AM	12.4	12.2	12.2	12.3
2022-06-30 8:00 AM	12.7	12.5	12.5	12.6
2022-06-30 9:00 AM	12.9	12.7	12.7	12.8
2022-06-30 10:00 AM	13.1	12.9	12.9	13.0
2022-06-30 11:00 AM	13.8	13.5	13.5	13.7
2022-06-30 12:00 PM	18.0	16.9	16.9	17.4
2022-06-30 1:00 PM	18.6	17.7	17.7	18.2
2022-06-30 2:00 PM	19.4	18.1	18.1	18.8
2022-06-30 3:00 PM	19.0	18.8	18.8	18.9
2022-06-30 4:00 PM	19.8	20.5	20.5	20.1
2022-06-30 5:00 PM	20.3	21.8	21.8	21.0
2022-06-30 6:00 PM	20.0	21.4	21.4	20.7
2022-06-30 7:00 PM	18.8	21.2	21.2	20.0
2022-06-30 8:00 PM	18.5	20.6	20.6	19.6
2022-06-30 9:00 PM	17.8	19.4	19.4	18.6

2022-06-30 10:00 PM	17.1	18.1	18.1	17.6
2022-06-30 11:00 PM	16.2	16.7	16.7	16.5
2022-07-01 12:00 AM	15.4	15.8	15.8	15.6
2022-07-01 1:00 AM	15.2	15.6	15.6	15.4
2022-07-01 2:00 AM	14.6	14.9	14.9	14.8
2022-07-01 3:00 AM	13.6	13.9	13.9	13.8
2022-07-01 4:00 AM	12.8	13.0	13.0	12.9
2022-07-01 5:00 AM	11.9	12.1	12.1	12.0
2022-07-01 6:00 AM	11.7	12.0	12.0	11.8
2022-07-01 7:00 AM	11.6	11.7	11.7	11.7
2022-07-01 8:00 AM	11.3	11.4	11.4	11.3
2022-07-01 9:00 AM	12.0	12.0	12.0	12.0
2022-07-01 10:00 AM	13.4	13.2	13.2	13.3
2022-07-01 11:00 AM	15.2	14.7	14.7	14.9
2022-07-01 12:00 PM	18.5	18.1	18.1	18.3
2022-07-01 1:00 PM	17.5	17.0	17.0	17.3
2022-07-01 2:00 PM	17.2	16.9	16.9	17.1
2022-07-01 3:00 PM	16.8	16.8	16.8	16.8
2022-07-01 4:00 PM	18.9	20.1	20.1	19.5
2022-07-01 5:00 PM	18.8	20.1	20.1	19.4
2022-07-01 6:00 PM	18.4	19.0	19.0	18.7
2022-07-01 7:00 PM	17.0	18.0	18.0	17.5
2022-07-01 8:00 PM	16.6	17.9	17.9	17.3
2022-07-01 9:00 PM	16.2	17.4	17.4	16.8
2022-07-01 10:00 PM	15.6	16.3	16.3	15.9
2022-07-01 11:00 PM	14.9	15.3	15.3	15.1
2022-07-02 12:00 AM	14.1	14.4	14.4	14.3
2022-07-02 1:00 AM	13.7	14.0	14.0	13.8
2022-07-02 2:00 AM	12.9	13.0	13.0	12.9
2022-07-02 3:00 AM	12.1	12.2	12.2	12.2
2022-07-02 4:00 AM	11.6	11.6	11.6	11.6
2022-07-02 5:00 AM	11.1	11.1	11.1	11.1
2022-07-02 6:00 AM	10.8	10.9	10.9	10.8
2022-07-02 7:00 AM	10.4	10.4	10.4	10.4
2022-07-02 8:00 AM	10.6	10.6	10.6	10.6
2022-07-02 9:00 AM	11.5	11.5	11.5	11.5
2022-07-02 10:00 AM	12.8	12.9	12.9	12.8
2022-07-02 11:00 AM	14.4	14.7	14.7	14.5
2022-07-02 12:00 PM	17.6	18.3	18.3	17.9
2022-07-02 1:00 PM	18.8	19.4	19.4	19.1
2022-07-02 2:00 PM	18.8	18.8	18.8	18.8
2022-07-02 3:00 PM	17.3	17.9	17.9	17.6
2022-07-02 4:00 PM	16.7	17.3	17.3	17.0

2022-07-02 5:00 PM	16.8	17.0	17.0	16.9
2022-07-02 6:00 PM	17.5	17.8	17.8	17.6
2022-07-02 7:00 PM	16.3	16.7	16.7	16.5
2022-07-02 8:00 PM	15.8	16.3	16.3	16.0
2022-07-02 9:00 PM	15.4	15.8	15.8	15.6
2022-07-02 10:00 PM	14.9	15.0	15.0	14.9
2022-07-02 11:00 PM	14.2	14.4	14.4	14.3
2022-07-03 12:00 AM	13.8	14.0	14.0	13.9
2022-07-03 1:00 AM	13.4	13.5	13.5	13.4
2022-07-03 2:00 AM	12.4	12.5	12.5	12.4
2022-07-03 3:00 AM	11.9	12.0	12.0	11.9
2022-07-03 4:00 AM	11.4	11.4	11.4	11.4
2022-07-03 5:00 AM	10.9	11.0	11.0	10.9
2022-07-03 6:00 AM	10.7	10.8	10.8	10.8
2022-07-03 7:00 AM	11.2	11.1	11.1	11.2
2022-07-03 8:00 AM	11.9	11.7	11.7	11.8
2022-07-03 9:00 AM	12.8	12.5	12.5	12.7
2022-07-03 10:00 AM	13.9	13.6	13.6	13.8
2022-07-03 11:00 AM	15.8	15.3	15.3	15.6
2022-07-03 12:00 PM	19.3	18.9	18.9	19.1
2022-07-03 1:00 PM	21.3	20.8	20.8	21.0
2022-07-03 2:00 PM	21.3	20.3	20.3	20.8
2022-07-03 3:00 PM	20.7	21.2	21.2	21.0
2022-07-03 4:00 PM	19.5	21.3	21.3	20.4
2022-07-03 5:00 PM	18.4	19.3	19.3	18.8
2022-07-03 6:00 PM	17.2	17.4	17.4	17.3
2022-07-03 7:00 PM	16.8	16.8	16.8	16.8
2022-07-03 8:00 PM	16.5	16.5	16.5	16.5
2022-07-03 9:00 PM	16.1	16.0	16.0	16.1
2022-07-03 10:00 PM	15.0	15.1	15.1	15.1
2022-07-03 11:00 PM	14.7	14.7	14.7	14.7
2022-07-04 12:00 AM	13.9	14.0	14.0	13.9
2022-07-04 1:00 AM	13.2	13.3	13.3	13.3
2022-07-04 2:00 AM	13.0	13.0	13.0	13.0
2022-07-04 3:00 AM	12.7	12.7	12.7	12.7
2022-07-04 4:00 AM	12.5	12.2	12.2	12.3
2022-07-04 5:00 AM	12.2	12.0	12.0	12.1
2022-07-04 6:00 AM	12.0	11.8	11.8	11.9
2022-07-04 7:00 AM	12.0	11.8	11.8	11.9
2022-07-04 8:00 AM	12.5	12.4	12.4	12.4
2022-07-04 9:00 AM	13.1	12.8	12.8	13.0
2022-07-04 10:00 AM	14.0	13.0	13.0	13.5
2022-07-04 11:00 AM	13.8	12.8	12.8	13.3

2022-07-04 12:00 PM	13.9	13.1	13.1	13.5
2022-07-04 1:00 PM	15.1	14.4	14.4	14.8
2022-07-04 2:00 PM	14.8	14.2	14.2	14.5
2022-07-04 3:00 PM	14.5	14.0	14.0	14.3
2022-07-04 4:00 PM	14.0	13.5	13.5	13.8
2022-07-04 5:00 PM	14.3	13.9	13.9	14.1
2022-07-04 6:00 PM	14.5	14.1	14.1	14.3
2022-07-04 7:00 PM	14.4	14.1	14.1	14.3
2022-07-04 8:00 PM	14.1	13.9	13.9	14.0
2022-07-04 9:00 PM	13.7	13.6	13.6	13.7
2022-07-04 10:00 PM	13.7	13.6	13.6	13.7
2022-07-04 11:00 PM	13.7	13.5	13.5	13.6
2022-07-05 12:00 AM	13.5	13.4	13.4	13.4
2022-07-05 1:00 AM	13.7	13.6	13.6	13.7
2022-07-05 2:00 AM	13.9	13.8	13.8	13.8
2022-07-05 3:00 AM	14.0	13.9	13.9	13.9
2022-07-05 4:00 AM	14.0	13.9	13.9	13.9
2022-07-05 5:00 AM	14.0	13.9	13.9	13.9
2022-07-05 6:00 AM	14.0	13.9	13.9	13.9
2022-07-05 7:00 AM	14.0	14.0	14.0	14.0
2022-07-05 8:00 AM	14.0	14.0	14.0	14.0
2022-07-05 9:00 AM	13.9	13.8	13.8	13.8
2022-07-05 10:00 AM	14.0	13.9	13.9	13.9
2022-07-05 11:00 AM	14.2	14.1	14.1	14.2
2022-07-05 12:00 PM	14.4	14.4	14.4	14.4
2022-07-05 1:00 PM	15.0	14.9	14.9	14.9
2022-07-05 2:00 PM	15.9	15.7	15.7	15.8
2022-07-05 3:00 PM	15.9	16.1	16.1	16.0
2022-07-05 4:00 PM	16.4	16.7	16.7	16.6
2022-07-05 5:00 PM	16.9	17.4	17.4	17.1
2022-07-05 6:00 PM	16.8	17.3	17.3	17.0
2022-07-05 7:00 PM	16.2	17.2	17.2	16.7
2022-07-05 8:00 PM	16.0	16.8	16.8	16.4
2022-07-05 9:00 PM	15.8	16.6	16.6	16.2
2022-07-05 10:00 PM	15.5	16.1	16.1	15.8
2022-07-05 11:00 PM	15.0	15.5	15.5	15.3
2022-07-06 12:00 AM	14.3	14.6	14.6	14.5
2022-07-06 1:00 AM	13.6	13.9	13.9	13.8
2022-07-06 2:00 AM	13.4	13.6	13.6	13.5
2022-07-06 3:00 AM	12.9	13.2	13.2	13.0
2022-07-06 4:00 AM	12.7	12.8	12.8	12.8
2022-07-06 5:00 AM	12.5	12.5	12.5	12.5
2022-07-06 6:00 AM	12.2	12.2	12.2	12.2

2022-07-06 7:00 AM	12.1	11.9	11.9	12.0
2022-07-06 8:00 AM	11.8	11.9	11.9	11.8
2022-07-06 9:00 AM	12.5	12.5	12.5	12.5
2022-07-06 10:00 AM	13.8	13.6	13.6	13.7
2022-07-06 11:00 AM	15.3	15.0	15.0	15.2
2022-07-06 12:00 PM	19.2	18.7	18.7	19.0
2022-07-06 1:00 PM	20.2	19.9	19.9	20.1
2022-07-06 2:00 PM	20.6	19.6	19.6	20.1
2022-07-06 3:00 PM	20.0	20.6	20.6	20.3
2022-07-06 4:00 PM	19.0	20.9	20.9	19.9
2022-07-06 5:00 PM	18.3	20.2	20.2	19.3
2022-07-06 6:00 PM	18.1	18.4	18.4	18.3
2022-07-06 7:00 PM	16.5	16.8	16.8	16.6
2022-07-06 8:00 PM	15.7	16.0	16.0	15.8
2022-07-06 9:00 PM	15.4	15.6	15.6	15.5
2022-07-06 10:00 PM	14.8	15.0	15.0	14.9
2022-07-06 11:00 PM	13.7	14.0	14.0	13.8
2022-07-07 12:00 AM	13.4	13.5	13.5	13.4
2022-07-07 1:00 AM	13.1	13.2	13.2	13.2
2022-07-07 2:00 AM	12.8	12.7	12.7	12.8
2022-07-07 3:00 AM	12.4	12.4	12.4	12.4
2022-07-07 4:00 AM	12.0	11.9	11.9	11.9
2022-07-07 5:00 AM	11.7	11.5	11.5	11.6
2022-07-07 6:00 AM	11.6	11.2	11.2	11.4
2022-07-07 7:00 AM	11.7	11.5	11.5	11.6
2022-07-07 8:00 AM	12.2	11.9	11.9	12.0
2022-07-07 9:00 AM	12.8	12.5	12.5	12.7
2022-07-07 10:00 AM	13.8	13.4	13.4	13.6
2022-07-07 11:00 AM	15.3	14.9	14.9	15.1
2022-07-07 12:00 PM	19.4	19.1	19.1	19.3
2022-07-07 1:00 PM	20.5	20.4	20.4	20.4
2022-07-07 2:00 PM	20.3	20.5	20.5	20.4
2022-07-07 3:00 PM	21.1	21.6	21.6	21.4
2022-07-07 4:00 PM	20.4	21.7	21.7	21.1
2022-07-07 5:00 PM	19.5	21.0	21.0	20.3
2022-07-07 6:00 PM	19.1	19.5	19.5	19.3
2022-07-07 7:00 PM	18.3	18.4	18.4	18.3
2022-07-07 8:00 PM	17.6	17.8	17.8	17.7
2022-07-07 9:00 PM	17.1	17.8	17.8	17.4
2022-07-07 10:00 PM	16.7	17.2	17.2	17.0
2022-07-07 11:00 PM	16.3	16.8	16.8	16.5
2022-07-08 12:00 AM	16.0	16.4	16.4	16.2
2022-07-08 1:00 AM	15.6	15.9	15.9	15.8

2022-07-08 2:00 AM	14.9	15.1	15.1	15.0
2022-07-08 3:00 AM	14.4	14.5	14.5	14.4
2022-07-08 4:00 AM	13.7	13.8	13.8	13.8
2022-07-08 5:00 AM	13.2	13.4	13.4	13.3
2022-07-08 6:00 AM	12.5	12.7	12.7	12.6
2022-07-08 7:00 AM	12.2	12.4	12.4	12.3
2022-07-08 8:00 AM	12.4	12.4	12.4	12.4
2022-07-08 9:00 AM	13.2	13.1	13.1	13.2
2022-07-08 10:00 AM	14.7	14.5	14.5	14.6
2022-07-08 11:00 AM	16.3	15.9	15.9	16.1
2022-07-08 12:00 PM	19.5	19.0	19.0	19.3
2022-07-08 1:00 PM	21.3	21.1	21.1	21.2
2022-07-08 2:00 PM	21.6	20.8	20.8	21.2
2022-07-08 3:00 PM	21.6	21.8	21.8	21.7
2022-07-08 4:00 PM	20.6	22.0	22.0	21.3
2022-07-08 5:00 PM	20.2	21.5	21.5	20.9
2022-07-08 6:00 PM	19.4	19.9	19.9	19.6
2022-07-08 7:00 PM	17.7	18.1	18.1	17.9
2022-07-08 8:00 PM	17.0	17.3	17.3	17.1
2022-07-08 9:00 PM	16.7	16.9	16.9	16.8
2022-07-08 10:00 PM	16.1	16.2	16.2	16.2
2022-07-08 11:00 PM	15.4	15.7	15.7	15.5
2022-07-09 12:00 AM	15.1	15.3	15.3	15.2
2022-07-09 1:00 AM	14.6	14.8	14.8	14.7
2022-07-09 2:00 AM	14.3	14.5	14.5	14.4
2022-07-09 3:00 AM	13.7	13.9	13.9	13.8
2022-07-09 4:00 AM	13.1	13.3	13.3	13.2
2022-07-09 5:00 AM	12.7	12.8	12.8	12.8
2022-07-09 6:00 AM	12.6	12.6	12.6	12.6
2022-07-09 7:00 AM	12.1	12.2	12.2	12.2
2022-07-09 8:00 AM	12.2	12.2	12.2	12.2
2022-07-09 9:00 AM	13.3	13.2	13.2	13.3
2022-07-09 10:00 AM	14.8	14.6	14.6	14.7
2022-07-09 11:00 AM	16.5	16.1	16.1	16.3
2022-07-09 12:00 PM	20.3	19.9	19.9	20.1
2022-07-09 1:00 PM	21.7	21.7	21.7	21.7
2022-07-09 2:00 PM	22.2	21.6	21.6	21.9
2022-07-09 3:00 PM	21.8	22.4	22.4	22.1
2022-07-09 4:00 PM	20.7	22.1	22.1	21.4
2022-07-09 5:00 PM	20.2	21.4	21.4	20.8
2022-07-09 6:00 PM	20.3	20.5	20.5	20.4
2022-07-09 7:00 PM	18.5	18.6	18.6	18.6
2022-07-09 8:00 PM	17.5	17.7	17.7	17.6

2022-07-09 9:00 PM	17.1	17.2	17.2	17.2
2022-07-09 10:00 PM	16.6	16.7	16.7	16.7
2022-07-09 11:00 PM	15.7	15.9	15.9	15.8
2022-07-10 12:00 AM	15.1	15.2	15.2	15.2
2022-07-10 1:00 AM	14.7	14.6	14.6	14.7
2022-07-10 2:00 AM	14.3	14.1	14.1	14.2
2022-07-10 3:00 AM	14.2	13.8	13.8	14.0
2022-07-10 4:00 AM	14.2	14.0	14.0	14.1
2022-07-10 5:00 AM	14.0	13.6	13.6	13.8
2022-07-10 6:00 AM	13.7	13.4	13.4	13.5
2022-07-10 7:00 AM	13.7	13.4	13.4	13.5
2022-07-10 8:00 AM	14.4	14.2	14.2	14.3
2022-07-10 9:00 AM	14.8	14.7	14.7	14.8
2022-07-10 10:00 AM	15.6	15.4	15.4	15.5
2022-07-10 11:00 AM	16.7	16.4	16.4	16.6
2022-07-10 12:00 PM	17.6	17.3	17.3	17.4
2022-07-10 1:00 PM	18.4	18.1	18.1	18.3
2022-07-10 2:00 PM	20.5	19.9	19.9	20.2
2022-07-10 3:00 PM	20.7	20.6	20.6	20.7
2022-07-10 4:00 PM	18.4	18.3	18.3	18.3
2022-07-10 5:00 PM	17.2	17.3	17.3	17.3
2022-07-10 6:00 PM	17.5	17.7	17.7	17.6
2022-07-10 7:00 PM	17.5	17.5	17.5	17.5
2022-07-10 8:00 PM	17.7	17.7	17.7	17.7
2022-07-10 9:00 PM	17.5	17.5	17.5	17.5
2022-07-10 10:00 PM	17.0	17.2	17.2	17.1
2022-07-10 11:00 PM	16.8	16.9	16.9	16.8
2022-07-11 12:00 AM	16.4	16.5	16.5	16.4
2022-07-11 1:00 AM	16.3	16.4	16.4	16.3
2022-07-11 2:00 AM	16.1	16.2	16.2	16.2
2022-07-11 3:00 AM	16.1	16.1	16.1	16.1
2022-07-11 4:00 AM	16.4	16.5	16.5	16.4
2022-07-11 5:00 AM	16.2	16.3	16.3	16.3
2022-07-11 6:00 AM	16.2	16.3	16.3	16.3
2022-07-11 7:00 AM	16.1	16.1	16.1	16.1
2022-07-11 8:00 AM	16.1	16.2	16.2	16.2
2022-07-11 9:00 AM	16.5	16.9	16.9	16.7
2022-07-11 10:00 AM	17.2	17.6	17.6	17.4
2022-07-11 11:00 AM	18.2	18.4	18.4	18.3
2022-07-11 12:00 PM	20.7	21.2	21.2	21.0
2022-07-11 1:00 PM	20.3	20.8	20.8	20.5
2022-07-11 2:00 PM	20.3	20.7	20.7	20.5
2022-07-11 3:00 PM	20.6	21.4	21.4	21.0

2022-07-11 4:00 PM	20.1	21.1	21.1	20.6
2022-07-11 5:00 PM	19.9	20.5	20.5	20.2
2022-07-11 6:00 PM	19.7	20.4	20.4	20.1
2022-07-11 7:00 PM	19.2	20.1	20.1	19.7
2022-07-11 8:00 PM	19.2	20.0	20.0	19.6
2022-07-11 9:00 PM	18.3	19.5	19.5	18.9
2022-07-11 10:00 PM	17.6	18.1	18.1	17.9
2022-07-11 11:00 PM	17.2	17.5	17.5	17.4
2022-07-12 12:00 AM	17.0	17.1	17.1	17.1
2022-07-12 1:00 AM	16.9	17.1	17.1	17.0
2022-07-12 2:00 AM	17.0	17.1	17.1	17.1
2022-07-12 3:00 AM	17.0	17.1	17.1	17.1
2022-07-12 4:00 AM	16.9	17.0	17.0	16.9
2022-07-12 5:00 AM	16.8	16.8	16.8	16.8
2022-07-12 6:00 AM	16.7	16.7	16.7	16.7
2022-07-12 7:00 AM	16.5	16.6	16.6	16.6
2022-07-12 8:00 AM	16.4	16.3	16.3	16.3
2022-07-12 9:00 AM	16.2	16.1	16.1	16.2
2022-07-12 10:00 AM	16.1	16.0	16.0	16.1
2022-07-12 11:00 AM	15.9	15.7	15.7	15.8
2022-07-12 12:00 PM	15.8	15.3	15.3	15.6
2022-07-12 1:00 PM	15.5	15.2	15.2	15.3
2022-07-12 2:00 PM	16.3	15.9	15.9	16.1
2022-07-12 3:00 PM	17.8	17.3	17.3	17.5
2022-07-12 4:00 PM	17.7	17.1	17.1	17.4
2022-07-12 5:00 PM	17.1	16.6	16.6	16.9
2022-07-12 6:00 PM	17.4	16.9	16.9	17.1
2022-07-12 7:00 PM	16.8	16.4	16.4	16.6
2022-07-12 8:00 PM	16.5	16.2	16.2	16.4
2022-07-12 9:00 PM	16.2	15.9	15.9	16.1
2022-07-12 10:00 PM	15.6	15.5	15.5	15.6
2022-07-12 11:00 PM	14.8	14.7	14.7	14.8
2022-07-13 12:00 AM	14.6	14.6	14.6	14.6
2022-07-13 1:00 AM	14.1	14.1	14.1	14.1
2022-07-13 2:00 AM	13.6	13.5	13.5	13.6
2022-07-13 3:00 AM	13.2	13.2	13.2	13.2
2022-07-13 4:00 AM	12.7	12.8	12.8	12.8
2022-07-13 5:00 AM	12.2	12.3	12.3	12.3
2022-07-13 6:00 AM	12.0	11.9	11.9	11.9
2022-07-13 7:00 AM	11.7	11.7	11.7	11.7
2022-07-13 8:00 AM	12.0	11.8	11.8	11.9
2022-07-13 9:00 AM	12.8	12.6	12.6	12.7
2022-07-13 10:00 AM	13.9	13.8	13.8	13.8

2022-07-13 11:00 AM	15.5	15.3	15.3	15.4
2022-07-13 12:00 PM	19.1	19.0	19.0	19.1
2022-07-13 1:00 PM	19.5	19.5	19.5	19.5
2022-07-13 2:00 PM	20.4	19.6	19.6	20.0
2022-07-13 3:00 PM	19.9	19.9	19.9	19.9
2022-07-13 4:00 PM	19.5	20.8	20.8	20.1
2022-07-13 5:00 PM	19.1	20.7	20.7	19.9
2022-07-13 6:00 PM	18.9	19.4	19.4	19.1
2022-07-13 7:00 PM	17.6	17.8	17.8	17.7
2022-07-13 8:00 PM	16.7	16.9	16.9	16.8
2022-07-13 9:00 PM	16.3	16.5	16.5	16.4
2022-07-13 10:00 PM	15.9	15.8	15.8	15.8
2022-07-13 11:00 PM	15.2	15.0	15.0	15.1
2022-07-14 12:00 AM	14.9	14.8	14.8	14.8
2022-07-14 1:00 AM	14.6	14.3	14.3	14.5
2022-07-14 2:00 AM	14.3	13.7	13.7	14.0
2022-07-14 3:00 AM	13.9	13.4	13.4	13.6
2022-07-14 4:00 AM	13.2	13.0	13.0	13.1
2022-07-14 5:00 AM	12.6	12.5	12.5	12.6
2022-07-14 6:00 AM	12.0	11.9	11.9	11.9
2022-07-14 7:00 AM	11.8	11.7	11.7	11.8
2022-07-14 8:00 AM	12.1	11.8	11.8	12.0
2022-07-14 9:00 AM	12.9	12.6	12.6	12.8
2022-07-14 10:00 AM	14.3	14.0	14.0	14.2
2022-07-14 11:00 AM	15.2	15.0	15.0	15.1
2022-07-14 12:00 PM	18.3	18.3	18.3	18.3
2022-07-14 1:00 PM	19.8	19.9	19.9	19.8
2022-07-14 2:00 PM	21.0	20.3	20.3	20.6
2022-07-14 3:00 PM	20.6	21.1	21.1	20.9
2022-07-14 4:00 PM	19.8	22.1	22.1	20.9
2022-07-14 5:00 PM	19.4	21.4	21.4	20.4
2022-07-14 6:00 PM	19.3	20.0	20.0	19.6
2022-07-14 7:00 PM	17.9	18.2	18.2	18.1
2022-07-14 8:00 PM	17.0	17.3	17.3	17.1
2022-07-14 9:00 PM	16.5	16.6	16.6	16.6
2022-07-14 10:00 PM	16.1	16.1	16.1	16.1
2022-07-14 11:00 PM	15.6	15.5	15.5	15.6
2022-07-15 12:00 AM	15.2	15.1	15.1	15.2
2022-07-15 1:00 AM	14.9	14.8	14.8	14.8
2022-07-15 2:00 AM	14.8	14.5	14.5	14.7
2022-07-15 3:00 AM	14.6	14.3	14.3	14.5
2022-07-15 4:00 AM	14.5	14.2	14.2	14.3
2022-07-15 5:00 AM	14.5	14.3	14.3	14.4

2022-07-15 6:00 AM	14.4	14.1	14.1	14.3
2022-07-15 7:00 AM	14.3	14.0	14.0	14.2
2022-07-15 8:00 AM	14.3	14.1	14.1	14.2
2022-07-15 9:00 AM	15.0	14.8	14.8	14.9
2022-07-15 10:00 AM	15.2	15.1	15.1	15.2
2022-07-15 11:00 AM	15.9	15.9	15.9	15.9
2022-07-15 12:00 PM	18.5	19.4	19.4	18.9
2022-07-15 1:00 PM	20.1	21.2	21.2	20.7
2022-07-15 2:00 PM	20.9	20.5	20.5	20.7
2022-07-15 3:00 PM	20.6	20.8	20.8	20.7
2022-07-15 4:00 PM	19.7	21.5	21.5	20.6
2022-07-15 5:00 PM	19.6	21.2	21.2	20.4
2022-07-15 6:00 PM	19.4	19.7	19.7	19.6
2022-07-15 7:00 PM	18.4	18.8	18.8	18.6
2022-07-15 8:00 PM	18.2	18.7	18.7	18.5
2022-07-15 9:00 PM	17.8	18.1	18.1	17.9
2022-07-15 10:00 PM	17.4	17.6	17.6	17.5
2022-07-15 11:00 PM	16.8	16.9	16.9	16.8
2022-07-16 12:00 AM	16.3	16.1	16.1	16.2
2022-07-16 1:00 AM	15.9	15.7	15.7	15.8
2022-07-16 2:00 AM	15.8	15.5	15.5	15.7
2022-07-16 3:00 AM	15.3	15.2	15.2	15.3
2022-07-16 4:00 AM	15.1	14.9	14.9	15.0
2022-07-16 5:00 AM	14.9	14.7	14.7	14.8
2022-07-16 6:00 AM	14.9	14.7	14.7	14.8
2022-07-16 7:00 AM	14.8	14.5	14.5	14.7
2022-07-16 8:00 AM	14.8	14.6	14.6	14.7
2022-07-16 9:00 AM	15.4	15.3	15.3	15.3
2022-07-16 10:00 AM	16.4	16.5	16.5	16.4
2022-07-16 11:00 AM	17.6	17.8	17.8	17.7
2022-07-16 12:00 PM	19.9	21.1	21.1	20.5
2022-07-16 1:00 PM	20.4	21.5	21.5	20.9
2022-07-16 2:00 PM	21.6	21.3	21.3	21.4
2022-07-16 3:00 PM	21.2	21.2	21.2	21.2
2022-07-16 4:00 PM	20.6	22.1	22.1	21.4
2022-07-16 5:00 PM	20.8	22.3	22.3	21.5
2022-07-16 6:00 PM	21.0	21.4	21.4	21.2
2022-07-16 7:00 PM	19.7	19.9	19.9	19.8
2022-07-16 8:00 PM	18.9	19.1	19.1	19.0
2022-07-16 9:00 PM	18.4	18.5	18.5	18.4
2022-07-16 10:00 PM	17.8	17.9	17.9	17.8
2022-07-16 11:00 PM	17.2	17.1	17.1	17.2
2022-07-17 12:00 AM	16.8	16.7	16.7	16.8

2022-07-17 1:00 AM	16.4	16.3	16.3	16.3
2022-07-17 2:00 AM	16.1	16.0	16.0	16.1
2022-07-17 3:00 AM	15.8	15.6	15.6	15.7
2022-07-17 4:00 AM	15.5	15.3	15.3	15.4
2022-07-17 5:00 AM	15.2	15.0	15.0	15.1
2022-07-17 6:00 AM	15.1	14.8	14.8	15.0
2022-07-17 7:00 AM	15.0	14.6	14.6	14.8
2022-07-17 8:00 AM	15.0	14.7	14.7	14.8
2022-07-17 9:00 AM	15.4	15.2	15.2	15.3
2022-07-17 10:00 AM	16.0	15.8	15.8	15.9
2022-07-17 11:00 AM	16.6	16.2	16.2	16.4
2022-07-17 12:00 PM	18.2	18.3	18.3	18.3
2022-07-17 1:00 PM	19.6	20.0	20.0	19.8
2022-07-17 2:00 PM	21.3	20.8	20.8	21.0
2022-07-17 3:00 PM	20.7	20.7	20.7	20.7
2022-07-17 4:00 PM	19.5	20.7	20.7	20.1
2022-07-17 5:00 PM	18.7	19.8	19.8	19.3
2022-07-17 6:00 PM	19.0	19.3	19.3	19.1
2022-07-17 7:00 PM	17.7	17.6	17.6	17.7
2022-07-17 8:00 PM	17.1	17.0	17.0	17.1
2022-07-17 9:00 PM	16.7	16.6	16.6	16.7
2022-07-17 10:00 PM	16.2	16.0	16.0	16.1
2022-07-17 11:00 PM	15.9	15.6	15.6	15.8
2022-07-18 12:00 AM	15.8	15.5	15.5	15.7
2022-07-18 1:00 AM	15.8	15.4	15.4	15.6
2022-07-18 2:00 AM	15.6	15.2	15.2	15.4
2022-07-18 3:00 AM	15.6	15.3	15.3	15.5
2022-07-18 4:00 AM	15.4	15.2	15.2	15.3
2022-07-18 5:00 AM	15.5	15.3	15.3	15.4
2022-07-18 6:00 AM	15.6	15.4	15.4	15.5
2022-07-18 7:00 AM	15.8	15.7	15.7	15.8
2022-07-18 8:00 AM	15.7	15.6	15.6	15.7
2022-07-18 9:00 AM	16.3	16.3	16.3	16.3
2022-07-18 10:00 AM	17.2	17.3	17.3	17.3
2022-07-18 11:00 AM	18.2	18.5	18.5	18.4
2022-07-18 12:00 PM	21.1	22.7	22.7	21.9
2022-07-18 1:00 PM	23.4	25.5	25.5	24.4
2022-07-18 2:00 PM	25.1	25.7	25.7	25.4
2022-07-18 3:00 PM	24.0	24.9	24.9	24.4
2022-07-18 4:00 PM	24.2	26.1	26.1	25.2
2022-07-18 5:00 PM	24.1	25.7	25.7	24.9
2022-07-18 6:00 PM	23.9	25.1	25.1	24.5
2022-07-18 7:00 PM	22.6	24.5	24.5	23.6

2022-07-18 8:00 PM	22.2	23.1	23.1	22.7
2022-07-18 9:00 PM	21.9	22.4	22.4	22.1
2022-07-18 10:00 PM	21.2	21.6	21.6	21.4
2022-07-18 11:00 PM	20.6	21.1	21.1	20.9
2022-07-19 12:00 AM	20.0	20.3	20.3	20.1
2022-07-19 1:00 AM	19.9	20.1	20.1	20.0
2022-07-19 2:00 AM	19.9	20.1	20.1	20.0
2022-07-19 3:00 AM	19.7	19.8	19.8	19.8
2022-07-19 4:00 AM	19.6	19.8	19.8	19.7
2022-07-19 5:00 AM	19.3	19.6	19.6	19.4
2022-07-19 6:00 AM	19.2	19.3	19.3	19.3
2022-07-19 7:00 AM	19.0	19.2	19.2	19.1
2022-07-19 8:00 AM	19.0	19.1	19.1	19.1
2022-07-19 9:00 AM	18.9	19.0	19.0	18.9
2022-07-19 10:00 AM	18.9	19.0	19.0	18.9
2022-07-19 11:00 AM	18.8	18.8	18.8	18.8
2022-07-19 12:00 PM	18.3	18.3	18.3	18.3
2022-07-19 1:00 PM	17.7	17.2	17.2	17.5
2022-07-19 2:00 PM	17.9	17.6	17.6	17.8
2022-07-19 3:00 PM	18.1	17.9	17.9	18.0
2022-07-19 4:00 PM	17.6	17.3	17.3	17.4
2022-07-19 5:00 PM	17.3	16.9	16.9	17.1
2022-07-19 6:00 PM	17.8	17.4	17.4	17.6
2022-07-19 7:00 PM	18.0	17.7	17.7	17.9
2022-07-19 8:00 PM	17.9	17.6	17.6	17.8
2022-07-19 9:00 PM	17.5	17.2	17.2	17.4
2022-07-19 10:00 PM	17.2	16.8	16.8	17.0
2022-07-19 11:00 PM	16.9	16.5	16.5	16.7
2022-07-20 12:00 AM	16.8	16.5	16.5	16.6
2022-07-20 1:00 AM	16.9	16.6	16.6	16.8
2022-07-20 2:00 AM	16.8	16.5	16.5	16.6
2022-07-20 3:00 AM	16.6	16.4	16.4	16.5
2022-07-20 4:00 AM	16.5	16.2	16.2	16.4
2022-07-20 5:00 AM	16.3	15.9	15.9	16.1
2022-07-20 6:00 AM	15.8	15.5	15.5	15.7
2022-07-20 7:00 AM	15.5	15.2	15.2	15.3
2022-07-20 8:00 AM	15.5	15.2	15.2	15.3
2022-07-20 9:00 AM	16.1	15.9	15.9	16.0
2022-07-20 10:00 AM	16.7	16.5	16.5	16.6
2022-07-20 11:00 AM	17.7	17.9	17.9	17.8
2022-07-20 12:00 PM	19.1	19.4	19.4	19.3
2022-07-20 1:00 PM	19.5	19.8	19.8	19.6
2022-07-20 2:00 PM	19.5	19.8	19.8	19.6

2022-07-20 3:00 PM	19.4	19.6	19.6	19.5
2022-07-20 4:00 PM	19.3	20.0	20.0	19.6
2022-07-20 5:00 PM	19.2	19.8	19.8	19.5
2022-07-20 6:00 PM	19.3	19.9	19.9	19.6
2022-07-20 7:00 PM	19.4	20.1	20.1	19.8
2022-07-20 8:00 PM	19.4	20.3	20.3	19.8
2022-07-20 9:00 PM	19.4	20.3	20.3	19.8
2022-07-20 10:00 PM	19.3	19.9	19.9	19.6
2022-07-20 11:00 PM	18.7	19.0	19.0	18.9
2022-07-21 12:00 AM	18.2	18.4	18.4	18.3
2022-07-21 1:00 AM	17.9	18.0	18.0	17.9
2022-07-21 2:00 AM	17.5	17.7	17.7	17.6
2022-07-21 3:00 AM	17.3	17.4	17.4	17.3
2022-07-21 4:00 AM	17.4	17.6	17.6	17.5
2022-07-21 5:00 AM	17.4	17.5	17.5	17.4
2022-07-21 6:00 AM	17.3	17.5	17.5	17.4
2022-07-21 7:00 AM	17.2	17.3	17.3	17.3
2022-07-21 8:00 AM	17.0	17.2	17.2	17.1
2022-07-21 9:00 AM	17.3	17.7	17.7	17.5
2022-07-21 10:00 AM	17.7	18.6	18.6	18.2
2022-07-21 11:00 AM	18.3	19.8	19.8	19.0
2022-07-21 12:00 PM	20.1	22.5	22.5	21.3
2022-07-21 1:00 PM	21.0	23.1	23.1	22.1
2022-07-21 2:00 PM	21.5	22.8	22.8	22.1
2022-07-21 3:00 PM	21.8	23.7	23.7	22.8
2022-07-21 4:00 PM	21.5	24.0	24.0	22.8
2022-07-21 5:00 PM	21.8	23.8	23.8	22.8
2022-07-21 6:00 PM	20.9	21.9	21.9	21.4
2022-07-21 7:00 PM	19.6	20.2	20.2	19.9
2022-07-21 8:00 PM	19.3	19.6	19.6	19.4
2022-07-21 9:00 PM	19.1	19.2	19.2	19.2
2022-07-21 10:00 PM	18.7	18.7	18.7	18.7
2022-07-21 11:00 PM	17.9	17.8	17.8	17.8
2022-07-22 12:00 AM	17.3	17.0	17.0	17.1
2022-07-22 1:00 AM	16.9	16.6	16.6	16.8
2022-07-22 2:00 AM	16.6	16.3	16.3	16.4
2022-07-22 3:00 AM	16.3	16.0	16.0	16.1
2022-07-22 4:00 AM	16.0	15.7	15.7	15.8
2022-07-22 5:00 AM	15.7	15.4	15.4	15.5
2022-07-22 6:00 AM	15.5	15.1	15.1	15.3
2022-07-22 7:00 AM	15.3	15.0	15.0	15.2
2022-07-22 8:00 AM	15.4	15.0	15.0	15.2
2022-07-22 9:00 AM	16.0	15.7	15.7	15.8

2022-07-22 10:00 AM	17.0	16.9	16.9	16.9
2022-07-22 11:00 AM	18.1	18.2	18.2	18.2
2022-07-22 12:00 PM	20.4	21.7	21.7	21.1
2022-07-22 1:00 PM	21.8	23.9	23.9	22.8
2022-07-22 2:00 PM	22.9	24.0	24.0	23.4
2022-07-22 3:00 PM	23.1	24.7	24.7	23.9
2022-07-22 4:00 PM	21.1	23.8	23.8	22.4
2022-07-22 5:00 PM	20.6	23.0	23.0	21.8
2022-07-22 6:00 PM	20.0	22.2	22.2	21.1
2022-07-22 7:00 PM	20.0	21.6	21.6	20.8
2022-07-22 8:00 PM	19.9	21.0	21.0	20.4
2022-07-22 9:00 PM	19.7	21.1	21.1	20.4
2022-07-22 10:00 PM	19.3	19.8	19.8	19.5
2022-07-22 11:00 PM	18.6	18.9	18.9	18.8
2022-07-23 12:00 AM	18.3	18.4	18.4	18.3
2022-07-23 1:00 AM	17.8	17.8	17.8	17.8
2022-07-23 2:00 AM	17.5	17.4	17.4	17.4
2022-07-23 3:00 AM	17.0	16.8	16.8	16.9
2022-07-23 4:00 AM	16.5	16.4	16.4	16.4
2022-07-23 5:00 AM	16.3	16.0	16.0	16.1
2022-07-23 6:00 AM	16.1	15.8	15.8	16.0
2022-07-23 7:00 AM	16.0	15.7	15.7	15.8
2022-07-23 8:00 AM	16.1	15.9	15.9	16.0
2022-07-23 9:00 AM	16.7	16.4	16.4	16.6
2022-07-23 10:00 AM	17.4	17.2	17.2	17.3
2022-07-23 11:00 AM	18.1	18.1	18.1	18.1
2022-07-23 12:00 PM	20.1	21.3	21.3	20.7
2022-07-23 1:00 PM	21.4	22.6	22.6	22.0
2022-07-23 2:00 PM	20.6	21.0	21.0	20.8
2022-07-23 3:00 PM	20.3	20.7	20.7	20.5
2022-07-23 4:00 PM	19.2	19.4	19.4	19.3
2022-07-23 5:00 PM	19.5	19.7	19.7	19.6
2022-07-23 6:00 PM	19.1	19.1	19.1	19.1
2022-07-23 7:00 PM	18.5	18.4	18.4	18.4
2022-07-23 8:00 PM	18.2	18.1	18.1	18.2
2022-07-23 9:00 PM	18.1	18.0	18.0	18.1
2022-07-23 10:00 PM	18.0	17.9	17.9	17.9
2022-07-23 11:00 PM	17.8	17.7	17.7	17.8
2022-07-24 12:00 AM	17.6	17.5	17.5	17.6
2022-07-24 1:00 AM	17.3	17.1	17.1	17.2
2022-07-24 2:00 AM	17.4	17.3	17.3	17.3
2022-07-24 3:00 AM	17.2	17.1	17.1	17.2
2022-07-24 4:00 AM	17.1	17.0	17.0	17.1

2022-07-24 5:00 AM	17.0	16.8	16.8	16.9
2022-07-24 6:00 AM	16.9	16.8	16.8	16.8
2022-07-24 7:00 AM	16.8	16.7	16.7	16.8
2022-07-24 8:00 AM	16.7	16.5	16.5	16.6
2022-07-24 9:00 AM	16.8	16.7	16.7	16.8
2022-07-24 10:00 AM	17.0	16.9	16.9	16.9
2022-07-24 11:00 AM	17.3	17.5	17.5	17.4
2022-07-24 12:00 PM	17.2	17.4	17.4	17.3
2022-07-24 1:00 PM	17.1	17.4	17.4	17.3
2022-07-24 2:00 PM	16.8	17.2	17.2	17.0
2022-07-24 3:00 PM	16.2	16.3	16.3	16.3
2022-07-24 4:00 PM	15.7	15.6	15.6	15.7
2022-07-24 5:00 PM	15.9	16.1	16.1	16.0
2022-07-24 6:00 PM	15.9	16.0	16.0	15.9
2022-07-24 7:00 PM	15.6	15.6	15.6	15.6
2022-07-24 8:00 PM	15.6	15.5	15.5	15.6
2022-07-24 9:00 PM	15.5	15.3	15.3	15.4
2022-07-24 10:00 PM	15.2	14.8	14.8	15.0
2022-07-24 11:00 PM	15.0	14.6	14.6	14.8
2022-07-25 12:00 AM	15.1	14.7	14.7	14.9
2022-07-25 1:00 AM	15.1	14.7	14.7	14.9
2022-07-25 2:00 AM	14.9	14.5	14.5	14.7
2022-07-25 3:00 AM	14.8	14.4	14.4	14.6
2022-07-25 4:00 AM	14.5	14.1	14.1	14.3
2022-07-25 5:00 AM	14.0	13.5	13.5	13.8
2022-07-25 6:00 AM	13.7	13.3	13.3	13.5
2022-07-25 7:00 AM	13.5	13.0	13.0	13.3
2022-07-25 8:00 AM	13.6	13.1	13.1	13.4
2022-07-25 9:00 AM	14.0	13.6	13.6	13.8
2022-07-25 10:00 AM	14.8	14.6	14.6	14.7
2022-07-25 11:00 AM	15.6	15.8	15.8	15.7
2022-07-25 12:00 PM	17.0	18.3	18.3	17.6
2022-07-25 1:00 PM	18.4	20.2	20.2	19.3
2022-07-25 2:00 PM	19.7	20.7	20.7	20.2
2022-07-25 3:00 PM	19.4	20.7	20.7	20.1
2022-07-25 4:00 PM	19.6	21.5	21.5	20.6
2022-07-25 5:00 PM	19.5	21.1	21.1	20.3
2022-07-25 6:00 PM	19.7	20.5	20.5	20.1
2022-07-25 7:00 PM	18.7	19.3	19.3	19.0
2022-07-25 8:00 PM	18.1	18.7	18.7	18.4
2022-07-25 9:00 PM	17.8	18.0	18.0	17.9
2022-07-25 10:00 PM	17.2	17.2	17.2	17.2
2022-07-25 11:00 PM	16.6	16.5	16.5	16.6

2022-07-26 12:00 AM	16.0	15.8	15.8	15.9
2022-07-26 1:00 AM	15.4	15.1	15.1	15.3
2022-07-26 2:00 AM	15.2	14.8	14.8	15.0
2022-07-26 3:00 AM	14.9	14.6	14.6	14.8
2022-07-26 4:00 AM	14.7	14.4	14.4	14.5
2022-07-26 5:00 AM	14.3	14.0	14.0	14.2
2022-07-26 6:00 AM	14.3	13.9	13.9	14.1
2022-07-26 7:00 AM	14.2	13.7	13.7	13.9
2022-07-26 8:00 AM	14.3	13.9	13.9	14.1
2022-07-26 9:00 AM	14.9	14.7	14.7	14.8
2022-07-26 10:00 AM	15.8	15.5	15.5	15.7
2022-07-26 11:00 AM	16.4	16.2	16.2	16.3
2022-07-26 12:00 PM	17.1	17.0	17.0	17.1
2022-07-26 1:00 PM	17.8	17.9	17.9	17.8
2022-07-26 2:00 PM	19.5	19.9	19.9	19.7
2022-07-26 3:00 PM	19.0	19.4	19.4	19.2
2022-07-26 4:00 PM	18.7	19.0	19.0	18.9
2022-07-26 5:00 PM	19.0	19.3	19.3	19.1
2022-07-26 6:00 PM	19.1	19.3	19.3	19.2
2022-07-26 7:00 PM	18.7	18.9	18.9	18.8
2022-07-26 8:00 PM	18.4	18.6	18.6	18.5
2022-07-26 9:00 PM	18.1	18.1	18.1	18.1
2022-07-26 10:00 PM	18.0	18.1	18.1	18.1
2022-07-26 11:00 PM	17.6	17.6	17.6	17.6
2022-07-27 12:00 AM	17.3	17.3	17.3	17.3
2022-07-27 1:00 AM	17.3	17.3	17.3	17.3
2022-07-27 2:00 AM	17.1	17.2	17.2	17.2
2022-07-27 3:00 AM	17.1	17.1	17.1	17.1
2022-07-27 4:00 AM	16.7	16.6	16.6	16.7
2022-07-27 5:00 AM	16.2	16.1	16.1	16.2
2022-07-27 6:00 AM	15.9	15.9	15.9	15.9
2022-07-27 7:00 AM	15.7	15.6	15.6	15.7
2022-07-27 8:00 AM	15.8	15.6	15.6	15.7
2022-07-27 9:00 AM	16.1	16.0	16.0	16.1
2022-07-27 10:00 AM	16.5	16.5	16.5	16.5
2022-07-27 11:00 AM	17.0	17.0	17.0	17.0
2022-07-27 12:00 PM	18.8	19.7	19.7	19.3
2022-07-27 1:00 PM	20.7	22.1	22.1	21.4
2022-07-27 2:00 PM	21.6	22.1	22.1	21.9
2022-07-27 3:00 PM	20.4	21.2	21.2	20.8
2022-07-27 4:00 PM	20.8	22.5	22.5	21.6
2022-07-27 5:00 PM	20.7	22.0	22.0	21.4
2022-07-27 6:00 PM	20.1	20.7	20.7	20.4

2022-07-27 7:00 PM	18.4	18.7	18.7	18.6
2022-07-27 8:00 PM	17.7	17.7	17.7	17.7
2022-07-27 9:00 PM	16.5	16.2	16.2	16.4
2022-07-27 10:00 PM	16.6	16.3	16.3	16.4
2022-07-27 11:00 PM	16.4	16.2	16.2	16.3
2022-07-28 12:00 AM	16.1	15.9	15.9	16.0
2022-07-28 1:00 AM	15.6	15.5	15.5	15.6
2022-07-28 2:00 AM	15.7	15.5	15.5	15.6
2022-07-28 3:00 AM	15.7	15.4	15.4	15.5
2022-07-28 4:00 AM	15.7	15.4	15.4	15.5
2022-07-28 5:00 AM	15.6	15.4	15.4	15.5
2022-07-28 6:00 AM	15.5	15.3	15.3	15.4
2022-07-28 7:00 AM	15.3	15.0	15.0	15.2
2022-07-28 8:00 AM	15.2	14.9	14.9	15.0
2022-07-28 9:00 AM	15.4	15.1	15.1	15.3
2022-07-28 10:00 AM	15.3	15.0	15.0	15.2
2022-07-28 11:00 AM	15.6	15.6	15.6	15.6
2022-07-28 12:00 PM	16.1	16.4	16.4	16.3
2022-07-28 1:00 PM	17.0	17.8	17.8	17.4
2022-07-28 2:00 PM	17.7	18.4	18.4	18.1
2022-07-28 3:00 PM	17.2	17.9	17.9	17.6
2022-07-28 4:00 PM	16.9	17.2	17.2	17.1
2022-07-28 5:00 PM	17.8	18.7	18.7	18.3
2022-07-28 6:00 PM	17.7	18.2	18.2	18.0
2022-07-28 7:00 PM	17.1	17.4	17.4	17.3
2022-07-28 8:00 PM	17.0	17.3	17.3	17.1
2022-07-28 9:00 PM	16.9	17.0	17.0	16.9
2022-07-28 10:00 PM	16.4	16.3	16.3	16.3
2022-07-28 11:00 PM	16.3	16.2	16.2	16.3
2022-07-29 12:00 AM	16.0	15.9	15.9	15.9
2022-07-29 1:00 AM	15.6	15.5	15.5	15.6
2022-07-29 2:00 AM	15.4	15.3	15.3	15.3
2022-07-29 3:00 AM	15.5	15.3	15.3	15.4
2022-07-29 4:00 AM	15.5	15.4	15.4	15.4
2022-07-29 5:00 AM	15.4	15.2	15.2	15.3
2022-07-29 6:00 AM	15.3	15.1	15.1	15.2
2022-07-29 7:00 AM	15.5	15.2	15.2	15.3
2022-07-29 8:00 AM	15.4	15.3	15.3	15.3
2022-07-29 9:00 AM	15.3	15.2	15.2	15.3
2022-07-29 10:00 AM	15.8	16.1	16.1	16.0
2022-07-29 11:00 AM	16.4	17.4	17.4	16.9
2022-07-29 12:00 PM	17.2	18.3	18.3	17.8
2022-07-29 1:00 PM	17.8	18.8	18.8	18.3

2022-07-29 2:00 PM	18.1	18.8	18.8	18.4
2022-07-29 3:00 PM	18.1	18.6	18.6	18.4
2022-07-29 4:00 PM	18.5	18.6	18.6	18.6
2022-07-29 5:00 PM	18.0	17.8	17.8	17.9
2022-07-29 6:00 PM	17.5	17.3	17.3	17.4
2022-07-29 7:00 PM	17.5	17.4	17.4	17.4
2022-07-29 8:00 PM	16.8	17.0	17.0	16.9
2022-07-29 9:00 PM	16.8	16.8	16.8	16.8
2022-07-29 10:00 PM	16.4	16.2	16.2	16.3
2022-07-29 11:00 PM	16.3	16.2	16.2	16.3
2022-07-30 12:00 AM	16.1	16.0	16.0	16.1
2022-07-30 1:00 AM	15.8	15.5	15.5	15.7
2022-07-30 2:00 AM	15.4	15.1	15.1	15.3
2022-07-30 3:00 AM	15.1	14.7	14.7	14.9
2022-07-30 4:00 AM	14.7	14.4	14.4	14.5
2022-07-30 5:00 AM	14.4	14.0	14.0	14.2
2022-07-30 6:00 AM	14.3	13.9	13.9	14.1
2022-07-30 7:00 AM	14.2	13.7	13.7	13.9
2022-07-30 8:00 AM	14.3	13.9	13.9	14.1
2022-07-30 9:00 AM	14.8	14.6	14.6	14.7
2022-07-30 10:00 AM	15.7	15.5	15.5	15.6
2022-07-30 11:00 AM	16.8	16.8	16.8	16.8
2022-07-30 12:00 PM	19.5	20.6	20.6	20.1
2022-07-30 1:00 PM	21.3	22.7	22.7	22.0
2022-07-30 2:00 PM	21.1	21.8	21.8	21.4
2022-07-30 3:00 PM	21.1	22.4	22.4	21.8
2022-07-30 4:00 PM	21.3	23.2	23.2	22.3
2022-07-30 5:00 PM	21.7	23.7	23.7	22.7
2022-07-30 6:00 PM	21.7	22.8	22.8	22.3
2022-07-30 7:00 PM	20.5	21.1	21.1	20.8
2022-07-30 8:00 PM	19.7	20.2	20.2	20.0
2022-07-30 9:00 PM	19.2	19.6	19.6	19.4
2022-07-30 10:00 PM	18.6	18.7	18.7	18.7
2022-07-30 11:00 PM	18.0	18.0	18.0	18.0
2022-07-31 12:00 AM	17.5	17.4	17.4	17.4
2022-07-31 1:00 AM	17.1	17.0	17.0	17.1
2022-07-31 2:00 AM	16.9	16.7	16.7	16.8
2022-07-31 3:00 AM	16.7	16.5	16.5	16.6
2022-07-31 4:00 AM	16.5	16.3	16.3	16.4
2022-07-31 5:00 AM	16.2	16.0	16.0	16.1
2022-07-31 6:00 AM	16.3	15.9	15.9	16.1
2022-07-31 7:00 AM	16.0	15.7	15.7	15.8
2022-07-31 8:00 AM	16.0	15.8	15.8	15.9

2022-07-31 9:00 AM	16.6	16.4	16.4	16.5
2022-07-31 10:00 AM	17.1	17.0	17.0	17.1
2022-07-31 11:00 AM	17.6	17.6	17.6	17.6
2022-07-31 12:00 PM	18.9	19.2	19.2	19.1
2022-07-31 1:00 PM	20.2	21.2	21.2	20.7
2022-07-31 2:00 PM	21.3	22.1	22.1	21.7
2022-07-31 3:00 PM	19.3	19.7	19.7	19.5
2022-07-31 4:00 PM	19.0	19.3	19.3	19.1
2022-07-31 5:00 PM	20.2	20.7	20.7	20.5
2022-07-31 6:00 PM	19.6	19.9	19.9	19.8
2022-07-31 7:00 PM	19.1	19.1	19.1	19.1
2022-07-31 8:00 PM	18.8	18.8	18.8	18.8
2022-07-31 9:00 PM	18.3	18.3	18.3	18.3
2022-07-31 10:00 PM	17.8	17.6	17.6	17.7
2022-07-31 11:00 PM	17.6	17.4	17.4	17.5
2022-08-01 12:00 AM	17.3	17.1	17.1	17.2
2022-08-01 1:00 AM	17.0	16.8	16.8	16.9
2022-08-01 2:00 AM	17.0	16.7	16.7	16.9
2022-08-01 3:00 AM	17.0	16.8	16.8	16.9
2022-08-01 4:00 AM	17.0	16.8	16.8	16.9
2022-08-01 5:00 AM	17.0	16.8	16.8	16.9
2022-08-01 6:00 AM	16.9	16.7	16.7	16.8
2022-08-01 7:00 AM	17.0	16.9	16.9	16.9
2022-08-01 8:00 AM	17.1	17.0	17.0	17.1
2022-08-01 9:00 AM	16.9	16.7	16.7	16.8
2022-08-01 10:00 AM	16.6	16.5	16.5	16.6
2022-08-01 11:00 AM	16.7	16.9	16.9	16.8
2022-08-01 12:00 PM	16.7	17.2	17.2	17.0
2022-08-01 1:00 PM	17.0	17.8	17.8	17.4
2022-08-01 2:00 PM	17.1	17.9	17.9	17.5
2022-08-01 3:00 PM	17.3	18.1	18.1	17.7
2022-08-01 4:00 PM	17.6	18.2	18.2	17.9
2022-08-01 5:00 PM	18.3	19.3	19.3	18.8
2022-08-01 6:00 PM	18.4	19.1	19.1	18.8
2022-08-01 7:00 PM	18.0	18.5	18.5	18.3
2022-08-01 8:00 PM	17.7	18.2	18.2	18.0
2022-08-01 9:00 PM	17.4	17.7	17.7	17.6
2022-08-01 10:00 PM	16.7	16.6	16.6	16.7
2022-08-01 11:00 PM	16.4	16.2	16.2	16.3
2022-08-02 12:00 AM	16.1	15.7	15.7	15.9
2022-08-02 1:00 AM	15.6	15.2	15.2	15.4
2022-08-02 2:00 AM	15.3	14.8	14.8	15.1
2022-08-02 3:00 AM	15.2	14.7	14.7	14.9

2022-08-02 4:00 AM	14.9	14.4	14.4	14.6
2022-08-02 5:00 AM	14.7	14.1	14.1	14.4
2022-08-02 6:00 AM	14.3	14.0	14.0	14.2
2022-08-02 7:00 AM	14.6	14.1	14.1	14.4
2022-08-02 8:00 AM	14.7	14.2	14.2	14.4
2022-08-02 9:00 AM	15.1	14.7	14.7	14.9
2022-08-02 10:00 AM	16.1	15.9	15.9	16.0
2022-08-02 11:00 AM	16.5	16.4	16.4	16.4
2022-08-02 12:00 PM	18.4	19.2	19.2	18.8
2022-08-02 1:00 PM	19.9	21.3	21.3	20.6
2022-08-02 2:00 PM	21.5	22.1	22.1	21.8
2022-08-02 3:00 PM	21.1	22.1	22.1	21.6
2022-08-02 4:00 PM	20.7	22.3	22.3	21.5
2022-08-02 5:00 PM	19.7	20.5	20.5	20.1
2022-08-02 6:00 PM	18.9	19.1	19.1	19.0
2022-08-02 7:00 PM	18.1	18.0	18.0	18.1
2022-08-02 8:00 PM	17.5	17.3	17.3	17.4
2022-08-02 9:00 PM	17.2	17.0	17.0	17.1
2022-08-02 10:00 PM	17.0	16.9	16.9	16.9
2022-08-02 11:00 PM	16.7	16.5	16.5	16.6
2022-08-03 12:00 AM	16.6	16.3	16.3	16.4
2022-08-03 1:00 AM	16.6	16.4	16.4	16.5
2022-08-03 2:00 AM	16.6	16.3	16.3	16.4
2022-08-03 3:00 AM	16.6	16.3	16.3	16.4
2022-08-03 4:00 AM	16.6	16.4	16.4	16.5
2022-08-03 5:00 AM	16.6	16.4	16.4	16.5
2022-08-03 6:00 AM	16.3	16.2	16.2	16.3
2022-08-03 7:00 AM	16.2	16.0	16.0	16.1
2022-08-03 8:00 AM	16.1	15.9	15.9	16.0
2022-08-03 9:00 AM	16.5	16.4	16.4	16.4
2022-08-03 10:00 AM	17.6	17.7	17.7	17.7
2022-08-03 11:00 AM	18.6	18.8	18.8	18.7
2022-08-03 12:00 PM	19.9	20.4	20.4	20.1
2022-08-03 1:00 PM	19.6	19.9	19.9	19.8
2022-08-03 2:00 PM	20.5	21.2	21.2	20.9
2022-08-03 3:00 PM	20.2	21.9	21.9	21.1
2022-08-03 4:00 PM	20.3	22.6	22.6	21.4
2022-08-03 5:00 PM	20.5	22.1	22.1	21.3
2022-08-03 6:00 PM	20.1	20.7	20.7	20.4
2022-08-03 7:00 PM	19.3	19.8	19.8	19.5
2022-08-03 8:00 PM	19.1	19.5	19.5	19.3
2022-08-03 9:00 PM	18.7	19.0	19.0	18.9
2022-08-03 10:00 PM	18.2	18.4	18.4	18.3

2022-08-03 11:00 PM	17.8	17.7	17.7	17.8
2022-08-04 12:00 AM	17.6	17.4	17.4	17.5
2022-08-04 1:00 AM	17.3	17.1	17.1	17.2
2022-08-04 2:00 AM	16.9	16.6	16.6	16.8
2022-08-04 3:00 AM	16.5	16.2	16.2	16.4
2022-08-04 4:00 AM	15.8	15.3	15.3	15.6
2022-08-04 5:00 AM	15.3	14.8	14.8	15.1
2022-08-04 6:00 AM	14.9	14.3	14.3	14.6
2022-08-04 7:00 AM	14.5	14.1	14.1	14.3
2022-08-04 8:00 AM	14.5	14.0	14.0	14.3
2022-08-04 9:00 AM	15.0	14.6	14.6	14.8
2022-08-04 10:00 AM	16.1	15.8	15.8	16.0
2022-08-04 11:00 AM	16.8	16.5	16.5	16.6
2022-08-04 12:00 PM	18.4	18.5	18.5	18.4
2022-08-04 1:00 PM	19.4	20.4	20.4	19.9
2022-08-04 2:00 PM	20.8	21.5	21.5	21.1
2022-08-04 3:00 PM	20.0	21.4	21.4	20.7
2022-08-04 4:00 PM	20.1	21.9	21.9	21.0
2022-08-04 5:00 PM	19.9	21.5	21.5	20.7
2022-08-04 6:00 PM	19.7	20.6	20.6	20.2
2022-08-04 7:00 PM	18.6	19.1	19.1	18.9
2022-08-04 8:00 PM	18.1	18.4	18.4	18.3
2022-08-04 9:00 PM	17.8	17.9	17.9	17.8
2022-08-04 10:00 PM	17.4	17.3	17.3	17.3
2022-08-04 11:00 PM	16.9	16.8	16.8	16.8
2022-08-05 12:00 AM	16.6	16.3	16.3	16.4
2022-08-05 1:00 AM	16.1	15.8	15.8	16.0
2022-08-05 2:00 AM	15.8	15.4	15.4	15.6
2022-08-05 3:00 AM	15.6	15.1	15.1	15.4
2022-08-05 4:00 AM	15.4	14.8	14.8	15.1
2022-08-05 5:00 AM	15.2	14.8	14.8	15.0
2022-08-05 6:00 AM	15.2	14.7	14.7	14.9
2022-08-05 7:00 AM	15.0	14.5	14.5	14.8
2022-08-05 8:00 AM	15.2	14.8	14.8	15.0
2022-08-05 9:00 AM	15.6	15.3	15.3	15.5
2022-08-05 10:00 AM	16.0	15.8	15.8	15.9
2022-08-05 11:00 AM	16.4	16.3	16.3	16.3
2022-08-05 12:00 PM	18.4	19.1	19.1	18.8
2022-08-05 1:00 PM	20.3	21.8	21.8	21.0
2022-08-05 2:00 PM	21.5	22.5	22.5	22.0
2022-08-05 3:00 PM	20.6	21.5	21.5	21.1
2022-08-05 4:00 PM	20.9	22.9	22.9	21.9
2022-08-05 5:00 PM	20.8	22.3	22.3	21.5

2022-08-05 6:00 PM	19.8	20.3	20.3	20.0
2022-08-05 7:00 PM	18.9	19.1	19.1	19.0
2022-08-05 8:00 PM	18.7	18.9	18.9	18.8
2022-08-05 9:00 PM	18.5	18.5	18.5	18.5
2022-08-05 10:00 PM	18.0	18.0	18.0	18.0
2022-08-05 11:00 PM	17.8	17.7	17.7	17.8
2022-08-06 12:00 AM	17.4	17.1	17.1	17.3
2022-08-06 1:00 AM	17.3	17.1	17.1	17.2
2022-08-06 2:00 AM	17.5	17.4	17.4	17.4
2022-08-06 3:00 AM	17.5	17.4	17.4	17.4
2022-08-06 4:00 AM	17.5	17.5	17.5	17.5
2022-08-06 5:00 AM	17.7	17.7	17.7	17.7
2022-08-06 6:00 AM	17.9	18.0	18.0	17.9
2022-08-06 7:00 AM	17.8	17.9	17.9	17.8
2022-08-06 8:00 AM	17.9	18.0	18.0	17.9
2022-08-06 9:00 AM	18.4	18.5	18.5	18.4
2022-08-06 10:00 AM	19.1	19.3	19.3	19.2
2022-08-06 11:00 AM	19.5	19.9	19.9	19.7
2022-08-06 12:00 PM	20.3	20.6	20.6	20.4
2022-08-06 1:00 PM	20.4	20.7	20.7	20.6
2022-08-06 2:00 PM	20.5	20.8	20.8	20.6
2022-08-06 3:00 PM	20.8	21.9	21.9	21.3
2022-08-06 4:00 PM	20.8	22.4	22.4	21.6
2022-08-06 5:00 PM	20.4	22.2	22.2	21.3
2022-08-06 6:00 PM	20.9	21.7	21.7	21.3
2022-08-06 7:00 PM	20.5	21.3	21.3	20.9
2022-08-06 8:00 PM	19.8	20.7	20.7	20.3
2022-08-06 9:00 PM	19.4	19.8	19.8	19.6
2022-08-06 10:00 PM	18.9	19.0	19.0	18.9
2022-08-06 11:00 PM	18.3	18.3	18.3	18.3
2022-08-07 12:00 AM	17.6	17.3	17.3	17.4
2022-08-07 1:00 AM	17.1	16.7	16.7	16.9
2022-08-07 2:00 AM	16.8	16.3	16.3	16.5
2022-08-07 3:00 AM	16.6	16.0	16.0	16.3
2022-08-07 4:00 AM	16.3	15.7	15.7	16.0
2022-08-07 5:00 AM	16.2	15.4	15.4	15.8
2022-08-07 6:00 AM	15.9	15.0	15.0	15.4
2022-08-07 7:00 AM	15.7	14.8	14.8	15.3
2022-08-07 8:00 AM	15.5	14.6	14.6	15.1
2022-08-07 9:00 AM	15.6	14.7	14.7	15.2
2022-08-07 10:00 AM	15.8	15.1	15.1	15.5
2022-08-07 11:00 AM	16.4	15.9	15.9	16.1
2022-08-07 12:00 PM	17.8	17.5	17.5	17.6

2022-08-07 1:00 PM	19.1	19.5	19.5	19.3
2022-08-07 2:00 PM	19.7	19.8	19.8	19.8
2022-08-07 3:00 PM	19.2	20.1	20.1	19.7
2022-08-07 4:00 PM	18.6	20.0	20.0	19.3
2022-08-07 5:00 PM	18.0	18.6	18.6	18.3
2022-08-07 6:00 PM	17.4	17.2	17.2	17.3
2022-08-07 7:00 PM	17.0	16.6	16.6	16.8
2022-08-07 8:00 PM	16.7	16.3	16.3	16.5
2022-08-07 9:00 PM	16.4	16.0	16.0	16.2
2022-08-07 10:00 PM	16.1	15.6	15.6	15.9
2022-08-07 11:00 PM	15.9	15.4	15.4	15.6
2022-08-08 12:00 AM	15.8	15.3	15.3	15.6
2022-08-08 1:00 AM	15.6	15.1	15.1	15.4
2022-08-08 2:00 AM	15.4	14.9	14.9	15.1
2022-08-08 3:00 AM	15.1	14.7	14.7	14.9
2022-08-08 4:00 AM	15.1	14.6	14.6	14.9
2022-08-08 5:00 AM	15.0	14.5	14.5	14.8
2022-08-08 6:00 AM	15.0	14.5	14.5	14.8
2022-08-08 7:00 AM	14.9	14.6	14.6	14.8
2022-08-08 8:00 AM	14.9	14.4	14.4	14.6
2022-08-08 9:00 AM	15.0	14.6	14.6	14.8
2022-08-08 10:00 AM	15.3	15.0	15.0	15.2
2022-08-08 11:00 AM	15.9	15.7	15.7	15.8
2022-08-08 12:00 PM	18.0	18.0	18.0	18.0
2022-08-08 1:00 PM	19.6	20.4	20.4	20.0
2022-08-08 2:00 PM	20.7	21.1	21.1	20.9
2022-08-08 3:00 PM	19.9	21.3	21.3	20.6
2022-08-08 4:00 PM	19.4	20.9	20.9	20.1
2022-08-08 5:00 PM	19.4	20.8	20.8	20.1
2022-08-08 6:00 PM	19.1	19.7	19.7	19.4
2022-08-08 7:00 PM	18.7	19.1	19.1	18.9
2022-08-08 8:00 PM	18.1	18.2	18.2	18.2
2022-08-08 9:00 PM	17.7	17.7	17.7	17.7
2022-08-08 10:00 PM	17.3	17.1	17.1	17.2
2022-08-08 11:00 PM	16.8	16.6	16.6	16.7
2022-08-09 12:00 AM	16.3	15.9	15.9	16.1
2022-08-09 1:00 AM	16.0	15.6	15.6	15.8
2022-08-09 2:00 AM	15.7	15.2	15.2	15.4
2022-08-09 3:00 AM	15.4	14.9	14.9	15.1
2022-08-09 4:00 AM	15.2	14.5	14.5	14.8
2022-08-09 5:00 AM	15.0	14.3	14.3	14.7
2022-08-09 6:00 AM	14.8	14.1	14.1	14.5
2022-08-09 7:00 AM	14.3	14.0	14.0	14.2

2022-08-09 8:00 AM	14.3	13.6	13.6	14.0
2022-08-09 9:00 AM	14.8	14.3	14.3	14.6
2022-08-09 10:00 AM	15.9	15.4	15.4	15.6
2022-08-09 11:00 AM	16.5	16.2	16.2	16.4
2022-08-09 12:00 PM	18.4	18.5	18.5	18.4
2022-08-09 1:00 PM	19.8	21.1	21.1	20.4
2022-08-09 2:00 PM	21.8	22.9	22.9	22.3
2022-08-09 3:00 PM	21.6	23.7	23.7	22.7
2022-08-09 4:00 PM	21.4	24.1	24.1	22.8
2022-08-09 5:00 PM	21.5	23.7	23.7	22.6
2022-08-09 6:00 PM	21.7	23.1	23.1	22.4
2022-08-09 7:00 PM	20.9	21.7	21.7	21.3
2022-08-09 8:00 PM	20.6	21.2	21.2	20.9
2022-08-09 9:00 PM	20.3	20.7	20.7	20.5
2022-08-09 10:00 PM	19.8	20.1	20.1	19.9
2022-08-09 11:00 PM	19.5	19.7	19.7	19.6
2022-08-10 12:00 AM	19.1	19.3	19.3	19.2
2022-08-10 1:00 AM	18.7	18.8	18.8	18.8
2022-08-10 2:00 AM	18.3	18.3	18.3	18.3
2022-08-10 3:00 AM	17.7	17.5	17.5	17.6
2022-08-10 4:00 AM	17.0	16.6	16.6	16.8
2022-08-10 5:00 AM	16.6	16.0	16.0	16.3
2022-08-10 6:00 AM	16.6	16.3	16.3	16.4
2022-08-10 7:00 AM	16.3	15.9	15.9	16.1
2022-08-10 8:00 AM	16.1	15.8	15.8	16.0
2022-08-10 9:00 AM	16.3	16.0	16.0	16.1
2022-08-10 10:00 AM	16.8	16.7	16.7	16.8
2022-08-10 11:00 AM	17.3	17.4	17.4	17.3
2022-08-10 12:00 PM	18.5	19.0	19.0	18.8
2022-08-10 1:00 PM	19.1	20.0	20.0	19.6
2022-08-10 2:00 PM	19.4	20.9	20.9	20.1
2022-08-10 3:00 PM	20.0	21.2	21.2	20.6
2022-08-10 4:00 PM	19.5	20.5	20.5	20.0
2022-08-10 5:00 PM	19.9	21.0	21.0	20.4
2022-08-10 6:00 PM	19.8	20.4	20.4	20.1
2022-08-10 7:00 PM	18.8	19.1	19.1	18.9
2022-08-10 8:00 PM	18.5	18.7	18.7	18.6
2022-08-10 9:00 PM	18.1	18.3	18.3	18.2
2022-08-10 10:00 PM	17.5	17.6	17.6	17.6
2022-08-10 11:00 PM	17.1	16.9	16.9	17.0
2022-08-11 12:00 AM	16.6	16.3	16.3	16.4
2022-08-11 1:00 AM	16.2	15.9	15.9	16.1
2022-08-11 2:00 AM	15.8	15.3	15.3	15.6

2022-08-11 3:00 AM	15.4	14.8	14.8	15.1
2022-08-11 4:00 AM	15.0	14.2	14.2	14.6
2022-08-11 5:00 AM	14.6	13.6	13.6	14.1
2022-08-11 6:00 AM	14.3	13.1	13.1	13.7
2022-08-11 7:00 AM	13.8	12.7	12.7	13.3
2022-08-11 8:00 AM	13.4	12.6	12.6	13.0
2022-08-11 9:00 AM	13.8	13.1	13.1	13.5
2022-08-11 10:00 AM	14.7	14.2	14.2	14.4
2022-08-11 11:00 AM	15.6	15.4	15.4	15.5
2022-08-11 12:00 PM	17.6	17.7	17.7	17.7
2022-08-11 1:00 PM	19.3	20.5	20.5	19.9
2022-08-11 2:00 PM	20.7	21.6	21.6	21.2
2022-08-11 3:00 PM	20.1	21.9	21.9	21.0
2022-08-11 4:00 PM	20.2	22.6	22.6	21.4
2022-08-11 5:00 PM	20.0	21.7	21.7	20.9
2022-08-11 6:00 PM	19.9	20.6	20.6	20.3
2022-08-11 7:00 PM	18.6	18.9	18.9	18.8
2022-08-11 8:00 PM	18.0	18.1	18.1	18.1
2022-08-11 9:00 PM	17.5	17.4	17.4	17.4
2022-08-11 10:00 PM	16.9	16.6	16.6	16.8
2022-08-11 11:00 PM	16.4	16.1	16.1	16.3
2022-08-12 12:00 AM	15.9	15.6	15.6	15.8
2022-08-12 1:00 AM	15.4	14.7	14.7	15.0
2022-08-12 2:00 AM	15.0	14.1	14.1	14.6
2022-08-12 3:00 AM	14.6	13.6	13.6	14.1
2022-08-12 4:00 AM	14.2	13.1	13.1	13.7
2022-08-12 5:00 AM	13.8	12.7	12.7	13.3
2022-08-12 6:00 AM	13.4	12.4	12.4	12.9
2022-08-12 7:00 AM	13.1	12.1	12.1	12.6
2022-08-12 8:00 AM	12.9	11.9	11.9	12.4
2022-08-12 9:00 AM	12.8	12.1	12.1	12.5
2022-08-12 10:00 AM	14.2	13.6	13.6	13.9
2022-08-12 11:00 AM	15.3	15.1	15.1	15.2
2022-08-12 12:00 PM	16.6	16.5	16.5	16.6
2022-08-12 1:00 PM	17.4	18.3	18.3	17.8
2022-08-12 2:00 PM	18.4	19.5	19.5	18.9
2022-08-12 3:00 PM	17.7	19.4	19.4	18.6
2022-08-12 4:00 PM	17.7	20.0	20.0	18.9
2022-08-12 5:00 PM	17.9	19.8	19.8	18.8
2022-08-12 6:00 PM	17.9	18.9	18.9	18.4
2022-08-12 7:00 PM	17.2	17.5	17.5	17.4
2022-08-12 8:00 PM	16.6	16.7	16.7	16.7
2022-08-12 9:00 PM	16.3	16.3	16.3	16.3

2022-08-12 10:00 PM	16.0	15.9	15.9	15.9
2022-08-12 11:00 PM	15.7	15.4	15.4	15.5
2022-08-13 12:00 AM	15.4	15.1	15.1	15.3
2022-08-13 1:00 AM	15.2	14.8	14.8	15.0
2022-08-13 2:00 AM	14.9	14.4	14.4	14.6
2022-08-13 3:00 AM	14.8	14.4	14.4	14.6
2022-08-13 4:00 AM	15.0	14.6	14.6	14.8
2022-08-13 5:00 AM	15.1	14.8	14.8	15.0
2022-08-13 6:00 AM	15.2	15.0	15.0	15.1
2022-08-13 7:00 AM	15.3	15.1	15.1	15.2
2022-08-13 8:00 AM	15.3	15.2	15.2	15.3
2022-08-13 9:00 AM	15.5	15.4	15.4	15.4
2022-08-13 10:00 AM	15.9	15.9	15.9	15.9
2022-08-13 11:00 AM	16.1	16.1	16.1	16.1
2022-08-13 12:00 PM	17.0	17.4	17.4	17.2
2022-08-13 1:00 PM	18.9	20.3	20.3	19.6
2022-08-13 2:00 PM	20.2	21.5	21.5	20.9
2022-08-13 3:00 PM	19.9	22.0	22.0	20.9
2022-08-13 4:00 PM	19.4	21.8	21.8	20.6
2022-08-13 5:00 PM	19.3	21.2	21.2	20.3
2022-08-13 6:00 PM	19.1	20.0	20.0	19.6
2022-08-13 7:00 PM	17.9	18.1	18.1	18.0
2022-08-13 8:00 PM	17.2	17.2	17.2	17.2
2022-08-13 9:00 PM	16.7	16.6	16.6	16.7
2022-08-13 10:00 PM	16.5	16.4	16.4	16.4
2022-08-13 11:00 PM	16.5	16.3	16.3	16.4
2022-08-14 12:00 AM	16.3	16.1	16.1	16.2
2022-08-14 1:00 AM	16.2	16.0	16.0	16.1
2022-08-14 2:00 AM	16.2	16.0	16.0	16.1
2022-08-14 3:00 AM	16.1	16.0	16.0	16.1
2022-08-14 4:00 AM	16.1	16.0	16.0	16.1
2022-08-14 5:00 AM	16.1	16.1	16.1	16.1
2022-08-14 6:00 AM	16.0	16.0	16.0	16.0
2022-08-14 7:00 AM	16.0	16.0	16.0	16.0
2022-08-14 8:00 AM	16.1	16.0	16.0	16.1
2022-08-14 9:00 AM	16.1	16.1	16.1	16.1
2022-08-14 10:00 AM	16.2	16.1	16.1	16.2
2022-08-14 11:00 AM	16.4	16.4	16.4	16.4
2022-08-14 12:00 PM	16.5	16.5	16.5	16.5
2022-08-14 1:00 PM	17.0	17.0	17.0	17.0
2022-08-14 2:00 PM	18.1	18.2	18.2	18.2
2022-08-14 3:00 PM	18.6	19.0	19.0	18.8
2022-08-14 4:00 PM	18.4	18.6	18.6	18.5

2022-08-14 5:00 PM	18.3	19.1	19.1	18.7
2022-08-14 6:00 PM	18.2	19.0	19.0	18.6
2022-08-14 7:00 PM	17.5	17.7	17.7	17.6
2022-08-14 8:00 PM	17.2	17.3	17.3	17.3
2022-08-14 9:00 PM	16.8	16.7	16.7	16.8
2022-08-14 10:00 PM	16.2	16.1	16.1	16.2
2022-08-14 11:00 PM	16.0	15.8	15.8	15.9
2022-08-15 12:00 AM	15.9	15.7	15.7	15.8
2022-08-15 1:00 AM	16.0	15.9	15.9	15.9
2022-08-15 2:00 AM	16.1	16.0	16.0	16.1
2022-08-15 3:00 AM	16.0	15.9	15.9	15.9
2022-08-15 4:00 AM	16.1	16.0	16.0	16.1
2022-08-15 5:00 AM	16.1	16.0	16.0	16.1
2022-08-15 6:00 AM	16.0	15.9	15.9	15.9
2022-08-15 7:00 AM	16.0	15.9	15.9	15.9
2022-08-15 8:00 AM	16.0	16.0	16.0	16.0
2022-08-15 9:00 AM	16.2	16.1	16.1	16.2
2022-08-15 10:00 AM	16.6	16.6	16.6	16.6
2022-08-15 11:00 AM	17.2	17.3	17.3	17.3
2022-08-15 12:00 PM	18.5	18.4	18.4	18.4
2022-08-15 1:00 PM	19.8	20.4	20.4	20.1
2022-08-15 2:00 PM	20.5	21.1	21.1	20.8
2022-08-15 3:00 PM	20.1	21.6	21.6	20.9
2022-08-15 4:00 PM	19.9	21.8	21.8	20.8
2022-08-15 5:00 PM	19.9	21.3	21.3	20.6
2022-08-15 6:00 PM	19.9	20.4	20.4	20.1
2022-08-15 7:00 PM	18.6	18.8	18.8	18.7
2022-08-15 8:00 PM	18.0	18.1	18.1	18.1
2022-08-15 9:00 PM	17.5	17.6	17.6	17.6
2022-08-15 10:00 PM	17.0	17.1	17.1	17.1
2022-08-15 11:00 PM	16.4	16.5	16.5	16.4
2022-08-16 12:00 AM	16.1	16.2	16.2	16.2
2022-08-16 1:00 AM	15.9	15.7	15.7	15.8
2022-08-16 2:00 AM	15.5	15.2	15.2	15.3
2022-08-16 3:00 AM	15.2	14.5	14.5	14.8
2022-08-16 4:00 AM	14.9	14.2	14.2	14.5
2022-08-16 5:00 AM	14.4	14.2	14.2	14.3
2022-08-16 6:00 AM	14.3	13.9	13.9	14.1
2022-08-16 7:00 AM	14.0	13.7	13.7	13.8
2022-08-16 8:00 AM	14.0	13.6	13.6	13.8
2022-08-16 9:00 AM	14.6	14.3	14.3	14.5
2022-08-16 10:00 AM	15.5	15.2	15.2	15.3
2022-08-16 11:00 AM	16.5	16.1	16.1	16.3

2022-08-16 12:00 PM	18.9	17.8	17.8	18.3
2022-08-16 1:00 PM	21.0	19.9	19.9	20.4
2022-08-16 2:00 PM	22.1	20.7	20.7	21.4
2022-08-16 3:00 PM	21.2	21.6	21.6	21.4
2022-08-16 4:00 PM	20.4	21.9	21.9	21.1
2022-08-16 5:00 PM	20.0	20.9	20.9	20.4
2022-08-16 6:00 PM	19.8	20.0	20.0	19.9
2022-08-16 7:00 PM	18.8	18.8	18.8	18.8
2022-08-16 8:00 PM	18.0	18.1	18.1	18.1
2022-08-16 9:00 PM	17.8	17.8	17.8	17.8
2022-08-16 10:00 PM	17.5	17.4	17.4	17.4
2022-08-16 11:00 PM	17.0	17.0	17.0	17.0
2022-08-17 12:00 AM	16.7	16.7	16.7	16.7
2022-08-17 1:00 AM	16.9	16.8	16.8	16.8
2022-08-17 2:00 AM	17.0	16.9	16.9	16.9
2022-08-17 3:00 AM	17.1	17.0	17.0	17.1
2022-08-17 4:00 AM	17.0	16.9	16.9	16.9
2022-08-17 5:00 AM	16.7	16.7	16.7	16.7
2022-08-17 6:00 AM	16.5	16.5	16.5	16.5
2022-08-17 7:00 AM	16.4	16.3	16.3	16.3
2022-08-17 8:00 AM	16.5	16.3	16.3	16.4
2022-08-17 9:00 AM	16.9	16.7	16.7	16.8
2022-08-17 10:00 AM	17.2	17.1	17.1	17.2
2022-08-17 11:00 AM	17.8	17.6	17.6	17.7
2022-08-17 12:00 PM	19.1	18.5	18.5	18.8
2022-08-17 1:00 PM	19.9	19.4	19.4	19.6
2022-08-17 2:00 PM	20.5	20.4	20.4	20.4
2022-08-17 3:00 PM	20.6	20.7	20.7	20.7
2022-08-17 4:00 PM	20.7	21.5	21.5	21.1
2022-08-17 5:00 PM	20.0	20.3	20.3	20.1
2022-08-17 6:00 PM	19.2	19.3	19.3	19.3
2022-08-17 7:00 PM	19.0	19.2	19.2	19.1
2022-08-17 8:00 PM	18.5	18.5	18.5	18.5
2022-08-17 9:00 PM	18.1	18.1	18.1	18.1
2022-08-17 10:00 PM	17.7	17.6	17.6	17.7
2022-08-17 11:00 PM	17.3	17.4	17.4	17.3
2022-08-18 12:00 AM	17.0	17.1	17.1	17.1
2022-08-18 1:00 AM	17.1	17.0	17.0	17.1
2022-08-18 2:00 AM	17.2	17.2	17.2	17.2
2022-08-18 3:00 AM	17.0	17.1	17.1	17.1
2022-08-18 4:00 AM	16.8	16.9	16.9	16.8
2022-08-18 5:00 AM	16.6	16.7	16.7	16.7
2022-08-18 6:00 AM	16.2	16.4	16.4	16.3

2022-08-18 7:00 AM	16.1	16.3	16.3	16.2
2022-08-18 8:00 AM	16.2	16.3	16.3	16.3
2022-08-18 9:00 AM	16.6	16.6	16.6	16.6
2022-08-18 10:00 AM	17.4	17.4	17.4	17.4
2022-08-18 11:00 AM	18.4	18.3	18.3	18.3
2022-08-18 12:00 PM	21.0	20.3	20.3	20.6
2022-08-18 1:00 PM	23.7	22.6	22.6	23.2
2022-08-18 2:00 PM	22.6	22.2	22.2	22.4
2022-08-18 3:00 PM	20.7	20.8	20.8	20.8
2022-08-18 4:00 PM	19.7	19.7	19.7	19.7
2022-08-18 5:00 PM	19.6	19.6	19.6	19.6
2022-08-18 6:00 PM	19.1	19.2	19.2	19.2
2022-08-18 7:00 PM	19.0	19.1	19.1	19.1
2022-08-18 8:00 PM	19.0	19.1	19.1	19.1
2022-08-18 9:00 PM	18.4	18.6	18.6	18.5
2022-08-18 10:00 PM	17.9	18.1	18.1	18.0
2022-08-18 11:00 PM	17.6	17.9	17.9	17.8
2022-08-19 12:00 AM	17.4	17.6	17.6	17.5
2022-08-19 1:00 AM	17.5	17.5	17.5	17.5
2022-08-19 2:00 AM	17.6	17.7	17.7	17.7
2022-08-19 3:00 AM	17.6	17.6	17.6	17.6
2022-08-19 4:00 AM	17.6	17.7	17.7	17.7
2022-08-19 5:00 AM	17.6	17.7	17.7	17.7
2022-08-19 6:00 AM	17.6	17.6	17.6	17.6
2022-08-19 7:00 AM	17.5	17.5	17.5	17.5
2022-08-19 8:00 AM	17.4	17.4	17.4	17.4
2022-08-19 9:00 AM	17.6	17.5	17.5	17.6
2022-08-19 10:00 AM	17.8	17.7	17.7	17.8
2022-08-19 11:00 AM	18.3	18.1	18.1	18.2
2022-08-19 12:00 PM	19.0	18.7	18.7	18.9
2022-08-19 1:00 PM	20.3	19.7	19.7	20.0
2022-08-19 2:00 PM	22.4	21.3	21.3	21.8
2022-08-19 3:00 PM	21.7	22.2	22.2	22.0
2022-08-19 4:00 PM	21.5	22.6	22.6	22.1
2022-08-19 5:00 PM	21.6	22.4	22.4	22.0
2022-08-19 6:00 PM	20.8	21.0	21.0	20.9
2022-08-19 7:00 PM	20.2	20.2	20.2	20.2
2022-08-19 8:00 PM	20.0	20.0	20.0	20.0
2022-08-19 9:00 PM	19.5	19.6	19.6	19.6
2022-08-19 10:00 PM	19.0	19.2	19.2	19.1
2022-08-19 11:00 PM	18.6	18.7	18.7	18.7
2022-08-20 12:00 AM	18.3	18.4	18.4	18.3
2022-08-20 1:00 AM	18.0	18.0	18.0	18.0

2022-08-20 2:00 AM	18.0	18.0	18.0	18.0
2022-08-20 3:00 AM	18.0	18.0	18.0	18.0
2022-08-20 4:00 AM	17.8	17.7	17.7	17.8
2022-08-20 5:00 AM	17.8	17.6	17.6	17.7
2022-08-20 6:00 AM	17.6	17.5	17.5	17.6
2022-08-20 7:00 AM	17.2	17.2	17.2	17.2
2022-08-20 8:00 AM	16.9	16.9	16.9	16.9
2022-08-20 9:00 AM	17.2	16.9	16.9	17.1
2022-08-20 10:00 AM	17.4	17.1	17.1	17.3
2022-08-20 11:00 AM	18.1	17.7	17.7	17.9
2022-08-20 12:00 PM	20.1	19.1	19.1	19.6
2022-08-20 1:00 PM	19.8	19.2	19.2	19.5
2022-08-20 2:00 PM	21.4	20.4	20.4	20.9
2022-08-20 3:00 PM	21.2	21.5	21.5	21.4
2022-08-20 4:00 PM	20.6	22.0	22.0	21.3
2022-08-20 5:00 PM	20.6	21.5	21.5	21.1
2022-08-20 6:00 PM	20.5	20.6	20.6	20.6
2022-08-20 7:00 PM	19.6	19.5	19.5	19.6
2022-08-20 8:00 PM	18.7	18.6	18.6	18.7
2022-08-20 9:00 PM	18.2	18.1	18.1	18.2
2022-08-20 10:00 PM	17.6	17.6	17.6	17.6
2022-08-20 11:00 PM	17.1	17.1	17.1	17.1
2022-08-21 12:00 AM	16.6	16.2	16.2	16.4
2022-08-21 1:00 AM	16.1	15.6	15.6	15.9
2022-08-21 2:00 AM	15.5	15.4	15.4	15.4
2022-08-21 3:00 AM	15.1	14.9	14.9	15.0
2022-08-21 4:00 AM	14.6	14.5	14.5	14.6
2022-08-21 5:00 AM	14.2	14.3	14.3	14.3
2022-08-21 6:00 AM	14.0	14.0	14.0	14.0
2022-08-21 7:00 AM	13.7	13.8	13.8	13.8
2022-08-21 8:00 AM	13.6	13.6	13.6	13.6
2022-08-21 9:00 AM	14.1	14.0	14.0	14.1
2022-08-21 10:00 AM	15.2	15.0	15.0	15.1
2022-08-21 11:00 AM	16.5	16.0	16.0	16.3
2022-08-21 12:00 PM	19.6	18.2	18.2	18.9
2022-08-21 1:00 PM	21.7	19.9	19.9	20.8
2022-08-21 2:00 PM	22.6	21.2	21.2	21.9
2022-08-21 3:00 PM	21.3	21.8	21.8	21.5
2022-08-21 4:00 PM	20.6	22.0	22.0	21.3
2022-08-21 5:00 PM	20.4	21.4	21.4	20.9
2022-08-21 6:00 PM	20.2	20.6	20.6	20.4
2022-08-21 7:00 PM	19.2	19.4	19.4	19.3
2022-08-21 8:00 PM	18.7	18.7	18.7	18.7

2022-08-21 9:00 PM	18.1	18.1	18.1	18.1
2022-08-21 10:00 PM	17.6	17.6	17.6	17.6
2022-08-21 11:00 PM	17.3	17.4	17.4	17.3
2022-08-22 12:00 AM	16.9	17.1	17.1	17.0
2022-08-22 1:00 AM	16.6	16.6	16.6	16.6
2022-08-22 2:00 AM	16.3	16.1	16.1	16.2
2022-08-22 3:00 AM	16.0	15.8	15.8	15.9
2022-08-22 4:00 AM	15.6	15.7	15.7	15.7
2022-08-22 5:00 AM	15.2	15.3	15.3	15.3
2022-08-22 6:00 AM	15.1	15.2	15.2	15.2
2022-08-22 7:00 AM	14.8	15.0	15.0	14.9
2022-08-22 8:00 AM	14.7	14.8	14.8	14.8
2022-08-22 9:00 AM	15.1	15.1	15.1	15.1
2022-08-22 10:00 AM	16.5	16.3	16.3	16.4
2022-08-22 11:00 AM	17.8	17.5	17.5	17.6
2022-08-22 12:00 PM	19.8	19.0	19.0	19.4
2022-08-22 1:00 PM	22.1	20.6	20.6	21.4
2022-08-22 2:00 PM	21.0	20.6	20.6	20.8
2022-08-22 3:00 PM	21.0	21.7	21.7	21.4
2022-08-22 4:00 PM	20.0	20.3	20.3	20.1
2022-08-22 5:00 PM	18.9	18.9	18.9	18.9
2022-08-22 6:00 PM	19.1	19.1	19.1	19.1
2022-08-22 7:00 PM	19.3	19.3	19.3	19.3
2022-08-22 8:00 PM	19.0	19.2	19.2	19.1
2022-08-22 9:00 PM	18.6	18.7	18.7	18.7
2022-08-22 10:00 PM	18.2	18.2	18.2	18.2
2022-08-22 11:00 PM	18.3	18.2	18.2	18.3
2022-08-23 12:00 AM	18.2	18.2	18.2	18.2
2022-08-23 1:00 AM	18.0	18.1	18.1	18.1
2022-08-23 2:00 AM	18.0	18.0	18.0	18.0
2022-08-23 3:00 AM	18.0	18.0	18.0	18.0
2022-08-23 4:00 AM	18.0	18.0	18.0	18.0
2022-08-23 5:00 AM	17.9	17.9	17.9	17.9
2022-08-23 6:00 AM	17.7	17.7	17.7	17.7
2022-08-23 7:00 AM	17.5	17.5	17.5	17.5
2022-08-23 8:00 AM	17.4	17.4	17.4	17.4
2022-08-23 9:00 AM	17.7	17.6	17.6	17.7
2022-08-23 10:00 AM	18.0	17.9	17.9	17.9
2022-08-23 11:00 AM	18.7	18.5	18.5	18.6
2022-08-23 12:00 PM	19.2	19.0	19.0	19.1
2022-08-23 1:00 PM	20.7	19.9	19.9	20.3
2022-08-23 2:00 PM	22.0	21.2	21.2	21.6
2022-08-23 3:00 PM	21.3	21.3	21.3	21.3

2022-08-23 4:00 PM	20.9	21.5	21.5	21.2
2022-08-23 5:00 PM	21.3	22.2	22.2	21.8
2022-08-23 6:00 PM	21.2	21.9	21.9	21.6
2022-08-23 7:00 PM	20.2	20.5	20.5	20.4
2022-08-23 8:00 PM	19.4	19.6	19.6	19.5
2022-08-23 9:00 PM	18.6	18.8	18.8	18.7
2022-08-23 10:00 PM	18.3	18.4	18.4	18.3
2022-08-23 11:00 PM	17.8	18.0	18.0	17.9
2022-08-24 12:00 AM	17.5	17.6	17.6	17.6
2022-08-24 1:00 AM	17.2	17.3	17.3	17.3
2022-08-24 2:00 AM	16.9	16.8	16.8	16.8
2022-08-24 3:00 AM	16.4	16.5	16.5	16.4
2022-08-24 4:00 AM	16.0	16.0	16.0	16.0
2022-08-24 5:00 AM	16.0	16.0	16.0	16.0
2022-08-24 6:00 AM	16.1	16.0	16.0	16.1
2022-08-24 7:00 AM	16.2	16.0	16.0	16.1
2022-08-24 8:00 AM	16.2	16.1	16.1	16.2
2022-08-24 9:00 AM	16.4	16.2	16.2	16.3
2022-08-24 10:00 AM	16.6	16.3	16.3	16.4
2022-08-24 11:00 AM	16.9	16.7	16.7	16.8
2022-08-24 12:00 PM	17.2	17.0	17.0	17.1
2022-08-24 1:00 PM	17.2	17.0	17.0	17.1
2022-08-24 2:00 PM	17.3	17.1	17.1	17.2
2022-08-24 3:00 PM	17.3	16.9	16.9	17.1
2022-08-24 4:00 PM	17.3	16.9	16.9	17.1
2022-08-24 5:00 PM	17.5	17.0	17.0	17.3
2022-08-24 6:00 PM	17.6	17.3	17.3	17.4
2022-08-24 7:00 PM	17.6	17.4	17.4	17.5
2022-08-24 8:00 PM	17.5	17.3	17.3	17.4
2022-08-24 9:00 PM	17.2	17.2	17.2	17.2
2022-08-24 10:00 PM	17.1	17.0	17.0	17.1
2022-08-24 11:00 PM	17.0	16.8	16.8	16.9
2022-08-25 12:00 AM	16.6	16.5	16.5	16.6
2022-08-25 1:00 AM	16.3	16.1	16.1	16.2
2022-08-25 2:00 AM	16.0	15.6	15.6	15.8
2022-08-25 3:00 AM	15.9	15.4	15.4	15.6
2022-08-25 4:00 AM	15.9	15.4	15.4	15.6
2022-08-25 5:00 AM	15.8	15.3	15.3	15.6
2022-08-25 6:00 AM	15.4	14.8	14.8	15.1
2022-08-25 7:00 AM	15.5	14.9	14.9	15.2
2022-08-25 8:00 AM	15.3	14.7	14.7	15.0
2022-08-25 9:00 AM	15.2	14.6	14.6	14.9
2022-08-25 10:00 AM	15.9	15.2	15.2	15.5

2022-08-25 11:00 AM	16.4	15.8	15.8	16.1
2022-08-25 12:00 PM	17.4	16.7	16.7	17.1
2022-08-25 1:00 PM	20.2	18.8	18.8	19.5
2022-08-25 2:00 PM	20.1	19.2	19.2	19.7
2022-08-25 3:00 PM	19.8	20.0	20.0	19.9
2022-08-25 4:00 PM	19.7	20.1	20.1	19.9
2022-08-25 5:00 PM	19.0	18.8	18.8	18.9
2022-08-25 6:00 PM	18.7	18.5	18.5	18.6
2022-08-25 7:00 PM	18.4	18.2	18.2	18.3
2022-08-25 8:00 PM	18.0	17.8	17.8	17.9
2022-08-25 9:00 PM	17.6	17.5	17.5	17.6
2022-08-25 10:00 PM	17.3	17.2	17.2	17.3
2022-08-25 11:00 PM	17.2	17.1	17.1	17.2
2022-08-26 12:00 AM	16.9	16.9	16.9	16.9
2022-08-26 1:00 AM	16.8	16.7	16.7	16.8
2022-08-26 2:00 AM	16.8	16.7	16.7	16.8
2022-08-26 3:00 AM	16.4	16.4	16.4	16.4
2022-08-26 4:00 AM	15.9	15.9	15.9	15.9
2022-08-26 5:00 AM	15.9	15.9	15.9	15.9
2022-08-26 6:00 AM	15.8	15.8	15.8	15.8
2022-08-26 7:00 AM	15.6	15.6	15.6	15.6
2022-08-26 8:00 AM	15.8	15.8	15.8	15.8
2022-08-26 9:00 AM	16.0	16.0	16.0	16.0
2022-08-26 10:00 AM	16.4	16.3	16.3	16.3
2022-08-26 11:00 AM	17.2	16.9	16.9	17.1
2022-08-26 12:00 PM	19.4	18.7	18.7	19.1
2022-08-26 1:00 PM	20.8	19.9	19.9	20.3
2022-08-26 2:00 PM	21.6	21.1	21.1	21.4
2022-08-26 3:00 PM	21.6	21.9	21.9	21.8
2022-08-26 4:00 PM	21.2	21.7	21.7	21.5
2022-08-26 5:00 PM	21.2	21.8	21.8	21.5
2022-08-26 6:00 PM	21.0	21.5	21.5	21.3
2022-08-26 7:00 PM	20.5	20.9	20.9	20.7
2022-08-26 8:00 PM	20.1	20.4	20.4	20.3
2022-08-26 9:00 PM	19.6	19.8	19.8	19.7
2022-08-26 10:00 PM	18.9	19.1	19.1	19.0
2022-08-26 11:00 PM	18.6	18.8	18.8	18.7
2022-08-27 12:00 AM	18.2	18.5	18.5	18.4
2022-08-27 1:00 AM	17.8	18.0	18.0	17.9
2022-08-27 2:00 AM	17.6	17.7	17.7	17.7
2022-08-27 3:00 AM	17.4	17.5	17.5	17.4
2022-08-27 4:00 AM	17.1	17.2	17.2	17.2
2022-08-27 5:00 AM	16.9	16.9	16.9	16.9

2022-08-27 6:00 AM	16.6	16.6	16.6	16.6
2022-08-27 7:00 AM	16.4	16.3	16.3	16.3
2022-08-27 8:00 AM	16.2	16.0	16.0	16.1
2022-08-27 9:00 AM	16.5	16.3	16.3	16.4
2022-08-27 10:00 AM	17.4	17.3	17.3	17.3
2022-08-27 11:00 AM	18.4	18.3	18.3	18.3
2022-08-27 12:00 PM	19.8	19.5	19.5	19.6
2022-08-27 1:00 PM	21.0	20.2	20.2	20.6
2022-08-27 2:00 PM	20.1	19.7	19.7	19.9
2022-08-27 3:00 PM	19.9	19.5	19.5	19.7
2022-08-27 4:00 PM	19.3	19.0	19.0	19.1
2022-08-27 5:00 PM	18.7	18.5	18.5	18.6
2022-08-27 6:00 PM	18.7	18.5	18.5	18.6
2022-08-27 7:00 PM	18.5	18.5	18.5	18.5
2022-08-27 8:00 PM	18.5	18.5	18.5	18.5
2022-08-27 9:00 PM	18.4	18.4	18.4	18.4
2022-08-27 10:00 PM	18.4	18.3	18.3	18.3
2022-08-27 11:00 PM	18.4	18.3	18.3	18.3
2022-08-28 12:00 AM	18.2	18.2	18.2	18.2
2022-08-28 1:00 AM	18.0	18.1	18.1	18.1
2022-08-28 2:00 AM	18.2	18.3	18.3	18.3
2022-08-28 3:00 AM	18.2	18.3	18.3	18.3
2022-08-28 4:00 AM	18.2	18.3	18.3	18.3
2022-08-28 5:00 AM	18.2	18.3	18.3	18.3
2022-08-28 6:00 AM	18.1	18.2	18.2	18.2
2022-08-28 7:00 AM	17.8	17.9	17.9	17.8
2022-08-28 8:00 AM	17.8	17.9	17.9	17.8
2022-08-28 9:00 AM	18.0	18.1	18.1	18.1
2022-08-28 10:00 AM	18.2	18.3	18.3	18.3
2022-08-28 11:00 AM	18.9	18.9	18.9	18.9
2022-08-28 12:00 PM	19.8	19.6	19.6	19.7
2022-08-28 1:00 PM	20.4	20.2	20.2	20.3
2022-08-28 2:00 PM	20.0	19.9	19.9	19.9
2022-08-28 3:00 PM	20.0	20.0	20.0	20.0
2022-08-28 4:00 PM	20.2	20.2	20.2	20.2
2022-08-28 5:00 PM	19.8	19.8	19.8	19.8
2022-08-28 6:00 PM	19.6	19.6	19.6	19.6
2022-08-28 7:00 PM	19.7	19.7	19.7	19.7
2022-08-28 8:00 PM	19.5	19.6	19.6	19.6
2022-08-28 9:00 PM	19.1	19.2	19.2	19.2
2022-08-28 10:00 PM	18.9	19.0	19.0	18.9
2022-08-28 11:00 PM	18.7	18.7	18.7	18.7
2022-08-29 12:00 AM	18.3	18.5	18.5	18.4

2022-08-29 1:00 AM	18.0	18.1	18.1	18.1
2022-08-29 2:00 AM	17.8	18.0	18.0	17.9
2022-08-29 3:00 AM	18.0	18.0	18.0	18.0
2022-08-29 4:00 AM	18.0	18.1	18.1	18.1
2022-08-29 5:00 AM	18.0	18.1	18.1	18.1
2022-08-29 6:00 AM	18.0	18.1	18.1	18.1
2022-08-29 7:00 AM	18.1	18.1	18.1	18.1
2022-08-29 8:00 AM	17.9	18.0	18.0	17.9
2022-08-29 9:00 AM	18.2	18.2	18.2	18.2
2022-08-29 10:00 AM	18.3	18.4	18.4	18.3
2022-08-29 11:00 AM	19.2	19.1	19.1	19.2
2022-08-29 12:00 PM	21.6	20.9	20.9	21.3
2022-08-29 1:00 PM	22.5	21.6	21.6	22.1
2022-08-29 2:00 PM	23.1	22.1	22.1	22.6
2022-08-29 3:00 PM	22.0	22.5	22.5	22.3
2022-08-29 4:00 PM	21.4	22.7	22.7	22.1
2022-08-29 5:00 PM	21.3	21.9	21.9	21.6
2022-08-29 6:00 PM	20.9	21.1	21.1	21.0
2022-08-29 7:00 PM	20.4	20.4	20.4	20.4
2022-08-29 8:00 PM	20.1	20.1	20.1	20.1
2022-08-29 9:00 PM	19.1	19.2	19.2	19.2
2022-08-29 10:00 PM	18.7	18.8	18.8	18.8
2022-08-29 11:00 PM	18.4	18.5	18.5	18.4
2022-08-30 12:00 AM	18.4	18.5	18.5	18.4
2022-08-30 1:00 AM	18.0	18.1	18.1	18.1
2022-08-30 2:00 AM	17.5	17.7	17.7	17.6
2022-08-30 3:00 AM	17.4	17.5	17.5	17.4
2022-08-30 4:00 AM	17.4	17.4	17.4	17.4
2022-08-30 5:00 AM	17.4	17.4	17.4	17.4
2022-08-30 6:00 AM	17.1	17.2	17.2	17.2
2022-08-30 7:00 AM	16.7	16.7	16.7	16.7
2022-08-30 8:00 AM	16.0	15.9	15.9	15.9
2022-08-30 9:00 AM	15.4	15.3	15.3	15.3
2022-08-30 10:00 AM	15.6	15.3	15.3	15.5
2022-08-30 11:00 AM	16.4	16.0	16.0	16.2
2022-08-30 12:00 PM	18.3	17.8	17.8	18.0
2022-08-30 1:00 PM	19.9	19.2	19.2	19.6
2022-08-30 2:00 PM	20.3	20.0	20.0	20.1
2022-08-30 3:00 PM	19.6	19.4	19.4	19.5
2022-08-30 4:00 PM	19.6	19.6	19.6	19.6
2022-08-30 5:00 PM	19.8	19.8	19.8	19.8
2022-08-30 6:00 PM	19.3	19.1	19.1	19.2
2022-08-30 7:00 PM	18.9	18.9	18.9	18.9

2022-08-30 8:00 PM	18.4	18.5	18.5	18.4
2022-08-30 9:00 PM	17.8	17.8	17.8	17.8
2022-08-30 10:00 PM	17.1	17.2	17.2	17.2
2022-08-30 11:00 PM	16.7	16.8	16.8	16.8
2022-08-31 12:00 AM	16.4	16.5	16.5	16.4
2022-08-31 1:00 AM	16.2	16.3	16.3	16.3
2022-08-31 2:00 AM	16.0	16.1	16.1	16.1
2022-08-31 3:00 AM	15.8	15.8	15.8	15.8
2022-08-31 4:00 AM	15.5	15.6	15.6	15.6
2022-08-31 5:00 AM	15.4	15.5	15.5	15.4
2022-08-31 6:00 AM	15.1	15.3	15.3	15.2
2022-08-31 7:00 AM	14.9	15.1	15.1	15.0
2022-08-31 8:00 AM	14.5	14.7	14.7	14.6
2022-08-31 9:00 AM	14.3	14.5	14.5	14.4
2022-08-31 10:00 AM	14.4	14.3	14.3	14.3
2022-08-31 11:00 AM	15.0	14.7	14.7	14.8
2022-08-31 12:00 PM	16.8	16.4	16.4	16.6
2022-08-31 1:00 PM	18.5	17.9	17.9	18.2
2022-08-31 2:00 PM	19.0	18.7	18.7	18.9
2022-08-31 3:00 PM	17.2	18.3	18.3	17.8
2022-08-31 4:00 PM	16.4	18.7	18.7	17.6
2022-08-31 5:00 PM	17.2	18.6	18.6	17.9
2022-08-31 6:00 PM	17.4	18.3	18.3	17.8
2022-08-31 7:00 PM	16.8	17.4	17.4	17.1
2022-08-31 8:00 PM	16.3	16.7	16.7	16.5
2022-08-31 9:00 PM	15.9	16.0	16.0	15.9
2022-08-31 10:00 PM	15.6	15.5	15.5	15.6
2022-08-31 11:00 PM	15.3	15.3	15.3	15.3
2022-09-01 12:00 AM	15.2	15.2	15.2	15.2
2022-09-01 1:00 AM	15.4	15.4	15.4	15.4
2022-09-01 2:00 AM	15.3	15.3	15.3	15.3
2022-09-01 3:00 AM	14.7	14.7	14.7	14.7
2022-09-01 4:00 AM	14.5	14.5	14.5	14.5
2022-09-01 5:00 AM	14.5	14.4	14.4	14.4
2022-09-01 6:00 AM	14.3	14.3	14.3	14.3
2022-09-01 7:00 AM	14.0	14.0	14.0	14.0
2022-09-01 8:00 AM	14.0	13.8	13.8	13.9
2022-09-01 9:00 AM	14.2	14.0	14.0	14.1
2022-09-01 10:00 AM	15.1	14.8	14.8	15.0
2022-09-01 11:00 AM	15.9	15.5	15.5	15.7
2022-09-01 12:00 PM	17.1	16.6	16.6	16.9
2022-09-01 1:00 PM	19.5	18.2	18.2	18.9
2022-09-01 2:00 PM	20.4	19.7	19.7	20.1

2022-09-01 3:00 PM	19.3	19.8	19.8	19.5
2022-09-01 4:00 PM	19.1	20.1	20.1	19.6
2022-09-01 5:00 PM	19.2	19.9	19.9	19.6
2022-09-01 6:00 PM	19.2	19.7	19.7	19.5
2022-09-01 7:00 PM	18.3	18.6	18.6	18.4
2022-09-01 8:00 PM	17.8	18.0	18.0	17.9
2022-09-01 9:00 PM	17.3	17.5	17.5	17.4
2022-09-01 10:00 PM	16.9	17.1	17.1	17.0
2022-09-01 11:00 PM	16.4	16.7	16.7	16.6
2022-09-02 12:00 AM	16.0	16.3	16.3	16.1
2022-09-02 1:00 AM	15.7	16.0	16.0	15.8
2022-09-02 2:00 AM	15.4	15.7	15.7	15.5
2022-09-02 3:00 AM	15.2	15.3	15.3	15.3
2022-09-02 4:00 AM	15.1	15.2	15.2	15.2
2022-09-02 5:00 AM	15.0	15.0	15.0	15.0
2022-09-02 6:00 AM	14.9	14.9	14.9	14.9
2022-09-02 7:00 AM	14.8	14.8	14.8	14.8
2022-09-02 8:00 AM	15.0	15.1	15.1	15.1
2022-09-02 9:00 AM	15.6	15.6	15.6	15.6
2022-09-02 10:00 AM	16.3	16.3	16.3	16.3
2022-09-02 11:00 AM	17.9	17.6	17.6	17.8
2022-09-02 12:00 PM	18.7	18.6	18.6	18.7
2022-09-02 1:00 PM	19.2	19.1	19.1	19.2
2022-09-02 2:00 PM	21.5	21.3	21.3	21.4
2022-09-02 3:00 PM	21.8	22.1	22.1	21.9
2022-09-02 4:00 PM	21.3	21.6	21.6	21.4
2022-09-02 5:00 PM	20.9	21.7	21.7	21.3
2022-09-02 6:00 PM	20.7	21.6	21.6	21.2
2022-09-02 7:00 PM	20.0	20.8	20.8	20.4
2022-09-02 8:00 PM	19.2	20.2	20.2	19.7
2022-09-02 9:00 PM	18.4	19.0	19.0	18.7
2022-09-02 10:00 PM	17.8	18.1	18.1	17.9
2022-09-02 11:00 PM	17.1	17.3	17.3	17.2
2022-09-03 12:00 AM	16.6	16.8	16.8	16.7
2022-09-03 1:00 AM	16.1	16.2	16.2	16.2
2022-09-03 2:00 AM	15.5	15.5	15.5	15.5
2022-09-03 3:00 AM	14.8	14.6	14.6	14.7
2022-09-03 4:00 AM	14.2	13.8	13.8	14.0
2022-09-03 5:00 AM	13.4	12.9	12.9	13.1
2022-09-03 6:00 AM	12.9	13.0	13.0	12.9
2022-09-03 7:00 AM	12.2	12.6	12.6	12.4
2022-09-03 8:00 AM	11.9	12.3	12.3	12.1
2022-09-03 9:00 AM	12.0	12.1	12.1	12.1

2022-09-03 10:00 AM	12.4	12.0	12.0	12.2
2022-09-03 11:00 AM	13.0	12.2	12.2	12.6
2022-09-03 12:00 PM	14.1	13.9	13.9	14.0
2022-09-03 1:00 PM	16.7	15.4	15.4	16.1
2022-09-03 2:00 PM	17.4	16.5	16.5	16.9
2022-09-03 3:00 PM	16.9	17.4	17.4	17.1
2022-09-03 4:00 PM	17.0	18.0	18.0	17.5
2022-09-03 5:00 PM	16.9	17.5	17.5	17.2
2022-09-03 6:00 PM	16.5	16.7	16.7	16.6
2022-09-03 7:00 PM	15.6	15.7	15.7	15.7
2022-09-03 8:00 PM	15.1	15.3	15.3	15.2
2022-09-03 9:00 PM	14.4	14.7	14.7	14.5
2022-09-03 10:00 PM	13.8	13.9	13.9	13.8
2022-09-03 11:00 PM	13.2	13.6	13.6	13.4
2022-09-04 12:00 AM	12.7	12.9	12.9	12.8
2022-09-04 1:00 AM	12.1	12.1	12.1	12.1
2022-09-04 2:00 AM	11.5	11.9	11.9	11.7
2022-09-04 3:00 AM	11.1	11.1	11.1	11.1
2022-09-04 4:00 AM	10.5	10.9	10.9	10.7
2022-09-04 5:00 AM	10.1	10.1	10.1	10.1
2022-09-04 6:00 AM	9.7	9.9	9.9	9.8
2022-09-04 7:00 AM	9.1	9.7	9.7	9.4
2022-09-04 8:00 AM	9.0	9.7	9.7	9.3
2022-09-04 9:00 AM	9.5	9.7	9.7	9.6
2022-09-04 10:00 AM	10.6	10.6	10.6	10.6
2022-09-04 11:00 AM	12.4	12.1	12.1	12.3
2022-09-04 12:00 PM	14.4	14.8	14.8	14.6
2022-09-04 1:00 PM	17.3	16.4	16.4	16.8
2022-09-04 2:00 PM	17.7	17.2	17.2	17.5
2022-09-04 3:00 PM	17.1	17.8	17.8	17.4
2022-09-04 4:00 PM	16.8	17.9	17.9	17.3
2022-09-04 5:00 PM	16.3	17.0	17.0	16.6
2022-09-04 6:00 PM	15.9	16.2	16.2	16.1
2022-09-04 7:00 PM	14.9	15.2	15.2	15.0
2022-09-04 8:00 PM	14.2	14.6	14.6	14.4
2022-09-04 9:00 PM	13.4	13.9	13.9	13.6
2022-09-04 10:00 PM	13.0	13.2	13.2	13.1
2022-09-04 11:00 PM	12.4	12.6	12.6	12.5
2022-09-05 12:00 AM	11.8	11.9	11.9	11.8
2022-09-05 1:00 AM	11.3	11.2	11.2	11.3
2022-09-05 2:00 AM	10.8	10.7	10.7	10.8
2022-09-05 3:00 AM	10.4	10.5	10.5	10.4
2022-09-05 4:00 AM	10.1	9.8	9.8	10.0

2022-09-05 5:00 AM	9.6	9.8	9.8	9.7
2022-09-05 6:00 AM	9.0	9.7	9.7	9.3
2022-09-05 7:00 AM	8.8	9.3	9.3	9.1
2022-09-05 8:00 AM	8.6	9.2	9.2	8.9
2022-09-05 9:00 AM	8.9	9.3	9.3	9.1
2022-09-05 10:00 AM	9.8	10.0	10.0	9.9
2022-09-05 11:00 AM	11.7	11.6	11.6	11.7
2022-09-05 12:00 PM	13.6	14.2	14.2	13.9
2022-09-05 1:00 PM	16.7	16.0	16.0	16.4
2022-09-05 2:00 PM	17.3	17.0	17.0	17.1
2022-09-05 3:00 PM	16.8	17.6	17.6	17.2
2022-09-05 4:00 PM	16.7	17.8	17.8	17.3
2022-09-05 5:00 PM	16.7	17.4	17.4	17.1
2022-09-05 6:00 PM	16.3	16.7	16.7	16.5
2022-09-05 7:00 PM	15.3	15.7	15.7	15.5
2022-09-05 8:00 PM	14.7	15.2	15.2	14.9
2022-09-05 9:00 PM	14.1	14.6	14.6	14.4
2022-09-05 10:00 PM	13.5	14.0	14.0	13.8
2022-09-05 11:00 PM	13.2	13.4	13.4	13.3
2022-09-06 12:00 AM	12.8	13.0	13.0	12.9
2022-09-06 1:00 AM	12.4	12.6	12.6	12.5
2022-09-06 2:00 AM	12.0	11.9	11.9	11.9
2022-09-06 3:00 AM	11.7	11.7	11.7	11.7
2022-09-06 4:00 AM	11.3	11.6	11.6	11.5
2022-09-06 5:00 AM	10.8	11.3	11.3	11.1
2022-09-06 6:00 AM	10.3	10.9	10.9	10.6
2022-09-06 7:00 AM	10.0	10.7	10.7	10.3
2022-09-06 8:00 AM	9.8	10.5	10.5	10.2
2022-09-06 9:00 AM	10.1	10.6	10.6	10.4
2022-09-06 10:00 AM	11.2	11.3	11.3	11.3
2022-09-06 11:00 AM	12.8	12.6	12.6	12.7
2022-09-06 12:00 PM	14.9	15.2	15.2	15.0
2022-09-06 1:00 PM	17.4	16.9	16.9	17.1
2022-09-06 2:00 PM	18.1	18.1	18.1	18.1
2022-09-06 3:00 PM	17.1	18.1	18.1	17.6
2022-09-06 4:00 PM	17.7	19.0	19.0	18.4
2022-09-06 5:00 PM	17.8	18.8	18.8	18.3
2022-09-06 6:00 PM	17.6	18.2	18.2	17.9
2022-09-06 7:00 PM	16.9	17.3	17.3	17.1
2022-09-06 8:00 PM	16.5	16.8	16.8	16.6
2022-09-06 9:00 PM	16.0	16.3	16.3	16.1
2022-09-06 10:00 PM	15.6	16.0	16.0	15.8
2022-09-06 11:00 PM	15.4	15.7	15.7	15.5

2022-09-07 12:00 AM	15.2	15.5	15.5	15.3
2022-09-07 1:00 AM	14.9	15.3	15.3	15.1
2022-09-07 2:00 AM	14.6	15.0	15.0	14.8
2022-09-07 3:00 AM	14.1	14.6	14.6	14.4
2022-09-07 4:00 AM	13.9	14.3	14.3	14.1
2022-09-07 5:00 AM	13.7	14.2	14.2	13.9
2022-09-07 6:00 AM	13.5	14.0	14.0	13.8
2022-09-07 7:00 AM	13.2	13.7	13.7	13.4
2022-09-07 8:00 AM	13.0	13.5	13.5	13.3
2022-09-07 9:00 AM	13.2	13.6	13.6	13.4
2022-09-07 10:00 AM	14.2	14.3	14.3	14.3
2022-09-07 11:00 AM	15.5	15.4	15.4	15.4
2022-09-07 12:00 PM	18.0	17.9	17.9	17.9
2022-09-07 1:00 PM	20.2	19.7	19.7	20.0
2022-09-07 2:00 PM	21.2	20.9	20.9	21.1
2022-09-07 3:00 PM	20.5	21.1	21.1	20.8
2022-09-07 4:00 PM	20.5	21.4	21.4	20.9
2022-09-07 5:00 PM	20.0	20.5	20.5	20.3
2022-09-07 6:00 PM	19.2	19.5	19.5	19.4
2022-09-07 7:00 PM	18.2	18.3	18.3	18.3
2022-09-07 8:00 PM	17.3	17.4	17.4	17.3
2022-09-07 9:00 PM	16.3	16.5	16.5	16.4
2022-09-07 10:00 PM	15.7	15.9	15.9	15.8
2022-09-07 11:00 PM	15.0	15.2	15.2	15.1
2022-09-08 12:00 AM	14.4	14.5	14.5	14.4
2022-09-08 1:00 AM	13.8	13.7	13.7	13.8
2022-09-08 2:00 AM	13.2	12.9	12.9	13.0
2022-09-08 3:00 AM	12.8	12.4	12.4	12.6
2022-09-08 4:00 AM	12.3	12.1	12.1	12.2
2022-09-08 5:00 AM	12.0	11.9	11.9	11.9
2022-09-08 6:00 AM	11.7	11.5	11.5	11.6
2022-09-08 7:00 AM	11.8	11.5	11.5	11.7
2022-09-08 8:00 AM	11.8	11.9	11.9	11.8
2022-09-08 9:00 AM	12.3	12.3	12.3	12.3
2022-09-08 10:00 AM	14.1	13.8	13.8	14.0
2022-09-08 11:00 AM	15.3	15.0	15.0	15.2
2022-09-08 12:00 PM	17.6	17.1	17.1	17.4
2022-09-08 1:00 PM	18.4	18.1	18.1	18.3
2022-09-08 2:00 PM	18.6	18.4	18.4	18.5
2022-09-08 3:00 PM	18.3	18.2	18.2	18.3
2022-09-08 4:00 PM	18.7	18.6	18.6	18.7
2022-09-08 5:00 PM	18.5	18.4	18.4	18.4
2022-09-08 6:00 PM	18.3	18.2	18.2	18.3

2022-09-08 7:00 PM	18.0	18.0	18.0	18.0
2022-09-08 8:00 PM	17.8	17.8	17.8	17.8
2022-09-08 9:00 PM	17.4	17.6	17.6	17.5
2022-09-08 10:00 PM	17.5	17.7	17.7	17.6
2022-09-08 11:00 PM	17.4	17.5	17.5	17.4
2022-09-09 12:00 AM	17.4	17.6	17.6	17.5
2022-09-09 1:00 AM	17.3	17.4	17.4	17.3
2022-09-09 2:00 AM	17.3	17.4	17.4	17.3
2022-09-09 3:00 AM	17.3	17.5	17.5	17.4
2022-09-09 4:00 AM	17.5	17.7	17.7	17.6
2022-09-09 5:00 AM	17.3	17.5	17.5	17.4
2022-09-09 6:00 AM	17.2	17.3	17.3	17.3
2022-09-09 7:00 AM	16.9	17.1	17.1	17.0
2022-09-09 8:00 AM	16.6	16.7	16.7	16.7
2022-09-09 9:00 AM	16.3	16.5	16.5	16.4
2022-09-09 10:00 AM	16.3	16.3	16.3	16.3
2022-09-09 11:00 AM	16.2	16.1	16.1	16.2
2022-09-09 12:00 PM	15.9	16.0	16.0	15.9
2022-09-09 1:00 PM	15.6	15.6	15.6	15.6
2022-09-09 2:00 PM	15.6	15.4	15.4	15.5
2022-09-09 3:00 PM	15.5	15.5	15.5	15.5
2022-09-09 4:00 PM	15.5	15.4	15.4	15.4
2022-09-09 5:00 PM	15.5	15.6	15.6	15.6
2022-09-09 6:00 PM	15.4	15.6	15.6	15.5
2022-09-09 7:00 PM	15.4	15.5	15.5	15.4
2022-09-09 8:00 PM	15.2	15.4	15.4	15.3
2022-09-09 9:00 PM	15.0	15.2	15.2	15.1
2022-09-09 10:00 PM	14.9	15.1	15.1	15.0
2022-09-09 11:00 PM	14.8	15.1	15.1	15.0
2022-09-10 12:00 AM	14.7	14.9	14.9	14.8
2022-09-10 1:00 AM	14.6	14.9	14.9	14.8
2022-09-10 2:00 AM	14.6	14.8	14.8	14.7
2022-09-10 3:00 AM	14.5	14.7	14.7	14.6
2022-09-10 4:00 AM	14.3	14.6	14.6	14.5
2022-09-10 5:00 AM	14.1	14.4	14.4	14.3
2022-09-10 6:00 AM	13.7	14.2	14.2	13.9
2022-09-10 7:00 AM	13.7	14.0	14.0	13.8
2022-09-10 8:00 AM	13.4	13.9	13.9	13.6
2022-09-10 9:00 AM	13.6	14.0	14.0	13.8
2022-09-10 10:00 AM	14.4	14.4	14.4	14.4
2022-09-10 11:00 AM	14.8	14.8	14.8	14.8
2022-09-10 12:00 PM	15.4	15.3	15.3	15.3
2022-09-10 1:00 PM	16.4	16.1	16.1	16.3

2022-09-10 2:00 PM	16.7	16.4	16.4	16.6
2022-09-10 3:00 PM	17.3	17.4	17.4	17.3
2022-09-10 4:00 PM	17.8	18.0	18.0	17.9
2022-09-10 5:00 PM	17.1	17.0	17.0	17.1
2022-09-10 6:00 PM	16.7	16.5	16.5	16.6
2022-09-10 7:00 PM	16.1	16.1	16.1	16.1
2022-09-10 8:00 PM	15.5	15.6	15.6	15.6
2022-09-10 9:00 PM	14.7	15.0	15.0	14.8
2022-09-10 10:00 PM	14.3	14.5	14.5	14.4
2022-09-10 11:00 PM	13.8	13.9	13.9	13.8
2022-09-11 12:00 AM	13.5	13.7	13.7	13.6
2022-09-11 1:00 AM	12.9	13.3	13.3	13.1
2022-09-11 2:00 AM	12.6	12.9	12.9	12.8
2022-09-11 3:00 AM	12.0	12.5	12.5	12.3
2022-09-11 4:00 AM	11.6	12.2	12.2	11.9
2022-09-11 5:00 AM	11.1	11.8	11.8	11.5
2022-09-11 6:00 AM	10.8	11.6	11.6	11.2
2022-09-11 7:00 AM	10.4	11.0	11.0	10.7
2022-09-11 8:00 AM	10.2	10.8	10.8	10.5
2022-09-11 9:00 AM	10.5	11.0	11.0	10.8
2022-09-11 10:00 AM	11.5	11.7	11.7	11.6
2022-09-11 11:00 AM	13.1	13.0	13.0	13.1
2022-09-11 12:00 PM	16.8	15.4	15.4	16.1
2022-09-11 1:00 PM	17.6	16.9	16.9	17.3
2022-09-11 2:00 PM	18.2	17.9	17.9	18.1
2022-09-11 3:00 PM	17.8	18.1	18.1	17.9
2022-09-11 4:00 PM	17.7	17.8	17.8	17.8
2022-09-11 5:00 PM	17.6	17.7	17.7	17.7
2022-09-11 6:00 PM	17.4	17.3	17.3	17.3
2022-09-11 7:00 PM	16.5	16.4	16.4	16.4
2022-09-11 8:00 PM	15.8	15.9	15.9	15.8
2022-09-11 9:00 PM	14.9	15.1	15.1	15.0
2022-09-11 10:00 PM	14.4	14.6	14.6	14.5
2022-09-11 11:00 PM	13.8	14.2	14.2	14.0
2022-09-12 12:00 AM	13.2	13.4	13.4	13.3
2022-09-12 1:00 AM	12.5	13.0	13.0	12.8
2022-09-12 2:00 AM	12.0	12.5	12.5	12.3
2022-09-12 3:00 AM	11.4	11.9	11.9	11.6
2022-09-12 4:00 AM	11.0	11.7	11.7	11.3
2022-09-12 5:00 AM	10.7	11.3	11.3	11.0
2022-09-12 6:00 AM	10.5	11.0	11.0	10.8
2022-09-12 7:00 AM	10.2	10.9	10.9	10.5
2022-09-12 8:00 AM	10.0	10.7	10.7	10.3

2022-09-12 9:00 AM	10.2	10.8	10.8	10.5
2022-09-12 10:00 AM	11.4	11.6	11.6	11.5
2022-09-12 11:00 AM	12.8	12.8	12.8	12.8
2022-09-12 12:00 PM	16.3	15.0	15.0	15.6
2022-09-12 1:00 PM	17.9	17.1	17.1	17.5
2022-09-12 2:00 PM	18.5	18.2	18.2	18.4
2022-09-12 3:00 PM	18.2	18.8	18.8	18.5
2022-09-12 4:00 PM	17.8	18.3	18.3	18.0
2022-09-12 5:00 PM	17.4	17.8	17.8	17.6
2022-09-12 6:00 PM	17.2	17.2	17.2	17.2
2022-09-12 7:00 PM	16.5	16.6	16.6	16.6
2022-09-12 8:00 PM	16.0	16.1	16.1	16.1
2022-09-12 9:00 PM	15.4	15.6	15.6	15.5
2022-09-12 10:00 PM	14.8	15.1	15.1	15.0
2022-09-12 11:00 PM	14.3	14.6	14.6	14.5
2022-09-13 12:00 AM	13.8	13.9	13.9	13.8
2022-09-13 1:00 AM	13.4	13.4	13.4	13.4
2022-09-13 2:00 AM	12.9	13.0	13.0	12.9
2022-09-13 3:00 AM	12.5	12.8	12.8	12.7
2022-09-13 4:00 AM	11.9	12.5	12.5	12.2
2022-09-13 5:00 AM	11.7	12.3	12.3	12.0
2022-09-13 6:00 AM	11.4	11.9	11.9	11.6
2022-09-13 7:00 AM	11.3	11.8	11.8	11.6
2022-09-13 8:00 AM	11.3	11.8	11.8	11.6
2022-09-13 9:00 AM	12.1	12.3	12.3	12.2
2022-09-13 10:00 AM	13.1	13.0	13.0	13.1
2022-09-13 11:00 AM	14.0	13.9	13.9	13.9
2022-09-13 12:00 PM	16.3	15.2	15.2	15.7
2022-09-13 1:00 PM	16.5	16.1	16.1	16.3
2022-09-13 2:00 PM	16.5	16.3	16.3	16.4
2022-09-13 3:00 PM	16.3	16.5	16.5	16.4
2022-09-13 4:00 PM	15.8	15.9	15.9	15.8
2022-09-13 5:00 PM	15.4	15.3	15.3	15.3
2022-09-13 6:00 PM	15.0	14.9	14.9	14.9
2022-09-13 7:00 PM	14.7	14.7	14.7	14.7
2022-09-13 8:00 PM	14.1	14.2	14.2	14.2
2022-09-13 9:00 PM	13.2	13.4	13.4	13.3
2022-09-13 10:00 PM	12.9	12.9	12.9	12.9
2022-09-13 11:00 PM	12.5	12.3	12.3	12.4
2022-09-14 12:00 AM	11.8	11.9	11.9	11.8
2022-09-14 1:00 AM	11.0	11.4	11.4	11.2
2022-09-14 2:00 AM	10.5	11.0	11.0	10.8
2022-09-14 3:00 AM	10.3	10.8	10.8	10.6

2022-09-14 4:00 AM	10.2	10.2	10.2	10.2
2022-09-14 5:00 AM	9.3	9.9	9.9	9.6
2022-09-14 6:00 AM	9.1	9.6	9.6	9.4
2022-09-14 7:00 AM	8.5	9.2	9.2	8.8
2022-09-14 8:00 AM	8.2	9.0	9.0	8.6
2022-09-14 9:00 AM	8.4	8.9	8.9	8.6
2022-09-14 10:00 AM	9.3	9.6	9.6	9.5
2022-09-14 11:00 AM	11.0	10.9	10.9	10.9
2022-09-14 12:00 PM	14.4	12.6	12.6	13.5
2022-09-14 1:00 PM	15.5	14.1	14.1	14.8
2022-09-14 2:00 PM	15.5	14.6	14.6	15.1
2022-09-14 3:00 PM	15.1	15.3	15.3	15.2
2022-09-14 4:00 PM	14.8	15.0	15.0	14.9
2022-09-14 5:00 PM	14.4	14.7	14.7	14.5
2022-09-14 6:00 PM	13.8	13.9	13.9	13.8
2022-09-14 7:00 PM	13.0	13.1	13.1	13.1
2022-09-14 8:00 PM	12.7	12.8	12.8	12.8
2022-09-14 9:00 PM	12.1	12.4	12.4	12.3
2022-09-14 10:00 PM	11.4	11.8	11.8	11.6
2022-09-14 11:00 PM	11.2	11.2	11.2	11.2
2022-09-15 12:00 AM	10.8	10.5	10.5	10.7
2022-09-15 1:00 AM	10.6	10.4	10.4	10.5
2022-09-15 2:00 AM	10.8	11.0	11.0	10.9
2022-09-15 3:00 AM	11.3	11.3	11.3	11.3
2022-09-15 4:00 AM	11.5	11.5	11.5	11.5
2022-09-15 5:00 AM	11.7	11.7	11.7	11.7
2022-09-15 6:00 AM	11.9	11.9	11.9	11.9
2022-09-15 7:00 AM	12.0	12.0	12.0	12.0
2022-09-15 8:00 AM	12.0	12.0	12.0	12.0
2022-09-15 9:00 AM	12.3	12.3	12.3	12.3
2022-09-15 10:00 AM	12.4	12.4	12.4	12.4
2022-09-15 11:00 AM	12.5	12.6	12.6	12.6
2022-09-15 12:00 PM	12.8	12.8	12.8	12.8
2022-09-15 1:00 PM	13.1	13.1	13.1	13.1
2022-09-15 2:00 PM	13.2	13.2	13.2	13.2
2022-09-15 3:00 PM	13.4	13.4	13.4	13.4
2022-09-15 4:00 PM	13.6	13.6	13.6	13.6
2022-09-15 5:00 PM	13.6	13.6	13.6	13.6
2022-09-15 6:00 PM	13.8	13.8	13.8	13.8
2022-09-15 7:00 PM	13.7	13.9	13.9	13.8
2022-09-15 8:00 PM	13.7	13.9	13.9	13.8
2022-09-15 9:00 PM	13.7	13.8	13.8	13.8
2022-09-15 10:00 PM	13.7	13.8	13.8	13.8

2022-09-15 11:00 PM	13.7	13.9	13.9	13.8
2022-09-16 12:00 AM	13.6	13.7	13.7	13.7
2022-09-16 1:00 AM	13.5	13.6	13.6	13.6
2022-09-16 2:00 AM	13.5	13.6	13.6	13.6
2022-09-16 3:00 AM	13.4	13.4	13.4	13.4
2022-09-16 4:00 AM	13.1	13.1	13.1	13.1
2022-09-16 5:00 AM	12.9	12.9	12.9	12.9
2022-09-16 6:00 AM	12.8	12.8	12.8	12.8
2022-09-16 7:00 AM	12.7	12.7	12.7	12.7
2022-09-16 8:00 AM	12.5	12.3	12.3	12.4
2022-09-16 9:00 AM	12.4	12.3	12.3	12.3
2022-09-16 10:00 AM	12.5	12.4	12.4	12.4
2022-09-16 11:00 AM	12.6	12.5	12.5	12.6
2022-09-16 12:00 PM	12.7	12.6	12.6	12.7
2022-09-16 1:00 PM	12.9	12.7	12.7	12.8
2022-09-16 2:00 PM	13.0	12.9	12.9	12.9
2022-09-16 3:00 PM	12.9	12.8	12.8	12.8
2022-09-16 4:00 PM	12.9	12.9	12.9	12.9
2022-09-16 5:00 PM	13.0	12.9	12.9	12.9
2022-09-16 6:00 PM	13.0	13.0	13.0	13.0
2022-09-16 7:00 PM	13.0	13.0	13.0	13.0
2022-09-16 8:00 PM	13.0	13.0	13.0	13.0
2022-09-16 9:00 PM	13.1	13.2	13.2	13.2
2022-09-16 10:00 PM	13.3	13.3	13.3	13.3
2022-09-16 11:00 PM	13.4	13.5	13.5	13.4
2022-09-17 12:00 AM	13.5	13.6	13.6	13.6
2022-09-17 1:00 AM	13.6	13.7	13.7	13.7
2022-09-17 2:00 AM	13.6	13.8	13.8	13.7
2022-09-17 3:00 AM	13.7	13.8	13.8	13.8
2022-09-17 4:00 AM	13.7	13.9	13.9	13.8
2022-09-17 5:00 AM	13.7	13.9	13.9	13.8
2022-09-17 6:00 AM	13.8	13.9	13.9	13.8
2022-09-17 7:00 AM	13.8	13.9	13.9	13.8
2022-09-17 8:00 AM	13.9	13.9	13.9	13.9
2022-09-17 9:00 AM	14.0	14.1	14.1	14.1
2022-09-17 10:00 AM	14.3	14.3	14.3	14.3
2022-09-17 11:00 AM	14.6	14.6	14.6	14.6
2022-09-17 12:00 PM	14.9	14.9	14.9	14.9
2022-09-17 1:00 PM	15.3	15.2	15.2	15.3
2022-09-17 2:00 PM	15.8	15.5	15.5	15.7
2022-09-17 3:00 PM	15.8	15.6	15.6	15.7
2022-09-17 4:00 PM	15.7	15.6	15.6	15.7
2022-09-17 5:00 PM	15.6	15.5	15.5	15.6

2022-09-17 6:00 PM	15.5	15.4	15.4	15.4
2022-09-17 7:00 PM	15.4	15.4	15.4	15.4
2022-09-17 8:00 PM	15.2	15.3	15.3	15.3
2022-09-17 9:00 PM	15.2	15.3	15.3	15.3
2022-09-17 10:00 PM	15.2	15.3	15.3	15.3
2022-09-17 11:00 PM	15.1	15.2	15.2	15.2
2022-09-18 12:00 AM	15.1	15.2	15.2	15.2
2022-09-18 1:00 AM	15.2	15.3	15.3	15.3
2022-09-18 2:00 AM	15.1	15.2	15.2	15.2
2022-09-18 3:00 AM	15.1	15.2	15.2	15.2
2022-09-18 4:00 AM	15.1	15.2	15.2	15.2
2022-09-18 5:00 AM	15.1	15.2	15.2	15.2
2022-09-18 6:00 AM	15.1	15.2	15.2	15.2
2022-09-18 7:00 AM	15.1	15.2	15.2	15.2
2022-09-18 8:00 AM	15.0	15.1	15.1	15.1
2022-09-18 9:00 AM	15.0	15.0	15.0	15.0
2022-09-18 10:00 AM	15.0	15.1	15.1	15.1
2022-09-18 11:00 AM	15.3	15.3	15.3	15.3
2022-09-18 12:00 PM	15.5	15.5	15.5	15.5
2022-09-18 1:00 PM	16.2	16.0	16.0	16.1
2022-09-18 2:00 PM	17.0	16.7	16.7	16.9
2022-09-18 3:00 PM	17.2	17.0	17.0	17.1
2022-09-18 4:00 PM	17.4	17.3	17.3	17.3
2022-09-18 5:00 PM	17.1	17.0	17.0	17.1
2022-09-18 6:00 PM	17.1	16.9	16.9	17.0
2022-09-18 7:00 PM	16.9	16.8	16.8	16.8
2022-09-18 8:00 PM	16.5	16.5	16.5	16.5
2022-09-18 9:00 PM	16.1	16.2	16.2	16.2
2022-09-18 10:00 PM	15.6	15.8	15.8	15.7
2022-09-18 11:00 PM	15.0	15.2	15.2	15.1
2022-09-19 12:00 AM	14.6	14.8	14.8	14.7
2022-09-19 1:00 AM	14.2	14.5	14.5	14.3
2022-09-19 2:00 AM	13.9	14.3	14.3	14.1
2022-09-19 3:00 AM	13.6	13.9	13.9	13.8
2022-09-19 4:00 AM	13.3	13.6	13.6	13.5
2022-09-19 5:00 AM	13.3	13.6	13.6	13.5
2022-09-19 6:00 AM	13.1	13.4	13.4	13.3
2022-09-19 7:00 AM	12.9	13.2	13.2	13.0
2022-09-19 8:00 AM	12.8	13.1	13.1	13.0
2022-09-19 9:00 AM	12.9	13.2	13.2	13.0
2022-09-19 10:00 AM	13.7	13.8	13.8	13.8
2022-09-19 11:00 AM	14.8	14.7	14.7	14.8
2022-09-19 12:00 PM	17.2	15.8	15.8	16.5

2022-09-19 1:00 PM	18.3	17.2	17.2	17.8
2022-09-19 2:00 PM	19.1	19.1	19.1	19.1
2022-09-19 3:00 PM	18.7	19.4	19.4	19.1
2022-09-19 4:00 PM	18.3	18.5	18.5	18.4
2022-09-19 5:00 PM	17.9	18.2	18.2	18.1
2022-09-19 6:00 PM	17.5	17.6	17.6	17.6
2022-09-19 7:00 PM	16.7	16.9	16.9	16.8
2022-09-19 8:00 PM	16.3	16.5	16.5	16.4
2022-09-19 9:00 PM	15.7	16.0	16.0	15.8
2022-09-19 10:00 PM	15.4	15.6	15.6	15.5
2022-09-19 11:00 PM	15.1	15.4	15.4	15.3
2022-09-20 12:00 AM	15.2	15.3	15.3	15.3
2022-09-20 1:00 AM	15.2	15.3	15.3	15.3
2022-09-20 2:00 AM	15.1	15.2	15.2	15.2
2022-09-20 3:00 AM	15.0	15.1	15.1	15.1
2022-09-20 4:00 AM	15.0	15.2	15.2	15.1
2022-09-20 5:00 AM	15.0	15.1	15.1	15.1
2022-09-20 6:00 AM	14.8	15.0	15.0	14.9
2022-09-20 7:00 AM	15.0	15.1	15.1	15.1
2022-09-20 8:00 AM	15.1	15.1	15.1	15.1
2022-09-20 9:00 AM	15.1	15.2	15.2	15.2
2022-09-20 10:00 AM	15.4	15.4	15.4	15.4
2022-09-20 11:00 AM	16.0	15.8	15.8	15.9
2022-09-20 12:00 PM	17.5	16.8	16.8	17.1
2022-09-20 1:00 PM	19.1	18.2	18.2	18.7
2022-09-20 2:00 PM	19.5	19.9	19.9	19.7
2022-09-20 3:00 PM	19.6	20.5	20.5	20.1
2022-09-20 4:00 PM	19.1	19.3	19.3	19.2
2022-09-20 5:00 PM	18.4	18.5	18.5	18.4
2022-09-20 6:00 PM	17.8	17.9	17.9	17.8
2022-09-20 7:00 PM	17.7	17.8	17.8	17.8
2022-09-20 8:00 PM	17.4	17.6	17.6	17.5
2022-09-20 9:00 PM	17.1	17.2	17.2	17.2
2022-09-20 10:00 PM	16.6	16.7	16.7	16.7
2022-09-20 11:00 PM	16.5	16.7	16.7	16.6
2022-09-21 12:00 AM	16.5	16.6	16.6	16.6
2022-09-21 1:00 AM	16.4	16.5	16.5	16.4
2022-09-21 2:00 AM	16.2	16.3	16.3	16.3
2022-09-21 3:00 AM	16.0	16.0	16.0	16.0
2022-09-21 4:00 AM	15.7	15.8	15.8	15.8
2022-09-21 5:00 AM	15.4	15.6	15.6	15.5
2022-09-21 6:00 AM	15.1	15.3	15.3	15.2
2022-09-21 7:00 AM	14.5	14.7	14.7	14.6

2022-09-21 8:00 AM	14.0	14.2	14.2	14.1
2022-09-21 9:00 AM	13.9	14.1	14.1	14.0
2022-09-21 10:00 AM	14.4	14.5	14.5	14.4
2022-09-21 11:00 AM	14.7	14.5	14.5	14.6
2022-09-21 12:00 PM	15.0	14.4	14.4	14.7
2022-09-21 1:00 PM	14.5	13.9	13.9	14.2
2022-09-21 2:00 PM	14.9	14.5	14.5	14.7
2022-09-21 3:00 PM	13.7	13.3	13.3	13.5
2022-09-21 4:00 PM	13.2	12.7	12.7	12.9
2022-09-21 5:00 PM	13.0	12.7	12.7	12.8
2022-09-21 6:00 PM	12.3	11.9	11.9	12.1
2022-09-21 7:00 PM	12.0	11.5	11.5	11.8
2022-09-21 8:00 PM	11.8	11.3	11.3	11.6
2022-09-21 9:00 PM	11.5	11.0	11.0	11.3
2022-09-21 10:00 PM	11.5	11.3	11.3	11.4
2022-09-21 11:00 PM	11.5	11.4	11.4	11.4
2022-09-22 12:00 AM	11.3	11.3	11.3	11.3
2022-09-22 1:00 AM	11.0	10.8	10.8	10.9
2022-09-22 2:00 AM	10.7	10.1	10.1	10.4
2022-09-22 3:00 AM	10.4	10.0	10.0	10.2
2022-09-22 4:00 AM	9.9	10.1	10.1	10.0
2022-09-22 5:00 AM	10.0	9.6	9.6	9.8
2022-09-22 6:00 AM	10.0	10.0	10.0	10.0
2022-09-22 7:00 AM	10.1	9.6	9.6	9.9
2022-09-22 8:00 AM	9.4	8.7	8.7	9.0
2022-09-22 9:00 AM	9.2	8.8	8.8	9.0
2022-09-22 10:00 AM	9.0	8.3	8.3	8.7
2022-09-22 11:00 AM	9.2	8.5	8.5	8.8
2022-09-22 12:00 PM	11.0	9.8	9.8	10.4
2022-09-22 1:00 PM	11.8	10.7	10.7	11.3
2022-09-22 2:00 PM	12.1	12.1	12.1	12.1
2022-09-22 3:00 PM	12.1	12.9	12.9	12.5
2022-09-22 4:00 PM	12.0	12.1	12.1	12.1
2022-09-22 5:00 PM	11.9	12.1	12.1	12.0
2022-09-22 6:00 PM	11.9	11.9	11.9	11.9
2022-09-22 7:00 PM	11.5	11.6	11.6	11.6
2022-09-22 8:00 PM	10.9	11.1	11.1	11.0
2022-09-22 9:00 PM	10.4	10.5	10.5	10.4
2022-09-22 10:00 PM	9.9	10.1	10.1	10.0
2022-09-22 11:00 PM	9.6	9.6	9.6	9.6
2022-09-23 12:00 AM	9.0	9.1	9.1	9.1
2022-09-23 1:00 AM	8.6	8.8	8.8	8.7
2022-09-23 2:00 AM	8.4	8.3	8.3	8.3

2022-09-23 3:00 AM	8.1	8.0	8.0	8.1
2022-09-23 4:00 AM	7.9	7.6	7.6	7.8
2022-09-23 5:00 AM	7.7	7.3	7.3	7.5
2022-09-23 6:00 AM	7.4	7.4	7.4	7.4
2022-09-23 7:00 AM	7.1	7.2	7.2	7.1
2022-09-23 8:00 AM	6.8	6.1	6.1	6.5
2022-09-23 9:00 AM	6.7	6.7	6.7	6.7
2022-09-23 10:00 AM	7.6	7.7	7.7	7.6
2022-09-23 11:00 AM	9.3	9.1	9.1	9.2
2022-09-23 12:00 PM	12.0	11.3	11.3	11.7
2022-09-23 1:00 PM	12.8	12.1	12.1	12.5
2022-09-23 2:00 PM	12.7	12.5	12.5	12.6
